# Dissecting Bonding Interactions in Cysteine Dimers

**DOI:** 10.3390/molecules27248665

**Published:** 2022-12-07

**Authors:** Santiago Gómez, Sara Gómez, Jorge David, Doris Guerra, Chiara Cappelli, Albeiro Restrepo

**Affiliations:** 1 Instituto de Química, Universidad de Antioquia UdeA, Calle 70 No. 52-21, Medellín 050010, Colombia; 2 Scuola Normale Superiore, Classe di Scienze, Piazza dei Cavalieri 7, 56126 Pisa, Italy; 3 Escuela de Ciencias y Humanidades, Departamento de Ciencias Básicas, Universidad Eafit, AA 3300, Medellín 050022, Colombia

**Keywords:** cysteine dimers, NBO, NCI, QTAIM, stochastic optimization, hydrogen bonding, salt bridges, non-covalent interactions

## Abstract

Neutral (*n*) and zwitterionic (*z*) forms of cysteine monomers are combined in this work to extensively explore the potential energy surfaces for the formation of cysteine dimers in aqueous environments represented by a continuum. A simulated annealing search followed by optimization and characterization of the candidate structures afforded a total of 746 structurally different dimers held together via 80 different types of intermolecular contacts in 2894 individual non-covalent interactions as concluded from Natural Bond Orbitals (NBO), Quantum Theory of Atoms in Molecules (QTAIM) and Non-Covalent Interactions (NCI) analyses. This large pool of interaction possibilities includes the traditional primary hydrogen bonds and salt bridges which actually dictate the structures of the dimers, as well as the less common secondary hydrogen bonds, exotic X⋯Y (X = C, N, O, S) contacts, and H⋯H dihydrogen bonds. These interactions are not homogeneous but have rather complex distributions of strengths, interfragment distances and overall stabilities. Judging by their Gibbs bonding energies, most of the structures located here are suitable for experimental detection at room conditions.

## 1. Introduction

Cysteine, HOOCCH(NH_2_)CH_2_SH is the only amino acid among the unique list of 20 found in proteins that possesses a thiol functional group [1]. This thiol group, which is comparatively a weaker Brønsted–Lowry acid than O–H in carboxylic acids, is extremely important in biochemistry. Among a large number of known functionalities, the S–H group is responsible for nucleophilic additions to α,β–unsaturated carbonyl compounds via Michael reactions [2,3,4,5], serves as a deprotonation agent [6], and its strong nucleophilicity renders cysteine a key component of the active sites of several protease enzymes [7,8]. In addition to S–H, depending on the conditions, sulfur atoms in cysteine engage in S–S disulfide bonds, which are a central element determining secondary and tertiary structure in proteins [9,10,11] and are relevant in physiological redox activity. According to the SwissProt databank [12], six percent of all proteins contain at least one disulfide bridge, and the median number of disulfide bonds is two.

Protein⋯protein interaction is one of the central problems in molecular biology. Unfortunately, with present days computational methods, a thorough understanding from a molecular perspective is unattainable because the number of explicit contacts grows exponentially with the size of the protein. For example, insulin is one of the smallest biologically active proteins containing a primary sequence of just 51 amino acids (six cysteines among them), for the dimer of this protein, not counting salt bridges and other intermolecular interactions, there are at least 6 $\times \;10^{4}$ possibilities for hydrogen bonding in the classical X${}^{\delta -}\cdots^{+\delta }$H–Y${}^{\delta -}$ description [13]. Typical proteins and other biomolecules contain in excess of 1000 amino acids and it is not uncommon to find very large proteins such as titin, which depending on the splice isoform, contains between 27,000 to 35,000 amino acids [14]; thus, the number of specific amino acid⋯amino acid contacts quickly becomes intractable.

In an effort to understand the intricacies of protein⋯protein interactions, the astonishingly large number of specific contacts calls for the use of reduced molecular models, often as gas phase isolated dimers of individual amino acids [13]. In this context, we attempt a detailed study of the cysteine dimers. This is a very complicated issue in its own right: First, there are the two enantiomeric forms of the amino acid. Second, there is the possibility of neutral and zwitterionic forms. Third, there is a large number of conformers of the monomer in a small energy window amenable to form dimers, which, for just the neutral form, has been estimated via ab initio computations to be 42, 51 or 71 depending on the sophistication of the model chemistry [1,9,15]. Notice that six well defined conformers have been experimentally identified via IR and MW spectroscopies [16,17]. Fourth, cysteine contains seven hydrogen atoms and four electronegative atoms; thus, ignoring salt bridges, dispersive dihydrogen interactions and other exotic contacts [13], from the classical X${}^{\delta -}\cdots^{+\delta }$H–Y${}^{\delta -}$ perspective, a total of 28 individual primary plus secondary hydrogen bonds are possible for each dimer. The number of possibilities is reduced to 20, distributed as 12 primary and 8 secondary HBs if the two H–C${}_{\beta }$ bonds are grouped into just one type and if the two N–H bonds are considered as another type. Fifth, as seen for example in alanine [13], several dimers are attached by more than one contact. Sixth, S–H leads to considerably weaker interactions than O–H, then, the potential energy surface (PES) for the dimers is expected to be considerably richer in weakly bound pairs and thus high levels of electron correlation are needed to correctly describe the intermolecular interactions. Seventh, the environment sensibly impacts the ability of biomolecules to interact in biological settings; thus, using gas phase dimers as a reduced model for protein⋯protein interactions does not seem enough and at least solvent effects must be included.

Cysteine has been thoroughly studied through experiments and computations. Besides the above mentioned publications dealing with the conformations of the monomer [1,9,15,16,17], Kaminski et al. [18] and Sadlej et al. [19] undertook somewhat exhaustive explorations of the conformational PES for the monomers to rationalize Raman Optical Activity (ROA) and Vibrational Circular Dichroism (VCD) spectra. For the dimers, early studies focused on gas phase and implicitly solvated models with limited explorations of the PES using a few hand constructed configurations [20], later studies considered both explicit water molecules and the neutral and zwitterionic forms [21,22]. There are reports dealing with the formation of the dimers, their stability and bonding (via density differences) when adsorbed in gold surfaces [23,24]. Group IA cations bonded to cysteine dimers have also been studied [25].

In view of the expected complexity arising from the multiple classical donor and acceptor hydrogen bonding sites of cysteine, which as a reduced molecular model has profound implications in the protein interaction problem, the brief summary of the scientific literature dealing with cysteine dimers just exposed reveals an unsatisfactory level of understanding not only of the potential energy surface but of the nature of the intermolecular bonding interactions for cysteine⋯cysteine. The present work is an attempt to remedy this situation. To that end, we undertake systematic explorations of the neutral (*n*) and zwitterionic (*z*) pairs in n⋯n, n⋯z and z⋯z combinations of low lying energy monomers via stochastic samplings of the corresponding PES, and dissected the nature of the interactions using formal quantum descriptors of bonding as provided by the Quantum Theory of Atoms in Molecules [26,27,28,29] (QTAIM), the Natural Bond Orbitals [30,31,32,33] (NBO) and the Non Covalent Interactions [34,35] (NCI), as discussed in the Section 2.

## 2. Computer Methods

Sampling the potential energy surfaces for all possible neutral (72 computed, 6 experimentally found) and zwitterionic forms (12 computed) is not only impossible but unnecessary under the premise that a few representative pairs would capture the vast majority of the specific contacts and thus would provide a sound picture applicable to all cases. Accordingly, we took two of the experimentally detected neutral monomers [17] ($n_{1},n_{2}$ in Table 1) and two of the computed lowest energy zwitterions [9] ($z_{1},z_{2}$ in Table 1) and exhausted all x(x−1)/2+x = 10 possible dimeric combinations for x = 4. Each pair was superimposed at the center of a cubic box of 512 Å${}^{3}$ (8 Å side) and was allowed to evolve under simulated annealing conditions [36,37,38] as implemented in the ASCEC program [39]. Superimposing the interacting system at the center of the box gives the algorithm the worst possible starting point (we call this the big bang initial conditions) and guarantees that the located stationary points within the corresponding PES are free of any structural bias. ASCEC [40,41], after its Spanish acronym Annealing Simulado Con Energía Cuántica, randomly explores the quantum energy landscape for the dimeric interaction, subjects the generated structures to a modified Metropolis acceptance test, and delivers a set of candidate structures that undergo further optimization via gradient following techniques and characterization as true minima via harmonic vibrational analysis. Each one of the 10 possible dimeric combinations was treated to duplicate ASCEC runs. All ASCEC runs and geometry optimizations were carried out in an aqueous environment represented by a continuum according to the PCM (ASCEC) and IEFPCM (optimization) models [42,43,44].

Final equilibrium geometries and Gibbs energies for every located dimer computed with the Gaussian09 suite of programs [45] are reported here using the dispersion corrected B3LYP–D3/6–311++G(d,p) model chemistry. Binding using the Gibbs energies at room conditions (1 atm, 298.16 K) are calculated as the negative difference between the energy of the cluster and the energy of the fragments, $BE\;=\;-(E_{cluster}-\sum E_{fragments})$, in this way, positive binding energies indicate strongly bonded clusters.

Once the molecular wavefunctions and electron densities for the optimized geometries are recovered by the procedure just stated, we use them to gain insight into the nature of intermolecular bonding interactions using the tools provided by QTAIM, NBO, and NCI following strategies described elsewhere [46,47,48,49,50,51,52,53]. At this point, we state that we use those methods as well established analysis tools, the interested reader is directed to the specialized literature for detailed discussions of their merits and shortcomings and for a description of how the calculated descriptors are related to bonding [6,33,49]. In short, use the Multiwfn suite [54] to find the bond critical points (BCPs, **r**${}_{c}$) corresponding to intermolecular interactions, analyze their properties, i.e., the electron density $\rho \left(r_{c}\right)$, its Laplacian $\nabla^{2}\rho \left(r_{c}\right)$, the total, kinetic, and potential energy densities $H\left(r_{c}\right)\;=\;G\left(r_{c}\right)\;+\;V\left(r_{c}\right)$, and the virial ratio $\left|V\left(r_{c}\right)\right|/G\left(r_{c}\right)$. With the same program, we calculate the Laplacian of the electron density, a scalar field that gives direct information about the most probable regions to find the electrons. Then, we use NBO6 [55] to pinpoint the specific orbitals involved in the intermolecular interactions associated with each BCP and estimate the strength of the interaction via second order perturbation energy for the interaction between the donor and acceptor orbitals, $E_{d\to a}^{\left(2\right)}$. The NCIPLOT program [56] was used to derive the non-covalent interaction surfaces. Jmol and VMD [57,58] were used to visualize the molecular structures, and their related surfaces and orbital interactions.

## 3. Results

Table 1 shows the structures of the $n_{1},n_{2}$ neutral and $z_{1},z_{2}$ zwitterionic forms chosen in this work to study the dimers of cysteine. The four non-covalent intramolecular interactions, as derived from QTAIM are displayed as dotted lines along with the involved NBOs. QTAIM and NBO descriptors are included as well. Only $n_{2}$ has a structure free from intramolecular hydrogen bonds while $z_{1}$ exhibits two intramolecular contacts. Except for $n_{1}$, all intramolecular interactions are characterized as weak, long range contacts because of the positive Laplacians, relatively small accumulation of electron densities at BCPs, virial ratios smaller than 1, and positive bond degree parameters. However, the $n_{N}\to \sigma_{O-H}^{*}$ interaction in $n_{1}$ is uncharacteristically strong, with values for the bonding descriptors that in every case surpass those of the archetypal hydrogen bond in the water dimer [30,59]. These intramolecular contacts are quite important because the formation of the dimers will usually involve investing energy to eliminate those interactions in favor of dimeric contacts. The electrostatic potentials in Figure S1 of the Supplementary Information show the blue and red regions which are more susceptible to the formation of intermolecular contacts according to the classic electrostatic view of hydrogen bonding.

### 3.1. Structures and Energies

**Figure 1:**
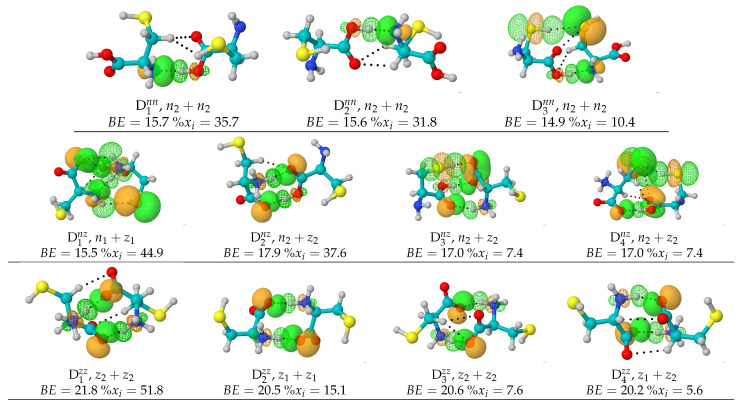
Lowest energy structures and the NBOs responsible for the strongest intermolecular interactions in the neutral n⋯n (top), neutral + zwitterionic n⋯z (middle) and zwitterionic z⋯z (bottom) B3LYP–D3/6–311++g(d,p) potential energy surfaces of the cysteine dimers under the continuum IEFPCM solvent model for water. Solid/meshed surfaces correspond to charge donor/acceptor orbitals, respectively. BE: binding energies in kcal/mol calculated using the Gibbs free energies at room conditions. See Table 1 for the structures of $n_{1},n_{2},z_{1},z_{2}$, the isolated monomers. Only those structures with populations (%$x_{i}$) higher than 5% within each PES are included. Energetics for the entire set of 746 dimers is provided in Tables S1–S3 of the supplementary material.

Complex and rich potential energy surfaces are uncovered by our stochastic searches in every case. A total of 746 distinct well defined dimers were located in the 10 monomer + monomer possible combinations. Table 2 lists the number of structural isomers for each PES and also shows that the vicinities of the putative global minima are populated with other close energy dimers; thus, all structures accounting for populations larger than 1% are within 2.1 kcal/mol of the n⋯n lowest energy structure, and so on. This point is emphasized by the results shown in Tables S1–S3 in the Supplementary information and in Figure 1, which clearly show that there are no dominant isomers.

Although 746 is a very large number of structures and is considerably higher than the numbers reported in any of the previous studies, we recognize that given the complexity of our problem, no stochastic or analytic search algorithm is able to locate all possible geometries. A representative set including only those dimers with populations exceeding 5% within each PES is shown in Figure 1, along with the NBOs responsible for the intermolecular interactions. Cartesian coordinates for all 746 structures located in this work are provided in the Supplementary Material.

**Figure 2:**
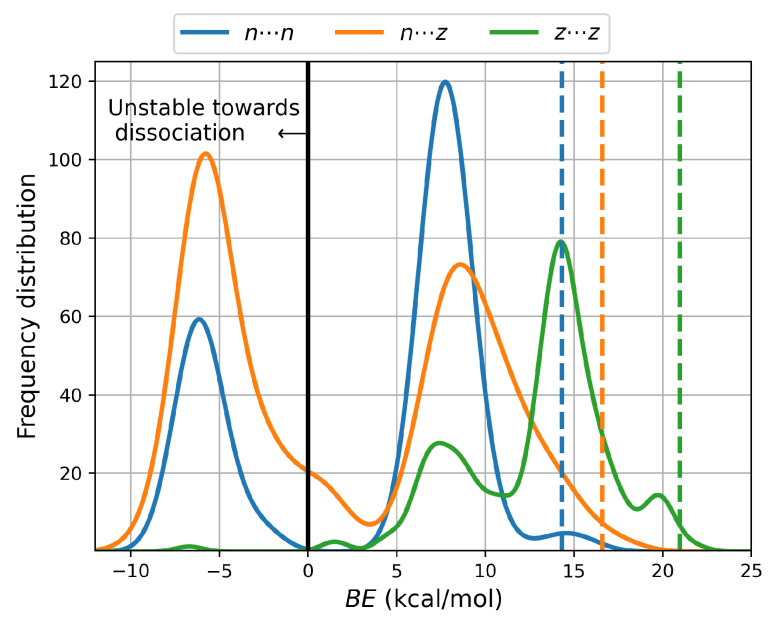
Distribution of binding energies of the cysteine dimers using the Gibbs energies. Dashed vertical lines mark the expected value for each potential energy surface using the Boltzmann populations at room conditions as weighing factors.

On the basis of purely ZPE–corrected electronic energies (Tables S1–S3), all cysteine dimers are stable towards fragmentation into the corresponding monomers, however, Figure 2 shows that consideration of temperature and entropy leads to 235 clusters (80 n⋯n, 154 n⋯z and 1 z⋯z) having negative binding energies as calculated from the Gibbs energies; thus, those particular structures correspond to unstable dimers and are not amenable to experimental detection at room conditions in aqueous environments, a fact that is emphasized by their $\%x_{i}\;\approx \;0$ populations. Notice the contrast with the 416 n⋯n gas phase equilibrium structures reported for the Alanine dimers [13], which are all strongly bonded. Binding energies show a clear $BE_{n\cdots n}\;<\;BE_{n\cdots z}\;<\;BE_{z\cdots z}$ ordering; thus, there is a marked preference for charged cysteine dimers in aqueous environments.

Figure 2, showing distribution plots of the Gibbs binding energies leads to a few relevant observations: Dashed vertical lines indicate the expected values of the binding energies using the Boltzmann populations of the Gibbs energies within each PES as weighing factors. 14.3, 16.6 and 20.9 kcal/mol are obtained for n⋯n, n⋯z, z⋯z, again showing a preference for charged dimers in aqueous environments. To put these binding energies in context, they are larger than the gas phase Gibbs binding energies of acetamide and acetic acid, which are 2.1 and 3.8 kcal/mol respectively, according to Copeland et al. [60] Notice that the same authors reported substantially higher binding energies when using only the ZPE-corrected electronic energies: 12.3 kcal/mol for acetamide and 14.7 kcal/mol for acetic acid. High ZPE-corrected binding energies have also been reported for the lowest energy structures in similar systems: 16.6 kcal/mol for the dimers of formic acid according to Kalescky et al. [61] and 12.7 according to Farfán et al. [62], 19.0 kcal/mol for the dimers of carbonic acid [63], and 20.9 kcal/mol for the alanine dimers [13]. Tables S1–S3 in the Supplementary material show exactly the same trend for all cysteine dimers calculated here, that is, comparatively much higher binding energies are obtained when only ZPE-corrected are considered with expected values of 25.9, 28.9, 33.7 kcal/mol for the n⋯n, n⋯z, and z⋯z cases, respectively. Notice that these numbers are up to over 6 times larger than the 5.0 kcal/mol binding energy arising because of a single hydrogen bond in the archetypal water dimer [59]. Finally, notice that those structures being unstable towards fragmentation (BE < 0) have minimal populations and thus do not contribute to the expected value of the binding energy. The role of dispersive interactions is clearly seen in the fact that when the D3 correction is removed from B3LYP, all strongly bound isomers become unstable towards fragmentation (values within parentheses in Tables S1–S3). For the cysteine dimers with positive binding energies, Tables S1–S3 show that the structures with the largest populations are strongly bonded.

As general structural features of the cysteine dimers, we point out that in all cases where neutral monomers are involved, $n_{2}$ (no intramolecular HB, Table 1) leads to lower energy dimers. Additionally, except for $D_{1}^{nz}$, in all structures that contain $n_{1}$, the intramolecular HB in $n_{1}$ remains in the dimer. A surprising result is that contrary to the well known structures of the dimers of carboxylic acids, out of the 244 well characterized n⋯n local minima, only two ($D_{5}^{nn},\%x_{i}=1.7$ and $D_{7}^{nn},\%x_{i}=1.3$) exhibit the traditional eight atom, cyclic double C=O⋯H–O stabilizing network. We attribute this to two factors: one, the influence of the solvent which favors other configurations, and two, the intramolecular hydrogen bond occupying the O–H bond in $n_{1}$ remains in all but one n⋯n dimer; thus, this bond is not available for intermolecular bonding (see the dissection of intermolecular bonding interactions below).

### 3.2. Bonding

The configurational space for the cysteine dimers is complex and rich. We located and characterized a total of 746 structures and there might as well be many more. This geometrical variety arises because of the large number of possible interactions discussed above. Our stochastic search and subsequent dissection of bonding interactions (see below) uncovers an astonishing total of 80 well characterized physically different types of direct intermolecular contacts listed in Table 3 and Table 4. Gratifyingly, the found structures account for every single one of the 20 possible hydrogen bonds among the monomers as exposed in the Introduction and also reveal additional salt bridges, dihydrogen bonds, and a number of exotic X⋯Y (X, Y = O, S, N, C) and C⋯H–C, C⋯H–S contacts. Notice that the n⋯n dimers exhibit a well balanced field of all non-charged interactions while the z⋯z dimers favor the salt bridges by a long shot (159 appearances) and, to a lesser extent, other interactions where only one of the fragments is charged. What should be clear is that the largest contributors to the stabilization of the dimers are N⋯H–O interactions in the n⋯n dimers, C=O${}^{-}\cdots$H–O in n⋯z, and C=O${}^{-}\cdots$H–N${}^{+}$ salt bridges in z⋯z. We think it is important to point out that, as a general rule, due to the comparatively larger interaction strength, it is the primary neutral, charged, or salt bridges forms of HBs that determine the molecular geometry of the dimers while secondary HBs and exotic contacts are a consequence of the structure (*vide infra*), however, the collective action of the multiple weak interactions on the stabilization energy of each cluster should not be ignored.

Our topological analysis of the electron densities of the 746 equilibrium structures located in this work affords a total of 2894 intermolecular contacts, which are collected into 80 different types in Table 3 and Table 4. Without a single exception, positive Laplacians at bond critical points (see Figure S2 in the supplementary material) indicate that bonding in the n⋯n, n⋯z and z⋯z cysteine dimers occurs via closed shell interactions, in the form of either ionic bonding or long range weak interactions. We dissect the nature of intermolecular interactions next.

#### 3.2.1. Interaction Distances

**Figure 3:**
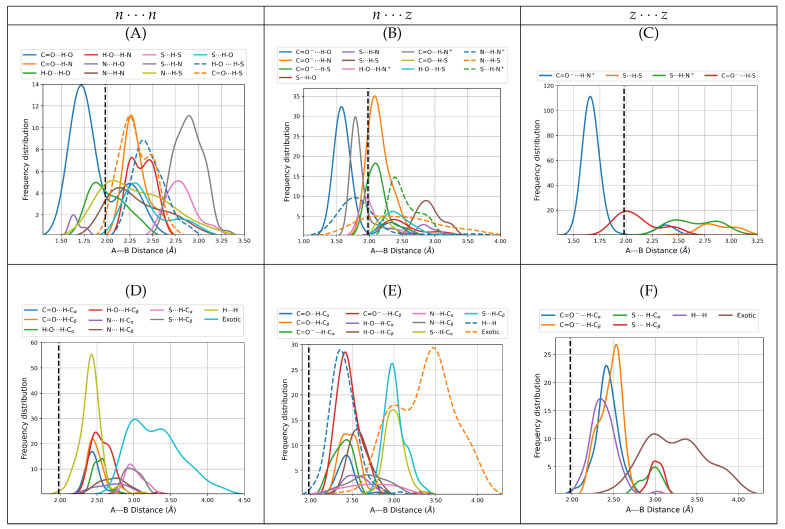
Distributions of the A⋯B distances for intermolecular contacts for all dimers of cysteine found in this work. The distributions are fitted to the actual histograms, so the center of the peaks of the distributions are statistically relevant. The top row shows only primary hydrogen bonds including salt bridges. The bottom row shows secondary hydrogen bonds, dihydrogen bonds, and all exotic interactions. The left column is reserved for the n⋯n dimers (subfigures (**A**,**D**)), the middle column for n⋯z (subfigures (**B**,**E**)) and the right column for z⋯z (subfigures (**C**,**F**)). All distances taken from the B3LYP–D3/6–311++G(d,p) potential energy surfaces with water represented as a continuum solvent. The dashed vertical lines at 1.98 Å mark the reference H⋯O distance in the gas phase water dimer.

Figure 3 shows the distribution of the distances associated with individual intermolecular contacts separated by interaction type, that is, primary and secondary hydrogen bonds, dihydrogen bonds, and exotic contacts for all dimers. Remarkably, the spectrum of A⋯B distances for direct intermolecular contacts covers a wide range, from the very short (1.50 Å for a C=O${}^{-}\cdots$H–O in $D_{38}^{nz}$) to the very large (4.19 Å for the exotic S⋯S in $D_{77}^{nn}$), which sensibly departs in both directions from the reference 1.98 Å in the isolated gas phase water dimer. Notice that regardless of the constituting monomers, only primary hydrogen bonds fall below 1.98 Å. In a classical sense, a zwitterion may be conveniently seen as two remote charge islands within the same molecule, in this view, the effect of the charges in the structural complexity of the cysteine dimers is clear: on one hand, intermolecular distances are reduced for the dimers with more charge islands, i.e., $r_{AB}^{nn}\;>\;r_{AB}^{nz}\;>\;r_{AB}^{zz}$, on the other, the structural complexity is also sensibly reduced for the more charge-separated species because the n⋯n distributions have more peaks than n⋯z which in turn have more peaks than z⋯z. In addition, it may be argued that among all the types of interactions stabilizing the cysteine dimers, salt bridges should be the strongest and thus the most important structural determining factor whenever they occur. Indeed, the lowest energy z⋯z dimers with populations larger than 5% shown in Figure 1, are actually stabilized by two salt bridges. Notice that the center of the peak for the distribution of C=O${}^{-}\cdots$H-N${}^{+}$ distances (1.66 Å, Figure 3C) is actually larger than 1.57 Å, the center of the peak for the distribution of C=O${}^{-}\cdots$H-O interactions (Figure 3B), which are *a priori* not as strong as the salt bridges but which dictate the structures of the n⋯z dimers, the reason for this apparent contradiction is that formation of the two salt bridges confers structural rigidity to the clusters.

**Figure 4:**
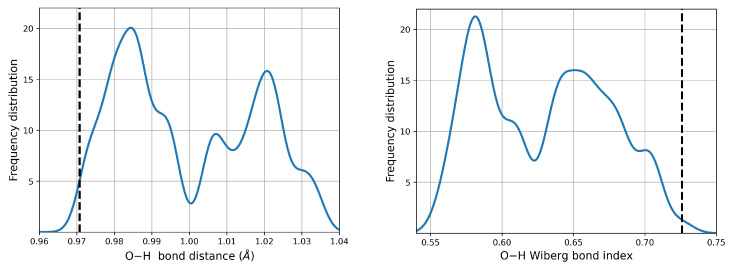
Variation of the O–H bond properties as a consequence of intermolecular interactions. The reference values for the $n_{2}$ cysteine monomer are included as vertical dashed lines.

When immersed in a continuum aqueous environment, there is partial dissociation of the O–H bonds upon the formation of the dimers. Figure 4 shows the changes in the corresponding distances and Wiberg Bond Indices (WBI) compared against the reference monomer. Evidence for partial dissociation is provided by the peak centered at ≈0.58 WBI, which actually corresponds to O–H groups of the low energy, high population dimers where neutral monomers are involved.

#### 3.2.2. Electron Densities at Bond Critical Points, $\rho (r_{c})$

**Figure 5:**
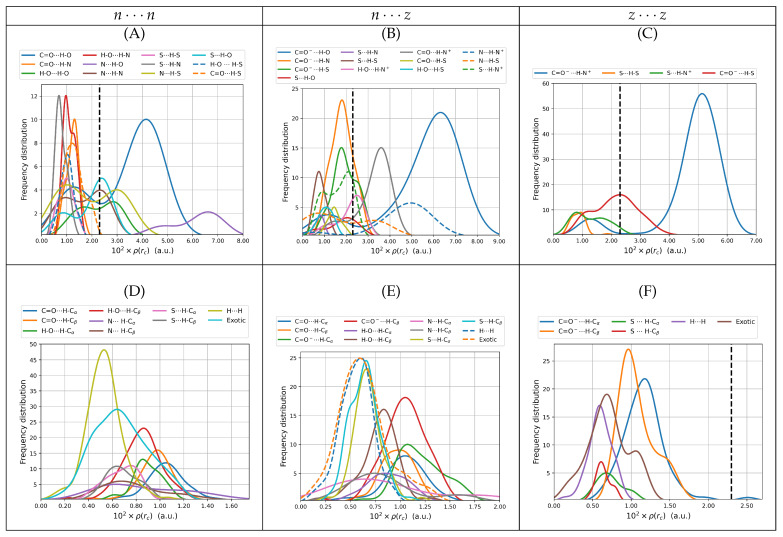
Electron densities at bond critical points for intermolecular contacts for all dimers of cysteine found in this work. The top row shows only primary hydrogen bonds including salt bridges. The bottom row shows secondary hydrogen bonds, dihydrogen bonds, and all exotic interactions. The left column is reserved for the n⋯n dimers (subfigures (**A**,**D**)), the middle column for n⋯z (subfigures (**B**,**E**)) and the right column for z⋯z (subfigures (**C**,**F**)). All values taken from the B3LYP–D3/6–311++G(d,p) potential energy surfaces with water represented as a continuum solvent. The dashed vertical lines mark the reference value for the gas phase water dimer.

The relationship between electron density at bond critical points and the nature of the interaction is clear: large accumulations of electron densities at BCPs indicate that the electrons are shared between two fragments or atoms, otherwise known as covalent bonding. Conversely, small electron densities at BCPs indicate that the electrons are displaced towards the nuclei, thus signaling either ionic bonding or long range interactions. Figure 5 shows the values for the calculated electron densities at the 2894 bond critical points associated to intermolecular interactions in the 746 cysteine dimers. Electron densities at those points cover the $\left[9.1\times 10^{-4},7.6\times 10^{-2}\right]$ a.u. interval. These values are sensibly smaller than the 0.24 and 0.35 a.u obtained for the covalent C–C and O–H bonds in $D_{1}^{nn}$. The smaller electron densities correspond to secondary HBs and exotic contacts while among primary HBs, those with the smallest densities involve the S–H group. Only some primary HBs and salt bridges have larger densities than the reference water dimer. The distributions plotted in Figure 5 are wide; thus, there are many possibilities for the same type of interaction. Finally, as expected [49,64,65,66], there seems to be an inverse correlation between interaction distance and electron density at intermolecular BCPs.

#### 3.2.3. Bond Degree Parameters $H\left(r_{c}\right)/\rho \left(r_{c}\right)$

The bond degree parameter is related to chemical bonding as follows. Kinetic energy is everywhere positive and repulsive ($mv^{2}/2=p^{2}/2m>0$ in classical mechanics) while potential energy is everywhere negative and attractive. The total energy is the sum of kinetic and potential energies, H = G + V; thus, its sign reveals the winner of the local kinetic vs potential energy tug of war and dictates the nature of the interaction. Indeed, positive total energies at BCPs are obtained when there is a local dominance of the repulsive kinetic energy, indicating local depletion of electrons in the internuclear region and displacement of the electron density associated with the particular bonding interactions towards the nuclei. Conversely, negative total energies are obtained when there is a local dominance of the attractive potential energy indicating that there is shared electron density concentrated in the internuclear region and signaling an increasingly covalent character of the interaction. An alternative rigorous physical meaning to energy densities is offered by a dimensional analysis: energy density has units of pressure (E/V = F/A = P); thus, local negative energy densities may be equated to negative quantum pressures which strongly attract electrons towards the BCP, indicating increasingly covalent interactions while local positive energy densities correspond to positive quantum pressures that push electrons away from the BCPs towards the nuclei, indicating anionic or long range interactions.

**Figure 6:**
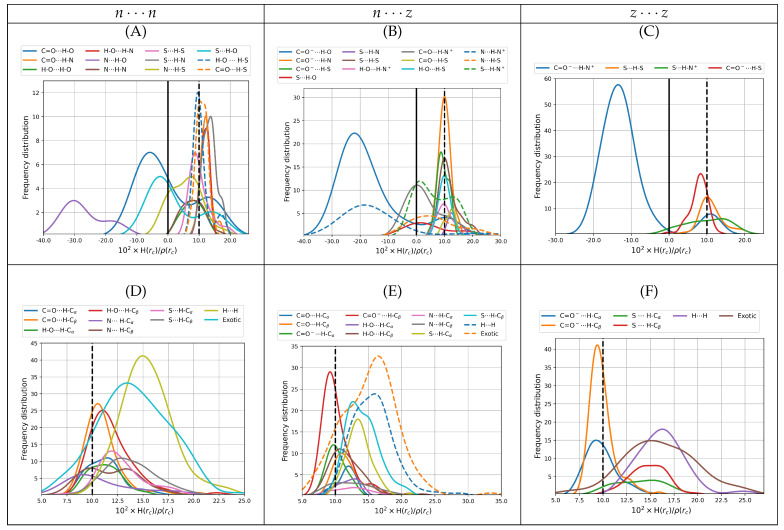
Bond degree parameters for intermolecular contacts for all dimers of cysteine found in this work. The top row shows only primary hydrogen bonds including salt bridges. The bottom row shows secondary hydrogen bonds, dihydrogen bonds, and all exotic interactions. The left column is reserved for the n⋯n dimers (subfigures (**A**,**D**)), the middle column for n⋯z (subfigures (**B**,**E**)) and the right column for z⋯z (subfigures (**C**,**F**)). All values taken from the B3LYP–D3/6–311++G(d,p) potential energy surfaces with water represented as a continuum solvent. Solid vertical lines mark the QTAIM boundaries separating locally stabilizing from the locally destabilizing interactions. Dashed vertical lines mark the reference value for the gas phase water dimer.

It is well known that the sign of $\nabla^{2}\rho \left(r_{c}\right)$ is not a sufficient criterium to establish the nature of the interaction in every case [28,67,68,69,70], specifically, it is quite often the case that a particular interaction has both positive Laplacian and negative bond degree parameter at the same time. Thus, the bond degree parameter is used in conjunction with the Laplacian of the electron density at BCPs to remove any ambiguity according to Rozas et al. [68]: weak to medium strength hydrogen bonds have both $\nabla^{2}\rho \left(r_{c}\right),H\left(r_{c}\right)/\rho \left(r_{c}\right)\;>\;0$, strong hydrogen bonds have $\nabla^{2}\rho \left(r_{c}\right)\;>\;0,H\left(r_{c}\right)/\rho \left(r_{c}\right)\;<\;0$ and very strong HBs have both $\nabla^{2}\rho \left(r_{c}\right),H\left(r_{c}\right)/\rho \left(r_{c}\right)\;<\;0$. Figure 6 plots distributions of the bond degree parameters for all dimers found in this work. It is clear from the distributions of $H\left(r_{c}\right)/\rho \left(r_{c}\right)$ that all intermolecular contacts found here cover a wide spectrum of possibilities with a substantial number of only primary hydrogen bonds or salt bridges having $H\left(r_{c}\right)/\rho \left(r_{c}\right)\;<\;0$ (Figure 6A–C), thus should be considered as strong contacts by the above criteria. The wide spectrum of bond degree parameters, the large number of structural possibilities and the strong character of the interactions have deep implications in the biological role of cysteine and of the aminoacids that make up proteins and biomolecules: similar results have been obtained for example in the interactions between the spike protein of SARS-COV-2 and the ACE2 receptors [48,71] and between the envelope protein of the Zika virus and the glycosaminoglycans that act as receptors [47]. In the case of SARS-COV-2, the formation of strong salt bridges and hydrogen bonds is one of the main factors of the pressure driving the evolution of the virus towards new variants. For the cysteine dimers, many of the primary hydrogen bonds with positive bond degree parameters are located to the left of the reference isolated gas phase water dimer, which confers them medium to strong character. All secondary and exotic contacts (Figure 6D–F) exhibit positive bond degree parameters and many areas actually to the right of the reference water dimer; thus, they are classified as weak. As a general rule, hydrogen bonds involving the carbonyl, carboxylate and amino groups as electron donors and the hydroxyl, amino and ammonium groups as electron acceptors, are the ones with highly negative $H\left(r_{c}\right)/\rho \left(r_{c}\right)$ values. Some HBs involving the thiol group, either as donor or acceptor, have slightly negative bond degree parameters.

#### 3.2.4. Virial Ratios, $\left|V(r_{c})\right|/G\left(r_{c}\right)$

Analysis of the virial ratios at bond critical points serves as a more quantitative description of the nature of the interactions than the Laplacians of the electron density and the bond degree parameters. See the works of Grabowski [28] and of Rozas et al. [68] for a formal description of how the virial ratio is related to bonding. In short, local depletion of electron density (local dominance of the repulsive kinetic energy), which is indicative of ionic or long interactions have $0\;<\;\left|V(r_{c})\right|/G\left(r_{c}\right)\;<\;1$, local concentration of electron density (local dominance of the attractive potential energy), indicative of covalent interactions have $\left|V(r_{c})\right|/G\left(r_{c}\right)\;>\;2$, and the $1\;<\;\left|V(r_{c})\right|/G\left(r_{c}\right)\;<\;2$ interval describes interactions with mixed contributions.

**Figure 7:**
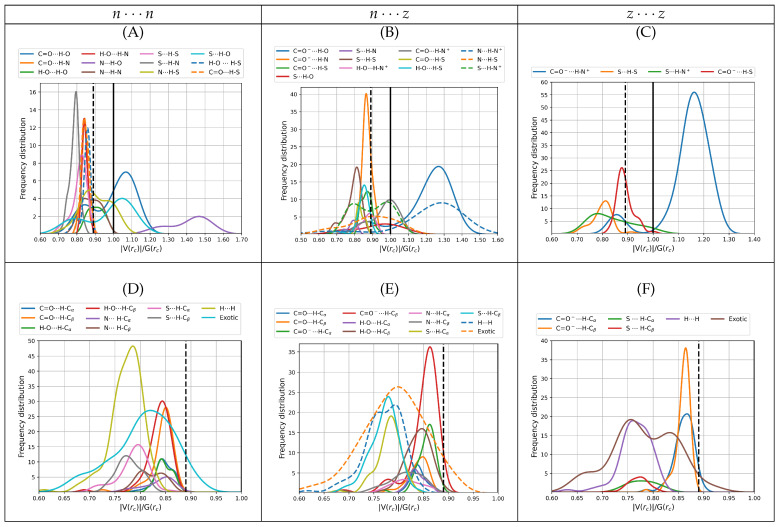
Virial ratios at bond critical points for intermolecular contacts for all dimers of cysteine found in this work. The top row shows only primary hydrogen bonds including salt bridges. The bottom row shows secondary hydrogen bonds, dihydrogen bonds, and all exotic interactions. The left column is reserved for the n⋯n dimers (subfigures (**A**,**D**)), the middle column for n⋯z (subfigures (**B**,**E**)) and the right column for z⋯z (subfigures (**C**,**F**)). All values taken from the B3LYP–D3/6–311++G(d,p) potential energy surfaces with water represented as a continuum solvent. Vertical solid lines mark the QTAIM boundaries separating long range from intermediate character interactions. Dashed vertical lines mark the reference value for the gas phase water dimer.

Figure 7 shows the distribution of the virial ratios for all dimers found in this work, which cover the [0.61, 1.49] interval for primary hydrogen bonds and salt bridges (Figure 7A–C) and the [0.56, 0.92] interval for secondary HBs, exotic and dihydrogen contacts (Figure 7A–C). Notice that as in the analysis of the previous descriptors, the fitted distributions go a little beyond the actual limits. It is quite revealing that a large number of contacts, especially those involving the carbonyl group have virial ratios larger than 1, which confers them a high degree of covalency while not being formal bonds. Interestingly, these include the charged carboxylate which may naively be thought as being involved in highly ionic contacts. Virial ratios larger than 1 transcend the carbonyl group, which is indeed the case for the following HBs: N⋯H–O, C=O⋯H–O, S⋯H–O, N⋯H–S for n⋯n dimers, C=O${}^{-}\cdots$H–O, N⋯H–N${}^{+}$, S⋯H–N${}^{+}$, C=O ⋯H–N${}^{+}$ for n⋯z and for all salt bridges in z⋯z. This high covalency of the *a priori* ionic contacts has been reported for other cases, including for example the microsolvation of charged species [49,53,72]. Most secondary HBs, H⋯H, and exotic contacts have virials smaller than the water dimer reference. Surprisingly, the thiol group in a number of cases is involved in stronger interactions than the H–O⋯H–O and N⋯H–O contacts.

#### 3.2.5. NBO and NCI Picture of Intermolecular Interactions

Intermolecular interactions have been successfully studied under the NBO formalism in a wide range of problems [30,33]. In the particular case of the cysteine dimers, we proceeded to identify the localized donor Lewis orbitals from which charge is transferred to acceptor orbitals according to the $\phi_{d}\to \phi_{a}$ scheme. Table 3 and Table 4 list the involved orbitals for each one of the 80 types of interactions found in this work, Figure 1 provides the corresponding surfaces for those dimers with populations larger than 5%. Once identified, we quantified the strength of the orbital interaction by second order perturbation theory on the Fock matrix as given by $-E_{d\to a}^{\left(2\right)}\;=\;q_{d}{\left|<\phi_{d}|F|\phi_{a}>\right|}^{2}/\left(E_{a}-E_{d}\right)$. With this procedure, the strength of the interaction is directly related to the magnitude of $E_{d\to a}^{\left(2\right)}$.

**Figure 8:**
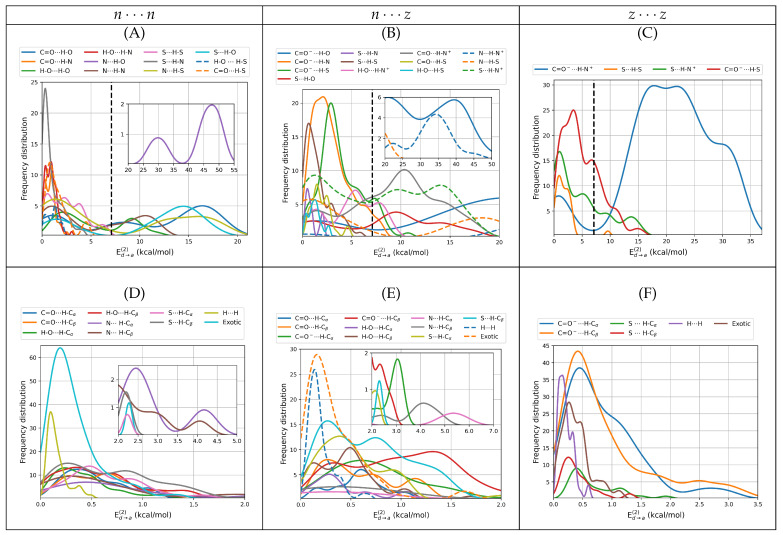
Donor ⋯ acceptor NBO energies for intermolecular contacts for all dimers of cysteine found in this work. The top row shows only primary hydrogen bonds including salt bridges. The bottom row shows secondary hydrogen bonds, dihydrogen bonds, and all exotic interactions. The left column is reserved for the n⋯n dimers (subfigures (**A**,**D**)), the middle column for n⋯z (subfigures (**B**,**E**)) and the right column for z⋯z (subfigures (**C**,**F**)). All values taken from the B3LYP–D3/6–311++G(d,p) potential energy surfaces with water represented as a continuum solvent. The dashed vertical lines mark the reference value for the gas phase water dimer.

Donor → acceptor orbital interactions resulting in primary hydrogen bonds and salt bridges in n⋯n, n⋯z and z⋯z cysteine dimers include everything from the very weak to the very strong, covering the wide [0.06, 50.30] kcal/mol interval (Figure 8A–C). Salt bridges exhibit an uncommonly complex distribution of energies with several shoulders. Interestingly, the strongest contacts are not salt bridges but rather N⋯H–O primary hydrogen bonds, listed as interaction **10** in Table 3 and shown in Figure 8A. This interaction type, which is also the strongest intermolecular contact found in alanine dimers [13], arises from $n_{N}\to \sigma_{H-O}^{*}$ charge transfer in n⋯n dimers. Next in the strength hierarchy are the highly ionic C=O${}^{-}\cdots$H–O and N⋯H–N${}^{+}$ contacts arising from $n_{O}\to \sigma_{H-O}^{*}$ and $n_{N}\to \sigma_{H-N}^{*}$ charge transfers in n⋯z dimers. These are listed as interactions **2, 12** with the corresponding distributions shown in Figure 8B. C=O${}^{-}\cdots$H–N${}^{+}$ salt bridges in z⋯z dimers come only third in the interaction energy scale. They arise from $n_{O}\to \sigma_{H-N}^{*}$ charge transfer, are listed as interaction **6** and the corresponding distributions are shown in Figure 8C. These sets of interactions are present on the structures with populations higher than 5%. In a manner consistent with the QTAIM descriptors analyzed above, a large number of primary HBs and salt bridges have interaction energies larger than 6.63 [30] kcal/mol, the orbital interaction energy for the reference water dimer, however, no secondary hydrogen bond, no exotic contact and no dihydrogen bond exceed the reference.

As stated above, primary HBs and salt bridges determine the structure of the dimers. According to Table 3, they always arise from orbital interactions of the $n_{X}\to \sigma_{H-Y}^{*}$ type with X, Y = O, N, S. As also stated above, secondary HBs, dihydrogen bonds and exotic contacts are usually a consequence of the structure. Notwithstanding, the orbitals involved in the weaker interactions offer a quite interesting and rather uncommon picture. First, notice that all exotic O⋯O contacts (**53–58** in Table 4) put the two negative ends of the fragments with various degrees of negative character in direct contact, with the most severe case being interaction **54** connecting two formal negative charges with no intermediaries. Second, notice that the 139 interactions grouped into **58, 61–63, 65, 71, 76, 78–80** may all be described by the general $n_{X}\to \sigma_{Y-H}^{*}$ charge transfer scheme with X, Y = O, N, S. These correspond to what David et al. [13] have called inverted hydrogen bonds because the lone pair on X donates electron charge to an antibonding $\sigma_{Y-H}^{*}$ orbital which is inverted from the usual $\sigma_{H-Y}^{*}$, in other words, the charge donation occurs between two orbitals overlapping from one negative atom to another negative atom with no bridging proton. Anti electrostatic hydrogen bonds of the type described in this paragraph have been reported by Weinhold and Klein [73].

**Figure 9:**
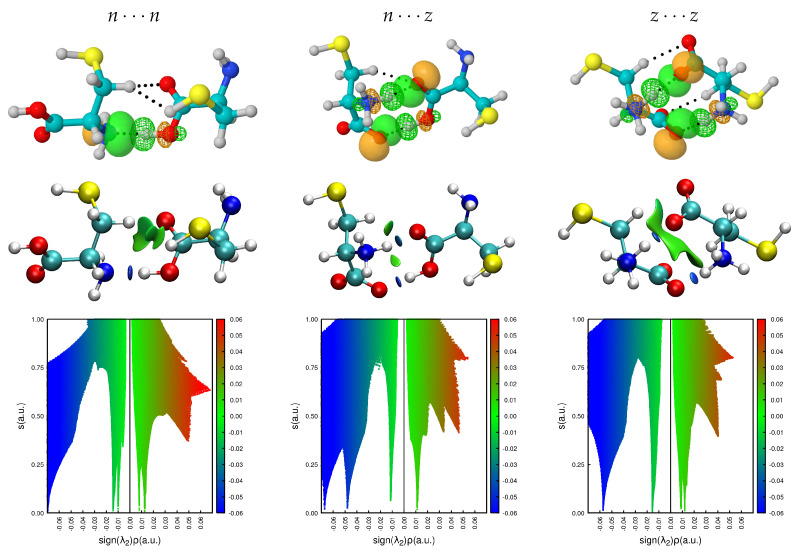
NCI surfaces and thoroughs describing the intermolecular contacts for the highest binding energy cysteine dimers. NBO pictures are also included to ease visualization of the interactions.

Figure 9 shows the obtained non covalent surfaces as well as the thoroughs of the reduced gradients for the largest binding energy dimers in the n⋯n, n⋯z, z⋯z potential energy surfaces, we include the corresponding interacting NBOs to help visualize the source of the NCI surfaces. See Figures S3–S5 in the supplementary material for the corresponding surfaces in all dimers with populations larger than 5%. The standard NCI color code [34,35] assigns green surfaces to weakly bonding contacts and blue surfaces to strong interactions, for the thoroughs, negative values of sign$(\lambda_{2})\rho$ reveal bonding interactions [34] which are weaker when sign$(\lambda_{2})\rho \approx 0$. NCI reveals that the two salt bridges in the z⋯z dimers are actually very similar (in fact, they cannot be told apart in the thoroughs) and that in most cases, large stabilizing surfaces arising from individual contacts transferring tiny amounts of charge to the interstitial region have significant contributions to the overall binding of the dimers. Unexpectedly, these charge transfer contributions are major contributors to the charged cases. Notice that charge transfer to the interstitial region appears to be the norm when several molecular units are stabilized via non covalent interactions: these fluxional surfaces of charge have been found to be a major player in the molecular interpretation of hydrophobicity [46], in the initial recognition and attachment of viruses to cell receptors [47,48,71], in the microsolvation and encapsulation of charged and neutral species, in the microscopic structure of ionic liquids [51], etc.

## 4. Discussion and Context

Accurate description and characterization of chemical bonding is a notoriously hard problem in chemistry, whose difficulty is magnified when dealing with weak intermolecular non covalent interactions. When studying molecules and their interactions, a large portion of the conceptual framework developed by experimentalists and theoreticians invokes a number of useful ideas that correspond to non observable quantities (partial atom charges, orbital interactions, virial ratios at BCPs, etc.); thus, there are no quantum mechanical operators whose expected values may be used to calculate them and therefore, approximate methods, however accurate, are used to determine these quantities. This approach has a fundamental problem: each quantity may be obtained by several methods and the results quite often vary among them. In this context, it is impressive and certainly reassuring that QTAIM, NBO and NCI, which are conceptually and methodologically substantially different, afford a consistent, complementary picture of intermolecular bonding in the dimers of cysteine.

The large number of interactions, isomers, and types of binary contacts are intimately connected to the problem of molecular evolution and to the complexity of life observable on this planet. By virtue of the large number of accessible states, molecular systems where specific cysteine to cysteine contacts are observed are thermodynamically favored because of the ever increasing entropy of the universe, in other words, this type of systems will evolve towards equilibrium states with large structural diversity. Since the interactions dissected here are responsible for the molecular interactions between all aminoacid pairs, this argument of entropy driving molecular evolution readily applies to large proteins and biomolecules.

## 5. Summary and Conclusions

An intensive exploration of the potential energy surfaces for the interaction of neutral and charged cysteine monomers to form dimers in an aqueous environment represented by a continuum afforded a large number of isomers, amounting to 746 well characterized local minima. The isomers with the largest population are distributed within small energy differences of the putative global minima. Ten potential energy surfaces were explored in total for the n⋯n, n⋯z, and z⋯z combinations with two neutral (*n*) and two zwitterionic (*z*) forms. A number of strongly bound dimers were found, with interaction energies exceeding 20 kcal/mol in several cases and with interaction distances covering the very small to the very large in the [1.50, 4.19] Å interval. The nature of intermolecular bonding interactions was dissected using QTAIM, NCI, and NBO, three conceptually different methods, which for the present case afford consistent, complementary pictures. A total of 80 types of different intermolecular contacts were found in this complex and large universe of dimers. As a general rule, primary hydrogen bonds and salt bridges are the strongest of the interactions and determine the molecular geometry, conversely, secondary hydrogen bonds, exotic X⋯Y (X = C, N, O, S) and H⋯H dihydrogen contacts are weaker and most often a consequence of the structure. All interactions, even the highly ionic, may be described by the $\phi_{d}\to \phi_{a}$ orbital charge transfer scheme, leading to accumulation or depletion of electron density at the bond critical points as revealed by topological analysis of the electron densities. The large binding energies mentioned above are the result of unusually strong charge assisted hydrogen bonds and salt bridges. We found that the highly ionic salt bridges have large degrees of covalency; thus, a simplistic electrostatic attraction between positively and negatively charged fragments does not suffice for a proper account of bonding interactions whenever the zwitterions are involved. The weaker secondary hydrogen bonds, exotic X⋯Y and dihydrogen contacts in no few cases are stronger than, for example, the archetypal hydrogen bond in the water dimer. Moreover, independent of how strong or weak individual interactions are, their collective action cannot be ignored because they lead to the formation of large attractive non-covalent surfaces in the interstitial region between the fragments. A few antielectrostatic contacts [73] as well as a few inverted hydrogen bonds [13] in which the charge transfer occurs between the two negative ends of the fragments, were found; thus, they appear to be of common occurrence in nature.

## Figures and Tables

**Table 1:** Structures of the B3LYP–D3/6–311++G(d,p) monomers of neutral ($n_{1},n_{2}$) and zwitterionic ($z_{1},z_{2}$) L–cysteine. In each case, ΔΔG are the corresponding differences in Gibbs energies at room conditions with respect to $n_{1},z_{1}$, the lowest energy monomers. Descriptors of intramolecular bonding derived from QTAIM and NBO are included along with the specific NBO orbitals responsible for the interactions. $\Delta \Delta G,E_{d\to a}^{\left(2\right)}$ in kcal mol${}^{-1}$, all other descriptors in a.u. $n_{1}$ and $n_{2}$ have been experimentally detected [17].

Monomer →	$n_{1}$	$n_{2}$	$z_{1}$		$z_{2}$
**Properties ↓**	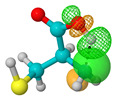	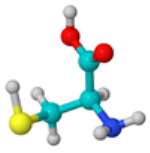	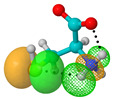	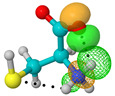	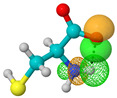
ΔΔG	0.0	2.2	0.0	0.0	0.3
A⋯B Distance (Å)	1.86		2.63	1.91	1.94
$10^{2}\times \rho \left(r_{c}\right)$	4.07		1.37	3.29	3.11
$10^{2}\times \nabla^{2}\rho \left(r_{c}\right)$	10.87		4.63	11.78	11.29
$\left|V(r_{c})\right|/G\left(r_{c}\right)$	1.12		0.82	0.93	0.92
$10^{2}\times H\left(r_{c}\right)/\rho \left(r_{c}\right)$	−9.18		12.73	5.62	7.04
$\phi_{d}\to \phi_{a}$	$n_{N}\to \sigma_{O-H}^{*}$		$n_{S}\to \sigma_{N-H}^{*}$	$n_{O}\to \sigma_{N-H}^{*}$	$n_{O}\to \sigma_{N-H}^{*}$
$E_{d\to a}^{\left(2\right)}$	14.9		2.3	8.3	7.3

**Table 2:** Summary of structural and energetical properties of the cysteine dimers. ΔG range: Gibbs energy difference between the highest and lowest energy structure in kcal/mol. %$x_{i}$: isomer populations listed in Tables S1–S3 in the Supplementary Material.

Composition	Structures	ΔG Range	
		for $\%x_{i}\;>\;1$	for All Structures
n⋯n	244	2.1	21.4
n⋯z	316	1.1	25.5
z⋯z	186	2.3	27.9

**Table 3:** Properties of the 20 types of primary hydrogen bonds, 10 types of secondary hydrogen bonds, and 12 types of dihydrogen bonds stabilizing the cysteine dimers. $N_{i}^{A\cdots B}$ is the number of times that the interaction appears in the corresponding type of dimers. $\phi_{d},\phi_{a}$ are the charge donor and acceptor orbitals as identified from NBO. An example of a dimer containing each particular interaction is given in the rightmost column.

Label	Type	$N_{i}^{A\cdots B}$			$\phi_{d}\to \phi_{a}$	Example
		n⋯n	z⋯z	n⋯z		
**Primary hydrogen bonds**						
**1**	C=O⋯H–O	22	-	-	$n_{O}\to \sigma_{H-O}^{*}$	D${}_{5}^{nn}$
**2**	C=O${}^{-}$⋯H–O	-	-	52	$n_{O}\to \sigma_{H-O}^{*}$	D${}_{38}^{nz}$
**3**	C=O⋯H–N	46	-	-	$n_{O}\to \sigma_{H-N}^{*}$	D${}_{119}^{nn}$
**4**	C=O⋯H–N${}^{+}$	-	-	51	$n_{O}\to \sigma_{H-N}^{*}$	D${}_{2}^{nz}$
**5**	C=O${}^{-}$⋯H–N	-	-	89	$n_{O}\to \sigma_{H-N}^{*}$	D${}_{55}^{nz}$
**6**	C=O${}^{-}$⋯H–N${}^{+}$	-	159	-	$n_{O}\to \sigma_{H-N}^{*}$	D${}_{16}^{zz}$
**7**	H–O⋯H–O	18	-	-	$n_{O}\to \sigma_{H-O}^{*}$	D${}_{172}^{nn}$
**8**	H–O⋯H–N	51	-	-	$n_{O}\to \sigma_{H-N}^{*}$	D${}_{137}^{nn}$
**9**	H–O⋯H–N${}^{+}$	-	-	32	$n_{O}\to \sigma_{H-N}^{*}$	D${}_{207}^{nz}$
**10**	N⋯H–O	7	-	-	$n_{N}\to \sigma_{H-O}^{*}$	D${}_{1}^{nn}$
**11**	N⋯H–N	19	-	-	$n_{N}\to \sigma_{H-N}^{*}$	D${}_{174}^{nn}$
**12**	N⋯H–N${}^{+}$	-	-	19	$n_{N}\to \sigma_{H-N}^{*}$	D${}_{1}^{nz}$
**13**	S⋯H–S	29	30	54	$n_{S}\to \sigma_{H-S}^{*}$	D${}_{61}^{nn}$
**14**	S⋯H–N	55	-	19	$n_{S}\to \sigma_{H-N}^{*}$	D${}_{51}^{nn}$
**15**	S⋯H–N${}^{+}$	-	41	52	$n_{S}\to \sigma_{H-N}^{*}$	D${}_{175}^{zz}$
**16**	N⋯H–S	19	-	14	$n_{N}\to \sigma_{H-S}^{*}$	D${}_{108}^{nn}$
**17**	S⋯H–O	16	-	12	$n_{S}\to \sigma_{H-O}^{*}$	D${}_{107}^{nn}$
**18**	H–O⋯H–S	34	-	29	$n_{O}\to \sigma_{H-S}^{*}$	D${}_{8}^{nn}$
**19**	C=O⋯H–S	49	-	27	$n_{O}\to \sigma_{H-S}^{*}$	D${}_{18}^{nn}$
**20**	C=O${}^{-}$⋯H–S	-	85	68	$n_{O}\to \sigma_{H-S}^{*}$	D${}_{142}^{zz}$
**Secondary hydrogen bonds**						
**21**	C=O⋯H–C${}_{\alpha }$	49	-	26	$n_{O}\to \sigma_{H-C}^{*}$	D${}_{46}^{nn}$
**22**	C=O⋯H–C${}_{\beta }$	57	-	44	$n_{O}\to \sigma_{H-C}^{*}$	D${}_{37}^{nn}$
**23**	C=O${}^{-}$⋯H–C${}_{\alpha }$	-	68	40	$n_{O}\to \sigma_{H-C}^{*}$	D${}_{145}^{zz}$
**24**	C=O${}^{-}$⋯H–C${}_{\beta }$	-	108	71	$n_{O}\to \sigma_{H-C}^{*}$	D${}_{144}^{zz}$
**25**	H–O⋯H–C${}_{\alpha }$	48	-	20	$n_{O}\to \sigma_{H-C}^{*}$	D${}_{144}^{nn}$
**26**	H–O⋯H–C${}_{\beta }$	71	-	41	$n_{O}\to \sigma_{H-C}^{*}$	D${}_{156}^{nn}$
**27**	N⋯H–C${}_{\alpha }$	16	-	11	$n_{N}\to \sigma_{H-C}^{*}$	D${}_{188}^{nn}$
**28**	N⋯H–C${}_{\beta }$	34	-	19	$n_{N}\to \sigma_{H-C}^{*}$	D${}_{186}^{nn}$
**29**	S⋯H–C${}_{\alpha }$	52	24	57	$n_{S}\to \sigma_{H-C}^{*}$	D${}_{39}^{nn}$
**30**	S⋯H–C${}_{\beta }$	59	37	81	$n_{S}\to \sigma_{H-C}^{*}$	D${}_{44}^{nn}$
**Dihydrogen contacts**						
**31**	C${}_{\alpha }$–H⋯H–N	7	-	3	$\sigma_{C-H}\to \sigma_{H-N}^{*}$	D${}_{135}^{nn}$
**32**	C${}_{\beta }$–H⋯H–N	13	-	3	$\sigma_{C-H}\to \sigma_{H-N}^{*}$	D${}_{86}^{nn}$
**33**	C${}_{\beta }$–H⋯H–N${}^{+}$	-	-	1	$\sigma_{C-H}\to \sigma_{H-N}^{*}$	D${}_{139}^{nz}$
**34**	N–H⋯H–N	3	-	-	$\sigma_{N-H}\to \sigma_{H-N}^{*}$	D${}_{99}^{nn}$
**35**	C${}_{\alpha }$–H⋯H–C${}_{\alpha }$	4	3	-	$\sigma_{C-H}\to \sigma_{H-C}^{*}$	D${}_{152}^{nn}$
**36**	C${}_{\beta }$–H⋯H–C${}_{\beta }$	42	15	23	$\sigma_{C-H}\to \sigma_{H-C}^{*}$	D${}_{78}^{nn}$
**37**	C${}_{\alpha }$–H⋯H–C${}_{\beta }$	27	20	22	$\sigma_{C-H}\to \sigma_{H-C}^{*}$	D${}_{64}^{nn}$
**38**	S–H⋯H–S	5	3	3	$\sigma_{S-H}\to \sigma_{H-S}^{*}$	D${}_{62}^{nn}$
**39**	S–H⋯H–N	5	-	3	$\sigma_{S-H}\to \sigma_{H-N}^{*}$	D${}_{61}^{nn}$
**40**	S–H⋯H–N${}^{+}$	-	1	-	$\sigma_{S-H}\to \sigma_{H-N}^{*}$	D${}_{53}^{zz}$
**41**	S–H⋯H–C${}_{\alpha }$	15	8	12	$\sigma_{S-H}\to \sigma_{H-C}^{*}$	D${}_{159}^{nn}$
**42**	S–H⋯H–C${}_{\beta }$	23	14	21	$\sigma_{S-H}\to \sigma_{H-C}^{*}$	D${}_{157}^{nn}$

**Table 4:** Properties of the “exotic” intermolecular contacts found in the cysteine dimers. $N_{i}^{A\cdots B}$ is the number of times that the interaction appears in the corresponding type of dimers. $\phi_{d},\phi_{a}$ are the charge donor and acceptor orbitals as identified from NBO. An example of a dimer containing each particular interaction is given in the rightmost column.

Label	Type	$N_{i}^{A\cdots B}$			$\phi_{d}\to \phi_{a}$	Example
		n⋯n	z⋯z	n⋯z		
**O⋯C contacts**						
**43**	C=O⋯C=O	6	-	-	$n_{O}\to \pi_{C=O}^{*}$	D${}_{113}^{nn}$
**44**	C=O⋯C=O${}^{-}$	-	-	4	$n_{O}\to \pi_{C=O}^{*}$	D${}_{6}^{nz}$
**45**	C=O${}^{-}$⋯C=O	-	-	5	$n_{O}\to \pi_{C=O}^{*}$	D${}_{90}^{nz}$
**46**	C=O${}^{-}$⋯C=O${}^{-}$	-	7	-	$n_{O}\to \pi_{C=O}^{*}$	D${}_{14}^{zz}$
**47**	C=O⋯C${}_{\alpha }$–C	5	-	-	$\pi_{C=O}\to \sigma_{C-C}^{*}$	D${}_{112}^{nn}$
**48**	C=O${}^{-}$⋯C${}_{\alpha }$–C	-	-	1	$n_{O}\to \sigma_{C-C}^{*}$	D${}_{125}^{nz}$
**49**	C=O⋯C${}_{\beta }$–S	2	-	2	$n_{O}\to \sigma_{C-S}^{*}$	D${}_{25}^{nn}$
**50**	C=O${}^{-}$⋯C${}_{\beta }$–S	-	2	2	$n_{O}\to \sigma_{C-S}^{*}$	D${}_{132}^{zz}$
**51**	H–O⋯C=O	4	-	-	$n_{O}\to \pi_{C=O}^{*}$	D${}_{161}^{nn}$
**52**	H–O⋯C${}_{\beta }$–S	2	-	1	$n_{O}\to \sigma_{C-S}^{*}$	D${}_{80}^{nn}$
**O⋯O contacts**						
**53**	C=O⋯O=C	14	-	-	$\pi_{C=O}\to \pi_{O=C}^{*}$	D${}_{102}^{nn}$
**54**	C=O${}^{-}$⋯${}^{-}$O=C	-	17	-	$n_{O}\to \pi_{O=C}^{*}$	D${}_{10}^{zz}$
**55**	C=O${}^{-}$⋯O=C	-	-	14	$n_{O}\to \pi_{O=C}^{*}$	D${}_{23}^{nz}$
**56**	C=O⋯O–H	26	-	-	$n_{O}\to \sigma_{O-H}^{*}$	D${}_{230}^{nn}$
**57**	C=O${}^{-}$⋯O–H	-	-	13	$n_{O}\to \sigma_{O-H}^{*}$	D${}_{217}^{nz}$
**58**	H–O⋯O–H	11	-	-	$n_{O}\to \sigma_{O-H}^{*}$	D${}_{45}^{nn}$
**O⋯N contacts**						
**59**	N⋯O=C	2	-	-	$n_{N}\to \pi_{O=C}^{*}$	D${}_{11}^{nn}$
**60**	N⋯O–H	2	-	-	$n_{N}\to \sigma_{O-H}^{*}$	D${}_{35}^{nn}$
**61**	C=O${}^{-}$⋯${}^{+}$N–H	-	16	-	$n_{O}\to \sigma_{N-H}^{*}$	D${}_{117}^{zz}$
**62**	C=O⋯${}^{+}$N–H	-	-	3	$n_{O}\to \sigma_{N-H}^{*}$	D${}_{12}^{nz}$
**63**	H-O⋯${}^{+}$N–H	-	-	1	$n_{O}\to \sigma_{N-H}^{*}$	D${}_{15}^{nz}$
**N⋯C contacts**						
**64**	N⋯C=O	3	-	-	$n_{N}\to \pi_{C=O}^{*}$	D${}_{130}^{nn}$
**N⋯N contacts**						
**65**	N⋯N–H	2	-	-	$n_{N}\to \sigma_{N-H}^{*}$	D${}_{173}^{nn}$
**C⋯H contacts**						
**66**	H–C${}_{\beta }$⋯H–C${}_{\alpha }$	1	2	2	$\sigma_{H-C}\to \sigma_{H-C}^{*}$	D${}_{70}^{nn}$
**67**	H–C${}_{\beta }$⋯H–C${}_{\beta }$	2	2	2	$\sigma_{H-C}\to \sigma_{H-C}^{*}$	D${}_{205}^{nn}$
**68**	H–C${}_{\beta }$⋯H–S	2	1	3	$\sigma_{H-C}\to \sigma_{H-S}^{*}$	D${}_{105}^{nn}$
**69**	${}^{-}$O=C⋯H–C${}_{\alpha }$	-	1	-	$\pi_{O=C}\to \sigma_{H-C}^{*}$	D${}_{32}^{zz}$
**70**	${}^{-}$O=C⋯H–C${}_{\beta }$	-	6	1	$\pi_{O=C}\to \sigma_{H-C}^{*}$	D${}_{20}^{zz}$
**S⋯S contacts**						
**71**	S⋯S–H	12	7	11	$n_{S}\to \sigma_{S-H}^{*}$	D${}_{71}^{nn}$
**S⋯C contacts**						
**72**	S⋯C=O	7	-	2	$n_{S}\to \pi_{C=O}^{*}$	D${}_{135}^{nn}$
**73**	S⋯C=O${}^{-}$	-	12	2	$n_{S}\to \pi_{C=O}^{*}$	D${}_{113}^{zz}$
**74**	H–C${}_{\alpha }\cdots$S–H	-	1	-	$\sigma_{H-C}\to \sigma_{S-H}^{*}$	D${}_{109}^{zz}$
**75**	H–C${}_{\beta }$⋯ S–H	-	1	-	$\sigma_{H-C}\to \sigma_{S-H}^{*}$	D${}_{110}^{zz}$
**S⋯O contacts**						
**76**	C=O⋯S–H	10	-	10	$n_{O}\to \sigma_{S-H}^{*}$	D${}_{127}^{nn}$
**77**	C=O${}^{-}$⋯S–H	-	27	11	$\pi_{C=O}\to \sigma_{S-H}^{*}$	D${}_{51}^{zz}$
**78**	S⋯O–H	26	-	12	$n_{S}\to \sigma_{O-H}^{*}$	D${}_{148}^{nn}$
**S⋯N contacts**						
**79**	N⋯S–H	9	-	3	$n_{N}\to \sigma_{S-H}^{*}$	D${}_{173}^{nn}$
**80**	S⋯${}^{+}$N–H	-	3	3	$n_{S}\to \sigma_{N-H}^{*}$	D${}_{178}^{zz}$

## Data Availability

All data are available within the article or Supplementary Materials.

## Supplementary Materials

The following supporting information can be downloaded at: https://www.mdpi.com/article/10.3390/molecules27248665/s1, Figure S1: Electrostatic potential surfaces for the cysteine monomers.; Figure S2: Distributions of the Laplacians of the electron densities at bond critical points for all dimers.; Figures S3–S5: Additional NBO and NCI plots of descriptors of bonding interactions for the dimers with populations larger than 5%; Table S1: Binding energies and energy differences for neutral dimers.; Table S2: Binding energies and energy differences for mixed dimers. Table S3: Binding energies and energy differences for zwitterionic dimers. Cartesian coordinates for the entire set of 746 dimers are also provided.
