# Supplementary material for: Dissecting Bonding Interactions in Cysteine Dimers
Source: Molecules. 2022 Dec 7;27(24):8665. doi: 10.3390/molecules27248665 (PMC9786917; doi:10.3390/molecules27248665)
Supplement: Supplementary file 1 [file molecules-27-08665-s001.zip › molecules-2059908-supplementary.pdf]

Dissecting Bonding Interactions in Cysteine Dimers

Santiago Gómez [1], Sara Gómez [2], Jorge David [3], Doris Guerra [1],
Chiara Cappelli [2,*] and Albeiro Restrepo [1,*]

1 Instituto de Química, Universidad de Antioquia UdeA, Calle 70 No. 52-21, Medellín 050010, Colombia

2 Scuola Normale Superiore, Classe di Scienze, Piazza dei Cavalieri 7, 56126 Pisa, Italy

3 Escuela de Ciencias y Humanidades, Departamento de Ciencias Básicas, Universidad Eafit, AA 3300, Medellín 050022, Colombia

* Correspondence: chiara.cappelli@sns.it (C.C.); albeiro.restrepo@udea.edu.co (A.R.)

Supplementary information

1 Electrostatic potential of the cysteine monomers

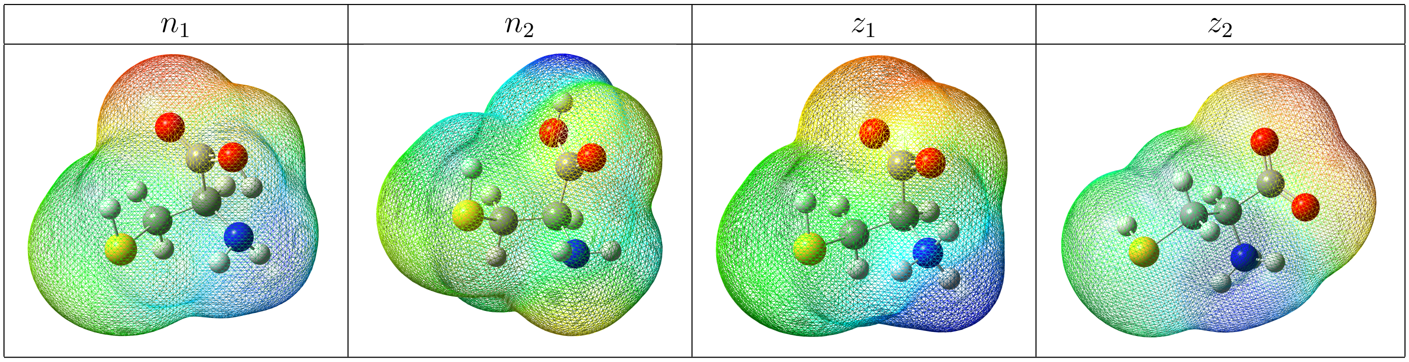

Figure S1: Electrostatic potential surfaces for the cysteine monomers.

2 Energetics for cysteine dimers

Table S1: Binding energies and energy differences in kcal mol^{-1} for neutral dimers. ΔΔG: Gibbs energies relative to the global minimum. We list electronic ($BE_{B3LYP-D3}$) and Gibbs (BE_{Gibbs}) binding energies corrected by the B3LYP–D3 vibrational ZPE (values within parentheses are calculated for the bare B3LYP functional with no dispersion corrections). Isomer populations $\%\chi_i(G)$ derived from Boltzmann distributions of the Gibbs energies are also reported.

**Dimer**	Composition	$\mathbf{BE}_{B3LYP-D3}$	$\mathbf{BE}_{Gibbs}$	**ΔΔG**	$\%\chi_i(G))$
D_1^{nn}	$n_2 + n_2$	27.1	15.7 (-2.4)	≈0.0	35.7
D_2^{nn}	$n_2 + n_2$	27.0	15.6 (-1.4)	0.1	31.8
D_3^{nn}	$n_2 + n_2$	27.3	14.9 (-2.1)	0.7	10.4
D_4^{nn}	$n_2 + n_2$	26.8	14.2	1.5	3.1
D_5^{nn}	$n_2 + n_2$	26.3	13.9	1.8	1.7
D_6^{nn}	$n_1 + n_1$	20.3	9.3	1.9	1.3
D_7^{nn}	$n_2 + n_2$	26.3	13.7	2.0	1.3
D_8^{nn}	$n_1 + n_1$	19.0	9.2	2.0	1.2
D_9^{nn}	$n_1 + n_1$	20.3	9.1	2.1	1.0
D_{10}^{nn}	$n_1 + n_1$	20.4	9.0	2.2	0.9
D_{11}^{nn}	$n_1 + n_1$	20.7	9.0	2.2	0.8
D_{12}^{nn}	$n_1 + n_1$	20.6	8.9	2.3	0.7
D_{13}^{nn}	$n_1 + n_1$	20.5	8.8	2.4	0.6
D_{14}^{nn}	$n_1 + n_1$	20.5	8.8	2.4	0.6
D_{15}^{nn}	$n_1 + n_1$	20.5	8.8	2.5	0.6
D_{16}^{nn}	$n_1 + n_1$	20.5	8.7	2.6	0.5
D_{17}^{nn}	$n_1 + n_1$	20.2	8.7	2.6	0.5

**Dimer**	Composition	$\mathbf{BE}_{\mathrm{B3LYP-D3}}$	$\mathbf{BE}_{\mathrm{Gibbs}}$	$\Delta\Delta$**G**	$\%\chi_i(G))$
D_{18}^{nn}	$n_1 + n_1$	20.4	8.6	2.7	0.4
D_{19}^{nn}	$n_1 + n_1$	20.4	8.6	2.7	0.4
D_{20}^{nn}	$n_1 + n_1$	19.9	8.5	2.7	0.4
D_{21}^{nn}	$n_1 + n_1$	20.2	8.5	2.7	0.4
D_{22}^{nn}	$n_1 + n_1$	20.7	8.4	2.8	0.3
D_{23}^{nn}	$n_1 + n_1$	20.0	8.3	3.0	0.2
D_{24}^{nn}	$n_1 + n_1$	19.2	8.2	3.0	0.2
D_{25}^{nn}	$n_1 + n_1$	18.8	8.2	3.0	0.2
D_{26}^{nn}	$n_1 + n_1$	20.3	8.2	3.0	0.2
D_{27}^{nn}	$n_1 + n_1$	18.8	8.2	3.0	0.2
D_{28}^{nn}	$n_1 + n_1$	19.8	8.1	3.1	0.2
D_{29}^{nn}	$n_1 + n_1$	18.3	8.1	3.2	0.2
D_{30}^{nn}	$n_1 + n_1$	20.0	8.1	3.2	0.2
D_{31}^{nn}	$n_1 + n_1$	19.1	8.1	3.2	0.2
D_{32}^{nn}	$n_1 + n_1$	19.4	8.0	3.2	0.2
D_{33}^{nn}	$n_1 + n_1$	19.3	8.0	3.2	0.2
D_{34}^{nn}	$n_1 + n_1$	19.7	8.0	3.2	0.2
D_{35}^{nn}	$n_1 + n_1$	19.4	8.0	3.2	0.2
D_{36}^{nn}	$n_1 + n_1$	19.6	7.9	3.3	0.1
D_{37}^{nn}	$n_1 + n_1$	19.0	7.9	3.4	0.1
D_{38}^{nn}	$n_1 + n_1$	19.7	7.8	3.4	0.1
D_{39}^{nn}	$n_1 + n_1$	19.7	7.8	3.4	0.1
D_{40}^{nn}	$n_1 + n_1$	18.8	7.8	3.4	0.1
D_{41}^{nn}	$n_1 + n_1$	18.8	7.8	3.5	0.1
D_{42}^{nn}	$n_1 + n_1$	19.1	7.8	3.5	0.1
D_{43}^{nn}	$n_1 + n_1$	19.5	7.7	3.5	0.1
D_{44}^{nn}	$n_1 + n_1$	19.5	7.7	3.5	0.1
D_{45}^{nn}	$n_1 + n_1$	20.3	7.7	3.6	0.1
D_{46}^{nn}	$n_1 + n_1$	17.9	7.7	3.6	0.1
D_{47}^{nn}	$n_1 + n_1$	20.0	7.6	3.6	0.1
D_{48}^{nn}	$n_1 + n_1$	20.0	7.6	3.6	0.1
D_{49}^{nn}	$n_1 + n_1$	19.5	7.6	3.6	0.1
D_{50}^{nn}	$n_1 + n_1$	19.6	7.6	3.7	0.1
D_{51}^{nn}	$n_1 + n_1$	19.9	7.5	3.7	0.1
D_{52}^{nn}	$n_1 + n_1$	19.2	7.5	3.7	0.1

**Dimer**	Composition	$\mathbf{BE}_{\mathrm{B3LYP-D3}}$	$\mathbf{BE}_{\mathrm{Gibbs}}$	$\Delta\Delta$**G**	$\%\chi_i(G))$
D^{nn}_{53}	$n_1 + n_1$	18.3	7.5	3.7	0.1
D^{nn}_{54}	$n_1 + n_1$	19.5	7.5	3.8	0.1
D^{nn}_{55}	$n_1 + n_1$	19.1	7.5	3.8	0.1
D^{nn}_{56}	$n_1 + n_1$	18.7	7.4	3.8	0.1
D^{nn}_{57}	$n_1 + n_1$	18.6	7.4	3.8	0.1
D^{nn}_{58}	$n_1 + n_1$	18.9	7.4	3.8	0.1
D^{nn}_{59}	$n_1 + n_1$	19.5	7.4	3.9	0.1
D^{nn}_{60}	$n_1 + n_1$	19.5	7.4	3.9	0.1
D^{nn}_{61}	$n_1 + n_1$	19.4	7.3	3.9	0.1
D^{nn}_{62}	$n_1 + n_1$	19.2	7.2	4.0	≈0.0
D^{nn}_{63}	$n_1 + n_1$	18.8	7.2	4.0	≈0.0
D^{nn}_{64}	$n_1 + n_1$	17.9	7.2	4.0	≈0.0
D^{nn}_{65}	$n_1 + n_1$	17.4	7.1	4.1	≈0.0
D^{nn}_{66}	$n_1 + n_1$	17.3	7.1	4.1	≈0.0
D^{nn}_{67}	$n_1 + n_1$	17.8	7.1	4.2	≈0.0
D^{nn}_{68}	$n_2 + n_2$	24.1	11.5	4.2	≈0.0
D^{nn}_{69}	$n_1 + n_1$	18.0	7.0	4.2	≈0.0
D^{nn}_{70}	$n_1 + n_1$	18.0	7.0	4.2	≈0.0
D^{nn}_{71}	$n_1 + n_1$	17.7	7.0	4.2	≈0.0
D^{nn}_{72}	$n_1 + n_1$	17.3	7.0	4.3	≈0.0
D^{nn}_{73}	$n_1 + n_1$	18.4	6.9	4.3	≈0.0
D^{nn}_{74}	$n_1 + n_1$	18.0	6.9	4.3	≈0.0
D^{nn}_{75}	$n_1 + n_1$	18.0	6.9	4.4	≈0.0
D^{nn}_{76}	$n_1 + n_1$	17.4	6.9	4.4	≈0.0
D^{nn}_{77}	$n_1 + n_1$	18.9	6.9	4.4	≈0.0
D^{nn}_{78}	$n_1 + n_1$	18.8	6.8	4.4	≈0.0
D^{nn}_{79}	$n_1 + n_1$	18.0	6.8	4.4	≈0.0
D^{nn}_{80}	$n_1 + n_1$	18.2	6.8	4.4	≈0.0
D^{nn}_{81}	$n_1 + n_1$	17.1	6.7	4.5	≈0.0
D^{nn}_{82}	$n_1 + n_1$	18.9	6.7	4.6	≈0.0
D^{nn}_{83}	$n_1 + n_1$	17.9	6.5	4.8	≈0.0
D^{nn}_{84}	$n_1 + n_1$	16.6	6.4	4.8	≈0.0
D^{nn}_{85}	$n_1 + n_1$	17.5	6.4	4.8	≈0.0
D^{nn}_{86}	$n_1 + n_1$	17.7	6.3	4.9	≈0.0
D^{nn}_{87}	$n_1 + n_1$	18.0	6.3	5.0	≈0.0

**Dimer**	Composition	$\mathbf{BE}_{\text{B3LYP-D3}}$	$\mathbf{BE}_{\text{Gibbs}}$	$\Delta\Delta$**G**	$\%\chi_i(G))$
D_{88}^{nn}	$n_2 + n_2$	22.3	10.7	5.0	≈0.0
D_{89}^{nn}	$n_1 + n_1$	17.0	6.2	5.0	≈0.0
D_{90}^{nn}	$n_1 + n_1$	17.3	6.2	5.0	≈0.0
D_{91}^{nn}	$n_2 + n_2$	21.4	10.6	5.1	≈0.0
D_{92}^{nn}	$n_1 + n_1$	16.4	6.1	5.1	≈0.0
D_{93}^{nn}	$n_1 + n_1$	17.1	6.0	5.2	≈0.0
D_{94}^{nn}	$n_1 + n_1$	16.9	6.0	5.2	≈0.0
D_{95}^{nn}	$n_1 + n_1$	16.8	5.8	5.4	≈0.0
D_{96}^{nn}	$n_2 + n_2$	22.6	9.9	5.7	≈0.0
D_{97}^{nn}	$n_1 + n_1$	16.2	5.4	5.8	≈0.0
D_{98}^{nn}	$n_2 + n_2$	22.4	9.7	5.9	≈0.0
D_{99}^{nn}	$n_2 + n_2$	20.8	9.5	6.2	≈0.0
D_{100}^{nn}	$n_2 + n_2$	22.6	9.3	6.3	≈0.0
D_{101}^{nn}	$n_2 + n_2$	20.8	9.3	6.4	≈0.0
D_{102}^{nn}	$n_2 + n_2$	23.2	9.1	6.5	≈0.0
D_{103}^{nn}	$n_2 + n_2$	20.4	9.1	6.5	≈0.0
D_{104}^{nn}	$n_2 + n_2$	20.4	9.0	6.6	≈0.0
D_{105}^{nn}	$n_2 + n_2$	20.6	9.0	6.6	≈0.0
D_{106}^{nn}	$n_2 + n_2$	20.6	9.0	6.7	≈0.0
D_{107}^{nn}	$n_2 + n_2$	18.6	9.0	6.7	≈0.0
D_{108}^{nn}	$n_2 + n_2$	20.7	8.8	6.8	≈0.0
D_{109}^{nn}	$n_2 + n_2$	19.8	8.8	6.8	≈0.0
D_{110}^{nn}	$n_2 + n_2$	19.8	8.8	6.8	≈0.0
D_{111}^{nn}	$n_2 + n_2$	19.8	8.8	6.9	≈0.0
D_{112}^{nn}	$n_2 + n_2$	21.6	8.8	6.9	≈0.0
D_{113}^{nn}	$n_2 + n_2$	21.9	8.7	7.0	≈0.0
D_{114}^{nn}	$n_2 + n_2$	21.2	8.7	7.0	≈0.0
D_{115}^{nn}	$n_2 + n_2$	19.9	8.6	7.1	≈0.0
D_{116}^{nn}	$n_2 + n_2$	19.4	8.5	7.2	≈0.0
D_{117}^{nn}	$n_2 + n_2$	19.8	8.5	7.2	≈0.0
D_{118}^{nn}	$n_2 + n_2$	21.5	8.5	7.2	≈0.0
D_{119}^{nn}	$n_2 + n_2$	18.7	8.3	7.3	≈0.0
D_{120}^{nn}	$n_2 + n_2$	21.4	8.3	7.3	≈0.0
D_{121}^{nn}	$n_2 + n_2$	21.0	8.3	7.4	≈0.0
D_{122}^{nn}	$n_2 + n_2$	18.8	8.3	7.4	≈0.0

**Dimer**	Composition	$\mathbf{BE}_{\mathrm{B3LYP-D3}}$	$\mathbf{BE}_{\mathrm{Gibbs}}$	$\Delta\Delta\mathbf{G}$	$\%\chi_i(G))$
D^{nn}_{123}	$n_2 + n_2$	20.6	8.2	7.5	≈0.0
D^{nn}_{124}	$n_2 + n_2$	19.4	8.1	7.5	≈0.0
D^{nn}_{125}	$n_2 + n_2$	19.5	8.1	7.6	≈0.0
D^{nn}_{126}	$n_2 + n_2$	20.9	8.0	7.6	≈0.0
D^{nn}_{127}	$n_2 + n_2$	20.3	8.0	7.6	≈0.0
D^{nn}_{128}	$n_2 + n_2$	20.6	8.0	7.7	≈0.0
D^{nn}_{129}	$n_2 + n_2$	20.3	7.9	7.7	≈0.0
D^{nn}_{130}	$n_2 + n_2$	20.1	7.9	7.8	≈0.0
D^{nn}_{131}	$n_2 + n_2$	20.4	7.9	7.8	≈0.0
D^{nn}_{132}	$n_2 + n_2$	20.5	7.8	7.9	≈0.0
D^{nn}_{133}	$n_2 + n_2$	20.4	7.8	7.9	≈0.0
D^{nn}_{134}	$n_2 + n_2$	17.3	7.8	7.9	≈0.0
D^{nn}_{135}	$n_2 + n_2$	19.5	7.8	7.9	≈0.0
D^{nn}_{136}	$n_2 + n_2$	18.2	7.7	8.0	≈0.0
D^{nn}_{137}	$n_2 + n_2$	18.5	7.6	8.0	≈0.0
D^{nn}_{138}	$n_2 + n_2$	18.3	7.6	8.1	≈0.0
D^{nn}_{139}	$n_2 + n_2$	18.9	7.5	8.2	≈0.0
D^{nn}_{140}	$n_2 + n_2$	18.7	7.5	8.2	≈0.0
D^{nn}_{141}	$n_2 + n_2$	20.6	7.5	8.2	≈0.0
D^{nn}_{142}	$n_2 + n_2$	17.6	7.5	8.2	≈0.0
D^{nn}_{143}	$n_2 + n_2$	17.3	7.5	8.2	≈0.0
D^{nn}_{144}	$n_2 + n_2$	17.7	7.4	8.2	≈0.0
D^{nn}_{145}	$n_2 + n_2$	18.7	7.4	8.2	≈0.0
D^{nn}_{146}	$n_2 + n_2$	19.4	7.4	8.2	≈0.0
D^{nn}_{147}	$n_2 + n_2$	17.7	7.4	8.2	≈0.0
D^{nn}_{148}	$n_2 + n_2$	19.7	7.4	8.3	≈0.0
D^{nn}_{149}	$n_2 + n_2$	18.6	7.4	8.3	≈0.0
D^{nn}_{150}	$n_2 + n_2$	18.1	7.4	8.3	≈0.0
D^{nn}_{151}	$n_2 + n_2$	18.0	7.3	8.4	≈0.0
D^{nn}_{152}	$n_2 + n_2$	18.2	7.3	8.4	≈0.0
D^{nn}_{153}	$n_2 + n_2$	18.1	7.3	8.4	≈0.0
D^{nn}_{154}	$n_2 + n_2$	19.9	7.3	8.4	≈0.0
D^{nn}_{155}	$n_2 + n_2$	18.0	7.2	8.5	≈0.0
D^{nn}_{156}	$n_2 + n_2$	18.1	7.1	8.5	≈0.0
D^{nn}_{157}	$n_2 + n_2$	18.7	7.0	8.6	≈0.0

**Dimer**	Composition	$\mathbf{BE}_{\text{B3LYP-D3}}$	$\mathbf{BE}_{\text{Gibbs}}$	$\Delta\Delta$**G**	$\%\chi_i(G))$
D^{nn}_{158}	$n_2 + n_2$	17.4	7.0	8.7	≈0.0
D^{nn}_{159}	$n_2 + n_2$	18.8	6.9	8.7	≈0.0
D^{nn}_{160}	$n_2 + n_2$	17.0	6.8	8.8	≈0.0
D^{nn}_{161}	$n_2 + n_2$	19.1	6.8	8.9	≈0.0
D^{nn}_{162}	$n_2 + n_2$	17.9	6.7	8.9	≈0.0
D^{nn}_{163}	$n_2 + n_2$	17.0	6.5	9.1	≈0.0
D^{nn}_{164}	$n_2 + n_2$	18.1	6.4	9.2	≈0.0
D^{nn}_{165}	$n_1 + n_2$	7.2	-2.3	15.7	≈0.0
D^{nn}_{166}	$n_1 + n_2$	9.0	-2.3	15.7	≈0.0
D^{nn}_{167}	$n_1 + n_2$	8.1	-2.6	16.0	≈0.0
D^{nn}_{168}	$n_1 + n_2$	9.0	-2.6	16.0	≈0.0
D^{nn}_{169}	$n_1 + n_2$	8.4	-2.8	16.2	≈0.0
D^{nn}_{170}	$n_1 + n_2$	6.6	-3.6	17.0	≈0.0
D^{nn}_{171}	$n_1 + n_2$	5.2	-3.8	17.2	≈0.0
D^{nn}_{172}	$n_1 + n_2$	6.3	-4.4	17.8	≈0.0
D^{nn}_{173}	$n_1 + n_2$	6.8	-4.5	17.9	≈0.0
D^{nn}_{174}	$n_1 + n_2$	5.6	-4.6	18.0	≈0.0
D^{nn}_{175}	$n_1 + n_2$	5.9	-4.6	18.0	≈0.0
D^{nn}_{176}	$n_1 + n_2$	5.9	-4.8	18.2	≈0.0
D^{nn}_{177}	$n_1 + n_2$	6.7	-4.8	18.2	≈0.0
D^{nn}_{178}	$n_1 + n_2$	5.0	-5.0	18.5	≈0.0
D^{nn}_{179}	$n_1 + n_2$	4.9	-5.1	18.5	≈0.0
D^{nn}_{180}	$n_1 + n_2$	3.1	-5.2	18.6	≈0.0
D^{nn}_{181}	$n_1 + n_2$	2.3	-5.3	18.8	≈0.0
D^{nn}_{182}	$n_1 + n_2$	3.1	-5.4	18.9	≈0.0
D^{nn}_{183}	$n_1 + n_2$	4.4	-5.5	18.9	≈0.0
D^{nn}_{184}	$n_1 + n_2$	4.8	-5.5	18.9	≈0.0
D^{nn}_{185}	$n_1 + n_2$	5.1	-5.5	18.9	≈0.0
D^{nn}_{186}	$n_1 + n_2$	3.0	-5.5	19.0	≈0.0
D^{nn}_{187}	$n_1 + n_2$	3.1	-5.5	19.0	≈0.0
D^{nn}_{188}	$n_1 + n_2$	3.9	-5.6	19.0	≈0.0
D^{nn}_{189}	$n_1 + n_2$	6.5	-5.6	19.0	≈0.0
D^{nn}_{190}	$n_1 + n_2$	3.4	-5.6	19.0	≈0.0
D^{nn}_{191}	$n_1 + n_2$	6.3	-5.6	19.1	≈0.0
D^{nn}_{192}	$n_1 + n_2$	4.9	-5.6	19.1	≈0.0

**Dimer**	Composition	$\mathbf{BE}_{\mathrm{B3LYP-D3}}$	$\mathbf{BE}_{\mathrm{Gibbs}}$	$\Delta\Delta$**G**	$\%\chi_i(G))$
D_{193}^{nn}	$n_1 + n_2$	3.6	-5.7	19.1	≈0.0
D_{194}^{nn}	$n_1 + n_2$	4.8	-5.7	19.2	≈0.0
D_{195}^{nn}	$n_1 + n_2$	5.7	-5.7	19.2	≈0.0
D_{196}^{nn}	$n_1 + n_2$	5.4	-5.8	19.3	≈0.0
D_{197}^{nn}	$n_1 + n_2$	5.3	-5.8	19.3	≈0.0
D_{198}^{nn}	$n_1 + n_2$	4.1	-5.9	19.3	≈0.0
D_{199}^{nn}	$n_1 + n_2$	3.3	-5.9	19.3	≈0.0
D_{200}^{nn}	$n_1 + n_2$	3.6	-6.0	19.4	≈0.0
D_{201}^{nn}	$n_1 + n_2$	5.0	-6.0	19.4	≈0.0
D_{202}^{nn}	$n_1 + n_2$	3.1	-6.0	19.4	≈0.0
D_{203}^{nn}	$n_1 + n_2$	3.7	-6.1	19.5	≈0.0
D_{204}^{nn}	$n_1 + n_2$	5.4	-6.1	19.6	≈0.0
D_{205}^{nn}	$n_1 + n_2$	3.3	-6.2	19.6	≈0.0
D_{206}^{nn}	$n_1 + n_2$	3.3	-6.2	19.6	≈0.0
D_{207}^{nn}	$n_1 + n_2$	3.2	-6.2	19.7	≈0.0
D_{208}^{nn}	$n_1 + n_2$	3.4	-6.2	19.7	≈0.0
D_{209}^{nn}	$n_1 + n_2$	2.9	-6.3	19.7	≈0.0
D_{210}^{nn}	$n_1 + n_2$	3.2	-6.3	19.7	≈0.0
D_{211}^{nn}	$n_1 + n_2$	4.1	-6.3	19.7	≈0.0
D_{212}^{nn}	$n_1 + n_2$	3.3	-6.3	19.8	≈0.0
D_{213}^{nn}	$n_1 + n_2$	4.6	-6.3	19.8	≈0.0
D_{214}^{nn}	$n_1 + n_2$	5.4	-6.3	19.8	≈0.0
D_{215}^{nn}	$n_1 + n_2$	3.2	-6.4	19.9	≈0.0
D_{216}^{nn}	$n_1 + n_2$	2.8	-6.4	19.9	≈0.0
D_{217}^{nn}	$n_1 + n_2$	3.0	-6.4	19.9	≈0.0
D_{218}^{nn}	$n_1 + n_2$	2.8	-6.5	19.9	≈0.0
D_{219}^{nn}	$n_1 + n_2$	3.2	-6.5	19.9	≈0.0
D_{220}^{nn}	$n_1 + n_2$	3.0	-6.5	19.9	≈0.0
D_{221}^{nn}	$n_1 + n_2$	3.6	-6.5	19.9	≈0.0
D_{222}^{nn}	$n_1 + n_2$	3.6	-6.5	20.0	≈0.0
D_{223}^{nn}	$n_1 + n_2$	3.2	-6.5	20.0	≈0.0
D_{224}^{nn}	$n_1 + n_2$	3.2	-6.6	20.0	≈0.0
D_{225}^{nn}	$n_1 + n_2$	3.7	-6.7	20.1	≈0.0
D_{226}^{nn}	$n_1 + n_2$	3.4	-6.7	20.1	≈0.0
D_{227}^{nn}	$n_1 + n_2$	2.8	-6.7	20.1	≈0.0

**Dimer**	Composition	$\mathbf{BE}_{\text{B3LYP-D3}}$	$\mathbf{BE}_{\text{Gibbs}}$	$\Delta\Delta$**G**	$\%\chi_i(G))$
D_{228}^{nn}	$n_1 + n_2$	4.0	-6.7	20.1	≈0.0
D_{229}^{nn}	$n_1 + n_2$	4.5	-6.7	20.1	≈0.0
D_{230}^{nn}	$n_1 + n_2$	4.5	-6.8	20.2	≈0.0
D_{231}^{nn}	$n_1 + n_2$	3.0	-6.8	20.2	≈0.0
D_{232}^{nn}	$n_1 + n_2$	2.7	-6.8	20.3	≈0.0
D_{233}^{nn}	$n_1 + n_2$	4.5	-6.8	20.3	≈0.0
D_{234}^{nn}	$n_1 + n_2$	4.2	-6.8	20.3	≈0.0
D_{235}^{nn}	$n_1 + n_2$	3.9	-6.9	20.4	≈0.0
D_{236}^{nn}	$n_1 + n_2$	4.2	-6.9	20.4	≈0.0
D_{237}^{nn}	$n_1 + n_2$	3.5	-6.9	20.4	≈0.0
D_{238}^{nn}	$n_1 + n_2$	2.8	-7.0	20.4	≈0.0
D_{239}^{nn}	$n_1 + n_2$	3.5	-7.0	20.5	≈0.0
D_{240}^{nn}	$n_1 + n_2$	3.0	-7.1	20.6	≈0.0
D_{241}^{nn}	$n_1 + n_2$	1.5	-7.5	20.9	≈0.0
D_{242}^{nn}	$n_1 + n_2$	2.4	-7.6	21.0	≈0.0
D_{243}^{nn}	$n_1 + n_2$	2.7	-7.9	21.3	≈0.0
D_{244}^{nn}	$n_1 + n_2$	2.5	-7.9	21.4	≈0.0

Table S2: Binding energies and energy differences in kcal mol^{-1} for mixed dimers. ΔΔG: Gibbs energies relative to the global minimum. We list electronic ($BE_{B3LYP-D3}$) and Gibbs (BE_{Gibbs}) binding energies corrected by the B3LYP–D3 vibrational ZPE (values within parentheses are calculated for the bare B3LYP functional with no dispersion corrections). Isomer populations $\%\chi_i(G)$ derived from Boltzmann distributions of the Gibbs energies are also reported.

**Dimer**	Composition	$\mathbf{BE}_{B3LYP-D3}$	$\mathbf{BE}_{Gibbs}$	**ΔΔG**	$\%\chi_i(G))$
D_1^{nz}	$n_1 + z_1$	28.2	15.5 (-7.6)	≈0.0	44.9
D_2^{nz}	$n_2 + z_2$	29.7	17.9 (-0.5)	0.1	37.6
D_3^{nz}	$n_2 + z_2$	29.8	17.0 (-0.4)	1.1	7.4
D_4^{nz}	$n_2 + z_2$	29.8	17.0 (-2.5)	1.1	7.3
D_5^{nz}	$n_2 + z_2$	28.7	15.4	2.6	0.5
D_6^{nz}	$n_2 + z_2$	29.0	15.2	2.9	0.3
D_7^{nz}	$n_2 + z_2$	28.7	15.1	2.9	0.3
D_8^{nz}	$n_1 + z_1$	24.5	12.5	3.1	0.2
D_9^{nz}	$n_2 + z_2$	25.5	14.9	3.1	0.2
D_{10}^{nz}	$n_2 + z_2$	27.2	14.7	3.3	0.2
D_{11}^{nz}	$n_1 + z_1$	23.9	12.2	3.3	0.2
D_{12}^{nz}	$n_1 + z_1$	22.8	11.9	3.6	0.1
D_{13}^{nz}	$n_2 + z_2$	26.7	14.2	3.8	0.1
D_{14}^{nz}	$n_2 + z_2$	26.6	14.2	3.9	0.1
D_{15}^{nz}	$n_1 + z_1$	23.4	11.4	4.1	≈0.0
D_{16}^{nz}	$n_1 + z_1$	22.9	11.4	4.2	≈0.0
D_{17}^{nz}	$n_2 + z_2$	25.6	13.9	4.2	≈0.0
D_{18}^{nz}	$n_2 + z_2$	25.4	13.9	4.2	≈0.0
D_{19}^{nz}	$n_2 + z_2$	26.5	13.8	4.2	≈0.0
D_{20}^{nz}	$n_1 + z_1$	23.8	11.3	4.2	≈0.0
D_{21}^{nz}	$n_2 + z_2$	25.4	13.8	4.3	≈0.0
D_{22}^{nz}	$n_2 + z_2$	25.8	13.8	4.3	≈0.0
D_{23}^{nz}	$n_1 + z_1$	22.8	11.2	4.4	≈0.0
D_{24}^{nz}	$n_1 + z_1$	23.2	11.1	4.5	≈0.0
D_{25}^{nz}	$n_1 + z_1$	22.1	11.0	4.6	≈0.0
D_{26}^{nz}	$n_1 + z_1$	22.7	10.9	4.6	≈0.0
D_{27}^{nz}	$n_1 + z_1$	23.3	10.9	4.6	≈0.0
D_{28}^{nz}	$n_2 + z_2$	26.3	13.4	4.7	≈0.0
D_{29}^{nz}	$n_1 + z_1$	22.3	10.8	4.7	≈0.0
D_{30}^{nz}	$n_1 + z_1$	23.5	10.8	4.7	≈0.0
D_{31}^{nz}	$n_2 + z_2$	24.0	13.3	4.8	≈0.0
D_{32}^{nz}	$n_2 + z_2$	23.8	13.2	4.8	≈0.0
D_{33}^{nz}	$n_1 + z_1$	21.4	10.7	4.8	≈0.0
D_{34}^{nz}	$n_1 + z_1$	22.3	10.7	4.9	≈0.0
D_{35}^{nz}	$n_1 + z_1$	22.6	10.7	4.9	≈0.0

**Dimer**	Composition	$\mathbf{BE}_{\mathrm{B3LYP-D3}}$	$\mathbf{BE}_{Gibbs}$	ΔΔG	$\%\chi_i(G))$
D_{36}^{nz}	$n_2 + z_2$	23.4	13.2	4.9	≈0.0
D_{37}^{nz}	$n_2 + z_2$	24.4	13.1	5.0	≈0.0
D_{38}^{nz}	$n_2 + z_2$	25.6	13.0	5.0	≈0.0
D_{39}^{nz}	$n_1 + z_1$	21.9	10.5	5.0	≈0.0
D_{40}^{nz}	$n_1 + z_1$	21.6	10.4	5.1	≈0.0
D_{41}^{nz}	$n_1 + z_1$	22.1	10.4	5.1	≈0.0
D_{42}^{nz}	$n_1 + z_1$	22.2	10.3	5.2	≈0.0
D_{43}^{nz}	$n_1 + z_1$	21.3	10.3	5.2	≈0.0
D_{44}^{nz}	$n_1 + z_1$	21.9	10.3	5.2	≈0.0
D_{45}^{nz}	$n_1 + z_1$	21.6	10.1	5.4	≈0.0
D_{46}^{nz}	$n_2 + z_2$	25.3	12.5	5.5	≈0.0
D_{47}^{nz}	$n_1 + z_1$	22.5	9.9	5.6	≈0.0
D_{48}^{nz}	$n_2 + z_2$	26.1	12.3	5.7	≈0.0
D_{49}^{nz}	$n_2 + z_2$	24.3	12.3	5.7	≈0.0
D_{50}^{nz}	$n_1 + z_1$	21.6	9.8	5.7	≈0.0
D_{51}^{nz}	$n_1 + z_1$	21.0	9.8	5.7	≈0.0
D_{52}^{nz}	$n_1 + z_1$	21.1	9.8	5.7	≈0.0
D_{53}^{nz}	$n_1 + z_1$	22.5	9.6	5.9	≈0.0
D_{54}^{nz}	$n_1 + z_1$	21.3	9.6	5.9	≈0.0
D_{55}^{nz}	$n_1 + z_1$	21.9	9.6	5.9	≈0.0
D_{56}^{nz}	$n_2 + z_2$	22.7	12.0	6.0	≈0.0
D_{57}^{nz}	$n_1 + z_1$	20.4	9.5	6.0	≈0.0
D_{58}^{nz}	$n_2 + z_2$	25.0	12.0	6.1	≈0.0
D_{59}^{nz}	$n_1 + z_1$	21.5	9.5	6.1	≈0.0
D_{60}^{nz}	$n_2 + z_2$	25.0	12.0	6.1	≈0.0
D_{61}^{nz}	$n_1 + z_1$	21.4	9.4	6.1	≈0.0
D_{62}^{nz}	$n_1 + z_1$	21.5	9.4	6.2	≈0.0
D_{63}^{nz}	$n_2 + z_2$	24.0	11.8	6.2	≈0.0
D_{64}^{nz}	$n_1 + z_1$	21.4	9.3	6.2	≈0.0
D_{65}^{nz}	$n_1 + z_1$	21.1	9.3	6.3	≈0.0
D_{66}^{nz}	$n_2 + z_2$	23.6	11.7	6.3	≈0.0
D_{67}^{nz}	$n_1 + z_1$	19.0	9.1	6.5	≈0.0
D_{68}^{nz}	$n_1 + z_1$	20.4	9.1	6.5	≈0.0
D_{69}^{nz}	$n_2 + z_2$	24.3	11.5	6.5	≈0.0
D_{70}^{nz}	$n_1 + z_1$	19.1	9.0	6.6	≈0.0

**Dimer**	Composition	$\mathbf{BE}_{\mathrm{B3LYP-D3}}$	$\mathbf{BE}_{Gibbs}$	$\Delta\Delta$**G**	$\%\chi_i(G))$
D_{71}^{nz}	$n_1 + z_1$	20.3	8.8	6.7	≈0.0
D_{72}^{nz}	$n_1 + z_1$	21.3	8.8	6.8	≈0.0
D_{73}^{nz}	$n_2 + z_2$	22.2	11.3	6.8	≈0.0
D_{74}^{nz}	$n_2 + z_2$	25.2	11.2	6.8	≈0.0
D_{75}^{nz}	$n_1 + z_1$	20.1	8.7	6.9	≈0.0
D_{76}^{nz}	$n_1 + z_1$	20.1	8.7	6.9	≈0.0
D_{77}^{nz}	$n_1 + z_1$	21.0	8.6	6.9	≈0.0
D_{78}^{nz}	$n_1 + z_1$	19.2	8.6	6.9	≈0.0
D_{79}^{nz}	$n_1 + z_1$	17.9	8.5	7.0	≈0.0
D_{80}^{nz}	$n_1 + z_1$	20.1	8.5	7.0	≈0.0
D_{81}^{nz}	$n_1 + z_1$	19.3	8.5	7.1	≈0.0
D_{82}^{nz}	$n_1 + z_1$	19.1	8.4	7.1	≈0.0
D_{83}^{nz}	$n_1 + z_1$	18.5	8.3	7.2	≈0.0
D_{84}^{nz}	$n_1 + z_1$	18.6	8.3	7.2	≈0.0
D_{85}^{nz}	$n_1 + z_1$	18.4	8.2	7.3	≈0.0
D_{86}^{nz}	$n_1 + z_1$	19.4	8.2	7.3	≈0.0
D_{87}^{nz}	$n_1 + z_1$	19.0	8.1	7.5	≈0.0
D_{88}^{nz}	$n_1 + z_1$	19.7	8.0	7.6	≈0.0
D_{89}^{nz}	$n_2 + z_2$	22.3	10.4	7.6	≈0.0
D_{90}^{nz}	$n_1 + z_1$	19.3	7.9	7.7	≈0.0
D_{91}^{nz}	$n_2 + z_2$	22.1	10.3	7.8	≈0.0
D_{92}^{nz}	$n_1 + z_1$	19.4	7.7	7.8	≈0.0
D_{93}^{nz}	$n_1 + z_1$	19.5	7.7	7.8	≈0.0
D_{94}^{nz}	$n_1 + z_1$	20.4	7.7	7.9	≈0.0
D_{95}^{nz}	$n_1 + z_1$	18.4	7.6	7.9	≈0.0
D_{96}^{nz}	$n_1 + z_1$	19.4	7.6	8.0	≈0.0
D_{97}^{nz}	$n_2 + z_2$	22.3	10.1	8.0	≈0.0
D_{98}^{nz}	$n_1 + z_1$	18.8	7.5	8.0	≈0.0
D_{99}^{nz}	$n_1 + z_1$	17.6	7.2	8.3	≈0.0
D_{100}^{nz}	$n_2 + z_2$	22.4	9.7	8.4	≈0.0
D_{101}^{nz}	$n_1 + z_1$	18.3	7.2	8.4	≈0.0
D_{102}^{nz}	$n_1 + z_1$	18.1	7.0	8.5	≈0.0
D_{103}^{nz}	$n_1 + z_1$	17.6	7.0	8.5	≈0.0
D_{104}^{nz}	$n_1 + z_1$	18.6	6.9	8.6	≈0.0
D_{105}^{nz}	$n_1 + z_1$	17.6	6.8	8.7	≈0.0

**Dimer**	Composition	$\mathbf{BE}_{\text{B3LYP-D3}}$	$\mathbf{BE}_{Gibbs}$	**ΔΔG**	$\%\chi_i(G))$
D^{nz}_{106}	$n_1 + z_1$	18.7	6.8	8.8	≈0.0
D^{nz}_{107}	$n_2 + z_2$	21.2	9.2	8.8	≈0.0
D^{nz}_{108}	$n_2 + z_2$	22.1	9.2	8.8	≈0.0
D^{nz}_{109}	$n_2 + z_2$	20.2	9.2	8.9	≈0.0
D^{nz}_{110}	$n_1 + z_1$	17.2	6.4	9.1	≈0.0
D^{nz}_{111}	$n_2 + z_2$	19.9	8.9	9.2	≈0.0
D^{nz}_{112}	$n_2 + z_2$	20.0	8.8	9.2	≈0.0
D^{nz}_{113}	$n_2 + z_2$	20.2	8.8	9.2	≈0.0
D^{nz}_{114}	$n_2 + z_2$	20.3	8.8	9.2	≈0.0
D^{nz}_{115}	$n_2 + z_2$	20.7	8.8	9.3	≈0.0
D^{nz}_{116}	$n_2 + z_2$	20.6	8.8	9.3	≈0.0
D^{nz}_{117}	$n_1 + z_1$	16.9	6.3	9.3	≈0.0
D^{nz}_{118}	$n_2 + z_2$	19.8	8.6	9.5	≈0.0
D^{nz}_{119}	$n_2 + z_2$	21.0	8.4	9.6	≈0.0
D^{nz}_{120}	$n_2 + z_2$	20.4	8.3	9.7	≈0.0
D^{nz}_{121}	$n_2 + z_2$	20.0	8.1	9.9	≈0.0
D^{nz}_{122}	$n_2 + z_2$	19.6	8.0	10.0	≈0.0
D^{nz}_{123}	$n_2 + z_2$	20.1	8.0	10.1	≈0.0
D^{nz}_{124}	$n_2 + z_2$	19.2	7.9	10.1	≈0.0
D^{nz}_{125}	$n_2 + z_2$	20.5	7.9	10.1	≈0.0
D^{nz}_{126}	$n_2 + z_2$	18.7	7.9	10.2	≈0.0
D^{nz}_{127}	$n_2 + z_2$	18.9	7.9	10.2	≈0.0
D^{nz}_{128}	$n_1 + z_1$	16.9	5.3	10.2	≈0.0
D^{nz}_{129}	$n_1 + z_1$	15.5	5.3	10.2	≈0.0
D^{nz}_{130}	$n_2 + z_2$	18.4	7.7	10.3	≈0.0
D^{nz}_{131}	$n_2 + z_2$	18.3	7.6	10.4	≈0.0
D^{nz}_{132}	$n_2 + z_2$	18.8	7.6	10.4	≈0.0
D^{nz}_{133}	$n_2 + z_2$	17.4	7.6	10.4	≈0.0
D^{nz}_{134}	$n_2 + z_2$	18.9	7.5	10.5	≈0.0
D^{nz}_{135}	$n_2 + z_2$	18.7	7.4	10.7	≈0.0
D^{nz}_{136}	$n_2 + z_2$	18.4	7.3	10.8	≈0.0
D^{nz}_{137}	$n_2 + z_2$	18.3	7.2	10.9	≈0.0
D^{nz}_{138}	$n_2 + z_2$	18.8	7.1	10.9	≈0.0
D^{nz}_{139}	$n_2 + z_2$	18.7	7.1	11.0	≈0.0
D^{nz}_{140}	$n_2 + z_2$	18.3	7.0	11.0	≈0.0

**Dimer**	Composition	$\mathbf{BE}_{\text{B3LYP-D3}}$	$\mathbf{BE}_{Gibbs}$	$\Delta\Delta$**G**	$\%\chi_i(G))$
D^{nz}_{141}	$n_2 + z_2$	16.9	6.9	11.1	≈0.0
D^{nz}_{142}	$n_2 + z_2$	18.4	6.8	11.2	≈0.0
D^{nz}_{143}	$n_2 + z_2$	17.9	6.6	11.5	≈0.0
D^{nz}_{144}	$n_2 + z_2$	16.8	6.5	11.6	≈0.0
D^{nz}_{145}	$n_2 + z_2$	18.0	6.4	11.6	≈0.0
D^{nz}_{146}	$n_2 + z_2$	17.7	6.4	11.7	≈0.0
D^{nz}_{147}	$n_2 + z_2$	16.6	5.9	12.1	≈0.0
D^{nz}_{148}	$n_2 + z_2$	16.3	5.2	12.9	≈0.0
D^{nz}_{149}	$n_2 + z_1$	14.3	3.3	14.4	≈0.0
D^{nz}_{150}	$n_2 + z_1$	11.9	2.0	15.7	≈0.0
D^{nz}_{151}	$n_2 + z_1$	13.6	2.0	15.8	≈0.0
D^{nz}_{152}	$n_2 + z_1$	13.8	1.3	16.4	≈0.0
D^{nz}_{153}	$n_2 + z_1$	11.1	1.3	16.4	≈0.0
D^{nz}_{154}	$n_2 + z_1$	13.8	1.3	16.5	≈0.0
D^{nz}_{155}	$n_2 + z_1$	11.2	1.0	16.8	≈0.0
D^{nz}_{156}	$n_2 + z_1$	12.2	0.9	16.9	≈0.0
D^{nz}_{157}	$n_2 + z_1$	12.3	0.8	16.9	≈0.0
D^{nz}_{158}	$n_2 + z_1$	11.1	0.8	16.9	≈0.0
D^{nz}_{159}	$n_2 + z_1$	11.9	0.7	17.0	≈0.0
D^{nz}_{160}	$n_2 + z_1$	11.9	0.7	17.1	≈0.0
D^{nz}_{161}	$n_2 + z_1$	10.9	0.6	17.1	≈0.0
D^{nz}_{162}	$n_2 + z_1$	10.9	0.6	17.1	≈0.0
D^{nz}_{163}	$n_1 + z_2$	9.5	-1.5	17.4	≈0.0
D^{nz}_{164}	$n_1 + z_2$	7.6	-1.6	17.4	≈0.0
D^{nz}_{165}	$n_2 + z_1$	11.5	0.3	17.4	≈0.0
D^{nz}_{166}	$n_1 + z_2$	10.0	-1.7	17.5	≈0.0
D^{nz}_{167}	$n_1 + z_2$	9.5	-2.0	17.9	≈0.0
D^{nz}_{168}	$n_1 + z_2$	7.4	-2.0	17.9	≈0.0
D^{nz}_{169}	$n_2 + z_1$	11.2	-0.1	17.9	≈0.0
D^{nz}_{170}	$n_2 + z_1$	10.0	-0.2	17.9	≈0.0
D^{nz}_{171}	$n_1 + z_2$	8.0	-2.1	17.9	≈0.0
D^{nz}_{172}	$n_1 + z_2$	7.5	-2.4	18.3	≈0.0
D^{nz}_{173}	$n_1 + z_2$	8.3	-2.5	18.3	≈0.0
D^{nz}_{174}	$n_2 + z_1$	11.5	-0.6	18.4	≈0.0
D^{nz}_{175}	$n_1 + z_2$	7.3	-2.6	18.4	≈0.0

**Dimer**	Composition	$\mathbf{BE}_{\mathrm{B3LYP-D3}}$	$\mathbf{BE}_{Gibbs}$	$\Delta\Delta$**G**	$\%\chi_i(G))$
D^{nz}_{176}	$n_1 + z_2$	8.2	-2.7	18.5	≈0.0
D^{nz}_{177}	$n_1 + z_2$	8.5	-2.8	18.6	≈0.0
D^{nz}_{178}	$n_1 + z_2$	8.3	-2.8	18.6	≈0.0
D^{nz}_{179}	$n_1 + z_2$	7.9	-2.8	18.6	≈0.0
D^{nz}_{180}	$n_2 + z_1$	9.9	-1.0	18.7	≈0.0
D^{nz}_{181}	$n_2 + z_1$	10.3	-1.1	18.8	≈0.0
D^{nz}_{182}	$n_1 + z_1$	8.0	-3.3	18.9	≈0.0
D^{nz}_{183}	$n_1 + z_2$	8.0	-3.1	19.0	≈0.0
D^{nz}_{184}	$n_2 + z_1$	10.3	-1.3	19.0	≈0.0
D^{nz}_{185}	$n_2 + z_1$	9.8	-1.3	19.1	≈0.0
D^{nz}_{186}	$n_1 + z_2$	8.2	-3.5	19.3	≈0.0
D^{nz}_{187}	$n_1 + z_2$	7.9	-3.9	19.8	≈0.0
D^{nz}_{188}	$n_2 + z_1$	9.0	-2.1	19.8	≈0.0
D^{nz}_{189}	$n_1 + z_2$	5.7	-4.1	20.0	≈0.0
D^{nz}_{190}	$n_2 + z_1$	9.1	-2.3	20.1	≈0.0
D^{nz}_{191}	$n_1 + z_2$	7.3	-4.3	20.1	≈0.0
D^{nz}_{192}	$n_1 + z_2$	7.1	-4.3	20.2	≈0.0
D^{nz}_{193}	$n_1 + z_2$	7.9	-4.6	20.4	≈0.0
D^{nz}_{194}	$n_1 + z_2$	7.0	-4.6	20.4	≈0.0
D^{nz}_{195}	$n_2 + z_1$	8.6	-2.7	20.5	≈0.0
D^{nz}_{196}	$n_1 + z_2$	5.7	-4.7	20.5	≈0.0
D^{nz}_{197}	$n_1 + z_2$	5.7	-4.7	20.5	≈0.0
D^{nz}_{198}	$n_1 + z_1$	5.9	-5.0	20.5	≈0.0
D^{nz}_{199}	$n_2 + z_1$	9.7	-2.9	20.7	≈0.0
D^{nz}_{200}	$n_1 + z_2$	5.1	-4.9	20.7	≈0.0
D^{nz}_{201}	$n_1 + z_2$	5.4	-5.0	20.8	≈0.0
D^{nz}_{202}	$n_1 + z_1$	5.9	-5.3	20.8	≈0.0
D^{nz}_{203}	$n_1 + z_2$	5.8	-5.0	20.9	≈0.0
D^{nz}_{204}	$n_1 + z_2$	6.1	-5.0	20.9	≈0.0
D^{nz}_{205}	$n_1 + z_2$	5.4	-5.1	20.9	≈0.0
D^{nz}_{206}	$n_1 + z_2$	6.3	-5.1	20.9	≈0.0
D^{nz}_{207}	$n_1 + z_2$	6.5	-5.2	21.0	≈0.0
D^{nz}_{208}	$n_1 + z_2$	6.6	-5.2	21.0	≈0.0
D^{nz}_{209}	$n_1 + z_2$	4.7	-5.2	21.0	≈0.0
D^{nz}_{210}	$n_1 + z_2$	5.4	-5.2	21.0	≈0.0

**Dimer**	Composition	$\mathbf{BE}_{\mathrm{B3LYP-D3}}$	$\mathbf{BE}_{Gibbs}$	ΔΔ**G**	$\%\chi_i(G))$
D_{211}^{nz}	$n_2 + z_1$	7.4	-3.3	21.0	≈0.0
D_{212}^{nz}	$n_1 + z_2$	6.4	-5.2	21.1	≈0.0
D_{213}^{nz}	$n_1 + z_2$	6.4	-5.3	21.1	≈0.0
D_{214}^{nz}	$n_1 + z_2$	6.0	-5.4	21.2	≈0.0
D_{215}^{nz}	$n_1 + z_2$	5.8	-5.4	21.2	≈0.0
D_{216}^{nz}	$n_1 + z_2$	5.7	-5.4	21.3	≈0.0
D_{217}^{nz}	$n_1 + z_2$	6.4	-5.4	21.3	≈0.0
D_{218}^{nz}	$n_2 + z_1$	7.2	-3.6	21.3	≈0.0
D_{219}^{nz}	$n_1 + z_2$	5.7	-5.5	21.3	≈0.0
D_{220}^{nz}	$n_2 + z_1$	7.4	-3.6	21.3	≈0.0
D_{221}^{nz}	$n_1 + z_2$	4.8	-5.5	21.4	≈0.0
D_{222}^{nz}	$n_1 + z_2$	6.0	-5.5	21.4	≈0.0
D_{223}^{nz}	$n_1 + z_2$	5.8	-5.6	21.4	≈0.0
D_{224}^{nz}	$n_1 + z_2$	5.2	-5.6	21.4	≈0.0
D_{225}^{nz}	$n_1 + z_2$	5.5	-5.6	21.5	≈0.0
D_{226}^{nz}	$n_1 + z_2$	4.6	-5.6	21.5	≈0.0
D_{227}^{nz}	$n_1 + z_2$	6.1	-5.6	21.5	≈0.0
D_{228}^{nz}	$n_1 + z_2$	5.8	-5.7	21.6	≈0.0
D_{229}^{nz}	$n_2 + z_1$	6.7	-3.8	21.6	≈0.0
D_{230}^{nz}	$n_1 + z_2$	5.4	-5.8	21.6	≈0.0
D_{231}^{nz}	$n_1 + z_2$	5.7	-5.8	21.6	≈0.0
D_{232}^{nz}	$n_1 + z_2$	5.5	-5.9	21.7	≈0.0
D_{233}^{nz}	$n_1 + z_2$	4.5	-5.9	21.7	≈0.0
D_{234}^{nz}	$n_1 + z_2$	5.6	-5.9	21.7	≈0.0
D_{235}^{nz}	$n_1 + z_2$	4.8	-5.9	21.8	≈0.0
D_{236}^{nz}	$n_2 + z_1$	7.2	-4.0	21.8	≈0.0
D_{237}^{nz}	$n_1 + z_2$	4.4	-6.0	21.8	≈0.0
D_{238}^{nz}	$n_1 + z_2$	4.9	-6.0	21.9	≈0.0
D_{239}^{nz}	$n_1 + z_2$	3.4	-6.0	21.9	≈0.0
D_{240}^{nz}	$n_1 + z_2$	3.2	-6.1	21.9	≈0.0
D_{241}^{nz}	$n_1 + z_2$	4.7	-6.1	21.9	≈0.0
D_{242}^{nz}	$n_1 + z_2$	5.0	-6.1	21.9	≈0.0
D_{243}^{nz}	$n_1 + z_2$	5.5	-6.1	22.0	≈0.0
D_{244}^{nz}	$n_1 + z_2$	5.3	-6.2	22.0	≈0.0
D_{245}^{nz}	$n_1 + z_2$	5.3	-6.2	22.0	≈0.0

**Dimer**	Composition	$\mathbf{BE}_{\mathrm{B3LYP-D3}}$	$\mathbf{BE}_{Gibbs}$	ΔΔ**G**	$\%\chi_i(G))$
D_{246}^{nz}	$n_2 + z_1$	7.0	-4.3	22.0	≈0.0
D_{247}^{nz}	$n_1 + z_2$	4.9	-6.3	22.1	≈0.0
D_{248}^{nz}	$n_1 + z_2$	2.9	-6.3	22.1	≈0.0
D_{249}^{nz}	$n_1 + z_2$	3.7	-6.3	22.1	≈0.0
D_{250}^{nz}	$n_1 + z_2$	4.8	-6.3	22.1	≈0.0
D_{251}^{nz}	$n_2 + z_1$	6.6	-4.4	22.2	≈0.0
D_{252}^{nz}	$n_1 + z_2$	4.2	-6.4	22.2	≈0.0
D_{253}^{nz}	$n_1 + z_2$	5.2	-6.4	22.2	≈0.0
D_{254}^{nz}	$n_1 + z_2$	4.6	-6.5	22.3	≈0.0
D_{255}^{nz}	$n_1 + z_2$	2.8	-6.5	22.4	≈0.0
D_{256}^{nz}	$n_1 + z_2$	5.0	-6.6	22.4	≈0.0
D_{257}^{nz}	$n_1 + z_2$	3.4	-6.6	22.4	≈0.0
D_{258}^{nz}	$n_2 + z_1$	6.1	-4.7	22.5	≈0.0
D_{259}^{nz}	$n_1 + z_2$	4.1	-6.7	22.6	≈0.0
D_{260}^{nz}	$n_1 + z_2$	2.5	-6.8	22.6	≈0.0
D_{261}^{nz}	$n_1 + z_2$	2.4	-6.8	22.7	≈0.0
D_{262}^{nz}	$n_1 + z_2$	2.9	-6.8	22.7	≈0.0
D_{263}^{nz}	$n_1 + z_2$	5.2	-6.9	22.7	≈0.0
D_{264}^{nz}	$n_1 + z_2$	2.7	-6.9	22.7	≈0.0
D_{265}^{nz}	$n_2 + z_1$	5.2	-5.0	22.7	≈0.0
D_{266}^{nz}	$n_1 + z_2$	4.5	-7.0	22.8	≈0.0
D_{267}^{nz}	$n_1 + z_2$	3.9	-7.0	22.8	≈0.0
D_{268}^{nz}	$n_1 + z_2$	4.2	-7.1	22.9	≈0.0
D_{269}^{nz}	$n_1 + z_2$	3.7	-7.1	22.9	≈0.0
D_{270}^{nz}	$n_2 + z_1$	5.7	-5.2	23.0	≈0.0
D_{271}^{nz}	$n_2 + z_1$	6.1	-5.2	23.0	≈0.0
D_{272}^{nz}	$n_1 + z_2$	3.1	-7.2	23.0	≈0.0
D_{273}^{nz}	$n_2 + z_1$	5.5	-5.3	23.1	≈0.0
D_{274}^{nz}	$n_2 + z_1$	5.0	-5.3	23.1	≈0.0
D_{275}^{nz}	$n_2 + z_1$	5.5	-5.4	23.1	≈0.0
D_{276}^{nz}	$n_1 + z_2$	3.3	-7.3	23.2	≈0.0
D_{277}^{nz}	$n_2 + z_1$	5.2	-5.4	23.2	≈0.0
D_{278}^{nz}	$n_2 + z_1$	5.5	-5.5	23.2	≈0.0
D_{279}^{nz}	$n_2 + z_1$	5.9	-5.5	23.2	≈0.0
D_{280}^{nz}	$n_1 + z_2$	4.4	-7.4	23.2	≈0.0

**Dimer**	Composition	$\mathbf{BE}_{\mathrm{B3LYP-D3}}$	$\mathbf{BE}_{Gibbs}$	$\Delta\Delta$**G**	$\%\chi_i(G))$
D^{nz}_{281}	$n_1 + z_2$	3.1	-7.4	23.3	≈0.0
D^{nz}_{282}	$n_2 + z_1$	5.5	-5.5	23.3	≈0.0
D^{nz}_{283}	$n_2 + z_1$	4.6	-5.6	23.3	≈0.0
D^{nz}_{284}	$n_1 + z_2$	3.6	-7.5	23.3	≈0.0
D^{nz}_{285}	$n_1 + z_2$	2.3	-7.5	23.3	≈0.0
D^{nz}_{286}	$n_2 + z_1$	5.5	-5.6	23.4	≈0.0
D^{nz}_{287}	$n_2 + z_1$	5.9	-5.6	23.4	≈0.0
D^{nz}_{288}	$n_2 + z_1$	4.7	-5.6	23.4	≈0.0
D^{nz}_{289}	$n_2 + z_1$	5.1	-5.7	23.4	≈0.0
D^{nz}_{290}	$n_2 + z_1$	5.8	-5.7	23.4	≈0.0
D^{nz}_{291}	$n_2 + z_1$	3.5	-5.7	23.5	≈0.0
D^{nz}_{292}	$n_2 + z_1$	4.3	-5.7	23.5	≈0.0
D^{nz}_{293}	$n_2 + z_1$	4.4	-5.8	23.6	≈0.0
D^{nz}_{294}	$n_2 + z_1$	3.9	-5.9	23.7	≈0.0
D^{nz}_{295}	$n_2 + z_1$	4.0	-6.0	23.8	≈0.0
D^{nz}_{296}	$n_2 + z_1$	4.6	-6.0	23.8	≈0.0
D^{nz}_{297}	$n_2 + z_1$	4.8	-6.1	23.9	≈0.0
D^{nz}_{298}	$n_2 + z_1$	4.2	-6.1	23.9	≈0.0
D^{nz}_{299}	$n_1 + z_2$	1.3	-8.0	23.9	≈0.0
D^{nz}_{300}	$n_2 + z_1$	3.0	-6.2	23.9	≈0.0
D^{nz}_{301}	$n_2 + z_1$	5.5	-6.2	24.0	≈0.0
D^{nz}_{302}	$n_1 + z_2$	2.2	-8.1	24.0	≈0.0
D^{nz}_{303}	$n_2 + z_1$	3.9	-6.2	24.0	≈0.0
D^{nz}_{304}	$n_2 + z_1$	4.2	-6.3	24.0	≈0.0
D^{nz}_{305}	$n_2 + z_1$	4.9	-6.3	24.1	≈0.0
D^{nz}_{306}	$n_2 + z_1$	2.9	-6.3	24.1	≈0.0
D^{nz}_{307}	$n_2 + z_1$	4.9	-6.5	24.2	≈0.0
D^{nz}_{308}	$n_1 + z_2$	2.4	-8.4	24.3	≈0.0
D^{nz}_{309}	$n_2 + z_1$	3.5	-6.5	24.3	≈0.0
D^{nz}_{310}	$n_1 + z_2$	2.0	-8.5	24.4	≈0.0
D^{nz}_{311}	$n_2 + z_1$	3.5	-6.7	24.5	≈0.0
D^{nz}_{312}	$n_2 + z_1$	3.1	-6.8	24.6	≈0.0
D^{nz}_{313}	$n_2 + z_1$	4.5	-6.8	24.6	≈0.0
D^{nz}_{314}	$n_2 + z_1$	4.8	-7.0	24.8	≈0.0
D^{nz}_{315}	$n_2 + z_1$	4.1	-7.1	24.8	≈0.0

**Dimer**	Composition	$\mathbf{BE}_{\mathrm{B3LYP-D3}}$	$\mathbf{BE}_{Gibbs}$	$\Delta\Delta$**G**	$\%\chi_i(G))$
D_{316}^{nz}	$n_1 + z_2$	1.5	-9.7	25.5	≈0.0

Table S3: Binding energies and energy differences in kcal mol^{-1} for zwitterionic dimers. $\Delta\Delta$G: Gibbs energies relative to the global minimum. We list electronic (BE$_{\mathrm{B3LYP-D3}}$) and Gibbs (BE$_{\mathrm{Gibbs}}$) binding energies corrected by the B3LYP–D3 vibrational ZPE (values within parentheses are calculated for the bare B3LYP functional with no dispersion corrections). Isomer populations $\%\chi_i(G)$ derived from Boltzmann distributions of the Gibbs energies are also reported.

**Dimer**	Composition	**BE**$_{\mathrm{B3LYP-D3}}$	**BE**$_{Gibbs}$	$\Delta\Delta$**G**	$\%\chi_i(G))$
D_1^{zz}	z_2+z_2	35.2	21.8 (-0.2)	≈0.0	51.8
D_2^{zz}	z_1+z_1	31.1	20.5 (-0.9)	0.7	15.1
D_3^{zz}	z_2+z_2	33.6	20.6 (-1.2)	1.1	7.6
D_4^{zz}	z_1+z_2	32.5	20.2 (-0.5)	1.3	5.6
D_5^{zz}	z_1+z_2	32.6	20.0	1.5	4.0
D_6^{zz}	z_1+z_2	33.1	19.9	1.6	3.5
D_7^{zz}	z_2+z_2	32.7	20.0	1.8	2.4
D_8^{zz}	z_1+z_2	31.4	19.5	2.0	1.9
D_9^{zz}	z_1+z_2	32.0	19.5	2.0	1.8
D_{10}^{zz}	z_2+z_2	32.8	19.7	2.1	1.5
D_{11}^{zz}	z_2+z_2	32.1	19.6	2.2	1.4
D_{12}^{zz}	z_2+z_2	31.6	19.5	2.2	1.2
D_{13}^{zz}	z_1+z_2	31.5	19.2	2.3	1.0
D_{14}^{zz}	z_1+z_2	31.9	18.7	2.8	0.4
D_{15}^{zz}	z_1+z_2	29.5	17.9	3.6	0.1
D_{16}^{zz}	z_1+z_2	29.5	17.7	3.8	0.1
D_{17}^{zz}	z_2+z_2	30.1	17.9	3.8	0.1
D_{18}^{zz}	z_2+z_2	30.0	17.7	4.1	0.1
D_{19}^{zz}	z_1+z_1	29.2	17.1	4.1	≈0.0
D_{20}^{zz}	z_2+z_2	30.1	17.5	4.2	≈0.0
D_{21}^{zz}	z_2+z_2	30.1	17.5	4.2	≈0.0
D_{22}^{zz}	z_1+z_1	29.3	16.7	4.5	≈0.0
D_{23}^{zz}	z_1+z_2	29.2	17.0	4.5	≈0.0
D_{24}^{zz}	z_1+z_2	28.6	16.8	4.6	≈0.0
D_{25}^{zz}	z_1+z_1	27.8	16.5	4.7	≈0.0
D_{26}^{zz}	z_1+z_2	28.1	16.7	4.8	≈0.0
D_{27}^{zz}	z_1+z_2	28.9	16.6	4.8	≈0.0
D_{28}^{zz}	z_2+z_2	30.1	16.6	5.1	≈0.0
D_{29}^{zz}	z_1+z_1	28.1	16.0	5.2	≈0.0
D_{30}^{zz}	z_1+z_2	29.5	16.2	5.3	≈0.0
D_{31}^{zz}	z_1+z_2	28.9	16.2	5.3	≈0.0
D_{32}^{zz}	z_2+z_2	29.2	16.5	5.3	≈0.0
D_{33}^{zz}	z_1+z_2	28.0	16.2	5.3	≈0.0
D_{34}^{zz}	z_1+z_2	27.2	16.2	5.3	≈0.0
D_{35}^{zz}	z_1+z_1	28.1	15.8	5.4	≈0.0

**Dimer**	Composition	$\mathbf{BE}_{\mathrm{B3LYP-D3}}$	$\mathbf{BE}_{Gibbs}$	ΔΔG	$\%\chi_i(G))$
D_{36}^{zz}	$z_1 + z_2$	27.7	16.1	5.4	≈0.0
D_{37}^{zz}	$z_1 + z_1$	27.1	15.7	5.5	≈0.0
D_{38}^{zz}	$z_1 + z_1$	27.7	15.7	5.5	≈0.0
D_{39}^{zz}	$z_1 + z_1$	26.8	15.7	5.5	≈0.0
D_{40}^{zz}	$z_1 + z_1$	27.9	15.6	5.6	≈0.0
D_{41}^{zz}	$z_1 + z_2$	28.1	15.8	5.7	≈0.0
D_{42}^{zz}	$z_1 + z_2$	27.8	15.7	5.8	≈0.0
D_{43}^{zz}	$z_1 + z_2$	28.7	15.7	5.8	≈0.0
D_{44}^{zz}	$z_1 + z_1$	27.6	15.3	5.9	≈0.0
D_{45}^{zz}	$z_1 + z_1$	26.6	15.3	5.9	≈0.0
D_{46}^{zz}	$z_1 + z_2$	27.5	15.5	6.0	≈0.0
D_{47}^{zz}	$z_1 + z_2$	26.1	15.4	6.1	≈0.0
D_{48}^{zz}	$z_1 + z_1$	27.6	15.1	6.1	≈0.0
D_{49}^{zz}	$z_1 + z_1$	27.3	15.0	6.2	≈0.0
D_{50}^{zz}	$z_1 + z_2$	27.6	15.3	6.2	≈0.0
D_{51}^{zz}	$z_1 + z_2$	26.1	15.2	6.3	≈0.0
D_{52}^{zz}	$z_1 + z_1$	25.9	14.8	6.4	≈0.0
D_{53}^{zz}	$z_1 + z_1$	26.3	14.8	6.4	≈0.0
D_{54}^{zz}	$z_1 + z_2$	26.1	15.0	6.5	≈0.0
D_{55}^{zz}	$z_1 + z_1$	27.6	14.7	6.5	≈0.0
D_{56}^{zz}	$z_1 + z_1$	25.8	14.6	6.6	≈0.0
D_{57}^{zz}	$z_1 + z_2$	26.4	14.9	6.6	≈0.0
D_{58}^{zz}	$z_1 + z_2$	26.5	14.8	6.7	≈0.0
D_{59}^{zz}	$z_1 + z_1$	25.8	14.5	6.7	≈0.0
D_{60}^{zz}	$z_1 + z_2$	26.4	14.8	6.7	≈0.0
D_{61}^{zz}	$z_1 + z_2$	26.0	14.8	6.7	≈0.0
D_{62}^{zz}	$z_2 + z_2$	26.7	15.0	6.7	≈0.0
D_{63}^{zz}	$z_1 + z_1$	26.5	14.4	6.8	≈0.0
D_{64}^{zz}	$z_1 + z_2$	25.5	14.6	6.9	≈0.0
D_{65}^{zz}	$z_1 + z_2$	26.3	14.6	6.9	≈0.0
D_{66}^{zz}	$z_1 + z_2$	25.7	14.6	6.9	≈0.0
D_{67}^{zz}	$z_1 + z_2$	26.7	14.6	6.9	≈0.0
D_{68}^{zz}	$z_1 + z_1$	24.8	14.3	6.9	≈0.0
D_{69}^{zz}	$z_2 + z_2$	26.6	14.8	6.9	≈0.0
D_{70}^{zz}	$z_2 + z_2$	26.6	14.8	7.0	≈0.0

**Dimer**	Composition	$\mathbf{BE}_{\text{B3LYP-D3}}$	$\mathbf{BE}_{Gibbs}$	ΔΔ**G**	$\%\chi_i(G))$
D^{zz}_{71}	$z_1 + z_2$	26.0	14.5	7.0	≈0.0
D^{zz}_{72}	$z_1 + z_2$	26.4	14.5	7.0	≈0.0
D^{zz}_{73}	$z_1 + z_2$	25.6	14.4	7.1	≈0.0
D^{zz}_{74}	$z_1 + z_1$	27.1	14.1	7.1	≈0.0
D^{zz}_{75}	$z_1 + z_2$	25.6	14.4	7.1	≈0.0
D^{zz}_{76}	$z_2 + z_2$	27.1	14.6	7.2	≈0.0
D^{zz}_{77}	$z_1 + z_2$	25.8	14.2	7.3	≈0.0
D^{zz}_{78}	$z_1 + z_2$	25.7	14.2	7.3	≈0.0
D^{zz}_{79}	$z_1 + z_2$	25.7	14.2	7.3	≈0.0
D^{zz}_{80}	$z_2 + z_2$	25.2	14.5	7.3	≈0.0
D^{zz}_{81}	$z_1 + z_2$	25.9	14.2	7.3	≈0.0
D^{zz}_{82}	$z_2 + z_2$	26.0	14.4	7.3	≈0.0
D^{zz}_{83}	$z_1 + z_1$	25.4	13.9	7.3	≈0.0
D^{zz}_{84}	$z_1 + z_2$	25.7	14.1	7.3	≈0.0
D^{zz}_{85}	$z_1 + z_2$	25.9	14.1	7.3	≈0.0
D^{zz}_{86}	$z_2 + z_2$	27.2	14.4	7.4	≈0.0
D^{zz}_{87}	$z_1 + z_1$	26.8	13.7	7.5	≈0.0
D^{zz}_{88}	$z_2 + z_2$	26.9	14.3	7.5	≈0.0
D^{zz}_{89}	$z_2 + z_2$	26.4	14.3	7.5	≈0.0
D^{zz}_{90}	$z_2 + z_2$	26.1	14.2	7.5	≈0.0
D^{zz}_{91}	$z_1 + z_2$	26.3	13.9	7.6	≈0.0
D^{zz}_{92}	$z_1 + z_2$	25.4	13.9	7.6	≈0.0
D^{zz}_{93}	$z_1 + z_2$	26.0	13.9	7.6	≈0.0
D^{zz}_{94}	$z_1 + z_2$	25.1	13.8	7.7	≈0.0
D^{zz}_{95}	$z_2 + z_2$	26.3	14.1	7.7	≈0.0
D^{zz}_{96}	$z_2 + z_2$	25.1	14.0	7.7	≈0.0
D^{zz}_{97}	$z_2 + z_2$	26.2	14.0	7.8	≈0.0
D^{zz}_{98}	$z_2 + z_2$	26.5	13.8	7.9	≈0.0
D^{zz}_{99}	$z_2 + z_2$	26.4	13.8	8.0	≈0.0
D^{zz}_{100}	$z_1 + z_2$	24.7	13.5	8.0	≈0.0
D^{zz}_{101}	$z_1 + z_2$	25.3	13.5	8.0	≈0.0
D^{zz}_{102}	$z_2 + z_2$	24.8	13.8	8.0	≈0.0
D^{zz}_{103}	$z_1 + z_2$	25.9	13.5	8.0	≈0.0
D^{zz}_{104}	$z_2 + z_2$	25.8	13.8	8.0	≈0.0
D^{zz}_{105}	$z_2 + z_2$	26.2	13.7	8.0	≈0.0

**Dimer**	Composition	$\mathbf{BE}_{\mathrm{B3LYP-D3}}$	$\mathbf{BE}_{Gibbs}$	$\Delta\Delta$**G**	$\%\chi_i(G))$
D_{106}^{zz}	$z_2 + z_2$	25.9	13.7	8.0	≈0.0
D_{107}^{zz}	$z_1 + z_1$	25.1	13.1	8.1	≈0.0
D_{108}^{zz}	$z_2 + z_2$	26.0	13.7	8.1	≈0.0
D_{109}^{zz}	$z_2 + z_2$	25.8	13.7	8.1	≈0.0
D_{110}^{zz}	$z_2 + z_2$	26.1	13.7	8.1	≈0.0
D_{111}^{zz}	$z_2 + z_2$	25.9	13.6	8.2	≈0.0
D_{112}^{zz}	$z_2 + z_2$	24.8	13.5	8.3	≈0.0
D_{113}^{zz}	$z_2 + z_2$	25.5	13.5	8.3	≈0.0
D_{114}^{zz}	$z_1 + z_1$	23.8	12.8	8.4	≈0.0
D_{115}^{zz}	$z_2 + z_2$	25.8	13.3	8.4	≈0.0
D_{116}^{zz}	$z_1 + z_1$	25.0	12.7	8.5	≈0.0
D_{117}^{zz}	$z_1 + z_2$	24.0	13.0	8.5	≈0.0
D_{118}^{zz}	$z_2 + z_2$	25.7	13.2	8.5	≈0.0
D_{119}^{zz}	$z_2 + z_2$	24.9	13.1	8.6	≈0.0
D_{120}^{zz}	$z_1 + z_2$	24.5	12.8	8.7	≈0.0
D_{121}^{zz}	$z_2 + z_2$	24.7	13.0	8.8	≈0.0
D_{122}^{zz}	$z_2 + z_2$	24.8	12.8	8.9	≈0.0
D_{123}^{zz}	$z_2 + z_2$	25.0	12.8	9.0	≈0.0
D_{124}^{zz}	$z_1 + z_2$	24.5	12.5	9.0	≈0.0
D_{125}^{zz}	$z_2 + z_2$	24.0	12.8	9.0	≈0.0
D_{126}^{zz}	$z_2 + z_2$	24.9	12.7	9.1	≈0.0
D_{127}^{zz}	$z_2 + z_2$	24.0	12.7	9.1	≈0.0
D_{128}^{zz}	$z_2 + z_2$	23.6	12.6	9.2	≈0.0
D_{129}^{zz}	$z_1 + z_1$	22.0	11.3	9.9	≈0.0
D_{130}^{zz}	$z_1 + z_1$	22.7	11.2	10.0	≈0.0
D_{131}^{zz}	$z_2 + z_2$	24.6	11.8	10.0	≈0.0
D_{132}^{zz}	$z_1 + z_1$	22.7	11.2	10.0	≈0.0
D_{133}^{zz}	$z_1 + z_1$	23.7	11.2	10.0	≈0.0
D_{134}^{zz}	$z_1 + z_1$	22.6	10.8	10.4	≈0.0
D_{135}^{zz}	$z_1 + z_2$	23.2	10.8	10.7	≈0.0
D_{136}^{zz}	$z_1 + z_1$	21.9	10.5	10.7	≈0.0
D_{137}^{zz}	$z_1 + z_2$	23.0	10.7	10.8	≈0.0
D_{138}^{zz}	$z_2 + z_2$	20.8	10.3	11.4	≈0.0
D_{139}^{zz}	$z_1 + z_1$	20.6	9.8	11.4	≈0.0
D_{140}^{zz}	$z_1 + z_1$	21.3	9.7	11.5	≈0.0

**Dimer**	Composition	$\mathbf{BE}_{\text{B3LYP-D3}}$	$\mathbf{BE}_{Gibbs}$	$\Delta\Delta$**G**	$\%\chi_i(G)$)
D^{zz}_{141}	$z_1 + z_2$	21.7	9.9	11.6	≈0.0
D^{zz}_{142}	$z_1 + z_1$	21.1	9.3	11.9	≈0.0
D^{zz}_{143}	$z_1 + z_1$	21.7	9.1	12.1	≈0.0
D^{zz}_{144}	$z_2 + z_2$	22.2	9.4	12.4	≈0.0
D^{zz}_{145}	$z_2 + z_2$	21.7	9.4	12.4	≈0.0
D^{zz}_{146}	$z_1 + z_2$	21.6	8.9	12.6	≈0.0
D^{zz}_{147}	$z_1 + z_1$	19.2	8.6	12.6	≈0.0
D^{zz}_{148}	$z_1 + z_1$	19.9	8.4	12.8	≈0.0
D^{zz}_{149}	$z_1 + z_1$	20.2	8.3	12.9	≈0.0
D^{zz}_{150}	$z_2 + z_2$	21.1	8.8	12.9	≈0.0
D^{zz}_{151}	$z_1 + z_2$	19.8	8.5	13.0	≈0.0
D^{zz}_{152}	$z_2 + z_2$	20.4	8.7	13.1	≈0.0
D^{zz}_{153}	$z_1 + z_2$	20.6	8.3	13.2	≈0.0
D^{zz}_{154}	$z_1 + z_2$	19.7	8.3	13.2	≈0.0
D^{zz}_{155}	$z_2 + z_2$	20.3	8.5	13.2	≈0.0
D^{zz}_{156}	$z_2 + z_2$	20.0	8.2	13.5	≈0.0
D^{zz}_{157}	$z_1 + z_1$	18.0	7.6	13.6	≈0.0
D^{zz}_{158}	$z_2 + z_2$	20.0	8.0	13.8	≈0.0
D^{zz}_{159}	$z_2 + z_2$	19.9	8.0	13.8	≈0.0
D^{zz}_{160}	$z_1 + z_2$	19.0	7.6	13.9	≈0.0
D^{zz}_{161}	$z_2 + z_2$	18.8	7.6	14.2	≈0.0
D^{zz}_{162}	$z_1 + z_2$	19.2	7.2	14.3	≈0.0
D^{zz}_{163}	$z_2 + z_2$	20.1	7.5	14.3	≈0.0
D^{zz}_{164}	$z_2 + z_2$	20.2	7.4	14.3	≈0.0
D^{zz}_{165}	$z_1 + z_1$	18.3	6.9	14.3	≈0.0
D^{zz}_{166}	$z_1 + z_2$	18.5	7.1	14.4	≈0.0
D^{zz}_{167}	$z_1 + z_2$	18.1	7.1	14.4	≈0.0
D^{zz}_{168}	$z_1 + z_1$	17.8	6.8	14.4	≈0.0
D^{zz}_{169}	$z_1 + z_2$	17.6	7.0	14.5	≈0.0
D^{zz}_{170}	$z_2 + z_2$	18.4	7.2	14.6	≈0.0
D^{zz}_{171}	$z_1 + z_2$	17.7	6.7	14.8	≈0.0
D^{zz}_{172}	$z_2 + z_2$	18.2	6.9	14.9	≈0.0
D^{zz}_{173}	$z_1 + z_1$	17.3	6.3	14.9	≈0.0
D^{zz}_{174}	$z_1 + z_2$	16.7	6.4	15.1	≈0.0
D^{zz}_{175}	$z_1 + z_2$	18.0	6.3	15.2	≈0.0

**Dimer**	Composition	$\mathbf{BE}_{\mathrm{B3LYP-D3}}$	$\mathbf{BE}_{Gibbs}$	$\Delta\Delta$**G**	$\%\chi_i(G))$
D_{176}^{zz}	$z_2 + z_2$	18.0	6.6	15.2	≈0.0
D_{177}^{zz}	$z_1 + z_1$	17.9	6.0	15.2	≈0.0
D_{178}^{zz}	$z_2 + z_2$	17.9	6.5	15.3	≈0.0
D_{179}^{zz}	$z_2 + z_2$	17.1	6.4	15.4	≈0.0
D_{180}^{zz}	$z_2 + z_2$	17.1	5.9	15.9	≈0.0
D_{181}^{zz}	$z_1 + z_2$	15.6	4.8	16.7	≈0.0
D_{182}^{zz}	$z_1 + z_2$	16.6	4.7	16.8	≈0.0
D_{183}^{zz}	$z_1 + z_2$	15.8	4.3	17.1	≈0.0
D_{184}^{zz}	$z_1 + z_1$	12.6	1.7	19.5	≈0.0
D_{185}^{zz}	$z_1 + z_1$	13.2	1.3	19.9	≈0.0
D_{186}^{zz}	$z_1 + z_1$	4.1	-6.7	27.9	≈0.0

3 Laplacian of the electron density distributions

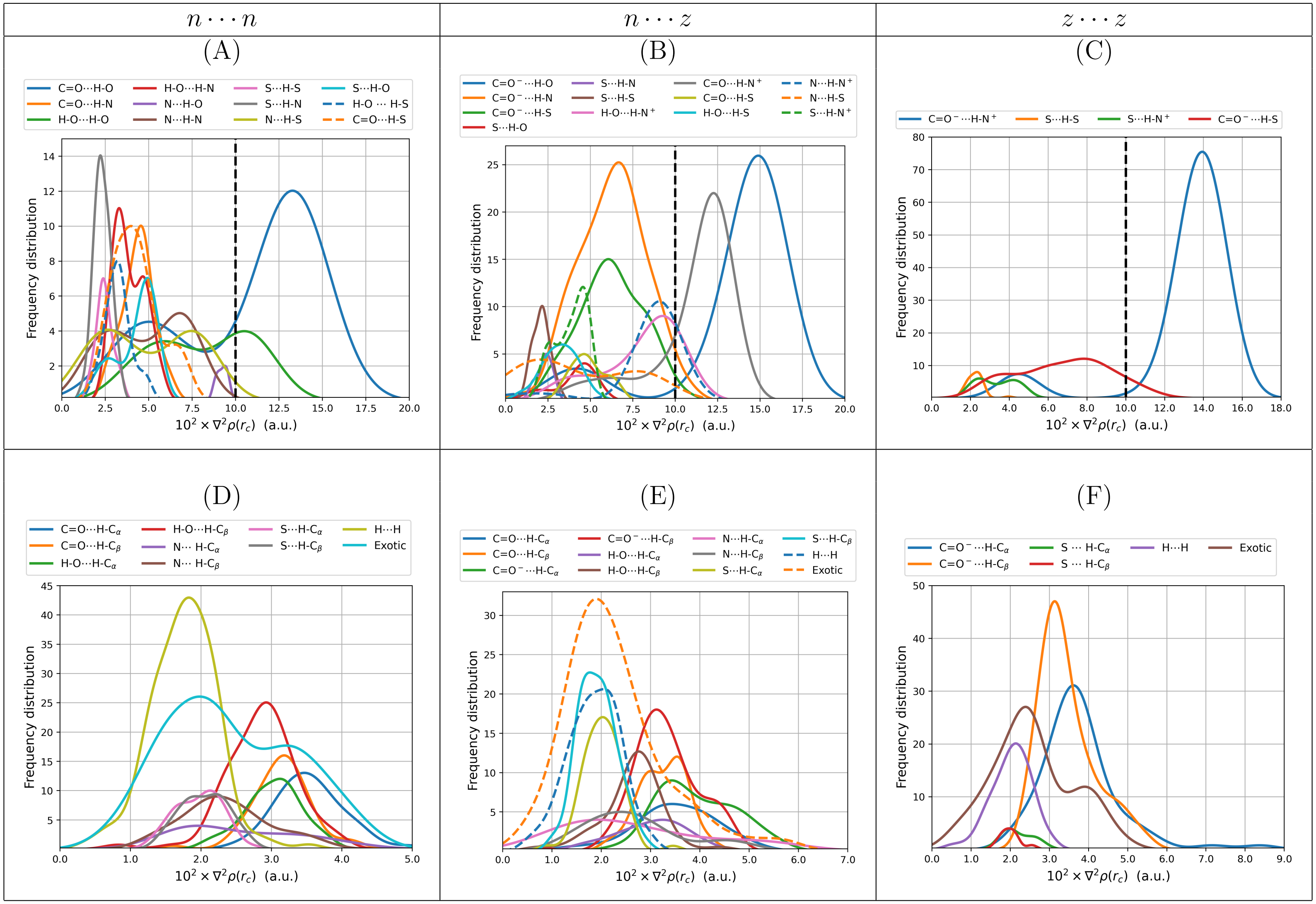

Figure S2: Laplacian of the electron densities at bond critical points for intermolecular contacts for all dimers of cysteine found in this work. The top row shows only primary hydrogen bonds including salt bridges. The bottom row shows secondary hydrogen bonds, dihydrogen bonds, and all exotic interactions. The left column is reserved for the $n \cdots n$ dimers, the middle column for $n \cdots z$ and the right column for $z \cdots z$. All values taken from the B3LYP–D3/6–311++G(d, p) potential energy surfaces with water represented as a continuum solvent. Dashed vertical lines mark the reference value for the gas phase water dimer.

4 NBO and NCI surfaces

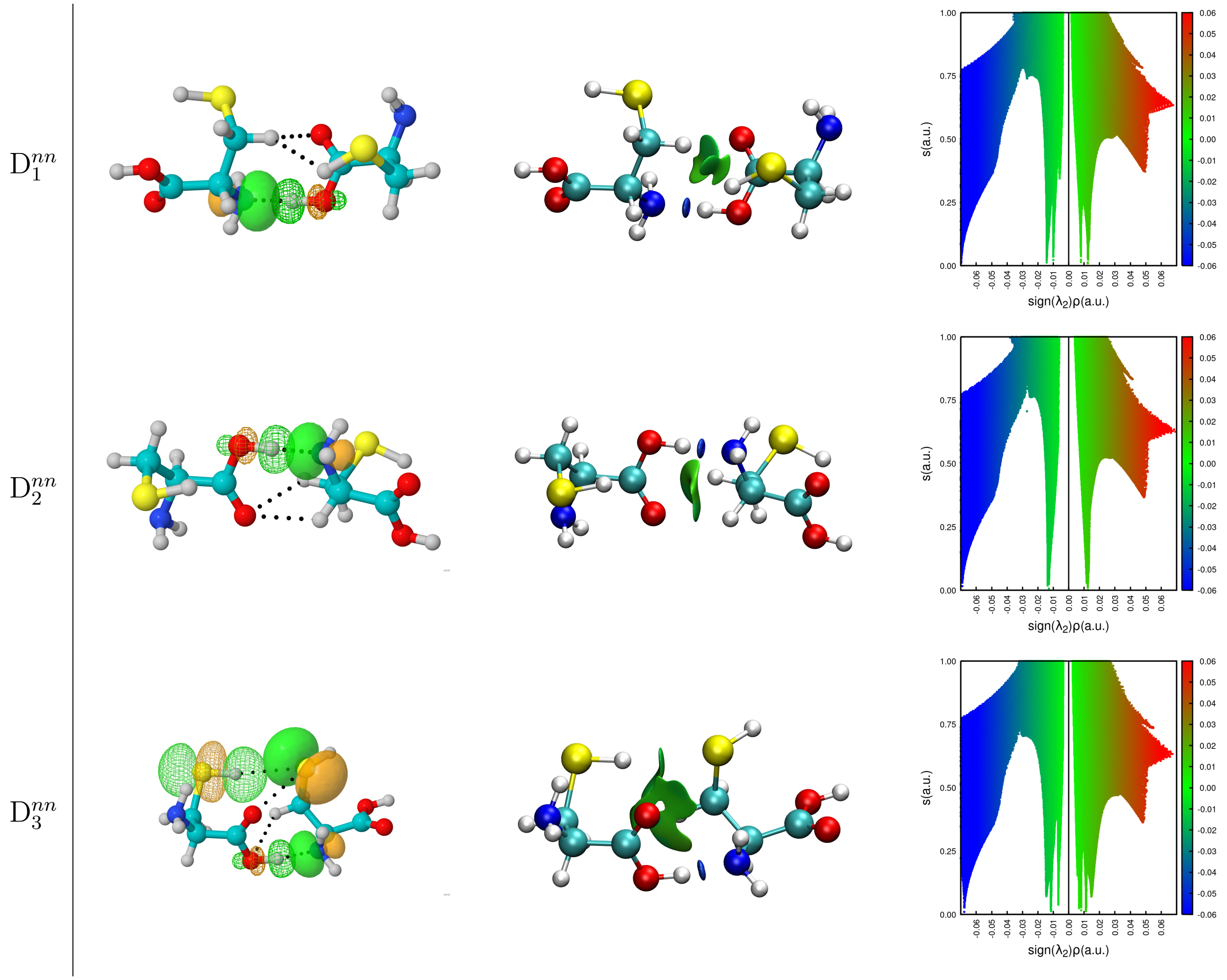

Figure S3: NBOs, NCI surfaces and thoroughs describing the intermolecular contacts for the $n \cdots n$ cysteine dimers with populations higher than 5%.

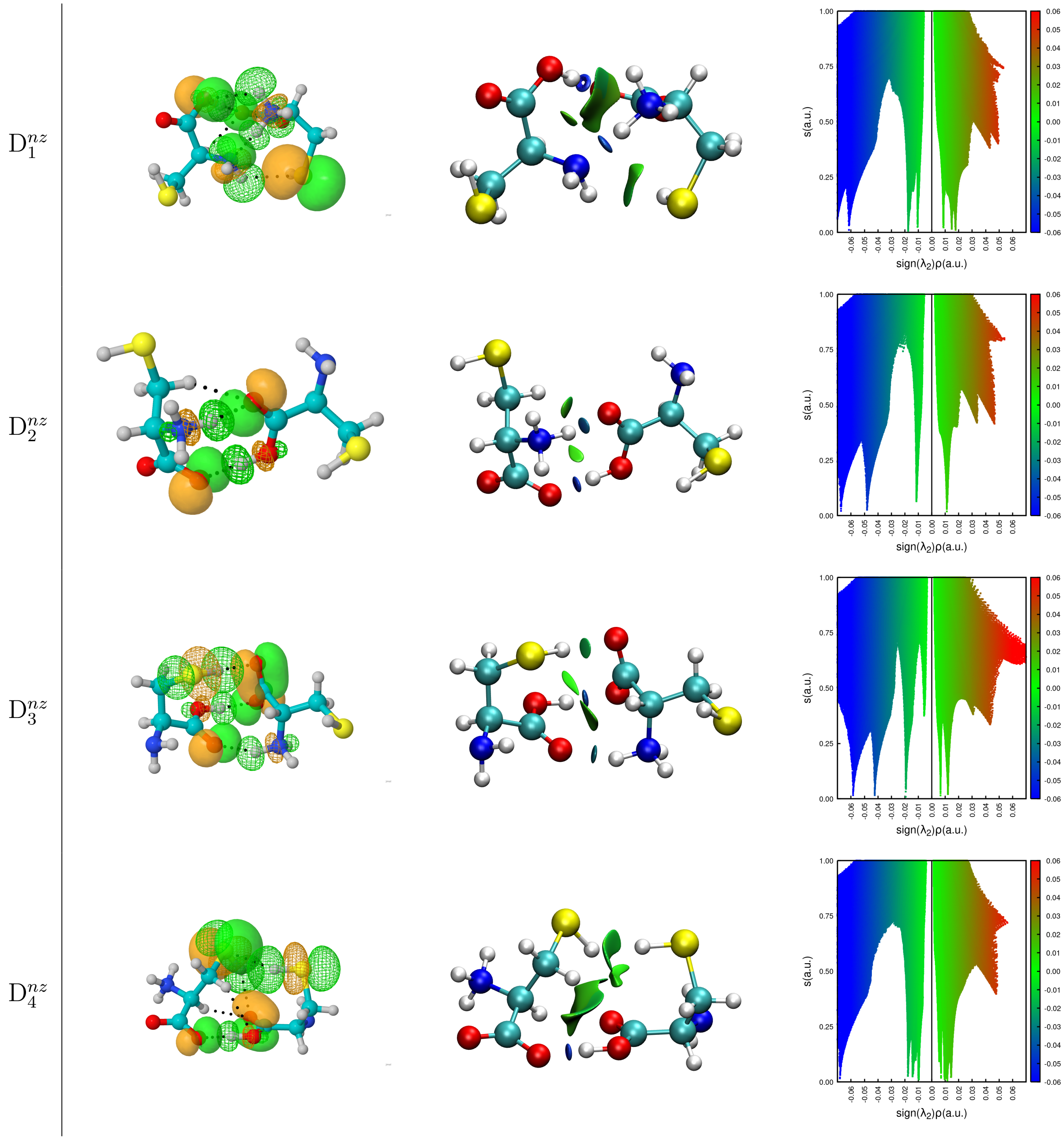

Figure S4: NBOs, NCI surfaces and thoroughs describing the intermolecular contacts for the $n \cdots z$ cysteine dimers with populations higher than 5%.

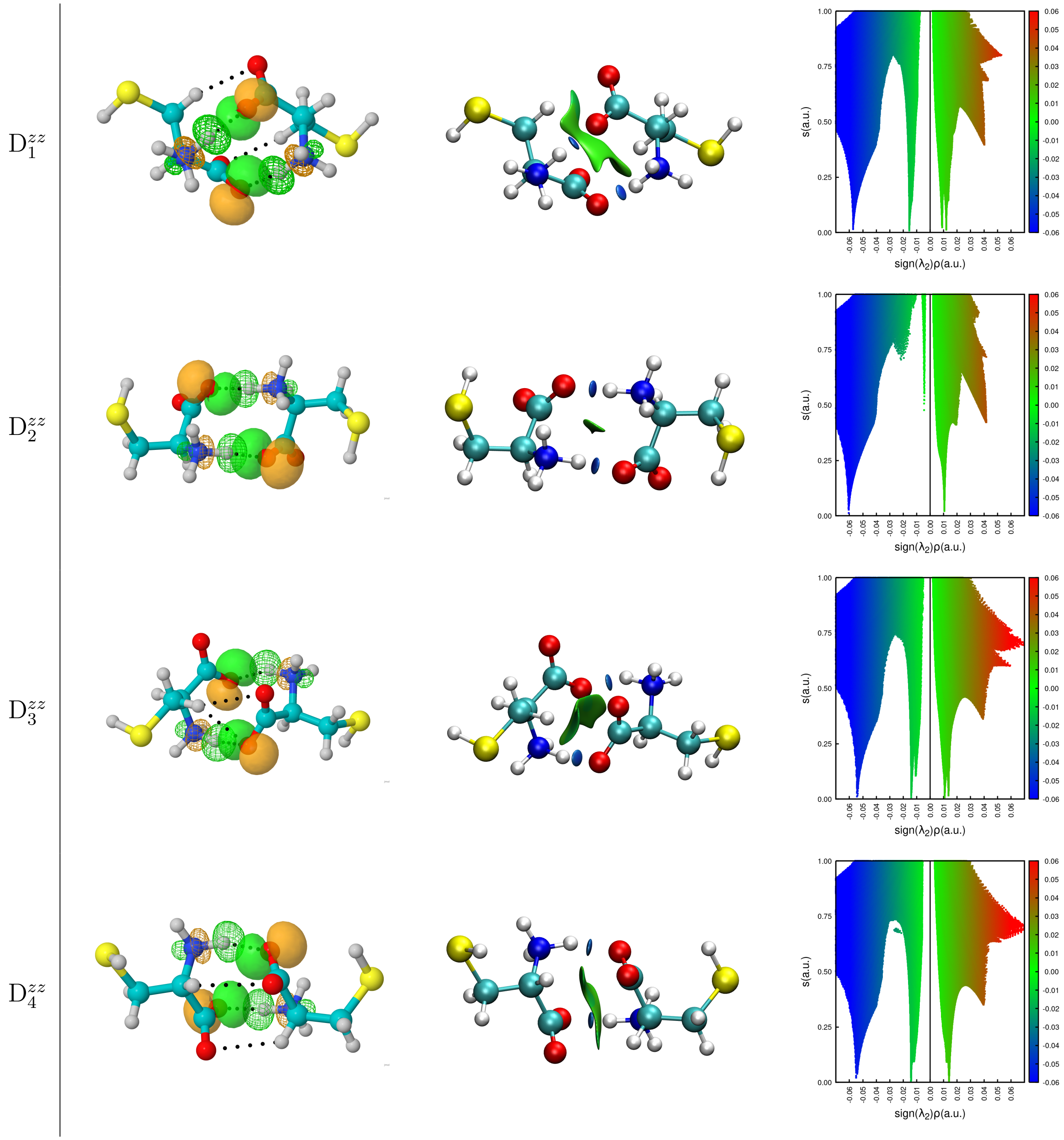

Figure S5: NBOs, NCI surfaces and thoroughs describing the intermolecular contacts for the $z \cdots z$ cysteine dimers with populations higher than 5%.

```
28
Dimer 1 of the n...n  type
C  -3.255452   0.495334   0.623992
C  -3.518970  -1.002092   0.409208
N  -3.875423   1.294425  -0.426063
H  -3.452093   1.053962  -1.319783
H  -3.659228   2.275665  -0.277475
H  -3.721300   0.759962   1.581256
C  -1.752201   0.763500   0.797845
O  -1.161356   1.642731   0.196874
O  -1.175646  -0.071587   1.649392
H  -0.154551   0.087083   1.672313
H  -3.243338  -1.571805   1.293514
H  -4.579308  -1.151143   0.206303
S  -2.625357  -1.722550  -1.043165
H  -1.425145  -1.818590  -0.437937
C   1.947061  -0.597683   0.454282
C   1.327569  -0.181710  -0.888404
N   1.448707   0.232773   1.552740
H   1.652370   1.211816   1.357231
H   1.937251   0.003891   2.414646
H   1.638131  -1.632652   0.640791
C   3.475136  -0.616124   0.391735
O   4.199472  -0.086411   1.199612
O   3.915727  -1.307850  -0.672455
H   4.886417  -1.312505  -0.670289
H   1.598230  -0.896296  -1.662193
H   0.244914  -0.159626  -0.795791
S   1.791759   1.514848  -1.441535
H   3.037799   1.200684  -1.853457
28
Dimer 2 of the n...n  type
C  -3.021964  -1.109549   0.091679
C  -3.962275  -0.232924  -0.749604
N  -3.558203  -1.331534   1.427661
H  -3.611574  -0.441071   1.916630
H  -2.910336  -1.901266   1.963362
H  -2.959649  -2.075836  -0.423166
C  -1.596893  -0.534905   0.078314
O  -0.967263  -0.296603   1.093835
O  -1.141611  -0.333890  -1.147856
H  -0.194052   0.077927  -1.131725
H  -3.630875  -0.204363  -1.785379
H  -4.968583  -0.649975  -0.709201
S  -4.131883   1.503744  -0.137669
H  -2.898360   1.915126  -0.496971
C   1.905093   0.490935   0.199937
C   2.090294  -1.011397   0.450777
N   1.284641   0.736442  -1.103073
H   1.877903   0.360031  -1.841014
H   1.218402   1.736619  -1.274527
H   1.214716   0.844551   0.971954
C   3.206649   1.268518   0.376055
O   3.642606   2.066715  -0.419218
O   3.809493   0.964607   1.538622
H   4.621504   1.489708   1.622797
H   2.435821  -1.181390   1.468017
H   1.127662  -1.504551   0.327358
S   3.240872  -1.843439  -0.727140
H   4.377306  -1.295452  -0.249307
28
Dimer 3 of the n...n  type
C   2.204763   0.494231   0.705661
C   1.297969  -0.739079   0.576171
N   1.617991   1.650872   0.027260
H   1.547977   1.464150  -0.972074
H   2.217966   2.464684   0.129912
H   2.279861   0.725499   1.773817
C   3.626991   0.197943   0.228171
```



```
O   4.233595   0.867244  -0.572585
O   4.122828  -0.902124   0.819533
H   5.027031  -1.055574   0.501551
H   1.668533  -1.548244   1.200678
H   0.298687  -0.478349   0.919961
S   1.122899  -1.361960  -1.155085
H   2.222640  -2.142486  -1.136202
C  -3.084425   0.942048   0.267240
C  -3.061442  -0.308069   1.169815
N  -4.040025   0.799260  -0.820334
H  -3.726368   0.069197  -1.454215
H  -4.077261   1.656516  -1.363288
H  -3.398719   1.772708   0.906436
C  -1.648341   1.238464  -0.178684
O  -1.219423   0.951955  -1.282063
O  -0.918780   1.777160   0.789291
H   0.066969   1.834539   0.496190
H  -2.355198  -0.164666   1.987296
H  -4.056263  -0.457126   1.590571
S  -2.689048  -1.901127   0.310118
H  -1.462076  -1.589476  -0.153307
28
Dimer 4 of the n...n  type
C   2.993992   0.744406  -0.564280
C   2.899805  -0.779387  -0.786846
N   4.128543   1.088348   0.281967
H   3.964571   0.741171   1.223250
H   4.211504   2.097814   0.355315
H   3.144470   1.189487  -1.551895
C   1.642551   1.240069  -0.041777
O   1.393712   1.361675   1.144959
O   0.769409   1.468083  -1.014371
H  -0.153017   1.644701  -0.602456
H   2.049891  -1.014593  -1.428288
H   3.812553  -1.117427  -1.278545
S   2.793127  -1.797776   0.748548
H   1.588536  -1.347479   1.154372
C  -2.597580   0.760608  -0.474081
C  -1.950976  -0.317024  -1.366777
N  -1.583587   1.572835   0.196086
H  -1.224954   1.085512   1.015591
H  -1.981491   2.446284   0.527541
H  -3.182747   1.404802  -1.136384
C  -3.575851   0.120265   0.509623
O  -3.452100   0.136850   1.710946
O  -4.581757  -0.494534  -0.134649
H  -5.168101  -0.915048   0.514746
H  -2.718564  -0.885760  -1.888256
H  -1.316415   0.172968  -2.104552
S  -0.828395  -1.474179  -0.475681
H  -1.782384  -2.169639   0.177076
28
Dimer 5 of the n...n  type
C   3.282958  -0.585161   0.103305
C   3.259882   0.728804   0.905649
N   3.873176  -0.389681  -1.211041
H   3.290521   0.246369  -1.750488
H   3.880208  -1.266460  -1.722923
H   3.903390  -1.289609   0.669379
C   1.874639  -1.181832   0.064681
O   1.312474  -1.485253  -0.978055
O   1.325171  -1.293910   1.262277
H   0.362150  -1.532959   1.179967
H   3.018209   0.538542   1.948903
H   4.245411   1.189307   0.846783
S   2.075414   1.992398   0.254090
H   0.961956   1.427796   0.764650
C  -3.282901  -0.585239  -0.103308
C  -3.259897   0.728727  -0.905647
```

```
N  -3.873139  -0.389809   1.211044
H  -3.290564   0.246313   1.750491
H  -3.880063  -1.266591   1.722924
H  -3.903306  -1.289707  -0.669388
C  -1.874569  -1.181878  -0.064696
O  -1.312393  -1.485331   0.978023
O  -1.325088  -1.293907  -1.262295
H  -0.362041  -1.532904  -1.179952
H  -3.018151   0.538477  -1.948885
H  -4.245476   1.189130  -0.846843
S  -2.075567   1.992442  -0.254060
H  -0.962041   1.427969  -0.764623
28
Dimer 6 of the n...n  type
C   2.994012  -0.341362  -0.006800
C   3.010077   1.117957  -0.477673
N   2.619909  -1.343515  -1.006473
H   1.972643  -0.961465  -1.689656
H   3.428076  -1.698951  -1.502180
H   4.002825  -0.576679   0.349721
C   2.105455  -0.509246   1.239776
O   2.081446   0.285049   2.151900
O   1.387362  -1.632128   1.244796
H   1.579359  -2.057696   0.369530
H   3.323722   1.768831   0.336995
H   3.714207   1.222313  -1.303220
S   1.390706   1.716896  -1.129715
H   0.725126   1.720989   0.045252
C  -2.828832  -0.315440   0.623478
C  -1.790954  -1.429580   0.432919
N  -3.956846  -0.311237  -0.312375
H  -3.693351  -0.727179  -1.202105
H  -4.753386  -0.817794   0.053495
H  -3.222156  -0.409178   1.641256
C  -2.152322   1.067437   0.598086
O  -1.108553   1.307224   1.165249
O  -2.826295   1.988071  -0.085703
H  -3.619214   1.499339  -0.432734
H  -0.982728  -1.321973   1.153400
H  -2.270099  -2.398152   0.577265
S  -1.064280  -1.494600  -1.260907
H  -0.365557  -0.340196  -1.183772
28
Dimer 7 of the n...n  type
C   3.280788   0.584037  -0.103978
C   3.249814  -0.724104  -0.915948
N   3.876265   0.377225   1.206121
H   3.292690  -0.258590   1.744789
H   3.891524   1.250884   1.723133
H   3.900710   1.290456  -0.668009
C   1.873984   1.183222  -0.055333
O   1.311179   1.467832   0.992320
O   1.325973   1.317946  -1.251272
H   0.363073   1.556235  -1.165696
H   3.000549  -0.525568  -1.955902
H   4.235211  -1.186151  -0.867761
S   2.069472  -1.991509  -0.264774
H   0.951320  -1.420413  -0.757624
C  -3.282174   0.582782   0.097786
C  -3.264971  -0.733810   0.895514
N  -3.862300   0.391475  -1.221848
H  -3.276381  -0.244382  -1.758047
H  -3.863175   1.269459  -1.731721
H  -3.907451   1.285314   0.661059
C  -1.874390   1.181848   0.071128
O  -1.311509   1.503843  -0.965604
O  -1.326582   1.275733   1.271132
H  -0.363531   1.516128   1.193654
H  -3.036584  -0.546872   1.942314
```



```
H  -4.248217  -1.197199   0.823139
S  -2.067176  -1.990433   0.254184
H  -0.965024  -1.425811   0.788577
28
Dimer 8 of the n...n  type
C  -1.897502  -0.563674   0.392203
C  -3.102016  -0.972407  -0.460326
N  -2.160512  -0.298830   1.808596
H  -3.106138   0.045622   1.950301
H  -2.028400  -1.127606   2.374278
H  -1.159871  -1.366328   0.327804
C  -1.175984   0.650561  -0.212629
O  -1.012118   0.818092  -1.400327
O  -0.715231   1.507727   0.700212
H  -1.001641   1.109951   1.565684
H  -2.782396  -1.185595  -1.479316
H  -3.554876  -1.869922  -0.038727
S  -4.453975   0.284996  -0.508374
H  -3.771013   1.205875  -1.219045
C   3.103995  -0.896558   0.092882
C   3.492472   0.477100   0.652958
N   3.360978  -1.119397  -1.332174
H   3.233588  -0.259037  -1.859833
H   4.302962  -1.453779  -1.494994
H   3.664387  -1.649648   0.658704
C   1.630280  -1.223715   0.387005
O   1.056204  -0.904693   1.403641
O   1.031952  -1.925830  -0.576808
H   1.711831  -1.987776  -1.296308
H   3.260630   0.524999   1.715991
H   4.563791   0.628358   0.519994
S   2.681140   1.892965  -0.207165
H   1.435307   1.674896   0.262849
28
Dimer 9 of the n...n  type
C   2.996793   0.340783   0.003221
C   3.014657  -1.118923   0.472765
N   2.625876   1.342436   1.004490
H   1.975351   0.961812   1.685420
H   3.434955   1.692285   1.502714
H   4.004483   0.576772  -0.356141
C   2.104468   0.509876  -1.240378
O   2.072909  -0.286326  -2.150584
O   1.391838   1.636318  -1.245386
H   1.587598   2.062106  -0.371155
H   3.327798  -1.768847  -0.342863
H   3.719925  -1.223406   1.297322
S   1.396483  -1.719715   1.125939
H   0.729548  -1.721161  -0.048189
C  -2.827907   0.320879  -0.622935
C  -1.782723   1.427680  -0.430352
N  -3.959163   0.325816   0.308956
H  -3.694421   0.737185   1.200462
H  -4.749114   0.841507  -0.058481
H  -3.217410   0.416293  -1.642059
C  -2.162109  -1.067135  -0.594263
O  -1.116439  -1.314639  -1.154498
O  -2.848346  -1.983342   0.083405
H  -3.639086  -1.488589   0.426647
H  -0.973582   1.314485  -1.148886
H  -2.254711   2.399520  -0.576275
S  -1.059693   1.488234   1.265115
H  -0.365728   0.330921   1.188660
28
Dimer 10 of the n...n  type
C  -1.231856   0.048852   0.038950
C  -2.061890  -1.093056  -0.550333
N  -1.044700   0.047584   1.490241
H  -1.900746  -0.238816   1.959482
```

```
H  -0.304803  -0.588624   1.770313
H  -0.244744  -0.005774  -0.427140
C  -1.753015   1.426399  -0.397477
O  -2.241638   1.648397  -1.481901
O  -1.579231   2.380630   0.519836
H  -1.218675   1.902998   1.308957
H  -2.089305  -1.014147  -1.636261
H  -1.595552  -2.039900  -0.279327
S  -3.788710  -1.169343   0.092365
H  -4.214091  -0.035186  -0.501069
C   2.982851  -0.735401  -0.362236
C   3.322439   0.638207   0.229930
N   2.610754  -0.762207  -1.779196
H   2.106882   0.081172  -2.042337
H   3.422669  -0.843192  -2.379113
H   3.862808  -1.375485  -0.232577
C   1.875139  -1.431686   0.443588
O   1.736041  -1.313750   1.642059
O   1.059302  -2.183284  -0.294022
H   1.362432  -2.035978  -1.227846
H   3.646697   0.534286   1.263982
H   4.131225   1.084935  -0.348150
S   1.929124   1.849050   0.164009
H   1.228383   1.324929   1.189496
28
Dimer 11 of the n...n  type
C  -2.719625   0.961317  -0.068753
C  -3.312534  -0.370200  -0.553704
N  -1.953357   1.735689  -1.039305
H  -1.352480   1.139332  -1.602864
H  -2.557114   2.264395  -1.656594
H  -3.558447   1.580771   0.268679
C  -1.870062   0.750338   1.194920
O  -2.164889  -0.021468   2.079005
O  -0.766468   1.499807   1.252588
H  -0.721037   1.984970   0.391601
H  -3.880818  -0.840631   0.247604
H  -3.980725  -0.179173  -1.393648
S  -2.060616  -1.565787  -1.188773
H  -1.441644  -1.794190  -0.009750
C   2.742453  -0.366590  -0.761711
C   1.662067  -1.431204  -0.533399
N   3.944526  -0.467233   0.070176
H   3.734086  -0.910777   0.960706
H   4.676026  -0.998577  -0.385428
H   3.042265  -0.429344  -1.813208
C   2.148283   1.047537  -0.616618
O   1.068757   1.366412  -1.064028
O   2.939734   1.901482   0.027423
H   3.729918   1.355019   0.281703
H   0.804909  -1.225861  -1.173176
H   2.061064  -2.415350  -0.779074
S   1.092072  -1.560802   1.215867
H   0.594739  -0.311540   1.313347
28
Dimer 12 of the n...n  type
C  -3.055216  -0.591789  -0.352445
C  -3.401708   0.902110  -0.351130
N  -3.065273  -1.271141   0.945420
H  -2.765837  -0.641350   1.686005
H  -3.986639  -1.620207   1.179442
H  -3.783698  -1.091251  -1.001351
C  -1.703081  -0.856999  -1.037062
O  -1.295623  -0.240133  -1.996215
O  -1.017455  -1.861449  -0.492516
H  -1.557263  -2.129384   0.296719
H  -3.336511   1.299070  -1.363142
H  -4.420822   1.034215   0.012403
S  -2.349464   1.925226   0.767784
```



```
H  -1.198947   1.793249   0.072598
C   1.643366  -0.187321  -0.559886
C   3.108457  -0.162692  -1.009667
N   0.953444   1.104812  -0.549875
H   1.596605   1.864725  -0.344242
H   0.513675   1.277729  -1.446139
H   1.099770  -0.845401  -1.242399
C   1.500219  -0.865216   0.811687
O   2.128677  -1.847296   1.137170
O   0.607082  -0.293911   1.622015
H   0.271315   0.487655   1.123944
H   3.510421  -1.174323  -1.034320
H   3.169451   0.265172  -2.010479
S   4.199028   0.890513   0.044045
H   4.144602   0.106330   1.140273
28
Dimer 13 of the n...n  type
C   2.928047   0.602084   0.192906
C   3.031131  -0.399173  -0.964194
N   3.044244   0.053006   1.546764
H   2.624646  -0.872473   1.597651
H   4.011111  -0.022290   1.838759
H   3.729375   1.338006   0.059580
C   1.636895   1.433798   0.110462
O   1.114858   1.778064  -0.925036
O   1.139950   1.772078   1.302921
H   1.721221   1.303634   1.955696
H   2.923300   0.118876  -1.916011
H   4.010599  -0.876562  -0.934281
S   1.803452  -1.773557  -0.884430
H   0.720428  -1.061529  -1.271778
C  -1.801996   0.769156   0.270025
C  -1.154202  -0.313454   1.137658
N  -3.086564   1.293233   0.742963
H  -3.642456   0.556729   1.171130
H  -2.958305   2.029069   1.427098
H  -1.095889   1.602821   0.211692
C  -1.967035   0.317140  -1.188426
O  -1.198081  -0.421493  -1.765615
O  -3.035067   0.835953  -1.791325
H  -3.505888   1.332108  -1.072828
H  -0.163578  -0.563067   0.769845
H  -1.052211   0.062549   2.155175
S  -2.134376  -1.869350   1.264093
H  -1.941643  -2.259146  -0.012574
28
Dimer 14 of the n...n  type
C  -2.492463  -0.737212   0.845934
C  -3.090697   0.656887   0.630445
N  -1.288801  -0.814151   1.678460
H  -0.709049   0.019220   1.603278
H  -1.517706  -0.946304   2.655859
H  -3.267233  -1.354092   1.315165
C  -2.209590  -1.437552  -0.496938
O  -2.898832  -1.311993  -1.483033
O  -1.128798  -2.220474  -0.468022
H  -0.765553  -2.089379   0.447075
H  -4.032986   0.582202   0.089934
H  -3.279242   1.119925   1.599062
S  -1.980255   1.821197  -0.274903
H  -2.134622   1.250609  -1.486868
C   1.307715   0.107590  -0.615140
C   1.858549  -1.160455   0.046019
N   1.891190   0.480181  -1.904519
H   2.866762   0.198403  -1.958964
H   1.393493   0.056178  -2.677814
H   0.235277  -0.041311  -0.763448
C   1.389090   1.309044   0.340154
O   1.155639   1.230121   1.527781
```

```
O   1.713266   2.452097  -0.253817
H   1.859396   2.201004  -1.203222
H   1.350649  -1.337941   0.992438
H   1.685927  -2.011513  -0.612442
S   3.677054  -1.118812   0.348910
H   3.643547  -0.175186   1.312128
28
Dimer 15 of the n...n  type
C   1.306489   0.107255  -0.614514
C   1.858168  -1.159973   0.047466
N   1.888804   0.478723  -1.904757
H   2.864324   0.196931  -1.959859
H   1.390361   0.054114  -2.677236
H   0.234038  -0.042100  -0.761904
C   1.388377   1.309735   0.339458
O   1.156241   1.231883   1.527404
O   1.711466   2.452249  -0.256153
H   1.856763   2.200003  -1.205392
H   1.350860  -1.336782   0.994336
H   1.685530  -2.011705  -0.610119
S   3.676823  -1.117451   0.349259
H   3.643615  -0.172988   1.311655
C  -2.492237  -0.737963   0.845166
C  -3.091882   0.655366   0.628548
N  -1.289656  -0.813455   1.679325
H  -0.710201   0.020082   1.603695
H  -1.519839  -0.944258   2.656603
H  -3.266928  -1.355636   1.313515
C  -2.206919  -1.438308  -0.497086
O  -2.894291  -1.312850  -1.484478
O  -1.126132  -2.221274  -0.466216
H  -0.764320  -2.090048   0.449336
H  -4.032786   0.579441   0.085808
H  -3.283246   1.117984   1.596815
S  -1.980823   1.821334  -0.273762
H  -2.128458   1.249225  -1.485858
28
Dimer 16 of the n...n  type
C  -2.949066  -0.611656   0.168361
C  -3.012957   0.375413  -1.004024
N  -3.145884  -0.047276   1.506592
H  -2.772235   0.897366   1.560007
H  -4.125919  -0.014323   1.759661
H  -3.726269  -1.366384   0.002552
C  -1.636080  -1.412797   0.158209
O  -1.079961  -1.788787  -0.848320
O  -1.161204  -1.683402   1.376037
H  -1.780589  -1.213522   1.992494
H  -2.852059  -0.150762  -1.943785
H  -3.999664   0.838059  -1.025463
S  -1.808419   1.768280  -0.881550
H  -0.704173   1.070901  -1.235607
C   1.818046  -0.767765   0.292877
C   1.199229   0.340767   1.148280
N   3.112166  -1.287676   0.744025
H   3.686515  -0.542767   1.131359
H   2.998785  -1.999914   1.455234
H   1.105817  -1.598112   0.275447
C   1.946230  -0.356888  -1.181315
O   1.173469   0.379731  -1.755951
O   2.987103  -0.910045  -1.801223
H   3.473373  -1.391406  -1.083120
H   0.198903   0.582753   0.802741
H   1.124846  -0.007295   2.178136
S   2.189381   1.894677   1.206101
H   1.965628   2.250340  -0.075452
28
Dimer 17 of the n...n  type
C  -3.085244   0.199527   0.026518
```



```
C  -3.002971  -1.331519   0.036782
N  -2.895797   0.871934  -1.259273
H  -2.127181   0.452763  -1.777172
H  -3.730180   0.821195  -1.831454
H  -4.083591   0.468533   0.392573
C  -2.130897   0.830264   1.052917
O  -1.777524   0.284057   2.074207
O  -1.737129   2.063524   0.734997
H  -2.073720   2.208969  -0.183085
H  -3.203074  -1.706784   1.039648
H  -3.755951  -1.732838  -0.641959
S  -1.396487  -2.017718  -0.546318
H  -0.647826  -1.607826   0.495986
C   2.635765   0.518838   0.662534
C   1.263184   1.132045   0.363810
N   3.784560   1.123837  -0.017399
H   3.519006   1.476209  -0.933698
H   4.175455   1.890612   0.516258
H   2.798766   0.599617   1.743040
C   2.636916  -0.995664   0.389759
O   1.703261  -1.723292   0.646733
O   3.776303  -1.448647  -0.129772
H   4.329797  -0.633674  -0.252164
H   0.493818   0.616504   0.933796
H   1.258334   2.180794   0.658486
S   0.813534   1.121765  -1.423907
H   0.532470  -0.195301  -1.503202
28
Dimer 18 of the n...n  type
C   1.828842   0.755758  -0.331300
C   1.229542  -0.386360  -1.155385
N   3.137205   1.253009  -0.766366
H   3.724870   0.489243  -1.092110
H   3.046927   1.924090  -1.519706
H   1.120376   1.588446  -0.371287
C   1.911834   0.414186   1.163622
O   1.132692  -0.308995   1.746421
O   2.920987   1.014868   1.792169
H   3.428769   1.464895   1.069210
H   0.214867  -0.603322  -0.835673
H   1.193933  -0.084826  -2.202026
S   2.208138  -1.948095  -1.111576
H   1.947389  -2.240476   0.178924
C  -2.943985   0.620462  -0.159764
C  -2.981791  -0.346208   1.031082
N  -3.189512   0.034625  -1.480664
H  -2.840981  -0.920010  -1.523943
H  -4.176053   0.022135  -1.708799
H  -3.706479   1.387155   0.018219
C  -1.621634   1.405084  -0.203622
O  -1.042626   1.809770   0.778548
O  -1.166837   1.626008  -1.438864
H  -1.808889   1.148593  -2.026254
H  -2.774835   0.192302   1.954742
H  -3.975571  -0.789086   1.098005
S  -1.810574  -1.765628   0.887280
H  -0.679779  -1.083759   1.184645
28
Dimer 19 of the n...n  type
C   1.820497   0.765346  -0.302181
C   1.205242  -0.350787  -1.150154
N   3.118318   1.278535  -0.750427
H   3.694632   0.528603  -1.124923
H   3.010627   1.982475  -1.470726
H   1.109604   1.597008  -0.297847
C   1.938554   0.370743   1.177291
O   1.163068  -0.360507   1.755089
O   2.974112   0.932606   1.798342
H   3.465498   1.405505   1.078149
```

```
H   0.201448  -0.586139  -0.810269
H   1.139724  -0.013467  -2.184215
S   2.191873  -1.907646  -1.184412
H   1.960329  -2.247916   0.099941
C  -2.948113   0.613918  -0.164687
C  -3.004370  -0.368426   1.012161
N  -3.158932   0.044459  -1.498674
H  -2.791705  -0.902789  -1.550390
H  -4.141094   0.016538  -1.743974
H  -3.721322   1.371767   0.005378
C  -1.632408   1.410714  -0.169391
O  -1.069417   1.793760   0.830643
O  -1.163454   1.668752  -1.392162
H  -1.789348   1.197272  -2.000885
H  -2.831560   0.160726   1.948143
H  -3.992764  -0.826773   1.045418
S  -1.807150  -1.767214   0.882908
H  -0.696227  -1.071933   1.220437
28
Dimer 20 of the n...n  type
C  -1.525582  -0.265653  -0.043943
C  -2.600814  -1.016466  -0.836054
N  -1.234016  -0.769648   1.298366
H  -2.053791  -1.197835   1.719476
H  -0.480364  -1.447204   1.262787
H  -0.601527  -0.329799  -0.623473
C  -1.841753   1.237267   0.034468
O  -2.302305   1.874235  -0.886158
O  -1.545959   1.787051   1.212802
H  -1.246205   1.021607   1.769129
H  -2.699016  -0.588443  -1.832486
H  -2.312303  -2.063641  -0.929043
S  -4.257430  -1.029846  -0.022360
H  -4.505710   0.287068  -0.176253
C   2.923084  -0.200486   0.743582
C   1.890308   0.932241   0.761871
N   4.273266   0.150478   0.298698
H   4.245958   0.891869  -0.397020
H   4.854593   0.459292   1.068088
H   2.990312  -0.591584   1.764936
C   2.422320  -1.396222  -0.088394
O   1.266720  -1.764816  -0.106246
O   3.378919  -2.011767  -0.774023
H   4.193046  -1.469881  -0.598763
H   0.941387   0.559109   1.138069
H   2.235707   1.725269   1.425233
S   1.631337   1.754243  -0.867378
H   1.196279   0.666299  -1.534492
28
Dimer 21 of the n...n  type
C  -3.113581   0.394002  -0.078962
C  -3.082963  -1.020355   0.513997
N  -3.294221   0.488463  -1.530052
H  -2.827790  -0.280621  -2.004839
H  -4.273121   0.473865  -1.789016
H  -3.941632   0.930421   0.398418
C  -1.861415   1.195114   0.311217
O  -1.297616   1.100187   1.376265
O  -1.440125   2.029473  -0.645330
H  -2.046168   1.853019  -1.411918
H  -2.965431  -0.972753   1.595431
H  -4.025140  -1.519286   0.286800
S  -1.756452  -2.103406  -0.181466
H  -0.748878  -1.673480   0.607850
C   1.317144   0.302509  -0.320599
C   2.193048  -0.654560  -1.138695
N   1.622166   1.725402  -0.486015
H   2.413311   1.886847  -1.098580
H   0.814342   2.234174  -0.825205
```



```
H   0.288044   0.096537  -0.618286
C   1.407859  -0.058084   1.172930
O   1.135372  -1.160144   1.604846
O   1.843380   0.931320   1.938935
H   1.939354   1.690895   1.297783
H   1.895622  -1.682206  -0.934141
H   2.049562  -0.454378  -2.200961
S   4.008510  -0.479845  -0.854372
H   4.010743  -0.906511   0.425477
28
Dimer 22 of the n...n  type
C  -1.643365  -0.178708   0.627496
C  -3.145851  -0.239540   0.919451
N  -1.015635   1.139423   0.737716
H  -1.681946   1.886832   0.567111
H  -0.592100   1.266916   1.648418
H  -1.143274  -0.850018   1.330373
C  -1.323549  -0.782459  -0.751295
O  -1.855211  -1.778516  -1.186624
O  -0.376544  -0.124938  -1.423986
H  -0.171138   0.662963  -0.858930
H  -3.498472  -1.266950   0.844441
H  -3.332450   0.124854   1.929740
S  -4.169178   0.828271  -0.185997
H  -3.954795   0.120331  -1.313911
C   2.923271  -0.683011   0.141587
C   3.436753   0.760341   0.160883
N   2.630242  -1.279536  -1.162008
H   2.091444  -0.639510  -1.740989
H   3.478300  -1.526691  -1.657944
H   3.698284  -1.300024   0.613816
C   1.696073  -0.883193   1.042988
O   1.417820  -0.189367   1.994125
O   0.955897  -1.940846   0.697747
H   1.335100  -2.247247  -0.162979
H   3.641721   1.068073   1.185613
H   4.364278   0.813385  -0.409785
S   2.311620   1.994538  -0.617167
H   1.368592   1.968925   0.345231
28
Dimer 23 of the n...n  type
C   2.692755  -0.504102  -0.632530
C   1.362977  -1.221590  -0.373672
N   3.848442  -0.974450   0.136531
H   3.560881  -1.322748   1.047650
H   4.345805  -1.713771  -0.344349
H   2.921170  -0.619118  -1.697831
C   2.549791   1.016075  -0.435742
O   1.583340   1.650775  -0.797216
O   3.604794   1.585930   0.142763
H   4.214268   0.826731   0.339527
H   0.579165  -0.794032  -0.994552
H   1.464019  -2.276548  -0.627319
S   0.828769  -1.192612   1.390977
H   0.433961   0.097965   1.415874
C  -3.065626  -0.227143  -0.054585
C  -3.018923   1.302573   0.027154
N  -2.894559  -0.970162   1.194440
H  -2.151916  -0.564874   1.758605
H  -3.745420  -0.972860   1.744207
H  -4.048619  -0.494862  -0.461176
C  -2.074716  -0.779311  -1.091220
O  -1.696494  -0.165438  -2.063760
O  -1.681246  -2.029432  -0.845087
H  -2.033421  -2.234757   0.055122
H  -3.215525   1.733027  -0.954000
H  -3.789483   1.644819   0.718296
S  -1.434960   1.991145   0.668426
H  -0.673709   1.673106  -0.397334
```

```
28
Dimer 24 of the n...n  type
C   2.113182  -0.217223   0.794167
C   1.962636   1.275719   0.483456
N   3.382468  -0.641282   1.389431
H   4.159036  -0.111158   1.001186
H   3.383661  -0.511302   2.393956
H   1.310010  -0.483835   1.488765
C   1.838835  -1.078935  -0.447932
O   1.045810  -0.779230  -1.312882
O   2.532455  -2.216390  -0.477394
H   3.134983  -2.159350   0.307090
H   0.962762   1.483610   0.109781
H   2.112740   1.847541   1.399265
S   3.206182   1.920432  -0.717660
H   2.719046   1.264630  -1.790630
C  -1.878065   0.015845  -0.712786
C  -2.971815   1.083151  -0.606303
N  -2.258915  -1.249632  -1.343913
H  -3.242502  -1.455190  -1.190188
H  -2.084199  -1.238756  -2.340929
H  -1.048387   0.442451  -1.283049
C  -1.259299  -0.283367   0.660886
O  -1.019240   0.568369   1.489087
O  -0.959316  -1.566123   0.851937
H  -1.265509  -2.009655   0.016824
H  -2.565328   1.990351  -0.161660
H  -3.346196   1.316930  -1.603059
S  -4.449682   0.550256   0.363813
H  -3.829488   0.504968   1.560664
28
Dimer 25 of the n...n  type
C  -1.557540  -0.327594   0.122470
C  -1.587257   1.092230  -0.451704
N  -1.614251  -1.426394  -0.844131
H  -2.057225  -1.142712  -1.712691
H  -0.699501  -1.816171  -1.041029
H  -0.630896  -0.407996   0.696715
C  -2.680108  -0.517182   1.159935
O  -2.994176   0.326720   1.970334
O  -3.266164  -1.711537   1.099099
H  -2.829081  -2.148152   0.318308
H  -1.511531   1.815605   0.356652
H  -0.737477   1.240808  -1.115311
S  -3.082022   1.460366  -1.469735
H  -3.967553   1.430519  -0.452621
C   2.854528   0.594612  -0.609847
C   1.946099  -0.480625  -1.215584
N   4.265647   0.245261  -0.440638
H   4.372318  -0.738360  -0.205262
H   4.800832   0.428676  -1.280602
H   2.799149   1.469325  -1.267623
C   2.299991   1.096738   0.736246
O   1.116852   1.194000   0.976022
O   3.242428   1.440881   1.610768
H   4.094639   1.199118   1.165543
H   0.939440  -0.087573  -1.337460
H   2.325446  -0.766289  -2.196700
S   1.875836  -2.050616  -0.250468
H   1.394842  -1.514440   0.889830
28
Dimer 26 of the n...n  type
C  -2.927298  -0.017988   0.638196
C  -2.272256  -1.379399   0.374802
N  -3.952315   0.405390  -0.319650
H  -3.764087   0.023153  -1.243206
H  -4.878250   0.110198  -0.035294
H  -3.385816  -0.066900   1.631908
C  -1.874684   1.099372   0.768550
```



```
O  -0.823265   0.965617   1.356819
O  -2.242057   2.249980   0.213938
H  -3.124021   2.049662  -0.198389
H  -1.522829  -1.588719   1.136284
H  -3.035132  -2.157102   0.416231
S  -1.508391  -1.536564  -1.295341
H  -0.588182  -0.555989  -1.159385
C   2.927370  -0.018212  -0.638046
C   2.272106  -1.379584  -0.374778
N   3.952283   0.405078   0.319925
H   3.763357   0.023554   1.243635
H   4.878075   0.108864   0.036158
H   3.386075  -0.067143  -1.631672
C   1.874947   1.099265  -0.768549
O   0.823417   0.965631  -1.356615
O   2.242572   2.250022  -0.214281
H   3.124499   2.049838   0.198027
H   1.522896  -1.588864  -1.136470
H   3.034974  -2.157308  -0.416033
S   1.508015  -1.536658   1.295165
H   0.588226  -0.555704   1.159173
28
Dimer 27 of the n...n  type
C   3.137619  -0.489123   0.565300
C   2.563499   0.865442   1.004198
N   4.351287  -0.442108  -0.256631
H   4.377725   0.405260  -0.818251
H   5.191622  -0.477760   0.307052
H   3.361840  -1.056219   1.475417
C   2.078044  -1.339206  -0.157934
O   0.932877  -1.442030   0.220404
O   2.542574  -1.990361  -1.223301
H   3.495363  -1.714699  -1.278866
H   1.634483   0.721633   1.553489
H   3.287431   1.365018   1.648100
S   2.239376   2.033227  -0.387719
H   1.042515   1.525200  -0.755578
C  -2.124305  -0.826276   0.306304
C  -3.270910  -0.906718  -0.706742
N  -2.498881  -0.884589   1.721575
H  -3.405643  -0.451725   1.878069
H  -2.540133  -1.838713   2.058262
H  -1.439190  -1.652961   0.100065
C  -1.261893   0.421691   0.071368
O  -0.988780   0.848426  -1.031013
O  -0.818132   0.994083   1.186728
H  -1.212498   0.446903   1.913930
H  -2.875248  -0.898663  -1.721259
H  -3.824339  -1.833044  -0.552087
S  -4.516634   0.446058  -0.539190
H  -3.705276   1.442289  -0.949944
28
Dimer 28 of the n...n  type
C  -3.132448  -0.428296  -0.266063
C  -3.229167   0.898264   0.495595
N  -3.120123  -1.649251   0.543528
H  -2.612566  -1.503084   1.412984
H  -4.057507  -1.956841   0.772761
H  -3.996510  -0.481140  -0.938439
C  -1.915757  -0.450182  -1.206165
O  -1.503204   0.518716  -1.800510
O  -1.357612  -1.658068  -1.330660
H  -1.882751  -2.233052  -0.714505
H  -3.270195   1.731670  -0.203994
H  -4.139074   0.899398   1.095743
S  -1.845736   1.188237   1.683488
H  -0.917062   1.530105   0.763907
C   2.077105  -0.880214  -0.277015
C   1.726834   0.144956  -1.360755
```

```
N   1.047182  -1.169977   0.722822
H   0.542394  -0.326867   0.983272
H   0.368989  -1.827693   0.360294
H   2.310346  -1.819837  -0.791647
C   3.381942  -0.513389   0.450958
O   4.317175   0.041570  -0.080391
O   3.406072  -0.892113   1.730030
H   2.502627  -1.264202   1.895567
H   2.552093   0.235339  -2.065669
H   0.833361  -0.178705  -1.891205
S   1.308411   1.819876  -0.715705
H   2.523141   2.092026  -0.195112
28
Dimer 29 of the n...n  type
C   3.100110   1.027208  -0.017870
C   3.865272  -0.259013  -0.352273
N   2.922043   1.338921   1.401846
H   2.802476   0.487322   1.945438
H   3.715944   1.843730   1.776872
H   3.646470   1.858303  -0.478554
C   1.724012   1.056398  -0.705455
O   1.504620   0.581148  -1.796968
O   0.784898   1.687716   0.000044
H   1.228935   1.907372   0.859642
H   3.934842  -0.379170  -1.432383
H   4.872396  -0.195960   0.060216
S   3.130350  -1.782718   0.381797
H   1.975060  -1.752198  -0.313790
C  -2.707200   0.537278  -0.597002
C  -1.610843  -0.442298  -1.028133
N  -2.285147   1.632846   0.281620
H  -1.540031   1.314330   0.896677
H  -1.902495   2.400507  -0.257436
H  -3.129732   0.977202  -1.507735
C  -3.889099  -0.196103   0.060682
O  -4.289207  -1.282201  -0.294850
O  -4.457988   0.489926   1.052664
H  -3.893763   1.302283   1.140619
H  -2.016560  -1.186953  -1.711190
H  -0.810100   0.101552  -1.525692
S  -0.801900  -1.310577   0.384482
H  -1.829009  -2.141504   0.655791
28
Dimer 30 of the n...n  type
C   2.923512  -0.322064   0.702128
C   2.350288   1.098524   0.780492
N   4.079775  -0.504347  -0.179524
H   4.033600   0.136578  -0.968250
H   4.953266  -0.349114   0.308559
H   3.218388  -0.612383   1.717228
C   1.859760  -1.362034   0.315148
O   0.708943  -1.345742   0.716107
O   2.309171  -2.324367  -0.469394
H   3.253644  -2.061109  -0.653663
H   1.518836   1.136451   1.481967
H   3.131357   1.766667   1.143938
S   1.817340   1.789444  -0.843302
H   0.738390   0.963794  -1.047850
C  -2.049717   0.666992  -0.859753
C  -3.378882  -0.121313  -0.820094
N  -0.904335  -0.071782  -1.379440
H  -0.861379  -1.014972  -1.001423
H  -0.974880  -0.155378  -2.388309
H  -2.215236   1.530820  -1.507907
C  -1.808591   1.272743   0.529666
O  -2.486493   2.198730   0.920778
O  -0.893591   0.730718   1.333041
H  -0.381011  -0.022145   0.957334
H  -4.189598   0.520357  -0.475122
```



```
H  -3.610509  -0.467547  -1.828115
S  -3.322581  -1.650347   0.210533
H  -3.241549  -1.036474   1.408890
28
Dimer 31 of the n...n  type
C  -1.801341  -0.105094  -0.738074
C  -2.454817   1.200429  -0.273037
N  -2.591493  -0.945579  -1.640676
H  -3.582841  -0.884290  -1.421874
H  -2.463717  -0.676501  -2.608591
H  -0.868130   0.148960  -1.248639
C  -1.340297  -0.954314   0.457434
O  -0.886657  -0.481839   1.477373
O  -1.462157  -2.265840   0.269895
H  -1.884513  -2.347830  -0.622867
H  -1.766044   1.766164   0.352843
H  -2.712876   1.804163  -1.143199
S  -4.034605   0.960170   0.650202
H  -3.478478   0.391169   1.739320
C   2.195437  -0.106052   0.862547
C   1.632406   1.276923   0.521295
N   3.573870  -0.143905   1.357777
H   4.145382   0.555471   0.889683
H   3.616293   0.030603   2.354541
H   1.540582  -0.533713   1.629058
C   2.073169  -1.078955  -0.320581
O   1.162354  -1.061139  -1.118892
O   3.053704  -1.979727  -0.378329
H   3.675211  -1.714630   0.346983
H   0.577236   1.202142   0.277214
H   1.735350   1.925827   1.391187
S   2.517565   2.137217  -0.849209
H   2.097237   1.311389  -1.829129
28
Dimer 32 of the n...n  type
C  -1.626377  -0.353966  -0.611017
C  -2.091410   1.079447  -0.340009
N  -2.385746  -1.110715  -1.609101
H  -3.373643  -0.871407  -1.571901
H  -2.051767  -0.927417  -2.547577
H  -0.587551  -0.313829  -0.946059
C  -1.544609  -1.177467   0.683280
O  -1.224507  -0.712458   1.755281
O  -1.833098  -2.467302   0.519578
H  -2.103269  -2.539978  -0.431104
H  -1.427169   1.568186   0.369224
H  -2.072416   1.645043  -1.271437
S  -3.825054   1.196177   0.282272
H  -3.582758   0.630881   1.482665
C   2.698467   0.054412   0.816629
C   1.561165   1.081631   0.827648
N   4.052437   0.575991   0.615025
H   4.040564   1.379530  -0.008532
H   4.478432   0.860606   1.488701
H   2.674745  -0.462681   1.782576
C   2.438073  -1.058276  -0.215043
O   1.337451  -1.499230  -0.463685
O   3.542676  -1.519198  -0.797716
H   4.268309  -0.945179  -0.439258
H   0.616638   0.588422   1.046512
H   1.752371   1.819786   1.607020
S   1.413396   2.060760  -0.728557
H   1.095165   1.030929  -1.538330
28
Dimer 33 of the n...n  type
C   1.703060   0.526640   0.078905
C   1.096564  -0.835401  -0.270503
N   1.999299   1.420225  -1.042084
H   2.346362   0.890528  -1.838414
```

```
H   1.156604   1.903617  -1.330986
H   0.982289   1.038450   0.726581
C   2.962268   0.376431   0.944309
O   3.118520  -0.501466   1.763713
O   3.870960   1.331775   0.736541
H   3.500583   1.863260  -0.013949
H   0.895781  -1.400038   0.638181
H   0.160464  -0.683959  -0.805499
S   2.142246  -1.865625  -1.386258
H   3.123231  -2.099996  -0.490564
C  -3.121046   0.748349  -0.180830
C  -3.498216  -0.299610   0.872092
N  -3.161777   0.314316  -1.579559
H  -2.876728  -0.658238  -1.666991
H  -4.089691   0.408193  -1.974456
H  -3.814363   1.589938  -0.069412
C  -1.735552   1.351922   0.102150
O  -1.286520   1.532800   1.211159
O  -1.060069   1.676032  -1.002587
H  -1.633221   1.355011  -1.747604
H  -3.503860   0.147751   1.864789
H  -4.494999  -0.683265   0.654428
S  -2.384265  -1.771748   0.887777
H  -1.328402  -1.138182   1.433735
28
Dimer 34 of the n...n  type
C   2.921826   0.518305   0.680027
C   3.152887  -0.997919   0.666631
N   1.960441   1.018802   1.666779
H   1.261866   0.311547   1.876257
H   2.415304   1.273130   2.535079
H   3.890019   0.992984   0.872825
C   2.522294   1.022875  -0.718825
O   2.999288   0.597693  -1.745580
O   1.607669   1.994652  -0.702277
H   1.357638   2.092375   0.252225
H   3.890238  -1.255223  -0.091940
H   3.523075  -1.316090   1.641249
S   1.621630  -1.987631   0.378179
H   1.396479  -1.560918  -0.881320
C  -1.584547  -0.009333   0.580657
C  -3.001897  -0.334168   1.066582
N  -1.210665   1.403981   0.553726
H  -2.001647   1.981993   0.281141
H  -0.888385   1.720811   1.460220
H  -0.886431  -0.534570   1.241629
C  -1.313981  -0.637474  -0.795743
O  -1.747090  -1.716856  -1.130900
O  -0.517884   0.081441  -1.588244
H  -0.323994   0.916889  -1.100182
H  -3.152054  -1.412228   1.096166
H  -3.138084   0.068414   2.070398
S  -4.332423   0.429647   0.041273
H  -4.135133  -0.340020  -1.048494
28
Dimer 35 of the n...n  type
C  -3.137046  -0.010089  -0.026388
C  -2.709890   0.647826  -1.344085
N  -3.247034   0.867420   1.141444
H  -2.506954   1.565688   1.136306
H  -4.136839   1.350913   1.162984
H  -4.119773  -0.466706  -0.193691
C  -2.225191  -1.191027   0.338870
O  -1.667280  -1.893757  -0.475313
O  -2.104689  -1.392941   1.651038
H  -2.578979  -0.628281   2.065238
H  -2.695855  -0.089426  -2.145445
H  -3.426915   1.428103  -1.599833
S  -1.066601   1.481454  -1.267174
```



```
H  -0.314726   0.363265  -1.340603
C   2.197407  -0.421148   1.102535
C   2.814581  -1.322090   0.025891
N   0.751833  -0.562306   1.281209
H   0.321605  -1.013937   0.481281
H   0.504462  -1.096097   2.104369
H   2.705481  -0.645153   2.046214
C   2.529761   1.057425   0.816054
O   3.635548   1.441394   0.502676
O   1.493189   1.878971   0.951073
H   0.727887   1.277086   1.158532
H   3.888907  -1.156039  -0.032064
H   2.630702  -2.366753   0.277128
S   2.080293  -1.084331  -1.653478
H   2.576454   0.152865  -1.861605
28
Dimer 36 of the n...n  type
C  -3.184756   0.421974   0.562816
C  -2.794173  -1.050821   0.756892
N  -3.987402   0.727923  -0.623756
H  -3.845905   0.033531  -1.352670
H  -4.976005   0.774915  -0.411860
H  -3.737913   0.732389   1.454978
C  -1.922953   1.302525   0.551246
O  -1.006944   1.174142   1.333113
O  -1.908522   2.219015  -0.415206
H  -2.765465   2.072824  -0.900295
H  -2.164737  -1.152700   1.639978
H  -3.693116  -1.651780   0.893513
S  -1.935086  -1.797196  -0.696456
H  -0.855516  -0.992291  -0.674962
C   1.801084   0.634589   0.231627
C   1.510479  -0.703696   0.921137
N   1.141284   0.882117  -1.050175
H   1.035272   0.032517  -1.596970
H   0.243872   1.336650  -0.942417
H   1.478910   1.419207   0.923067
C   3.316514   0.846590   0.058840
O   4.143495   0.481752   0.864638
O   3.643513   1.499458  -1.057012
H   2.780095   1.618605  -1.530869
H   2.073343  -0.767578   1.850957
H   0.450096  -0.765681   1.157625
S   1.893797  -2.181543  -0.113511
H   3.231027  -2.002955  -0.132701
28
Dimer 37 of the n...n  type
C   3.091427   0.187923  -0.684764
C   2.008282  -0.678518  -1.338916
N   4.239124  -0.523991  -0.116958
H   3.954322  -1.423295   0.263642
H   4.959107  -0.687692  -0.810141
H   3.465319   0.875897  -1.451431
C   2.495724   1.109258   0.395397
O   1.410944   1.638712   0.301550
O   3.299228   1.300943   1.440472
H   4.081338   0.718038   1.259605
H   1.229642  -0.048289  -1.766026
H   2.456096  -1.265368  -2.140921
S   1.244902  -1.908362  -0.197162
H   0.601126  -1.024385   0.594616
C  -2.215213  -0.628032  -0.530652
C  -1.526247   0.674063  -0.951720
N  -3.578447  -0.835285  -1.026002
H  -4.071672   0.050106  -1.111230
H  -3.583843  -1.289452  -1.931095
H  -1.589622  -1.453414  -0.888103
C  -2.215612  -0.785679   0.999584
O  -1.289062  -0.454589   1.708114
```

```
O  -3.320055  -1.348877   1.483267
H  -3.900333  -1.470884   0.686901
H  -0.515127   0.723289  -0.557076
H  -1.485718   0.722919  -2.040116
S  -2.415900   2.198794  -0.415654
H  -2.128016   2.076965   0.896368
28
Dimer 38 of the n...n  type
C   1.296643  -0.239050   0.567975
C   2.081852   1.077217   0.590852
N   1.763596  -1.293692   1.468062
H   2.771400  -1.244536   1.594611
H   1.326175  -1.230210   2.379127
H   0.266765   0.007441   0.832996
C   1.208528  -0.800317  -0.862007
O   1.019821  -0.102817  -1.836170
O   1.346252  -2.118305  -0.940035
H   1.470906  -2.407784   0.000327
H   1.623151   1.798951  -0.081928
H   2.069310   1.484957   1.601753
S   3.864062   0.894793   0.149839
H   3.663355   0.596521  -1.150287
C  -2.933915  -0.039884   0.496528
C  -2.980427   1.283055  -0.276720
N  -2.423627   0.026105   1.869811
H  -1.755267   0.785016   1.976534
H  -3.168280   0.168684   2.541088
H  -3.955667  -0.433584   0.531423
C  -2.141079  -1.108749  -0.279216
O  -2.174042  -1.228043  -1.481639
O  -1.419719  -1.914448   0.503812
H  -1.565392  -1.560910   1.419313
H  -3.376379   1.116064  -1.277095
H  -3.630544   1.983811   0.247081
S  -1.346971   2.132192  -0.408549
H  -0.763339   1.249118  -1.246603
28
Dimer 39 of the n...n  type
C   1.222008   0.509841  -0.138206
C   1.852202  -0.479960  -1.118908
N   1.511988   1.928758  -0.343602
H   2.439605   2.064282  -0.736277
H   0.830738   2.359599  -0.957478
H   0.139987   0.391260  -0.224623
C   1.531316   0.141419   1.321509
O   1.619372  -0.997915   1.724084
O   1.649854   1.198401   2.122525
H   1.590433   1.976148   1.507070
H   1.504101  -1.486788  -0.897404
H   1.550226  -0.219238  -2.133631
S   3.696789  -0.452550  -1.125806
H   3.852321  -0.970755   0.109694
C  -2.610954  -0.547453   0.681947
C  -3.315354   0.583364  -0.076254
N  -1.676986  -0.136967   1.733173
H  -1.296672   0.787301   1.552306
H  -2.124587  -0.110310   2.640907
H  -3.392093  -1.163806   1.141119
C  -1.901747  -1.507688  -0.291729
O  -2.338061  -1.789771  -1.384923
O  -0.767399  -2.028627   0.175801
H  -0.602291  -1.595247   1.047923
H  -4.039036   0.172639  -0.777932
H  -3.837767   1.222775   0.635259
S  -2.171094   1.700440  -1.001884
H  -1.846055   0.809503  -1.960945
28
Dimer 40 of the n...n  type
C  -3.099750  -0.507833   0.619164
```



```
C  -1.773134  -1.251792   0.810424
N  -4.059322  -1.098809  -0.315862
H  -3.579162  -1.585815  -1.068662
H  -4.676788  -1.756180   0.144394
H  -3.577695  -0.444356   1.603096
C  -2.856619   0.959899   0.224455
O  -1.962811   1.637180   0.683739
O  -3.742297   1.437192  -0.645723
H  -4.320699   0.658526  -0.858238
H  -1.141622  -0.696585   1.502070
H  -1.968503  -2.241714   1.222659
S  -0.837810  -1.556940  -0.749736
H  -0.537205  -0.271310  -1.018970
C   2.423612   0.867542   0.836832
C   3.662985  -0.015946   0.643454
N   1.300006   0.275184   1.559537
H   1.166495  -0.689580   1.264437
H   1.465345   0.276102   2.559116
H   2.745412   1.755760   1.394136
C   1.921569   1.429797  -0.502076
O   2.634093   1.615295  -1.462547
O   0.623359   1.741346  -0.523202
H   0.231301   1.462689   0.336594
H   4.444283   0.539952   0.127016
H   4.037837  -0.323337   1.620095
S   3.332651  -1.583882  -0.269030
H   3.018036  -1.003730  -1.444863
28
Dimer 41 of the n...n  type
C  -3.099513   0.508501  -0.618977
C  -1.772541   1.251828  -0.810368
N  -4.058598   1.099723   0.316375
H  -3.577970   1.585768   1.069507
H  -4.675322   1.758070  -0.143481
H  -3.577739   0.445491  -1.602804
C  -2.857063  -0.959460  -0.224694
O  -1.963431  -1.636975  -0.683911
O  -3.743223  -1.436703   0.645100
H  -4.321313  -0.657905   0.857795
H  -1.141519   0.696428  -1.502326
H  -1.967504   2.241919  -1.222395
S  -0.836668   1.556047   0.749582
H  -0.537769   0.270029   1.018827
C   2.423966  -0.868072  -0.836325
C   3.662998   0.015769  -0.642855
N   1.300484  -0.276408  -1.559908
H   1.166689   0.688524  -1.265458
H   1.466276  -0.277912  -2.559416
H   2.746154  -1.756549  -1.392989
C   1.921328  -1.429804   0.502620
O   2.633271  -1.614538   1.463631
O   0.623178  -1.741866   0.522956
H   0.231742  -1.463189  -0.337163
H   4.444099  -0.539560  -0.125512
H   4.038455   0.322568  -1.619449
S   3.331831   1.584340   0.268331
H   3.016500   1.004993   1.444361
28
Dimer 42 of the n...n  type
C  -1.751732  -0.064256   0.814441
C  -1.842751  -1.349135  -0.016275
N  -2.777526   0.123259   1.842083
H  -3.673311  -0.247328   1.534186
H  -2.525709  -0.336663   2.708577
H  -0.775550  -0.049978   1.308928
C  -1.713836   1.182764  -0.082934
O  -1.176948   1.212815  -1.168961
O  -2.299276   2.250420   0.454956
H  -2.691112   1.916243   1.301975
```

```
H  -1.012413  -1.412119  -0.716898
H  -1.800421  -2.211455   0.649204
S  -3.419997  -1.520389  -0.956911
H  -3.177925  -0.513485  -1.821057
C   1.751813  -0.064290  -0.814586
C   1.842586  -1.349226   0.015987
N   2.777936   0.123271  -1.841926
H   3.673476  -0.247929  -1.534076
H   2.526033  -0.335932  -2.708769
H   0.775791  -0.049900  -1.309395
C   1.713919   1.182704   0.082907
O   1.176626   1.212798   1.168742
O   2.299922   2.250148  -0.454684
H   2.691955   1.915805  -1.301627
H   1.012087  -1.412341   0.716405
H   1.800457  -2.211508  -0.649554
S   3.419611  -1.520620   0.957045
H   3.177366  -0.513770   1.821206
28
Dimer 43 of the n...n  type
C  -1.364356   0.287470   0.023859
C  -2.394386   0.660984   1.092251
N  -1.337406   1.109545  -1.184676
H  -2.264058   1.457244  -1.413881
H  -0.697221   1.888144  -1.085272
H  -0.376196   0.347251   0.493049
C  -1.479950  -1.189429  -0.387707
O  -1.804408  -2.081262   0.363037
O  -1.128599  -1.406534  -1.656557
H  -0.994906  -0.490663  -2.024329
H  -2.287240   0.010816   1.959281
H  -2.231927   1.692941   1.403639
S  -4.141143   0.590639   0.500994
H  -4.187522  -0.748882   0.350964
C   3.054282   0.645436   0.363605
C   3.296191  -0.858586   0.202288
N   2.604160   1.095751   1.684184
H   1.994238   0.406803   2.117803
H   3.383728   1.262272   2.309106
H   3.997341   1.157038   0.139552
C   2.070715   1.174463  -0.694019
O   1.985964   0.744602  -1.819835
O   1.313955   2.187082  -0.254279
H   1.549005   2.273715   0.706337
H   3.698801  -1.073923  -0.786129
H   4.015330  -1.187096   0.952465
S   1.783580  -1.883440   0.464819
H   1.224082  -1.621034  -0.732736
28
Dimer 44 of the n...n  type
C   1.921135   0.659711   0.539234
C   1.341987  -0.665784   1.048228
N   1.261399   1.267699  -0.617234
H   0.910707   0.558368  -1.255339
H   0.475187   1.849491  -0.354382
H   1.873741   1.372087   1.370963
C   3.424410   0.523999   0.231682
O   4.184077  -0.164159   0.876354
O   3.824431   1.259558  -0.805257
H   2.985221   1.663409  -1.151870
H   1.901531  -1.006585   1.918084
H   0.302970  -0.518836   1.339221
S   1.317331  -2.014373  -0.210358
H   2.651996  -2.204305  -0.251573
C  -2.954856  -0.347972  -0.589807
C  -3.477190   0.329705   0.681032
N  -2.400987  -1.695756  -0.440852
H  -1.876207  -1.780048   0.426296
H  -3.129272  -2.399774  -0.434467
```



```
H  -3.794652  -0.412414  -1.291874
C  -1.919822   0.523940  -1.318749
O  -1.885520   1.732629  -1.267728
O  -1.046547  -0.180439  -2.042445
H  -1.220363  -1.126177  -1.806135
H  -3.925179   1.292041   0.437840
H  -4.237273  -0.304666   1.137026
S  -2.193785   0.584024   1.981738
H  -1.543532   1.577082   1.343626
28
Dimer 45 of the n...n  type
C  -2.361664  -1.125995   0.505985
C  -3.240706  -0.484633  -0.574661
N  -2.285592  -0.462464   1.807828
H  -2.182895   0.542415   1.686307
H  -3.116036  -0.635153   2.361619
H  -2.758009  -2.134844   0.676212
C  -0.929383  -1.381544   0.012282
O  -0.625157  -1.547128  -1.147232
O  -0.033289  -1.455421   1.000216
H  -0.534905  -1.182369   1.808461
H  -3.154019  -1.057865  -1.497162
H  -4.280929  -0.507112  -0.248046
S  -2.887728   1.289887  -0.918196
H  -1.580944   1.156483  -1.230926
C   2.361938   1.126040   0.505479
C   3.240506   0.484045  -0.575190
N   2.285863   0.462886   1.807546
H   2.183619  -0.542077   1.686432
H   3.115994   0.636256   2.361577
H   2.758695   2.134758   0.675410
C   0.929599   1.381914   0.011959
O   0.625249   1.547604  -1.147518
O   0.033736   1.456042   1.000067
H   0.535551   1.182735   1.808177
H   3.153600   1.056927  -1.497890
H   4.280866   0.506486  -0.249004
S   2.887173  -1.290529  -0.917911
H   1.580354  -1.157070  -1.230475
28
Dimer 46 of the n...n  type
C  -1.589855   0.133377   0.321583
C  -2.542470  -0.833347   1.029295
N  -1.691426   1.545759   0.692680
H  -2.648398   1.794247   0.929252
H  -1.095811   1.776381   1.477992
H  -0.573819  -0.213394   0.532062
C  -1.698584   0.015407  -1.207228
O  -1.935013  -1.017352  -1.791927
O  -1.460179   1.163882  -1.844985
H  -1.375094   1.832542  -1.115361
H  -2.342814  -1.854931   0.709669
H  -2.389649  -0.765749   2.106542
S  -4.329464  -0.457895   0.757689
H  -4.344293  -0.777250  -0.552582
C   3.004364  -0.302262   0.907131
C   2.092551   0.911105   1.126175
N   4.335477  -0.028363   0.363010
H   4.304077   0.742907  -0.299491
H   4.996865   0.208278   1.092616
H   3.131117  -0.793626   1.878546
C   2.319997  -1.364797   0.028397
O   1.130494  -1.593967   0.056639
O   3.159605  -2.035973  -0.755663
H   4.038188  -1.600045  -0.609580
H   1.161629   0.598139   1.597530
H   2.589797   1.618585   1.789782
S   1.725003   1.860138  -0.409913
H   1.035963   0.886639  -1.035838
```

```
28
Dimer 47 of the n...n  type
C   2.909954   0.040607  -0.442694
C   2.538842  -1.445853  -0.511003
N   3.357262   0.546834   0.856763
H   2.854886   0.087272   1.612716
H   4.348953   0.396497   0.997217
H   3.716598   0.208537  -1.165552
C   1.759606   0.930100  -0.945052
O   1.003866   0.615054  -1.837237
O   1.681242   2.105778  -0.320678
H   2.374379   2.052234   0.387945
H   2.231359  -1.706767  -1.522729
H   3.408226  -2.045893  -0.241983
S   1.210707  -1.937517   0.669730
H   0.219713  -1.258477   0.060379
C  -2.433192   1.049298   0.271750
C  -3.293471  -0.040648  -0.379287
N  -1.599219   1.853767  -0.615208
H  -1.098333   1.283925  -1.293915
H  -2.140874   2.550934  -1.110944
H  -3.119788   1.732404   0.786765
C  -1.549710   0.483484   1.396026
O  -1.862747  -0.457386   2.090878
O  -0.400102   1.138861   1.561247
H  -0.365415   1.807109   0.831986
H  -3.956712  -0.493285   0.356469
H  -3.900497   0.410975  -1.163994
S  -2.324108  -1.380968  -1.197876
H  -2.034625  -2.054297  -0.067040
28
Dimer 48 of the n...n  type
C   1.286127   0.192039  -0.018790
C   2.312354   0.937881  -0.876724
N   1.023640   0.738203   1.312231
H   1.891421   1.022463   1.760930
H   0.424004   1.554296   1.243401
H   0.338119   0.205227  -0.567055
C   1.622284  -1.300687   0.100631
O   2.186658  -1.935383  -0.758840
O   1.195220  -1.864071   1.237172
H   0.841136  -1.122217   1.781645
H   2.378431   0.484842  -1.864846
H   1.997021   1.975809  -0.986753
S   3.998180   1.004297  -0.130544
H   4.272018  -0.307213  -0.283982
C  -2.831597  -0.448243   0.451464
C  -3.266321   1.008590   0.255345
N  -1.947616  -0.726550   1.584718
H  -1.292682   0.034516   1.744068
H  -2.471328  -0.874196   2.438863
H  -3.743673  -1.040828   0.586606
C  -2.194660  -1.018456  -0.827797
O  -2.525798  -0.697376  -1.946012
O  -1.241220  -1.923403  -0.596397
H  -1.148720  -1.953144   0.389035
H  -3.994304   1.078183  -0.551394
H  -3.725550   1.372649   1.174322
S  -1.882171   2.176224  -0.105631
H  -1.642186   1.734830  -1.356840
28
Dimer 49 of the n...n  type
C  -3.001218   0.538587   0.265779
C  -3.456784  -0.833681  -0.241363
N  -2.635595   1.528218  -0.750923
H  -2.179110   1.087109  -1.545480
H  -3.446412   2.028193  -1.095145
H  -3.823278   0.957906   0.857296
C  -1.836979   0.417536   1.263116
```



```
O  -1.670184  -0.528301   2.001331
O  -1.015331   1.467269   1.254770
H  -1.340166   2.034392   0.509166
H  -3.775910  -1.457248   0.592288
H  -4.297743  -0.702164  -0.922216
S  -2.168830  -1.742255  -1.202356
H  -1.418817  -2.084953  -0.136713
C   1.739336  -0.705190   0.119920
C   1.099915   0.182447  -0.953542
N   1.448876  -0.359419   1.513799
H   1.314524   0.644174   1.608043
H   0.592834  -0.796551   1.836587
H   1.392640  -1.729871  -0.056784
C   3.266490  -0.778627  -0.062831
O   3.815097  -0.804512  -1.142216
O   3.935116  -0.835097   1.087851
H   3.225938  -0.742889   1.779925
H   1.351343  -0.188537  -1.945725
H   0.018682   0.156411  -0.846521
S   1.573140   1.960349  -0.839243
H   2.841391   1.801622  -1.269634
28
Dimer 50 of the n...n  type
C  -3.112534   0.623264  -0.227493
C  -3.321404  -0.879207  -0.012441
N  -2.894628   1.057886  -1.610088
H  -2.341214   0.374026  -2.120904
H  -3.769715   1.187236  -2.103473
H  -4.004378   1.138397   0.147627
C  -1.962519   1.163140   0.639977
O  -1.680042   0.737274   1.735506
O  -1.300564   2.176102   0.070617
H  -1.698568   2.254662  -0.834836
H  -3.560731  -1.083703   1.029910
H  -4.150684  -1.216263  -0.634096
S  -1.861743  -1.902721  -0.494915
H  -1.157515  -1.665075   0.627549
C   1.443594   0.366552  -0.140958
C   2.560933   0.483773  -1.181103
N   1.437145   1.352917   0.936229
H   2.381199   1.631731   1.185848
H   0.890542   2.168259   0.689992
H   0.491258   0.452479  -0.676895
C   1.388219  -1.039086   0.477063
O   1.706515  -2.052542  -0.103361
O   0.887956  -1.050216   1.714044
H   0.768829  -0.090797   1.942443
H   2.434983  -0.272127  -1.954942
H   2.520755   1.470556  -1.642517
S   4.258627   0.336774  -0.471822
H   4.158828  -0.965403  -0.135634
28
Dimer 51 of the n...n  type
C  -2.290704   0.139181  -1.098366
C  -2.718082  -1.243921  -0.592759
N  -0.923766   0.248766  -1.609741
H  -0.291859  -0.389807  -1.130143
H  -0.875022   0.041071  -2.599654
H  -2.980103   0.416709  -1.903861
C  -2.505460   1.216296  -0.019217
O  -3.444256   1.221752   0.745524
O  -1.567962   2.163005  -0.013336
H  -0.916945   1.876064  -0.706438
H  -3.777223  -1.243095  -0.340226
H  -2.544702  -1.980904  -1.376913
S  -1.768565  -1.840844   0.874425
H  -2.336834  -0.999031   1.761576
C   2.378103   0.227678   1.062806
C   2.907712  -1.093596   0.494594
```

```
N   1.079820   0.177361   1.736475
H   0.447149  -0.485400   1.290915
H   1.173088  -0.089565   2.709054
H   3.118017   0.587591   1.787288
C   2.333431   1.328440  -0.011979
O   3.133080   1.421338  -0.915524
O   1.339289   2.200978   0.159989
H   0.824872   1.845022   0.930597
H   3.922212  -0.965575   0.120426
H   2.918490  -1.843215   1.285782
S   1.871618  -1.804225  -0.857643
H   2.266419  -0.932700  -1.807742
28
Dimer 52 of the n...n  type
C   2.904685  -0.258336   0.369853
C   2.723438  -1.227701  -0.804175
N   2.376984  -0.692706   1.666994
H   1.565509  -1.295876   1.550895
H   3.070608  -1.196800   2.205606
H   3.979947  -0.081028   0.482201
C   2.327560   1.131655   0.036281
O   2.403348   1.647546  -1.053502
O   1.754436   1.734776   1.082077
H   1.842556   1.076284   1.820204
H   3.127532  -0.786376  -1.713972
H   3.257758  -2.154431  -0.594028
S   0.972624  -1.730954  -1.091128
H   0.499481  -0.506262  -1.409029
C  -2.255897   0.235337   1.071226
C  -2.322038   1.582190   0.340815
N  -0.934650  -0.145440   1.576373
H  -0.212011   0.244851   0.980525
H  -0.781529   0.196925   2.517148
H  -2.949325   0.285221   1.917678
C  -2.804903  -0.897567   0.185011
O  -3.775880  -0.773303  -0.527577
O  -2.129499  -2.041161   0.282435
H  -1.365138  -1.836648   0.878625
H  -3.346607   1.804504   0.047462
H  -1.965175   2.369410   1.004941
S  -1.240424   1.666276  -1.154821
H  -1.991455   0.845765  -1.918172
28
Dimer 53 of the n...n  type
C  -1.645120   0.178342   0.606846
C  -2.090339  -1.259033   0.321699
N  -2.327921   0.872158   1.701355
H  -3.293351   0.562864   1.782231
H  -1.868258   0.712605   2.589441
H  -0.578411   0.126580   0.837440
C  -1.724233   1.043690  -0.660064
O  -1.421234   0.642099  -1.762920
O  -2.123730   2.293790  -0.437332
H  -2.315788   2.318175   0.536193
H  -1.476499  -1.684337  -0.468158
H  -1.960105  -1.859180   1.222128
S  -3.874410  -1.413631  -0.126946
H  -3.778370  -0.795654  -1.321978
C   3.041579  -0.380963   0.734279
C   2.246955   0.892568   1.044548
N   4.296365  -0.205280   0.000095
H   4.224518   0.562827  -0.662787
H   5.071239  -0.012996   0.623293
H   3.274690  -0.860606   1.691646
C   2.172960  -1.413519  -0.007729
O   0.991025  -1.570993   0.205319
O   2.845461  -2.143195  -0.894945
H   3.759863  -1.759013  -0.888582
H   1.373021   0.650757   1.648098
```



```
H   2.875875   1.578463   1.612251
S   1.726125   1.836894  -0.450879
H   0.942033   0.879048  -0.984615
28
Dimer 54 of the n...n  type
C   2.783799   0.480028   0.387381
C   3.154035  -0.446680  -0.778140
N   2.541913  -0.118608   1.697701
H   1.914582  -0.914418   1.617725
H   3.405946  -0.426215   2.127948
H   3.626273   1.174760   0.501580
C   1.611435   1.412504   0.044447
O   1.310147   1.740460  -1.080440
O   0.990788   1.906427   1.119051
H   1.333965   1.374995   1.876687
H   3.265507   0.145314  -1.686070
H   4.106673  -0.930747  -0.559716
S   1.985227  -1.833894  -1.096852
H   0.871995  -1.083135  -1.184447
C  -2.177895  -0.878847  -0.553355
C  -1.814014   0.446546  -1.233187
N  -3.606394  -1.123684  -0.336883
H  -4.089613  -0.257889  -0.109702
H  -4.045893  -1.524539  -1.156684
H  -1.790948  -1.689474  -1.182307
C  -1.442729  -1.049669   0.785610
O  -0.313080  -0.663157   0.981710
O  -2.148600  -1.695854   1.712329
H  -3.033633  -1.843905   1.290933
H  -0.744650   0.511618  -1.422944
H  -2.341792   0.510449  -2.184854
S  -2.307914   1.938073  -0.266916
H  -1.265874   1.896074   0.588701
28
Dimer 55 of the n...n  type
C   2.917466   0.881493  -0.473552
C   3.483211  -0.529570  -0.264515
N   2.984799   1.795047   0.669655
H   2.908488   1.280818   1.544229
H   3.853519   2.315242   0.683877
H   3.475645   1.331970  -1.302134
C   1.468008   0.833741  -0.989513
O   1.064069   0.016552  -1.786512
O   0.695460   1.809995  -0.514304
H   1.274810   2.287917   0.134261
H   3.313633  -1.126602  -1.159500
H   4.556272  -0.464782  -0.083064
S   2.799094  -1.412725   1.202679
H   1.501928  -1.412266   0.820586
C  -2.029744   0.116817  -0.804493
C  -2.439208   1.173152   0.223835
N  -3.092218  -0.446722  -1.640842
H  -3.947636  -0.571521  -1.104746
H  -3.300325   0.154326  -2.429155
H  -1.289626   0.576301  -1.465542
C  -1.253796  -1.033983  -0.152391
O  -0.555601  -0.906717   0.830407
O  -1.387243  -2.199758  -0.783059
H  -2.061970  -2.025417  -1.486228
H  -1.554212   1.551732   0.730696
H  -2.932894   2.000350  -0.286795
S  -3.652475   0.570081   1.477694
H  -2.800680  -0.264508   2.107375
28
Dimer 56 of the n...n  type
C  -2.848339  -0.500460   0.865123
C  -3.651234   0.468947  -0.011764
N  -2.051530   0.066181   1.952125
H  -1.535367   0.884463   1.635360
```

```
H  -2.629840   0.330179   2.740594
H  -3.570987  -1.193254   1.313435
C  -1.940603  -1.414244   0.022839
O  -2.189213  -1.769989  -1.105212
O  -0.851328  -1.825829   0.679306
H  -0.874411  -1.327049   1.535906
H  -4.161942  -0.088621  -0.796240
H  -4.398920   0.972864   0.601398
S  -2.658796   1.823571  -0.768554
H  -1.766396   1.040007  -1.406205
C   2.098193  -0.843395  -0.144104
C   3.213473  -0.492003   0.845137
N   2.510054  -1.167472  -1.511944
H   3.312272  -0.608876  -1.792464
H   2.756549  -2.145233  -1.606191
H   1.558326  -1.706762   0.257066
C   1.028225   0.258284  -0.197198
O   0.687324   0.913997   0.761713
O   0.472367   0.404229  -1.400110
H   0.972237  -0.225257  -1.981393
H   2.795051  -0.322168   1.836135
H   3.921067  -1.319438   0.899071
S   4.210982   0.982053   0.354958
H   3.226223   1.886144   0.534016
28
Dimer 57 of the n...n  type
C  -3.134731   0.159019  -0.569502
C  -3.419406  -0.955682   0.441938
N  -2.628415  -0.245366  -1.882396
H  -1.968098  -1.012945  -1.801953
H  -3.379113  -0.544326  -2.493332
H  -4.081725   0.687101  -0.735109
C  -2.206400   1.239998   0.005190
O  -2.128671   1.517153   1.178855
O  -1.508557   1.892902  -0.931358
H  -1.670827   1.382379  -1.763934
H  -3.888102  -0.541988   1.333651
H  -4.101605  -1.677905  -0.006638
S  -1.937541  -1.925185   0.961716
H  -1.412597  -0.958890   1.741562
C   1.545718  -0.248678   0.435341
C   2.731444  -1.221478   0.480492
N   0.674673  -0.334227  -0.732064
H   1.224453  -0.436196  -1.581446
H   0.061860  -1.138440  -0.650947
H   0.930052  -0.455487   1.319282
C   2.007610   1.203044   0.635627
O   2.987144   1.506286   1.280763
O   1.227880   2.125515   0.072672
H   0.507491   1.663175  -0.412366
H   3.273140  -1.117330   1.419452
H   2.356533  -2.242231   0.401456
S   3.916115  -1.019654  -0.919599
H   4.399766   0.175469  -0.524304
28
Dimer 58 of the n...n  type
C   1.221681   0.034866  -0.539585
C   1.350166  -0.853293   0.700567
N   1.472290  -0.596408  -1.835122
H   2.181178  -1.321126  -1.759632
H   0.626345  -1.009362  -2.210237
H   0.197622   0.415806  -0.555203
C   2.093136   1.296953  -0.419514
O   2.299258   1.876810   0.622388
O   2.582489   1.718976  -1.586393
H   2.301354   1.022618  -2.234671
H   1.069417  -0.291065   1.587781
H   0.681147  -1.707489   0.605438
S   3.032286  -1.572847   0.931073
```



```
H   3.645647  -0.409214   1.228811
C  -2.819515   0.726652   0.296402
C  -3.333714  -0.521547  -0.425970
N  -2.242544   1.778817  -0.545191
H  -1.782506   1.387604  -1.362970
H  -2.950024   2.426768  -0.870078
H  -3.668344   1.160686   0.837130
C  -1.812352   0.367223   1.403719
O  -1.846920  -0.660172   2.041240
O  -0.892195   1.309110   1.618277
H  -1.051828   1.987258   0.915373
H  -3.827235  -1.189274   0.278201
H  -4.052089  -0.224465  -1.189785
S  -2.022501  -1.475963  -1.310110
H  -1.472923  -2.007561  -0.201188
28
Dimer 59 of the n...n  type
C   2.522749  -1.265346   0.130265
C   3.255247  -0.175063   0.922946
N   2.871112  -1.396081  -1.286400
H   3.141674  -0.496089  -1.675825
H   3.635093  -2.045436  -1.427743
H   2.733054  -2.220958   0.623487
C   0.996881  -1.108117   0.253646
O   0.439700  -0.771161   1.275311
O   0.332879  -1.407784  -0.859787
H   1.045015  -1.603702  -1.524123
H   2.880361  -0.151868   1.945197
H   4.322174  -0.397530   0.943654
S   3.123429   1.509608   0.183250
H   1.777813   1.602665   0.226632
C  -2.563242   0.916828  -0.704100
C  -3.495741   0.181470   0.268614
N  -2.106161   0.187171  -1.886362
H  -1.882482  -0.776619  -1.650884
H  -2.807947   0.186378  -2.616652
H  -3.108259   1.802293  -1.052380
C  -1.341846   1.515806   0.018072
O  -1.372300   1.967013   1.138934
O  -0.244574   1.560547  -0.741366
H  -0.490433   1.075547  -1.569776
H  -3.675815   0.811945   1.138667
H  -4.448724  -0.014548  -0.223697
S  -2.892770  -1.472483   0.818098
H  -1.656649  -1.086102   1.209827
28
Dimer 60 of the n...n  type
C   2.810986   0.234531   0.465050
C   3.076782  -0.776451  -0.658048
N   2.372868  -0.296388   1.752530
H   1.627213  -0.976492   1.631794
H   3.138445  -0.729208   2.254822
H   3.756062   0.766062   0.635130
C   1.843151   1.343383   0.025232
O   1.746326   1.736465  -1.116887
O   1.138340   1.878782   1.020965
H   1.321697   1.303323   1.802570
H   3.484940  -0.268561  -1.530901
H   3.808303  -1.505640  -0.308824
S   1.610686  -1.763210  -1.186245
H   1.023541  -0.778349  -1.892106
C  -2.008488  -0.774558  -0.561451
C  -1.545780   0.569386  -1.132032
N  -3.443110  -1.059229  -0.657615
H  -3.989483  -0.214786  -0.506002
H  -3.689117  -1.434099  -1.565944
H  -1.474459  -1.563567  -1.103730
C  -1.568195  -0.960986   0.898387
O  -0.534756  -0.525256   1.349990
```

```
O  -2.419498  -1.683377   1.628653
H  -3.188641  -1.845568   1.025855
H  -0.462787   0.643559  -1.118879
H  -1.886444   0.650918  -2.164333
S  -2.219047   2.037958  -0.243978
H  -1.337839   1.981017   0.775122
28
Dimer 61 of the n...n  type
C  -2.619067  -0.930070  -0.466048
C  -2.436396   0.303486  -1.361942
N  -3.650095  -0.839724   0.570134
H  -3.793406   0.125340   0.857202
H  -4.536482  -1.213542   0.254640
H  -2.859383  -1.772302  -1.123433
C  -1.283745  -1.328757   0.188237
O  -0.224549  -1.322298  -0.400090
O  -1.392623  -1.700250   1.460140
H  -2.357978  -1.582164   1.665709
H  -1.602333   0.139039  -2.043405
H  -3.339733   0.462085  -1.950975
S  -2.188089   1.883500  -0.439186
H  -1.093136   1.509786   0.268396
C   2.518008   0.745282  -0.531086
C   2.396905   1.364469   0.866855
N   1.444529   0.988499  -1.491667
H   0.542051   0.775987  -1.080131
H   1.435995   1.952275  -1.803755
H   3.437001   1.159608  -0.965857
C   2.801671  -0.764950  -0.479414
O   3.319113  -1.324548   0.460338
O   2.496281  -1.405692  -1.609130
H   1.986964  -0.755216  -2.145915
H   3.275593   1.099697   1.454252
H   2.354763   2.450288   0.773965
S   0.884926   0.911347   1.815787
H   0.989622  -0.424313   1.674743
28
Dimer 62 of the n...n  type
C  -2.669327   0.075374   0.729207
C  -3.034721   0.783545  -0.579680
N  -1.828536   0.824953   1.664936
H  -1.152779   1.401379   1.169030
H  -2.380148   1.425345   2.265719
H  -3.607520  -0.169794   1.240450
C  -2.014597  -1.292488   0.463742
O  -2.304256  -2.011888  -0.464352
O  -1.107921  -1.632731   1.382494
H  -1.042938  -0.833547   1.968007
H  -3.708367   0.162177  -1.167881
H  -3.534371   1.725221  -0.351934
S  -1.574995   1.228733  -1.616256
H  -1.252341  -0.038591  -1.942044
C   2.669284  -0.074161   0.729375
C   3.034796  -0.784380  -0.578354
N   1.828522  -0.822326   1.666282
H   1.152695  -1.399438   1.171270
H   2.380172  -1.421895   2.267855
H   3.607428   0.171895   1.240289
C   2.014463   1.293223   0.461760
O   2.303977   2.011129  -0.467534
O   1.107865   1.634926   1.380052
H   1.042953   0.836698   1.966851
H   3.708459  -0.163919  -1.167499
H   3.534509  -1.725649  -0.349069
S   1.575203  -1.231351  -1.614320
H   1.252305   0.035435  -1.941938
28
Dimer 63 of the n...n  type
C   2.658320   0.907971   0.296038
```



```
C   3.479933  -0.290912   0.784468
N   1.454024   1.221670   1.066146
H   1.029026   0.369366   1.419803
H   1.660053   1.817986   1.858516
H   3.314477   1.785313   0.321910
C   2.281132   0.755770  -1.188394
O   2.984376   0.210231  -2.006844
O   1.104491   1.300777  -1.511164
H   0.722072   1.642089  -0.665954
H   4.381351  -0.402084   0.184224
H   3.766122  -0.129799   1.823741
S   2.557167  -1.890564   0.764617
H   2.537239  -2.026286  -0.577237
C  -2.035835  -0.806580   0.575523
C  -2.005079   0.519645   1.345563
N  -0.882769  -1.120678  -0.266535
H  -0.537162  -0.296689  -0.750036
H  -0.117518  -1.529632   0.258043
H  -2.131447  -1.606642   1.318776
C  -3.314661  -0.922899  -0.275282
O  -4.383005  -0.455856   0.048896
O  -3.148023  -1.618007  -1.401086
H  -2.179414  -1.824358  -1.425550
H  -2.912098   0.622467   1.939342
H  -1.151036   0.534842   2.020946
S  -1.819691   2.012145   0.280801
H  -3.025637   1.916061  -0.316387
28
Dimer 64 of the n...n  type
C   1.596213  -0.229425  -0.044418
C   2.498483  -0.604055   1.133730
N   1.647323  -1.105512  -1.216553
H   2.596012  -1.432238  -1.383158
H   1.045965  -1.913174  -1.100853
H   0.566413  -0.215173   0.321550
C   1.821468   1.221592  -0.495842
O   2.146867   2.118304   0.248535
O   1.596445   1.416647  -1.797265
H   1.424425   0.510632  -2.157240
H   2.333772   0.079563   1.965314
H   2.261630  -1.617091   1.459774
S   4.297486  -0.623365   0.720846
H   4.413899   0.707818   0.539063
C  -2.993770  -0.100586   0.962784
C  -2.086226  -1.325203   0.799947
N  -4.358504  -0.222888   0.447147
H  -4.377825  -0.786726  -0.399223
H  -4.977849  -0.649939   1.125220
H  -3.057596   0.114846   2.035390
C  -2.347348   1.159551   0.358649
O  -1.154650   1.370571   0.365838
O  -3.223715   2.018384  -0.157334
H  -4.098483   1.558936  -0.078179
H  -1.128336  -1.150300   1.287611
H  -2.557740  -2.187602   1.271431
S  -1.806305  -1.823455  -0.954000
H  -1.177417  -0.694130  -1.337688
28
Dimer 65 of the n...n  type
C   3.208344  -0.304694  -0.844609
C   1.723644  -0.660597  -0.711639
N   4.166132  -1.351461  -0.477253
H   3.808724  -1.916564   0.289134
H   4.366267  -1.966468  -1.256566
H   3.386783  -0.036844  -1.892108
C   3.546493   0.975443  -0.057782
O   2.797351   1.920915   0.040637
O   4.762080   0.962811   0.490434
H   5.114925   0.058870   0.286029
```

```
H   1.102631   0.164264  -1.054001
H   1.505993  -1.539818  -1.317825
S   1.218255  -1.102838   1.006181
H   1.284821   0.149850   1.499688
C  -2.340788  -0.463305   0.571778
C  -3.698902  -0.982590   0.087672
N  -2.360058   0.418687   1.741388
H  -3.228457   0.944927   1.791732
H  -2.248770  -0.098938   2.604333
H  -1.720088  -1.331577   0.810072
C  -1.580531   0.236384  -0.567821
O  -1.538873  -0.184164  -1.702592
O  -0.938660   1.341343  -0.191715
H  -1.155003   1.430605   0.773388
H  -3.560835  -1.649598  -0.761999
H  -4.183453  -1.534422   0.893335
S  -4.887170   0.350215  -0.381044
H  -4.218162   0.766012  -1.476056
28
Dimer 66 of the n...n  type
C  -2.312119  -0.731052   0.196646
C  -3.377704  -0.687360  -0.902951
N  -2.782559  -1.010396   1.554967
H  -3.709692  -0.621112   1.706984
H  -2.821786  -2.005213   1.740046
H  -1.587131  -1.505070  -0.074797
C  -1.477513   0.560052   0.214413
O  -1.149212   1.158169  -0.785847
O  -1.112465   0.950511   1.435602
H  -1.548112   0.296365   2.041078
H  -2.908717  -0.518186  -1.871080
H  -3.907709  -1.639503  -0.930217
S  -4.678491   0.595287  -0.634905
H  -3.876175   1.660334  -0.837818
C   3.171253  -0.043905  -0.969486
C   1.751662  -0.620213  -1.004404
N   4.262905  -1.008030  -0.805466
H   3.979195  -1.777761  -0.204200
H   4.557479  -1.394159  -1.694189
H   3.330798   0.487020  -1.914961
C   3.305631   1.046492   0.109209
O   2.424483   1.827618   0.387509
O   4.502448   1.075158   0.697582
H   4.988232   0.304222   0.307314
H   1.023384   0.168879  -1.181607
H   1.681479  -1.353944  -1.807595
S   1.280136  -1.519673   0.537216
H   1.120730  -0.418447   1.298239
28
Dimer 67 of the n...n  type
C   2.001090  -0.769141  -0.406507
C   3.103360  -0.817907   0.655666
N   2.432565  -0.741044  -1.805654
H   3.280676  -0.189176  -1.910742
H   2.617201  -1.672697  -2.157673
H   1.373190  -1.656514  -0.269915
C   1.036641   0.402854  -0.162326
O   0.749796   0.820828   0.935960
O   0.502498   0.895281  -1.281627
H   0.973007   0.415430  -2.010250
H   2.662447  -0.884254   1.649289
H   3.727197  -1.695986   0.488169
S   4.252836   0.624990   0.603634
H   3.350631   1.540077   1.012700
C  -3.123006  -0.004971  -0.438505
C  -3.071252  -1.326881   0.336713
N  -2.446732   0.034418  -1.733854
H  -1.554867  -0.451692  -1.693406
H  -3.010144  -0.392176  -2.459592
```



```
H  -4.182320   0.218474  -0.613169
C  -2.639903   1.177286   0.421631
O  -2.804445   1.241641   1.619634
O  -2.073973   2.159430  -0.275688
H  -1.976843   1.804737  -1.192502
H  -3.626656  -1.233226   1.268844
H  -3.525372  -2.115031  -0.264503
S  -1.367644  -1.922508   0.709530
H  -1.004651  -0.886177   1.492424
28
Dimer 68 of the n...n  type
C   2.756740   0.607552  -0.748556
C   2.567870   1.523758   0.478407
N   1.727886   0.823064  -1.746281
H   0.800530   0.662833  -1.367043
H   1.847460   0.200312  -2.537952
H   3.727942   0.869466  -1.179147
C   2.884473  -0.832881  -0.250164
O   1.999388  -1.668490  -0.286187
O   4.082079  -1.075757   0.282314
H   4.105740  -1.980858   0.635105
H   3.373704   1.361762   1.193238
H   2.598995   2.560536   0.143328
S   0.940928   1.357146   1.331624
H   0.980365   0.031732   1.579723
C  -2.856641  -1.063527  -0.237066
C  -3.014282   0.185470  -1.119654
N  -3.769166  -1.039103   0.895256
H  -3.554439  -0.238640   1.484672
H  -3.624199  -1.862275   1.471623
H  -3.113494  -1.919238  -0.873522
C  -1.387731  -1.277700   0.151433
O  -0.998884  -1.426072   1.290515
O  -0.588849  -1.294238  -0.918954
H   0.352104  -1.455672  -0.654034
H  -2.392093   0.101075  -2.008388
H  -4.057115   0.269871  -1.425421
S  -2.634420   1.780704  -0.269934
H  -1.313116   1.566206  -0.079957
28
Dimer 69 of the n...n  type
C  -2.187292   0.620953   0.655598
C  -3.116577  -0.542366   1.013347
N  -2.792532   1.951335   0.568584
H  -3.729540   1.897192   0.176330
H  -2.856478   2.391897   1.478326
H  -1.400148   0.665620   1.417043
C  -1.415955   0.345892  -0.643043
O  -1.060955  -0.753422  -1.000830
O  -1.108350   1.451699  -1.326985
H  -1.585515   2.177977  -0.849106
H  -2.545172  -1.465897   1.095354
H  -3.596669  -0.339460   1.970643
S  -4.492782  -0.791090  -0.190300
H  -3.718463  -1.227927  -1.204406
C   1.889036   0.187149  -0.434346
C   3.303935   0.555797  -0.896488
N   1.301867   1.044982   0.598486
H   2.017079   1.382294   1.238363
H   0.844421   1.848841   0.185575
H   1.237289   0.222912  -1.311454
C   1.828300  -1.277447   0.031724
O   2.395668  -2.180984  -0.540633
O   1.093742  -1.475698   1.126084
H   0.764083  -0.576383   1.370038
H   3.623049  -0.111331  -1.695733
H   3.304164   1.579478  -1.271488
S   4.564584   0.523698   0.451701
H   4.558189  -0.813800   0.626778
```

```
28
Dimer 70 of the n...n  type
C   1.970457  -0.354905   0.803018
C   2.592857   1.038164   0.652406
N   2.771507  -1.361818   1.499682
H   3.760882  -1.238907   1.297967
H   2.644075  -1.313602   2.503208
H   1.032302  -0.233986   1.357142
C   1.538744  -0.926294  -0.558410
O   1.089236  -0.246006  -1.456032
O   1.651590  -2.247025  -0.649125
H   2.080670  -2.517763   0.204114
H   1.893738   1.716081   0.165573
H   2.830247   1.432538   1.640345
S   4.181701   1.045181  -0.283524
H   3.643175   0.740193  -1.481951
C  -1.802863  -0.391640  -0.167188
C  -1.356569   0.987664   0.330906
N  -1.951802  -0.543199  -1.614851
H  -2.361007   0.298054  -2.015418
H  -1.043037  -0.677484  -2.046251
H  -1.055147  -1.121346   0.164342
C  -3.096258  -0.854576   0.522583
O  -3.380194  -0.586939   1.668162
O  -3.872588  -1.614508  -0.251397
H  -3.418427  -1.619202  -1.131762
H  -1.197512   0.972097   1.408345
H  -0.424404   1.248138  -0.167697
S  -2.531321   2.346466  -0.081436
H  -3.512137   1.949310   0.754842
28
Dimer 71 of the n...n  type
C   3.277871  -0.078002  -0.459018
C   3.035813   1.433691  -0.389451
N   2.629597  -0.805014  -1.550978
H   1.689822  -0.454422  -1.716184
H   3.152471  -0.722954  -2.414739
H   4.359706  -0.223647  -0.563471
C   2.938043  -0.772269   0.872065
O   3.047796  -0.240993   1.954136
O   2.551020  -2.040010   0.733303
H   2.461748  -2.172594  -0.242649
H   3.615920   1.869386   0.422661
H   3.347572   1.891320  -1.328441
S   1.265214   1.902777  -0.175354
H   1.145379   1.423833   1.079132
C  -2.689714   0.686356  -0.483453
C  -1.634786  -0.153747  -1.211845
N  -2.203740   1.565672   0.582833
H  -1.450272   1.120843   1.100759
H  -1.862371   2.446204   0.216986
H  -3.184099   1.313479  -1.233835
C  -3.815959  -0.199435   0.078731
O  -4.225708  -1.194665  -0.474551
O  -4.328467   0.244272   1.227520
H  -3.776504   1.032914   1.459834
H  -2.101118  -0.740776  -2.001309
H  -0.889867   0.508220  -1.651101
S  -0.691636  -1.283628  -0.099457
H  -1.691356  -2.171192   0.080931
28
Dimer 72 of the n...n  type
C   2.357116  -0.214442  -0.721596
C   3.694630  -0.896328  -0.414264
N   2.418099   1.004825  -1.530960
H   3.283787   1.511787  -1.365347
H   2.351929   0.801815  -2.520684
H   1.728146  -0.939984  -1.245708
C   1.579592   0.083786   0.571302
```



```
O   1.524173  -0.677147   1.511304
O   0.939466   1.252546   0.560801
H   1.172956   1.650943  -0.317451
H   3.525776  -1.810960   0.152030
H   4.194596  -1.147953  -1.349664
S   4.884907   0.170288   0.510289
H   4.191908   0.193770   1.667340
C  -3.215511  -0.467491   0.767057
C  -1.731757  -0.793089   0.567383
N  -4.176966  -1.420670   0.205611
H  -3.824932  -1.820928  -0.660628
H  -4.369930  -2.179561   0.847982
H  -3.392803  -0.406978   1.846904
C  -3.551392   0.941306   0.244394
O  -2.797086   1.884469   0.323442
O  -4.771167   1.040584  -0.285703
H  -5.128769   0.116253  -0.257327
H  -1.109577  -0.051526   1.063126
H  -1.514555  -1.773402   0.991079
S  -1.225905  -0.894509  -1.203363
H  -1.299653   0.429101  -1.449435
28
Dimer 73 of the n...n  type
C  -2.944950  -0.733070  -0.438123
C  -3.474791   0.328388   0.531100
N  -2.375814  -1.944189   0.159378
H  -1.886336  -1.730752   1.024686
H  -3.092129  -2.629147   0.368493
H  -3.784353  -1.038989  -1.073393
C  -1.919383  -0.144076  -1.422021
O  -1.936025   1.000055  -1.814593
O  -1.000599  -1.025218  -1.823832
H  -1.194251  -1.842360  -1.297787
H  -3.948001   1.139403  -0.020195
H  -4.214716  -0.125666   1.189913
S  -2.190572   1.051171   1.642732
H  -1.593985   1.802339   0.696561
C   1.404371  -0.360356   0.355202
C   1.313663   0.743452  -0.702943
N   1.304625   0.056854   1.755214
H   1.739986   0.965913   1.892044
H   0.335618   0.125485   2.046774
H   0.589713  -1.062820   0.155635
C   2.678005  -1.204148   0.174964
O   3.158586  -1.474220  -0.902778
O   3.194871  -1.643294   1.323014
H   2.626000  -1.227725   2.021740
H   1.350096   0.307511  -1.698558
H   0.366990   1.271176  -0.601430
S   2.620285   2.034654  -0.545503
H   3.652111   1.244048  -0.905846
28
Dimer 74 of the n...n  type
C  -2.122182   0.093390   0.860262
C  -3.062986  -1.066037   0.513068
N  -2.691063   1.195843   1.636564
H  -3.663941   1.352032   1.384266
H  -2.646705   1.015199   2.632076
H  -1.286890  -0.325417   1.433983
C  -1.460146   0.670896  -0.400198
O  -1.121845  -0.002878  -1.349284
O  -1.226931   1.979290  -0.336288
H  -1.640363   2.261995   0.521140
H  -2.518685  -1.844079  -0.020236
H  -3.467831  -1.487660   1.433031
S  -4.526478  -0.559310  -0.489095
H  -3.840931  -0.289143  -1.618760
C   1.865937   0.375775  -0.196461
C   1.504769  -0.963620   0.456923
```

```
N   1.905565   0.397015  -1.659415
H   2.309376  -0.467088  -2.013989
H   0.963673   0.471174  -2.030622
H   1.119567   1.112344   0.120721
C   3.188861   0.935191   0.349732
O   3.563205   0.786939   1.491346
O   3.882669   1.637667  -0.547409
H   3.365737   1.545676  -1.387862
H   1.412236  -0.847868   1.535963
H   0.554179  -1.307650   0.050735
S   2.705002  -2.316692   0.097217
H   3.720373  -1.805214   0.822683
28
Dimer 75 of the n...n  type
C  -3.158494   0.674716  -0.374578
C  -3.536053  -0.145117   0.864723
N  -3.404580   0.040564  -1.672270
H  -3.243673  -0.962627  -1.624917
H  -4.353493   0.194074  -1.991195
H  -3.734360   1.606341  -0.340270
C  -1.688264   1.122864  -0.320595
O  -1.108363   1.425008   0.697804
O  -1.093332   1.151924  -1.515198
H  -1.781725   0.809355  -2.142859
H  -3.381990   0.440945   1.769245
H  -4.588094  -0.422796   0.801941
S  -2.600844  -1.730094   1.018894
H  -1.472670  -1.178594   1.503067
C   1.912488  -0.207489  -0.796862
C   2.010683   1.254909  -0.347665
N   0.833606  -0.992661  -0.186372
H   0.572410  -0.585455   0.707192
H   0.010189  -1.005463  -0.774282
H   1.771873  -0.207206  -1.882819
C   3.247211  -0.941997  -0.567065
O   4.330557  -0.452090  -0.798810
O   3.101269  -2.180658  -0.100067
H   2.117112  -2.272533   0.021687
H   2.833986   1.747997  -0.861883
H   1.078741   1.763450  -0.578733
S   2.231927   1.456674   1.472827
H   3.504706   1.011126   1.519207
28
Dimer 76 of the n...n  type
C  -3.049040  -0.299067  -0.759167
C  -2.159612   0.948406  -0.776916
N  -4.451641  -0.099237  -0.385120
H  -4.538118   0.637591   0.310644
H  -5.020385   0.152591  -1.184397
H  -3.026188  -0.728011  -1.767330
C  -2.450614  -1.398922   0.136550
O  -1.261839  -1.603001   0.245568
O  -3.364897  -2.132282   0.769593
H  -4.228955  -1.705928   0.533546
H  -1.159581   0.698910  -1.125372
H  -2.587098   1.685598  -1.456991
S  -2.031070   1.802559   0.851261
H  -1.370193   0.813565   1.484933
C   2.689969  -0.112640   0.860669
C   1.389629   0.564483   0.416080
N   3.812739   0.774309   1.178731
H   3.808115   1.592013   0.573790
H   3.777232   1.092869   2.139440
H   2.459836  -0.699673   1.757222
C   3.159686  -1.155041  -0.170544
O   2.407054  -1.848533  -0.816647
O   4.486245  -1.246579  -0.273539
H   4.829430  -0.533706   0.324288
H   0.607485  -0.177847   0.267580
```



```
H   1.069710   1.266576   1.185312
S   1.561001   1.572945  -1.119060
H   1.759509   0.531728  -1.952992
28
Dimer 77 of the n...n  type
C  -2.838337   0.271316  -0.166823
C  -2.779033  -1.204518  -0.573859
N  -2.402513   1.251592  -1.163431
H  -1.591951   0.908584  -1.674421
H  -3.135439   1.455332  -1.832285
H  -3.881910   0.496153   0.084266
C  -2.073169   0.533787   1.142373
O  -1.925910  -0.284342   2.019834
O  -1.604798   1.782486   1.240939
H  -1.812069   2.194976   0.364830
H  -3.171125  -1.828315   0.228293
H  -3.388391  -1.355503  -1.465025
S  -1.090268  -1.793891  -1.017447
H  -0.571702  -1.682045   0.220946
C   2.838417  -0.271226  -0.166770
C   2.778936   1.204628  -0.573772
N   2.402820  -1.251563  -1.163405
H   1.592162  -0.908735  -1.674372
H   3.135782  -1.455127  -1.832275
H   3.882010  -0.495888   0.084390
C   2.073188  -0.533781   1.142367
O   1.925668   0.284372   2.019775
O   1.604982  -1.782518   1.240922
H   1.812612  -2.195031   0.364931
H   3.170883   1.828434   0.228440
H   3.388353   1.355739  -1.464880
S   1.090134   1.793808  -1.017464
H   0.571249   1.681334   0.220748
28
Dimer 78 of the n...n  type
C   2.171064   0.521655  -0.706738
C   1.686493  -0.924983  -0.832772
N   3.591824   0.756215  -0.984288
H   4.159637  -0.005130  -0.619946
H   3.768004   0.830097  -1.979165
H   1.581327   1.112327  -1.415208
C   1.835565   1.143625   0.659391
O   0.866969   0.853951   1.324843
O   2.708283   2.077172   1.045483
H   3.424433   2.044457   0.361452
H   0.602749  -0.956092  -0.785936
H   1.995951  -1.314146  -1.803214
S   2.399029  -2.084050   0.410653
H   1.772832  -1.550916   1.479337
C  -2.171190   0.521623   0.706816
C  -1.686471  -0.925016   0.832755
N  -3.592002   0.755881   0.984204
H  -4.159646  -0.005735   0.620181
H  -3.768301   0.830318   1.979010
H  -1.581628   1.112346   1.415408
C  -1.835587   1.143756  -0.659241
O  -0.866552   0.854645  -1.324303
O  -2.708724   2.076740  -1.045689
H  -3.424985   2.043700  -0.361751
H  -0.602727  -0.956031   0.785996
H  -1.996005  -1.314275   1.803131
S  -2.398814  -2.084022  -0.410850
H  -1.772880  -1.550478  -1.479484
28
Dimer 79 of the n...n  type
C   3.100540  -0.016920   0.702061
C   3.161253  -1.262806  -0.187517
N   2.384122  -0.156418   1.973037
H   1.623782  -0.826633   1.892240
```

```
H   2.997341  -0.470687   2.715366
H   4.133248   0.272619   0.927644
C   2.530788   1.192737  -0.062714
O   2.709895   1.384589  -1.243583
O   1.841181   2.035456   0.706781
H   1.806809   1.593248   1.592448
H   3.754899  -1.061318  -1.077609
H   3.622910  -2.079776   0.367007
S   1.503494  -1.888092  -0.703591
H   1.219255  -0.877065  -1.549425
C  -2.135744  -0.440138   0.693921
C  -1.088454   0.640906   0.408507
N  -1.972403  -1.714823  -0.009522
H  -1.641329  -1.556336  -0.958602
H  -1.293332  -2.305995   0.454682
H  -2.101213  -0.646146   1.770051
C  -3.563299   0.079752   0.450509
O  -3.907013   1.221535   0.657499
O  -4.407564  -0.855475   0.012811
H  -3.844500  -1.661051  -0.111626
H  -1.280979   1.526434   1.011570
H  -0.105966   0.252164   0.661147
S  -0.971338   1.127769  -1.364064
H  -2.209960   1.654376  -1.453704
28
Dimer 80 of the n...n  type
C   3.026002   0.979580   0.086458
C   3.567433  -0.374627  -0.386600
N   2.806258   1.129387   1.527398
H   2.493387   0.254025   1.940190
H   3.650339   1.418630   2.006644
H   3.746195   1.745223  -0.224094
C   1.728493   1.355174  -0.649996
O   1.495174   1.061467  -1.800272
O   0.872507   2.055927   0.096555
H   1.293667   2.086693   0.993958
H   3.731418  -0.358752  -1.462914
H   4.517160  -0.572896   0.110135
S   2.479495  -1.806373   0.030836
H   1.526583  -1.545796  -0.884402
C  -1.996504   0.545996   0.499802
C  -0.911376  -0.452316   0.092913
N  -2.572964   0.377157   1.836132
H  -2.628830  -0.607644   2.084562
H  -2.021048   0.849147   2.542123
H  -1.538141   1.539202   0.446938
C  -3.133918   0.584854  -0.533431
O  -2.963892   0.453568  -1.724953
O  -4.336719   0.806399  -0.000757
H  -4.174447   0.819544   0.977037
H  -0.522402  -0.193147  -0.889183
H  -0.095566  -0.406463   0.812157
S  -1.452074  -2.213551   0.079892
H  -2.267461  -2.105654  -0.988960
28
Dimer 81 of the n...n  type
C  -2.110414   0.487177   0.632420
C  -2.706474  -0.913977   0.794624
N  -2.855398   1.597728   1.229279
H  -3.857874   1.445650   1.147390
H  -2.632578   1.710638   2.210864
H  -1.116026   0.462368   1.090455
C  -1.837007   0.811849  -0.844760
O  -1.507449  -0.013551  -1.666602
O  -1.954409   2.107222  -1.139264
H  -2.289105   2.521506  -0.303647
H  -2.035344  -1.652062   0.360390
H  -2.828517  -1.131903   1.855900
S  -4.384575  -1.103062   0.050812
```



```
H  -3.981610  -1.012424  -1.232968
C   2.986331   0.385546  -0.677745
C   1.485753   0.245962  -0.400658
N   3.572496  -0.598911  -1.590975
H   3.119318  -1.503512  -1.485869
H   3.482683  -0.311624  -2.558007
H   3.146634   1.381122  -1.106807
C   3.797723   0.395869   0.630827
O   3.410172   0.910817   1.655557
O   4.987487  -0.196672   0.529632
H   5.004054  -0.560875  -0.392522
H   1.147223   1.060065   0.239693
H   0.937143   0.286425  -1.340041
S   1.012698  -1.363369   0.365937
H   1.652480  -1.161282   1.536211
28
Dimer 82 of the n...n  type
C   2.198120  -1.261467  -0.446371
C   3.401281  -0.312859  -0.450587
N   1.233377  -1.102449  -1.537324
H   1.137052  -0.120534  -1.785398
H   1.525707  -1.605510  -2.366633
H   2.593088  -2.283160  -0.497411
C   1.446460  -1.226249   0.896285
O   1.974634  -0.994539   1.959152
O   0.151606  -1.530564   0.791992
H  -0.023918  -1.598110  -0.178404
H   4.058315  -0.540181   0.387509
H   3.957620  -0.442293  -1.379046
S   2.951382   1.474778  -0.391505
H   2.423161   1.450105   0.848648
C  -2.197997   1.261129  -0.447251
C  -3.401116   0.312518  -0.451332
N  -1.232802   1.101385  -1.537733
H  -1.136595   0.119353  -1.785368
H  -1.524596   1.604203  -2.367375
H  -2.592957   2.282779  -0.499143
C  -1.446758   1.226851   0.895695
O  -1.975232   0.995743   1.958547
O  -0.151958   1.531380   0.791552
H   0.023694   1.598001  -0.178908
H  -4.058477   0.540343   0.386373
H  -3.957111   0.441333  -1.380079
S  -2.951211  -1.475092  -0.390851
H  -2.424089  -1.449555   0.849755
28
Dimer 83 of the n...n  type
C  -2.661274   0.135776  -0.949903
C  -1.238423   0.607210  -0.631196
N  -3.683939   1.181850  -1.046800
H  -3.507642   1.927979  -0.378675
H  -3.716155   1.589572  -1.973370
H  -2.624267  -0.386116  -1.912874
C  -3.139555  -0.934266   0.048686
O  -2.421547  -1.789647   0.513063
O  -4.440832  -0.857765   0.335156
H  -4.755159  -0.054319  -0.152599
H  -0.549169  -0.234233  -0.635966
H  -0.921008   1.326579  -1.386173
S  -1.094233   1.466974   0.997296
H  -0.914267   0.347608   1.725135
C   3.202238  -0.868443   0.105606
C   3.489779   0.553498   0.603432
N   3.469287  -1.123458  -1.312788
H   3.303817  -0.287856  -1.868547
H   4.423875  -1.423790  -1.467680
H   3.819320  -1.553795   0.697032
C   1.754502  -1.283941   0.421653
O   1.185278  -1.005214   1.452526
```

```
O   1.179377  -2.002834  -0.543425
H   1.864207  -2.040088  -1.261219
H   3.263826   0.630983   1.665910
H   4.545237   0.779528   0.451209
S   2.559881   1.864956  -0.304300
H   1.357751   1.643009   0.278768
28
Dimer 84 of the n...n  type
C   2.528236   0.262541   0.839039
C   1.921754  -1.144596   0.781085
N   1.763928   1.339392   0.209520
H   1.319050   1.021886  -0.647494
H   1.041555   1.708939   0.815299
H   2.655899   0.517786   1.897302
C   3.958432   0.273860   0.266806
O   4.731438  -0.651589   0.369627
O   4.285786   1.418356  -0.334347
H   3.454068   1.956460  -0.315461
H   2.576010  -1.854159   1.285157
H   0.957460  -1.148806   1.289004
S   1.595094  -1.746742  -0.932494
H   2.889517  -1.908310  -1.274471
C  -2.720304  -0.636635  -0.475525
C  -1.419151   0.167193  -0.559468
N  -3.727907  -0.365424  -1.502272
H  -3.720101   0.616223  -1.768232
H  -3.571958  -0.919302  -2.335659
H  -2.449971  -1.696792  -0.539092
C  -3.367235  -0.487724   0.913683
O  -2.732890  -0.393958   1.940319
O  -4.699771  -0.498022   0.889695
H  -4.930550  -0.533884  -0.073393
H  -0.749526  -0.137992   0.240578
H  -0.932076  -0.022810  -1.515372
S  -1.648432   1.993759  -0.464714
H  -2.025010   2.024573   0.830349
28
Dimer 85 of the n...n  type
C   1.969319  -0.101051   0.776267
C   1.904667   1.300973   0.159389
N   3.001962  -0.330509   1.786916
H   3.845691   0.194312   1.569542
H   2.688424  -0.058380   2.710759
H   0.998051  -0.302152   1.241160
C   2.088365  -1.178217  -0.316208
O   1.549363  -1.094097  -1.397536
O   2.812115  -2.235181   0.045873
H   3.158389  -2.002891   0.944937
H   1.093191   1.352140  -0.563942
H   1.725825   2.035261   0.944995
S   3.465260   1.826242  -0.670638
H   3.380845   0.955247  -1.697111
C  -2.374163   0.768743  -0.529481
C  -1.695174  -0.507644  -1.039959
N  -1.671259   1.523863   0.510264
H  -1.174449   0.904873   1.144919
H  -1.016544   2.191643   0.124418
H  -2.506600   1.430926  -1.392438
C  -3.808793   0.481068  -0.045331
O  -4.539724  -0.334361  -0.560603
O  -4.190094   1.236636   0.984808
H  -3.382411   1.760914   1.219682
H  -2.310908  -0.971183  -1.809110
H  -0.719460  -0.268977  -1.461613
S  -1.342018  -1.748588   0.277887
H  -2.625448  -1.984882   0.619160
28
Dimer 86 of the n...n  type
C   2.673546   0.757507   0.447680
```



```
C   2.295248  -0.276991   1.514784
N   1.573585   1.430808  -0.243385
H   0.806583   0.792228  -0.425087
H   1.219218   2.222530   0.279074
H   3.273328   1.527811   0.945835
C   3.623340   0.151272  -0.601833
O   4.455624  -0.688391  -0.344561
O   3.468124   0.654952  -1.826662
H   2.698498   1.271922  -1.752429
H   3.192661  -0.654503   2.002143
H   1.663513   0.193436   2.268575
S   1.325771  -1.712334   0.876368
H   2.313465  -2.235497   0.122470
C  -2.053577  -0.666097   0.021800
C  -1.711032   0.443314   1.024113
N  -1.346478  -0.660659  -1.258345
H  -1.227168   0.284262  -1.614529
H  -0.439720  -1.107074  -1.183761
H  -1.831761  -1.619503   0.514817
C  -3.568787  -0.715942  -0.250567
O  -4.410406  -0.433588   0.571509
O  -3.879562  -1.133926  -1.478572
H  -3.008752  -1.232781  -1.938409
H  -2.338530   0.350033   1.908853
H  -0.671736   0.351832   1.333184
S  -1.866834   2.149118   0.346280
H  -3.205021   2.124969   0.179438
28
Dimer 87 of the n...n  type
C  -2.535191  -0.759502   0.858359
C  -3.519630   0.415283   0.835321
N  -1.286254  -0.567801   1.600504
H  -0.923038   0.368523   1.433831
H  -1.431135  -0.677107   2.597530
H  -3.061104  -1.606977   1.314861
C  -2.201292  -1.258259  -0.557238
O  -2.923894  -1.121680  -1.515740
O  -1.038924  -1.918453  -0.630734
H  -0.626749  -1.805539   0.256451
H  -4.410640   0.138620   0.273219
H  -3.812396   0.655452   1.857777
S  -2.832776   1.979810   0.146805
H  -2.619556   1.511541  -1.099280
C   2.722696  -0.810824  -0.111770
C   1.839723  -0.512407  -1.329064
N   2.040498  -1.253057   1.106244
H   1.137976  -0.800434   1.225030
H   1.903596  -2.256049   1.123476
H   3.424486  -1.599321  -0.406671
C   3.621771   0.391627   0.230998
O   4.107156   1.124812  -0.600853
O   3.844066   0.541337   1.537041
H   3.289452  -0.160793   1.963859
H   2.460461  -0.259583  -2.187239
H   1.243043  -1.391499  -1.569444
S   0.615017   0.838447  -1.054638
H   1.511789   1.843400  -0.985349
28
Dimer 88 of the n...n  type
C   3.139650   0.045431  -0.755270
C   3.179622   1.227052   0.236259
N   2.256319   0.306643  -1.873802
H   1.283675   0.348581  -1.579279
H   2.322887  -0.439609  -2.558372
H   4.162381  -0.066925  -1.129026
C   2.823961  -1.237731   0.014182
O   1.748085  -1.805387   0.017330
O   3.871078  -1.669610   0.722447
H   3.616823  -2.456965   1.231753
```

```
H   3.901977   1.032331   1.028241
H   3.491773   2.116490  -0.311013
S   1.559506   1.685766   0.990718
H   1.397611   0.580495   1.745293
C  -2.359576   0.907220   0.336324
C  -3.372258  -0.003240   1.044870
N  -3.015992   1.823030  -0.584359
H  -3.484268   1.290166  -1.313358
H  -2.319438   2.391623  -1.056249
H  -1.868032   1.496997   1.120845
C  -1.228522   0.094823  -0.301999
O  -0.822194   0.272885  -1.431089
O  -0.723909  -0.810539   0.537670
H   0.096235  -1.208071   0.163750
H  -2.891724  -0.562681   1.844446
H  -4.163118   0.613887   1.470660
S  -4.230667  -1.194319  -0.077837
H  -3.172242  -2.007313  -0.273743
28
Dimer 89 of the n...n  type
C  -2.665482   0.022507   0.849421
C  -1.614620   0.750579   0.004159
N  -2.387749  -1.375322   1.183566
H  -1.943748  -1.855051   0.404485
H  -1.780888  -1.454239   1.990646
H  -2.763415   0.577045   1.789762
C  -4.060283   0.113749   0.204628
O  -4.445323   1.070474  -0.428390
O  -4.827529  -0.953583   0.429678
H  -4.242320  -1.585985   0.917039
H  -1.900750   1.790467  -0.143909
H  -0.661258   0.723876   0.529987
S  -1.303815  -0.025845  -1.638826
H  -2.506774   0.268365  -2.172396
C   2.456735  -0.377587  -0.684449
C   2.112613   1.115275  -0.608851
N   1.535851  -1.310500  -0.032862
H   1.132778  -0.912579   0.810659
H   0.788756  -1.608389  -0.647001
H   2.506275  -0.642993  -1.746403
C   3.881109  -0.638993  -0.155618
O   4.803773   0.129659  -0.305755
O   4.016333  -1.810649   0.465135
H   3.100072  -2.188852   0.480933
H   2.877977   1.695239  -1.121924
H   1.156412   1.298211  -1.097380
S   1.925568   1.757414   1.109822
H   3.213674   1.590133   1.473554
28
Dimer 90 of the n...n  type
C   2.916450  -0.063243   0.957015
C   3.194033  -1.259249   0.040953
N   1.878481  -0.244543   1.976068
H   1.150267  -0.872579   1.647290
H   2.258526  -0.633544   2.830601
H   3.854090   0.172863   1.473188
C   2.604913   1.209970   0.148434
O   3.088712   1.455730  -0.932773
O   1.767372   2.044003   0.766534
H   1.482080   1.554447   1.578732
H   4.012675  -1.029379  -0.638912
H   3.472258  -2.120684   0.648146
S   1.734526  -1.805655  -0.948621
H   1.699370  -0.736602  -1.770277
C  -1.904719  -0.522201   0.467363
C  -1.034543   0.660163   0.030210
N  -1.889343  -1.699007  -0.404959
H  -1.842085  -1.415220  -1.381214
H  -1.078251  -2.276876  -0.218165
```



```
H  -1.554406  -0.836129   1.457913
C  -3.362934  -0.091743   0.699777
O  -3.681397   0.989197   1.141171
O  -4.255752  -1.034322   0.394702
H  -3.717003  -1.775034   0.016938
H  -1.066027   1.455062   0.772753
H  -0.010083   0.317081  -0.075282
S  -1.469360   1.349001  -1.620455
H  -2.658725   1.868856  -1.253831
28
Dimer 91 of the n...n  type
C  -2.202537  -0.516114  -0.618800
C  -3.706907  -0.420709  -0.930728
N  -1.916978  -1.609313   0.289185
H  -2.253835  -1.395736   1.223165
H  -0.917418  -1.760979   0.346065
H  -1.689853  -0.710580  -1.566187
C  -1.677557   0.841165  -0.147094
O  -1.090149   1.039402   0.899286
O  -1.957584   1.820509  -1.011679
H  -1.601951   2.659458  -0.675322
H  -3.895364   0.311791  -1.713204
H  -4.048238  -1.397977  -1.270267
S  -4.760903  -0.016705   0.531770
H  -4.424839   1.287229   0.614493
C   3.084286  -1.150903   0.238057
C   3.984630  -0.022874   0.768532
N   3.550111  -1.642752  -1.048869
H   3.485414  -0.897938  -1.738478
H   2.939241  -2.384263  -1.377507
H   3.146432  -1.966115   0.968708
C   1.619228  -0.701301   0.234468
O   0.898747  -0.734274  -0.741977
O   1.221734  -0.266136   1.434345
H   0.308230   0.100551   1.364191
H   3.720685   0.225833   1.794226
H   5.021272  -0.357923   0.739569
S   3.941387   1.522228  -0.245279
H   2.701700   1.911123   0.118074
28
Dimer 92 of the n...n  type
C   2.764253   0.271164   0.940336
C   1.451737   0.417180   0.163517
N   3.585502   1.476980   1.068176
H   3.512692   2.054966   0.234393
H   3.309894   2.036418   1.866143
H   2.507775  -0.071859   1.949150
C   3.629124  -0.862234   0.358305
O   3.172773  -1.886001  -0.098468
O   4.939223  -0.625923   0.425335
H   5.006795   0.293365   0.790133
H   0.910109  -0.526782   0.165088
H   0.838021   1.182351   0.636381
S   1.667015   0.972843  -1.580698
H   2.266035  -0.154470  -2.016078
C  -2.101164   0.260783   0.562202
C  -3.468109   0.305880   1.256941
N  -1.789678   1.365505  -0.343746
H  -2.618486   1.649632  -0.860547
H  -1.444611   2.175212   0.157517
H  -1.339381   0.245606   1.349811
C  -1.903452  -1.070548  -0.184266
O  -2.334382  -2.129220   0.213422
O  -1.181596  -0.969880  -1.300786
H  -0.984711  -0.006656  -1.409831
H  -3.570451  -0.534304   1.941970
H  -3.552332   1.234009   1.822467
S  -4.896591   0.305162   0.088939
H  -4.741377  -0.968122  -0.327697
```

```
28
Dimer 93 of the n...n  type
C  -2.726720  -0.511310   0.688968
C  -1.955138   0.772893   1.015515
N  -2.027179  -1.506120  -0.125252
H  -1.424452  -1.056751  -0.810043
H  -1.455934  -2.124207   0.437900
H  -2.994633  -0.982035   1.641607
C  -4.078790  -0.188737   0.025181
O  -4.754405   0.773656   0.312331
O  -4.457040  -1.085761  -0.885407
H  -3.695160  -1.717315  -0.942364
H  -2.560552   1.424976   1.642973
H  -1.042641   0.522326   1.554940
S  -1.411349   1.712922  -0.474310
H  -2.641740   2.115808  -0.851759
C   2.726591   0.511335   0.688856
C   1.954996  -0.772843   1.015416
N   2.027067   1.506053  -0.125540
H   1.424552   1.056497  -0.810398
H   1.455540   2.123994   0.437482
H   2.994306   0.982171   1.641497
C   4.078756   0.188846   0.025250
O   4.754359  -0.773552   0.312464
O   4.457099   1.085925  -0.885211
H   3.695122   1.717423  -0.942190
H   2.560268  -1.424805   1.643146
H   1.042440  -0.522089   1.554671
S   1.411556  -1.713099  -0.474374
H   2.642060  -2.115940  -0.851505
28
Dimer 94 of the n...n  type
C  -2.659150  -1.023362   0.562543
C  -3.532048   0.232359   0.649624
N  -1.539361  -1.110222   1.506038
H  -1.167699  -0.184643   1.703017
H  -1.826338  -1.519635   2.387197
H  -3.312291  -1.885838   0.739017
C  -2.124365  -1.246046  -0.863284
O  -2.706862  -0.903579  -1.865836
O  -0.957023  -1.894285  -0.899584
H  -0.689675  -1.977548   0.048192
H  -4.348531   0.169553  -0.067957
H  -3.951242   0.310517   1.652755
S  -2.613650   1.808428   0.373543
H  -2.373399   1.607833  -0.938202
C   1.801445  -0.153493   0.692431
C   0.992221   0.677359  -0.309115
N   2.385208   0.573692   1.822510
H   2.663412   1.511623   1.544056
H   1.733561   0.653939   2.593759
H   1.132282  -0.918518   1.101272
C   2.909738  -0.952014  -0.015659
O   2.783532  -1.443224  -1.114543
O   4.023405  -1.081421   0.706214
H   3.852206  -0.545434   1.522328
H   0.575998   0.034477  -1.081006
H   0.172541   1.174424   0.208358
S   1.951475   2.032983  -1.108967
H   2.741566   1.233650  -1.855071
28
Dimer 95 of the n...n  type
C   2.544624   0.503242   0.834202
C   1.971706  -0.872164   1.196956
N   1.661782   1.413696   0.105660
H   1.140464   0.910356  -0.608096
H   0.994741   1.865376   0.720417
H   2.829982   0.993401   1.772163
C   3.865655   0.369878   0.055377
```



```
O   4.657920  -0.528751   0.223903
O   4.081381   1.360161  -0.812626
H   3.259804   1.909551  -0.782392
H   2.720249  -1.463834   1.721587
H   1.112285  -0.742954   1.855318
S   1.354347  -1.827223  -0.255390
H   2.560524  -2.021669  -0.826256
C  -1.849642  -0.003886   0.468671
C  -3.148104  -0.711009   0.874102
N  -1.659960   1.356713   0.978953
H  -2.549490   1.848410   1.029689
H  -1.257078   1.350626   1.908675
H  -1.023802  -0.621494   0.833503
C  -1.676228   0.003725  -1.060336
O  -1.987117  -0.920271  -1.773733
O  -1.125321   1.125564  -1.534689
H  -1.055997   1.720750  -0.746238
H  -3.157393  -1.727249   0.483094
H  -3.210424  -0.751254   1.961624
S  -4.674541   0.164112   0.320599
H  -4.516471  -0.085438  -0.995402
28
Dimer 96 of the n...n  type
C  -3.216806   0.391601   0.458738
C  -2.940295  -1.083130   0.809593
N  -4.144838   0.501004  -0.656351
H  -3.710744   0.116559  -1.491822
H  -4.332811   1.478252  -0.858098
H  -3.674388   0.844342   1.344908
C  -1.889999   1.117774   0.229158
O  -1.518557   1.541218  -0.843572
O  -1.168147   1.211945   1.355862
H  -0.295803   1.610197   1.138559
H  -2.330411  -1.147608   1.710010
H  -3.894233  -1.576701   0.993961
S  -2.105854  -2.042510  -0.531890
H  -0.842305  -1.603693  -0.234863
C   1.912501  -0.789500  -0.399760
C   1.617942   0.543614  -1.108775
N   0.906251  -1.113661   0.597012
H   0.879047  -0.410903   1.329329
H   1.132271  -1.992639   1.052925
H   1.884024  -1.556966  -1.180656
C   3.340621  -0.784656   0.152629
O   3.623614  -0.821455   1.326639
O   4.248672  -0.708602  -0.836081
H   5.139586  -0.680068  -0.451506
H   2.325272   0.691588  -1.922409
H   0.604399   0.520418  -1.507106
S   1.639485   2.043546  -0.028535
H   2.819662   1.820170   0.586652
28
Dimer 97 of the n...n  type
C   2.039467  -0.286786   0.624632
C   1.514795   0.596425  -0.512439
N   2.300174   0.382409   1.900435
H   2.638093   1.330032   1.751579
H   1.469080   0.429036   2.477436
H   1.289184  -1.065670   0.798630
C   3.299408  -1.056829   0.194187
O   3.471948  -1.487303  -0.923920
O   4.181545  -1.235828   1.177661
H   3.799649  -0.739419   1.945814
H   1.305975  -0.009351  -1.392970
H   0.594527   1.080926  -0.190117
S   2.646876   1.971936  -0.985224
H   3.605581   1.190018  -1.522334
C  -2.804434   0.814388  -0.238718
C  -2.033587   0.038783  -1.313896
```

```
N  -2.045728   1.253840   0.933098
H  -1.366986   0.552125   1.215832
H  -1.564789   2.130099   0.771883
H  -3.222031   1.706201  -0.719621
C  -4.038141   0.024525   0.237612
O  -4.690567  -0.697534  -0.481375
O  -4.347724   0.229582   1.518519
H  -3.624142   0.810171   1.863040
H  -2.699667  -0.221982  -2.134655
H  -1.228849   0.660377  -1.704979
S  -1.222036  -1.496011  -0.691257
H  -2.360877  -2.175452  -0.445928
28
Dimer 98 of the n...n  type
C   2.203107   0.287475  -1.091420
C   3.049812   1.351623  -0.372002
N   0.790077   0.616847  -1.050650
H   0.460333   0.585369  -0.089295
H   0.255175  -0.060439  -1.583300
H   2.536110   0.276556  -2.135761
C   2.519672  -1.109431  -0.546060
O   1.693599  -1.900678  -0.133025
O   3.826694  -1.378623  -0.569682
H   3.982308  -2.272465  -0.222281
H   4.111057   1.163556  -0.520911
H   2.801478   2.326640  -0.790020
S   2.712989   1.493408   1.437545
H   3.336674   0.354758   1.804201
C  -3.111727  -0.860134   0.411089
C  -3.367849  -0.151774  -0.935021
N  -3.831964  -0.212548   1.495186
H  -3.449239   0.716691   1.648941
H  -3.695856  -0.726584   2.360097
H  -3.489777  -1.880895   0.294508
C  -1.603098  -0.969995   0.640356
O  -0.990370  -0.316637   1.459880
O  -1.029545  -1.829623  -0.205286
H  -0.046987  -1.832869  -0.087888
H  -2.867499  -0.681860  -1.744309
H  -4.441106  -0.154112  -1.125251
S  -2.887389   1.631964  -0.993696
H  -1.551573   1.467504  -1.078805
28
Dimer 99 of the n...n  type
C   2.043172  -0.039342   0.887842
C   3.580748   0.025222   0.939549
N   1.557148  -1.404079   0.960815
H   1.807536  -1.912361   0.117687
H   0.544792  -1.397689   1.031251
H   1.674828   0.506288   1.761653
C   1.541613   0.736849  -0.331601
O   0.988583   0.247502  -1.290695
O   1.798062   2.051842  -0.222185
H   1.480162   2.505814  -1.019405
H   3.918673   1.059413   0.983864
H   3.917404  -0.495095   1.836007
S   4.435185  -0.827516  -0.458163
H   4.108061   0.061592  -1.418426
C  -2.043726  -0.037851  -0.888131
C  -3.581343   0.027219  -0.938738
N  -1.558057  -1.402544  -0.963173
H  -1.807883  -1.911929  -0.120556
H  -0.545765  -1.396134  -1.034401
H  -1.675816   0.508892  -1.761405
C  -1.541359   0.736615   0.332122
O  -0.989704   0.245647   1.291191
O  -1.795492   2.052115   0.223536
H  -1.477266   2.504928   1.021288
H  -3.918925   1.061614  -0.981022
```



```
H  -3.918647  -0.491391  -1.835945
S  -4.435175  -0.827859   0.457867
H  -4.107166   0.059298   1.419630
28
Dimer 100 of the n...n  type
C   2.607758  -1.008033   0.628522
C   2.716637   0.496683   0.953577
N   3.773953  -1.491156  -0.098002
H   3.810851  -1.060902  -1.017890
H   3.706389  -2.494204  -0.240732
H   2.555693  -1.527398   1.588709
C   1.279001  -1.244208  -0.091457
O   1.123104  -1.086400  -1.286805
O   0.304425  -1.598082   0.746941
H  -0.561425  -1.653559   0.268024
H   1.801214   0.840347   1.437330
H   3.552825   0.646737   1.637248
S   3.092428   1.600592  -0.478201
H   2.053436   1.231929  -1.253281
C  -2.987533   0.315602   0.737195
C  -2.499465   1.661140   0.161641
N  -2.268746  -0.041323   1.943714
H  -1.268818  -0.089572   1.775797
H  -2.567042  -0.944391   2.296221
H  -4.046150   0.451774   0.979519
C  -2.939125  -0.732310  -0.376331
O  -2.093518  -1.601253  -0.486260
O  -3.926548  -0.572714  -1.258547
H  -3.833234  -1.223034  -1.974448
H  -3.074419   1.921591  -0.725972
H  -2.650321   2.432775   0.916608
S  -0.697074   1.735147  -0.225130
H  -0.664446   0.757770  -1.155314
28
Dimer 101 of the n...n  type
C   3.057171  -0.899698   0.035075
C   3.251018   0.395948   0.841032
N   3.658927  -0.793219  -1.285209
H   3.157484  -0.095589  -1.829875
H   3.551540  -1.669602  -1.786506
H   3.577739  -1.687184   0.593349
C   1.584232  -1.318952   0.003174
O   0.962244  -1.558037  -1.007617
O   1.050610  -1.382473   1.232549
H   0.101056  -1.616231   1.171895
H   2.910241   0.268430   1.866092
H   4.313649   0.636347   0.850989
S   2.411252   1.882578   0.124043
H   1.230466   1.740205   0.754790
C  -2.063102  -0.383525  -0.856000
C  -1.116833   0.760166  -0.453456
N  -3.023980   0.044883  -1.855284
H  -3.652827   0.737826  -1.458565
H  -3.599010  -0.737496  -2.150735
H  -1.418569  -1.159261  -1.280324
C  -2.701483  -0.992837   0.392771
O  -3.873661  -0.970510   0.666339
O  -1.773626  -1.577829   1.199361
H  -2.210322  -1.934779   1.990041
H  -0.375300   0.401022   0.249342
H  -0.598042   1.114629  -1.342779
S  -1.921741   2.248323   0.275797
H  -2.255457   1.689795   1.457663
28
Dimer 102 of the n...n  type
C   2.583299   0.027583  -0.808485
C   2.978850   0.656193   0.535942
N   2.070603   1.027902  -1.731858
H   1.272860   1.497189  -1.310111
```

```
H   1.731542   0.582615  -2.578898
H   3.501045  -0.402258  -1.231247
C   1.641613  -1.169218  -0.616663
O   0.692300  -1.412239  -1.329949
O   1.994291  -1.933352   0.425538
H   1.295707  -2.589301   0.585263
H   3.496919  -0.069785   1.158189
H   3.645190   1.497520   0.346634
S   1.566148   1.360222   1.496775
H   0.918567   0.196304   1.713969
C  -2.583416   0.028112   0.808394
C  -2.978665   0.656382  -0.536252
N  -2.070646   1.028587   1.731566
H  -1.272680   1.497499   1.309812
H  -1.731868   0.583452   2.578803
H  -3.501291  -0.401464   1.231145
C  -1.641861  -1.168840   0.617016
O  -0.692416  -1.411626   1.330154
O  -1.994878  -1.933455  -0.424798
H  -1.296372  -2.589531  -0.584328
H  -3.496710  -0.069706  -1.158397
H  -3.644937   1.497847  -0.347341
S  -1.565673   1.359938  -1.497060
H  -0.918578   0.195741  -1.714160
28
Dimer 103 of the n...n  type
C   3.106829   0.861876  -0.227892
C   3.428445  -0.639677  -0.304517
N   3.360646   1.391730   1.102911
H   2.738644   0.939487   1.768899
H   3.132041   2.380580   1.131454
H   3.776700   1.361320  -0.938281
C   1.682569   1.131145  -0.728464
O   0.861579   1.787180  -0.128548
O   1.444203   0.540818  -1.908663
H   0.492807   0.591606  -2.098846
H   3.406103  -0.982342  -1.336614
H   4.425214  -0.806118   0.102998
S   2.297487  -1.711430   0.691491
H   1.212812  -1.559537  -0.098262
C  -2.097251  -0.707563   0.986380
C  -3.360965   0.172450   0.930291
N  -0.984929  -0.001794   1.592284
H  -0.657339   0.736019   0.974451
H  -0.195861  -0.625312   1.726182
H  -2.353125  -1.573075   1.607992
C  -1.798086  -1.262960  -0.410062
O  -0.781529  -1.059210  -1.036308
O  -2.816863  -1.999484  -0.882670
H  -2.594431  -2.318653  -1.772277
H  -4.218078  -0.406707   0.591464
H  -3.561478   0.548490   1.933178
S  -3.199744   1.677373  -0.126875
H  -3.226293   1.043545  -1.317471
28
Dimer 104 of the n...n  type
C  -1.654586  -0.585819  -0.260549
C  -1.178318   0.401729   0.815176
N  -1.173970  -0.207295  -1.580054
H  -1.520311   0.717500  -1.821502
H  -1.539369  -0.844350  -2.281211
H  -1.207884  -1.549312   0.008128
C  -3.171378  -0.772187  -0.194425
O  -3.932554  -0.599728  -1.117438
O  -3.570489  -1.160052   1.033026
H  -4.534890  -1.268617   1.032817
H  -1.464482   0.054311   1.805377
H  -0.096472   0.463365   0.762890
S  -1.760504   2.136785   0.586720
```



```
H  -3.050596   1.923888   0.919882
C   2.701443  -0.937309  -0.608401
C   3.304100   0.473344  -0.690072
N   1.751254  -1.180616  -1.678148
H   0.897498  -0.628246  -1.554247
H   1.451536  -2.150409  -1.654232
H   3.544843  -1.631423  -0.722787
C   2.141049  -1.235481   0.788448
O   1.081204  -1.775378   1.003730
O   2.979239  -0.840173   1.767330
H   2.592222  -1.072787   2.626644
H   4.147724   0.574608  -0.010413
H   3.652455   0.638227  -1.709242
S   2.114223   1.845174  -0.348158
H   2.114183   1.714266   0.994078
28
Dimer 105 of the n...n  type
C  -3.158745  -0.538295  -0.488303
C  -3.125631   0.982707  -0.715347
N  -3.978198  -0.884058   0.661824
H  -3.556562  -0.501677   1.504779
H  -3.994307  -1.891583   0.786066
H  -3.609906  -0.975894  -1.386532
C  -1.733562  -1.095591  -0.418495
O  -1.294586  -1.743265   0.506079
O  -1.010248  -0.765001  -1.498740
H  -0.109471  -1.142897  -1.413186
H  -2.635207   1.221024  -1.656941
H  -4.149791   1.353657  -0.745398
S  -2.299888   1.932421   0.638299
H  -1.037898   1.606256   0.303763
C   1.583185  -0.002806   0.814202
C   1.601498   1.150620  -0.202789
N   2.232309   0.382751   2.054839
H   3.216445   0.572071   1.882075
H   2.198606  -0.382248   2.721220
H   0.532606  -0.230523   1.029110
C   2.148960  -1.292320   0.209617
O   2.958192  -2.011839   0.732470
O   1.621395  -1.554033  -1.018332
H   1.985275  -2.388454  -1.357565
H   0.947426   0.946618  -1.048006
H   1.252842   2.053076   0.299393
S   3.279841   1.575928  -0.841510
H   3.409656   0.503348  -1.649153
28
Dimer 106 of the n...n  type
C   1.848766  -0.172267   0.881311
C   1.417445   1.067340   0.078347
N   2.662889   0.201883   2.024091
H   3.541056   0.603239   1.705186
H   2.902132  -0.620343   2.568903
H   0.927740  -0.647372   1.236744
C   2.518403  -1.204698  -0.027952
O   3.607498  -1.687259   0.138223
O   1.726504  -1.536637  -1.085606
H   2.174683  -2.200837  -1.634654
H   0.670978   0.813480  -0.671581
H   0.987291   1.784946   0.776839
S   2.798089   1.969058  -0.748638
H   3.003446   1.070924  -1.734275
C  -3.146408  -0.756517  -0.385773
C  -3.232392   0.721671  -0.807846
N  -3.965354  -1.023445   0.784562
H  -3.602033  -0.505067   1.580235
H  -3.908286  -2.006006   1.033312
H  -3.535914  -1.338089  -1.228842
C  -1.680204  -1.170574  -0.227755
O  -1.182966  -1.554396   0.808303
```

```
O  -1.001033  -1.040915  -1.375377
H  -0.067465  -1.313850  -1.247669
H  -2.690775   0.885359  -1.737698
H  -4.280451   0.979544  -0.959110
S  -2.625887   1.921251   0.459201
H  -1.314054   1.643981   0.336694
28
Dimer 107 of the n...n  type
C  -3.154172   0.939437   0.744346
C  -4.184497  -0.183516   0.944742
N  -3.711749   2.040654  -0.025479
H  -3.952043   1.712171  -0.957667
H  -3.009257   2.762377  -0.154174
H  -2.898890   1.307257   1.745641
C  -1.847366   0.381473   0.164817
O  -1.288193   0.833418  -0.810016
O  -1.394444  -0.663883   0.868846
H  -0.548726  -0.988131   0.475904
H  -3.811745  -0.924034   1.649125
H  -5.103510   0.249900   1.338940
S  -4.678881  -1.044571  -0.613692
H  -3.517859  -1.706417  -0.797269
C   3.288392   0.855296  -0.399598
C   1.851984   0.431455  -0.061068
N   3.556466   0.739648  -1.823323
H   3.523967  -0.237665  -2.102011
H   4.497210   1.061689  -2.027846
H   3.369738   1.910196  -0.112883
C   4.295373   0.105071   0.480915
O   5.223979  -0.546858   0.066822
O   4.017772   0.257958   1.790244
H   4.675880  -0.227002   2.313489
H   1.620103   0.644661   0.980568
H   1.151735   0.972086  -0.694801
S   1.490958  -1.353368  -0.385247
H   2.125959  -1.852545   0.695527
28
Dimer 108 of the n...n  type
C   1.467223  -0.458845  -0.190099
C   1.779192   0.890722  -0.866091
N   0.676675  -0.279815   1.017487
H   1.253115   0.109942   1.758313
H   0.308131  -1.161353   1.358741
H   0.878341  -1.029328  -0.913214
C   2.760052  -1.240731   0.046032
O   3.201463  -1.531656   1.132390
O   3.366064  -1.549375  -1.114988
H   4.193532  -2.021002  -0.927468
H   2.343344   0.730995  -1.783221
H   0.842116   1.387281  -1.113610
S   2.677264   2.094596   0.202424
H   3.868120   1.460422   0.200815
C  -3.133636  -0.308136  -0.608920
C  -2.288165   0.900040  -1.062476
N  -4.265400   0.108043   0.200329
H  -3.931037   0.543438   1.055926
H  -4.816587  -0.698331   0.476522
H  -3.509381  -0.779705  -1.523172
C  -2.242313  -1.354369   0.065182
O  -2.260472  -1.641483   1.238302
O  -1.412835  -1.940945  -0.825359
H  -0.916070  -2.645655  -0.380096
H  -1.453698   0.560110  -1.676526
H  -2.919407   1.549071  -1.669008
S  -1.647442   1.959827   0.305371
H  -0.704478   1.061360   0.742035
28
Dimer 109 of the n...n  type
C   2.553747  -0.953890   0.391017
```



```
C   1.305152  -0.120225   0.710702
N   3.507467  -0.928122   1.486722
H   3.878040   0.012235   1.597855
H   4.301866  -1.522801   1.272369
H   2.204812  -1.984214   0.253951
C   3.151253  -0.534085  -0.958442
O   4.303139  -0.216340  -1.133370
O   2.227559  -0.561413  -1.937916
H   2.641411  -0.293802  -2.774106
H   0.551520  -0.245472  -0.063261
H   0.904497  -0.441570   1.671957
S   1.624028   1.691960   0.897089
H   1.694317   1.969789  -0.420971
C  -3.356072   0.568218  -0.265015
C  -3.502837  -0.529030   0.805288
N  -3.797964   0.098820  -1.568178
H  -3.178055  -0.640909  -1.888895
H  -3.722331   0.846817  -2.250486
H  -4.001842   1.395476   0.050100
C  -1.923062   1.110772  -0.256458
O  -1.166277   1.058003  -1.200755
O  -1.592700   1.624023   0.936482
H  -0.643001   1.888483   0.930119
H  -3.293291  -0.128104   1.794961
H  -4.526509  -0.902307   0.783334
S  -2.439386  -2.015423   0.529197
H  -1.275071  -1.457862   0.914621
28
Dimer 110 of the n...n  type
C   2.564202   0.982922  -0.326382
C   1.310990   0.191182  -0.727062
N   3.542218   1.013734  -1.400205
H   3.902512   0.077222  -1.564447
H   4.339199   1.581392  -1.129691
H   2.226688   2.007142  -0.129410
C   3.122764   0.462839   1.004265
O   4.257113   0.085618   1.175977
O   2.185327   0.473360   1.970622
H   2.573163   0.138027   2.794771
H   0.542177   0.277926   0.037406
H   0.937115   0.581488  -1.673414
S   1.609315  -1.609258  -1.026691
H   1.665885  -1.970168   0.271784
C  -3.341892  -0.578959   0.296869
C  -3.542570   0.543195  -0.737001
N  -3.722425  -0.142004   1.630885
H  -3.096604   0.600525   1.933660
H  -3.598019  -0.901567   2.293056
H  -3.999726  -1.401733  -0.005684
C  -1.909927  -1.118962   0.211586
O  -1.131077  -1.137222   1.138873
O  -1.608394  -1.543192  -1.023485
H  -0.659097  -1.807278  -1.056976
H  -3.406825   0.162467  -1.747102
H  -4.555533   0.932535  -0.637513
S  -2.430627   2.001317  -0.501088
H  -1.313631   1.425153  -0.986266
28
Dimer 111 of the n...n  type
C   2.589181  -0.197113  -1.072720
C   3.535232  -1.083397  -0.244185
N   3.314614   0.840133  -1.783863
H   3.756049   1.468097  -1.116967
H   2.673538   1.405702  -2.331114
H   2.109099  -0.857632  -1.804906
C   1.452321   0.333634  -0.198101
O   1.160291   1.507622  -0.090903
O   0.801672  -0.653975   0.437176
H   0.092394  -0.279137   0.990940
```

```
H   2.999715  -1.936742   0.166566
H   4.329625  -1.444879  -0.896522
S   4.395678  -0.199705   1.130817
H   3.322893  -0.084569   1.940666
C  -3.472283   0.296373   0.708662
C  -2.968336  -1.152772   0.841046
N  -4.661363   0.364341  -0.123645
H  -4.427790   0.090745  -1.074742
H  -4.997592   1.320747  -0.175853
H  -3.730666   0.625099   1.721679
C  -2.340295   1.214579   0.240482
O  -2.352313   1.867120  -0.774488
O  -1.297321   1.206509   1.100699
H  -0.557479   1.733671   0.724507
H  -2.167223  -1.219033   1.575315
H  -3.797384  -1.771873   1.182874
S  -2.410869  -1.928051  -0.741159
H  -1.208434  -1.323441  -0.815395
28
Dimer 112 of the n...n  type
C  -3.154657  -0.521437  -0.226067
C  -3.016322   0.840998  -0.940851
N  -3.875814  -0.397735   1.028614
H  -3.335418   0.161821   1.682649
H  -4.000142  -1.310733   1.454577
H  -3.729226  -1.161571  -0.902613
C  -1.770726  -1.155300  -0.094007
O  -1.122915  -1.189525   0.930779
O  -1.328233  -1.632218  -1.266925
H  -0.421405  -1.984550  -1.161957
H  -2.466547   0.721616  -1.873996
H  -4.016957   1.206946  -1.171452
S  -2.253359   2.195480   0.054242
H  -0.981830   1.739329   0.119780
C   1.366585   0.187522  -0.148337
C   2.504393   0.824963  -0.969671
N   0.981457   1.014484   0.980998
H   1.798466   1.249279   1.537738
H   0.341281   0.504011   1.581428
H   0.523562   0.112465  -0.838203
C   1.761028  -1.233850   0.242914
O   2.313687  -1.549887   1.264779
O   1.445641  -2.124238  -0.736410
H   1.757270  -3.007861  -0.479821
H   2.728588   0.206688  -1.838599
H   2.165749   1.801420  -1.317872
S   4.061018   1.170897  -0.043800
H   4.367319  -0.108577   0.252328
28
Dimer 113 of the n...n  type
C  -1.939045   0.775967   0.469174
C  -3.209169   0.099477   1.011829
N  -2.241372   1.789348  -0.526101
H  -2.640734   1.350134  -1.351252
H  -1.373092   2.228412  -0.817925
H  -1.451614   1.260476   1.320283
C  -0.953880  -0.285905  -0.018604
O  -0.641582  -0.458211  -1.175717
O  -0.463819  -1.012342   1.001380
H   0.248146  -1.599065   0.665934
H  -2.957373  -0.587869   1.818206
H  -3.875645   0.869278   1.400741
S  -4.210008  -0.800438  -0.253611
H  -3.344616  -1.814259  -0.461330
C   2.857879   0.700608   0.552028
C   3.482560  -0.558926  -0.060296
N   2.421744   0.482742   1.925334
H   1.610822  -0.129618   1.940551
H   2.112084   1.361627   2.328510
```



```
H   3.660370   1.449893   0.570132
C   1.770013   1.333823  -0.327080
O   0.835601   1.963567   0.115374
O   1.968291   1.132017  -1.638284
H   1.230369   1.524770  -2.132303
H   3.955257  -0.326913  -1.012359
H   4.241897  -0.933971   0.624871
S   2.336280  -1.981614  -0.340116
H   1.663096  -1.435196  -1.373397
28
Dimer 114 of the n...n  type
C   2.985029  -1.006295   0.317002
C   3.063431   0.353391   1.038172
N   4.009581  -1.120481  -0.707514
H   3.825704  -0.449460  -1.448858
H   3.974906  -2.040042  -1.136158
H   3.165117  -1.769515   1.081542
C   1.564430  -1.244468  -0.198163
O   1.249791  -1.282037  -1.366588
O   0.688593  -1.395705   0.808898
H  -0.205160  -1.570243   0.451175
H   2.326266   0.404595   1.838556
H   4.058481   0.452822   1.471314
S   2.855727   1.824650  -0.059307
H   1.503543   1.806853  -0.098292
C  -2.910788   0.139316  -0.874022
C  -1.503340   0.750304  -0.926670
N  -3.933227   1.171056  -0.832247
H  -3.847019   1.705590   0.028568
H  -4.856652   0.749833  -0.816337
H  -3.030766  -0.434039  -1.801837
C  -3.054029  -0.895438   0.246405
O  -3.960284  -0.940676   1.036432
O  -2.050882  -1.818759   0.235745
H  -2.254050  -2.499155   0.900183
H  -0.751979  -0.003655  -1.148898
H  -1.485231   1.487708  -1.728690
S  -1.015019   1.681843   0.590613
H  -0.695102   0.617354   1.352926
28
Dimer 115 of the n...n  type
C   3.001558  -0.439677  -0.351139
C   2.793322   0.934617  -1.008108
N   2.391173  -1.504018  -1.128493
H   1.378638  -1.400935  -1.132788
H   2.577412  -2.401975  -0.692871
H   4.085546  -0.603649  -0.321086
C   2.550723  -0.413916   1.114722
O   1.779724  -1.199447   1.613001
O   3.119713   0.600132   1.794716
H   2.814401   0.574669   2.715794
H   3.383276   1.695425  -0.501067
H   3.111711   0.874789  -2.048505
S   1.034777   1.491961  -1.067352
H   0.881174   1.708475   0.253791
C  -2.567326  -0.241772   0.969118
C  -1.331482  -1.065023   0.559055
N  -3.795314  -0.994880   0.774454
H  -3.944153  -1.155700  -0.218191
H  -4.592411  -0.460383   1.105400
H  -2.450401  -0.026635   2.035874
C  -2.550123   1.106230   0.244488
O  -3.262202   1.402103  -0.685533
O  -1.619630   1.935800   0.760201
H  -1.632459   2.771441   0.265943
H  -0.414452  -0.513425   0.751137
H  -1.312059  -1.976752   1.155409
S  -1.321997  -1.637674  -1.193629
H  -0.982578  -0.453097  -1.742748
```

```
28
Dimer 116 of the n...n  type
C   2.018260   0.662891  -0.630055
C   1.258469  -0.660646  -0.796661
N   3.142474   0.754248  -1.547501
H   3.817816   0.025099  -1.331496
H   3.626712   1.637118  -1.418134
H   1.300849   1.456148  -0.869565
C   2.402199   0.894022   0.834084
O   3.506880   1.185465   1.225120
O   1.342039   0.744531   1.655917
H   1.617566   0.918182   2.570151
H   0.337972  -0.656616  -0.222131
H   1.013018  -0.790232  -1.850464
S   2.225958  -2.160698  -0.323968
H   2.135758  -1.983903   1.010334
C  -2.789051   0.306208   0.858574
C  -3.023805   1.250193  -0.331135
N  -1.809476   0.847030   1.784483
H  -0.899486   0.914689   1.336556
H  -1.685483   0.212508   2.567201
H  -3.752388   0.219520   1.376292
C  -2.460374  -1.113288   0.378609
O  -1.518849  -1.768455   0.759490
O  -3.356704  -1.558620  -0.521981
H  -3.118659  -2.462097  -0.785326
H  -3.860882   0.907520  -0.935777
H  -3.249318   2.244575   0.053339
S  -1.553953   1.475483  -1.425796
H  -1.587677   0.252340  -1.992492
28
Dimer 117 of the n...n  type
C   2.551603  -0.954712   0.387990
C   1.303236  -0.123653   0.715143
N   3.510197  -0.929910   1.479512
H   3.880150   0.010688   1.590691
H   4.304322  -1.523211   1.260399
H   2.203580  -1.985250   0.250245
C   3.142524  -0.530915  -0.963065
O   4.292789  -0.209546  -1.142002
O   2.215077  -0.559301  -1.938955
H   2.624830  -0.288822  -2.776239
H   0.545877  -0.248808  -0.055140
H   0.907735  -0.447590   1.677557
S   1.620238   1.688646   0.903979
H   1.678722   1.969953  -0.413909
C  -3.352055   0.565672  -0.272860
C  -3.507377  -0.532844   0.794596
N  -3.780546   0.096225  -1.580683
H  -3.154871  -0.640841  -1.896449
H  -3.700226   0.845172  -2.261444
H  -4.002505   1.391602   0.036252
C  -1.920489   1.111629  -0.251636
O  -1.159832   1.071593  -1.193404
O  -1.596271   1.612939   0.948076
H  -0.647512   1.880782   0.947240
H  -3.314489  -0.131863   1.787596
H  -4.528380  -0.912114   0.757614
S  -2.429141  -2.011464   0.532705
H  -1.281093  -1.455369   0.966111
28
Dimer 118 of the n...n  type
C   2.744672   0.849971   0.191546
C   3.243949  -0.581681   0.442514
N   2.207796   1.446001   1.402090
H   1.366863   0.954506   1.692128
H   1.957030   2.416051   1.244022
H   3.621537   1.430323  -0.122337
C   1.787381   0.912506  -1.005037
```



```
O   0.750308   1.539775  -1.028076
O   2.232988   0.201780  -2.051486
H   1.615550   0.299359  -2.794611
H   3.746387  -0.974188  -0.438908
H   3.951938  -0.559112   1.270383
S   1.928860  -1.767279   0.961984
H   1.288582  -1.841718  -0.225168
C  -2.768328  -0.165061   0.855868
C  -1.775183  -1.303634   0.550112
N  -4.070826  -0.415360   0.262560
H  -3.987619  -0.414581  -0.750749
H  -4.712438   0.335431   0.498072
H  -2.871878  -0.130734   1.945521
C  -2.124820   1.153039   0.420583
O  -2.513248   1.838291  -0.496189
O  -1.021669   1.424038   1.140228
H  -0.493219   2.083127   0.656438
H  -0.853524  -1.155156   1.109490
H  -2.223777  -2.252524   0.843426
S  -1.356121  -1.503029  -1.238861
H  -0.761088  -0.302247  -1.399563
28
Dimer 119 of the n...n  type
C   2.536627  -0.948308  -0.442973
C   3.523451   0.167879  -0.831165
N   1.375426  -0.967210  -1.312583
H   0.810725  -0.127353  -1.174910
H   0.773088  -1.744839  -1.060882
H   3.084823  -1.890562  -0.560111
C   2.203356  -0.847545   1.050697
O   1.100048  -0.670984   1.513273
O   3.308579  -0.971695   1.811140
H   3.060325  -0.891587   2.746098
H   4.448782   0.072505  -0.265793
H   3.747722   0.074876  -1.893679
S   2.873329   1.883519  -0.625512
H   2.869721   1.895705   0.723468
C  -2.189483   0.887057  -0.596573
C  -2.471427  -0.402033  -1.390670
N  -0.778664   1.023638  -0.292987
H  -0.499752   0.378409   0.442521
H  -0.564654   1.957162   0.039668
H  -2.494590   1.715409  -1.245592
C  -3.095028   0.957836   0.637141
O  -2.706761   1.035623   1.778418
O  -4.398420   0.905546   0.297664
H  -4.937914   0.941281   1.103778
H  -3.507663  -0.429259  -1.722939
H  -1.819003  -0.416716  -2.263639
S  -2.093098  -1.965587  -0.486613
H  -3.105185  -1.894185   0.402318
28
Dimer 120 of the n...n  type
C   2.582471   0.920275  -0.576499
C   1.940445  -0.322669  -1.225220
N   4.012468   0.754022  -0.388218
H   4.191604   0.044649   0.316955
H   4.423013   1.616844  -0.045463
H   2.408840   1.747779  -1.270608
C   1.803980   1.230144   0.703705
O   2.178352   0.956292   1.819541
O   0.614217   1.788830   0.424429
H   0.056053   1.772395   1.219076
H   0.875121  -0.152731  -1.379727
H   2.410431  -0.500703  -2.191871
S   2.174474  -1.902578  -0.290415
H   1.685901  -1.464592   0.889861
C  -2.890494  -0.796499   0.383430
C  -3.205106  -0.062962  -0.934013
```

```
N  -3.370805  -0.057255   1.538004
H  -2.876361   0.827855   1.610307
H  -3.170410  -0.571082   2.390327
H  -3.419859  -1.754143   0.333123
C  -1.397871  -1.137552   0.436424
O  -0.615460  -0.666258   1.233460
O  -1.055485  -2.005915  -0.525100
H  -0.090129  -2.194060  -0.482287
H  -2.892880  -0.666940  -1.784376
H  -4.281126   0.100437  -0.993592
S  -2.463035   1.621425  -1.079801
H  -1.168425   1.247362  -1.082380
28
Dimer 121 of the n...n  type
C  -1.463643  -0.027120   0.735862
C  -1.283558  -0.870911  -0.541487
N  -1.947189  -0.837500   1.836814
H  -2.879887  -1.183495   1.628214
H  -2.022987  -0.279710   2.681311
H  -0.479555   0.369948   1.003160
C  -2.328594   1.199638   0.438903
O  -3.420611   1.417093   0.894635
O  -1.710533   2.041030  -0.431928
H  -2.278589   2.807105  -0.614014
H  -0.815467  -0.288430  -1.330851
H  -0.639776  -1.716751  -0.301198
S  -2.835524  -1.623693  -1.192952
H  -3.378945  -0.488690  -1.679687
C   2.945937   0.404242  -0.712629
C   2.459937  -1.059576  -0.791649
N   4.130069   0.534653   0.118060
H   3.900616   0.323078   1.085099
H   4.469421   1.491247   0.098089
H   3.194870   0.698256  -1.736267
C   1.762379   1.263526  -0.268538
O   1.578037   1.639089   0.869311
O   0.903655   1.483145  -1.273214
H   0.092454   1.919645  -0.939984
H   1.584453  -1.128935  -1.437133
H   3.256328  -1.667144  -1.221762
S   2.096734  -1.866946   0.828851
H   1.178411  -0.987621   1.273319
28
Dimer 122 of the n...n  type
C   3.200824   0.529294   0.319412
C   3.144378  -0.278075  -0.993474
N   3.615933  -0.300986   1.436866
H   2.903740  -1.001303   1.626227
H   3.703064   0.260341   2.278127
H   3.953520   1.309528   0.163361
C   1.879851   1.263808   0.553179
O   1.097779   1.029021   1.444446
O   1.681691   2.224045  -0.372488
H   0.858081   2.696524  -0.173904
H   2.831477   0.358292  -1.819998
H   4.147372  -0.649951  -1.201745
S   2.072130  -1.780581  -0.945075
H   0.874807  -1.229539  -1.263634
C  -1.398457  -0.558493   0.061112
C  -1.426579   0.910595  -0.381988
N  -1.284660  -1.442890  -1.086889
H  -2.075845  -1.306540  -1.710790
H  -1.312862  -2.413433  -0.789707
H  -0.500806  -0.672179   0.680231
C  -2.574494  -0.890287   0.980918
O  -3.309365  -1.839832   0.847977
O  -2.686805   0.004248   1.981741
H  -3.428487  -0.250624   2.553614
H  -1.311940   1.565848   0.477389
```



```
H  -0.596797   1.072185  -1.069444
S  -2.934655   1.398315  -1.327954
H  -3.783385   1.383338  -0.279453
28
Dimer 123 of the n...n  type
C  -2.079933  -0.049001  -0.959535
C  -1.866077  -1.429807  -0.308464
N  -0.838583   0.703769  -1.051822
H  -0.584515   1.061861  -0.134925
H  -0.958040   1.509437  -1.657171
H  -2.455503  -0.240792  -1.969723
C  -3.185728   0.707997  -0.218277
O  -3.031170   1.749328   0.374616
O  -4.361089   0.055218  -0.285378
H  -5.029038   0.550457   0.215323
H  -2.803278  -1.983040  -0.281939
H  -1.150220  -1.995574  -0.901704
S  -1.146951  -1.378594   1.388024
H  -2.244358  -0.908529   2.016975
C   3.007391   0.248096   0.551262
C   3.178510  -0.590536  -0.725582
N   2.657905  -0.571898   1.699505
H   1.747171  -0.997457   1.545492
H   2.560354   0.013210   2.523480
H   3.984043   0.712755   0.739722
C   2.056800   1.432561   0.336352
O   1.185030   1.760930   1.108336
O   2.320416   2.098300  -0.802546
H   1.723735   2.860811  -0.870339
H   3.494585   0.045288  -1.550220
H   3.950371  -1.338908  -0.546317
S   1.688407  -1.553736  -1.235125
H   0.794866  -0.521402  -1.323580
28
Dimer 124 of the n...n  type
C   1.680134   0.296467   0.065404
C   2.941664   1.077813  -0.343348
N   1.437283   0.346774   1.496209
H   2.223425  -0.067514   1.989745
H   0.621927  -0.233451   1.684751
H   0.848689   0.777451  -0.452762
C   1.766121  -1.126345  -0.484623
O   2.189266  -2.080712   0.125608
O   1.346108  -1.191209  -1.764486
H   1.462908  -2.098373  -2.089843
H   3.054804   1.066575  -1.427359
H   2.826538   2.111870  -0.016654
S   4.516532   0.513732   0.437576
H   4.528476  -0.727617  -0.089532
C  -2.201450  -0.233257   0.879473
C  -1.892729   1.253688   0.627592
N  -1.123115  -1.088464   0.412556
H  -1.052810  -1.034751  -0.600568
H  -1.317785  -2.058818   0.636549
H  -2.292937  -0.347214   1.965545
C  -3.569453  -0.603116   0.297172
O  -3.758527  -1.470435  -0.522047
O  -4.548606   0.167950   0.806830
H  -5.396265  -0.090740   0.410904
H  -2.657990   1.881393   1.079677
H  -0.930261   1.495009   1.078293
S  -1.720939   1.708874  -1.153698
H  -3.019010   1.560051  -1.489424
28
Dimer 125 of the n...n  type
C   1.625261  -0.057294  -0.398809
C   2.378787   1.260170  -0.162224
N   0.698494  -0.338137   0.685279
H   1.201874  -0.435140   1.562792
```

```
H   0.214286  -1.214001   0.520302
H   1.046664   0.085354  -1.320043
C   2.587363  -1.211671  -0.688588
O   2.564747  -2.283031  -0.131394
O   3.465755  -0.892779  -1.657596
H   4.046486  -1.652673  -1.823105
H   2.963100   1.528301  -1.039662
H   1.650843   2.048635   0.026852
S   3.485301   1.245527   1.314772
H   4.435014   0.443436   0.790557
C  -3.305864  -0.363935  -0.567824
C  -2.127418   0.249162  -1.349032
N  -4.420321   0.565384  -0.485939
H  -4.149271   1.372427   0.069962
H  -5.203832   0.133356  -0.006367
H  -3.624314  -1.246622  -1.132458
C  -2.833223  -0.877067   0.794920
O  -3.137943  -0.400012   1.861441
O  -2.024803  -1.952436   0.669096
H  -1.779428  -2.263017   1.555720
H  -1.324824  -0.480602  -1.453526
H  -2.482714   0.517501  -2.343503
S  -1.434550   1.795470  -0.614509
H  -0.644609   1.146243   0.289843
28
Dimer 126 of the n...n  type
C  -3.120104   0.433001  -0.137634
C  -3.213472  -1.100790  -0.170865
N  -2.932959   0.988221  -1.467966
H  -2.098325   0.590062  -1.890253
H  -2.777925   1.989863  -1.410708
H  -4.076758   0.793349   0.261021
C  -2.066980   0.895686   0.875946
O  -1.169186   1.668105   0.634463
O  -2.269715   0.351753   2.088929
H  -1.589436   0.675347   2.699998
H  -3.460008  -1.482481   0.817951
H  -4.002353  -1.388553  -0.865623
S  -1.682770  -1.939409  -0.777856
H  -0.847795  -1.496288   0.212393
C   1.633355  -0.753227  -0.059156
C   1.292924   0.386364  -1.031804
N   0.788705  -0.703089   1.125957
H   0.753753   0.247037   1.485795
H   1.168580  -1.290158   1.861911
H   1.429443  -1.690666  -0.594573
C   3.128786  -0.797698   0.258812
O   3.597708  -0.884126   1.368572
O   3.871591  -0.753442  -0.863146
H   4.810855  -0.807577  -0.624912
H   1.724016   0.192085  -2.010976
H   0.211162   0.450522  -1.125445
S   1.836100   2.057612  -0.465852
H   3.141047   1.891943  -0.765525
28
Dimer 127 of the n...n  type
C   2.316951  -0.121759   1.145082
C   2.130199  -1.534462   0.568777
N   1.137925   0.322562   1.872063
H   0.288623   0.040774   1.392412
H   1.115563   1.336358   1.919956
H   3.164557  -0.181936   1.841265
C   2.780150   0.856009   0.061399
O   2.291719   1.939882  -0.151975
O   3.837219   0.371149  -0.619676
H   4.117173   1.026613  -1.278402
H   3.091345  -1.981961   0.327289
H   1.632218  -2.154271   1.314215
S   1.066899  -1.613968  -0.941662
```



```
H   2.010773  -1.204537  -1.812814
C  -3.091348   0.159596   0.077624
C  -2.558734   1.066322  -1.050933
N  -3.236988   0.891067   1.324442
H  -2.324046   1.194294   1.653156
H  -3.618771   0.285887   2.044452
H  -4.078396  -0.187149  -0.245988
C  -2.211982  -1.086779   0.189967
O  -1.472422  -1.331647   1.116472
O  -2.338404  -1.880163  -0.886901
H  -1.717891  -2.622797  -0.808947
H  -2.494992   0.508907  -1.984415
H  -3.257614   1.892193  -1.183723
S  -0.930305   1.861274  -0.701341
H  -0.156920   0.767980  -0.880801
28
Dimer 128 of the n...n  type
C  -2.973996   0.398384  -0.532836
C  -3.138523  -0.508194   0.696879
N  -2.922149  -0.375922  -1.763113
H  -2.113973  -0.993285  -1.742711
H  -2.786471   0.242627  -2.556738
H  -3.862389   1.041746  -0.562506
C  -1.787059   1.356455  -0.366760
O  -0.943297   1.550408  -1.210799
O  -1.794154   1.969523   0.828338
H  -0.985268   2.500572   0.907626
H  -3.305573   0.087224   1.591786
H  -3.999442  -1.157509   0.539266
S  -1.716546  -1.650461   0.996636
H  -0.835354  -0.704742   1.380387
C   2.091123   0.638276  -0.722795
C   1.338177  -0.666515  -1.021812
N   3.414083   0.635135  -1.327062
H   3.978006  -0.100024  -0.907482
H   3.892004   1.508209  -1.126895
H   1.492300   1.440086  -1.167623
C   2.114788   0.929843   0.780147
O   3.104844   1.179485   1.422939
O   0.876881   0.884932   1.326871
H   0.947806   1.070877   2.277372
H   0.289707  -0.576145  -0.759575
H   1.409780  -0.866808  -2.090557
S   2.030486  -2.162223  -0.192596
H   1.591045  -1.868640   1.048511
28
Dimer 129 of the n...n  type
C   2.217794   0.648093   0.968741
C   3.444939  -0.238866   0.702437
N   1.166111  -0.088478   1.646358
H   0.938306  -0.925039   1.115967
H   0.319066   0.468406   1.694870
H   2.559521   1.461242   1.621032
C   1.760977   1.337867  -0.321912
O   0.634852   1.302491  -0.766684
O   2.762335   2.013094  -0.907775
H   2.429558   2.444999  -1.711208
H   4.272896   0.356618   0.323945
H   3.745985  -0.707443   1.639287
S   3.129626  -1.639263  -0.460818
H   2.987709  -0.891403  -1.574609
C  -3.194956  -0.196049   0.532201
C  -2.151485  -1.292489   0.811850
N  -4.211297  -0.660065  -0.397194
H  -3.781583  -0.861870  -1.296452
H  -4.892853   0.073575  -0.564414
H  -3.671078   0.030853   1.492754
C  -2.492796   1.089947   0.085717
O  -2.654202   1.629793  -0.982019
```

```
O  -1.649911   1.550918   1.031460
H  -1.140204   2.286072   0.653516
H  -1.492285  -0.990820   1.623277
H  -2.674904  -2.199732   1.112824
S  -1.135145  -1.782111  -0.650717
H  -0.446953  -0.625055  -0.764418
28
Dimer 130 of the n...n  type
C  -1.810386   0.500769  -0.150214
C  -1.663259  -0.840981  -0.895356
N  -1.085371   0.496120   1.108656
H  -1.576103  -0.074972   1.790709
H  -1.030770   1.434191   1.491238
H  -1.366800   1.253835  -0.807684
C  -3.291881   0.848369  -0.002761
O  -3.895488   0.873441   1.044330
O  -3.861834   1.096143  -1.197331
H  -4.806164   1.279721  -1.067773
H  -2.203693  -0.809410  -1.840263
H  -0.606675  -0.998921  -1.103339
S  -2.196750  -2.321167   0.065111
H  -3.511060  -2.022290   0.126748
C   2.055613   0.102214   0.923132
C   3.593519   0.153239   0.837970
N   1.550193  -1.257634   0.861812
H   1.735631  -1.654102  -0.055430
H   0.541170  -1.216857   0.977146
H   1.782495   0.531006   1.890120
C   1.476730   1.025037  -0.147571
O   1.125717   0.671614  -1.251006
O   1.467660   2.311930   0.251173
H   1.159660   2.867642  -0.482703
H   3.942524   1.181122   0.937008
H   4.004525  -0.435402   1.658586
S   4.325881  -0.592390  -0.684194
H   3.764970   0.255038  -1.570834
28
Dimer 131 of the n...n  type
C   2.604828  -0.758838   0.837741
C   2.518548   0.782724   0.832543
N   3.834012  -1.243900   0.230964
H   3.848365  -1.015945  -0.759008
H   3.885532  -2.255304   0.303183
H   2.586399  -1.062208   1.887765
C   1.324441  -1.281890   0.187490
O   1.208657  -1.495064  -1.004425
O   0.325280  -1.400691   1.062258
H  -0.515632  -1.598237   0.586375
H   1.564128   1.098854   1.250619
H   3.329925   1.181274   1.442142
S   2.743495   1.587883  -0.818505
H   1.898234   0.801551  -1.513319
C  -2.148831   1.030677  -0.774920
C  -3.137556  -0.120815  -0.989144
N  -0.952107   0.868249  -1.584052
H  -0.515446  -0.032227  -1.407034
H  -0.268279   1.576046  -1.333383
H  -2.667667   1.940006  -1.107105
C  -1.855934   1.284068   0.709019
O  -0.766686   1.556847   1.153067
O  -2.970143   1.211415   1.461812
H  -2.745737   1.420480   2.382808
H  -4.119201   0.132961  -0.596370
H  -3.225524  -0.311062  -2.058156
S  -2.642281  -1.737597  -0.232869
H  -3.057698  -1.446823   1.017008
28
Dimer 132 of the n...n  type
C   3.098480  -0.270075   0.710734
```



```
C   2.037389   0.766933   1.131713
N   4.333590   0.376162   0.301589
H   4.173359   0.922095  -0.540728
H   5.035147  -0.319905   0.069616
H   3.293166  -0.885464   1.594343
C   2.501940  -1.189307  -0.356887
O   2.744472  -1.119006  -1.537009
O   1.627580  -2.080474   0.163078
H   1.198099  -2.543751  -0.575943
H   1.162614   0.272323   1.546481
H   2.470911   1.403570   1.902953
S   1.510830   1.938861  -0.196065
H   1.053915   1.015423  -1.066273
C  -2.006870   0.889750   0.169403
C  -3.341719   0.532596   0.844634
N  -2.211153   1.603882  -1.077456
H  -2.626814   0.983547  -1.767016
H  -1.319641   1.905944  -1.456434
H  -1.468433   1.546491   0.861695
C  -1.130226  -0.357732   0.030646
O  -0.675133  -0.773955  -1.010587
O  -0.903585  -0.935703   1.223864
H  -0.272447  -1.667057   1.110775
H  -3.171015   0.119985   1.837063
H  -3.937204   1.440483   0.936552
S  -4.392197  -0.643175  -0.118151
H  -3.647478  -1.744860   0.109217
28
Dimer 133 of the n...n  type
C  -2.005101   0.888846   0.168563
C  -3.339176   0.533167   0.845687
N  -2.210121   1.604189  -1.077516
H  -2.628064   0.985090  -1.766811
H  -1.318600   1.904695  -1.457713
H  -1.464411   1.544345   0.860306
C  -1.129811  -0.359444   0.027577
O  -0.672913  -0.772684  -1.014001
O  -0.906042  -0.941350   1.219478
H  -0.274531  -1.672250   1.105486
H  -3.167356   0.120420   1.837879
H  -3.933705   1.441599   0.938523
S  -4.391983  -0.641604  -0.115664
H  -3.648058  -1.743976   0.110909
C   3.095832  -0.270476   0.712652
C   2.033692   0.766011   1.132190
N   4.331936   0.376204   0.307133
H   4.173926   0.922491  -0.535360
H   5.034279  -0.319629   0.076870
H   3.288489  -0.886805   1.596046
C   2.501888  -1.188600  -0.357313
O   2.745980  -1.116182  -1.536966
O   1.627636  -2.081457   0.160043
H   1.199923  -2.544217  -0.580307
H   1.157641   0.270665   1.543367
H   2.464881   1.401385   1.905766
S   1.511081   1.939863  -0.195513
H   1.056074   1.017646  -1.068015
28
Dimer 134 of the n...n  type
C   1.897362  -0.317438   0.032497
C   2.003851   1.076999  -0.604653
N   1.530880  -0.234319   1.435225
H   2.293264   0.176262   1.967109
H   1.377851  -1.164277   1.811477
H   1.098908  -0.845419  -0.500729
C   3.167525  -1.134368  -0.223649
O   3.821295  -1.682343   0.631673
O   3.475096  -1.175511  -1.535036
H   4.279149  -1.705515  -1.656050
```

```
H   2.138168   0.998247  -1.681516
H   1.083648   1.624520  -0.405011
S   3.352521   2.130292   0.090483
H   4.379840   1.467370  -0.479641
C  -2.819181   0.885366   0.302766
C  -1.479090   0.166438   0.087067
N  -3.199392   0.888155   1.706000
H  -3.343888  -0.068177   2.020537
H  -4.088374   1.363805   1.825017
H  -2.667366   1.921769  -0.021801
C  -3.897758   0.314731  -0.625042
O  -4.972645  -0.101186  -0.262737
O  -3.503650   0.332235  -1.913673
H  -4.213742  -0.029770  -2.467532
H  -1.136901   0.295317  -0.938132
H  -0.735487   0.578670   0.767463
S  -1.500392  -1.636130   0.483492
H  -2.216557  -2.032168  -0.589102
28
Dimer 135 of the n...n  type
C   1.557859  -0.116110   0.587192
C   2.988225   0.066663   1.116795
N   1.192942  -1.518378   0.497572
H   1.829079  -1.992587  -0.138402
H   0.263307  -1.621897   0.098698
H   0.897466   0.379124   1.308814
C   1.347930   0.662530  -0.715978
O   0.871270   0.207685  -1.728167
O   1.737399   1.947843  -0.594448
H   1.565048   2.411017  -1.429741
H   3.188450   1.114349   1.330848
H   3.094901  -0.508937   2.035987
S   4.304056  -0.569439  -0.013681
H   4.197329   0.406985  -0.938355
C  -2.947190  -0.577282   0.719010
C  -3.136791   0.946051   0.712223
N  -2.109052  -1.009649   1.825705
H  -1.197932  -0.564186   1.766947
H  -1.943530  -2.009653   1.771982
H  -3.946890  -1.011011   0.851518
C  -2.478908  -1.100185  -0.644904
O  -1.614653  -1.932544  -0.797941
O  -3.164128  -0.552552  -1.662604
H  -2.854371  -0.938069  -2.498126
H  -3.878330   1.236766  -0.028458
H  -3.481338   1.259663   1.697405
S  -1.586947   1.903445   0.413742
H  -1.420019   1.537067  -0.873767
28
Dimer 136 of the n...n  type
C  -2.269539  -0.653985  -0.801116
C  -1.776980   0.797401  -0.730508
N  -3.526776  -0.755682  -1.525123
H  -4.249638  -0.245698  -1.022921
H  -3.832844  -1.723174  -1.556986
H  -1.492992  -1.202919  -1.346986
C  -2.316253  -1.292136   0.591614
O  -3.249881  -1.916178   1.035702
O  -1.171886  -1.086667   1.277922
H  -1.233109  -1.524227   2.142005
H  -0.762278   0.830458  -0.351397
H  -1.791294   1.220292  -1.734562
S  -2.820632   1.916923   0.300814
H  -2.413723   1.439146   1.494927
C   2.339917  -0.356292  -1.008883
C   2.119117   1.163194  -0.910931
N   1.091904  -1.089792  -0.894897
H   0.706164  -0.987303   0.040126
H   1.259447  -2.082815  -1.022184
```



```
H   2.763778  -0.538546  -2.003555
C   3.412311  -0.811203  -0.012006
O   3.269241  -1.690833   0.803407
O   4.551295  -0.104238  -0.150394
H   5.203376  -0.423739   0.493687
H   3.028654   1.698620  -1.175163
H   1.329872   1.444664  -1.607898
S   1.544667   1.756617   0.742312
H   2.702511   1.538376   1.398907
28
Dimer 137 of the n...n  type
C  -1.962439   0.262344   0.824682
C  -1.669604  -0.939205  -0.089492
N  -2.807228  -0.116815   1.943532
H  -3.722785  -0.398659   1.602773
H  -2.959525   0.681161   2.552043
H  -0.991292   0.587975   1.212445
C  -2.509810   1.434250   0.006625
O  -3.557793   1.997827   0.204925
O  -1.660443   1.773703  -0.989081
H  -2.022154   2.526326  -1.483745
H  -0.947330  -0.672391  -0.858233
H  -1.253686  -1.739979   0.519736
S  -3.153538  -1.663385  -0.912765
H  -3.356331  -0.657315  -1.788386
C   1.936209   0.066035  -0.801094
C   3.460889   0.020445  -1.025769
N   1.468249   1.421958  -0.588776
H   1.738978   1.743761   0.335665
H   0.456907   1.475755  -0.647978
H   1.481327  -0.325754  -1.717220
C   1.561670  -0.904862   0.323181
O   1.091307  -0.580796   1.388599
O   1.838116  -2.178683  -0.009645
H   1.615094  -2.758994   0.736031
H   3.781933  -0.987910  -1.282236
H   3.704761   0.690523  -1.849669
S   4.464588   0.626653   0.400120
H   4.279165  -0.443251   1.200612
28
Dimer 138 of the n...n  type
C   2.215954  -0.649002   0.556333
C   1.606237   0.758675   0.647477
N   3.307086  -0.821438   1.500608
H   4.059578  -0.176596   1.272252
H   3.696710  -1.755229   1.417687
H   1.418344  -1.353704   0.820111
C   2.596062  -0.982022  -0.890661
O   3.682963  -1.364173  -1.252061
O   1.554853  -0.802317  -1.728800
H   1.824499  -1.037263  -2.630960
H   0.711550   0.843786   0.035657
H   1.340156   0.955433   1.685492
S   2.757240   2.119197   0.163333
H   2.707766   1.896573  -1.166370
C  -2.300143   0.211302  -0.852902
C  -1.564655  -0.990733  -0.235441
N  -1.551687   1.443994  -0.669193
H  -1.559909   1.704039   0.313872
H  -2.002171   2.204486  -1.168071
H  -2.376491   0.004696  -1.926380
C  -3.739334   0.285682  -0.331688
O  -4.222092   1.239877   0.230430
O  -4.412005  -0.857173  -0.568801
H  -5.313792  -0.773264  -0.219699
H  -2.082566  -1.918794  -0.468850
H  -0.562839  -1.034583  -0.658665
S  -1.336127  -0.884560   1.594946
H  -2.600889  -1.204794   1.936703
```

```
28
Dimer 139 of the n...n  type
C  -3.237903   0.123475   0.791363
C  -2.216674  -0.985168   1.108034
N  -4.471552  -0.427177   0.258203
H  -4.289022  -0.871960  -0.637472
H  -5.140117   0.316017   0.081981
H  -3.455297   0.619643   1.743314
C  -2.595243   1.186105  -0.105281
O  -2.913338   1.415550  -1.247269
O  -1.610104   1.844331   0.539676
H  -1.226550   2.504340  -0.060114
H  -1.330637  -0.561250   1.576787
H  -2.675158  -1.686938   1.804581
S  -1.709748  -2.012797  -0.338610
H  -1.023385  -1.048508  -0.993934
C   2.695554   0.311727   0.941758
C   1.248067   0.136324   0.459269
N   3.066616   1.715235   1.009887
H   3.050070   2.117951   0.076300
H   4.021238   1.807770   1.342751
H   2.735557  -0.110972   1.952711
C   3.654169  -0.538873   0.099303
O   4.644185  -0.121216  -0.452313
O   3.258534  -1.825590   0.045352
H   3.892100  -2.330966  -0.488813
H   0.934257  -0.901624   0.552215
H   0.595402   0.763382   1.064402
S   0.948743   0.687013  -1.277500
H   1.600130  -0.326892  -1.883805
28
Dimer 140 of the n...n  type
C  -2.132777   0.115773   0.936443
C  -3.654035  -0.034710   1.104037
N  -1.752445   1.503135   0.740943
H  -2.140617   1.843285  -0.134977
H  -0.744540   1.591910   0.659935
H  -1.682243  -0.243508   1.870132
C  -1.611441  -0.825993  -0.155671
O  -0.890379  -0.489712  -1.067076
O  -2.034302  -2.088898   0.025860
H  -1.674280  -2.652050  -0.678188
H  -3.910658  -1.048079   1.405447
H  -3.987702   0.659941   1.874355
S  -4.625835   0.400992  -0.405423
H  -4.302910  -0.698308  -1.117442
C   1.855318  -0.387516   0.237310
C   3.042882  -0.208541   1.201688
N   1.923802  -1.647501  -0.479301
H   2.758007  -1.663060  -1.060003
H   1.129054  -1.717671  -1.107534
H   0.958548  -0.384632   0.862842
C   1.755314   0.834460  -0.676999
O   2.035836   0.851987  -1.850079
O   1.350056   1.926838   0.008002
H   1.332996   2.690324  -0.591411
H   2.917659   0.700413   1.788259
H   3.068995  -1.063040   1.877982
S   4.705824  -0.186671   0.400789
H   4.577112   1.003343  -0.221249
28
Dimer 141 of the n...n  type
C  -1.641878  -0.341392  -0.603041
C  -2.979570   0.234363  -1.095500
N  -1.671475  -1.790654  -0.529886
H  -2.327196  -2.087352   0.187920
H  -0.760306  -2.150416  -0.260045
H  -0.885711  -0.046668  -1.340991
C  -1.212594   0.332351   0.704731
```



```
O  -0.917705  -0.246871   1.721285
O  -1.163435   1.678492   0.582970
H  -0.777129   2.051662   1.393317
H  -2.895724   1.304578  -1.274632
H  -3.244290  -0.259919  -2.029717
S  -4.401060  -0.073384   0.041700
H  -4.051514   0.829721   0.980962
C   3.093733   0.403993  -0.687971
C   2.531376  -0.999804  -0.993807
N   4.233073   0.347029   0.209521
H   3.941715   0.007278   1.121752
H   4.617072   1.276044   0.350211
H   3.418620   0.817137  -1.647830
C   1.944612   1.285692  -0.194120
O   1.769232   1.613989   0.957439
O   1.105215   1.607035  -1.196026
H   0.344584   2.093014  -0.830630
H   1.697919  -0.921937  -1.691374
H   3.317772  -1.598029  -1.453818
S   2.010724  -1.979248   0.484314
H   1.157856  -1.080012   1.014097
28
Dimer 142 of the n...n  type
C  -2.914843  -0.526383   0.707792
C  -1.518699  -0.700233   0.090068
N  -3.834231  -1.554260   0.248505
H  -3.977566  -1.458427  -0.753657
H  -4.744418  -1.427445   0.680061
H  -2.785863  -0.629197   1.791727
C  -3.425164   0.901397   0.481503
O  -4.467120   1.186268  -0.059772
O  -2.561663   1.817170   0.961435
H  -2.917564   2.705364   0.798638
H  -0.811008  -0.001208   0.530104
H  -1.181633  -1.719812   0.272306
S  -1.459027  -0.487032  -1.741865
H  -1.602184   0.854266  -1.743413
C   2.379668   0.887093   0.576806
C   2.074115  -0.403185   1.355100
N   1.237368   1.315620  -0.211700
H   1.035365   0.627071  -0.933059
H   1.451833   2.180897  -0.696966
H   2.595720   1.656692   1.327592
C   3.667470   0.735891  -0.241780
O   3.768422   0.977830  -1.420893
O   4.691754   0.300197   0.517795
H   5.487384   0.228449  -0.033460
H   2.872471  -0.623496   2.060554
H   1.146553  -0.266731   1.910180
S   1.801877  -1.887444   0.289290
H   3.096928  -2.083534  -0.032502
28
Dimer 143 of the n...n  type
C   1.895990   0.317060  -0.032947
C   2.003612  -1.077114   0.604658
N   1.533031   0.233069  -1.436481
H   2.297720  -0.175386  -1.966662
H   1.378146   1.162623  -1.812931
H   1.095410   0.843814   0.498298
C   3.164119   1.136263   0.226252
O   3.819099   1.685205  -0.627506
O   3.468367   1.178002   1.538407
H   4.271319   1.709219   1.661395
H   2.135661  -0.998012   1.681774
H   1.084802  -1.626333   0.403300
S   3.355512  -2.128084  -0.087715
H   4.380535  -1.463511   0.484614
C  -2.818003  -0.890397  -0.290125
C  -1.478263  -0.166905  -0.088333
```

```
N  -3.202309  -0.913968  -1.692045
H  -3.348499   0.037485  -2.020231
H  -4.091273  -1.391981  -1.801441
H  -2.664046  -1.921868   0.048887
C  -3.894684  -0.307865   0.632514
O  -4.971048   0.101613   0.267283
O  -3.497082  -0.306719   1.920161
H  -4.206139   0.062186   2.470786
H  -1.133456  -0.280884   0.937758
H  -0.735965  -0.587929  -0.764690
S  -1.502304   1.629727  -0.510688
H  -2.221899   2.039529   0.554393
28
Dimer 144 of the n...n  type
C   1.979053  -0.484059   0.597717
C   1.579665   0.993617   0.459128
N   2.963120  -0.674087   1.649200
H   3.823382  -0.190818   1.403305
H   3.200475  -1.657699   1.731187
H   1.066371  -1.019938   0.877065
C   2.404022  -1.057858  -0.757675
O   3.451115  -1.616323  -0.979022
O   1.449881  -0.866861  -1.692766
H   1.740443  -1.252975  -2.534443
H   0.764608   1.105739  -0.250001
H   1.243694   1.354894   1.429267
S   2.956564   2.113234  -0.047745
H   3.035861   1.696940  -1.328643
C  -1.784811  -0.563037  -0.517423
C  -2.128617   0.912121  -0.784331
N  -2.594582  -1.453318  -1.331351
H  -3.572060  -1.368456  -1.064361
H  -2.332631  -2.418600  -1.158032
H  -0.736860  -0.693848  -0.806778
C  -1.850893  -0.867923   0.982363
O  -2.504022  -1.750608   1.484049
O  -1.070030  -0.025161   1.690017
H  -1.123197  -0.253064   2.631988
H  -1.438452   1.571548  -0.262668
H  -2.053071   1.098293  -1.855293
S  -3.852417   1.388917  -0.323940
H  -3.670750   1.359860   1.012538
28
Dimer 145 of the n...n  type
C  -2.056704  -0.439331  -0.653350
C  -3.507010  -0.041188  -0.980831
N  -1.988352  -1.741240  -0.015260
H  -2.439929  -1.700174   0.894517
H  -1.017477  -1.984872   0.150548
H  -1.524629  -0.485959  -1.607983
C  -1.381377   0.672592   0.150900
O  -0.979383   0.564444   1.285265
O  -1.292084   1.815080  -0.558573
H  -0.904926   2.507879   0.000507
H  -3.527600   0.880394  -1.559946
H  -3.959159  -0.838890  -1.569993
S  -4.606480   0.152452   0.490828
H  -4.067212   1.301996   0.946282
C   2.109191  -0.355447   0.880181
C   2.127801   1.089122   0.347755
N   3.210621  -0.602435   1.793998
H   4.093315  -0.550978   1.292404
H   3.147388  -1.541924   2.172941
H   1.164465  -0.463904   1.420799
C   2.046219  -1.333205  -0.294315
O   2.889463  -2.150708  -0.571498
O   0.913120  -1.162195  -1.012402
H   0.917262  -1.774196  -1.766027
H   1.257346   1.277028  -0.274872
```



```
H   2.107914   1.772226   1.196214
S   3.641380   1.541559  -0.608395
H   3.361314   0.814695  -1.709876
28
Dimer 146 of the n...n  type
C  -2.926445  -0.619245   0.459010
C  -3.403915   0.825211   0.235421
N  -2.180624  -0.748094   1.697974
H  -1.327355  -0.197852   1.650896
H  -1.876904  -1.708738   1.822326
H  -3.830602  -1.236232   0.522944
C  -2.165561  -1.122584  -0.772322
O  -1.036469  -1.553184  -0.757600
O  -2.906331  -1.021537  -1.892149
H  -2.388647  -1.348099  -2.645592
H  -4.081684   0.879182  -0.614086
H  -3.929384   1.160318   1.129422
S  -2.042305   2.047847  -0.013083
H  -1.693479   1.655050  -1.255455
C   1.487578   0.399794  -0.278090
C   2.697811   0.202687  -1.197275
N   1.433284   1.755230   0.249490
H   2.228280   1.916176   0.862512
H   0.596103   1.869527   0.812157
H   0.593978   0.233753  -0.890644
C   1.385478  -0.676596   0.809013
O   0.929116  -0.479716   1.912827
O   1.837517  -1.875928   0.404980
H   1.686498  -2.522079   1.113119
H   2.649465  -0.760149  -1.701342
H   2.697270   0.993226  -1.947199
S   4.331331   0.341387  -0.343100
H   4.243105  -0.824451   0.328610
28
Dimer 147 of the n...n  type
C  -3.009376   0.333267  -0.715351
C  -3.412864  -1.093335  -0.313997
N  -2.105634   0.330884  -1.853877
H  -1.301012  -0.255497  -1.652503
H  -1.732356   1.262208  -2.008557
H  -3.935865   0.847714  -1.001803
C  -2.478526   1.124275   0.487221
O  -1.471313   1.792058   0.480671
O  -3.283290   1.007623   1.560051
H  -2.924186   1.544087   2.285021
H  -4.210093  -1.073526   0.425553
H  -3.767662  -1.618666  -1.200603
S  -2.020357  -2.119553   0.337206
H  -1.898159  -1.479799   1.518439
C   2.204475  -0.274702   0.869705
C   1.120079  -0.169952  -0.213554
N   2.167496   0.867641   1.767917
H   2.351237   1.718544   1.242101
H   2.908941   0.793325   2.457501
H   1.974972  -1.180554   1.443934
C   3.580294  -0.520897   0.242016
O   4.565187   0.148196   0.447614
O   3.566796  -1.590499  -0.577574
H   4.454825  -1.717053  -0.948272
H   1.050352  -1.097495  -0.777391
H   0.167178   0.017976   0.272002
S   1.332249   1.236230  -1.388821
H   2.395007   0.731260  -2.050185
28
Dimer 148 of the n...n  type
C   2.825864   0.732205  -0.722135
C   2.113797  -0.533574  -1.241783
N   4.234029   0.485769  -0.459131
H   4.329447  -0.181709   0.301355
```

```
H   4.691087   1.340782  -0.157866
H   2.737105   1.480096  -1.515755
C   2.050990   1.259227   0.486130
O   2.364523   1.072711   1.638730
O   0.933721   1.916301   0.118770
H   0.404681   2.100630   0.913053
H   1.077386  -0.314891  -1.498115
H   2.634417  -0.876436  -2.136506
S   2.157910  -1.986048  -0.099827
H   1.530214  -1.409626   0.945564
C  -2.279248  -0.477593  -0.945832
C  -1.681129  -1.328282   0.186123
N  -1.259796   0.308600  -1.618085
H  -0.855559   0.984959  -0.976262
H  -1.672966   0.843961  -2.374912
H  -2.707401  -1.184250  -1.667081
C  -3.459603   0.355494  -0.433582
O  -3.578695   1.548528  -0.579752
O  -4.365538  -0.409632   0.206809
H  -5.099262   0.150491   0.506856
H  -2.404552  -2.059428   0.540563
H  -0.805820  -1.852434  -0.196903
S  -1.073569  -0.362524   1.640245
H  -2.283976  -0.073708   2.159856
28
Dimer 149 of the n...n  type
C  -1.665153  -0.156310  -0.364993
C  -2.871695  -0.775472  -1.083643
N  -1.083311  -1.081293   0.593193
H  -1.780890  -1.335512   1.287878
H  -0.330368  -0.623060   1.094757
H  -0.916883   0.061506  -1.136212
C  -2.002903   1.214802   0.233070
O  -1.679395   1.584053   1.338829
O  -2.680078   1.988894  -0.634013
H  -2.838715   2.856256  -0.227719
H  -3.193220  -0.145406  -1.909958
H  -2.580666  -1.749688  -1.475939
S  -4.322828  -1.101686   0.012831
H  -4.688022   0.187639   0.167118
C   2.749456   0.219533  -1.010142
C   2.183176  -1.183989  -0.738766
N   4.186117   0.178942  -1.229018
H   4.649121  -0.147533  -0.384317
H   4.538864   1.114894  -1.403189
H   2.265142   0.575946  -1.927602
C   2.325172   1.206976   0.083954
O   3.070987   1.945664   0.679612
O   0.996329   1.156663   0.300928
H   0.735979   1.792014   0.988044
H   1.095975  -1.182180  -0.751089
H   2.540770  -1.856430  -1.518240
S   2.746859  -1.938509   0.848407
H   2.027724  -1.144303   1.667707
28
Dimer 150 of the n...n  type
C   3.142154  -0.669183  -0.626780
C   1.634916  -0.566740  -0.351590
N   3.737496  -1.796914   0.070785
H   3.663095  -1.654291   1.074895
H   4.729997  -1.849849  -0.136129
H   3.246807  -0.830949  -1.706451
C   3.839351   0.667298  -0.341888
O   4.818481   0.800288   0.352806
O   3.227708   1.691131  -0.968780
H   3.704191   2.513758  -0.772709
H   1.174382   0.182095  -0.991792
H   1.173341  -1.533353  -0.549328
S   1.205428  -0.177591   1.401442
```



```
H   1.587786   1.115681   1.370345
C  -2.317792  -0.236479   0.745491
C  -3.765098  -0.727677   0.562174
N  -2.261019   1.007012   1.491912
H  -2.694136   1.750496   0.950553
H  -1.289555   1.271644   1.626014
H  -1.796307  -1.012025   1.314093
C  -1.623279  -0.166313  -0.615333
O  -1.268453   0.849919  -1.164509
O  -1.465846  -1.392033  -1.153029
H  -1.062479  -1.304142  -2.031422
H  -3.775547  -1.707898   0.088179
H  -4.230375  -0.805621   1.544689
S  -4.857888   0.413474  -0.394234
H  -4.283795   0.219759  -1.599501
28
Dimer 151 of the n...n  type
C  -1.698213  -0.412827   0.128994
C  -1.730808   1.043669  -0.357855
N  -1.397302  -1.325662  -0.962398
H  -2.157106  -1.319113  -1.637838
H  -1.329384  -2.275894  -0.612416
H  -0.886727  -0.491093   0.863049
C  -2.974235  -0.769105   0.894170
O  -3.652523  -1.748183   0.691393
O  -3.253020   0.142297   1.847708
H  -4.056345  -0.128775   2.320198
H  -1.780509   1.731703   0.483497
H  -0.822478   1.246225  -0.923872
S  -3.111919   1.425636  -1.523611
H  -4.099800   1.393960  -0.605683
C   1.698185  -0.412494  -0.130233
C   1.730792   1.042371   0.361452
N   1.397617  -1.328996   0.958119
H   2.157606  -1.324786   1.633362
H   1.329484  -2.278032   0.604960
H   0.886591  -0.488411  -0.864418
C   2.974192  -0.765944  -0.896790
O   3.653000  -1.745287  -0.697065
O   3.252288   0.148406  -1.847710
H   4.055694  -0.120830  -2.321109
H   1.780407   1.733175  -0.477646
H   0.822521   1.243158   0.928209
S   3.111953   1.420490   1.528376
H   4.099860   1.391424   0.610383
28
Dimer 152 of the n...n  type
C  -1.831964  -0.413006  -0.320700
C  -3.140080  -0.904218  -0.961087
N  -1.428040  -1.254146   0.789489
H  -2.125122  -1.199622   1.527226
H  -0.542524  -0.938402   1.189603
H  -1.067083  -0.474748  -1.106203
C  -1.913862   1.079035   0.030398
O  -1.545111   1.561052   1.075457
O  -2.417068   1.809225  -0.983649
H  -2.417804   2.745811  -0.728359
H  -3.346199  -0.368500  -1.885284
H  -3.039634  -1.966428  -1.182207
S  -4.615648  -0.771369   0.142533
H  -4.741985   0.569597   0.067108
C   1.874991  -0.667262   0.053185
C   1.669281   0.781302  -0.414911
N   1.554869  -0.810440   1.465246
H   2.122153  -0.168657   2.013246
H   1.789284  -1.745318   1.785281
H   1.177179  -1.288357  -0.523310
C   3.268455  -1.184762  -0.316151
O   4.014676  -1.754575   0.443132
```

```
O   3.559980  -0.941566  -1.607911
H   4.438153  -1.302610  -1.809716
H   1.702354   0.844831  -1.500067
H   0.697683   1.124456  -0.065505
S   2.869963   1.989429   0.296379
H   3.935969   1.591480  -0.428189
28
Dimer 153 of the n...n  type
C   1.827879  -0.878587  -0.279310
C   1.499710  -0.045424   0.969937
N   2.668560  -2.017986   0.046836
H   3.569589  -1.692075   0.387330
H   2.857786  -2.562392  -0.788772
H   0.869694  -1.250163  -0.656035
C   2.397499   0.015347  -1.384428
O   3.453405  -0.157515  -1.944686
O   1.562745   1.036123  -1.667902
H   1.944688   1.576080  -2.378016
H   0.787717   0.740187   0.734652
H   1.060929  -0.700294   1.720893
S   2.965400   0.728002   1.783762
H   3.179813   1.678949   0.851065
C  -2.756558  -0.299444  -0.861891
C  -1.754067   0.863713  -0.859299
N  -4.125383   0.176837  -0.975516
H  -4.353138   0.751480  -0.167888
H  -4.769721  -0.607552  -0.957783
H  -2.522174  -0.905097  -1.746167
C  -2.532366  -1.231785   0.335476
O  -3.401911  -1.627490   1.073503
O  -1.234860  -1.575524   0.467042
H  -1.141741  -2.188168   1.214164
H  -0.735067   0.503263  -0.963693
H  -1.973201   1.512571  -1.707135
S  -1.857435   1.961394   0.621711
H  -1.432473   1.053225   1.524004
28
Dimer 154 of the n...n  type
C  -2.162599   0.957337   0.257136
C  -1.639563  -0.142463   1.192780
N  -3.538026   1.313361   0.558300
H  -4.142065   0.510400   0.402444
H  -3.857222   2.041130  -0.073318
H  -1.528211   1.834555   0.436203
C  -1.928399   0.591104  -1.214582
O  -2.741969   0.698821  -2.097440
O  -0.668018   0.143262  -1.411244
H  -0.503898  -0.025362  -2.353097
H  -0.577069  -0.305101   1.041250
H  -1.804645   0.166903   2.224375
S  -2.507618  -1.762484   1.028504
H  -2.101068  -2.038028  -0.227828
C   3.016320  -0.540016   0.413486
C   2.292282   0.385054   1.409915
N   4.171616   0.110008  -0.179114
H   3.873094   0.914103  -0.724460
H   4.636928  -0.521022  -0.823846
H   3.355939  -1.405915   0.992265
C   2.010395  -1.078556  -0.609366
O   2.041290  -0.860461  -1.797280
O   1.053786  -1.815102  -0.012579
H   0.397681  -2.073294  -0.678683
H   1.500079  -0.159337   1.921285
H   3.011953   0.729348   2.152381
S   1.600489   1.929162   0.668550
H   0.806110   1.334271  -0.244014
28
Dimer 155 of the n...n  type
C   3.033553  -0.359326   0.628178
```



```
C   2.325801   0.971278   0.954080
N   4.166354  -0.154118  -0.258103
H   3.836809   0.152097  -1.169714
H   4.660923  -1.028263  -0.405985
H   3.398224  -0.753066   1.582194
C   2.013724  -1.370549   0.101946
O   1.926966  -1.738316  -1.045891
O   1.183043  -1.793119   1.077386
H   0.576759  -2.455568   0.709425
H   1.496389   0.803967   1.638735
H   3.049518   1.635204   1.426272
S   1.700366   1.900836  -0.513462
H   0.558819   1.208698  -0.669390
C  -2.305976  -0.864367   0.444722
C  -1.492919  -0.810166  -0.858040
N  -3.660629  -1.331163   0.205297
H  -4.144292  -0.675644  -0.403425
H  -4.181789  -1.349526   1.076306
H  -1.800455  -1.582969   1.101484
C  -2.239404   0.475278   1.188974
O  -3.194045   1.077009   1.616512
O  -0.968903   0.907802   1.313801
H  -0.961919   1.759357   1.779734
H  -0.440343  -0.645957  -0.649629
H  -1.590345  -1.766120  -1.371928
S  -2.066415   0.453799  -2.072743
H  -1.726744   1.540698  -1.348766
28
Dimer 156 of the n...n  type
C   2.523813  -0.658150   0.734316
C   1.630844   0.568049   0.977225
N   3.886070  -0.415720   1.183144
H   4.289650   0.344414   0.640801
H   4.463265  -1.230621   0.999593
H   2.097141  -1.474145   1.330787
C   2.441343  -1.132979  -0.722053
O   3.395202  -1.387947  -1.417633
O   1.166125  -1.247899  -1.142608
H   1.160943  -1.562517  -2.060820
H   0.585119   0.304097   0.858467
H   1.785406   0.910206   2.000306
S   2.019504   2.020523  -0.091458
H   1.480612   1.527136  -1.224904
C  -2.511950  -0.659206  -0.731651
C  -1.618940   0.566796  -0.973841
N  -3.868506  -0.424755  -1.201951
H  -4.281380   0.339710  -0.672800
H  -4.446144  -1.239273  -1.018053
H  -2.075003  -1.479796  -1.314397
C  -2.449458  -1.121919   0.729680
O  -3.412228  -1.389951   1.407869
O  -1.181089  -1.210785   1.176415
H  -1.188425  -1.519958   2.096455
H  -0.574358   0.305087  -0.842457
H  -1.763431   0.901612  -2.000792
S  -2.019896   2.025369   0.081652
H  -1.488065   1.540224   1.221965
28
Dimer 157 of the n...n  type
C  -3.249519  -0.279809  -0.229150
C  -2.579371   0.764718  -1.148398
N  -4.156708   0.336564   0.722133
H  -3.637541   0.909625   1.381065
H  -4.635347  -0.376097   1.263847
H  -3.820965  -0.940407  -0.887329
C  -2.140691  -1.122081   0.405037
O  -1.702720  -0.950188   1.521171
O  -1.652886  -2.025818  -0.458012
H  -0.853895  -2.448780  -0.087256
```

```
H  -1.918266   0.264268  -1.855694
H  -3.356717   1.283332  -1.709645
S  -1.659002   2.116334  -0.284667
H  -0.961286   1.345989   0.570774
C   2.069447   0.738917  -0.527809
C   1.021093  -0.367057  -0.349511
N   2.733805   0.641645  -1.815019
H   3.291555  -0.207619  -1.851326
H   3.380840   1.414688  -1.933731
H   1.514768   1.683763  -0.488267
C   3.026499   0.755991   0.668901
O   4.225431   0.628176   0.599158
O   2.359758   0.926361   1.827356
H   2.992908   0.927508   2.563010
H   0.480854  -0.233350   0.583409
H   0.318846  -0.319773  -1.179942
S   1.675394  -2.093670  -0.367430
H   2.180895  -2.085544   0.883024
28
Dimer 158 of the n...n  type
C   3.208423   0.076297   0.930534
C   1.705224   0.395278   0.895881
N   4.003796   1.275606   1.134753
H   3.881409   1.903289   0.344015
H   4.990784   1.039260   1.160169
H   3.361777  -0.596123   1.782930
C   3.614022  -0.726341  -0.311510
O   4.497523  -0.417675  -1.075499
O   2.858428  -1.831906  -0.455412
H   3.142427  -2.310283  -1.250712
H   1.121217  -0.522824   0.883021
H   1.447555   0.970039   1.785176
S   1.181662   1.439045  -0.533796
H   1.256130   0.468107  -1.466839
C  -3.263606  -0.052664   0.905400
C  -1.781331  -0.464096   0.961097
N  -4.141115  -1.205177   1.020082
H  -4.032268  -1.798702   0.201747
H  -5.111120  -0.905861   1.029878
H  -3.432168   0.605569   1.764748
C  -3.520113   0.797032  -0.343408
O  -4.251137   0.486973  -1.254331
O  -2.811854   1.941507  -0.312698
H  -2.974485   2.439366  -1.129749
H  -1.143446   0.418056   0.963740
H  -1.611099  -1.031006   1.876015
S  -1.239783  -1.573033  -0.411323
H  -1.124934  -0.612308  -1.350522
28
Dimer 159 of the n...n  type
C   1.549512   0.292667  -0.660348
C   2.040112  -1.111622  -0.290994
N   2.205690   0.794258  -1.857566
H   3.205126   0.876979  -1.687543
H   1.873553   1.730903  -2.066127
H   0.479961   0.205742  -0.872763
C   1.609838   1.266515   0.523074
O   1.923216   2.429425   0.435858
O   1.237023   0.683539   1.679290
H   1.229325   1.349881   2.384830
H   1.424237  -1.545970   0.492372
H   1.970209  -1.747241  -1.172975
S   3.809896  -1.178509   0.232668
H   3.632927  -0.572124   1.424405
C  -2.772650   0.039657   0.823458
C  -1.645718  -1.005456   0.891895
N  -4.079058  -0.586204   0.722645
H  -4.146336  -1.101938  -0.150822
H  -4.806023   0.121794   0.696402
```



```
H  -2.722340   0.601866   1.762997
C  -2.488842   1.058876  -0.285803
O  -3.210650   1.276809  -1.228477
O  -1.314513   1.694262  -0.086726
H  -1.175962   2.338291  -0.800014
H  -0.694002  -0.520892   1.092783
H  -1.861224  -1.693099   1.709754
S  -1.495568  -2.089293  -0.594752
H  -1.159236  -1.131844  -1.482763
28
Dimer 160 of the n...n  type
C  -2.724898  -0.786633   0.510735
C  -1.359446  -0.225873   0.085227
N  -3.140163  -1.880742  -0.351564
H  -3.258328  -1.537919  -1.301765
H  -4.048140  -2.228817  -0.059728
H  -2.595707  -1.172429   1.529264
C  -3.765015   0.334265   0.625429
O  -4.853447   0.322183   0.101731
O  -3.320821   1.348345   1.393670
H  -4.009817   2.029726   1.449275
H  -0.991580   0.483796   0.823861
H  -0.652721  -1.048242  -0.004914
S  -1.346956   0.583787  -1.573242
H  -2.057191   1.674924  -1.219279
C   2.196808  -0.273743   0.997562
C   1.957124   1.094047   0.337384
N   3.203873  -0.190726   2.042064
H   4.096332   0.074535   1.632838
H   3.344339  -1.102861   2.465205
H   1.245563  -0.569889   1.454629
C   2.498213  -1.339966  -0.062650
O   3.468941  -2.058156  -0.065273
O   1.536178  -1.385266  -1.005774
H   1.753682  -2.074323  -1.653643
H   1.114175   1.053790  -0.347633
H   1.744244   1.823950   1.118189
S   3.414579   1.762017  -0.579148
H   3.323695   0.931745  -1.638522
28
Dimer 161 of the n...n  type
C   2.674516  -0.228275   1.019055
C   1.926160   1.109877   0.856270
N   4.114047  -0.026257   1.019542
H   4.414143   0.291946   0.101664
H   4.592391  -0.905049   1.191314
H   2.375505  -0.632984   1.991448
C   2.195536  -1.225643  -0.036669
O   2.837652  -1.594739  -0.989526
O   0.935666  -1.645910   0.212394
H   0.654763  -2.241625  -0.502031
H   0.849101   0.970665   0.921943
H   2.246712   1.774173   1.658356
S   2.282773   2.009460  -0.716158
H   1.360621   1.371423  -1.464013
C  -2.015738  -0.904746   0.406928
C  -3.538831  -0.768504   0.209047
N  -1.587035  -0.383568   1.692527
H  -1.690311   0.626805   1.714987
H  -0.605921  -0.597844   1.835645
H  -1.797310  -1.974905   0.375247
C  -1.310232  -0.261237  -0.786182
O  -0.922073   0.883390  -0.820743
O  -1.197980  -1.114213  -1.822985
H  -0.790864  -0.651289  -2.573640
H  -3.835855  -1.210618  -0.742119
H  -4.037303  -1.307983   1.014773
S  -4.200226   0.950211   0.316815
H  -3.560089   1.444268  -0.762376
```

```
28
Dimer 162 of the n...n  type
C   2.003201  -0.743709   0.418941
C   1.934480   0.707132   0.920608
N   3.107297  -1.466899   1.027951
H   3.988687  -1.048737   0.740502
H   3.123488  -2.424618   0.691569
H   1.062590  -1.211726   0.727348
C   2.011700  -0.787568  -1.112061
O   2.835793  -1.356944  -1.786298
O   0.967397  -0.108689  -1.629752
H   0.995172  -0.164147  -2.598310
H   1.019040   1.184806   0.584894
H   1.937962   0.696246   2.010094
S   3.376728   1.751981   0.432148
H   3.044529   1.877935  -0.869283
C  -2.003302  -0.743382  -0.419525
C  -1.934030   0.707930  -0.919733
N  -3.107500  -1.465578  -1.029529
H  -3.988760  -1.046558  -0.742910
H  -3.124935  -2.423263  -0.693109
H  -1.062731  -1.211439  -0.728109
C  -2.012188  -0.788673   1.111439
O  -2.837162  -1.357498   1.785045
O  -0.966967  -0.111739   1.629803
H  -0.994727  -0.168055   2.598310
H  -1.018564   1.185000  -0.583267
H  -1.937166   0.698143  -2.009233
S  -3.376125   1.752723  -0.430702
H  -3.044463   1.877070   0.871019
28
Dimer 163 of the n...n  type
C   3.096585  -1.069813  -0.136602
C   1.633942  -0.602275  -0.199067
N   3.429471  -1.593695   1.177374
H   3.382638  -0.845183   1.864170
H   4.387672  -1.928958   1.186827
H   3.194453  -1.877494  -0.871385
C   4.036896   0.044248  -0.612143
O   4.971729   0.476130   0.019056
O   3.694147   0.492744  -1.835586
H   4.312798   1.191309  -2.102634
H   1.361478  -0.338587  -1.219284
H   0.990390  -1.414733   0.137989
S   1.245741   0.829466   0.903310
H   1.754930   1.789331   0.105256
C  -1.923400  -0.278999  -0.209042
C  -3.043922  -0.706730  -1.173075
N  -1.685557  -1.282242   0.814441
H  -2.545468  -1.447994   1.331588
H  -1.018025  -0.929999   1.493528
H  -1.023784  -0.172967  -0.821056
C  -2.216150   1.113758   0.352183
O  -2.452695   1.359451   1.511024
O  -2.202137   2.046552  -0.619885
H  -2.406056   2.912130  -0.231229
H  -3.127618   0.005464  -1.992042
H  -2.794632  -1.686012  -1.581984
S  -4.703637  -0.923591  -0.392474
H  -4.941901   0.381110  -0.146209
28
Dimer 164 of the n...n  type
C  -1.824793   0.510243   0.038392
C  -3.170120   0.998411   0.587754
N  -1.568785   1.044583  -1.292543
H  -2.337947   0.794337  -1.909902
H  -0.743986   0.609499  -1.693134
H  -1.048937   0.874120   0.721594
C  -1.678812  -1.011806   0.122342
```



```
O  -1.177651  -1.700094  -0.736888
O  -2.145154  -1.504792   1.284018
H  -1.982299  -2.461222   1.308216
H  -3.272019   0.738490   1.638931
H  -3.216632   2.082782   0.487752
S  -4.644228   0.358406  -0.325883
H  -4.545140  -0.914597   0.108731
C   1.724966  -0.332721  -0.134887
C   1.876210   1.161857  -0.463285
N   1.105606  -0.530533   1.163713
H   1.711801  -0.164688   1.893094
H   0.994580  -1.521979   1.349806
H   1.064554  -0.762551  -0.896737
C   3.060573  -1.066086  -0.296177
O   3.563031  -1.777303   0.541118
O   3.614266  -0.831008  -1.501882
H   4.448814  -1.322088  -1.568840
H   2.205715   1.296352  -1.491586
H   0.912989   1.655414  -0.339919
S   3.026616   2.073167   0.657200
H   4.168473   1.538788   0.176975
28
Dimer 165 of the n...n  type
C  -3.444051   0.564304   0.836848
C  -4.010146  -0.861395   0.872978
N  -4.257703   1.573073   0.155087
H  -4.803063   1.156017  -0.595549
H  -4.892650   2.040427   0.790756
H  -3.301227   0.883280   1.875655
C  -2.034721   0.595284   0.226841
O  -1.196621  -0.265461   0.437205
O  -1.792678   1.648398  -0.531660
H  -2.659342   2.141301  -0.546038
H  -3.332741  -1.522527   1.410549
H  -4.969624  -0.851304   1.389847
S  -4.360641  -1.576497  -0.789762
H  -3.075782  -1.749916  -1.161178
C   3.479369   0.616734  -0.830907
C   4.040253  -0.813908  -0.852867
N   4.446680   1.553074  -0.278761
H   4.630967   1.316040   0.693057
H   4.056512   2.490328  -0.272133
H   3.295181   0.887429  -1.877933
C   2.104232   0.661204  -0.146426
O   1.824347   1.414447   0.762900
O   1.253070  -0.217212  -0.677299
H   0.364357  -0.163152  -0.232729
H   3.369628  -1.482570  -1.387637
H   5.005388  -0.803874  -1.359011
S   4.384061  -1.529507   0.814802
H   3.100283  -1.743159   1.169442
28
Dimer 166 of the n...n  type
C   2.861957  -0.034961  -0.850278
C   1.538764  -0.800074  -0.957734
N   4.075693  -0.840466  -0.698293
H   3.890780  -1.678830  -0.152585
H   4.455765  -1.121182  -1.594343
H   2.964011   0.556770  -1.767722
C   2.844291   1.016299   0.272924
O   1.866998   1.672956   0.585576
O   4.013959   1.186610   0.863464
H   4.598669   0.494087   0.449960
H   0.714524  -0.115246  -1.148610
H   1.600754  -1.494947  -1.795411
S   1.143817  -1.853201   0.503287
H   0.874321  -0.850882   1.364524
C  -3.051564   1.041895   0.223178
C  -3.214900  -0.276415   1.002501
```

```
N  -3.979952   1.110046  -0.893775
H  -3.745022   0.394641  -1.576896
H  -3.889855   2.002217  -1.369670
H  -3.297811   1.842959   0.929206
C  -1.584918   1.258651  -0.166415
O  -1.179428   1.309614  -1.308837
O  -0.796849   1.377385   0.906126
H   0.145117   1.512930   0.639532
H  -2.570185  -0.287349   1.879293
H  -4.251440  -0.354606   1.329546
S  -2.906524  -1.809130   0.019069
H  -1.555719  -1.795234   0.084272
28
Dimer 167 of the n...n  type
C  -3.282856   0.325427   0.743611
C  -2.467643  -0.953658   0.976496
N  -4.607282   0.142712   0.144780
H  -4.611473  -0.648042  -0.495173
H  -5.322549  -0.008520   0.845791
H  -3.415136   0.806279   1.719939
C  -2.524259   1.371611  -0.091026
O  -1.332789   1.604677   0.013627
O  -3.291775   2.051293  -0.923434
H  -4.191057   1.632756  -0.819536
H  -1.516471  -0.727331   1.455345
H  -3.030859  -1.606748   1.642991
S  -2.160908  -1.948545  -0.545488
H  -1.134785  -1.209253  -1.020255
C   2.785928  -0.935364   0.528130
C   3.765366   0.164984   0.970286
N   3.407765  -1.848154  -0.417326
H   3.634889  -1.349208  -1.273744
H   2.752947  -2.578914  -0.677764
H   2.529195  -1.495910   1.434856
C   1.469909  -0.321862   0.032580
O   1.010981  -0.503314  -1.076994
O   0.881586   0.427374   0.966567
H   0.047928   0.832500   0.613454
H   3.329978   0.771206   1.761734
H   4.672556  -0.307009   1.346932
S   4.337977   1.279463  -0.386175
H   3.195671   1.984529  -0.518714
28
Dimer 168 of the n...n  type
C   3.086602   0.731095  -0.289593
C   3.486525  -0.742813  -0.124128
N   3.094385   1.542386   0.930669
H   2.977795   0.962126   1.757815
H   3.948306   2.077758   1.028408
H   3.773204   1.177649  -1.016359
C   1.695322   0.843906  -0.930263
O   1.342778   0.172146  -1.885031
O   0.903626   1.732988  -0.355459
H   1.461806   2.096484   0.391419
H   3.419783  -1.254548  -1.082738
H   4.515712  -0.797793   0.230271
S   2.488392  -1.656965   1.128352
H   1.308580  -1.620574   0.467713
C  -3.099584  -0.759870  -0.392593
C  -3.465447   0.713832  -0.137931
N  -3.482697  -1.598572   0.732163
H  -2.941951  -1.333523   1.551749
H  -3.249671  -2.567798   0.539618
H  -3.672559  -1.072270  -1.273248
C  -1.618823  -0.866131  -0.777857
O  -0.801921  -1.512315  -0.153093
O  -1.330709  -0.144258  -1.860975
H  -0.346778  -0.095049  -1.985982
H  -3.321513   1.305069  -1.039548
```



```
H  -4.513168   0.767843   0.156872
S  -2.532891   1.508687   1.245226
H  -1.347147   1.611773   0.607589
28
Dimer 169 of the n...n  type
C   1.999992   0.200824  -0.835778
C   1.448901  -1.105970  -0.246439
N   3.148778   0.062080  -1.732976
H   3.679467  -0.781119  -1.529980
H   2.870969   0.042721  -2.706505
H   1.174144   0.678472  -1.372967
C   2.364961   1.207819   0.266952
O   1.653822   1.447494   1.229996
O   3.515196   1.824421   0.078342
H   3.861173   1.426106  -0.769997
H   0.611717  -0.901935   0.417720
H   1.096497  -1.739755  -1.060181
S   2.688105  -2.127470   0.656244
H   2.828655  -1.311120   1.720949
C  -3.112848   0.865925   0.325629
C  -3.380662  -0.584350   0.766360
N  -3.851668   1.193183  -0.882840
H  -3.509756   0.628768  -1.656328
H  -3.689704   2.161720  -1.140208
H  -3.478163   1.501982   1.139933
C  -1.604418   1.128390   0.230367
O  -1.028965   1.435595  -0.793556
O  -0.992583   0.969985   1.406729
H  -0.019343   1.150421   1.318793
H  -2.894847  -0.793934   1.717149
H  -4.455829  -0.717441   0.884610
S  -2.880562  -1.873886  -0.457360
H  -1.550754  -1.805808  -0.249049
28
Dimer 170 of the n...n  type
C  -2.103496   0.180174  -0.677779
C  -3.523406  -0.160423  -1.163717
N  -1.215139  -0.956292  -0.468377
H  -1.666158  -1.649496   0.123829
H  -1.014720  -1.408772  -1.354403
H  -1.652763   0.806263  -1.459931
C  -2.150331   1.100213   0.555708
O  -3.114415   1.809915   0.777206
O  -1.094514   1.135812   1.352264
H  -0.301754   0.549911   1.117374
H  -4.092568   0.745155  -1.365804
H  -3.451577  -0.738948  -2.085516
S  -4.486587  -1.221950  -0.003330
H  -4.683072  -0.273630   0.934451
C   1.938945  -0.608663   0.109631
C   1.933892   0.399599  -1.048736
N   1.336961  -0.036355   1.308262
H   1.911594   0.733569   1.646386
H   1.321714  -0.727868   2.053132
H   1.309099  -1.443122  -0.212435
C   3.333452  -1.182356   0.357418
O   3.908668  -1.173374   1.420137
O   3.851554  -1.720629  -0.762243
H   4.729965  -2.081774  -0.561634
H   2.299105  -0.064930  -1.961928
H   0.907867   0.725149  -1.212299
S   2.888154   1.945709  -0.732712
H   4.120763   1.411816  -0.857807
28
Dimer 171 of the n...n  type
C  -1.976817  -0.177480  -0.334488
C  -2.969024  -1.343575  -0.248255
N  -1.314976   0.210910   0.910668
H  -1.914094   0.032766   1.712620
```

```
H  -0.432360  -0.298142   1.019727
H  -1.199319  -0.477537  -1.047559
C  -2.623117   1.068894  -0.970005
O  -3.394926   1.031702  -1.903408
O  -2.226895   2.209338  -0.407326
H  -1.639355   1.907402   0.340435
H  -3.382449  -1.562821  -1.231295
H  -2.450830  -2.230068   0.117832
S  -4.355601  -1.072578   0.937998
H  -4.996411  -0.128424   0.218913
C   2.074568  -0.468597  -0.397600
C   2.101006   1.045338  -0.119463
N   1.375146  -1.186762   0.658094
H   1.910257  -1.136498   1.521575
H   1.294268  -2.171235   0.423635
H   1.519052  -0.599178  -1.333135
C   3.485360  -1.001684  -0.668640
O   4.029963  -1.874569  -0.035604
O   4.057438  -0.374395  -1.713388
H   4.947543  -0.736668  -1.850776
H   2.546183   1.581121  -0.955049
H   1.076706   1.391916   0.012478
S   2.967835   1.526258   1.435851
H   4.227388   1.325455   0.996199
28
Dimer 172 of the n...n  type
C   3.438653  -0.319661  -0.413920
C   3.600578   0.891042   0.517910
N   3.125749  -0.014754  -1.814028
H   2.713225   0.910791  -1.902467
H   3.944591  -0.062545  -2.407797
H   4.373478  -0.887945  -0.373983
C   2.367980  -1.282019   0.133052
O   2.296504  -1.648705   1.279625
O   1.507379  -1.698606  -0.811561
H   1.818645  -1.227383  -1.639287
H   3.741636   0.549830   1.542256
H   4.476952   1.465678   0.217794
S   2.199408   2.088320   0.452743
H   1.225346   1.246333   0.863505
C  -3.028005  -0.546470   0.879325
C  -3.827650  -0.488975  -0.433292
N  -3.407217   0.533405   1.775927
H  -3.160016   1.426860   1.357867
H  -2.881786   0.468629   2.642121
H  -3.288512  -1.499936   1.354178
C  -1.522114  -0.620746   0.594518
O  -0.707033   0.162626   1.033134
O  -1.212997  -1.655876  -0.193165
H  -0.245980  -1.673524  -0.369496
H  -3.632556  -1.369074  -1.042073
H  -4.889864  -0.456181  -0.192185
S  -3.522661   1.019913  -1.453267
H  -2.300306   0.671661  -1.905180
28
Dimer 173 of the n...n  type
C  -2.150976  -0.280320   0.799376
C  -3.599013   0.180188   1.036863
N  -1.194991   0.774776   0.467803
H  -1.567072   1.355235  -0.279922
H  -1.054565   1.381813   1.268937
H  -1.813066  -0.737392   1.738907
C  -2.076444  -1.420822  -0.235304
O  -3.073186  -1.925991  -0.720368
O  -0.871702  -1.860688  -0.556863
H  -0.086367  -1.452172  -0.037515
H  -4.238610  -0.666571   1.282559
H  -3.611941   0.877688   1.875615
S  -4.358602   1.112835  -0.360980
```



```
H  -4.443697   0.065934  -1.205207
C   2.318022  -0.658917  -0.381682
C   1.852555   0.633909  -1.067572
N   1.367140  -1.077792   0.647781
H   1.200013  -0.308069   1.292833
H   1.755534  -1.842158   1.194210
H   2.347366  -1.435202  -1.155985
C   3.748718  -0.549212   0.148351
O   4.092776  -0.839456   1.269198
O   4.592881  -0.108845  -0.800671
H   5.491261  -0.074594  -0.434394
H   2.463375   0.842241  -1.942577
H   0.815982   0.509888  -1.374755
S   1.851232   2.116810   0.029652
H   3.171348   2.381045  -0.061107
28
Dimer 174 of the n...n  type
C  -2.151270  -0.613109   0.410311
C  -2.075089   0.868420   0.801668
N  -1.383535  -1.024381  -0.764626
H  -1.389877  -0.287339  -1.466349
H  -0.411948  -1.226141  -0.506728
H  -1.776027  -1.185610   1.267774
C  -3.608391  -1.083593   0.243338
O  -4.531432  -0.697384   0.926382
O  -3.757740  -1.996334  -0.716576
H  -2.848239  -2.068413  -1.116615
H  -2.696880   1.061621   1.674349
H  -1.044475   1.123146   1.047591
S  -2.549224   2.035377  -0.544367
H  -3.868025   1.751932  -0.545166
C   2.452440  -0.745259  -0.542127
C   2.016531   0.688719  -0.897010
N   1.459922  -1.411980   0.287551
H   1.415159  -0.963177   1.199270
H   1.727666  -2.377368   0.452518
H   2.529625  -1.285446  -1.492164
C   3.858527  -0.748426   0.066238
O   4.133370  -1.142943   1.174448
O   4.767761  -0.248115  -0.791203
H   5.641482  -0.259742  -0.368282
H   2.728179   1.145842  -1.581417
H   1.044070   0.648313  -1.386327
S   1.767597   1.806344   0.548478
H   3.068472   1.987089   0.856380
28
Dimer 175 of the n...n  type
C  -3.243484   0.337840   0.450426
C  -2.746523  -1.027542   0.956888
N  -3.940161   0.326517  -0.839860
H  -3.703421  -0.508320  -1.370946
H  -4.946002   0.364539  -0.729769
H  -3.917195   0.737756   1.215130
C  -2.085697   1.353082   0.387339
O  -1.304797   1.535607   1.295325
O  -2.030491   2.038267  -0.753971
H  -2.796261   1.682216  -1.282129
H  -2.124971  -0.881282   1.839260
H  -3.602856  -1.642682   1.233641
S  -1.824745  -2.023365  -0.292238
H  -0.806465  -1.131187  -0.529248
C   1.516568   0.417207   0.191936
C   1.925318  -0.898359   0.877457
N   0.658963   0.169184  -0.956239
H   1.196902  -0.228055  -1.721641
H   0.246373   1.034326  -1.291048
H   0.934777   0.980563   0.927969
C   2.741140   1.272484  -0.136441
O   3.053801   1.645601  -1.242668
```

```
O   3.447554   1.569729   0.971663
H   4.215858   2.107198   0.720508
H   2.499175  -0.700420   1.780480
H   1.019958  -1.439828   1.150694
S   2.870475  -2.068813  -0.191162
H   4.048375  -1.411787  -0.164236
28
Dimer 176 of the n...n  type
C   2.687028   0.626573  -0.830792
C   3.580337  -0.607366  -1.009600
N   3.328300   1.823158  -0.278730
H   4.082798   1.572432   0.356081
H   3.706520   2.419036  -1.005302
H   2.282687   0.880845  -1.817275
C   1.446696   0.303586   0.018319
O   0.819351  -0.726751  -0.047228
O   1.098130   1.305711   0.837447
H   1.803240   1.998036   0.695529
H   3.005507  -1.429557  -1.432487
H   4.394680  -0.365128  -1.692254
S   4.399257  -1.176911   0.541008
H   3.289669  -1.656408   1.139395
C  -3.648950   0.476208   0.292443
C  -3.496555  -0.973148   0.789468
N  -4.432392   0.533846  -0.931323
H  -3.936870   0.047098  -1.674125
H  -4.532927   1.496599  -1.237490
H  -4.188256   1.016147   1.079292
C  -2.269101   1.135771   0.183124
O  -1.796041   1.573230  -0.842284
O  -1.632377   1.149774   1.362330
H  -0.700105   1.422125   1.227114
H  -3.028622  -0.991793   1.771368
H  -4.486847  -1.422186   0.861846
S  -2.546029  -2.080622  -0.342706
H  -1.323723  -1.551195  -0.114060
28
Dimer 177 of the n...n  type
C   1.957395  -0.526062   0.471966
C   3.439281  -0.510667   0.878941
N   1.642695  -1.358971  -0.698533
H   2.302963  -1.174165  -1.453738
H   1.768859  -2.338698  -0.454739
H   1.381986  -0.927679   1.311280
C   1.432770   0.907670   0.317605
O   1.657026   1.764478   1.136714
O   0.685974   1.186547  -0.765428
H   0.607390   0.401178  -1.330636
H   3.574744   0.081009   1.782214
H   3.764531  -1.531425   1.081032
S   4.576165   0.111288  -0.432866
H   4.244591   1.414448  -0.328328
C  -3.205772  -0.960382  -0.124420
C  -3.441751   0.326275  -0.938585
N  -3.948596  -0.935921   1.125341
H  -3.580437  -0.203368   1.726610
H  -3.820673  -1.810046   1.625580
H  -3.584211  -1.783794  -0.740033
C  -1.701461  -1.201317   0.041942
O  -1.116531  -1.159627   1.105122
O  -1.097173  -1.435611  -1.126590
H  -0.104999  -1.502681  -0.991815
H  -2.953262   0.259733  -1.908934
H  -4.513887   0.445649  -1.093258
S  -2.906536   1.885272  -0.105552
H  -1.577483   1.729708  -0.281443
28
Dimer 178 of the n...n  type
C  -2.175439  -0.729764   0.040700
```



```
C  -3.625802  -1.189335  -0.155404
N  -1.750255  -0.478827   1.418711
H  -2.525038  -0.140403   1.984039
H  -1.376108  -1.312465   1.855604
H  -1.528784  -1.511242  -0.375314
C  -1.860324   0.512193  -0.811359
O  -2.278212   0.698988  -1.928335
O  -1.026747   1.372439  -0.205864
H  -0.863430   0.983537   0.697524
H  -3.828844  -1.358498  -1.211504
H  -3.782438  -2.123988   0.383137
S  -4.883335  -0.023367   0.522309
H  -4.704363   0.950490  -0.393480
C   3.642967   0.671124   0.067596
C   3.724617  -0.518174  -0.906860
N   4.295621   0.364108   1.329329
H   3.791619  -0.381796   1.802094
H   4.262748   1.169227   1.946828
H   4.179823   1.497210  -0.412601
C   2.188127   1.130443   0.212457
O   1.567070   1.130591   1.252854
O   1.666799   1.516872  -0.961619
H   0.712187   1.699029  -0.836555
H   3.348081  -0.237501  -1.888209
H   4.768159  -0.817357  -1.001776
S   2.842469  -2.040356  -0.344328
H   1.586520  -1.603679  -0.572828
28
Dimer 179 of the n...n  type
C  -2.054628   0.376096  -0.051704
C  -2.211336  -0.823704  -0.994968
N  -1.551832   0.092602   1.291591
H  -1.868412  -0.821226   1.607025
H  -0.527086   0.113111   1.326719
H  -1.356496   1.072254  -0.532773
C  -3.366401   1.177485   0.064229
O  -4.124518   1.376421  -0.859738
O  -3.575420   1.664016   1.286222
H  -2.812589   1.298635   1.815509
H  -2.569836  -0.495966  -1.969376
H  -1.244756  -1.310277  -1.126149
S  -3.329728  -2.144302  -0.357544
H  -4.480553  -1.454249  -0.493127
C   2.090905  -0.503332   0.142310
C   2.011932   0.534208  -0.990058
N   1.514764   0.011814   1.375843
H   1.999181   0.863328   1.651002
H   1.657300  -0.652950   2.130938
H   1.495791  -1.364334  -0.185864
C   3.519721  -1.031854   0.311152
O   4.143280  -1.024422   1.345639
O   4.002072  -1.528746  -0.843164
H   4.900895  -1.863065  -0.692448
H   2.339321   0.098734  -1.931262
H   0.977205   0.856177  -1.099698
S   2.958141   2.087041  -0.680908
H   4.190430   1.573561  -0.874732
28
Dimer 180 of the n...n  type
C  -2.071621  -0.263930  -0.142987
C  -2.350480   1.003536   0.673393
N  -2.134491  -0.127171  -1.598882
H  -2.823952   0.569826  -1.869847
H  -1.231004   0.148193  -1.970353
H  -1.058150  -0.594029   0.112097
C  -2.977282  -1.430865   0.291139
O  -3.298739  -1.645407   1.438792
O  -3.353673  -2.215642  -0.718562
H  -2.976950  -1.772038  -1.523792
```

```
H  -2.252858   0.797000   1.737770
H  -1.627723   1.772445   0.399853
S  -4.001060   1.761236   0.354378
H  -4.734432   0.797123   0.947438
C   3.254223   0.637300   0.805335
C   1.771710   0.491926   0.424497
N   3.808029   1.883109   0.304095
H   3.813053   1.874166  -0.712621
H   4.774857   1.981660   0.596961
H   3.285903   0.658972   1.901298
C   4.053370  -0.607263   0.395334
O   5.057295  -0.596481  -0.276442
O   3.504640  -1.735051   0.887568
H   4.045002  -2.493667   0.613967
H   1.333932  -0.378253   0.908853
H   1.241147   1.384336   0.754753
S   1.455949   0.389071  -1.392101
H   1.862487  -0.885782  -1.560409
28
Dimer 181 of the n...n  type
C   2.235566  -0.438625   0.308064
C   1.857194   1.010126  -0.019236
N   2.610872  -0.715631   1.696694
H   3.086101   0.082784   2.110848
H   1.801879  -0.932802   2.266600
H   1.362970  -1.053697   0.061180
C   3.342954  -0.959972  -0.623848
O   3.425231  -0.674991  -1.797841
O   4.195724  -1.794500  -0.027887
H   3.907894  -1.803869   0.921321
H   1.547687   1.093248  -1.059519
H   1.025639   1.315322   0.615521
S   3.190916   2.238282   0.318132
H   4.009037   1.859992  -0.685141
C  -3.268274  -0.040056   0.924426
C  -1.769869  -0.083938   0.581173
N  -3.759242  -1.352814   1.306419
H  -3.700296  -1.986163   0.513100
H  -4.740123  -1.301322   1.562276
H  -3.364922   0.632039   1.785453
C  -4.069501   0.617780  -0.206394
O  -5.013638   0.117915  -0.770367
O  -3.597477   1.845270  -0.500348
H  -4.134413   2.226498  -1.213541
H  -1.385386   0.919138   0.411404
H  -1.236026  -0.528505   1.420818
S  -1.345930  -1.136372  -0.875362
H  -1.708385  -0.255544  -1.829995
28
Dimer 182 of the n...n  type
C   2.301462   0.152396   0.668343
C   3.699910   0.779313   0.627073
N   2.200212  -1.168359   1.295369
H   3.059565  -1.698230   1.172072
H   2.006801  -1.100695   2.287322
H   1.649842   0.839633   1.218072
C   1.671332   0.085977  -0.734947
O   1.767422   0.967015  -1.559726
O   0.971599  -1.027573  -0.955869
H   1.090352  -1.558569  -0.124556
H   3.659303   1.759347   0.154814
H   4.071733   0.898157   1.644889
S   4.966745  -0.250246  -0.232335
H   4.481849  -0.088393  -1.480562
C  -3.368191   0.415821  -0.752872
C  -1.892807   0.824146  -0.610436
N  -4.256885   1.472035  -0.298670
H  -4.119977   1.631272   0.696311
H  -5.225545   1.191872  -0.415010
```



```
H  -3.538410   0.254074  -1.824142
C  -3.626203  -0.940581  -0.084460
O  -4.466492  -1.150970   0.757983
O  -2.799210  -1.893714  -0.557099
H  -3.000443  -2.733065  -0.112959
H  -1.238279   0.083760  -1.063648
H  -1.746217   1.779784  -1.112921
S  -1.341154   1.109124   1.128086
H  -1.262894  -0.187236   1.492889
28
Dimer 183 of the n...n  type
C   1.758255  -0.018675   0.351351
C   2.717728  -1.200891   0.530824
N   0.947106  -0.017559  -0.867018
H   1.460645  -0.417141  -1.648934
H   0.099476  -0.556814  -0.725248
H   1.063718  -0.038759   1.198401
C   2.489241   1.329230   0.472086
O   3.389913   1.541756   1.253076
O   2.017002   2.270852  -0.347157
H   1.327404   1.804340  -0.885504
H   3.248144  -1.117273   1.477936
H   2.145952  -2.129093   0.533148
S   3.947059  -1.394487  -0.830779
H   4.684462  -0.305320  -0.532082
C  -2.836607   0.655331   0.765145
C  -3.756041   0.514041  -0.463687
N  -1.794263   1.638139   0.530942
H  -1.175539   1.328333  -0.213156
H  -1.223096   1.759572   1.360908
H  -3.472832   1.006763   1.584157
C  -2.320102  -0.725375   1.181280
O  -1.177723  -1.107749   1.059437
O  -3.302111  -1.491303   1.685663
H  -2.941444  -2.365592   1.906001
H  -4.591511  -0.148753  -0.245592
H  -4.147294   1.500300  -0.712024
S  -2.915623  -0.064002  -2.002107
H  -2.782463  -1.359196  -1.649442
28
Dimer 184 of the n...n  type
C   2.633362  -0.731414  -0.632252
C   2.201169   0.534237  -1.383274
N   1.595326  -1.439904   0.119285
H   0.814138  -0.845549   0.420081
H   1.215533  -2.215992  -0.408686
H   3.044416  -1.416182  -1.383044
C   3.817810  -0.440161   0.311920
O   4.755554   0.270332   0.019763
O   3.723373  -1.067806   1.481209
H   2.841387  -1.534792   1.418949
H   3.021985   0.904547  -1.995182
H   1.364111   0.296326  -2.039653
S   1.608701   1.911760  -0.308130
H   2.817042   2.261242   0.176838
C  -1.814296   0.416599   0.004193
C  -2.101713  -0.693041  -1.023078
N  -0.992011  -0.075881   1.096426
H  -1.521953  -0.734877   1.660725
H  -0.720075   0.688218   1.706735
H  -1.252092   1.191146  -0.529019
C  -3.112960   1.086340   0.464822
O  -3.496729   1.144183   1.608859
O  -3.790579   1.614634  -0.572354
H  -4.605122   2.025702  -0.240903
H  -2.645487  -0.295533  -1.877420
H  -1.152000  -1.096511  -1.372500
S  -3.014463  -2.148110  -0.350589
H  -4.226073  -1.561733  -0.261200
```

```
28
Dimer 185 of the n...n  type
C   1.513086   0.122375  -0.672107
C   1.993217  -1.215691  -0.100497
N   2.224573   0.620364  -1.852653
H   3.200744   0.334798  -1.837536
H   1.808608   0.281154  -2.711709
H   0.459120  -0.010259  -0.935100
C   1.499574   1.222440   0.401099
O   1.160638   1.035644   1.550440
O   1.867491   2.419597  -0.049119
H   2.110117   2.258563  -0.998553
H   1.378447  -1.501608   0.749474
H   1.896918  -1.986111  -0.865038
S   3.770976  -1.236877   0.391851
H   3.646057  -0.441742   1.474144
C  -2.662759  -0.624992   0.897251
C  -3.217583   0.737847   0.448232
N  -1.653537  -0.473540   1.929365
H  -0.845909   0.040807   1.584414
H  -1.310795  -1.382703   2.222702
H  -3.512428  -1.175560   1.319076
C  -2.202683  -1.444204  -0.314599
O  -1.087893  -1.882744  -0.475822
O  -3.202333  -1.627093  -1.199251
H  -2.869583  -2.150525  -1.946047
H  -4.091918   0.609816  -0.186216
H  -3.507919   1.299439   1.335613
S  -2.002998   1.814380  -0.433954
H  -2.086698   1.206229  -1.634827
28
Dimer 186 of the n...n  type
C  -2.555733  -0.801844   0.362681
C  -1.907143   0.439957   0.986296
N  -1.907493  -1.343648  -0.835845
H  -1.472556  -0.603494  -1.380981
H  -1.200462  -2.030862  -0.605102
H  -2.552278  -1.584229   1.130329
C  -4.048369  -0.570546   0.055705
O  -4.793824   0.058852   0.772822
O  -4.461290  -1.156454  -1.068516
H  -3.637802  -1.557643  -1.451562
H  -2.463815   0.748563   1.869703
H  -0.877166   0.213415   1.270231
S  -1.762653   1.874706  -0.162599
H  -3.080741   2.140122  -0.272120
C   1.962175  -0.580937   0.141292
C   1.983460   0.612374  -0.829442
N   1.477623  -0.188876   1.452725
H   2.104468   0.500165   1.860207
H   1.475345  -0.986142   2.081173
H   1.261602  -1.307900  -0.286055
C   3.322781  -1.288175   0.168433
O   3.991113  -1.470770   1.158170
O   3.691989  -1.698960  -1.060376
H   4.553264  -2.142491  -0.999077
H   2.247460   0.286452  -1.833174
H   0.989323   1.056380  -0.858703
S   3.101940   1.992978  -0.332991
H   4.266934   1.386048  -0.640192
28
Dimer 187 of the n...n  type
C   2.932527  -0.443777  -0.833753
C   1.525862  -0.833938  -0.364420
N   4.004186  -1.403954  -0.555895
H   3.826506  -1.905988   0.310810
H   4.102867  -2.083563  -1.300537
H   2.880468  -0.301846  -1.919392
C   3.346604   0.935791  -0.284938
```



```
O   2.585076   1.869211  -0.164880
O   4.641093   1.022205   0.021318
H   4.992930   0.108001  -0.138979
H   0.809991  -0.061274  -0.637260
H   1.233793  -1.769020  -0.842920
S   1.395618  -1.159544   1.446267
H   1.565077   0.117298   1.846541
C  -2.238703   0.108784   0.804146
C  -2.339869  -1.130601  -0.098807
N  -3.298576   0.127716   1.798026
H  -4.200999   0.218658   1.338317
H  -3.199513   0.937471   2.402023
H  -1.276374   0.021357   1.323179
C  -2.141237   1.396123  -0.022056
O  -2.835681   2.373221   0.127062
O  -1.158476   1.323128  -0.943017
H  -1.109619   2.165149  -1.423467
H  -1.473912  -1.207678  -0.751628
H  -2.377491  -2.015836   0.535328
S  -3.868847  -1.209928  -1.129407
H  -3.508537  -0.273937  -2.031582
28
Dimer 188 of the n...n  type
C  -1.977283  -0.408690  -0.582791
C  -2.512593   1.024091  -0.683433
N  -2.796619  -1.455879  -1.198349
H  -3.785540  -1.221854  -1.153425
H  -2.549069  -1.603664  -2.169448
H  -0.990961  -0.405562  -1.066925
C  -1.683246  -0.798259   0.874121
O  -1.197496  -0.043077   1.688679
O  -1.964907  -2.069603   1.157925
H  -2.367863  -2.421085   0.320722
H  -1.823067   1.718055  -0.205618
H  -2.608747   1.299705  -1.733825
S  -4.195657   1.262393   0.034737
H  -3.838252   1.109194   1.326225
C   1.631250   0.483495  -0.077440
C   1.942766  -0.925916   0.452720
N   1.168007   0.436541  -1.455214
H   1.902868   0.069825  -2.054744
H   0.962370   1.373039  -1.789066
H   0.818460   0.866228   0.550707
C   2.813991   1.430895   0.144521
O   3.357402   2.078395  -0.718984
O   3.180076   1.472175   1.440558
H   3.923810   2.088267   1.538362
H   2.164257  -0.896705   1.517103
H   1.069411  -1.558209   0.297729
S   3.314575  -1.795560  -0.423926
H   4.330023  -1.088003   0.112549
28
Dimer 189 of the n...n  type
C   2.635441   0.504290   0.971654
C   3.403840  -0.657526   0.332151
N   1.529252   0.164242   1.865241
H   0.969341  -0.618652   1.531533
H   1.852100  -0.047075   2.801628
H   3.367605   1.070724   1.559848
C   2.128695   1.512611  -0.076072
O   2.720534   1.788231  -1.094610
O   0.972373   2.086906   0.263718
H   0.723482   1.641864   1.117759
H   4.302486  -0.292592  -0.162252
H   3.700819  -1.359922   1.110531
S   2.466288  -1.661111  -0.903594
H   2.551292  -0.772431  -1.914499
C  -2.962675  -0.990103  -0.197773
C  -3.188691   0.287523  -1.022657
```

```
N  -3.751824  -0.977257   1.023429
H  -3.444761  -0.213469   1.620694
H  -3.596056  -1.831701   1.548993
H  -3.306678  -1.823396  -0.823125
C  -1.467517  -1.243202   0.031425
O  -0.968517  -1.464522   1.114825
O  -0.768590  -1.204436  -1.107954
H   0.182271  -1.382674  -0.921073
H  -2.708548   0.209260  -1.995403
H  -4.260281   0.418876  -1.169925
S  -2.617436   1.841080  -0.204576
H  -1.297658   1.681803  -0.434892
28
Dimer 190 of the n...n  type
C   1.600506  -0.049552  -0.272092
C   1.924461  -0.337847   1.198824
N   1.727482  -1.174967  -1.200536
H   2.434814  -1.834325  -0.886570
H   0.848399  -1.668355  -1.318304
H   0.566558   0.309991  -0.304237
C   2.427206   1.136475  -0.805324
O   2.668248   2.132550  -0.160004
O   2.829579   0.980506  -2.065500
H   2.515823   0.068570  -2.307653
H   1.778427   0.559202   1.797767
H   1.259913  -1.116813   1.573320
S   3.626161  -0.987609   1.485096
H   4.280897   0.158723   1.208253
C  -3.037698  -0.209022   0.925558
C  -2.029770  -1.255558   0.428333
N  -4.397063  -0.557906   0.552055
H  -4.487804  -0.568918  -0.460467
H  -5.047195   0.142297   0.894422
H  -2.966650  -0.217406   2.020534
C  -2.623728   1.211111   0.512185
O  -3.356662   2.026467   0.007329
O  -1.328430   1.459470   0.800232
H  -1.119939   2.375462   0.554181
H  -1.042599  -1.057300   0.838179
H  -2.350889  -2.240150   0.766833
S  -1.911894  -1.411585  -1.407293
H  -1.478685  -0.159344  -1.663094
28
Dimer 191 of the n...n  type
C   2.348607   0.729064   0.928878
C   3.060373  -0.626496   1.000434
N   0.937090   0.733528   1.332911
H   0.514610  -0.190391   1.281632
H   0.827735   1.063800   2.284543
H   2.898427   1.407119   1.590623
C   2.492672   1.357005  -0.471445
O   3.522365   1.335891  -1.107500
O   1.386170   1.949141  -0.917163
H   0.712971   1.778462  -0.212294
H   4.125266  -0.503345   0.813493
H   2.929657  -1.053871   1.994194
S   2.433344  -1.909032  -0.174620
H   2.971378  -1.372454  -1.288932
C  -2.904362  -0.781069  -0.658767
C  -2.862165   0.734191  -0.947012
N  -3.984214  -1.117286   0.252771
H  -3.805103  -0.708394   1.165828
H  -4.035159  -2.122516   0.384710
H  -3.091545  -1.268178  -1.620924
C  -1.520369  -1.239742  -0.194197
O  -1.237788  -1.521660   0.952380
O  -0.633710  -1.248779  -1.196028
H   0.256299  -1.492944  -0.850448
H  -2.074971   0.963327  -1.663543
```



```
H  -3.820220   1.029300  -1.374638
S  -2.663576   1.815282   0.535627
H  -1.380600   1.501922   0.819017
28
Dimer 192 of the n...n  type
C   2.994097   0.196009   0.622230
C   1.962890   1.330766   0.564055
N   4.117667   0.293367  -0.314224
H   3.845227   0.794031  -1.156813
H   4.912177   0.768778   0.096322
H   3.397282   0.180212   1.640927
C   2.323623  -1.181228   0.455082
O   1.278053  -1.487783   0.984771
O   3.019354  -2.028607  -0.299810
H   3.806398  -1.498813  -0.596688
H   1.152014   1.137079   1.264788
H   2.444706   2.270115   0.835507
S   1.247017   1.621246  -1.109264
H   0.486075   0.507788  -1.167728
C  -2.799387  -0.973858   0.186756
C  -3.186608   0.236273   1.056455
N  -1.844617  -1.835855   0.856893
H  -0.935776  -1.384762   0.929084
H  -1.698747  -2.682227   0.316106
H  -3.727977  -1.535784   0.030127
C  -2.357467  -0.521444  -1.210139
O  -1.283624  -0.762225  -1.713343
O  -3.317993   0.184790  -1.834599
H  -2.995072   0.448724  -2.711275
H  -3.960223   0.826808   0.569484
H  -3.574465  -0.134440   2.004943
S  -1.790784   1.345221   1.527831
H  -1.551899   1.867938   0.306681
28
Dimer 193 of the n...n  type
C   2.868666  -0.436500   0.683029
C   1.875200   0.658773   1.086892
N   4.272978  -0.033451   0.568905
H   4.348318   0.931938   0.257036
H   4.762303  -0.120132   1.451602
H   2.803636  -1.224612   1.442039
C   2.444639  -1.129360  -0.626574
O   1.293857  -1.367712  -0.920271
O   3.468400  -1.473566  -1.405556
H   4.266075  -1.116263  -0.934213
H   0.869965   0.252249   1.173840
H   2.164098   1.066690   2.055935
S   1.845014   2.110500  -0.050301
H   1.340811   1.464334  -1.121130
C  -1.901300  -0.897098  -0.156816
C  -1.725263   0.413729  -0.940023
N  -2.758243  -1.829772  -0.869658
H  -3.702339  -1.456940  -0.927841
H  -2.823910  -2.705619  -0.361012
H  -0.897195  -1.328530  -0.083746
C  -2.347586  -0.619696   1.281356
O  -3.331246  -1.080465   1.810747
O  -1.490144   0.208546   1.913813
H  -1.799106   0.350657   2.822863
H  -1.007558   1.065273  -0.447524
H  -1.350830   0.172183  -1.934102
S  -3.281014   1.364851  -1.228915
H  -3.414488   1.852206   0.021920
28
Dimer 194 of the n...n  type
C   2.790018  -0.141651   0.990273
C   2.952880  -1.494556   0.284684
N   1.569523   0.071046   1.769950
H   0.741880  -0.394806   1.382539
```

```
H   1.682733  -0.235807   2.728571
H   3.642854  -0.045363   1.673474
C   2.961877   1.036609   0.012128
O   3.735440   1.037852  -0.920352
O   2.195485   2.083942   0.312970
H   1.655751   1.761431   1.086298
H   3.954882  -1.576729  -0.133945
H   2.817703  -2.295013   1.012349
S   1.732540  -1.840914  -1.052878
H   2.200115  -0.927214  -1.926805
C  -2.275125  -0.107580   0.917331
C  -1.822256   1.298753   0.487156
N  -1.177310  -1.056643   0.844072
H  -0.891380  -1.194146  -0.122421
H  -1.465049  -1.964581   1.195145
H  -2.592565  -0.020184   1.962869
C  -3.519243  -0.545476   0.136504
O  -3.601493  -1.546815  -0.534336
O  -4.532701   0.328874   0.289359
H  -5.298946   0.015227  -0.217406
H  -2.616878   2.024809   0.644870
H  -0.964503   1.582698   1.095529
S  -1.236552   1.421780  -1.258648
H  -2.450650   1.367590  -1.844377
28
Dimer 195 of the n...n  type
C   3.078525  -0.197505  -0.695982
C   3.227493   1.213940  -0.110627
N   2.211812  -0.331652  -1.865706
H   1.394126   0.269735  -1.796616
H   2.702273  -0.117160  -2.725387
H   4.082122  -0.533282  -0.981447
C   2.621130  -1.202812   0.376427
O   3.004297  -1.196574   1.521910
O   1.746778  -2.111559  -0.080202
H   1.594319  -1.854231  -1.029760
H   3.933088   1.209258   0.718437
H   3.606270   1.881506  -0.884555
S   1.648466   1.978246   0.462600
H   1.525891   1.255504   1.594343
C  -1.985769   0.769567   0.488313
C  -3.367740   0.261515   0.939926
N  -2.101209   1.808205  -0.520849
H  -2.511730   1.421782  -1.366703
H  -1.176465   2.145293  -0.772729
H  -1.516332   1.198836   1.379251
C  -1.106141  -0.408726   0.062456
O  -0.786170  -0.652177  -1.079706
O  -0.727763  -1.158891   1.110508
H  -0.101109  -1.839255   0.799067
H  -3.264894  -0.456135   1.752307
H  -3.946725   1.112703   1.298058
S  -4.404687  -0.482729  -0.392342
H  -3.697641  -1.620976  -0.545674
28
Dimer 196 of the n...n  type
C   3.242323  -0.365010   0.250995
C   3.185225   1.147904   0.512725
N   3.507045  -0.760348  -1.136692
H   3.204163  -0.033339  -1.780809
H   4.489898  -0.943553  -1.297832
H   4.033572  -0.775941   0.887117
C   1.955506  -1.064239   0.731383
O   1.432627  -0.850825   1.802807
O   1.475402  -1.953910  -0.136359
H   2.112923  -1.910745  -0.899715
H   2.906690   1.332347   1.549354
H   4.171335   1.577950   0.336828
S   2.035971   2.067438  -0.600116
```



```
H   0.858637   1.608810  -0.051416
C  -1.829386   0.772338  -0.346072
C  -1.550914  -0.547688  -1.082324
N  -0.856920   1.006381   0.709865
H  -0.720176   0.170560   1.273785
H  -1.176488   1.739101   1.336196
H  -1.727516   1.569468  -1.095103
C  -3.280824   0.863479   0.133382
O  -3.623368   1.169022   1.250885
O  -4.148520   0.589808  -0.858925
H  -5.053742   0.685774  -0.521793
H  -2.134752  -0.603499  -1.997916
H  -0.492539  -0.589729  -1.337264
S  -1.863054  -2.068513  -0.084257
H  -3.210264  -2.005708  -0.131473
28
Dimer 197 of the n...n  type
C   3.162145  -0.816855  -0.001015
C   3.508463   0.421340   0.839411
N   3.319298  -0.676058  -1.451499
H   3.229431   0.296857  -1.734335
H   4.215873  -1.021351  -1.771470
H   3.810504  -1.630130   0.342426
C   1.733933  -1.310648   0.298502
O   1.271595  -1.401156   1.415286
O   1.046871  -1.657696  -0.787536
H   1.670411  -1.466534  -1.539685
H   3.284628   0.231977   1.887933
H   4.572997   0.635335   0.743540
S   2.663604   1.970443   0.303375
H   1.400613   1.604670   0.612955
C  -1.993388  -0.765540  -0.236037
C  -3.453778  -0.590321  -0.688857
N  -1.904314  -1.355943   1.086713
H  -2.311414  -0.725132   1.771881
H  -0.926234  -1.478307   1.341560
H  -1.526619  -1.447711  -0.952759
C  -1.233961   0.556809  -0.374446
O  -0.784768   1.201312   0.546718
O  -1.121158   0.937327  -1.660471
H  -0.642492   1.781051  -1.698744
H  -3.497760  -0.237193  -1.717545
H  -3.950080  -1.558819  -0.629875
S  -4.473591   0.525936   0.370424
H  -3.915755   1.690233  -0.020490
28
Dimer 198 of the n...n  type
C   3.222221   0.701253  -0.279415
C   3.095602  -0.473254  -1.260119
N   3.908280   0.416925   0.984233
H   3.814010  -0.564303   1.235913
H   4.894769   0.641351   0.938060
H   3.767410   1.497948  -0.797099
C   1.842374   1.313334   0.028504
O   0.992969   1.510137  -0.813091
O   1.668506   1.631995   1.309669
H   2.512554   1.341811   1.748032
H   2.531272  -0.164191  -2.138347
H   4.089738  -0.788834  -1.577014
S   2.322576  -1.981666  -0.535634
H   1.096849  -1.464097  -0.307753
C  -2.559942   0.341555   1.057217
C  -3.603557   0.683316  -0.020377
N  -1.511940   1.341545   1.117282
H  -0.980555   1.359242   0.249296
H  -0.838957   1.114105   1.842120
H  -3.100658   0.338998   2.011927
C  -2.046258  -1.092509   0.880113
O  -0.884254  -1.408009   0.768472
```

```
O  -3.052702  -1.987794   0.876881
H  -2.681275  -2.878616   0.771965
H  -4.451408   0.003645   0.032157
H  -3.957266   1.699298   0.153200
S  -2.955107   0.693237  -1.748710
H  -2.849161  -0.643596  -1.894565
28
Dimer 199 of the n...n  type
C  -2.357097   0.362116   0.890611
C  -1.813548  -1.036639   0.576162
N  -3.728484   0.425136   1.403878
H  -4.296960  -0.321248   1.010506
H  -3.756709   0.346831   2.413381
H  -1.693543   0.803862   1.643153
C  -2.240409   1.302201  -0.324516
O  -1.301369   1.301097  -1.089554
O  -3.260971   2.149735  -0.445415
H  -3.881432   1.892270   0.285769
H  -0.786656  -0.981509   0.223194
H  -1.840018  -1.643593   1.481411
S  -2.804669  -1.972489  -0.665906
H  -2.437897  -1.241363  -1.738413
C   2.248641  -0.310035   0.957744
C   1.785814   1.050854   0.410040
N   3.419966  -0.171055   1.806102
H   4.201808   0.176972   1.256951
H   3.702319  -1.076637   2.167590
H   1.421493  -0.690375   1.569724
C   2.424058  -1.326861  -0.177259
O   3.434822  -1.947244  -0.406311
O   1.294652  -1.460291  -0.900993
H   1.438694  -2.119778  -1.598505
H   0.840595   0.962108  -0.120519
H   1.652890   1.731342   1.250953
S   3.003658   1.888773  -0.695737
H   2.789399   1.125831  -1.787278
28
Dimer 200 of the n...n  type
C   2.365389  -0.485047   0.631124
C   1.729984   0.910244   0.664238
N   3.684126  -0.614597   1.258108
H   4.213321   0.249773   1.170149
H   3.612794  -0.843953   2.242265
H   1.677408  -1.164925   1.146852
C   2.443962  -1.035306  -0.805686
O   1.574051  -0.878688  -1.634420
O   3.550301  -1.734418  -1.052208
H   4.076387  -1.662874  -0.212641
H   0.752012   0.905904   0.186700
H   1.607096   1.223639   1.701226
S   2.754833   2.226325  -0.122386
H   2.566761   1.829173  -1.397577
C  -2.431230   0.399560  -0.761296
C  -1.682974  -0.919062  -0.494606
N  -1.658600   1.545469  -0.312230
H  -1.611830   1.553493   0.703276
H  -2.115259   2.409160  -0.587358
H  -2.549954   0.465214  -1.848714
C  -3.850010   0.344498  -0.183246
O  -4.286970   1.090478   0.661213
O  -4.570144  -0.654094  -0.731057
H  -5.455687  -0.658504  -0.333459
H  -2.230927  -1.763946  -0.907006
H  -0.704897  -0.865189  -0.972002
S  -1.319754  -1.254987   1.283467
H  -2.565800  -1.610018   1.658446
28
Dimer 201 of the n...n  type
C  -2.788696  -0.851824   0.635566
```



```
C  -3.036066   0.558753   1.184456
N  -1.572305  -1.532633   1.079968
H  -0.778083  -0.903489   1.247347
H  -1.731462  -2.072193   1.922072
H  -3.647775  -1.462926   0.937915
C  -2.828407  -0.875345  -0.905110
O  -3.590795  -0.214496  -1.575296
O  -1.948976  -1.723081  -1.438970
H  -1.464701  -2.085440  -0.644900
H  -4.021439   0.912891   0.885338
H  -2.996897   0.528639   2.273487
S  -1.781634   1.813956   0.681905
H  -2.202699   1.950762  -0.591516
C   1.867031  -0.722457   0.244449
C   1.310187   0.023872  -0.978690
N   1.208779  -0.294397   1.469402
H   1.299252   0.712729   1.577656
H   1.658519  -0.715665   2.276430
H   1.640679  -1.784254   0.083880
C   3.395687  -0.637055   0.306770
O   4.031347  -0.281663   1.270707
O   3.962453  -1.030789  -0.849764
H   4.927704  -0.978281  -0.761491
H   1.691078  -0.411501  -1.899510
H   0.224951  -0.060136  -0.977218
S   1.647594   1.837654  -0.993138
H   2.939530   1.756875  -1.373407
28
Dimer 202 of the n...n  type
C   2.034005  -0.345359   0.122323
C   2.030781   0.927721  -0.733188
N   2.050017  -0.154942   1.573315
H   2.547058   0.695370   1.825930
H   1.104531  -0.104257   1.939922
H   1.125095  -0.905803  -0.127481
C   3.187525  -1.287374  -0.272253
O   3.556724  -1.461432  -1.412422
O   3.729271  -1.926239   0.764048
H   3.253883  -1.552590   1.553088
H   1.991638   0.672294  -1.790717
H   1.153725   1.526448  -0.487618
S   3.468252   2.043234  -0.435054
H   4.399438   1.256321  -1.011854
C  -3.255405   0.898276  -0.255513
C  -1.794388   0.765940   0.206385
N  -4.107695   1.379683   0.817305
H  -4.152850   0.687653   1.560669
H  -5.057338   1.506806   0.482283
H  -3.253550   1.643882  -1.059398
C  -3.742801  -0.405928  -0.900691
O  -4.717933  -1.029504  -0.555127
O  -2.946947  -0.781083  -1.921022
H  -3.291532  -1.603829  -2.304024
H  -1.149319   0.519617  -0.634279
H  -1.476316   1.718334   0.629444
S  -1.523998  -0.467626   1.553259
H  -1.626013  -1.576758   0.792242
28
Dimer 203 of the n...n  type
C  -1.978796   0.126512   0.793688
C  -1.972607  -1.308050   0.251746
N  -3.082268   0.480498   1.689468
H  -3.934517  -0.009221   1.427062
H  -2.870676   0.253966   2.653831
H  -1.039247   0.272794   1.337369
C  -1.910641   1.161384  -0.343235
O  -1.269016   1.005567  -1.359155
O  -2.598344   2.274988  -0.095739
H  -3.050248   2.097566   0.769768
```

```
H  -1.105918  -1.469216  -0.386878
H  -1.922275  -2.008392   1.085710
S  -3.493247  -1.773450  -0.681120
H  -3.260964  -0.992867  -1.756179
C   1.894991  -0.001366  -0.808241
C   1.985745  -1.274266   0.048836
N   2.805102  -0.059436  -1.939690
H   3.767266  -0.063299  -1.611098
H   2.699748   0.768632  -2.517041
H   0.868438   0.046220  -1.190086
C   2.054686   1.257417   0.051233
O   2.833855   2.154961  -0.163882
O   1.194492   1.263971   1.092127
H   1.316105   2.084940   1.595587
H   1.221783  -1.278800   0.823235
H   1.832921  -2.137955  -0.597752
S   3.629020  -1.555908   0.841877
H   3.517200  -0.607746   1.794938
28
Dimer 204 of the n...n  type
C   2.837746  -0.416745   0.735914
C   1.848003   0.696154   1.096208
N   4.223774  -0.011237   0.493756
H   4.267957   0.911251   0.067917
H   4.764304   0.010186   1.350200
H   2.835880  -1.125697   1.571764
C   2.344327  -1.242654  -0.467327
O   1.179266  -1.506067  -0.670660
O   3.323181  -1.672003  -1.259531
H   4.145622  -1.267461  -0.879157
H   0.860986   0.275786   1.282004
H   2.184055   1.204391   1.999722
S   1.707006   2.026628  -0.176762
H   1.164138   1.261615  -1.147886
C  -3.300388   0.498596   0.125477
C  -2.967738  -0.589070   1.167116
N  -4.124706  -0.030156  -0.947489
H  -3.603031  -0.729270  -1.469251
H  -4.365148   0.706071  -1.603692
H  -3.870388   1.266262   0.659798
C  -2.007268   1.171831  -0.344700
O  -1.532956   1.063321  -1.453471
O  -1.440097   1.886901   0.640819
H  -0.566336   2.222564   0.342001
H  -2.390173  -0.165294   1.986868
H  -3.903356  -0.981123   1.565690
S  -2.087847  -2.069456   0.505260
H  -0.887761  -1.500183   0.260803
28
Dimer 205 of the n...n  type
C   2.381825  -0.133844   0.809958
C   2.329193   1.222378   0.097596
N   3.661985  -0.502536   1.419314
H   4.441610  -0.137272   0.877321
H   3.742228  -0.150970   2.365903
H   1.621922  -0.104456   1.599037
C   1.923838  -1.276703  -0.113553
O   1.035682  -1.170469  -0.930227
O   2.575046  -2.421988   0.089775
H   3.261634  -2.196120   0.769830
H   1.331399   1.402750  -0.295923
H   2.568180   2.012413   0.809640
S   3.555109   1.410828  -1.266764
H   2.965915   0.553210  -2.124885
C  -3.002862   0.803243  -0.598974
C  -1.544140   0.909874  -0.122263
N  -3.861891   1.730214   0.118173
H  -3.897308   1.474178   1.101565
H  -4.813436   1.670892  -0.230154
```



```
H  -2.997561   1.081761  -1.659219
C  -3.484540  -0.651819  -0.551718
O  -4.426059  -1.052593   0.091318
O  -2.726626  -1.449961  -1.328244
H  -3.061259  -2.359015  -1.266912
H  -0.884585   0.286376  -0.722007
H  -1.231527   1.950143  -0.211633
S  -1.280862   0.482997   1.654683
H  -1.320060  -0.857532   1.511072
28
Dimer 206 of the n...n  type
C  -1.794111   0.229928   0.338877
C  -1.784696  -0.834947  -0.763422
N  -2.116155  -0.235537   1.690677
H  -2.791134  -0.996409   1.658909
H  -1.294096  -0.569513   2.179643
H  -0.790443   0.673149   0.349315
C  -2.717230   1.406016  -0.016625
O  -2.848704   1.846993  -1.136991
O  -3.340855   1.936234   1.037444
H  -3.077276   1.354527   1.796442
H  -1.534582  -0.381846  -1.721268
H  -1.032036  -1.586621  -0.525734
S  -3.366524  -1.767194  -0.936311
H  -4.098324  -0.747971  -1.431122
C   2.541741   0.964553  -0.036203
C   2.258871   0.314464   1.332217
N   1.333347   1.059873  -0.838303
H   1.104621   0.147612  -1.223403
H   1.482029   1.677124  -1.630128
H   2.900056   1.976856   0.177706
C   3.692768   0.239094  -0.741428
O   3.597825  -0.360654  -1.786297
O   4.839977   0.334356  -0.042955
H   5.536727  -0.152651  -0.511761
H   3.160659   0.285233   1.941039
H   1.510252   0.914641   1.848822
S   1.528978  -1.376688   1.250883
H   2.633941  -2.032441   0.841674
28
Dimer 207 of the n...n  type
C  -2.253366   0.623432   0.452054
C  -2.443396  -0.767728   1.067733
N  -3.396793   1.535387   0.532249
H  -4.273981   1.024502   0.464303
H  -3.400664   2.056436   1.401032
H  -1.408785   1.096503   0.966433
C  -1.788197   0.536582  -1.012314
O  -1.028784  -0.310474  -1.427872
O  -2.268406   1.509323  -1.786061
H  -2.883869   2.013812  -1.193784
H  -1.530652  -1.352612   0.970232
H  -2.676945  -0.663761   2.127414
S  -3.847326  -1.726114   0.353180
H  -3.279070  -1.963791  -0.846633
C   2.884676  -0.736235   0.788354
C   1.483895  -0.104379   0.866026
N   3.786343  -0.144923   1.762278
H   3.949602   0.831801   1.531715
H   4.690809  -0.604582   1.730003
H   2.753638  -1.796502   1.033676
C   3.414038  -0.700546  -0.650057
O   4.424761  -0.141884  -1.006051
O   2.609081  -1.379006  -1.490359
H   2.973722  -1.325022  -2.388258
H   0.789874  -0.610346   0.198116
H   1.123629  -0.193251   1.890726
S   1.428675   1.702855   0.491655
H   1.465146   1.595283  -0.852050
```

```
28
Dimer 208 of the n...n  type
C  -1.941631  -0.806398  -0.274097
C  -1.666316   0.548004  -0.939698
N  -3.045266  -1.592479  -0.830481
H  -3.772776  -0.986470  -1.202261
H  -2.733513  -2.206840  -1.573080
H  -1.022009  -1.394419  -0.362544
C  -2.152022  -0.652583   1.242737
O  -1.507882   0.105509   1.934135
O  -3.086674  -1.460559   1.740723
H  -3.450121  -1.932001   0.945973
H  -0.839986   1.052532  -0.443391
H  -1.399591   0.389665  -1.984617
S  -3.114717   1.688385  -0.973723
H  -3.129899   1.962331   0.346914
C   2.828859  -0.665245   0.597908
C   1.844918   0.393047   1.125807
N   4.205833  -0.283559   0.858588
H   4.432260   0.564116   0.345056
H   4.841278  -1.002698   0.527853
H   2.610405  -1.585802   1.151433
C   2.542789  -0.984806  -0.874179
O   3.323042  -0.835478  -1.783709
O   1.293206  -1.464304  -1.047136
H   1.156639  -1.648046  -1.990758
H   0.816015   0.048241   1.055344
H   2.070035   0.575864   2.176459
S   1.982468   2.051363   0.328189
H   1.500145   1.706373  -0.883542
28
Dimer 209 of the n...n  type
C  -2.941695   0.712978  -0.449833
C  -2.219524  -0.360047  -1.274317
N  -2.105061   1.530598   0.432769
H  -1.336919   0.980828   0.809502
H  -1.710970   2.326075  -0.055484
H  -3.435692   1.385364  -1.160632
C  -4.095735   0.113265   0.375993
O  -4.810374  -0.781630  -0.016265
O  -4.263107   0.695500   1.563830
H  -3.521895   1.351978   1.623971
H  -2.930511  -0.897390  -1.899383
H  -1.475256   0.117234  -1.911349
S  -1.264441  -1.573117  -0.266834
H  -2.326073  -2.203584   0.275983
C   3.095191   0.602283   0.874174
C   1.669386   0.844359   0.350167
N   3.998280   1.661653   0.459726
H   4.093544   1.656987  -0.552498
H   4.926271   1.503540   0.839373
H   3.023440   0.613188   1.968137
C   3.575233  -0.806447   0.502901
O   4.572597  -1.056872  -0.131382
O   2.742918  -1.753311   0.977885
H   3.079248  -2.626980   0.721404
H   0.981721   0.103835   0.753946
H   1.348966   1.837017   0.665637
S   1.512510   0.855249  -1.488055
H   1.629066  -0.475807  -1.674895
28
Dimer 210 of the n...n  type
C  -2.001375  -0.500579  -0.132975
C  -3.431449  -0.829660  -0.579318
N  -1.657491  -0.846621   1.249762
H  -2.468408  -0.756880   1.858096
H  -1.316044  -1.797753   1.322799
H  -1.319952  -1.041749  -0.796285
C  -1.665323   0.982272  -0.376103
```



```
O  -1.997972   1.592399  -1.366829
O  -0.946722   1.539743   0.600053
H  -0.853592   0.822204   1.277932
H  -3.576465  -0.539151  -1.618421
H  -3.599192  -1.903151  -0.491259
S  -4.745821  -0.050328   0.453720
H  -4.538617   1.217821   0.043835
C   2.123063  -0.665269   0.662068
C   2.381829   0.845838   0.796244
N   3.098349  -1.438025   1.411182
H   4.024475  -1.301896   1.014291
H   2.894659  -2.429757   1.338030
H   1.131914  -0.845400   1.095215
C   2.014004  -1.066482  -0.813666
O   2.700011  -1.893614  -1.364428
O   1.039341  -0.377092  -1.442800
H   1.006690  -0.652671  -2.373151
H   1.577957   1.417201   0.338538
H   2.425323   1.092857   1.856805
S   3.995267   1.417138   0.106176
H   3.652347   1.353684  -1.196847
28
Dimer 211 of the n...n  type
C   1.893448   0.583389   0.255222
C   3.376221   0.843904   0.558289
N   1.183456  -0.276168   1.201697
H   1.739575  -1.102005   1.414654
H   1.042094   0.216850   2.077623
H   1.384709   1.555129   0.260330
C   1.703631   0.068711  -1.178768
O   2.420459   0.398817  -2.097914
O   0.668303  -0.748554  -1.371447
H   0.203534  -0.891622  -0.519599
H   3.797283   1.540785  -0.164273
H   3.465586   1.279239   1.553929
S   4.417343  -0.678035   0.599497
H   4.401050  -0.909136  -0.728829
C  -3.040521  -0.170301   0.988309
C  -3.173026   1.258859   0.425417
N  -2.003810  -0.253357   2.001083
H  -1.083811  -0.218094   1.565582
H  -2.058795  -1.145879   2.481807
H  -4.006217  -0.402554   1.449448
C  -2.857584  -1.166968  -0.159645
O  -1.840929  -1.784140  -0.386232
O  -3.963755  -1.268603  -0.916360
H  -3.792305  -1.884072  -1.647620
H  -4.010838   1.323646  -0.266967
H  -3.355730   1.936137   1.259237
S  -1.662510   1.923487  -0.398359
H  -1.746624   1.195293  -1.530324
28
Dimer 212 of the n...n  type
C  -2.151313  -0.588539   0.278172
C  -1.917203   0.748609   0.993553
N  -1.460568  -0.779901  -0.997772
H  -1.382122   0.098399  -1.504740
H  -0.532266  -1.168749  -0.873301
H  -1.819669  -1.379410   0.961064
C  -3.655317  -0.859279   0.075238
O  -4.508943  -0.566291   0.882395
O  -3.933925  -1.481443  -1.069999
H  -3.056015  -1.549604  -1.529840
H  -2.488983   0.784746   1.919097
H  -0.860683   0.851296   1.238980
S  -2.318947   2.233539  -0.022118
H  -3.658488   2.075683  -0.008917
C   2.250148  -0.143725   0.921518
C   1.956631   0.995998  -0.071322
```

```
N   3.279727   0.238062   1.870558
H   4.162348   0.378395   1.386135
H   3.432077  -0.503691   2.546374
H   1.319711  -0.320597   1.473368
C   2.545360  -1.450294   0.175452
O   3.568772  -2.083687   0.254310
O   1.503980  -1.827551  -0.600536
H   1.729767  -2.653708  -1.058015
H   1.115859   0.746394  -0.714121
H   1.706490   1.890972   0.497890
S   3.383816   1.489492  -1.130637
H   3.351035   0.429403  -1.964423
28
Dimer 213 of the n...n  type
C   3.164855   0.407757   0.223228
C   3.266530  -0.992172  -0.396770
N   3.099337   0.466822   1.686290
H   2.607077  -0.341415   2.060092
H   4.021728   0.497301   2.104220
H   4.050412   0.968811  -0.097251
C   1.977775   1.199812  -0.354861
O   1.622360   1.143097  -1.509443
O   1.376269   1.987885   0.540642
H   1.845825   1.791772   1.394177
H   3.314364  -0.922498  -1.482068
H   4.175941  -1.474989  -0.039349
S   1.891154  -2.129229   0.071010
H   0.953839  -1.603709  -0.744715
C  -1.439054   0.237375   0.460393
C  -2.395848  -0.864723   0.951149
N  -1.689039   1.502663   1.124361
H  -2.575678   1.889775   0.814045
H  -0.962770   2.166339   0.873732
H  -0.432762  -0.099717   0.728820
C  -1.473665   0.321357  -1.069221
O  -1.875358   1.262985  -1.708505
O  -1.016536  -0.816042  -1.635765
H  -1.050417  -0.723950  -2.601484
H  -2.122530  -1.829014   0.526615
H  -2.318202  -0.925437   2.036378
S  -4.183499  -0.549618   0.621346
H  -4.158846  -0.797951  -0.704414
28
Dimer 214 of the n...n  type
C   2.875508  -0.162881  -0.749221
C   2.083359  -1.404728  -0.321045
N   4.171616   0.034650  -0.094389
H   4.155404  -0.322400   0.858019
H   4.921032  -0.428007  -0.594816
H   3.049835  -0.247186  -1.827917
C   2.046155   1.125062  -0.591236
O   0.859060   1.200651  -0.831671
O   2.754828   2.172533  -0.182992
H   3.666766   1.806860  -0.031433
H   1.159944  -1.484060  -0.891044
H   2.683127  -2.294130  -0.515166
S   1.688611  -1.469931   1.479401
H   0.765145  -0.486516   1.477975
C  -2.987890  -0.142932   0.736445
C  -2.199866  -1.334189   0.158959
N  -4.301747  -0.037946   0.123800
H  -4.201905   0.174715  -0.865478
H  -4.821232   0.732148   0.533280
H  -3.113691  -0.345570   1.805856
C  -2.149952   1.134586   0.629195
O  -2.435144   2.097246  -0.042584
O  -1.023410   1.055975   1.364689
H  -0.455174   1.810046   1.134115
H  -1.289360  -1.508567   0.727760
```



```
H  -2.825610  -2.223763   0.225537
S  -1.765154  -1.181369  -1.629170
H  -0.805235  -0.240450  -1.484395
28
Dimer 215 of the n...n  type
C   1.997865   0.820989  -0.175788
C   1.867792  -0.424305  -1.060705
N   3.107168   1.726882  -0.485338
H   3.909376   1.212105  -0.840779
H   2.844470   2.419913  -1.175880
H   1.061387   1.378924  -0.279361
C   2.061637   0.447760   1.315495
O   1.416503  -0.449924   1.811087
O   2.870191   1.228660   2.030947
H   3.281461   1.834596   1.360941
H   0.998161  -1.008427  -0.767194
H   1.740736  -0.115489  -2.098300
S   3.356106  -1.513437  -1.059785
H   3.213553  -1.970323   0.201213
C  -2.613206   0.788628   0.510576
C  -1.918106  -0.464380   1.067500
N  -4.009704   0.839623   0.910284
H  -4.511366   0.053270   0.505256
H  -4.449881   1.679237   0.547183
H  -2.093339   1.647965   0.951626
C  -2.402347   0.913139  -1.003147
O  -3.283073   1.035574  -1.820359
O  -1.094145   0.879884  -1.330670
H  -1.009636   0.972080  -2.293513
H  -0.841656  -0.419231   0.924908
H  -2.117638  -0.517424   2.137616
S  -2.543792  -2.061645   0.385945
H  -1.942515  -1.975972  -0.818525
28
Dimer 216 of the n...n  type
C   2.577640  -0.337075  -0.770676
C   1.730707   0.931029  -0.604432
N   2.017233  -1.577666  -0.229905
H   1.488803  -1.402110   0.621103
H   1.414504  -2.046906  -0.895083
H   2.730968  -0.480408  -1.846534
C   3.997746  -0.145295  -0.203550
O   4.618479   0.891064  -0.279611
O   4.504862  -1.243992   0.356500
H   3.770707  -1.908558   0.309008
H   2.249964   1.786964  -1.032039
H   0.779258   0.810000  -1.121245
S   1.277100   1.315702   1.141004
H   2.510114   1.667664   1.558893
C  -2.473847   0.488560   0.716368
C  -1.747386  -0.863153   0.614679
N  -3.639815   0.398391   1.576925
H  -4.324637  -0.230680   1.165748
H  -4.089862   1.304556   1.657379
H  -1.758712   1.184988   1.170582
C  -2.770576   1.056786  -0.676778
O  -3.858914   1.398429  -1.072678
O  -1.650899   1.144676  -1.424051
H  -1.876355   1.518718  -2.290989
H  -0.819401  -0.759364   0.059116
H  -1.514719  -1.208291   1.621481
S  -2.736122  -2.221649  -0.145948
H  -2.652123  -1.793036  -1.422397
28
Dimer 217 of the n...n  type
C   2.071619  -0.390928  -0.064761
C   2.156088   1.006504  -0.691069
N   1.535509  -0.465363   1.295824
H   1.743720   0.380315   1.820222
```

```
H   0.532270  -0.608292   1.286260
H   1.423795  -0.993223  -0.711636
C   3.435237  -1.107871  -0.104926
O   4.207320  -1.037215  -1.035303
O   3.678627  -1.847168   0.976547
H   2.897189  -1.674985   1.566736
H   2.556619   0.942527  -1.701291
H   1.158833   1.442926  -0.741193
S   3.158488   2.212607   0.278796
H   4.361434   1.658777   0.022947
C  -2.261086   0.650404  -0.460067
C  -2.906160   0.757376   0.933741
N  -3.032535   1.371211  -1.456410
H  -3.944516   0.935929  -1.567810
H  -2.574728   1.323175  -2.361008
H  -1.276103   1.122716  -0.375639
C  -1.996481  -0.815338  -0.822666
O  -2.436494  -1.385722  -1.790234
O  -1.195037  -1.416846   0.085462
H  -1.067410  -2.344735  -0.171077
H  -2.263734   0.316535   1.693090
H  -3.048403   1.812569   1.165230
S  -4.586969   0.007562   1.064497
H  -4.194705  -1.283155   1.073292
28
Dimer 218 of the n...n  type
C   2.921883   0.604426   0.516841
C   1.703368  -0.278551   0.808694
N   2.735988   1.645906  -0.497940
H   2.083733   1.338341  -1.215467
H   2.376017   2.502250  -0.093725
H   3.197587   1.095430   1.457236
C   4.156720  -0.238126   0.145295
O   4.431994  -1.295574   0.666578
O   4.924546   0.321439  -0.790118
H   4.436214   1.145891  -1.048318
H   1.935597  -1.004143   1.585948
H   0.878465   0.346707   1.147550
S   1.055178  -1.173709  -0.668724
H   2.005880  -2.130090  -0.697279
C  -3.153998  -0.249528   0.964506
C  -2.789587   1.246353   0.961578
N  -2.048419  -1.071837   1.423962
H  -1.313765  -1.101945   0.721227
H  -2.356162  -2.030542   1.552732
H  -3.982649  -0.354872   1.674244
C  -3.715816  -0.670643  -0.399078
O  -3.253850  -1.535631  -1.104834
O  -4.805822   0.049654  -0.730000
H  -5.126257  -0.244757  -1.597745
H  -3.663395   1.854138   0.735049
H  -2.424508   1.512042   1.953351
S  -1.422016   1.716635  -0.183633
H  -2.100694   1.563559  -1.339281
28
Dimer 219 of the n...n  type
C  -2.332902  -0.725290   0.312428
C  -3.460046  -0.802553  -0.723190
N  -2.736805  -0.740278   1.720655
H  -3.635954  -0.281428   1.846375
H  -2.808388  -1.684242   2.081198
H  -1.672647  -1.583104   0.141948
C  -1.421604   0.491337   0.070837
O  -1.110260   0.893437  -1.027890
O  -0.961213   1.046089   1.193104
H  -1.413166   0.539823   1.918280
H  -3.047018  -0.797033  -1.730462
H  -4.017442  -1.728366  -0.580485
S  -4.713131   0.543197  -0.578925
```



```
H  -3.918528   1.544291  -1.009695
C   3.332492  -0.023452  -0.909332
C   1.953362  -0.687513  -1.074518
N   4.379075  -1.014631  -0.729272
H   4.234405  -1.514469   0.144061
H   5.285729  -0.563535  -0.660094
H   3.521966   0.519933  -1.841779
C   3.280418   1.037111   0.196471
O   3.904013   0.996524   1.231103
O   2.437072   2.038295  -0.120022
H   2.414435   2.675028   0.612245
H   1.187639   0.052928  -1.297027
H   2.009380  -1.397032  -1.899848
S   1.400340  -1.691385   0.372690
H   1.101144  -0.660008   1.188813
28
Dimer 220 of the n...n  type
C  -2.820703  -0.660774   0.805038
C  -2.368197   0.716084   1.306118
N  -1.826219  -1.452407   0.077802
H  -1.189550  -0.865612  -0.459135
H  -1.269550  -2.022645   0.703093
H  -3.129455  -1.235872   1.685859
C  -4.097057  -0.558847  -0.052443
O  -5.004936   0.208450   0.178788
O  -4.128191  -1.423199  -1.066322
H  -3.246802  -1.879889  -1.023384
H  -3.161903   1.187000   1.883571
H  -1.496907   0.594822   1.949983
S  -1.832833   1.873444  -0.026006
H  -3.059353   2.099542  -0.538624
C   3.171233   0.761806   0.404497
C   1.684422   0.443571   0.173055
N   3.666466   1.711123  -0.576907
H   3.636054   1.295881  -1.504403
H   4.638933   1.934883  -0.391304
H   3.231708   1.223237   1.397217
C   3.997469  -0.527416   0.495442
O   4.943104  -0.797896  -0.206161
O   3.543728  -1.338730   1.470924
H   4.093833  -2.138160   1.497553
H   1.292139  -0.176944   0.976221
H   1.130257   1.381116   0.150628
S   1.308743  -0.364713  -1.442821
H   1.801655  -1.583603  -1.140389
28
Dimer 221 of the n...n  type
C  -1.834786  -0.117466  -0.280579
C  -3.081590  -0.598325  -1.033184
N  -1.417525  -0.925667   0.867414
H  -2.218489  -1.354464   1.324531
H  -0.776336  -1.656352   0.581486
H  -1.007548  -0.114502  -0.998727
C  -1.976005   1.350744   0.163081
O  -2.489663   2.212370  -0.514699
O  -1.447261   1.601791   1.361347
H  -1.143362   0.714592   1.685962
H  -3.292078   0.060571  -1.873774
H  -2.905890  -1.604382  -1.414453
S  -4.592151  -0.736997   0.015961
H  -4.785554   0.586445   0.190388
C   2.250861  -0.310911   0.835635
C   2.121656   1.039615   0.105253
N   3.369354  -0.304661   1.760528
H   4.242991  -0.221564   1.247589
H   3.411823  -1.179938   2.272701
H   1.323815  -0.437857   1.405635
C   2.285170  -1.458923  -0.178701
O   3.218832  -2.199101  -0.369055
```

```
O   1.120160  -1.547973  -0.859816
H   1.184540  -2.267807  -1.508209
H   1.237755   1.054126  -0.528611
H   2.025998   1.825653   0.853611
S   3.583703   1.522419  -0.910162
H   3.366314   0.675926  -1.937759
28
Dimer 222 of the n...n  type
C   1.717491   0.177419  -0.170045
C   2.842453   1.072571  -0.704793
N   1.250492   0.462250   1.188009
H   2.013406   0.794120   1.773148
H   0.518198   1.163233   1.181620
H   0.865048   0.299439  -0.847194
C   2.085070  -1.314605  -0.274442
O   2.687456  -1.794852  -1.208325
O   1.651663  -2.048090   0.750861
H   1.229804  -1.393021   1.363772
H   3.098920   0.788818  -1.724018
H   2.506177   2.109605  -0.705837
S   4.371309   1.061606   0.326455
H   4.759029  -0.192982   0.018770
C  -3.101456   0.508551   0.763345
C  -2.236818  -0.751114   0.950263
N  -4.499324   0.163093   0.574377
H  -4.610768  -0.364428  -0.287567
H  -5.061537   1.002488   0.476385
H  -3.006039   1.083102   1.692182
C  -2.534392   1.409592  -0.340049
O  -3.122731   1.740910  -1.340019
O  -1.274939   1.817510  -0.061551
H  -0.971908   2.402404  -0.775145
H  -1.215276  -0.487435   1.213477
H  -2.662310  -1.337597   1.764331
S  -2.211717  -1.901138  -0.492044
H  -1.440059  -1.150949  -1.304610
28
Dimer 223 of the n...n  type
C  -2.037310  -0.753976  -0.280215
C  -2.079839   0.580444  -1.033400
N  -3.140087  -1.684576  -0.535187
H  -4.008077  -1.181178  -0.702884
H  -2.955700  -2.273280  -1.338738
H  -1.102312  -1.245940  -0.568000
C  -1.908478  -0.541824   1.238308
O  -1.260706   0.348955   1.742061
O  -2.552278  -1.456537   1.963974
H  -3.022670  -2.015819   1.293079
H  -1.199638   1.173848  -0.795652
H  -2.086133   0.386468  -2.106262
S  -3.594749   1.582599  -0.714064
H  -3.293757   1.918285   0.557040
C   2.391659  -0.833866   0.379968
C   2.008739   0.500744   1.039568
N   3.708850  -1.277139   0.806155
H   4.410660  -0.608747   0.497850
H   3.941821  -2.161391   0.365197
H   1.650647  -1.567340   0.721527
C   2.230648  -0.764549  -1.143578
O   3.084129  -1.056223  -1.946829
O   0.999455  -0.342284  -1.497704
H   0.939944  -0.322646  -2.466537
H   0.963205   0.738190   0.862687
H   2.161073   0.406604   2.114515
S   3.052496   1.935808   0.530285
H   2.498323   2.117322  -0.686061
28
Dimer 224 of the n...n  type
C   2.120167  -0.632514   0.409466
```



```
C   3.608441  -0.986796   0.532988
N   1.482854  -0.940604  -0.870844
H   2.125124  -0.771372  -1.641895
H   1.191964  -1.910264  -0.917518
H   1.591192  -1.192127   1.190316
C   1.865404   0.844865   0.759749
O   2.461227   1.438189   1.630425
O   0.897601   1.421883   0.045350
H   0.594925   0.749411  -0.612082
H   3.975749  -0.742664   1.528196
H   3.736773  -2.056448   0.366659
S   4.683752  -0.168718  -0.722061
H   4.609774   1.074879  -0.205457
C  -2.366677   0.584453   0.625392
C  -1.923527  -0.888192   0.667852
N  -3.482924   0.827292   1.522313
H  -4.297340   0.305589   1.208612
H  -3.742478   1.808406   1.504547
H  -1.506579   1.171357   0.968662
C  -2.619333   1.037839  -0.817640
O  -3.641998   1.533373  -1.225340
O  -1.539586   0.824969  -1.599794
H  -1.730928   1.134513  -2.499608
H  -1.021112  -1.037114   0.079698
H  -1.716337  -1.155780   1.703528
S  -3.199181  -2.096323   0.104039
H  -3.088178  -1.849773  -1.217556
28
Dimer 225 of the n...n  type
C   3.190443   0.514392   0.513701
C   3.404738  -0.984778   0.273379
N   2.504466   0.884311   1.754984
H   1.823691   0.175371   2.017820
H   3.153570   0.999639   2.524165
H   4.180165   0.985536   0.521895
C   2.459372   1.176105  -0.669954
O   2.635568   0.876960  -1.828336
O   1.621326   2.147615  -0.298394
H   1.658333   2.134032   0.694989
H   3.932366  -1.143914  -0.665487
H   4.004639  -1.398032   1.084309
S   1.849487  -1.975003   0.265421
H   1.365014  -1.498398  -0.899737
C  -1.651338   0.365564   0.449927
C  -2.994444   0.185358   1.172602
N  -1.509598   1.710643  -0.079345
H  -2.196526   1.866555  -0.812088
H  -0.592294   1.832140  -0.497320
H  -0.869962   0.208586   1.205269
C  -1.408157  -0.735496  -0.590709
O  -0.948666  -0.553058  -1.693865
O  -1.733227  -1.956833  -0.123945
H  -1.540434  -2.617469  -0.808739
H  -3.035580  -0.770514   1.689662
H  -3.102812   0.985255   1.904354
S  -4.474451   0.331367   0.078121
H  -4.348515  -0.869657  -0.522596
28
Dimer 226 of the n...n  type
C  -1.944143  -0.175899  -0.489248
C  -3.094670  -1.181079  -0.625586
N  -1.063935  -0.349457   0.667127
H  -1.586099  -0.688525   1.471610
H  -0.315092  -1.004221   0.470827
H  -1.330488  -0.265921  -1.393486
C  -2.456518   1.276432  -0.516526
O  -3.378217   1.652420  -1.205945
O  -1.768681   2.104127   0.270082
H  -1.107897   1.525392   0.729176
```

```
H  -3.670108  -0.978705  -1.527222
H  -2.684831  -2.189016  -0.692320
S  -4.237610  -1.218112   0.821214
H  -4.821281  -0.024318   0.590502
C   3.341887  -0.505851   0.732235
C   2.356157   0.594408   1.159501
N   4.675048   0.034307   0.528969
H   4.663083   0.700418  -0.239175
H   5.318705  -0.703702   0.262036
H   3.378643  -1.221365   1.562584
C   2.812422  -1.300404  -0.468567
O   3.400379  -1.459565  -1.510038
O   1.593715  -1.836502  -0.226770
H   1.323182  -2.352400  -1.004038
H   1.396736   0.175146   1.451686
H   2.781289   1.118006   2.015369
S   2.062557   1.906711  -0.102333
H   1.308818   1.168193  -0.941787
28
Dimer 227 of the n...n  type
C  -2.380660  -0.796464  -0.308012
C  -1.626889  -0.405493   0.968604
N  -1.885781  -0.210151  -1.556789
H  -1.468968   0.703432  -1.394082
H  -1.197885  -0.802372  -2.005751
H  -2.319314  -1.886945  -0.397107
C  -3.888991  -0.503418  -0.183376
O  -4.527544  -0.685461   0.829120
O  -4.441983  -0.054803  -1.309717
H  -3.679721   0.025647  -1.940976
H  -2.063453  -0.904909   1.831736
H  -0.585206  -0.712389   0.881400
S  -1.579852   1.410640   1.288874
H  -2.875119   1.544241   1.639687
C   3.318060   0.069667   0.697291
C   2.223392   1.126802   0.477985
N   4.583063   0.489303   0.119820
H   4.490173   0.577082  -0.888852
H   5.296217  -0.213811   0.285094
H   3.443729  -0.018499   1.783248
C   2.850780  -1.313872   0.226169
O   3.444429  -2.019034  -0.553872
O   1.683129  -1.669741   0.801180
H   1.435914  -2.554867   0.487863
H   1.323731   0.868357   1.031202
H   2.589079   2.085849   0.843863
S   1.785880   1.430788  -1.287845
H   1.194995   0.241267  -1.520655
28
Dimer 228 of the n...n  type
C  -2.598898   0.067408   0.977428
C  -2.909519   1.432288   0.351176
N  -1.240679  -0.094742   1.504052
H  -0.588792   0.557225   1.076072
H  -1.207301   0.044055   2.506569
H  -3.312156  -0.082765   1.795303
C  -2.901460  -1.072457  -0.015630
O  -3.890460  -1.104705  -0.714250
O  -1.986290  -2.039665  -0.026779
H  -1.286916  -1.736185   0.608144
H  -3.946984   1.470473   0.024131
H  -2.747981   2.215792   1.091711
S  -1.817229   1.872833  -1.070937
H  -2.339718   0.991058  -1.947819
C   2.575821   0.363413  -0.883113
C   1.451740  -0.682644  -0.945424
N   3.834444  -0.189621  -1.355164
H   4.133956  -0.939268  -0.736628
H   4.562696   0.516833  -1.323432
```



```
H   2.277615   1.174144  -1.559535
C   2.673853   1.009618   0.504987
O   3.689349   1.119685   1.148037
O   1.480218   1.475201   0.933334
H   1.603119   1.900620   1.797679
H   0.481136  -0.220322  -0.781799
H   1.453791  -1.127478  -1.940057
S   1.654835  -2.099969   0.218050
H   1.267727  -1.449349   1.334340
28
Dimer 229 of the n...n  type
C   2.117310   0.791577  -0.454287
C   1.827947  -0.557108  -1.126170
N   3.467663   1.329771  -0.635071
H   4.147618   0.580082  -0.737070
H   3.530107   1.930378  -1.448182
H   1.401640   1.513413  -0.865032
C   1.799339   0.752038   1.052958
O   0.826906   0.196019   1.521169
O   2.668501   1.419624   1.802433
H   3.350850   1.742573   1.154533
H   0.805682  -0.869530  -0.925614
H   1.956986  -0.455921  -2.203863
S   2.974549  -1.913321  -0.630766
H   2.527846  -2.050349   0.634378
C  -3.067177  -0.672992  -0.520465
C  -2.256712   0.275840  -1.425275
N  -4.377298  -0.123011  -0.217122
H  -4.274558   0.727521   0.330219
H  -4.907833  -0.772629   0.354750
H  -3.197427  -1.601734  -1.086865
C  -2.232210  -1.023110   0.715728
O  -2.514171  -0.721872   1.851708
O  -1.107225  -1.679034   0.378826
H  -0.507406  -1.683186   1.143862
H  -1.347783  -0.211310  -1.771529
H  -2.865543   0.536569  -2.290583
S  -1.812366   1.891550  -0.650249
H  -0.970740   1.399748   0.284565
28
Dimer 230 of the n...n  type
C   2.121214  -0.791983   0.455799
C   1.827181   0.557085   1.125161
N   3.474771  -1.322791   0.634182
H   4.150571  -0.569578   0.737585
H   3.541367  -1.925393   1.445470
H   1.409855  -1.516080   0.869913
C   1.798881  -0.756210  -1.050654
O   0.819282  -0.210416  -1.515798
O   2.673377  -1.414120  -1.802622
H   3.359508  -1.731793  -1.156109
H   0.804181   0.865981   0.922899
H   1.955822   0.458539   2.203143
S   2.969505   1.916162   0.627808
H   2.523210   2.049372  -0.637926
C  -3.066715   0.670088   0.522354
C  -2.253528  -0.278756   1.424584
N  -4.376094   0.118076   0.219396
H  -4.272239  -0.731087  -0.329844
H  -4.908642   0.767890  -0.350376
H  -3.198325   1.597479   1.090671
C  -2.234585   1.025148  -0.714415
O  -2.518682   0.727766  -1.850845
O  -1.110196   1.682374  -0.377884
H  -0.515307   1.696340  -1.146489
H  -1.344419   0.209024   1.769513
H  -2.860290  -0.540652   2.290983
S  -1.809185  -1.893293   0.647269
H  -0.966061  -1.399998  -0.285490
```

```
28
Dimer 231 of the n...n  type
C   2.121594  -0.093703   0.285256
C   3.361691  -0.795482   0.851558
N   1.506369  -0.708256  -0.892386
H   2.201791  -1.177368  -1.467188
H   0.796919  -1.381297  -0.622638
H   1.370201  -0.081609   1.083377
C   2.399246   1.391986  -0.011999
O   3.080723   2.104200   0.691252
O   1.790003   1.839385  -1.110081
H   1.348336   1.034176  -1.488343
H   3.725469  -0.265566   1.730154
H   3.098366  -1.812647   1.142175
S   4.743994  -0.980107  -0.355322
H   5.076323   0.325808  -0.414462
C  -3.188875   0.845104   0.201065
C  -1.716710   0.407720   0.275040
N  -3.759760   1.007441   1.526704
H  -3.789430   0.109860   2.003198
H  -4.719085   1.332099   1.460074
H  -3.190563   1.820342  -0.300208
C  -3.992819  -0.089231  -0.713005
O  -4.998765  -0.676900  -0.393429
O  -3.443473  -0.175695  -1.940257
H  -3.984650  -0.767664  -2.487078
H  -1.262891   0.402816  -0.713693
H  -1.178772   1.115759   0.904956
S  -1.439359  -1.237627   1.065600
H  -1.925086  -1.991555   0.057651
28
Dimer 232 of the n...n  type
C  -1.958483  -0.491085  -0.243579
C  -1.599594   0.998569  -0.196513
N  -2.592136  -0.968861  -1.474967
H  -3.168071  -0.241522  -1.892245
H  -1.905048  -1.260295  -2.159965
H  -1.023927  -1.045767  -0.113842
C  -2.827490  -0.901996   0.958851
O  -2.676047  -0.467473   2.078814
O  -3.751942  -1.816280   0.663535
H  -3.662494  -1.948145  -0.316011
H  -1.138991   1.242248   0.759355
H  -0.889837   1.217975  -0.993223
S  -3.023946   2.133713  -0.479493
H  -3.652949   1.893228   0.689160
C   2.205157  -0.714761   0.637827
C   2.194756   0.806874   0.873679
N   3.288054  -1.353226   1.364000
H   4.182464  -1.034149   1.000958
H   3.260714  -2.358824   1.228741
H   1.253593  -1.090408   1.031908
C   2.193159  -1.024863  -0.863536
O   3.048920  -1.635109  -1.457277
O   1.090667  -0.525069  -1.460995
H   1.124277  -0.729838  -2.409387
H   1.322800   1.263294   0.411361
H   2.155732   0.987657   1.947443
S   3.705459   1.692505   0.293591
H   3.428304   1.654677  -1.026047
28
Dimer 233 of the n...n  type
C  -2.735159   0.899121  -0.147108
C  -3.256491  -0.370757  -0.831637
N  -1.738380   1.680172  -0.884012
H  -1.120896   1.068101  -1.413430
H  -2.173500   2.328110  -1.530063
H  -3.601545   1.546080   0.033726
C  -2.183941   0.595961   1.258716
```



```
O  -2.647311  -0.235230   2.004524
O  -1.144829   1.363982   1.601030
H  -0.972942   1.916435   0.795419
H  -3.975511  -0.873881  -0.187327
H  -3.753658  -0.100047  -1.763269
S  -1.942240  -1.565288  -1.322583
H  -1.543202  -1.900170  -0.076996
C   2.898672  -0.284019  -0.697330
C   1.787111  -1.325047  -0.469728
N   4.086740  -0.595245   0.077128
H   3.878111  -0.536322   1.070168
H   4.819031   0.082292  -0.109829
H   3.150978  -0.339727  -1.762170
C   2.355159   1.130496  -0.467730
O   2.720019   1.886761   0.400861
O   1.396883   1.443431  -1.364284
H   1.071926   2.339216  -1.177747
H   0.928777  -1.113508  -1.104927
H   2.178640  -2.309055  -0.726933
S   1.211862  -1.493335   1.274138
H   0.571125  -0.310130   1.368533
28
Dimer 234 of the n...n  type
C   2.911396   0.255981  -0.694796
C   2.064831  -0.992273  -0.971618
N   4.318485   0.029989  -0.354433
H   4.432216  -0.842868   0.155717
H   4.904091  -0.007819  -1.180153
H   2.876995   0.874996  -1.598771
C   2.274963   1.136287   0.397503
O   1.079733   1.293574   0.521170
O   3.161745   1.737195   1.186307
H   4.038940   1.384501   0.882260
H   1.054043  -0.707028  -1.256225
H   2.509286  -1.551855  -1.794958
S   1.996956  -2.187672   0.431327
H   1.346461  -1.385978   1.299317
C  -1.660105  -0.138386   0.378618
C  -2.822120  -1.136174   0.221242
N  -1.713684   0.556570   1.651354
H  -2.532024   1.158457   1.681513
H  -0.897057   1.154376   1.736062
H  -0.745746  -0.735786   0.349356
C  -1.601009   0.792633  -0.834243
O  -1.916442   1.958647  -0.839412
O  -1.176183   0.137733  -1.935648
H  -1.178061   0.752166  -2.687055
H  -2.717464  -1.702977  -0.702532
H  -2.791673  -1.830224   1.061147
S  -4.512034  -0.398646   0.285206
H  -4.490912   0.189607  -0.928239
28
Dimer 235 of the n...n  type
C   2.726247  -0.499352  -0.877105
C   3.082936   0.967505  -0.607156
N   1.477612  -0.744380  -1.603735
H   0.777823  -0.031284  -1.410792
H   1.625790  -0.774272  -2.605323
H   3.548131  -0.924632  -1.464840
C   2.717115  -1.328199   0.421900
O   3.501765  -1.167612   1.329913
O   1.776376  -2.272517   0.443990
H   1.276592  -2.135554  -0.403516
H   4.060068   1.037282  -0.132422
H   3.117949   1.507448  -1.553458
S   1.855853   1.875881   0.428959
H   2.169172   1.279761   1.597149
C  -1.615971   0.152171   0.772762
C  -1.966509  -1.213872   0.164883
```

```
N  -2.339866   0.384050   2.011284
H  -3.338281   0.429601   1.823727
H  -2.078769   1.281713   2.406677
H  -0.543844   0.125602   1.002854
C  -1.764428   1.285597  -0.249502
O  -2.316373   2.338905  -0.043638
O  -1.176215   0.981879  -1.427579
H  -1.257612   1.741024  -2.027279
H  -1.321506  -1.441450  -0.680751
H  -1.822876  -1.977972   0.928202
S  -3.724991  -1.387785  -0.368157
H  -3.631432  -0.632827  -1.482092
28
Dimer 236 of the n...n  type
C  -2.500099   1.124055  -0.416553
C  -3.526143  -0.013785  -0.507462
N  -1.495981   1.185868  -1.476101
H  -1.220004   0.251877  -1.768280
H  -1.842661   1.678424  -2.290673
H  -3.065997   2.063254  -0.438438
C  -1.799537   1.145817   0.953974
O  -2.347329   0.843461   1.990942
O  -0.538788   1.576243   0.922039
H  -0.290877   1.678707  -0.029283
H  -4.232731   0.046985   0.318490
H  -4.076816   0.074433  -1.444177
S  -2.787997  -1.702808  -0.542719
H  -2.342919  -1.706199   0.730167
C   3.032332   0.028196   0.552796
C   2.714150  -1.477426   0.493537
N   2.446001   0.651764   1.725464
H   1.433748   0.687592   1.633071
H   2.760351   1.613796   1.802961
H   4.123581   0.102098   0.625113
C   2.658379   0.710967  -0.768744
O   1.855835   1.607744  -0.888962
O   3.340313   0.191633  -1.807217
H   3.069357   0.646609  -2.620937
H   3.225400  -1.946122  -0.345228
H   3.064002  -1.937333   1.417507
S   0.918550  -1.889757   0.414046
H   0.701842  -1.496102  -0.856752
28
Dimer 237 of the n...n  type
C  -2.130588  -0.448750   0.429986
C  -1.643695   1.005897   0.393047
N  -1.722516  -1.305288  -0.685309
H  -1.638375  -0.768090  -1.544913
H  -0.836116  -1.765776  -0.501155
H  -1.742653  -0.892219   1.354533
C  -3.663036  -0.524395   0.579929
O  -4.311627   0.230672   1.269550
O  -4.215533  -1.528033  -0.100860
H  -3.453353  -1.937023  -0.590244
H  -2.026213   1.550161   1.254831
H  -0.555228   1.032255   0.423654
S  -2.095160   1.918206  -1.144196
H  -3.420072   1.996373  -0.903031
C   3.230822   0.205652  -0.704225
C   2.027473  -0.702878  -1.002782
N   4.472600  -0.546325  -0.679216
H   4.456940  -1.218067   0.083623
H   5.257067   0.073694  -0.504840
H   3.277165   0.926601  -1.529269
C   2.984291   1.044382   0.557334
O   3.714695   1.085588   1.518000
O   1.841530   1.755596   0.466723
H   1.735324   2.284045   1.274292
H   1.124752  -0.109824  -1.132861
```



```
H   2.221579  -1.247230  -1.926481
S   1.715267  -2.017469   0.254988
H   1.362770  -1.205432   1.273827
28
Dimer 238 of the n...n  type
C   2.956582  -0.829349   0.183608
C   1.551104  -0.354996  -0.203915
N   3.261443  -0.855280   1.616756
H   2.778856  -0.104203   2.104544
H   2.991080  -1.733829   2.042588
H   3.069651  -1.848634  -0.202909
C   4.047137  -0.019011  -0.543162
O   3.949453   0.365852  -1.686836
O   5.135602   0.197239   0.195580
H   4.914030  -0.191541   1.081033
H   1.429629  -0.387615  -1.285667
H   0.805997  -1.004853   0.254742
S   1.137217   1.340578   0.387814
H   1.913401   2.011139  -0.487516
C  -3.668164  -0.762121  -0.197376
C  -3.753888   0.680303  -0.730423
N  -4.203370  -0.862295   1.148591
H  -3.636311  -0.311157   1.787371
H  -4.163226  -1.822525   1.474913
H  -4.289074  -1.369399  -0.866059
C  -2.244281  -1.309594  -0.345499
O  -1.538720  -1.676607   0.564991
O  -1.860634  -1.342559  -1.636775
H  -0.964551  -1.710566  -1.693066
H  -3.411603   0.728403  -1.762303
H  -4.795707   0.997894  -0.692647
S  -2.842172   1.934987   0.268031
H  -1.585942   1.578064  -0.081047
28
Dimer 239 of the n...n  type
C  -2.787558  -0.277740   0.863280
C  -1.444668   0.366063   0.499540
N  -2.907183  -1.715980   0.610178
H  -2.375725  -1.987424  -0.213638
H  -2.576425  -2.264925   1.394657
H  -2.943230  -0.111019   1.935294
C  -3.965721   0.447656   0.185104
O  -4.014411   1.646373   0.023806
O  -4.953902  -0.366724  -0.185387
H  -4.620724  -1.275193   0.032628
H  -1.445961   1.424755   0.752399
H  -0.648481  -0.128479   1.055047
S  -0.972048   0.173709  -1.272418
H  -1.872388   1.041673  -1.777031
C   3.313867  -0.495982   0.742196
C   2.608462   0.798553   1.187573
N   4.652214  -0.221644   0.248905
H   4.600434   0.335995  -0.599481
H   5.122212  -1.086706   0.001801
H   3.394304  -1.120745   1.638936
C   2.440843  -1.286369  -0.238786
O   2.724735  -1.528952  -1.387144
O   1.293872  -1.701193   0.340602
H   0.784847  -2.215313  -0.307128
H   1.639118   0.577257   1.630221
H   3.229374   1.280613   1.942180
S   2.394447   2.069387  -0.133996
H   1.345323   1.494232  -0.765292
28
Dimer 240 of the n...n  type
C   1.988567   0.298764  -0.577298
C   1.554251  -0.755194   0.447895
N   2.333694  -0.197614  -1.912685
H   2.743973  -1.126965  -1.858303
```

```
H   1.522340  -0.246216  -2.517150
H   1.153098   1.000725  -0.681060
C   3.149996   1.161033  -0.050815
O   3.252694   1.523903   1.099846
O   4.028179   1.508512  -0.992501
H   3.709151   1.041789  -1.808372
H   1.322519  -0.277881   1.398867
H   0.659954  -1.258900   0.078925
S   2.792392  -2.088923   0.736542
H   3.702813  -1.329725   1.380160
C  -2.690515  -0.892054   0.039225
C  -2.108042  -0.194625   1.282810
N  -1.662699  -1.148557  -0.955196
H  -1.368580  -0.271898  -1.377284
H  -2.034290  -1.720008  -1.707166
H  -3.085466  -1.852553   0.388267
C  -3.893507  -0.110372  -0.501846
O  -3.967205   0.373805  -1.606504
O  -4.876787  -0.018025   0.414176
H  -5.612292   0.491034   0.037027
H  -2.871454  -0.068627   2.047885
H  -1.311277  -0.818578   1.685799
S  -1.310404   1.436816   0.957538
H  -2.438797   2.143849   0.742315
28
Dimer 241 of the n...n  type
C   2.026593  -0.465616   0.004977
C   2.091813   1.063234  -0.103519
N   1.920947  -1.017712   1.357681
H   2.402265  -0.425619   2.030205
H   0.957169  -1.116717   1.653203
H   1.150106  -0.795074  -0.564134
C   3.217165  -1.130640  -0.714092
O   3.681756  -0.728946  -1.757233
O   3.675018  -2.218195  -0.095049
H   3.134317  -2.273984   0.735544
H   2.148839   1.364831  -1.147967
H   1.192982   1.497034   0.333807
S   3.490121   1.827444   0.823086
H   4.474449   1.386679   0.013311
C  -2.384182  -0.757708  -0.367763
C  -1.674193   0.606842  -0.342410
N  -2.855586  -1.081225  -1.702725
H  -3.573497  -0.418750  -1.984750
H  -3.289265  -1.998882  -1.710625
H  -1.633219  -1.501089  -0.074822
C  -3.471760  -0.826997   0.711369
O  -4.634490  -1.085380   0.510482
O  -2.970873  -0.569830   1.935623
H  -3.687173  -0.628463   2.588097
H  -1.224905   0.787744   0.631559
H  -0.893543   0.602331  -1.102511
S  -2.742723   2.046705  -0.776642
H  -3.418331   2.110254   0.389333
28
Dimer 242 of the n...n  type
C   1.855129   0.421952  -0.185972
C   2.047675  -1.077780   0.068452
N   1.882209   0.852287  -1.586881
H   2.509629   0.268850  -2.135292
H   0.963122   0.813027  -2.011024
H   0.880670   0.688929   0.235119
C   2.864147   1.264103   0.614688
O   3.217293   0.999906   1.742530
O   3.295390   2.345030  -0.036153
H   2.870810   2.277729  -0.930806
H   1.989646  -1.289729   1.134509
H   1.259621  -1.634563  -0.438543
S   3.624763  -1.761765  -0.599675
```



```
H   4.445214  -1.154736   0.282073
C  -2.324823  -0.712550  -0.588409
C  -2.552721   0.775615  -0.904013
N  -3.386516  -1.538898  -1.135662
H  -4.268234  -1.312620  -0.682737
H  -3.204957  -2.518761  -0.942854
H  -1.385425  -0.989283  -1.081636
C  -2.082353  -0.926798   0.911410
O  -2.695179  -1.695436   1.612395
O  -1.077242  -0.151465   1.368090
H  -0.944924  -0.326890   2.313692
H  -1.699820   1.373911  -0.591396
H  -2.681425   0.884411  -1.980466
S  -4.083118   1.493493  -0.163526
H  -3.634513   1.566835   1.106551
28
Dimer 243 of the n...n  type
C   2.505415  -0.743552  -0.572211
C   1.841498   0.573615  -0.993843
N   1.799241  -1.543333   0.431520
H   1.312788  -0.945405   1.095070
H   1.124556  -2.170550   0.010872
H   2.607498  -1.353984  -1.476856
C   3.954469  -0.519901  -0.095477
O   4.709201   0.287259  -0.589683
O   4.321172  -1.326845   0.900448
H   3.499931  -1.838461   1.119177
H   2.462527   1.086537  -1.726221
H   0.868546   0.369527  -1.439139
S   1.478747   1.723010   0.400371
H   2.759109   1.979123   0.739451
C  -2.050713   0.035485   0.794838
C  -2.119580  -1.284324   0.009596
N  -2.903799  -0.001885   1.969722
H  -3.876786  -0.085515   1.686601
H  -2.826760   0.867630   2.487604
H  -1.011494   0.144558   1.128319
C  -2.304641   1.240558  -0.119485
O  -3.106458   2.118172   0.092976
O  -1.499954   1.220384  -1.201382
H  -1.667659   2.013425  -1.735462
H  -1.390401  -1.294230  -0.797322
H  -1.898376  -2.104213   0.692416
S  -3.781117  -1.676910  -0.690626
H  -3.744006  -0.792567  -1.708501
28
Dimer 244 of the n...n  type
C   2.165182   0.615271   0.446217
C   3.214565  -0.056460   1.340918
N   0.997973  -0.199396   0.097138
H   1.260382  -1.177562  -0.004140
H   0.277562  -0.134909   0.807944
H   1.807565   1.503213   0.980142
C   2.796708   1.173665  -0.842599
O   3.893785   1.682493  -0.887258
O   2.008941   1.079045  -1.914916
H   1.208043   0.595278  -1.594813
H   4.027771   0.635285   1.553076
H   2.750235  -0.348010   2.283068
S   3.914108  -1.613690   0.643769
H   4.634419  -1.036301  -0.339514
C  -3.591845  -0.282019   0.704338
C  -2.725874  -1.457775   0.222352
N  -4.812682  -0.177231  -0.076400
H  -4.585569   0.050612  -1.041028
H  -5.389974   0.581663   0.271576
H  -3.862743  -0.508237   1.742869
C  -2.791640   1.024392   0.771666
O  -3.105353   2.059975   0.237192
```

```
O  -1.683923   0.901398   1.537988
H  -1.254568   1.770661   1.596102
H  -1.877594  -1.614502   0.885392
H  -3.339902  -2.357653   0.232391
S  -2.113984  -1.305625  -1.513402
H  -1.064824  -0.499851  -1.245493
28
Dimer 1 of the n...z  type
C   1.600758  -0.103114  -0.595542
C   2.843504   0.616669  -1.136454
N   0.732437   0.720715   0.264632
H   1.302274   1.202128   0.959592
H   0.280869   1.443571  -0.289932
H   0.994117  -0.403352  -1.455308
C   1.944066  -1.399869   0.160065
O   3.061886  -1.674284   0.545581
O   0.921158  -2.217144   0.384860
H   0.080257  -1.944129  -0.116883
H   3.463641  -0.082609  -1.697294
H   2.521420   1.409413  -1.813439
S   3.885983   1.470770   0.123359
H   4.284607   0.351826   0.758311
C  -2.308106  -1.069979  -0.711329
C  -2.622546  -0.575278   0.729981
O  -1.169474  -1.587933  -0.915625
O  -3.219428  -0.919523  -1.535554
H  -3.282511  -1.317495   1.186822
C  -3.350444   0.766624   0.743444
N  -1.371385  -0.527814   1.558835
H  -1.535158  -0.080194   2.461109
H  -3.541709   1.096857   1.765994
H  -4.307138   0.632367   0.244275
S  -2.450882   2.152352  -0.074836
H  -1.026501  -1.469363   1.747224
H  -0.568505  -0.008381   1.052953
H  -2.540170   1.647609  -1.322750
28
Dimer 2 of the n...z  type
C  -2.554882  -0.610801  -1.098294
C  -3.794634   0.187847  -0.664892
N  -2.721793  -2.033004  -0.843408
H  -2.811952  -2.188194   0.157745
H  -1.887267  -2.534866  -1.130692
H  -2.452135  -0.459157  -2.179609
C  -1.288460  -0.009154  -0.479904
O  -0.478432  -0.687557   0.142112
O  -1.169010   1.282681  -0.690701
H  -0.291986   1.643121  -0.323375
H  -3.721331   1.220265  -0.999975
H  -4.678145  -0.267989  -1.111094
S  -4.104449   0.176832   1.157167
H  -3.095453   1.007937   1.490157
C   2.201522   1.839990  -0.166989
C   2.690494   0.518770   0.488995
O   1.044817   2.237090   0.178464
O   2.988059   2.364721  -0.962155
H   3.660137   0.697385   0.953271
C   2.795662  -0.585403  -0.562972
N   1.736895   0.106222   1.573772
H   1.583028   0.870311   2.231290
H   3.461948  -0.243115  -1.351694
H   1.814905  -0.788273  -0.996869
S   3.379828  -2.204914   0.096704
H   0.817405  -0.137463   1.140902
H   2.086530  -0.711616   2.078333
H   4.620850  -1.794542   0.429722
28
Dimer 3 of the n...z  type
C   3.168597   0.228119   0.624494
```



```
C   3.273704  -0.710299  -0.596082
N   3.341721  -0.506192   1.867433
H   2.568405  -1.156689   1.982643
H   3.296578   0.130303   2.657286
H   3.977497   0.959515   0.525216
C   1.851902   0.998504   0.528939
O   0.932558   0.827201   1.316142
O   1.804321   1.803129  -0.518388
H   0.854282   2.068947  -0.716992
H   3.279227  -0.132478  -1.518353
H   4.207350  -1.267069  -0.520782
S   1.940712  -1.988388  -0.708522
H   0.982971  -1.199424  -1.247562
C  -0.990857   0.752557  -1.209172
C  -1.810088   0.118742  -0.054764
O  -0.731235   1.987319  -1.087164
O  -0.650870  -0.021364  -2.115833
H  -1.292050  -0.782564   0.272861
C  -3.228274  -0.213122  -0.506154
N  -1.806909   1.061593   1.111607
H  -0.819168   1.186083   1.404867
H  -3.166060  -0.863954  -1.376245
H  -3.761772   0.694659  -0.794969
S  -4.266689  -1.003990   0.795951
H  -2.159424   1.977764   0.826447
H  -2.371305   0.702522   1.885867
H  -3.552685  -2.144644   0.885942
28
Dimer 4 of the n...z  type
C   2.707594  -1.177571  -0.466876
C   3.214615  -0.549798   0.843238
N   3.339979  -0.569390  -1.627457
H   3.060277   0.406339  -1.689640
H   3.001571  -1.013740  -2.475395
H   2.991739  -2.235068  -0.426764
C   1.173412  -1.153974  -0.502174
O   0.537144  -0.543748  -1.344676
O   0.636866  -1.850463   0.484353
H  -0.387103  -1.850388   0.470574
H   2.784477  -1.063662   1.700908
H   4.299880  -0.644317   0.882130
S   2.894143   1.262671   1.003518
H   1.559766   1.195888   1.174817
C  -2.815065  -1.170167   0.385433
C  -2.307539   0.112649  -0.322126
O  -4.034947  -1.261303   0.596317
O  -1.906716  -1.993847   0.690063
H  -1.705354  -0.168005  -1.183212
C  -1.474092   0.970300   0.627685
N  -3.489974   0.893779  -0.828027
H  -3.938009   0.414142  -1.610133
H  -0.691016   0.347159   1.052184
H  -2.083822   1.346735   1.451530
S  -0.728574   2.452674  -0.172924
H  -4.197184   0.970169  -0.090489
H  -3.205874   1.828875  -1.138965
H   0.059192   1.755891  -1.018185
28
Dimer 5 of the n...z  type
C  -2.981810  -0.479014   0.232318
C  -3.117462   0.712202  -0.727258
N  -3.244647  -0.075642   1.607856
H  -2.526620   0.581162   1.905998
H  -3.153150  -0.878365   2.223153
H  -3.742474  -1.210609  -0.066374
C  -1.626530  -1.174335   0.048615
O  -0.922088  -1.493235   0.997280
O  -1.296738  -1.360643  -1.213249
H  -0.329555  -1.702320  -1.302096
```

```
H  -3.142816   0.376499  -1.761022
H  -4.043435   1.240115  -0.502369
S  -1.760277   1.966950  -0.569984
H  -0.916462   1.368502  -1.432963
C   1.861150  -1.683766  -0.424930
C   1.602263  -0.225920   0.059876
O   2.771975  -2.283417   0.166695
O   1.140474  -2.098011  -1.377639
H   0.645955   0.145952  -0.291698
C   2.727576   0.695854  -0.397333
N   1.515484  -0.266424   1.557978
H   0.630495  -0.727644   1.812909
H   2.771727   0.683336  -1.485688
H   3.685315   0.344782  -0.010366
S   2.549327   2.433274   0.196204
H   2.298645  -0.815149   1.927311
H   1.538194   0.676732   1.958435
H   1.423416   2.720185  -0.488017
28
Dimer 6 of the n...z  type
C  -2.671210  -1.152976  -0.641450
C  -3.456489   0.051104  -0.082534
N  -2.713385  -2.282084   0.273561
H  -2.229440  -2.036957   1.133666
H  -2.210205  -3.070249  -0.121977
H  -3.159401  -1.434933  -1.580067
C  -1.259882  -0.681298  -0.990999
O  -0.268872  -1.034555  -0.370544
O  -1.242126   0.187534  -1.987908
H  -0.355642   0.660970  -2.030696
H  -3.520424   0.838273  -0.831844
H  -4.463264  -0.279249   0.172307
S  -2.761950   0.767514   1.474715
H  -1.675391   1.358683   0.921311
C   0.987947   1.582094  -0.447403
C   2.103390   0.812110   0.321981
O   0.148486   2.159713   0.271928
O   1.015456   1.514725  -1.704363
H   2.810039   1.539649   0.725706
C   2.823050  -0.228306  -0.522748
N   1.430917   0.166449   1.506162
H   2.104552  -0.322428   2.101049
H   3.282561   0.274205  -1.369958
H   2.107554  -0.954117  -0.907102
S   4.093460  -1.197867   0.399623
H   0.936239   0.879886   2.046863
H   0.727599  -0.503865   1.160592
H   4.933957  -0.170474   0.638712
28
Dimer 7 of the n...z  type
C  -2.741365  -0.827352  -0.549221
C  -2.567618   0.694218  -0.727508
N  -3.832617  -1.144360   0.359819
H  -3.586364  -0.865610   1.305538
H  -3.980040  -2.148723   0.384428
H  -2.982047  -1.223541  -1.539417
C  -1.380433  -1.404666  -0.143590
O  -1.067937  -1.601466   1.020049
O  -0.574140  -1.579162  -1.175024
H   0.370939  -1.863186  -0.878238
H  -1.753448   0.892836  -1.425236
H  -3.487765   1.112248  -1.135860
S  -2.278570   1.645780   0.835772
H  -1.398481   0.794618   1.402094
C   2.542283  -1.359951   0.061153
C   1.875365   0.016647   0.355318
O   3.724813  -1.495357   0.414559
O   1.813314  -2.187379  -0.552882
H   0.896514  -0.134827   0.804567
```



```
C   1.766337   0.819200  -0.946040
N   2.727023   0.744140   1.356767
H   2.508193   0.461338   2.312695
H   1.091536   0.292143  -1.616685
H   2.742766   0.890958  -1.429312
S   1.209468   2.562256  -0.735305
H   3.707827   0.486799   1.181901
H   2.596007   1.760300   1.281434
H  -0.016656   2.295931  -0.225776
28
Dimer 8 of the n...z  type
C  -3.070508   0.797186   0.082632
C  -3.311162  -0.462840   0.930530
N  -3.354679   0.672486  -1.349604
H  -3.323230  -0.301212  -1.641759
H  -4.257970   1.057433  -1.595198
H  -3.688770   1.596711   0.503232
C  -1.628035   1.295436   0.248118
O  -1.079719   1.418459   1.329103
O  -1.023281   1.601540  -0.886954
H  -1.709524   1.399084  -1.584335
H  -2.960374  -0.288275   1.946965
H  -4.380531  -0.672006   0.960601
S  -2.532665  -1.993775   0.257362
H  -1.225438  -1.609593   0.368385
C   1.133683  -0.590304  -0.559302
C   1.908255   0.743234  -0.355287
O   0.604770  -1.079442   0.472017
O   1.145335  -1.032187  -1.722940
H   1.471481   1.493830  -1.013104
C   3.393692   0.597153  -0.671052
N   1.695548   1.225711   1.051980
H   2.195279   2.095672   1.239900
H   3.919040   1.545311  -0.542214
H   3.487359   0.288112  -1.710299
S   4.285123  -0.606932   0.408248
H   0.677217   1.368062   1.229066
H   2.018856   0.510052   1.707853
H   3.629567  -1.705965  -0.018120
28
Dimer 9 of the n...z  type
C  -2.070052  -0.289384   0.757014
C  -1.914694   0.994910  -0.083829
N  -1.120439  -1.326597   0.350643
H  -1.501089  -1.839806  -0.442241
H  -0.999192  -2.003345   1.098588
H  -1.866951  -0.009601   1.793615
C  -3.517737  -0.770945   0.684252
O  -3.884052  -1.750633   0.077921
O  -4.344133   0.060118   1.338745
H  -5.259138  -0.245409   1.228918
H  -2.677074   1.718925   0.200705
H  -0.934606   1.434013   0.097539
S  -1.952227   0.734138  -1.905981
H  -3.201269   0.229615  -1.988560
C   1.804750   1.663761   0.545382
C   2.155245   0.150110   0.374524
O   1.155498   2.181798  -0.398507
O   2.238018   2.189524   1.588227
H   1.918831  -0.380623   1.296458
C   3.642350   0.012441   0.052640
N   1.303415  -0.422866  -0.711357
H   0.371381  -0.756123  -0.313615
H   4.212889   0.472215   0.856972
H   3.881123   0.530342  -0.878956
S   4.207622  -1.725653  -0.202178
H   1.111951   0.330162  -1.383152
H   1.754834  -1.214350  -1.176091
H   3.899346  -2.167342   1.034385
```

```
28
Dimer 10 of the n...z  type
C   1.999786   0.200509   0.834391
C   1.573528  -1.217931   0.408841
N   0.916560   1.166744   0.652719
H   0.821762   1.384218  -0.337196
H   1.150095   2.044314   1.109790
H   2.245896   0.147722   1.898517
C   3.271920   0.606226   0.091603
O   3.325383   1.466389  -0.755435
O   4.320191  -0.142110   0.471520
H   5.100985   0.113793  -0.045665
H   2.399441  -1.911762   0.558515
H   0.727192  -1.544266   1.012246
S   0.958858  -1.353299  -1.322659
H   2.101679  -0.985605  -1.939758
C  -2.228887  -1.651996   0.173784
C  -2.453834  -0.133606   0.480800
O  -1.492380  -2.269447   0.984214
O  -2.823919  -2.080224  -0.833403
H  -3.485184  -0.018161   0.818260
C  -2.207876   0.730863  -0.751294
N  -1.563995   0.266117   1.621431
H  -1.412819  -0.575230   2.189442
H  -2.929421   0.452421  -1.515155
H  -1.212194   0.545262  -1.153903
S  -2.281949   2.547230  -0.441724
H  -0.611863   0.599702   1.276178
H  -1.968164   1.012215   2.184504
H  -3.587936   2.601203  -0.108623
28
Dimer 11 of the n...z  type
C  -2.757754  -0.230289   0.737031
C  -1.705047  -1.346623   0.691312
N  -3.890536  -0.370868  -0.182456
H  -3.641696  -0.948730  -0.981315
H  -4.699269  -0.778132   0.270036
H  -3.138982  -0.186829   1.762513
C  -2.103528   1.145828   0.515725
O  -1.087346   1.498979   1.076456
O  -2.769903   1.932730  -0.319336
H  -3.547141   1.377132  -0.601209
H  -0.874557  -1.090959   1.348315
H  -2.152310  -2.279368   1.035143
S  -1.058768  -1.711561  -0.995737
H  -0.473066  -0.510530  -1.229398
C   1.890646   0.435855  -1.399627
C   2.625459   0.969189  -0.126239
O   0.818962   1.032215  -1.688369
O   2.444483  -0.508740  -1.986603
H   3.417748   1.639103  -0.467654
C   3.239930  -0.127337   0.734643
N   1.649538   1.805093   0.649261
H   2.091121   2.525636   1.219305
H   3.786247   0.293869   1.579754
H   3.932045  -0.691678   0.114156
S   1.999187  -1.277178   1.470100
H   0.996398   2.219491  -0.030953
H   1.059362   1.216856   1.250342
H   1.496584  -1.726820   0.299261
28
Dimer 12 of the n...z  type
C   3.099587  -0.843913   0.121617
C   3.301138   0.452375   0.921256
N   3.510306  -0.807296  -1.285043
H   3.457657   0.139790  -1.652461
H   4.454400  -1.149499  -1.413105
H   3.667702  -1.630357   0.629952
C   1.640691  -1.324518   0.198113
```



```
O   0.989440  -1.338043   1.220887
O   1.156193  -1.762720  -0.960175
H   1.904425  -1.622866  -1.602595
H   2.881055   0.336760   1.919616
H   4.369139   0.651095   1.012226
S   2.590966   1.957052   0.125589
H   1.273324   1.664401   0.302126
C  -1.213041   0.776273  -0.453155
C  -1.798327  -0.617082  -0.063310
O  -0.651048   1.403546   0.487656
O  -1.345617   1.105062  -1.642635
H  -1.129347  -1.365967  -0.485097
C  -3.209762  -0.852866  -0.583703
N  -1.708257  -0.738787   1.432712
H  -1.318384  -1.630552   1.733070
H  -3.563262  -1.850495  -0.319563
H  -3.188006  -0.763246  -1.667491
S  -4.463157   0.314924   0.102498
H  -1.057824   0.023917   1.702586
H  -2.611481  -0.568640   1.882057
H  -3.912151   1.432257  -0.415172
28
Dimer 13 of the n...z  type
C  -1.685630   0.351205   0.414489
C  -1.348187   0.090318  -1.063336
N  -0.807518  -0.385921   1.324863
H  -0.800283  -1.372016   1.067283
H  -1.192793  -0.352975   2.266219
H  -1.521806   1.422091   0.572510
C  -3.163200   0.073954   0.691642
O  -3.574604  -0.698125   1.525503
O  -3.958703   0.806482  -0.106309
H  -4.886465   0.608142   0.098547
H  -2.027701   0.655178  -1.697854
H  -0.339327   0.433630  -1.276834
S  -1.373489  -1.683195  -1.565992
H  -2.693825  -1.890888  -1.381990
C   1.465535   1.873156  -0.583167
C   2.312429   0.841991   0.234008
O   0.632399   2.539993   0.074518
O   1.716907   1.910341  -1.805652
H   3.324127   1.241835   0.324041
C   2.351607  -0.530990  -0.431422
N   1.740573   0.730151   1.612848
H   1.621616   1.660348   2.014583
H   2.706603  -0.410392  -1.451385
H   1.355041  -0.972611  -0.468549
S   3.381945  -1.778570   0.456904
H   0.767840   0.290480   1.545400
H   2.330150   0.159462   2.220872
H   4.566821  -1.160988   0.270837
28
Dimer 14 of the n...z  type
C  -3.047606  -0.948093  -0.331346
C  -3.515801   0.403663   0.238662
N  -3.165579  -2.008700   0.659683
H  -2.533235  -1.811437   1.431627
H  -2.850353  -2.887388   0.259811
H  -3.709869  -1.176592  -1.173697
C  -1.635460  -0.781483  -0.906106
O  -0.659052  -1.348266  -0.454915
O  -1.604682   0.084186  -1.913445
H  -0.647286   0.361705  -2.098265
H  -3.550980   1.150504  -0.552277
H  -4.513982   0.283820   0.660021
S  -2.470491   1.053984   1.618881
H  -1.375331   1.342310   0.873683
C   1.114971   1.194917  -0.909058
C   2.480774   0.725205  -0.349139
```

```
O   0.377483   1.828325  -0.127753
O   0.873775   0.818869  -2.084952
H   3.275586   0.909465  -1.068784
C   2.387204  -0.764437  -0.013145
N   2.785982   1.523558   0.889325
H   3.172429   2.440111   0.659930
H   2.143306  -1.301955  -0.925837
H   1.578355  -0.946817   0.695049
S   3.906849  -1.458925   0.760860
H   1.899944   1.685397   1.386166
H   3.454942   1.031447   1.492072
H   4.727247  -1.249065  -0.289094
28
Dimer 15 of the n...z  type
C   3.088838   0.481130  -0.535630
C   3.533741  -0.912618  -0.076374
N   3.089540   1.535807   0.480690
H   2.846506   1.162577   1.395123
H   3.991692   1.990157   0.551451
H   3.754029   0.791460  -1.349277
C   1.691910   0.437767  -1.165000
O   1.263977  -0.494717  -1.819525
O   0.955067   1.516849  -0.927811
H   1.512991   2.069033  -0.314833
H   3.572302  -1.596736  -0.922511
H   4.529164  -0.840707   0.361196
S   2.452972  -1.642763   1.231002
H   1.458697  -2.012293   0.400331
C  -3.426461  -0.764967  -0.126997
C  -1.864084  -0.685739  -0.226337
O  -4.036754  -0.615617  -1.214448
O  -3.881748  -0.991606   1.010584
H  -1.498936  -1.711637  -0.316834
C  -1.198350  -0.028826   0.975975
N  -1.516159   0.008080  -1.511071
H  -0.598892  -0.283380  -1.884788
H  -0.114005  -0.029246   0.885634
H  -1.470362  -0.595470   1.863016
S  -1.678866   1.734191   1.213395
H  -2.289150  -0.189921  -2.161598
H  -1.483045   1.021831  -1.363079
H  -2.969789   1.474445   1.507782
28
Dimer 16 of the n...z  type
C  -2.024908  -0.578565  -0.684660
C  -1.207675   0.680505  -0.977110
N  -3.440979  -0.534284  -1.068866
H  -3.826875   0.401548  -0.982978
H  -3.582968  -0.846939  -2.021393
H  -1.550950  -1.403614  -1.228753
C  -1.939169  -1.005408   0.788778
O  -0.919602  -0.979013   1.450632
O  -3.078566  -1.460521   1.286532
H  -3.727758  -1.358211   0.538770
H  -0.176219   0.531242  -0.678491
H  -1.233393   0.872346  -2.049635
S  -1.803411   2.189361  -0.087748
H  -0.665329   2.345794   0.627873
C   2.109133   1.295718   0.070440
C   2.688558  -0.014451   0.697514
O   1.391486   1.972660   0.851728
O   2.432074   1.534023  -1.107558
H   3.624664   0.261550   1.190825
C   2.983863  -1.141016  -0.282008
N   1.751068  -0.451667   1.785876
H   2.137139  -1.195734   2.367191
H   3.375511  -2.015332   0.240880
H   3.742594  -0.790531  -0.978131
S   1.531507  -1.740419  -1.247969
```



```
H   1.541022   0.366459   2.365224
H   0.831712  -0.759092   1.410748
H   1.408635  -0.621673  -1.990354
28
Dimer 17 of the n...z  type
C  -3.782794   0.476517   0.377008
C  -3.602234  -0.984675   0.824471
N  -4.677320   0.571266  -0.769253
H  -4.258058   0.080767  -1.555778
H  -4.756190   1.541086  -1.060356
H  -4.237107   1.007723   1.221419
C  -2.402249   1.109416   0.149954
O  -2.051326   1.578117  -0.916501
O  -1.642734   1.048521   1.233911
H  -0.662989   1.210318   0.996837
H  -3.080523  -1.022163   1.778510
H  -4.583204  -1.446731   0.936032
S  -2.687221  -2.041944  -0.386462
H  -1.468703  -1.468636  -0.219016
C   1.199279   0.198321   0.016688
C   2.671243   0.194464  -0.471836
O   0.844226   1.246784   0.634773
O   0.549931  -0.831374  -0.206764
H   2.724429  -0.195880  -1.486029
C   3.516255  -0.657583   0.476926
N   3.184259   1.609724  -0.501237
H   2.853009   2.111011  -1.326701
H   3.106710  -1.665770   0.480968
H   3.464651  -0.262063   1.493351
S   5.309446  -0.703843   0.058254
H   2.815102   2.105406   0.319272
H   4.210393   1.630540  -0.500361
H   5.170522  -1.315083  -1.135983
28
Dimer 18 of the n...z  type
C   3.788120   0.473776  -0.294904
C   3.611969  -0.949970  -0.850089
N   4.633776   0.477141   0.891864
H   4.183017  -0.075270   1.617793
H   4.695265   1.420614   1.262879
H   4.283734   1.056054  -1.080644
C   2.407235   1.112783  -0.082756
O   2.031007   1.544185   0.990775
O   1.680268   1.106186  -1.190211
H   0.694191   1.269372  -0.977167
H   3.127130  -0.914709  -1.823397
H   4.591858  -1.415692  -0.956349
S   2.641503  -2.077433   0.249544
H   1.443960  -1.452976   0.115252
C  -1.189645   0.274231  -0.036772
C  -2.668999   0.275890   0.428466
O  -0.820381   1.309245  -0.667609
O  -0.543537  -0.747923   0.228549
H  -2.716730   0.021767   1.486403
C  -3.475541  -0.725752  -0.399056
N  -3.241053   1.660573   0.276588
H  -2.889036   2.290000   0.999158
H  -3.023752  -1.707323  -0.272728
H  -3.437981  -0.466172  -1.459030
S  -5.266552  -0.790419   0.027708
H  -2.944879   2.051699  -0.623894
H  -4.265859   1.639917   0.331328
H  -5.106889  -1.254926   1.283852
28
Dimer 19 of the n...z  type
C  -3.069538   0.521620   0.562367
C  -3.252468  -0.284720  -0.737487
N  -3.238583   1.948376   0.323945
H  -2.485705   2.274754  -0.277098
```

```
H  -3.138981   2.456823   1.197378
H  -3.851325   0.182665   1.250347
C  -1.722101   0.145892   1.188021
O  -0.779405   0.910463   1.249950
O  -1.693479  -1.118404   1.598357
H  -0.735301  -1.405591   1.740993
H  -3.248246  -1.351548  -0.520470
H  -4.209551  -0.016754  -1.184930
S  -1.983799   0.054453  -2.040656
H  -0.968616  -0.626260  -1.456136
C   1.125550  -1.541221   0.407429
C   2.430112  -0.750814   0.134258
O   0.453000  -1.859760  -0.595610
O   0.846440  -1.707649   1.620967
H   3.226923  -1.069810   0.802582
C   2.134693   0.740209   0.303604
N   2.863949  -1.033588  -1.278605
H   3.365142  -1.919937  -1.350967
H   1.785475   0.907840   1.318665
H   1.330328   1.046850  -0.366848
S   3.559111   1.841632  -0.085486
H   2.013834  -1.115355  -1.853161
H   3.472352  -0.288283  -1.635301
H   4.371170   1.400721   0.897369
28
Dimer 20 of the n...z  type
C   2.719706  -0.073181   0.700860
C   1.940332   1.241646   0.859919
N   3.753836  -0.098121  -0.337106
H   3.583975   0.616715  -1.040082
H   4.681071   0.038768   0.045010
H   3.181061  -0.291870   1.669263
C   1.755030  -1.248788   0.468148
O   0.759866  -1.423057   1.146260
O   2.123092  -2.071648  -0.497365
H   2.966047  -1.666159  -0.844082
H   1.120749   1.085976   1.560585
H   2.603533   2.009928   1.257279
S   1.283371   1.939282  -0.715192
H   0.566904   0.853844  -1.115960
C  -1.744126  -0.068422  -1.442654
C  -2.617372  -0.796159  -0.377788
O  -0.632658  -0.601564  -1.693172
O  -2.236881   0.974875  -1.909539
H  -3.476181  -1.242205  -0.884965
C  -3.133896   0.144499   0.705197
N  -1.822583  -1.932173   0.205786
H  -2.352056  -2.456906   0.903179
H  -3.732964  -0.394253   1.441610
H  -3.766846   0.884877   0.220896
S  -1.820142   1.005854   1.669232
H  -1.542088  -2.569077  -0.541238
H  -0.929661  -1.608697   0.628895
H  -1.273760   1.667946   0.626240
28
Dimer 21 of the n...z  type
C   3.788481   0.473532  -0.292576
C   3.611916  -0.948887  -0.851043
N   4.633278   0.473823   0.894802
H   4.181615  -0.079773   1.619268
H   4.695168   1.416488   1.267817
H   4.284994   1.057258  -1.076671
C   2.407791   1.112840  -0.080050
O   2.030998   1.542088   0.994148
O   1.681650   1.108987  -1.188076
H   0.695429   1.271929  -0.975546
H   3.127945  -0.911185  -1.824687
H   4.591606  -1.414985  -0.957459
S   2.639874  -2.078204   0.245264
```



```
H   1.442967  -1.452225   0.112251
C  -1.189520   0.276318  -0.037909
C  -2.669074   0.278076   0.426714
O  -0.819507   1.311540  -0.667907
O  -0.543780  -0.746046   0.227571
H  -2.716552   0.028077   1.485663
C  -3.474571  -0.727955  -0.396481
N  -3.242794   1.661538   0.269588
H  -2.891401   2.294135   0.989695
H  -3.021780  -1.708470  -0.265675
H  -3.437259  -0.473222  -1.457635
S  -5.265544  -0.792430   0.030575
H  -2.947409   2.049834  -0.632335
H  -4.267571   1.639754   0.324549
H  -5.105456  -1.251098   1.288805
28
Dimer 22 of the n...z  type
C   3.571713  -0.803984   0.053594
C   3.751494   0.695041   0.351560
N   4.097853  -1.148316  -1.260934
H   3.558734  -0.658483  -1.971046
H   3.951556  -2.137615  -1.438133
H   4.147905  -1.343831   0.813851
C   2.099022  -1.186960   0.262570
O   1.400801  -1.652989  -0.618466
O   1.685168  -0.916816   1.490990
H   0.662132  -0.914208   1.538303
H   3.513651   0.900244   1.393302
H   4.787569   0.974830   0.160319
S   2.729544   1.809495  -0.713919
H   1.524242   1.480114  -0.186252
C  -1.200340   0.283589   0.897505
C  -2.722912   0.380262   0.618094
O  -0.477271   1.162338   0.391589
O  -0.866131  -0.720498   1.579683
H  -3.284843   0.236372   1.539082
C  -3.114077  -0.670527  -0.422187
N  -3.026633   1.763818   0.110128
H  -3.060293   2.443374   0.871915
H  -2.855820  -1.649860  -0.024759
H  -2.554325  -0.518727  -1.347479
S  -4.889628  -0.631805  -0.911457
H  -2.264356   2.054679  -0.514475
H  -3.924445   1.789105  -0.385655
H  -5.388813  -0.968375   0.295502
28
Dimer 23 of the n...z  type
C   3.059060  -0.303045  -0.414064
C   2.122119  -1.469089  -0.754385
N   3.799263  -0.389568   0.845651
H   3.268497  -0.905737   1.543238
H   4.694239  -0.847740   0.725570
H   3.785433  -0.231457  -1.231681
C   2.334218   1.053448  -0.467226
O   1.492042   1.332139  -1.301631
O   2.758277   1.923226   0.430842
H   3.414879   1.408423   0.974497
H   1.576841  -1.238007  -1.669709
H   2.716013  -2.365005  -0.934134
S   0.945299  -1.938179   0.588178
H   0.397051  -0.704191   0.798311
C  -1.598838   1.331925   0.688235
C  -2.090709   0.925992  -0.734676
O  -0.455075   0.939696   1.029431
O  -2.420089   2.006454   1.336782
H  -2.240334   1.838710  -1.314138
C  -3.404613   0.149499  -0.679007
N  -1.034870   0.129097  -1.449063
H  -1.265498  -0.008633  -2.434381
```

```
H  -3.761378  -0.100513  -1.679420
H  -4.146178   0.777489  -0.190656
S  -3.275364  -1.461794   0.213378
H  -0.110092   0.596970  -1.388010
H  -0.944254  -0.795969  -1.011762
H  -2.976443  -0.939993   1.420849
28
Dimer 24 of the n...z  type
C  -1.385847   0.200382   0.710297
C  -1.968762  -1.145644   0.265306
N  -2.116433   0.901445   1.769656
H  -3.096393   0.631947   1.784329
H  -1.720271   0.722996   2.683879
H  -0.364443   0.001595   1.045583
C  -1.231704   1.149701  -0.485068
O  -0.766534   0.802837  -1.557911
O  -1.640325   2.383829  -0.259107
H  -1.960308   2.357178   0.685709
H  -1.328142  -1.596557  -0.486947
H  -2.004104  -1.815943   1.123248
S  -3.700586  -1.046920  -0.365141
H  -3.412030  -0.380230  -1.501787
C   2.007658  -1.461952   0.616263
C   2.576491  -0.821470  -0.690583
O   0.924299  -2.080714   0.488003
O   2.704612  -1.287998   1.635778
H   3.375154  -1.474834  -1.050894
C   3.149124   0.577194  -0.497611
N   1.505408  -0.850138  -1.747520
H   1.879900  -0.701065  -2.685035
H   3.416770   1.027302  -1.455026
H   4.051351   0.490641   0.103263
S   2.009728   1.771558   0.326912
H   1.047081  -1.765055  -1.702977
H   0.748836  -0.156820  -1.582442
H   2.035107   1.156348   1.526859
28
Dimer 25 of the n...z  type
C   3.100572  -1.116221   0.040555
C   4.079503  -0.155768   0.727347
N   3.330151  -1.383359  -1.380662
H   3.731177  -0.571755  -1.844358
H   3.950499  -2.171113  -1.521681
H   3.141615  -2.069112   0.580063
C   1.651425  -0.642781   0.207915
O   1.229799  -0.116350   1.222842
O   0.879341  -0.868575  -0.841852
H   1.497025  -1.253996  -1.522249
H   3.842504  -0.073135   1.786915
H   5.092614  -0.543751   0.622792
S   4.111553   1.531955  -0.017225
H   2.887160   1.901913   0.410487
C  -3.119001   1.379756  -0.576402
C  -1.953307   0.390367  -0.250725
O  -3.155589   2.415693   0.133164
O  -3.862992   1.043974  -1.519694
H  -1.127822   0.625430  -0.925353
C  -2.304469  -1.081166  -0.418749
N  -1.459251   0.675888   1.136553
H  -0.462790   0.404963   1.260085
H  -1.443761  -1.710682  -0.193120
H  -2.599772  -1.246689  -1.451944
S  -3.662120  -1.643598   0.696873
H  -1.602023   1.681197   1.297646
H  -2.035466   0.175678   1.820309
H  -4.607836  -0.845546   0.158956
28
Dimer 26 of the n...z  type
C  -3.091455  -0.502937  -0.058736
```



```
C  -3.089072   0.991930  -0.409567
N  -3.330223  -0.840139   1.348318
H  -2.967127  -0.115443   1.962630
H  -4.316265  -0.960120   1.545018
H  -3.871580  -0.981101  -0.661389
C  -1.789803  -1.179904  -0.505627
O  -1.195318  -0.932734  -1.526560
O  -1.340295  -2.104226   0.358368
H  -1.975962  -2.061189   1.123783
H  -2.881076   1.125706  -1.470098
H  -4.074225   1.403684  -0.191508
S  -1.885484   1.995308   0.569307
H  -0.788262   1.805881  -0.215583
C   1.397078   0.492473  -1.121312
C   1.300315  -0.190848   0.276234
O   1.975542  -0.174424  -2.011005
O   0.936245   1.652410  -1.175126
H   0.304289  -0.050262   0.688592
C   2.306941   0.415346   1.252096
N   1.514192  -1.664924   0.072873
H   2.162513  -2.080830   0.742312
H   2.175610  -0.003793   2.251048
H   2.114744   1.486411   1.299862
S   4.076932   0.105740   0.832861
H   0.618359  -2.163762   0.112062
H   1.913290  -1.735303  -0.881765
H   4.024561   0.568711  -0.433425
28
Dimer 27 of the n...z  type
C   3.128844   0.658325   0.142488
C   3.315171  -0.842041  -0.115153
N   2.980223   1.063916   1.543253
H   2.502819   0.344120   2.080745
H   3.875641   1.245378   1.979913
H   3.998678   1.178931  -0.273256
C   1.928793   1.202875  -0.643933
O   1.603574   0.828122  -1.747760
O   1.239980   2.144234   0.011394
H   1.679909   2.197326   0.903188
H   3.478208  -1.024504  -1.176135
H   4.186490  -1.191112   0.438598
S   1.894640  -1.874289   0.456235
H   1.069572  -1.593617  -0.575918
C  -1.508923  -1.283533  -0.333833
C  -1.375270   0.184679   0.187217
O  -1.021445  -1.483187  -1.475474
O  -2.031920  -2.093097   0.453242
H  -0.423468   0.236040   0.721639
C  -2.492267   0.639397   1.114696
N  -1.242880   1.096087  -0.998726
H  -0.745499   1.958715  -0.766611
H  -2.327864   1.662304   1.456740
H  -2.508511  -0.021558   1.977831
S  -4.155321   0.633511   0.315170
H  -0.709212   0.588035  -1.717785
H  -2.168373   1.318025  -1.378526
H  -4.193968  -0.701065   0.120622
28
Dimer 28 of the n...z  type
C  -3.063991  -0.363657  -0.438314
C  -3.019806   0.117149   1.023325
N  -3.242949  -1.807791  -0.507451
H  -2.414358  -2.259438  -0.126877
H  -3.294049  -2.100185  -1.478698
H  -3.930309   0.122658  -0.900442
C  -1.818673   0.141641  -1.175730
O  -0.979452  -0.598602  -1.647807
O  -1.747627   1.470163  -1.194372
H  -0.807514   1.758937  -1.410845
```

```
H  -3.039870   1.204303   1.065355
H  -3.890742  -0.275537   1.547548
S  -1.545285  -0.470353   1.978834
H  -0.686074   0.480946   1.543210
C   1.169494   1.665896  -0.211905
C   2.384369   0.707430  -0.129411
O   0.616980   1.949166   0.872428
O   0.815790   1.979333  -1.375389
H   3.158721   0.993451  -0.838015
C   1.885889  -0.713324  -0.401054
N   2.968154   0.794738   1.254861
H   3.550830   1.625427   1.367342
H   1.435727  -0.734587  -1.389611
H   1.107085  -0.987650   0.313828
S   3.181987  -2.012462  -0.246614
H   2.190279   0.876603   1.922350
H   3.536927  -0.032508   1.467750
H   3.950456  -1.587301  -1.270647
28
Dimer 29 of the n...z  type
C   2.864108  -0.146259   0.769872
C   1.871827   1.016494   0.650367
N   4.251664   0.151293   0.406418
H   4.296033   0.883135  -0.298302
H   4.797956   0.444298   1.206934
H   2.843174  -0.486237   1.810995
C   2.404618  -1.369500  -0.043615
O   1.246898  -1.741060  -0.113861
O   3.383925  -2.004997  -0.660228
H   4.192796  -1.459382  -0.453280
H   0.885959   0.733909   1.009795
H   2.220862   1.843332   1.269352
S   1.728554   1.721264  -1.046601
H   1.304540   0.595730  -1.656381
C  -1.822808   1.249781   0.343545
C  -1.566198  -0.198234  -0.191398
O  -1.428787   1.453692   1.521926
O  -2.365133   2.033299  -0.454830
H  -0.676076  -0.153358  -0.819690
C  -2.710562  -0.770507  -1.014588
N  -1.206908  -1.061323   0.984662
H  -0.320109  -1.555094   0.807686
H  -2.471085  -1.772281  -1.374535
H  -2.869208  -0.118614  -1.870526
S  -4.289677  -0.938912  -0.076102
H  -1.088923  -0.385909   1.764066
H  -1.951924  -1.714593   1.232678
H  -4.440447   0.379520   0.167734
28
Dimer 30 of the n...z  type
C   2.760537   0.205371  -0.295241
C   2.627617  -1.288635  -0.617808
N   2.903321   0.606154   1.102545
H   2.149490   0.208426   1.660388
H   3.794640   0.311240   1.482964
H   3.653641   0.557746  -0.828361
C   1.624596   1.037006  -0.896328
O   0.893807   0.674161  -1.793583
O   1.513521   2.256941  -0.361424
H   2.073903   2.230471   0.451604
H   2.493600  -1.414670  -1.692231
H   3.546214  -1.796870  -0.322244
S   1.281406  -2.168928   0.274307
H   0.284091  -1.369865  -0.144130
C  -1.243560   0.231825   1.472677
C  -2.236958   1.010586   0.544367
O  -0.089336   0.718240   1.543854
O  -1.718923  -0.766809   2.045526
H  -2.784869   1.710729   1.179929
```



```
C  -3.235869   0.128852  -0.191902
N  -1.433894   1.848633  -0.411873
H  -1.976524   2.601062  -0.836247
H  -3.876657   0.719221  -0.848937
H  -3.861322  -0.365800   0.547325
S  -2.450711  -1.156227  -1.258918
H  -0.628006   2.240454   0.092769
H  -1.024579   1.274370  -1.162338
H  -1.991465  -1.897830  -0.230986
28
Dimer 31 of the n...z  type
C   2.131084   0.100912   0.632298
C   2.448681  -1.403299   0.621628
N   0.981211   0.402861  -0.210808
H   1.196216   0.195702  -1.183407
H   0.738188   1.389908  -0.156893
H   1.879246   0.363460   1.666041
C   3.363118   0.931569   0.268287
O   3.401238   1.752087  -0.616379
O   4.407867   0.630590   1.059578
H   5.169647   1.175215   0.803929
H   3.227959  -1.631289   1.345701
H   1.548052  -1.954983   0.891579
S   2.946441  -2.060467  -1.029352
H   4.166883  -1.486680  -1.064257
C  -2.153699   1.792642  -0.316579
C  -2.423322   0.255430  -0.223158
O  -1.361251   2.269982   0.531254
O  -2.777132   2.370282  -1.230416
H  -2.200892  -0.193537  -1.191930
C  -3.878102  -0.009658   0.155070
N  -1.481496  -0.358141   0.763527
H  -0.480682  -0.221477   0.404981
H  -4.517959   0.486316  -0.571484
H  -4.098039   0.401552   1.142864
S  -4.324413  -1.798036   0.263345
H  -1.540807   0.138050   1.654405
H  -1.685481  -1.348843   0.911393
H  -4.145121  -2.079503  -1.043427
28
Dimer 32 of the n...z  type
C  -3.662418   0.745009   0.594614
C  -4.213391  -0.640337   0.958968
N  -4.617495   1.499100  -0.208246
H  -4.776390   1.006285  -1.084150
H  -4.210725   2.393197  -0.466300
H  -3.514151   1.276447   1.543145
C  -2.261908   0.639485  -0.036196
O  -1.946437   1.244451  -1.044836
O  -1.460104  -0.160935   0.645213
H  -0.514215  -0.195133   0.242873
H  -3.575803  -1.123453   1.695766
H  -5.215058  -0.528195   1.374150
S  -4.407940  -1.779385  -0.484707
H  -3.090286  -1.984054  -0.688905
C   1.709465   0.540729   0.037379
C   3.171215   0.294018  -0.424641
O   1.482910   1.577134   0.684509
O   0.915249  -0.388228  -0.272818
H   3.190501   0.028271  -1.480065
C   3.796244  -0.817801   0.419659
N   3.943994   1.574760  -0.263126
H   3.770334   2.215339  -1.038320
H   3.204045  -1.719532   0.279246
H   3.772433  -0.553973   1.478933
S   5.567709  -1.154973   0.041013
H   3.618445   2.044953   0.590823
H   4.952909   1.393401  -0.214465
H   5.377989  -1.566812  -1.229264
```

```
28
Dimer 33 of the n...z  type
C   1.530220   0.249092  -0.083370
C   2.629119   0.987680  -0.853412
N   1.198752   0.772704   1.242118
H   1.987612   1.254099   1.663512
H   0.403699   1.401855   1.164887
H   0.621978   0.310541  -0.688423
C   1.842354  -1.252368   0.023642
O   2.302281  -1.910156  -0.883362
O   1.543261  -1.774913   1.213178
H   1.243991  -0.990101   1.746711
H   2.761075   0.540922  -1.837678
H   2.342007   2.032177  -0.975997
S   4.259230   1.023131   0.012711
H   4.511850  -0.297569  -0.095267
C  -2.430094   1.461714  -0.144966
C  -2.878849   0.263249   0.758883
O  -3.347533   2.004301  -0.804257
O  -1.218120   1.751600  -0.090533
H  -2.994774   0.653450   1.772038
C  -1.902133  -0.904097   0.785727
N  -4.245934  -0.156012   0.289784
H  -4.860290  -0.473987   1.038697
H  -2.251965  -1.695326   1.450483
H  -0.951676  -0.537341   1.160096
S  -1.659784  -1.716093  -0.851124
H  -4.632712   0.679679  -0.184502
H  -4.168482  -0.898738  -0.413712
H  -1.246568  -0.612436  -1.506728
28
Dimer 34 of the n...z  type
C   2.056182  -0.331860   0.782479
C   2.194792   1.170437   0.525095
N   3.213604  -1.020681   1.357194
H   4.082782  -0.639105   0.991688
H   3.231515  -0.938410   2.366442
H   1.207104  -0.464053   1.461950
C   1.621730  -1.086157  -0.477516
O   0.914253  -0.602358  -1.342281
O   2.057132  -2.335542  -0.538236
H   2.660658  -2.422055   0.245857
H   1.247116   1.574507   0.176539
H   2.456889   1.667579   1.459169
S   3.536007   1.606745  -0.665312
H   2.928221   1.098584  -1.756580
C  -1.225421   0.125600   0.790471
C  -1.819758   0.493641  -0.600776
O  -1.131911  -1.098220   1.048056
O  -0.916255   1.105625   1.497625
H  -1.148737   1.199408  -1.089632
C  -3.206916   1.117007  -0.477784
N  -1.841841  -0.743143  -1.455148
H  -0.863698  -1.001781  -1.657862
H  -3.593058   1.413355  -1.454975
H  -3.117222   2.008172   0.141224
S  -4.491080  -0.003448   0.230470
H  -2.261153  -1.509368  -0.920317
H  -2.351441  -0.616731  -2.330360
H  -3.909544  -0.164136   1.436879
28
Dimer 35 of the n...z  type
C  -3.089930   0.508328   0.068296
C  -3.087461  -0.989325   0.406985
N  -3.337530   0.857359  -1.334324
H  -2.977823   0.138123  -1.956966
H  -4.324799   0.978406  -1.524044
H  -3.865294   0.982798   0.679999
C  -1.784295   1.179731   0.511619
```



```
O  -1.182173   0.922470   1.525815
O  -1.339494   2.110857  -0.346996
H  -1.980186   2.075665  -1.108552
H  -2.871053  -1.132077   1.464654
H  -4.075142  -1.397476   0.193684
S  -1.893716  -1.986927  -0.589931
H  -0.791849  -1.814293   0.191555
C   1.400837  -0.526095   1.114643
C   1.300886   0.191649  -0.265322
O   1.971500   0.122990   2.022527
O   0.947142  -1.689807   1.138934
H   0.303547   0.060349  -0.677260
C   2.303782  -0.389064  -1.260487
N   1.514294   1.660133  -0.024764
H   0.623858   2.166306  -0.079579
H   2.164591   0.051063  -2.249370
H   2.115985  -1.459696  -1.330022
S   4.074676  -0.080177  -0.844835
H   1.887689   1.705940   0.942213
H   2.187096   2.084183  -0.664226
H   4.031956  -0.571495   0.411134
28
Dimer 36 of the n...z  type
C  -3.711539  -0.752091  -0.844645
C  -4.171553   0.712347  -0.821082
N  -4.710410  -1.630004  -0.248510
H  -4.832445  -1.379218   0.729898
H  -4.364656  -2.584912  -0.246558
H  -3.600716  -1.022691  -1.902088
C  -2.306576  -0.897186  -0.233492
O  -2.035178  -1.730287   0.611435
O  -1.447348  -0.026383  -0.738019
H  -0.510802  -0.132599  -0.338357
H  -3.504506   1.331471  -1.416664
H  -5.179130   0.776592  -1.232098
S  -4.289066   1.440902   0.874723
H  -2.960877   1.499806   1.103489
C   1.730210   0.697666  -0.036486
C   3.185334   0.458147   0.451129
O   0.929154  -0.257187   0.206802
O   1.519538   1.753379  -0.641522
H   3.570282   1.354944   0.932469
C   4.059257   0.070582  -0.742993
N   3.180869  -0.644518   1.477058
H   2.896263  -0.299767   2.394778
H   4.018856   0.880924  -1.468181
H   3.676437  -0.834988  -1.218005
S   5.810342  -0.305447  -0.310551
H   2.483269  -1.342665   1.189162
H   4.108341  -1.074018   1.568173
H   6.125934   0.928541   0.133219
28
Dimer 37 of the n...z  type
C   1.740643   0.320332   0.169373
C   2.243375  -0.804558   1.085285
N   0.670235  -0.146280  -0.711138
H   1.001613  -0.941687  -1.255157
H   0.447019   0.577071  -1.389971
H   1.326633   1.100018   0.819487
C   2.881712   0.983897  -0.602960
O   2.858935   1.218637  -1.787346
O   3.910040   1.294511   0.204254
H   4.602210   1.734775  -0.314786
H   2.938086  -0.406999   1.821604
H   1.398685  -1.245799   1.614380
S   3.045096  -2.207899   0.194431
H   4.166314  -1.543833  -0.154893
C  -3.692445  -1.012017  -0.386260
C  -2.794594  -0.061558   0.479509
```

```
O  -3.324425  -2.213363  -0.427312
O  -4.682481  -0.472877  -0.916744
H  -3.351083   0.166114   1.389869
C  -2.453211   1.228555  -0.255629
N  -1.562151  -0.819674   0.879155
H  -1.290181  -0.641108   1.843344
H  -3.379999   1.740341  -0.501593
H  -1.932874   1.012954  -1.189909
S  -1.334497   2.363146   0.675101
H  -1.803645  -1.814468   0.746694
H  -0.731894  -0.593726   0.251812
H  -2.166481   2.590912   1.711761
28
Dimer 38 of the n...z  type
C  -2.552058  -0.596008  -0.815990
C  -2.172690   0.887836  -0.638747
N  -3.907099  -0.859138  -0.362300
H  -3.968915  -0.716751   0.642067
H  -4.149174  -1.830120  -0.533136
H  -2.491536  -0.800090  -1.889377
C  -1.474890  -1.450067  -0.138156
O  -1.623510  -2.010218   0.926593
O  -0.333467  -1.457447  -0.828950
H   0.464737  -1.874985  -0.321809
H  -1.180586   1.073595  -1.049331
H  -2.887049   1.501868  -1.187226
S  -2.259325   1.519911   1.094611
H  -1.480218   0.581409   1.670259
C   2.674985  -1.250533   0.479502
C   2.510817   0.108005  -0.267011
O   1.838846  -2.166258   0.200551
O   3.589644  -1.288160   1.307569
H   3.490136   0.571375  -0.367396
C   1.566236   1.012352   0.525163
N   1.984544  -0.086953  -1.669764
H   2.585595  -0.711040  -2.209745
H   1.973676   1.141582   1.526309
H   0.585349   0.547366   0.618120
S   1.254909   2.657366  -0.243112
H   1.035068  -0.489423  -1.656698
H   1.931071   0.813859  -2.155756
H   2.533890   3.084708  -0.200489
28
Dimer 39 of the n...z  type
C  -1.767046   0.606682  -0.223245
C  -1.480297  -0.733288  -0.909752
N  -1.104138   0.859342   1.055328
H  -0.983976   0.008153   1.596503
H  -0.210394   1.331252   0.939213
H  -1.435864   1.382567  -0.920729
C  -3.280801   0.824327  -0.049639
O  -4.113501   0.471726  -0.856478
O  -3.598558   1.469076   1.073318
H  -2.724325   1.573628   1.537860
H  -2.057959  -0.805913  -1.829949
H  -0.423189  -0.783941  -1.163424
S  -1.835775  -2.210492   0.136462
H  -3.171594  -2.031336   0.199372
C   1.769561   1.346430  -0.552026
C   3.106731   0.540824  -0.559613
O   1.600196   2.111544   0.435844
O   1.008624   1.126821  -1.507773
H   3.727916   0.927720  -1.368084
C   2.881857  -0.954693  -0.767849
N   3.818150   0.825259   0.736960
H   4.825734   0.943678   0.638793
H   3.828088  -1.487616  -0.868078
H   2.304643  -1.081223  -1.681189
S   2.013936  -1.773748   0.640803
```



```
H   3.371614   1.682853   1.108021
H   3.631479   0.075710   1.413714
H   0.912145  -0.998438   0.619461
28
Dimer 40 of the n...z  type
C  -2.144323   0.260793   0.782745
C  -1.986634  -1.221169   0.428010
N  -3.438687   0.667904   1.334807
H  -4.198898   0.155446   0.894140
H  -3.484706   0.503782   2.333181
H  -1.365387   0.503169   1.514005
C  -1.814109   1.170642  -0.407918
O  -0.985880   0.905736  -1.253977
O  -2.498806   2.311113  -0.418883
H  -3.137637   2.221545   0.334678
H  -0.965758  -1.436447   0.121807
H  -2.208770  -1.821557   1.310320
S  -3.149074  -1.809500  -0.878695
H  -2.583317  -1.121514  -1.891273
C   1.314695  -0.207954   0.941623
C   1.740445  -0.148785  -0.561036
O   0.928744   0.883853   1.435520
O   1.375677  -1.332401   1.468708
H   0.949538  -0.629195  -1.135314
C   3.065361  -0.841219  -0.849048
N   1.745181   1.301105  -0.961422
H   1.254040   1.474432  -1.836753
H   3.306782  -0.797543  -1.912030
H   2.970171  -1.883545  -0.553007
S   4.507426  -0.085806   0.019072
H   1.243227   1.767133  -0.179354
H   2.694223   1.680489  -1.002949
H   4.067012  -0.337070   1.269364
28
Dimer 41 of the n...z  type
C  -1.240270   0.067063   0.011442
C  -2.064286  -1.071108  -0.590511
N  -1.012654   0.024139   1.456161
H  -1.846055  -0.303137   1.938841
H  -0.239313  -0.595664   1.691031
H  -0.264749   0.030598  -0.479676
C  -1.791143   1.448364  -0.367165
O  -2.314499   1.701050  -1.429389
O  -1.599573   2.374219   0.575935
H  -1.214834   1.866875   1.337412
H  -2.122332  -0.960663  -1.672577
H  -1.566074  -2.011583  -0.358123
S  -3.773250  -1.200677   0.091407
H  -4.233857  -0.053860  -0.449156
C   1.778729  -1.467411   0.441749
C   2.973964  -0.791039  -0.306401
O   1.000322  -2.132669  -0.283025
O   1.722166  -1.263016   1.671081
H   3.831524  -1.462723  -0.229166
C   3.359178   0.581942   0.229334
N   2.598900  -0.734458  -1.764125
H   3.397177  -0.772919  -2.397866
H   4.184547   1.007692  -0.342955
H   3.672618   0.468210   1.264147
S   1.989850   1.818240   0.148503
H   1.962353  -1.533811  -1.918759
H   2.065173   0.119434  -1.959431
H   1.237508   1.241417   1.107335
28
Dimer 42 of the n...z  type
C  -2.724287   0.916160  -0.216008
C  -3.299829  -0.484926  -0.514284
N  -1.975807   1.547674  -1.289765
H  -1.234826   0.941358  -1.633131
```

```
H  -2.590313   1.754638  -2.069394
H  -3.585537   1.555647   0.010337
C  -1.946275   0.878060   1.107877
O  -2.439128   0.390883   2.104438
O  -0.723937   1.403377   1.154907
H  -0.329438   1.630866   0.271963
H  -3.889386  -0.841929   0.329205
H  -3.945148  -0.420424  -1.391169
S  -2.030033  -1.749881  -0.945033
H  -1.464871  -1.845513   0.277267
C   2.135555   1.206496  -0.478725
C   2.648345  -0.220816  -0.839872
O   2.931683   1.944808   0.134443
O   0.968835   1.452257  -0.870846
H   2.880279  -0.224602  -1.906571
C   1.637446  -1.323272  -0.544319
N   3.943125  -0.444727  -0.104757
H   4.583980  -1.072905  -0.589722
H   2.004544  -2.293963  -0.880065
H   0.719675  -1.089607  -1.079534
S   1.261151  -1.530784   1.249730
H   4.372051   0.481332   0.036251
H   3.746981  -0.827584   0.828098
H   0.662232  -0.333448   1.422937
28
Dimer 43 of the n...z  type
C  -1.762194  -0.747807  -0.262426
C  -1.224166   0.416495  -1.096173
N  -2.998222  -1.373403  -0.742275
H  -3.619106  -0.681766  -1.155434
H  -2.806890  -2.082019  -1.440188
H  -0.975924  -1.508573  -0.241501
C  -1.946471  -0.361229   1.208951
O  -1.226920   0.412544   1.805193
O  -2.965696  -0.979882   1.804136
H  -3.400898  -1.489920   1.072711
H  -0.265139   0.749432  -0.711318
H  -1.073099   0.077911  -2.119902
S  -2.362176   1.866320  -1.193230
H  -2.233578   2.234489   0.098008
C   1.612835  -1.410257  -0.349307
C   2.947334  -0.595938  -0.278513
O   1.121657  -1.519793  -1.502208
O   1.197081  -1.871571   0.728151
H   3.764174  -1.319817  -0.321729
C   3.104834   0.264160   0.967874
N   3.025691   0.222703  -1.538981
H   3.975642   0.376781  -1.875650
H   4.083888   0.744518   0.988885
H   3.011655  -0.383068   1.836792
S   1.870035   1.628863   1.088728
H   2.458329  -0.298594  -2.230667
H   2.572498   1.132114  -1.392119
H   0.785351   0.854804   1.321627
28
Dimer 44 of the n...z  type
C  -3.034135  -0.237847   0.718198
C  -2.092940  -1.400635   0.382069
N  -4.337594  -0.239492   0.054217
H  -4.276551  -0.657169  -0.871098
H  -5.033054  -0.745783   0.588291
H  -3.199268  -0.255949   1.801214
C  -2.360949   1.119344   0.457166
O  -1.181626   1.340754   0.669424
O  -3.175207   2.046539  -0.008688
H  -4.042756   1.571344  -0.127122
H  -1.184474  -1.332919   0.978597
H  -2.585265  -2.341299   0.627742
S  -1.661821  -1.535765  -1.405438
```



```
H  -1.048861  -0.340474  -1.514429
C   2.948735  -1.181585   0.396620
C   1.618572  -0.386160   0.190756
O   3.220946  -1.467392   1.586964
O   3.567844  -1.463381  -0.649108
H   0.843867  -1.125525  -0.022993
C   1.649189   0.631510  -0.941118
N   1.238949   0.276812   1.483321
H   0.257016   0.599355   1.456704
H   0.700539   1.166113  -1.008207
H   1.832143   0.104426  -1.874159
S   2.933964   1.936108  -0.714026
H   1.417584  -0.376858   2.251084
H   1.841175   1.092050   1.636261
H   3.983489   1.091870  -0.795882
28
Dimer 45 of the n...z  type
C  -2.036465  -0.858763  -0.032720
C  -3.157177  -0.333013  -0.934860
N  -2.444406  -1.454499   1.241415
H  -3.273639  -0.996126   1.610822
H  -2.642343  -2.442875   1.145792
H  -1.470693  -1.608139  -0.593763
C  -0.989100   0.226014   0.244967
O  -0.644122   1.049049  -0.581191
O  -0.470477   0.180352   1.464662
H  -0.957764  -0.563240   1.907839
H  -2.740390   0.043076  -1.868008
H  -3.849790  -1.143917  -1.160723
S  -4.182758   0.992717  -0.160221
H  -3.218635   1.935795  -0.154559
C   1.931860  -1.455412  -0.114745
C   2.737317  -0.404462  -0.954930
O   0.869308  -1.853105  -0.654515
O   2.447781  -1.801459   0.963272
H   3.401509  -0.975499  -1.608956
C   3.578474   0.569090  -0.142384
N   1.764182   0.304087  -1.856313
H   2.214423   0.796373  -2.627962
H   4.178069   1.206522  -0.794490
H   4.246947  -0.018958   0.482676
S   2.601394   1.721398   0.912290
H   1.111572  -0.403819  -2.212921
H   1.182300   0.965881  -1.319118
H   1.876968   0.771248   1.536456
28
Dimer 46 of the n...z  type
C   2.614365  -0.398568   0.778086
C   2.735814  -1.165547  -0.548774
N   2.015371  -1.223428   1.816771
H   1.091912  -1.530813   1.521316
H   1.879652  -0.678418   2.662567
H   3.637204  -0.141924   1.082364
C   1.898412   0.944103   0.579986
O   1.040221   1.370675   1.325423
O   2.312668   1.589681  -0.512571
H   1.666649   2.283144  -0.737198
H   3.324637  -0.596208  -1.264111
H   3.234821  -2.115052  -0.356829
S   1.122408  -1.610057  -1.340026
H   0.745406  -0.349799  -1.678016
C  -1.137074   1.712697  -0.694569
C  -1.433736   0.418306   0.117615
O  -1.760076   2.734491  -0.334284
O  -0.316650   1.572128  -1.631566
H  -0.532568  -0.173219   0.214067
C  -2.511130  -0.417377  -0.556715
N  -1.824173   0.821184   1.509212
H  -0.978600   1.103767   2.014730
```

```
H  -2.210103  -0.588690  -1.589402
H  -3.473512   0.097231  -0.559432
S  -2.789550  -2.039082   0.279416
H  -2.442264   1.639068   1.448416
H  -2.279720   0.058893   2.019086
H  -1.536837  -2.503537   0.080540
28
Dimer 47 of the n...z  type
C  -2.242481  -1.166596  -0.232967
C  -3.267131  -0.109197   0.197829
N  -1.432285  -1.777582   0.809865
H  -0.983372  -1.091182   1.413910
H  -1.970566  -2.416151   1.381921
H  -2.808993  -1.971068  -0.719582
C  -1.313599  -0.646658  -1.340858
O  -1.635714   0.180176  -2.161330
O  -0.103086  -1.219498  -1.349698
H  -0.053139  -1.807706  -0.553740
H  -3.873659   0.203202  -0.651421
H  -3.923677  -0.543683   0.952005
S  -2.532091   1.389868   0.979258
H  -2.123187   1.959214  -0.171824
C   1.814921  -0.894840   1.068185
C   2.817452  -0.031517   0.227583
O   1.767153  -2.099339   0.714214
O   1.193830  -0.316426   1.979825
H   3.818768  -0.268335   0.595479
C   2.617585   1.474478   0.281501
N   2.756254  -0.551754  -1.185505
H   3.530485  -0.231770  -1.768329
H   3.400931   1.991631  -0.275037
H   2.673206   1.780996   1.323898
S   1.022121   2.049184  -0.434580
H   2.751218  -1.577117  -1.129982
H   1.866291  -0.275471  -1.620521
H   0.245040   1.421242   0.471252
28
Dimer 48 of the n...z  type
C   2.694552  -0.630902  -0.882602
C   1.794402  -1.588744  -0.077304
N   4.057026  -0.621975  -0.373628
H   4.085505  -0.169367   0.535774
H   4.648548  -0.066055  -0.983490
H   2.701025  -1.017925  -1.905869
C   2.011916   0.743793  -0.912548
O   2.401929   1.687697  -0.252910
O   0.910199   0.743541  -1.653276
H   0.283656   1.492310  -1.364320
H   0.822480  -1.676567  -0.559674
H   2.261856  -2.572134  -0.049890
S   1.543652  -1.120842   1.708521
H   1.721455   0.211906   1.586390
C  -1.363982   1.766655   0.384217
C  -1.605585   0.237424   0.204505
O  -1.786586   2.270351   1.438739
O  -0.805690   2.338936  -0.592079
H  -0.747148  -0.230146  -0.266149
C  -2.850740   0.023948  -0.654783
N  -1.763992  -0.384514   1.559718
H  -0.836108  -0.646615   1.928737
H  -2.693929   0.517095  -1.612502
H  -3.727082   0.467527  -0.178356
S  -3.277247  -1.748585  -0.923626
H  -2.187003   0.316643   2.182487
H  -2.338794  -1.232253   1.522382
H  -2.150390  -2.075392  -1.589167
28
Dimer 49 of the n...z  type
C  -1.971695  -0.782687  -0.704265
```



```
C  -2.169229  -1.344195   0.718749
N  -0.700004  -0.081147  -0.840656
H  -0.772386   0.881275  -0.509916
H  -0.426291  -0.030398  -1.817149
H  -1.977716  -1.643997  -1.377015
C  -3.168828   0.093193  -1.072263
O  -3.134773   1.298237  -1.157689
O  -4.280237  -0.641616  -1.240522
H  -5.029837  -0.052151  -1.423055
H  -3.128373  -1.855838   0.786272
H  -1.377820  -2.066295   0.922064
S  -2.031340  -0.101806   2.075075
H  -2.960363   0.762665   1.617393
C   2.004866   1.896383   0.071197
C   2.451097   0.441521   0.439036
O   0.842561   2.223280   0.411587
O   2.875774   2.571097  -0.515146
H   3.247507   0.511810   1.181631
C   2.944129  -0.314865  -0.791068
N   1.305828  -0.290500   1.068573
H   0.894915   0.267370   1.818550
H   3.747195   0.258195  -1.247582
H   2.140917  -0.419478  -1.522833
S   3.514166  -2.038335  -0.453162
H   0.537010  -0.407848   0.341986
H   1.590772  -1.203221   1.427006
H   4.579832  -1.702394   0.302450
28
Dimer 50 of the n...z  type
C   2.064567  -0.779407  -0.413137
C   1.720524   0.432845  -1.284544
N   1.034981  -1.235959   0.523346
H   0.619152  -0.436227   0.996025
H   0.283201  -1.677122  -0.000342
H   2.271076  -1.610328  -1.099185
C   3.380844  -0.587757   0.354187
O   4.315583   0.065206  -0.052072
O   3.420319  -1.242885   1.517992
H   2.517067  -1.637527   1.612628
H   2.540671   0.647328  -1.968590
H   0.813697   0.218826  -1.848114
S   1.317172   1.959311  -0.336757
H   2.529890   2.117246   0.233954
C  -1.802734  -0.375923  -1.294374
C  -3.086051  -0.448940  -0.402697
O  -1.197341  -1.471377  -1.444975
O  -1.534141   0.740229  -1.766972
H  -3.938500  -0.550053  -1.077857
C  -3.299062   0.760883   0.496401
N  -3.008587  -1.726189   0.390940
H  -3.918326  -2.136295   0.601619
H  -4.224943   0.667903   1.065409
H  -3.361675   1.644314  -0.134399
S  -1.959941   0.999640   1.742901
H  -2.432755  -2.369508  -0.177042
H  -2.507197  -1.563861   1.271542
H  -0.995705   1.337534   0.859253
28
Dimer 51 of the n...z  type
C  -2.420711  -0.330523   0.835087
C  -1.300828   0.708275   0.695603
N  -2.086461  -1.646827   0.273026
H  -1.114807  -1.693680  -0.046744
H  -2.219685  -2.378735   0.960820
H  -2.657969  -0.401621   1.901670
C  -3.698399   0.191658   0.150717
O  -4.228391   1.244692   0.435968
O  -4.150289  -0.616961  -0.801875
H  -3.482290  -1.367143  -0.786196
```

```
H  -1.584043   1.627888   1.204683
H  -0.399130   0.309590   1.157831
S  -0.853985   1.125610  -1.049269
H  -1.884012   1.970136  -1.264133
C   1.571908  -1.308864   0.375216
C   2.994915  -0.946453  -0.162618
O   0.765446  -1.711870  -0.504669
O   1.392960  -1.171995   1.597045
H   3.611950  -1.841708  -0.062080
C   3.666196   0.210817   0.565316
N   2.854224  -0.677939  -1.636888
H   3.685113  -0.912146  -2.179895
H   4.675109   0.380997   0.187296
H   3.723687  -0.043848   1.620903
S   2.780568   1.815111   0.365027
H   2.039073  -1.234973  -1.942750
H   2.626509   0.311766  -1.789497
H   1.645655   1.423114   0.976032
28
Dimer 52 of the n...z  type
C   2.642540   0.343757   0.763484
C   1.460910   1.257557   0.422416
N   3.933787   0.687183   0.160403
H   3.806859   1.132190  -0.744937
H   4.468295   1.314329   0.748816
H   2.758340   0.363699   1.852629
C   2.316216  -1.127624   0.442621
O   1.234156  -1.632659   0.642053
O   3.347130  -1.810718  -0.052400
H   4.073258  -1.137532  -0.126322
H   0.558044   0.919586   0.927672
H   1.682168   2.273638   0.749862
S   1.126012   1.399155  -1.388970
H   0.762833   0.113200  -1.579021
C  -2.196310   0.223876   1.437980
C  -2.944002   0.482085   0.085396
O  -1.522125   1.196808   1.859707
O  -2.376656  -0.895513   1.950537
H  -3.940011   0.850804   0.344022
C  -3.095931  -0.739832  -0.807913
N  -2.246392   1.618414  -0.611664
H  -2.832064   2.090670  -1.300736
H  -3.638494  -0.493026  -1.722015
H  -3.666377  -1.482272  -0.254291
S  -1.504365  -1.489091  -1.355375
H  -1.950535   2.274163   0.123964
H  -1.379930   1.308021  -1.076239
H  -1.013222  -1.721767  -0.121818
28
Dimer 53 of the n...z  type
C   2.328416  -1.022648  -0.626855
C   3.162895  -0.520360   0.561610
N   2.225865  -0.173895  -1.811096
H   1.977098   0.777799  -1.547180
H   3.090451  -0.161580  -2.338337
H   2.782266  -1.970915  -0.942624
C   0.910061  -1.439610  -0.206252
O   0.623018  -1.877406   0.883901
O   0.015142  -1.340304  -1.196529
H   0.513859  -0.882814  -1.922900
H   3.022074  -1.198484   1.403120
H   4.217505  -0.531306   0.283841
S   2.835929   1.211531   1.096504
H   1.476353   1.152012   1.180923
C  -0.802504   1.389635  -0.043526
C  -2.317621   1.185620  -0.378016
O  -0.075226   1.591178  -1.044798
O  -0.485246   1.348894   1.162603
H  -2.727812   2.178130  -0.578777
```



```
C  -3.153377   0.514979   0.703062
N  -2.367846   0.435175  -1.681954
H  -3.263354   0.514728  -2.164898
H  -4.214834   0.554230   0.454181
H  -2.991357   1.063645   1.628451
S  -2.775629  -1.267124   0.967421
H  -1.609741   0.811645  -2.266243
H  -2.166930  -0.558497  -1.510778
H  -1.465244  -1.112647   1.248208
28
Dimer 54 of the n...z  type
C   2.663098  -1.218696   0.153297
C   3.384186  -0.051341   0.839134
N   2.961765  -1.433278  -1.263893
H   3.174287  -0.552991  -1.727152
H   3.745779  -2.060463  -1.394294
H   2.922092  -2.131401   0.701479
C   1.140191  -1.092180   0.311382
O   0.612280  -0.677032   1.326979
O   0.449701  -1.483158  -0.744809
H   1.146412  -1.714492  -1.417575
H   3.092064   0.000837   1.886858
H   4.461031  -0.209124   0.780843
S   3.078684   1.583908   0.041505
H   1.757010   1.640478   0.311911
C  -2.992204  -0.849132  -0.271549
C  -2.906977   0.449649   0.602406
O  -2.881011  -1.917626   0.375676
O  -3.199897  -0.678176  -1.489431
H  -3.908196   0.609583   1.012478
C  -2.497628   1.715964  -0.134276
N  -2.017013   0.152271   1.777001
H  -2.022931   0.895002   2.476601
H  -2.531858   2.582697   0.528394
H  -3.214026   1.867520  -0.938945
S  -0.796782   1.703215  -0.838643
H  -2.344371  -0.721094   2.199472
H  -1.033763  -0.035015   1.485961
H  -0.933029   0.545771  -1.516018
28
Dimer 55 of the n...z  type
C  -2.490413   0.060604   0.919555
C  -2.504836   1.525241   0.463526
N  -1.292463  -0.382514   1.634395
H  -0.461614   0.175674   1.414684
H  -1.427269  -0.356300   2.637162
H  -3.360603  -0.080176   1.570608
C  -2.740935  -0.891472  -0.265916
O  -3.548088  -0.675510  -1.143632
O  -1.991807  -1.991613  -0.228479
H  -1.417349  -1.860313   0.578096
H  -3.493621   1.796008   0.096712
H  -2.261115   2.163982   1.312391
S  -1.270150   1.935524  -0.848711
H  -1.951363   1.365146  -1.862912
C   2.225167   1.013158   0.632736
C   2.046556   0.363523  -0.780765
O   3.355855   1.513846   0.839965
O   1.230235   0.990744   1.385269
H   1.643208   1.141135  -1.432568
C   1.135255  -0.851183  -0.851095
N   3.429634   0.045407  -1.285411
H   3.514840   0.081016  -2.301141
H   1.137873  -1.277174  -1.855980
H   0.123965  -0.527553  -0.635539
S   1.609438  -2.223488   0.278929
H   4.050459   0.738781  -0.834606
H   3.721651  -0.882440  -0.962261
H   1.397815  -1.529569   1.415754
```

```
28
Dimer 56 of the n...z  type
C  -2.701773  -0.585845   1.057672
C  -3.255053  -1.439096  -0.092717
N  -3.762010  -0.148253   1.954179
H  -4.414683   0.439490   1.440827
H  -3.372553   0.438447   2.685794
H  -2.017649  -1.234038   1.620575
C  -1.824131   0.559376   0.532660
O  -1.879483   1.697553   0.942230
O  -0.979274   0.153264  -0.421881
H  -0.329203   0.884005  -0.697469
H  -2.444627  -1.902475  -0.650833
H  -3.890958  -2.220656   0.322589
S  -4.329150  -0.509095  -1.274633
H  -3.336411   0.202399  -1.847145
C   1.997716   1.595772  -0.310032
C   2.466630   0.165773   0.105965
O   0.980529   1.663260  -1.065557
O   2.676297   2.523270   0.143497
H   2.259734   0.036598   1.169038
C   3.957430  -0.028623  -0.168636
N   1.669161  -0.892908  -0.613133
H   0.681909  -0.888838  -0.325699
H   4.502013   0.754580   0.352107
H   4.166003   0.064400  -1.236473
S   4.607186  -1.684993   0.315554
H   1.678213  -0.721864  -1.621278
H   2.069832  -1.820334  -0.433302
H   4.420265  -1.541100   1.643423
28
Dimer 57 of the n...z  type
C   2.059782   0.615127  -0.543324
C   3.202902  -0.164846  -1.203048
N   2.433585   1.813570   0.211139
H   3.367065   1.724017   0.604105
H   2.406221   2.645649  -0.365042
H   1.364547   0.909665  -1.335307
C   1.223513  -0.294300   0.365721
O   0.887239  -1.421391   0.086237
O   0.851581   0.288502   1.513621
H   1.248214   1.201597   1.462596
H   2.803546  -1.012497  -1.758058
H   3.731760   0.490774  -1.894693
S   4.480990  -0.767023  -0.016205
H   3.701748  -1.687582   0.587909
C  -3.280358  -0.951734  -0.545196
C  -2.980982  -0.237522   0.815639
O  -2.524891  -1.913805  -0.830677
O  -4.224965  -0.465510  -1.194559
H  -3.834029  -0.393573   1.476338
C  -2.768283   1.263405   0.644602
N  -1.786448  -0.917682   1.434273
H  -2.025349  -1.423914   2.285250
H  -2.493218   1.732248   1.591098
H  -3.705707   1.697486   0.303937
S  -1.434605   1.718137  -0.547379
H  -1.470142  -1.601622   0.717679
H  -1.003342  -0.288546   1.645881
H  -2.041753   1.212174  -1.640197
28
Dimer 58 of the n...z  type
C  -2.723676  -0.395155   0.987968
C  -1.881898  -1.534216   0.382950
N  -4.097645  -0.442481   0.513317
H  -4.111806  -0.286427  -0.491496
H  -4.632130   0.316314   0.924979
H  -2.718551  -0.552185   2.072342
C  -2.019783   0.943732   0.737578
```



```
O  -2.482624   1.841214   0.068693
O  -0.818689   0.983296   1.322908
H  -0.277930   1.736252   0.965931
H  -0.898614  -1.563272   0.847897
H  -2.386168  -2.481158   0.575234
S  -1.682776  -1.464384  -1.452298
H  -0.927081  -0.342450  -1.516257
C   1.224445   1.476483  -0.823764
C   2.402674   0.505714  -0.532166
O   1.044986   2.379515   0.045633
O   0.533656   1.203524  -1.815511
H   2.941566   0.258772  -1.444019
C   1.821599  -0.753639   0.118157
N   3.362598   1.179499   0.410125
H   4.010585   1.792589  -0.085900
H   1.143909  -1.221798  -0.592885
H   1.252493  -0.485532   1.010339
S   3.076982  -1.975722   0.679274
H   2.806269   1.769707   1.044529
H   3.909951   0.490639   0.938962
H   3.566732  -2.266793  -0.543488
28
Dimer 59 of the n...z  type
C  -2.919807   0.314933  -0.118696
C  -2.851079  -1.166267  -0.513625
N  -2.504956   1.272689  -1.147565
H  -1.880979   0.843110  -1.823828
H  -3.297567   1.655476  -1.647522
H  -3.958544   0.526500   0.155881
C  -2.127471   0.577424   1.176766
O  -2.212118  -0.123820   2.158670
O  -1.371555   1.674540   1.136515
H  -1.499188   2.031072   0.218734
H  -3.154760  -1.785829   0.328976
H  -3.531564  -1.347104  -1.345895
S  -1.191316  -1.720703  -1.100851
H  -0.533881  -1.617909   0.085427
C   1.933234  -1.143914   0.648829
C   2.738696   0.192650   0.701310
O   2.390302  -2.031252  -0.110887
O   0.918762  -1.172518   1.374320
H   3.214397   0.252521   1.680976
C   1.862481   1.424684   0.492800
N   3.833871   0.098947  -0.328019
H   4.723110   0.494935  -0.025592
H   2.430541   2.342674   0.649382
H   1.041264   1.383534   1.204602
S   1.171838   1.563282  -1.211882
H   3.919555  -0.912119  -0.536102
H   3.536703   0.558951  -1.197009
H   0.431056   0.431559  -1.176940
28
Dimer 60 of the n...z  type
C  -2.722062  -0.393016   0.988477
C  -1.879757  -1.532744   0.385421
N  -4.096153  -0.441792   0.514356
H  -4.110613  -0.289788  -0.491064
H  -4.630357   0.318721   0.923196
H  -2.716543  -0.548156   2.073127
C  -2.018653   0.945663   0.735813
O  -2.480876   1.841108   0.063807
O  -0.818695   0.987452   1.323335
H  -0.277515   1.739496   0.965159
H  -0.895982  -1.559279   0.849449
H  -2.382705  -2.479727   0.581030
S  -1.683311  -1.468826  -1.450168
H  -0.934931  -0.342389  -1.520760
C   1.223504   1.476246  -0.823771
C   2.401563   0.505116  -0.532673
```

```
O   1.045795   2.380512   0.044690
O   0.531144   1.202187  -1.814069
H   2.939044   0.256908  -1.445012
C   1.820594  -0.752963   0.119909
N   3.363372   1.179280   0.407557
H   4.010659   1.791858  -0.090010
H   1.140572  -1.220616  -0.589265
H   1.253838  -0.483385   1.013158
S   3.075897  -1.976065   0.679081
H   2.808477   1.770070   1.042594
H   3.911434   0.490574   0.935822
H   3.562630  -2.268332  -0.544583
28
Dimer 61 of the n...z  type
C  -2.523393   0.813787  -0.764203
C  -3.473143   0.045559   0.166072
N  -1.892057   0.092291  -1.865021
H  -1.412299  -0.746853  -1.536398
H  -2.561881  -0.157226  -2.582340
H  -3.120320   1.622847  -1.205272
C  -1.432810   1.574513   0.009620
O  -1.573834   2.032089   1.118877
O  -0.320221   1.774129  -0.707545
H  -0.456842   1.255255  -1.539453
H  -3.767273   0.701492   0.985616
H  -4.368151  -0.232276  -0.391716
S  -2.811026  -1.536371   0.846599
H  -1.533473  -1.112495   1.075343
C   0.919158  -1.107296   0.148053
C   2.469465  -1.243080   0.281075
O   0.439375  -1.480414  -0.950349
O   0.329354  -0.667088   1.153812
H   2.663642  -2.181006   0.805899
C   3.135903  -0.097297   1.032289
N   3.019504  -1.395883  -1.110463
H   3.857856  -1.973797  -1.162915
H   4.202815  -0.282656   1.163501
H   2.666916  -0.021853   2.010653
S   3.011229   1.522095   0.159134
H   2.249642  -1.800192  -1.669448
H   3.229877  -0.469065  -1.501069
H   1.661399   1.584802   0.142583
28
Dimer 62 of the n...z  type
C  -2.917945  -0.034507   0.627465
C  -2.240407  -1.388704   0.381849
N  -3.912590   0.383111  -0.364691
H  -3.694579  -0.006270  -1.278758
H  -4.847025   0.089974  -0.107533
H  -3.410022  -0.091412   1.604565
C  -1.881071   1.091486   0.799769
O  -0.858048   0.967856   1.438206
O  -2.232980   2.237606   0.225929
H  -3.095021   2.028062  -0.222818
H  -1.499575  -1.579809   1.156590
H  -2.993106  -2.176429   0.421651
S  -1.454345  -1.550861  -1.277113
H  -0.546776  -0.544504  -1.161581
C   1.815900   1.151453  -0.814431
C   2.872194   0.003019  -0.681683
O   2.147695   2.244532  -0.297611
O   0.785781   0.861242  -1.454834
H   3.398793  -0.051869  -1.636425
C   2.286189  -1.364519  -0.354442
N   3.874255   0.441063   0.351876
H   4.830903   0.151808   0.151923
H   3.060098  -2.133366  -0.368791
H   1.541302  -1.607023  -1.108448
S   1.528726  -1.460381   1.322754
```



```
H   3.790125   1.473115   0.382308
H   3.599339   0.080265   1.273738
H   0.604906  -0.488205   1.149620
28
Dimer 63 of the n...z  type
C  -2.159150  -1.037239  -0.475822
C  -3.029502  -0.523196   0.684840
N  -1.073934  -1.863165   0.006832
H  -0.543060  -1.399480   0.737292
H  -0.419962  -2.119277  -0.724843
H  -2.816058  -1.647556  -1.108641
C  -1.731595   0.127982  -1.373416
O  -0.590945   0.381345  -1.704388
O  -2.775882   0.856288  -1.778889
H  -2.467103   1.574908  -2.354850
H  -3.935436  -0.052814   0.309610
H  -3.305205  -1.371365   1.310912
S  -2.162937   0.658615   1.810756
H  -2.182422   1.716341   0.973596
C   2.028325  -1.652306   0.418124
C   2.372746  -0.185458  -0.009809
O   1.997591  -2.489831  -0.517084
O   1.823690  -1.823460   1.635226
H   3.438805  -0.030870   0.166178
C   1.552821   0.854519   0.741403
N   2.155848  -0.074824  -1.493213
H   2.484361  -0.945697  -1.922966
H   1.742170   0.740213   1.805202
H   0.488312   0.698799   0.575043
S   1.862952   2.596957   0.224353
H   1.143077   0.005028  -1.687366
H   2.627500   0.734800  -1.897389
H   3.133884   2.673196   0.670311
28
Dimer 64 of the n...z  type
C  -2.950196   0.583949  -0.137479
C  -2.968142  -0.464157   0.983135
N  -3.137489   0.079738  -1.501217
H  -2.763813  -0.861831  -1.593486
H  -4.114944   0.060578  -1.764892
H  -3.751176   1.301181   0.073710
C  -1.665633   1.428282  -0.093194
O  -1.141038   1.809089   0.928423
O  -1.177608   1.731053  -1.298370
H  -1.782657   1.262678  -1.931584
H  -2.786143   0.016660   1.943254
H  -3.949414  -0.938061   1.009451
S  -1.754578  -1.836233   0.754947
H  -0.629573  -1.161728   1.128155
C   1.940836   0.266843   1.292666
C   1.742295   0.785421  -0.168062
O   2.908877   0.775007   1.906734
O   1.113079  -0.575053   1.699800
H   1.049148   1.625104  -0.111795
C   1.208233  -0.236906  -1.158914
N   3.068360   1.341206  -0.615849
H   2.987547   2.096031  -1.296836
H   1.136266   0.193900  -2.158074
H   0.212245  -0.537878  -0.853164
S   2.263190  -1.739745  -1.318187
H   3.536407   1.666012   0.246405
H   3.647086   0.595052  -1.016214
H   2.084272  -2.150026  -0.045243
28
Dimer 65 of the n...z  type
C  -2.186439   0.005620  -0.936027
C  -1.577472  -1.362126  -0.603976
N  -1.330265   1.180056  -0.808997
H  -0.762507   1.159939   0.037320
```

```
H  -0.686863   1.286995  -1.584691
H  -2.530111  -0.048656  -1.976362
C  -3.472978   0.254557  -0.127598
O  -4.233712  -0.619655   0.223184
O  -3.694067   1.544201   0.131435
H  -2.888330   2.004177  -0.222891
H  -2.319633  -2.145048  -0.754442
H  -0.734037  -1.553933  -1.264359
S  -0.897961  -1.510011   1.101064
H  -2.072112  -1.376864   1.751035
C   1.760237   1.563757   0.100740
C   2.899694   0.616223   0.606973
O   0.949083   1.914388   0.996978
O   1.776859   1.868261  -1.104836
H   3.700437   1.256133   0.984934
C   3.470410  -0.333045  -0.435133
N   2.345403  -0.121013   1.796633
H   3.056718  -0.421629   2.462797
H   4.256958  -0.957120  -0.008466
H   3.896144   0.263177  -1.238979
S   2.226010  -1.494258  -1.143842
H   1.674879   0.530339   2.236036
H   1.809108  -0.939689   1.489186
H   1.498589  -0.530291  -1.744090
28
Dimer 66 of the n...z  type
C  -2.860907  -0.554102  -0.563513
C  -3.344662  -0.286537   0.875244
N  -1.895253  -1.630494  -0.606559
H  -1.104960  -1.489768   0.018381
H  -1.520528  -1.755046  -1.540633
H  -3.749671  -0.847659  -1.132648
C  -2.385077   0.762925  -1.176877
O  -1.221695   1.073822  -1.351053
O  -3.402978   1.569941  -1.485128
H  -3.059170   2.409913  -1.831718
H  -4.178838   0.412938   0.873238
H  -3.679374  -1.231725   1.302326
S  -2.050459   0.341216   2.037246
H  -2.040218   1.616857   1.598486
C   1.686445  -1.777799  -0.224173
C   2.042981  -0.264997  -0.379967
O   0.829267  -2.044122   0.656315
O   2.323847  -2.546918  -0.966695
H   2.052220   0.004115  -1.435114
C   3.410537  -0.003788   0.249436
N   0.982442   0.553098   0.289843
H   0.158487   0.676096  -0.327117
H   4.143018  -0.635893  -0.248164
H   3.397416  -0.262356   1.310494
S   3.955142   1.757863   0.188080
H   0.653451   0.043176   1.119887
H   1.328309   1.480580   0.552506
H   3.991181   1.859266  -1.156530
28
Dimer 67 of the n...z  type
C  -2.624264  -0.514929   0.862606
C  -1.615825  -1.213683  -0.054985
N  -4.032763  -0.881719   0.681845
H  -4.229433  -1.085568  -0.295081
H  -4.280003  -1.696787   1.229730
H  -2.342867  -0.752324   1.894985
C  -2.498494   1.015935   0.772722
O  -1.444198   1.597414   0.647420
O  -3.662698   1.657959   0.872306
H  -4.338556   0.933154   0.922549
H  -0.597327  -0.934818   0.208342
H  -1.714501  -2.292773   0.066286
S  -1.897754  -0.890483  -1.849594
```



```
H  -1.432888   0.375807  -1.839735
C   2.634363  -1.080169   0.808630
C   2.856771   0.470145   0.836286
O   3.671951  -1.761489   0.612406
O   1.465052  -1.458344   1.006365
H   2.944342   0.763396   1.884635
C   1.746639   1.278080   0.181517
N   4.195566   0.732052   0.197802
H   4.688967   1.535739   0.584866
H   1.950864   2.348025   0.244080
H   0.809176   1.073364   0.690621
S   1.535177   0.915790  -1.613120
H   4.731745  -0.142942   0.332908
H   4.082530   0.866575  -0.813011
H   1.129848  -0.360011  -1.450088
28
Dimer 68 of the n...z  type
C   2.304607  -0.178075  -0.838517
C   1.295480   0.952434  -0.613971
N   1.849604  -1.521606  -0.472563
H   1.243607  -1.477145   0.347496
H   1.236198  -1.880015  -1.198031
H   2.549209  -0.182824  -1.907787
C   3.644900   0.099980  -0.143204
O   4.141027   1.200579  -0.042238
O   4.236128  -1.001780   0.320782
H   3.576902  -1.722746   0.130166
H   1.659836   1.887711  -1.036118
H   0.370704   0.666607  -1.112448
S   0.855129   1.232561   1.156426
H   2.007948   1.850553   1.485680
C  -1.485655  -1.366798  -0.190251
C  -2.962719  -0.953991   0.129545
O  -0.843142  -1.790631   0.804003
O  -1.123143  -1.259099  -1.376214
H  -3.570213  -1.854588   0.014223
C  -3.537203   0.148971  -0.747464
N  -3.001527  -0.593228   1.589808
H  -3.905044  -0.756553   2.033797
H  -4.587180   0.329546  -0.513528
H  -3.457873  -0.170002  -1.784146
S  -2.692135   1.774103  -0.542393
H  -2.259992  -1.164433   2.029403
H  -2.747512   0.394261   1.710146
H  -1.516081   1.375974  -1.061280
28
Dimer 69 of the n...z  type
C  -2.930210  -0.506307  -0.535045
C  -2.412051   0.897679  -0.897339
N  -4.112836  -0.439941   0.306905
H  -3.874772  -0.018709   1.200862
H  -4.445476  -1.376984   0.511740
H  -3.198311  -0.984897  -1.483084
C  -1.780571  -1.334307   0.054623
O  -1.748498  -1.737547   1.196196
O  -0.802247  -1.534079  -0.835783
H   0.020734  -1.979555  -0.427710
H  -1.592393   0.823710  -1.609873
H  -3.220917   1.464226  -1.358746
S  -1.884754   1.927066   0.543453
H  -1.026784   1.039216   1.081848
C   2.461265  -1.619340   0.216745
C   2.481744  -0.184756  -0.390680
O   1.473628  -2.362651  -0.064299
O   3.423848  -1.877766   0.948612
H   3.484547   0.018289  -0.763716
C   2.110986   0.836939   0.686528
N   1.560763  -0.062119  -1.578516
H   1.879778  -0.647563  -2.351720
```

```
H   2.844471   0.759758   1.486415
H   1.132305   0.617662   1.112571
S   2.012200   2.577686   0.090499
H   0.598995  -0.356320  -1.355456
H   1.531152   0.912883  -1.897983
H   3.331223   2.723563  -0.153909
28
Dimer 70 of the n...z  type
C  -2.904236  -0.437791  -0.746480
C  -1.475942   0.107140  -0.645476
N  -3.842979   0.322699  -1.573634
H  -3.664850   1.321449  -1.503008
H  -3.780302   0.059760  -2.549669
H  -2.832405  -1.449168  -1.162259
C  -3.521464  -0.641035   0.649873
O  -2.884024  -0.979693   1.621349
O  -4.837319  -0.432422   0.689943
H  -5.066923  -0.114926  -0.220196
H  -0.857159  -0.569270  -0.057591
H  -1.053707   0.192639  -1.647056
S  -1.379325   1.805717   0.077245
H  -1.783202   1.455393   1.316085
C   1.852596  -1.231576  -0.326223
C   2.019131   0.326064  -0.288186
O   1.177031  -1.710191   0.622545
O   2.378525  -1.808107  -1.292801
H   1.274212   0.744672  -0.966984
C   3.395288   0.820667  -0.709334
N   1.642037   0.774147   1.095711
H   1.033053   1.596261   1.092389
H   3.443406   1.910216  -0.688834
H   3.578939   0.479115  -1.725373
S   4.758314   0.236429   0.386910
H   1.132932  -0.043052   1.488419
H   2.469486   0.941266   1.674796
H   4.587770  -1.075402   0.121226
28
Dimer 71 of the n...z  type
C   2.840186   0.399966  -0.548965
C   2.478063   1.736883   0.110182
N   2.120055  -0.018509  -1.745622
H   1.105016  -0.010719  -1.620395
H   2.359700   0.557753  -2.543279
H   3.899336   0.491377  -0.827361
C   2.856244  -0.771094   0.448414
O   3.008838  -0.650701   1.643013
O   2.793942  -1.965521  -0.145103
H   2.558425  -1.765385  -1.082885
H   3.090167   1.864383   1.003094
H   2.705291   2.547094  -0.583754
S   0.704356   1.968600   0.556686
H   0.471944   0.708996   0.982844
C  -1.011525  -1.016429  -0.505858
C  -2.492116  -1.302784  -0.097333
O  -0.151197  -1.267923   0.375148
O  -0.843604  -0.569334  -1.655498
H  -2.764472  -2.276685  -0.508669
C  -3.478888  -0.252368  -0.589074
N  -2.512846  -1.437865   1.401624
H  -3.202988  -2.104164   1.747294
H  -4.498387  -0.496585  -0.287412
H  -3.429445  -0.226202  -1.675052
S  -3.141499   1.434480   0.079047
H  -1.551170  -1.722843   1.658501
H  -2.684019  -0.524415   1.837089
H  -1.923308   1.564820  -0.489849
28
Dimer 72 of the n...z  type
C   2.571009   0.828010  -0.229060
```



```
C   3.049665  -0.164209   0.842524
N   1.692380   1.905812   0.201624
H   0.970264   1.607084   0.858729
H   2.210653   2.672049   0.612101
H   3.471176   1.279171  -0.663696
C   1.921659   0.079091  -1.404262
O   2.332634  -0.976131  -1.833968
O   0.855729   0.683772  -1.931770
H   0.674019   1.482849  -1.376129
H   3.751071  -0.874555   0.406426
H   3.555592   0.385870   1.636421
S   1.696494  -1.094596   1.680321
H   1.283731  -1.787170   0.598129
C  -1.670286   1.289621   0.501907
C  -2.728391   0.160581   0.701524
O  -1.593638   1.755833  -0.664451
O  -0.989568   1.556748   1.508110
H  -3.302892   0.355142   1.605381
C  -2.039954  -1.200893   0.826505
N  -3.663536   0.214064  -0.481803
H  -4.533018   0.700118  -0.263744
H  -2.745522  -1.966457   1.151785
H  -1.257944  -1.099800   1.578274
S  -1.323515  -1.846774  -0.746854
H  -3.137741   0.774815  -1.183042
H  -3.884964  -0.703465  -0.872808
H  -0.687461  -0.717829  -1.120228
28
Dimer 73 of the n...z  type
C  -2.598997   0.452281   0.825003
C  -3.385784   0.048396  -0.438606
N  -1.867848   1.686216   0.611597
H  -1.197229   1.594117  -0.144190
H  -1.349952   1.955743   1.441227
H  -3.340782   0.612020   1.612771
C  -1.733894  -0.728192   1.262879
O  -0.554426  -0.858369   0.992362
O  -2.425864  -1.636900   1.950438
H  -1.852753  -2.393161   2.160745
H  -4.012639  -0.819833  -0.240736
H  -4.024698   0.885595  -0.718628
S  -2.348969  -0.291977  -1.931027
H  -1.979116  -1.543700  -1.591966
C   2.960909  -1.344108  -0.110911
C   2.353959   0.036987  -0.538703
O   2.447927  -2.348154  -0.662520
O   3.904832  -1.282308   0.700425
H   3.022321   0.465140  -1.288198
C   2.179693   0.997638   0.629345
N   1.040997  -0.219124  -1.222040
H   1.139293  -1.081418  -1.769354
H   3.147046   1.143732   1.102227
H   1.494294   0.579059   1.367865
S   1.450203   2.628627   0.163133
H   0.309125  -0.397859  -0.512610
H   0.736827   0.553254  -1.815044
H   2.441397   3.019110  -0.664367
28
Dimer 74 of the n...z  type
C  -2.923768   0.115952   0.121071
C  -2.200930   0.589616  -1.159924
N  -3.559071   1.212681   0.837249
H  -2.858184   1.804407   1.273518
H  -4.133332   0.845310   1.589826
H  -3.697826  -0.578340  -0.214792
C  -1.894983  -0.682616   0.924336
O  -1.146585  -0.162171   1.732909
O  -1.844308  -1.958657   0.563012
H  -0.912842  -2.315953   0.692385
```

```
H  -1.704769  -0.255838  -1.635791
H  -2.933069   1.021584  -1.841396
S  -0.930259   1.929564  -0.907827
H  -0.611835   1.636353   0.369505
C   0.897080  -1.720528  -0.609151
C   2.073252  -0.707872  -0.503003
O   0.233454  -1.688997  -1.661392
O   0.722699  -2.450366   0.404705
H   3.008893  -1.237816  -0.690373
C   2.121125  -0.024995   0.861108
N   1.918820   0.299152  -1.610344
H   1.846029  -0.180867  -2.509053
H   2.274503  -0.792967   1.614640
H   1.171681   0.459840   1.084980
S   3.409369   1.283814   1.017777
H   1.036653   0.836206  -1.491394
H   2.700108   0.959523  -1.626752
H   4.477257   0.465871   0.916653
28
Dimer 75 of the n...z  type
C  -2.496352   0.438707  -0.671660
C  -1.747839  -0.894599  -0.775984
N  -1.796533   1.531075   0.007422
H  -1.275663   1.174056   0.806921
H  -1.087256   1.920393  -0.606477
H  -2.711350   0.766150  -1.696221
C  -3.880650   0.262133  -0.028731
O  -4.573020  -0.721328  -0.170118
O  -4.270852   1.318565   0.686532
H  -3.488570   1.930147   0.661131
H  -2.330003  -1.614859  -1.348789
H  -0.800792  -0.721057  -1.284700
S  -1.323251  -1.640971   0.857916
H  -2.570040  -2.059513   1.154960
C   1.320135   0.670750  -0.206410
C   2.121030  -0.672113  -0.266860
O   0.933772   1.105636  -1.322330
O   1.116690   1.130573   0.930195
H   1.404665  -1.481450  -0.110718
C   3.233192  -0.788547   0.765087
N   2.637666  -0.818190  -1.672975
H   2.722944  -1.786645  -1.981475
H   3.743211  -1.749620   0.688595
H   2.785910  -0.704720   1.752663
S   4.543002   0.495973   0.575157
H   1.971011  -0.295660  -2.265678
H   3.550701  -0.358016  -1.761376
H   3.734720   1.543430   0.839374
28
Dimer 76 of the n...z  type
C   1.697373   0.327328  -0.036228
C   2.147690  -0.681327   1.025761
N   1.111155  -0.220534  -1.257662
H   1.571591  -1.090396  -1.513964
H   0.109949  -0.405211  -1.180475
H   0.956687   0.975658   0.442918
C   2.840417   1.284036  -0.418188
O   3.669753   1.693071   0.364436
O   2.814890   1.650387  -1.699069
H   2.066120   1.116246  -2.080911
H   2.520893  -0.159856   1.905856
H   1.300163  -1.299106   1.320684
S   3.437030  -1.861388   0.434872
H   4.429745  -0.953490   0.336350
C  -2.874158  -0.776010  -0.964277
C  -3.132739   0.660966  -0.397863
O  -3.872077  -1.531063  -0.971933
O  -1.711764  -0.998937  -1.363126
H  -3.382370   1.299914  -1.247604
```



```
C  -1.959120   1.267479   0.356833
N  -4.366146   0.582185   0.460882
H  -4.895247   1.453526   0.500395
H  -2.208595   2.255256   0.746645
H  -1.132132   1.377971  -0.340999
S  -1.430607   0.273753   1.818188
H  -4.937340  -0.180268   0.061802
H  -4.113649   0.306801   1.416364
H  -1.132172  -0.839798   1.117392
28
Dimer 77 of the n...z  type
C  -2.792729   0.708930  -0.409772
C  -3.322147  -0.727764  -0.501394
N  -1.838999   1.127466  -1.429176
H  -1.162078   0.404184  -1.664032
H  -2.302592   1.422295  -2.279212
H  -3.665835   1.368449  -0.478958
C  -2.208361   1.000996   0.982744
O  -2.662991   0.542490   2.007364
O  -1.156127   1.819310   0.976971
H  -0.959511   2.009397   0.025384
H  -4.145207  -0.876349   0.195883
H  -3.683441  -0.910524  -1.513097
S  -2.073364  -2.049589  -0.171330
H  -2.036815  -1.876443   1.165960
C   1.301963   0.710855  -0.799566
C   1.482678  -0.078154   0.530779
O   0.991690   0.025412  -1.804201
O   1.474099   1.941115  -0.701409
H   0.714195   0.253924   1.230265
C   2.856727   0.132601   1.156918
N   1.227326  -1.534980   0.261774
H   1.316665  -2.107922   1.102443
H   2.955594  -0.422069   2.091597
H   2.968226   1.193735   1.369241
S   4.258058  -0.430321   0.095230
H   0.275928  -1.652731  -0.122624
H   1.886389  -1.874463  -0.444391
H   4.082919   0.488262  -0.877116
28
Dimer 78 of the n...z  type
C  -2.052628  -0.461315  -0.560204
C  -3.330886   0.214986  -1.063850
N  -2.209861  -1.797788   0.021926
H  -3.118895  -1.900859   0.465491
H  -2.111291  -2.523600  -0.677014
H  -1.353277  -0.537826  -1.396141
C  -1.308871   0.431462   0.443608
O  -1.212772   1.634426   0.334347
O  -0.756020  -0.239560   1.454132
H  -1.023768  -1.181911   1.287432
H  -3.092757   1.180211  -1.508330
H  -3.800049  -0.414391  -1.820299
S  -4.616059   0.458410   0.240884
H  -3.930510   1.378806   0.949491
C   1.829453  -1.169835  -0.538363
C   2.280592  -0.398217   0.747059
O   2.402509  -2.273707  -0.716880
O   0.944585  -0.619757  -1.220051
H   1.617792  -0.711967   1.553881
C   2.234524   1.117231   0.636165
N   3.659455  -0.894890   1.094760
H   3.875338  -0.847749   2.090430
H   2.565807   1.587433   1.563309
H   1.207966   1.415135   0.443399
S   3.326095   1.791292  -0.690098
H   3.694675  -1.869616   0.753896
H   4.366305  -0.361708   0.576774
H   2.651951   1.215708  -1.707393
```

```
28
Dimer 79 of the n...z  type
C  -3.679766  -0.883103   0.107981
C  -2.183138  -0.917091   0.434867
N  -4.058827  -1.200025  -1.269753
H  -3.375177  -0.840521  -1.931121
H  -4.142898  -2.198790  -1.415080
H  -4.169236  -1.609896   0.766184
C  -4.304594   0.469141   0.499645
O  -3.947165   1.119744   1.455522
O  -5.300557   0.852014  -0.298742
H  -5.328502   0.165357  -1.011970
H  -2.024627  -0.727307   1.495189
H  -1.777267  -1.896852   0.184180
S  -1.185797   0.293823  -0.541552
H  -1.655890   1.400734   0.069361
C   3.636666  -1.311381   0.079947
C   2.375383  -0.432499  -0.214216
O   3.595850  -1.951283   1.159357
O   4.520134  -1.294560  -0.798790
H   1.692510  -1.038450  -0.815326
C   2.657892   0.868074  -0.952984
N   1.671233  -0.172971   1.085619
H   0.673486   0.029965   0.926037
H   1.735223   1.422343  -1.131958
H   3.115444   0.626074  -1.909039
S   3.764693   2.012654  -0.021424
H   1.805301  -1.005150   1.674442
H   2.105334   0.620626   1.568312
H   4.836366   1.193038  -0.042870
28
Dimer 80 of the n...z  type
C   2.977636  -0.912979   0.266443
C   3.369109   0.519244   0.651954
N   3.141891  -1.266085  -1.147230
H   3.016414  -0.451117  -1.742893
H   4.054541  -1.662244  -1.335149
H   3.585118  -1.598351   0.866732
C   1.523639  -1.201558   0.677409
O   1.026217  -0.842598   1.716399
O   0.839557  -1.894086  -0.247132
H   1.502588  -2.027878  -0.980670
H   3.233218   0.670524   1.721636
H   4.417751   0.682546   0.403776
S   2.424236   1.816192  -0.261774
H   1.252380   1.683257   0.399185
C  -2.027684   1.543474   0.246398
C  -2.262962   0.650832  -1.018792
O  -0.857371   1.981212   0.379750
O  -3.030667   1.746707   0.954218
H  -2.601277   1.318442  -1.815020
C  -3.292923  -0.454815  -0.831622
N  -0.933272   0.103224  -1.460372
H  -0.910773  -0.148873  -2.448875
H  -3.406839  -1.045249  -1.742272
H  -4.246869   0.012308  -0.598457
S  -2.866648  -1.655632   0.500812
H  -0.225267   0.825274  -1.269359
H  -0.664813  -0.726425  -0.910747
H  -2.930680  -0.756201   1.504105
28
Dimer 81 of the n...z  type
C   2.596276  -0.554154   0.663166
C   1.705107   0.614742   1.095057
N   4.043330  -0.339051   0.754108
H   4.278375   0.625418   0.531992
H   4.392573  -0.542357   1.682757
H   2.342356  -1.408512   1.301211
C   2.261575  -1.029347  -0.762003
```



```
O   1.143050  -1.024248  -1.227377
O   3.316800  -1.473989  -1.441139
H   4.087044  -1.302191  -0.839715
H   0.654691   0.330141   1.109680
H   1.982609   0.915797   2.105826
S   1.910586   2.133695   0.067872
H   1.432086   1.606914  -1.077702
C  -2.158416  -0.502374   1.387443
C  -1.743899  -0.824903  -0.083823
O  -2.995260  -1.292144   1.893012
O  -1.590212   0.481945   1.897941
H  -0.806713  -1.380719  -0.048981
C  -1.541275   0.398845  -0.963057
N  -2.785118  -1.753413  -0.648759
H  -2.419826  -2.409053  -1.338819
H  -1.208632   0.113030  -1.960653
H  -0.775467   1.019343  -0.510173
S  -3.070103   1.406225  -1.186353
H  -3.180623  -2.248078   0.168338
H  -3.545859  -1.212481  -1.075068
H  -3.184888   1.756414   0.111710
28
Dimer 82 of the n...z  type
C   3.094642  -0.414453   0.687453
C   2.409218   0.910871   1.043042
N   4.245108  -0.331105  -0.215403
H   4.143778   0.448189  -0.861152
H   5.113492  -0.212412   0.291658
H   3.428000  -0.871456   1.625612
C   2.080029  -1.419256   0.112173
O   0.951292  -1.542599   0.533507
O   2.562322  -2.165797  -0.878629
H   3.482473  -1.820338  -1.019360
H   1.577544   0.727712   1.722293
H   3.125312   1.564210   1.541846
S   1.815220   1.865853  -0.417977
H   0.892041   0.979791  -0.849891
C  -1.699083   1.138435  -0.656884
C  -1.660386   0.215368   0.602707
O  -2.177107   2.284063  -0.472170
O  -1.218777   0.639008  -1.693879
H  -0.638425   0.220130   0.982175
C  -2.064058  -1.225264   0.326061
N  -2.533125   0.853861   1.649799
H  -2.200010   0.717847   2.603524
H  -1.996060  -1.831413   1.230098
H  -1.375257  -1.627598  -0.409814
S  -3.800828  -1.399786  -0.272681
H  -2.562004   1.857707   1.400293
H  -3.492175   0.497006   1.576536
H  -3.616865  -0.717335  -1.421993
28
Dimer 83 of the n...z  type
C   3.066077  -0.479212  -0.795801
C   1.704201   0.216234  -0.730847
N   3.168969  -1.779587  -0.128489
H   2.610797  -1.798521   0.721539
H   2.852105  -2.532177  -0.727712
H   3.301565  -0.627736  -1.855824
C   4.190663   0.436370  -0.279471
O   4.202409   1.636678  -0.434870
O   5.176200  -0.217135   0.336837
H   4.873086  -1.159960   0.360907
H   1.724636   1.146172  -1.296835
H   0.927173  -0.432286  -1.132577
S   1.149279   0.586985   0.990591
H   1.980532   1.626689   1.206468
C  -1.925941  -1.028909   0.216203
C  -2.472319   0.307621   0.806806
```

```
O  -1.290403  -0.934953  -0.860470
O  -2.222686  -2.042070   0.879548
H  -2.131970   0.404469   1.838358
C  -3.997858   0.353433   0.777938
N  -1.879098   1.460673   0.045144
H  -2.269578   2.362098   0.324374
H  -4.373397   1.275534   1.225038
H  -4.367039  -0.488765   1.360133
S  -4.729633   0.302469  -0.915372
H  -0.857506   1.469244   0.209819
H  -2.028746   1.318300  -0.957774
H  -4.298199  -0.935333  -1.233349
28
Dimer 84 of the n...z  type
C   2.625353   0.611240   0.616434
C   2.077079  -0.614357   1.356446
N   1.656405   1.448585  -0.086817
H   0.900071   0.912991  -0.508606
H   1.233322   2.137852   0.522141
H   3.133422   1.235214   1.361137
C   3.739505   0.211712  -0.369219
O   4.533488  -0.679095  -0.163135
O   3.767892   0.960386  -1.471128
H   2.979013   1.557291  -1.377887
H   2.878709  -1.118742   1.893970
H   1.325427  -0.292442   2.078101
S   1.233862  -1.837771   0.262117
H   2.345288  -2.247417  -0.381874
C  -1.788714   1.379062   0.064161
C  -1.943535  -0.052694   0.677966
O  -2.276968   2.308974   0.749626
O  -1.173137   1.428469  -1.018295
H  -1.016262  -0.273993   1.208386
C  -2.214437  -1.143860  -0.348003
N  -3.026570   0.020984   1.719504
H  -2.898089  -0.629170   2.494868
H  -2.284987  -2.123259   0.126800
H  -1.386937  -1.155695  -1.051445
S  -3.797118  -0.904235  -1.263282
H  -3.025906   1.000962   2.048166
H  -3.940340  -0.154170   1.285889
H  -3.421198   0.239200  -1.873068
28
Dimer 85 of the n...z  type
C   2.970638  -0.735102  -0.361732
C   1.614948  -0.025661  -0.430174
N   3.197007  -1.601625   0.796784
H   2.764967  -1.213326   1.631621
H   2.818270  -2.529305   0.649262
H   3.058406  -1.350978  -1.264032
C   4.131894   0.270098  -0.468038
O   4.090647   1.272195  -1.145501
O   5.209012  -0.076476   0.237017
H   4.936637  -0.896131   0.722037
H   1.541526   0.551098  -1.351343
H   0.813424  -0.764109  -0.417176
S   1.297216   1.101805   0.998853
H   2.136308   2.081339   0.606545
C  -2.121855  -1.276406  -0.199844
C  -2.177795   0.150359   0.445095
O  -1.382836  -1.362658  -1.215527
O  -2.784789  -2.156731   0.373922
H  -1.460074   0.154873   1.267629
C  -3.545678   0.545712   0.982127
N  -1.664676   1.118733  -0.584688
H  -1.041519   1.828392  -0.193285
H  -3.517022   1.534147   1.442616
H  -3.836238  -0.184475   1.733784
S  -4.849947   0.634308  -0.319120
```



```
H  -1.151789   0.516416  -1.257808
H  -2.437862   1.563672  -1.088419
H  -4.796845  -0.673821  -0.645723
28
Dimer 86 of the n...z  type
C  -1.494804  -0.460968  -0.182754
C  -1.446711   0.725425   0.784131
N  -1.175280  -0.181245  -1.585598
H  -1.497978   0.746780  -1.849760
H  -0.176544  -0.220251  -1.753286
H  -0.770347  -1.189533   0.195345
C  -2.851978  -1.178943  -0.120029
O  -3.498096  -1.314893   0.895521
O  -3.250810  -1.674927  -1.293278
H  -2.557342  -1.366593  -1.933215
H  -1.693084   0.390692   1.789721
H  -0.441276   1.141669   0.808578
S  -2.565226   2.117673   0.318599
H  -3.723197   1.464798   0.548001
C   1.844405  -0.155551   1.430100
C   3.052527  -0.611167   0.543393
O   1.022720  -1.065066   1.706792
O   1.840507   1.042551   1.765927
H   3.871480  -0.862113   1.220903
C   3.536293   0.427771  -0.457251
N   2.642295  -1.895609  -0.126548
H   3.415461  -2.538539  -0.296504
H   4.350642   0.036948  -1.068862
H   3.898691   1.289997   0.097290
S   2.234801   0.987311  -1.639099
H   1.938955  -2.312794   0.508540
H   2.172220  -1.705682  -1.016745
H   1.483014   1.611113  -0.709449
28
Dimer 87 of the n...z  type
C   1.991216  -0.545396   0.502252
C   0.940642   0.506874   0.148648
N   2.619045  -0.426650   1.821637
H   2.727638   0.550449   2.082863
H   2.066728  -0.883587   2.537191
H   1.472245  -1.508984   0.448724
C   3.089807  -0.621193  -0.567102
O   2.890794  -0.460965  -1.750979
O   4.298171  -0.910902  -0.078375
H   4.166263  -0.932163   0.903832
H   0.504552   0.274398  -0.820065
H   0.150768   0.479114   0.896052
S   1.553942   2.244611   0.140887
H   2.324880   2.123767  -0.959118
C  -1.618431  -1.373502  -0.585805
C  -3.031839  -0.998088  -0.023892
O  -0.873801  -1.983497   0.223091
O  -1.397647  -1.037135  -1.762555
H  -3.719928  -1.785803  -0.338187
C  -3.565488   0.347949  -0.494356
N  -2.941314  -1.075877   1.477148
H  -3.800330  -1.388327   1.929038
H  -4.553255   0.545884  -0.075945
H  -3.643546   0.321121  -1.578524
S  -2.510449   1.779805  -0.001205
H  -2.159115  -1.730917   1.659220
H  -2.675475  -0.165438   1.866425
H  -1.477011   1.467669  -0.805841
28
Dimer 88 of the n...z  type
C   2.846561  -0.077427  -0.853510
C   2.884425   1.362373  -0.325684
N   1.876665  -0.410408  -1.887634
H   0.923533  -0.222311  -1.579240
```

```
H   2.067841   0.077941  -2.753885
H   3.845134  -0.263476  -1.271581
C   2.735347  -1.105751   0.283032
O   3.091493  -0.893614   1.420484
O   2.261958  -2.291667  -0.101128
H   1.937165  -2.162518  -1.021413
H   3.710894   1.473917   0.375412
H   3.042416   2.044963  -1.161169
S   1.352286   1.965714   0.498896
H   1.275217   0.998049   1.435743
C  -1.042387  -0.510892   0.622798
C  -1.931744   0.763568   0.522110
O  -0.578090  -0.937194  -0.463172
O  -0.929024  -0.978397   1.770458
H  -1.633741   1.472819   1.294430
C  -3.411838   0.430314   0.697527
N  -1.672628   1.424687  -0.803464
H  -2.261245   2.244162  -0.962126
H  -4.022890   1.334264   0.671969
H  -3.532303  -0.038297   1.672998
S  -4.116696  -0.663336  -0.610894
H  -0.681807   1.708252  -0.828910
H  -1.822202   0.744606  -1.555184
H  -3.317122  -1.724275  -0.377830
28
Dimer 89 of the n...z  type
C  -2.271249  -0.799866   0.212814
C  -3.747742  -0.385273   0.068819
N  -1.776609  -0.548081   1.550938
H  -1.739274   0.449043   1.739689
H  -0.844119  -0.932246   1.662458
H  -2.228051  -1.877779   0.021348
C  -1.439796  -0.153022  -0.897675
O  -0.478256   0.569274  -0.719358
O  -1.892878  -0.462569  -2.114372
H  -1.339371  -0.032922  -2.787332
H  -4.141366  -0.690318  -0.898812
H  -4.319030  -0.878029   0.854752
S  -4.054180   1.420679   0.300545
H  -3.560961   1.822303  -0.889106
C   3.696556   1.064009  -0.138190
C   2.725122   0.036851   0.545521
O   3.414890   2.275328   0.053239
O   4.641484   0.563099  -0.772927
H   3.271654  -0.435206   1.361932
C   2.235248  -1.017039  -0.437491
N   1.595975   0.826330   1.155014
H   1.411125   0.575570   2.123815
H   3.104734  -1.509614  -0.865375
H   1.668004  -0.556144  -1.246876
S   1.098352  -2.277361   0.285705
H   1.927793   1.809622   1.091712
H   0.719781   0.744542   0.609798
H   1.956038  -2.764673   1.204889
28
Dimer 90 of the n...z  type
C  -2.083033  -0.778681   0.406154
C  -3.158928  -0.894048  -0.677570
N  -2.557478  -0.683013   1.789232
H  -3.440316  -0.180598   1.839089
H  -2.690186  -1.598119   2.201834
H  -1.426115  -1.649771   0.329293
C  -1.132782   0.391497   0.126746
O  -0.771760   0.709626  -0.988329
O  -0.720526   1.032087   1.216091
H  -1.189269   0.572401   1.958959
H  -2.694599  -1.008288  -1.655957
H  -3.779622  -1.767861  -0.478465
S  -4.324757   0.537528  -0.732884
```



```
H  -3.421680   1.440946  -1.165856
C   1.843222  -1.438109  -0.138775
C   3.034792  -0.617016   0.453429
O   2.006899  -1.867797  -1.309107
O   0.884494  -1.601281   0.636699
H   3.465760  -1.219420   1.254143
C   2.630940   0.746717   1.019921
N   4.076737  -0.489077  -0.625173
H   5.034235  -0.606991  -0.297111
H   3.430874   1.160583   1.634846
H   1.741763   0.608297   1.631895
S   2.288611   2.029278  -0.259525
H   3.819915  -1.207531  -1.326488
H   3.983507   0.427490  -1.083608
H   1.111135   1.521370  -0.694695
28
Dimer 91 of the n...z  type
C   2.971120   0.265614  -0.186071
C   2.571287  -0.927903  -1.082678
N   3.585700  -0.190553   1.049163
H   2.891020  -0.655120   1.627929
H   3.933299   0.598905   1.584336
H   3.699997   0.849752  -0.754625
C   1.743661   1.145505   0.023123
O   1.047316   1.098260   1.022700
O   1.474569   1.923995  -1.021608
H   0.642774   2.425514  -0.857212
H   2.155099  -0.578102  -2.026802
H   3.472616  -1.503954  -1.290831
S   1.395193  -2.127930  -0.312981
H   0.245057  -1.505355  -0.622027
C  -2.553300  -1.494473  -0.099347
C  -2.310408  -0.055804   0.486830
O  -1.887555  -2.412052   0.447558
O  -3.360288  -1.553338  -1.041174
H  -3.253921   0.328839   0.868774
C  -1.763369   0.857998  -0.599445
N  -1.357771  -0.207643   1.647386
H  -1.759765   0.099806   2.531116
H  -2.456595   0.827710  -1.435820
H  -0.799750   0.494161  -0.957482
S  -1.548433   2.627810  -0.079960
H  -1.184018  -1.235299   1.677384
H  -0.458200   0.279168   1.511240
H  -2.417573   3.162722  -0.959039
28
Dimer 92 of the n...z  type
C   3.092931  -0.072787   0.885543
C   2.240383   1.199443   0.961086
N   4.315302   0.003289   0.083049
H   4.193580   0.637191  -0.703027
H   5.104422   0.324852   0.630170
H   3.374595  -0.336033   1.911119
C   2.250767  -1.268258   0.403326
O   1.094470  -1.440325   0.718581
O   2.917573  -2.118325  -0.374491
H   3.809503  -1.699480  -0.489270
H   1.372604   1.024852   1.595849
H   2.830433   2.005937   1.396546
S   1.695732   1.832506  -0.683847
H   0.965345   0.758193  -1.064442
C  -1.492263  -0.654178  -0.986642
C  -1.621017   0.202289   0.318447
O  -0.919349  -0.058580  -1.937472
O  -1.933874  -1.814285  -0.935988
H  -0.671745   0.087134   0.843165
C  -2.762271  -0.189955   1.243567
N  -1.723024   1.646266  -0.100282
H  -1.344625   2.299596   0.585443
```

```
H  -2.767695   0.423368   2.145950
H  -2.625508  -1.230782   1.527131
S  -4.430687   0.016878   0.481779
H  -1.202376   1.728067  -0.985453
H  -2.703878   1.886535  -0.284421
H  -4.232137  -0.897595  -0.490190
28
Dimer 93 of the n...z  type
C   2.261813   0.753172  -0.455730
C   1.570997  -0.431670  -1.139402
N   3.710676   0.854197  -0.668088
H   4.130609  -0.061573  -0.802218
H   3.934929   1.426061  -1.472969
H   1.791850   1.666569  -0.837036
C   1.984670   0.775866   1.057962
O   0.891380   0.574968   1.542800
O   3.048370   1.072669   1.798766
H   3.780741   1.185027   1.135941
H   0.500316  -0.416411  -0.961774
H   1.758437  -0.377520  -2.211752
S   2.166170  -2.080961  -0.557913
H   1.196820  -2.232528   0.367777
C  -2.123151  -1.181661   0.538913
C  -3.010796  -0.379726  -0.474547
O  -2.506920  -1.137870   1.735232
O  -1.161916  -1.788446   0.035139
H  -3.587709  -1.111159  -1.042624
C  -2.211525   0.486713  -1.441759
N  -3.986483   0.430744   0.335655
H  -4.931720   0.447255  -0.044300
H  -2.859434   0.933005  -2.197175
H  -1.486924  -0.153348  -1.940891
S  -1.357583   1.907397  -0.632096
H  -3.961100  -0.011216   1.275663
H  -3.646060   1.394115   0.438230
H  -0.626847   1.187425   0.252194
28
Dimer 94 of the n...z  type
C   2.783555   0.346324   0.234341
C   2.799536  -1.165533   0.475754
N   2.264120   1.185788   1.315222
H   1.465582   0.742083   1.763527
H   2.967386   1.361701   2.022672
H   3.819621   0.652637   0.046147
C   2.047036   0.708322  -1.067402
O   1.995772  -0.006430  -2.040239
O   1.495101   1.926251  -1.040571
H   1.649946   2.246056  -0.115945
H   3.230236  -1.674295  -0.385573
H   3.409677  -1.382844   1.352515
S   1.148058  -1.902463   0.829253
H   0.586647  -1.683726  -0.381238
C  -1.952009  -0.516471  -1.253343
C  -2.799270  -0.390805   0.060109
O  -1.367838  -1.621605  -1.395977
O  -1.975663   0.469085  -2.009846
H  -3.811538  -0.724425  -0.180129
C  -2.860461   1.009543   0.650557
N  -2.246586  -1.382188   1.048572
H  -2.918266  -1.666804   1.761951
H  -3.475432   1.031335   1.551421
H  -3.302176   1.669982  -0.091913
S  -1.209459   1.667171   1.139807
H  -1.934135  -2.193682   0.496243
H  -1.415196  -0.991332   1.510877
H  -0.701959   1.666091  -0.109089
28
Dimer 95 of the n...z  type
C   3.379988   0.576547   0.396103
```



```
C   2.115847   1.171067   1.030839
N   3.872378   1.239539  -0.814356
H   3.107211   1.656712  -1.338434
H   4.543358   1.966711  -0.599463
H   4.170785   0.612967   1.153209
C   3.195627  -0.925836   0.109503
O   2.646664  -1.687431   0.872764
O   3.727651  -1.322947  -1.046527
H   4.086161  -0.489915  -1.449565
H   1.822624   0.580089   1.897371
H   2.318514   2.192604   1.352641
S   0.684824   1.300579  -0.127875
H   0.448838  -0.026927  -0.266560
C  -1.999526  -1.494616  -0.414097
C  -2.782335  -0.819052   0.762792
O  -0.794836  -1.760030  -0.170390
O  -2.663089  -1.713613  -1.442951
H  -3.271889  -1.620210   1.321976
C  -3.834002   0.190282   0.324293
N  -1.776136  -0.205098   1.700420
H  -2.151414  -0.023098   2.631877
H  -4.345478   0.626614   1.183883
H  -4.565026  -0.334792  -0.286094
S  -3.155677   1.612426  -0.632490
H  -0.978972  -0.850910   1.756270
H  -1.404788   0.673519   1.310679
H  -2.704683   0.869275  -1.664144
28
Dimer 96 of the n...z  type
C   1.930793   0.255998  -0.697018
C   3.416982   0.304723  -1.086701
N   1.405707   1.488148  -0.090405
H   2.082559   1.877172   0.565845
H   1.289732   2.193452  -0.813627
H   1.346488   0.057763  -1.596761
C   1.669439  -0.965375   0.189144
O   2.005403  -2.078408  -0.133702
O   1.066711  -0.748352   1.370423
H   0.911009   0.204467   1.471044
H   3.701812  -0.619956  -1.586177
H   3.581130   1.137566  -1.770943
S   4.557062   0.603557   0.334481
H   4.405460  -0.606364   0.911503
C  -1.621945  -0.938945  -0.257033
C  -2.025050   0.148743   0.786041
O  -0.929402  -0.541497  -1.222543
O  -2.053118  -2.081597  -0.004978
H  -1.541853  -0.088783   1.735011
C  -3.534004   0.208845   1.004688
N  -1.491533   1.482735   0.341618
H  -1.723281   2.228436   0.999865
H  -3.798984   0.978357   1.731663
H  -3.857322  -0.757223   1.385610
S  -4.492768   0.612858  -0.520419
H  -0.450907   1.453272   0.219393
H  -1.892027   1.717661  -0.570712
H  -4.198428  -0.527369  -1.178435
28
Dimer 97 of the n...z  type
C   2.418952   0.053346   1.145738
C   3.296039  -0.515755   0.018814
N   1.469126  -0.932775   1.621665
H   0.937262  -1.360481   0.865881
H   0.804453  -0.517708   2.265530
H   3.098679   0.318317   1.964698
C   1.790234   1.374273   0.690243
O   0.600822   1.589697   0.618476
O   2.725383   2.279016   0.362472
H   2.291948   3.097113   0.069677
```

```
H   4.155319   0.125367  -0.164277
H   3.647607  -1.503420   0.315473
S   2.408269  -0.757804  -1.588397
H   2.489684   0.523615  -2.002363
C  -1.649683  -1.918427   0.286386
C  -1.756921  -0.376439   0.047325
O  -0.603068  -2.446945  -0.166982
O  -2.574505  -2.435318   0.939945
H  -1.272737   0.110180   0.894436
C  -3.181229   0.132675  -0.108153
N  -0.922372  -0.002615  -1.146607
H   0.006583  -0.450980  -1.093590
H  -3.753271  -0.184144   0.759866
H  -3.654602  -0.293451  -0.995239
S  -3.303419   1.965204  -0.311920
H  -1.356899  -0.318720  -2.015400
H  -0.791463   1.010506  -1.196878
H  -2.800854   2.283491   0.898450
28
Dimer 98 of the n...z  type
C   2.633944   0.555961   0.776957
C   1.155404   0.642711   0.385684
N   3.499364   1.632200   0.280793
H   3.202528   1.954910  -0.636216
H   3.507220   2.427722   0.907332
H   2.681164   0.566938   1.871695
C   3.247825  -0.800038   0.382930
O   2.673987  -1.857638   0.505707
O   4.500513  -0.713284  -0.069810
H   4.697685   0.258305  -0.064431
H   0.602696  -0.209285   0.777519
H   0.730409   1.559892   0.792780
S   0.873188   0.673899  -1.442519
H   0.466421  -0.607801  -1.542783
C  -2.002082  -1.300329  -0.647004
C  -3.076091  -0.815349   0.383617
O  -0.998688  -1.869443  -0.142946
O  -2.267411  -1.084204  -1.840713
H  -3.898109  -1.532776   0.352710
C  -3.617974   0.581119   0.101730
N  -2.457411  -0.909371   1.752445
H  -3.110641  -1.184615   2.484994
H  -4.431044   0.833144   0.784067
H  -3.995325   0.593225  -0.918224
S  -2.361418   1.915473   0.316610
H  -1.689307  -1.596577   1.651438
H  -2.032734  -0.008292   2.004271
H  -1.498383   1.473335  -0.629602
28
Dimer 99 of the n...z  type
C   3.336962  -0.781115   0.441241
C   3.598938   0.680663   0.826512
N   3.779446  -1.193525  -0.893104
H   3.756382  -0.409409  -1.540507
H   4.720407  -1.567106  -0.879449
H   3.846055  -1.407588   1.181654
C   1.846379  -1.134128   0.608283
O   1.172311  -0.757315   1.540513
O   1.369319  -1.923468  -0.352873
H   2.136047  -2.041279  -0.972621
H   3.194639   0.877199   1.818408
H   4.673079   0.866237   0.839306
S   2.906836   1.903716  -0.368911
H   1.609169   1.548881  -0.233049
C  -3.627787   0.169563   0.862204
C  -2.994531  -0.790422  -0.202665
O  -4.751162   0.639366   0.548006
O  -2.960659   0.338177   1.898769
H  -3.238360  -1.811240   0.099409
```



```
C  -1.487410  -0.662412  -0.352311
N  -3.713541  -0.538849  -1.502679
H  -3.804323  -1.365518  -2.092536
H  -1.095357  -1.351103  -1.100090
H  -1.014822  -0.891510   0.598054
S  -0.949017   1.017022  -0.886768
H  -4.639830  -0.167603  -1.226378
H  -3.235559   0.197868  -2.032405
H  -1.371167   1.650175   0.227210
28
Dimer 100 of the n...z  type
C  -2.934226  -0.803534  -0.488932
C  -3.128323  -0.522520   1.008693
N  -2.380902  -2.127285  -0.710712
H  -1.531330  -2.258149  -0.170190
H  -2.137578  -2.260550  -1.686814
H  -3.931335  -0.758729  -0.944440
C  -2.139627   0.322572  -1.157808
O  -1.138915   0.154931  -1.825139
O  -2.677437   1.523847  -0.935148
H  -2.129320   2.204922  -1.358313
H  -3.699926   0.390638   1.157988
H  -3.670939  -1.356107   1.453078
S  -1.554108  -0.380046   1.972667
H  -1.235573   0.862082   1.551857
C   3.507443  -0.867620  -0.116615
C   2.242967   0.019764  -0.404193
O   3.292196  -2.106278  -0.087779
O   4.577152  -0.247441   0.019308
H   2.325232   0.366503  -1.435405
C   2.139580   1.207290   0.539835
N   1.037900  -0.877311  -0.343786
H   0.344273  -0.676180  -1.076407
H   3.069484   1.766690   0.473959
H   2.021256   0.872542   1.573109
S   0.711174   2.334154   0.224212
H   1.428338  -1.834011  -0.424780
H   0.539996  -0.816003   0.551934
H   0.944516   2.526603  -1.089967
28
Dimer 101 of the n...z  type
C   1.702945  -0.211906   0.565177
C   1.419062   0.836274  -0.512955
N   2.212319   0.287556   1.845271
H   2.834084   1.079667   1.702262
H   1.462053   0.584988   2.457064
H   0.759922  -0.739787   0.741185
C   2.645103  -1.310663   0.050623
O   2.660775  -1.708610  -1.092575
O   3.438655  -1.821547   0.995645
H   3.254675  -1.268222   1.796643
H   0.948091   0.360558  -1.369896
H   0.740085   1.585228  -0.106344
S   2.907242   1.781382  -1.058211
H   3.513217   0.751452  -1.683336
C  -2.210483  -1.313954  -0.409537
C  -3.013069  -0.035158  -0.830705
O  -2.902224  -2.230350   0.098300
O  -0.988145  -1.276142  -0.642926
H  -3.232848  -0.129746  -1.895801
C  -2.272258   1.272034  -0.587893
N  -4.331251  -0.082873  -0.105804
H  -5.106714   0.323070  -0.629007
H  -2.863979   2.130549  -0.908735
H  -1.351713   1.245143  -1.165347
S  -1.878059   1.576011   1.188311
H  -4.493052  -1.087171   0.087387
H  -4.254882   0.394470   0.798941
H  -1.145065   0.457368   1.354651
```

```
28
Dimer 102 of the n...z  type
C   2.808502  -0.082661   1.007589
C   2.972719  -1.432220   0.301719
N   1.652661   0.044660   1.899509
H   0.887218  -0.545083   1.589763
H   1.881410  -0.222611   2.849158
H   3.714766   0.084985   1.599911
C   2.789848   1.077939  -0.004816
O   3.461928   1.099164  -1.010623
O   1.972985   2.074844   0.335994
H   1.531076   1.760311   1.165573
H   3.909361  -1.457606  -0.252176
H   2.980147  -2.230184   1.043739
S   1.605445  -1.850713  -0.869107
H   1.932774  -0.938011  -1.805908
C  -3.129969  -0.532641   0.796491
C  -1.729810  -0.596341   0.096221
O  -3.990825  -1.318139   0.327526
O  -3.216759   0.267586   1.745460
H  -1.106703  -1.270604   0.687606
C  -1.034282   0.749427  -0.045353
N  -1.920856  -1.266197  -1.234786
H  -1.104079  -1.800299  -1.532755
H  -0.063405   0.638957  -0.526407
H  -0.891290   1.163220   0.949382
S  -1.964512   1.955732  -1.079792
H  -2.750130  -1.872692  -1.120211
H  -2.146708  -0.569470  -1.953150
H  -3.030319   2.038232  -0.256420
28
Dimer 103 of the n...z  type
C  -3.041141  -0.299208  -0.762752
C  -2.133981   0.934792  -0.795876
N  -4.447010  -0.072305  -0.418221
H  -4.535317   0.679448   0.261171
H  -4.997520   0.171684  -1.232571
H  -3.007096  -0.753198  -1.759649
C  -2.472882  -1.384397   0.169611
O  -1.288638  -1.594969   0.311318
O  -3.407669  -2.096845   0.796422
H  -4.262264  -1.667203   0.534069
H  -1.134395   0.663021  -1.128128
H  -2.542589   1.663842  -1.496025
S  -2.010514   1.820815   0.815688
H  -1.383197   0.830207   1.480338
C   3.234094  -1.199934  -0.217510
C   2.679644  -0.188677   0.844717
O   4.488003  -1.241951  -0.298877
O   2.377303  -1.854585  -0.837864
H   2.505270  -0.751053   1.764591
C   1.395058   0.518379   0.439682
N   3.786561   0.790219   1.137004
H   3.770098   1.164043   2.085610
H   1.067698   1.212923   1.214000
H   0.621121  -0.230676   0.289000
S   1.571047   1.533147  -1.090781
H   4.661867   0.271728   0.950408
H   3.745858   1.569589   0.470960
H   1.841753   0.490542  -1.903438
28
Dimer 104 of the n...z  type
C   3.011413  -0.117512  -0.899655
C   1.584733  -0.665641  -0.783471
N   4.099597  -1.088992  -0.769084
H   3.873925  -1.803669  -0.081814
H   4.298025  -1.549991  -1.648787
H   3.097999   0.351289  -1.886449
C   3.248637   1.034726   0.094122
```



```
O   2.399505   1.838898   0.402398
O   4.494756   1.088756   0.567194
H   4.943328   0.293289   0.184604
H   0.863782   0.133047  -0.949659
H   1.433179  -1.445738  -1.529119
S   1.239871  -1.449077   0.855641
H   0.986036  -0.295283   1.507147
C  -1.448589   0.853088   0.020609
C  -2.058512  -0.460088  -0.561566
O  -1.247202   0.868456   1.256749
O  -1.250591   1.742536  -0.831615
H  -1.422847  -0.818030  -1.372662
C  -3.469873  -0.238822  -1.100742
N  -2.041421  -1.521283   0.502604
H  -2.487655  -2.389521   0.202883
H  -3.872398  -1.153783  -1.539289
H  -3.414281   0.521843  -1.877147
S  -4.701184   0.268562   0.176252
H  -1.056269  -1.715871   0.757427
H  -2.510860  -1.168247   1.341305
H  -4.136767   1.457159   0.473047
28
Dimer 105 of the n...z  type
C  -2.942380  -0.341817   0.894715
C  -2.144909   0.944086   1.143541
N  -2.177773  -1.541112   0.554193
H  -1.407195  -1.325617  -0.072283
H  -1.798882  -1.995398   1.376235
H  -3.503073  -0.554330   1.812309
C  -4.028232  -0.124849  -0.175028
O  -4.614248   0.921914  -0.333232
O  -4.290799  -1.210711  -0.903235
H  -3.635364  -1.885670  -0.596922
H  -2.814041   1.752151   1.434408
H  -1.430136   0.777496   1.949850
S  -1.144830   1.493462  -0.304263
H  -2.178807   1.829974  -1.099993
C   4.124618   0.008701   0.122412
C   2.724968   0.521443   0.611083
O   4.719221   0.780958  -0.670498
O   4.489772  -1.084439   0.591160
H   2.880262   1.027627   1.566022
C   1.686153  -0.576837   0.786434
N   2.274504   1.570925  -0.368958
H   1.681304   2.294514   0.037897
H   0.742483  -0.177316   1.155641
H   2.065871  -1.289857   1.513887
S   1.292622  -1.463879  -0.780978
H   3.146626   1.968802  -0.757568
H   1.767981   1.130338  -1.144911
H   2.488299  -2.080863  -0.879865
28
Dimer 106 of the n...z  type
C   2.272230   0.071064   0.957258
C   1.593236  -1.247470   0.572657
N   1.439658   1.275545   0.891323
H   0.712034   1.193372   0.185856
H   1.016754   1.492870   1.784573
H   2.632819  -0.039064   1.985816
C   3.548247   0.303670   0.125133
O   4.323779  -0.577755  -0.171260
O   3.736610   1.575131  -0.225965
H   2.930646   2.042817   0.118108
H   2.285106  -2.075579   0.720037
H   0.707106  -1.401378   1.186030
S   0.963991  -1.296725  -1.160389
H   2.170644  -1.216031  -1.759765
C  -2.386522  -1.253459   0.191660
C  -3.035327   0.090766   0.670945
```

```
O  -1.578199  -1.764539   1.009016
O  -2.764280  -1.669023  -0.916218
H  -4.006663  -0.160616   1.101385
C  -3.228171   1.126189  -0.427602
N  -2.180510   0.612198   1.793201
H  -2.701865   1.085996   2.530004
H  -3.720341   2.020997  -0.044367
H  -3.850972   0.684276  -1.201740
S  -1.646199   1.705886  -1.179481
H  -1.688303  -0.227477   2.153090
H  -1.471602   1.252757   1.422680
H  -1.260680   0.501633  -1.649984
28
Dimer 107 of the n...z  type
C  -2.017388  -0.548083   0.750010
C  -1.595092   0.936187   0.710858
N  -1.009318  -1.430676   0.181232
H  -1.037150  -1.377648  -0.833677
H  -1.217051  -2.394722   0.421835
H  -2.145683  -0.795539   1.807223
C  -3.386531  -0.681431   0.079453
O  -3.562544  -1.127392  -1.031163
O  -4.369652  -0.190097   0.852507
H  -5.211146  -0.241028   0.370706
H  -2.383703   1.553051   1.140293
H  -0.684513   1.073563   1.295668
S  -1.170436   1.595756  -0.963309
H  -2.261033   1.152908  -1.622468
C   2.034254   1.610242   0.535797
C   1.857954   0.167537  -0.020351
O   2.591029   2.429144  -0.235808
O   1.614007   1.774532   1.699637
H   0.877393  -0.223662   0.237195
C   2.924494  -0.767381   0.534714
N   1.923380   0.228534  -1.521100
H   1.009269   0.520827  -1.884813
H   2.879678  -0.723347   1.621457
H   3.925597  -0.471543   0.215093
S   2.705638  -2.515145  -0.028853
H   2.605580   0.955898  -1.773492
H   2.172481  -0.674761  -1.934499
H   1.419745  -2.620711   0.368872
28
Dimer 108 of the n...z  type
C   2.322320   0.446627   1.001668
C   2.849829  -0.810961   0.291931
N   1.320497   0.123978   2.000053
H   0.654437  -0.583400   1.687940
H   0.786731   0.948088   2.255900
H   3.190702   0.899173   1.500571
C   1.882690   1.503499  -0.016040
O   0.817498   2.077964  -0.007637
O   2.845100   1.747370  -0.922638
H   2.539724   2.435897  -1.534989
H   3.811486  -0.611336  -0.174813
H   2.980074  -1.599131   1.033237
S   1.736488  -1.518094  -1.009108
H   2.108097  -0.670219  -1.989613
C  -1.422228  -1.824054   0.416501
C  -1.481147  -0.371955  -0.143333
O  -1.984915  -2.699230  -0.281859
O  -0.846131  -1.936269   1.519219
H  -0.524263   0.119743  -0.015966
C  -2.569706   0.425262   0.566269
N  -1.743064  -0.454226  -1.619866
H  -0.870616  -0.623734  -2.123586
H  -2.336516   0.431994   1.629677
H  -3.550558  -0.034230   0.430194
S  -2.736624   2.157618  -0.049158
```



```
H  -2.350501  -1.271893  -1.773642
H  -2.169757   0.404163  -1.983051
H  -1.451848   2.505057   0.170425
28
Dimer 109 of the n...z  type
C   2.834759   0.735681   0.947841
C   1.356980   0.814717   0.536232
N   3.531572   1.981428   0.669413
H   3.572986   2.133202  -0.335499
H   4.495101   1.917747   0.983031
H   2.843573   0.565704   2.031034
C   3.510556  -0.494859   0.328705
O   4.547359  -0.471613  -0.290911
O   2.808290  -1.618370   0.563661
H   3.268990  -2.372218   0.161845
H   0.807020  -0.049410   0.902341
H   0.924875   1.722591   0.955701
S   1.109174   0.932812  -1.296485
H   1.102201  -0.395406  -1.534339
C  -1.233248  -1.451331  -0.066679
C  -2.096730  -0.150609  -0.007354
O  -1.012107  -1.911255  -1.211661
O  -0.881225  -1.882884   1.048748
H  -1.609560   0.577898   0.639161
C  -3.494489  -0.471182   0.513891
N  -2.161838   0.455454  -1.379207
H  -1.244539   0.877077  -1.596471
H  -3.393668  -0.926842   1.496756
H  -3.997101  -1.183203  -0.144034
S  -4.621937   0.986520   0.608875
H  -2.314224  -0.291452  -2.064887
H  -2.896163   1.166503  -1.446995
H  -3.908929   1.668780   1.528158
28
Dimer 110 of the n...z  type
C  -2.317567  -0.739629   0.210870
C  -3.383858  -0.712095  -0.888902
N  -2.788504  -0.996166   1.573527
H  -3.717181  -0.607667   1.717738
H  -2.824376  -1.987579   1.776595
H  -1.594504  -1.519054  -0.049123
C  -1.482232   0.551503   0.206256
O  -1.148740   1.127399  -0.805484
O  -1.126731   0.970548   1.420109
H  -1.561105   0.327193   2.037629
H  -2.915148  -0.557284  -1.859555
H  -3.914164  -1.664372  -0.901871
S  -4.684364   0.574443  -0.639164
H  -3.881590   1.636796  -0.854160
C   3.385903   1.080233   0.157816
C   3.165127   0.008096  -0.964363
O   4.512286   1.036079   0.714897
O   2.436486   1.860262   0.349319
H   3.386858   0.490293  -1.918982
C   1.761464  -0.577387  -1.011699
N   4.208773  -1.059051  -0.764290
H   4.507706  -1.513922  -1.626591
H   1.667021  -1.312267  -1.812476
H   1.055065   0.229660  -1.188733
S   1.285316  -1.453382   0.540924
H   4.996878  -0.580573  -0.295020
H   3.859760  -1.772414  -0.115115
H   1.223180  -0.339119   1.298397
28
Dimer 111 of the n...z  type
C  -2.955818  -0.443048  -0.750455
C  -1.485200  -0.249779  -0.355636
N  -3.262767   0.214634  -2.009611
H  -3.169824   1.221521  -1.902626
```

```
H  -4.230525   0.044724  -2.264740
H  -3.095449  -1.523219  -0.876061
C  -3.885069  -0.035762   0.400039
O  -4.801570   0.744979   0.302932
O  -3.554554  -0.657286   1.548450
H  -4.167822  -0.380205   2.247946
H  -1.221803  -0.859378   0.505013
H  -0.851681  -0.544096  -1.191315
S  -1.028014   1.505927   0.007309
H  -1.658382   1.596337   1.197010
C   1.459181  -1.513275  -0.033342
C   2.257806  -0.169851  -0.070578
O   1.035202  -1.856537   1.096938
O   1.356881  -2.092056  -1.131135
H   1.938275   0.426888  -0.923731
C   3.752970  -0.465919  -0.152587
N   1.930889   0.607610   1.169129
H   1.021133   1.081464   1.035968
H   3.935624  -1.054733  -1.049387
H   4.075440  -1.048742   0.712841
S   4.826884   1.034283  -0.156665
H   1.814220  -0.067052   1.936044
H   2.646989   1.305197   1.391443
H   4.354129   1.555243  -1.307471
28
Dimer 112 of the n...z  type
C  -1.850737   0.292540  -0.805691
C  -2.337464   0.789994   0.564331
N  -2.860369   0.487162  -1.832970
H  -3.673710  -0.088128  -1.628574
H  -2.508806   0.177183  -2.733372
H  -0.973598   0.902904  -1.044759
C  -1.357912  -1.151867  -0.715147
O  -1.710657  -2.060224  -1.421278
O  -0.444041  -1.323597   0.282479
H  -0.174478  -2.256914   0.307680
H  -1.515953   0.823830   1.273702
H  -2.719187   1.802735   0.441231
S  -3.735724  -0.182682   1.277563
H  -3.013038  -1.270171   1.616859
C   1.495973   1.955070  -0.196547
C   2.349412   0.812438   0.442559
O   0.417372   2.215434   0.388161
O   1.992173   2.469081  -1.217610
H   3.269885   1.243705   0.838162
C   2.667854  -0.283187  -0.569726
N   1.596652   0.243513   1.616167
H   1.311814   0.995098   2.245757
H   3.203063   0.170358  -1.400197
H   1.749236  -0.724628  -0.958557
S   3.635256  -1.699748   0.110501
H   0.737622  -0.215321   1.283217
H   2.156762  -0.445833   2.124017
H   4.762362  -1.006144   0.370744
28
Dimer 113 of the n...z  type
C  -2.761584  -0.530811   0.425471
C  -1.296606  -0.777871   0.043859
N  -3.674620  -1.248280  -0.448577
H  -3.596544  -0.888896  -1.396532
H  -4.633889  -1.082550  -0.160509
H  -2.877642  -0.918217   1.445021
C  -3.057131   0.972021   0.524814
O  -4.000752   1.523574   0.011098
O  -2.131852   1.613927   1.264954
H  -2.363218   2.555150   1.317641
H  -0.632747  -0.362715   0.797943
H  -1.102448  -1.846618  -0.022577
S  -0.830897  -0.108739  -1.614901
```



```
H  -0.845969   1.199240  -1.282982
C   1.691042  -1.691730   0.836063
C   2.553015  -0.573281   0.156063
O   1.336770  -2.628965   0.083601
O   1.467075  -1.521910   2.050624
H   3.598730  -0.763558   0.405271
C   2.160333   0.832353   0.593513
N   2.432234  -0.720713  -1.335761
H   2.602752  -1.695362  -1.593302
H   2.236404   0.884063   1.676199
H   1.128612   1.052260   0.319524
S   3.156944   2.179002  -0.179974
H   1.459379  -0.505592  -1.621187
H   3.071507  -0.099657  -1.836628
H   4.330695   1.854101   0.399554
28
Dimer 114 of the n...z  type
C  -2.428640   0.723161  -0.064267
C  -1.819890  -0.118775  -1.201845
N  -1.415283   1.124495   0.900869
H  -1.107135   0.317344   1.436878
H  -1.796647   1.790069   1.565958
H  -2.842608   1.622866  -0.530716
C  -3.602725  -0.023335   0.573150
O  -3.639688  -0.394311   1.722160
O  -4.588905  -0.238295  -0.315746
H  -5.304334  -0.733431   0.114744
H  -2.584795  -0.373190  -1.933149
H  -1.048925   0.468182  -1.699822
S  -0.973331  -1.658818  -0.642385
H  -2.083541  -2.322310  -0.257159
C   3.211469  -1.228561   0.204089
C   2.246080  -0.013529   0.406018
O   4.394502  -1.036767   0.588478
O   2.710886  -2.220809  -0.352942
H   1.310514  -0.335150   0.857271
C   1.981417   0.651682  -0.947753
N   2.922365   0.948018   1.344889
H   2.665179   0.776725   2.316378
H   1.400083  -0.044290  -1.550207
H   2.921919   0.840249  -1.469243
S   1.141030   2.291155  -0.845104
H   3.934633   0.752375   1.249218
H   2.692668   1.921943   1.112455
H   0.069623   1.871247  -0.085024
28
Dimer 115 of the n...z  type
C   2.001269  -0.576908  -0.382300
C   1.360644   0.772453  -0.757833
N   1.247198  -1.284662   0.642618
H   1.147776  -0.696495   1.466867
H   1.761795  -2.107277   0.944747
H   2.002743  -1.181300  -1.295995
C   3.470623  -0.370458  -0.001012
O   3.940938  -0.613483   1.085346
O   4.181853   0.127861  -1.028190
H   5.101109   0.258612  -0.745309
H   1.949095   1.249635  -1.539354
H   0.348870   0.628072  -1.131790
S   1.148309   1.949074   0.647941
H   2.444615   1.999197   1.022578
C  -2.542095   1.429095  -0.332245
C  -2.121191   0.040977   0.238578
O  -3.669777   1.848830   0.023849
O  -1.710839   1.934199  -1.111742
H  -1.119911   0.095014   0.658062
C  -2.180650  -1.012501  -0.865481
N  -3.053259  -0.330907   1.360688
H  -2.782772   0.121201   2.234549
```

```
H  -1.552340  -0.672770  -1.686427
H  -3.200280  -1.116774  -1.242058
S  -1.640106  -2.685060  -0.309436
H  -3.987672   0.024348   1.119213
H  -3.067698  -1.345594   1.512289
H  -0.403158  -2.246300   0.091063
28
Dimer 116 of the n...z  type
C   2.048306   0.450176  -0.838414
C   2.062001   1.261937   0.467927
N   0.716647  -0.029048  -1.164706
H   0.408880  -0.739285  -0.499612
H   0.723136  -0.480329  -2.073838
H   2.373626   1.139387  -1.626884
C   3.101508  -0.662855  -0.801037
O   2.882246  -1.827892  -1.031966
O   4.319290  -0.185151  -0.477468
H   4.956175  -0.917583  -0.472206
H   3.029139   1.740774   0.606938
H   1.293586   2.031903   0.411453
S   1.656963   0.292044   1.984171
H   2.792300  -0.436014   2.019158
C  -2.368982  -1.637532   0.280879
C  -2.414016  -0.246122  -0.418632
O  -3.466983  -2.083248   0.690364
O  -1.222487  -2.122333   0.376454
H  -1.826251  -0.276794  -1.331925
C  -1.881619   0.831568   0.520850
N  -3.836022   0.066690  -0.807461
H  -4.108188  -0.432223  -1.655316
H  -0.954899   0.470932   0.957431
H  -2.587305   1.025333   1.331530
S  -1.569982   2.437489  -0.324345
H  -4.449444  -0.272157  -0.055466
H  -3.976285   1.068261  -0.969684
H  -0.610253   1.907719  -1.122137
28
Dimer 117 of the n...z  type
C   3.244285   0.004255   0.897754
C   1.762473   0.397044   0.917343
N   4.208977   1.105258   0.964230
H   3.855948   1.925864   0.478104
H   4.413134   1.367644   1.920832
H   3.420431  -0.651395   1.757929
C   3.571334  -0.870665  -0.326853
O   2.819794  -1.707571  -0.773080
O   4.780393  -0.643648  -0.841855
H   5.140650   0.107451  -0.304152
H   1.137282  -0.494241   0.899115
H   1.549968   0.960840   1.825752
S   1.264098   1.487811  -0.483100
H   1.237951   0.513291  -1.414231
C  -3.673636   0.853822  -0.389141
C  -3.210244   0.060311   0.882097
O  -4.826818   0.564304  -0.798127
O  -2.862061   1.686906  -0.829524
H  -3.420081   0.689858   1.749397
C  -1.737221  -0.321418   0.883799
N  -4.102859  -1.146940   0.994291
H  -4.277882  -1.445005   1.953385
H  -1.466335  -0.855263   1.795951
H  -1.149431   0.591469   0.818822
S  -1.267097  -1.427444  -0.513142
H  -4.979437  -0.875050   0.515123
H  -3.699345  -1.930555   0.469263
H  -1.493717  -0.511376  -1.476890
28
Dimer 118 of the n...z  type
C   1.360987  -0.090916  -0.032623
```



```
C   1.793146   1.241005  -0.665788
N   0.732620   0.112186   1.260665
H   1.424703   0.416174   1.939100
H   0.338156  -0.765277   1.586331
H   0.628533  -0.531749  -0.716194
C   2.525583  -1.082085  -0.000568
O   2.952372  -1.621071   0.992973
O   3.032517  -1.288546  -1.232692
H   3.765190  -1.922102  -1.171399
H   2.143227   1.087501  -1.684779
H   0.932119   1.909194  -0.686457
S   3.090806   2.153290   0.280475
H   4.115991   1.330790  -0.023753
C  -1.559469  -1.740325  -0.374141
C  -2.657185  -0.642193  -0.199669
O  -1.119670  -2.242916   0.690571
O  -1.203234  -1.941061  -1.549723
H  -3.482338  -0.820857  -0.886046
C  -2.036308   0.734064  -0.438700
N  -3.192193  -0.736564   1.205257
H  -3.943170  -1.423625   1.275641
H  -1.627161   0.748439  -1.446952
H  -1.222946   0.905613   0.271161
S  -3.195756   2.145577  -0.206451
H  -2.414498  -1.076906   1.790788
H  -3.548600   0.164205   1.540853
H  -4.069398   1.798823  -1.174216
28
Dimer 119 of the n...z  type
C   2.770743   0.463837  -0.290021
C   1.689689  -0.087006  -1.229909
N   4.053448  -0.178747  -0.511321
H   3.991892  -1.167476  -0.283447
H   4.753753   0.216379   0.108200
H   2.866345   1.529035  -0.534122
C   2.308003   0.430053   1.173641
O   2.957297  -0.002229   2.092512
O   1.072090   0.958306   1.320873
H   0.825572   0.946901   2.260147
H   0.787642   0.516716  -1.159933
H   2.055258  -0.037452  -2.255010
S   1.268855  -1.869344  -0.971681
H   0.934376  -1.785507   0.333474
C  -2.804233  -1.065776   0.791372
C  -1.840758  -0.113968   0.008745
O  -3.522902  -1.808348   0.075753
O  -2.764976  -0.948700   2.029550
H  -0.843301  -0.177571   0.432982
C  -2.350475   1.321781   0.088406
N  -1.782846  -0.595377  -1.414325
H  -0.981165  -1.230336  -1.547533
H  -2.426281   1.595670   1.138693
H  -3.342572   1.402964  -0.360147
S  -1.300836   2.549965  -0.803350
H  -2.652531  -1.141832  -1.548070
H  -1.704977   0.165643  -2.093539
H  -0.216416   2.410038  -0.014575
28
Dimer 120 of the n...z  type
C  -1.601102   0.013046  -0.575840
C  -3.057567   0.407253  -0.889044
N  -1.304721  -1.344158  -0.982743
H  -1.900450  -1.988413  -0.469821
H  -0.339020  -1.598283  -0.762641
H  -0.982214   0.707954  -1.155033
C  -1.294637   0.302992   0.896771
O  -0.991938  -0.524014   1.722494
O  -1.384839   1.623116   1.169213
H  -1.196175   1.769635   2.110571
```

```
H  -3.220776   1.463399  -0.681764
H  -3.242204   0.221597  -1.946953
S  -4.333188  -0.584547   0.003375
H  -4.125061  -0.056227   1.227099
C   2.507066  -1.641308  -0.080097
C   2.203382  -0.155300   0.294379
O   3.700298  -2.000321   0.038343
O   1.517792  -2.279908  -0.494311
H   1.345146  -0.115443   0.960028
C   1.953057   0.642386  -0.984011
N   3.387054   0.399572   1.039722
H   3.332690   0.189377   2.036769
H   1.134056   0.172520  -1.523787
H   2.831967   0.607609  -1.631205
S   1.620144   2.437161  -0.731875
H   4.220930  -0.085572   0.675850
H   3.467856   1.415828   0.931556
H   0.513933   2.300900   0.027623
28
Dimer 121 of the n...z  type
C  -2.440849  -1.015203  -0.590604
C  -2.958922  -0.746548   0.827035
N  -1.454097  -2.085330  -0.615089
H  -0.640173  -1.864357  -0.043396
H  -1.108369  -2.216094  -1.560439
H  -3.314180  -1.346856  -1.170056
C  -1.984998   0.254521  -1.327764
O  -1.159312   0.259175  -2.210716
O  -2.625608   1.363828  -0.914776
H  -2.294435   2.123862  -1.420572
H  -3.814933  -0.075712   0.801316
H  -3.271555  -1.694262   1.265098
S  -1.713546  -0.055644   2.004000
H  -1.667780   1.178951   1.462725
C   2.418310  -1.435036   0.057794
C   2.309899   0.066343   0.488978
O   3.371642  -1.706208  -0.711747
O   1.512273  -2.170333   0.494772
H   2.199524   0.142684   1.568885
C   1.110629   0.679791  -0.219013
N   3.587974   0.756619   0.092837
H   4.278859   0.736882   0.843373
H   0.241928   0.111617   0.092407
H   1.197455   0.593699  -1.302389
S   0.815842   2.463731   0.111875
H   3.970792   0.199368  -0.690173
H   3.437639   1.733557  -0.174922
H   0.694997   2.353339   1.451085
28
Dimer 122 of the n...z  type
C  -1.442266  -0.596294   0.217815
C  -1.819839   0.624994   1.083325
N  -0.668422  -0.210788  -0.946423
H  -1.277695   0.039824  -1.717424
H  -0.057936  -0.972356  -1.234545
H  -0.827457  -1.244768   0.851115
C  -2.699247  -1.399674  -0.119517
O  -3.150132  -1.544426  -1.231357
O  -3.272496  -1.905575   0.988671
H  -4.081989  -2.380282   0.740415
H  -2.377702   0.317437   1.966699
H  -0.902293   1.117645   1.406489
S  -2.762534   1.929854   0.186260
H  -3.911730   1.237598   0.044370
C   2.625107  -1.559542  -0.104226
C   2.225603  -0.120580   0.337499
O   3.756441  -1.954047   0.260748
O   1.764150  -2.136490  -0.803000
H   1.215889  -0.130611   0.733044
```



```
C   2.328116   0.851990  -0.833855
N   3.130989   0.328203   1.455837
H   2.893696  -0.138637   2.332236
H   1.881621   0.384936  -1.707258
H   3.372574   1.076797  -1.059650
S   1.470808   2.452904  -0.522282
H   4.092348   0.050641   1.227053
H   3.075596   1.339348   1.608223
H   0.246178   1.875351  -0.587041
28
Dimer 123 of the n...z  type
C   3.137503   0.180124  -0.083293
C   2.643286  -1.041586  -0.881279
N   3.769184  -0.227141   1.162817
H   3.061840  -0.575437   1.805150
H   4.190949   0.576348   1.618115
H   3.887452   0.672800  -0.711221
C   2.000396   1.185454   0.105601
O   1.497870   1.463806   1.171176
O   1.602821   1.713646  -1.063712
H   0.839267   2.306263  -0.904456
H   2.215750  -0.729676  -1.832488
H   3.499625  -1.686707  -1.073997
S   1.388493  -2.075320   0.012788
H   0.299995  -1.724117  -0.700166
C  -2.335784  -1.577505  -0.197848
C  -2.646103  -0.042629  -0.205906
O  -2.539934  -2.163294   0.891588
O  -1.867925  -2.018223  -1.265682
H  -3.273494   0.210974  -1.057466
C  -1.325461   0.714242  -0.257272
N  -3.404431   0.275461   1.055854
H  -4.413456   0.243390   0.908744
H  -0.798760   0.406173  -1.157618
H  -0.701857   0.468691   0.603155
S  -1.467722   2.552791  -0.233787
H  -3.162447  -0.487088   1.714192
H  -3.172099   1.194394   1.441451
H  -2.144066   2.670153  -1.395079
28
Dimer 124 of the n...z  type
C  -3.075001   0.681195   0.769677
C  -2.616801  -0.783857   0.921005
N  -4.465671   0.757776   0.348169
H  -4.553345   0.366943  -0.586616
H  -4.758912   1.727744   0.282497
H  -2.978413   1.140944   1.758822
C  -2.109316   1.414602  -0.159931
O  -2.361030   1.748229  -1.294362
O  -0.916441   1.626805   0.427232
H  -0.297779   1.927759  -0.257246
H  -1.599422  -0.833355   1.306119
H  -3.292771  -1.279276   1.618011
S  -2.663951  -1.772694  -0.638174
H  -1.458965  -1.341861  -1.096238
C   0.932149  -0.670536  -0.134742
C   2.220029   0.152287   0.192314
O   0.621424  -1.535207   0.716187
O   0.390231  -0.393653  -1.223808
H   2.070381   1.210318  -0.011094
C   3.380980  -0.393154  -0.637305
N   2.494568  -0.007920   1.662977
H   2.000051   0.692205   2.216479
H   3.123549  -0.286219  -1.689168
H   3.532702  -1.454315  -0.428451
S   5.007199   0.406204  -0.299443
H   2.105071  -0.931723   1.917318
H   3.493460   0.057258   1.882611
H   4.654431   1.645799  -0.697304
```

```
28
Dimer 125 of the n...z  type
C   2.046038   0.520998  -0.553058
C   3.355937  -0.208791  -0.914327
N   2.270985   1.648535   0.330178
H   2.571051   1.319853   1.243412
H   1.385772   2.136462   0.445940
H   1.621062   0.889305  -1.488969
C   1.061567  -0.503325  -0.000500
O   0.830898  -0.703584   1.165669
O   0.482254  -1.238732  -0.994112
H   0.059601  -2.022171  -0.604156
H   3.161509  -1.008012  -1.629274
H   4.030817   0.514172  -1.372908
S   4.301068  -0.891960   0.516438
H   3.410376  -1.841807   0.868025
C  -1.466574   1.799424   0.164351
C  -2.505030   0.718303  -0.268614
O  -0.517023   1.985667  -0.635044
O  -1.697346   2.338086   1.262211
H  -3.481050   1.188233  -0.391696
C  -2.586226  -0.401678   0.764443
N  -2.116570   0.177682  -1.621676
H  -2.100394   0.929704  -2.311581
H  -2.895207   0.041635   1.708253
H  -1.607833  -0.858216   0.921107
S  -3.717898  -1.785335   0.313273
H  -1.166118  -0.212342  -1.593836
H  -2.764617  -0.554184  -1.928268
H  -4.861131  -1.070530   0.355504
28
Dimer 126 of the n...z  type
C  -3.230396   0.689880  -0.224950
C  -1.748589   0.468799  -0.560591
N  -3.390250   1.538957   0.943744
H  -3.019900   1.067501   1.765046
H  -4.376356   1.694864   1.127774
H  -3.665915   1.202194  -1.091130
C  -3.974890  -0.647727  -0.121395
O  -4.677223  -0.975350   0.804649
O  -3.754462  -1.423411  -1.200463
H  -4.250210  -2.252223  -1.103224
H  -1.638005  -0.038318  -1.516557
H  -1.246190   1.433227  -0.611023
S  -0.824174  -0.490715   0.722518
H  -1.326217  -1.703880   0.412569
C   3.848651  -1.128298   0.302883
C   3.049246  -0.018308  -0.464193
O   3.566535  -2.309447  -0.021109
O   4.690546  -0.711154   1.119380
H   3.712938   0.384613  -1.230792
C   2.562614   1.098530   0.448289
N   1.910594  -0.688425  -1.183755
H   1.666119  -0.231355  -2.061167
H   3.428724   1.551958   0.923364
H   1.916405   0.703617   1.233537
S   1.556579   2.389474  -0.403410
H   2.214138  -1.663282  -1.334312
H   1.059200  -0.716369  -0.593148
H   2.521405   2.811233  -1.246508
28
Dimer 127 of the n...z  type
C  -2.765321   0.010812  -0.886149
C  -1.302903   0.422684  -0.663010
N  -3.595496   1.155567  -1.228999
H  -3.607837   1.809948  -0.450273
H  -4.557523   0.859830  -1.363133
H  -2.764055  -0.685945  -1.733385
C  -3.300874  -0.793447   0.304990
```



```
O  -4.348763  -0.576189   0.866083
O  -2.464223  -1.788629   0.655994
H  -2.849756  -2.277386   1.400663
H  -0.652382  -0.449034  -0.606465
H  -0.990223   1.045606  -1.501373
S  -1.042009   1.465975   0.840626
H  -1.136520   0.466649   1.741633
C   2.048491  -1.838727  -0.387588
C   2.210595  -0.440244   0.292709
O   3.072287  -2.565543  -0.370675
O   0.934698  -2.043909  -0.905235
H   1.359971  -0.221066   0.933713
C   2.352370   0.630787  -0.788183
N   3.444230  -0.486538   1.153678
H   3.237924  -0.847796   2.085103
H   1.449583   0.622613  -1.394077
H   3.199478   0.405948  -1.439246
S   2.661570   2.329218  -0.142696
H   4.083278  -1.159631   0.700887
H   3.876580   0.437546   1.256123
H   1.491233   2.429403   0.524249
28
Dimer 128 of the n...z  type
C  -2.847069  -0.771612   0.707186
C  -3.709436   0.493194   0.636022
N  -1.723296  -0.768316   1.646540
H  -1.232780   0.122703   1.610472
H  -2.035955  -0.918611   2.598552
H  -3.513029  -1.593449   0.998162
C  -2.323305  -1.196234  -0.675053
O  -2.864143  -0.928711  -1.721949
O  -1.215041  -1.944018  -0.611782
H  -0.966369  -1.936625   0.344471
H  -4.517561   0.348259  -0.079958
H  -4.143310   0.684241   1.617937
S  -2.789691   2.029993   0.205299
H  -2.420821   1.640609  -1.031669
C   3.878671   0.433786   0.076781
C   2.925770  -0.801153  -0.082741
O   4.276178   0.644642   1.250096
O   4.148143   1.043756  -0.973860
H   3.546330  -1.652967  -0.369496
C   1.820228  -0.617118  -1.113025
N   2.372529  -1.110708   1.283155
H   2.122398  -2.090567   1.415463
H   1.202810  -1.511263  -1.202433
H   2.286044  -0.408789  -2.073159
S   0.645556   0.739354  -0.694913
H   3.101201  -0.813390   1.951998
H   1.545617  -0.530104   1.462704
H   1.556706   1.733032  -0.752165
28
Dimer 129 of the n...z  type
C  -3.259467  -0.862482   0.575386
C  -2.201025  -0.010979   1.287139
N  -2.779917  -1.797630  -0.443121
H  -2.021187  -1.388320  -0.982147
H  -2.447131  -2.661644  -0.032462
H  -3.776281  -1.445585   1.346140
C  -4.362911   0.017755  -0.039784
O  -4.745568   1.055449   0.451092
O  -4.888206  -0.482097  -1.159235
H  -4.353721  -1.293680  -1.347621
H  -2.671493   0.615543   2.043230
H  -1.478408  -0.667343   1.773050
S  -1.205136   1.052863   0.158425
H  -2.213248   1.855463  -0.238292
C   2.535095   1.307606  -0.348576
C   2.171772  -0.022916   0.393881
```

```
O   1.969411   1.475112  -1.455474
O   3.331738   2.048845   0.258159
H   1.420354   0.219992   1.146858
C   3.353973  -0.701969   1.072094
N   1.511472  -0.936046  -0.603633
H   0.893162  -1.626173  -0.177645
H   3.047269  -1.618705   1.578344
H   3.760731  -0.013197   1.808534
S   4.688626  -1.195507  -0.102524
H   0.995293  -0.331430  -1.259367
H   2.229948  -1.428607  -1.146205
H   5.001520   0.062161  -0.478022
28
Dimer 130 of the n...z  type
C   3.162303   0.540228  -0.163538
C   2.717331   0.563753   1.308071
N   2.383252   1.452309  -0.978858
H   1.413415   1.146119  -1.056975
H   2.747554   1.463732  -1.926261
H   4.207766   0.873951  -0.165993
C   3.197781  -0.896957  -0.698123
O   2.684426  -1.267395  -1.726348
O   3.888141  -1.715698   0.122803
H   3.898208  -2.608895  -0.256729
H   3.455154   0.076666   1.942028
H   2.608017   1.601246   1.622158
S   1.080189  -0.225333   1.634749
H   1.476961  -1.504318   1.483073
C  -1.191793   1.282732  -0.572666
C  -2.035933   0.112659   0.019357
O  -1.241217   2.363030   0.060370
O  -0.591331   1.007085  -1.631261
H  -1.445835  -0.800837   0.019612
C  -3.314822  -0.062634  -0.794753
N  -2.364215   0.447152   1.448394
H  -1.571693   0.244505   2.060297
H  -3.033960  -0.274164  -1.824536
H  -3.907880   0.854356  -0.782877
S  -4.443483  -1.379931  -0.166961
H  -2.520372   1.463378   1.493851
H  -3.181732  -0.071531   1.785347
H  -3.595502  -2.411058  -0.358967
28
Dimer 131 of the n...z  type
C   2.068600  -0.602345  -0.346196
C   1.764013  -0.304469   1.126264
N   3.369576  -1.238416  -0.505455
H   4.101499  -0.597565  -0.208224
H   3.541844  -1.428941  -1.487688
H   1.293822  -1.301386  -0.680556
C   1.887013   0.634166  -1.229685
O   2.618205   0.943863  -2.141634
O   0.787842   1.340389  -0.895814
H   0.691913   2.087711  -1.507369
H   0.730233   0.002283   1.251574
H   1.915830  -1.215694   1.702225
S   2.871005   0.964084   1.890487
H   2.345501   2.027564   1.248592
C  -1.240728  -1.702123  -0.351816
C  -2.514114  -0.833540  -0.090844
O  -0.661610  -2.128388   0.677708
O  -0.917924  -1.820944  -1.548108
H  -3.330791  -1.155432  -0.733294
C  -2.178699   0.634722  -0.335407
N  -2.928208  -1.048025   1.341307
H  -3.558932  -1.844087   1.437806
H  -1.832040   0.736365  -1.360755
H  -1.366571   0.957911   0.315959
S  -3.564057   1.804761  -0.006659
```



```
H  -2.059235  -1.284502   1.849239
H  -3.390610  -0.224828   1.739411
H  -4.411817   1.306335  -0.930242
28
Dimer 132 of the n...z  type
C  -1.698019  -0.521151  -0.424714
C  -3.093345  -0.535509  -1.082341
N  -1.557136  -1.559973   0.576217
H  -2.087071  -1.315185   1.407182
H  -0.580871  -1.673580   0.846518
H  -0.982036  -0.709899  -1.230635
C  -1.413866   0.889473   0.097265
O  -1.430274   1.212865   1.262284
O  -1.142246   1.745104  -0.905588
H  -0.938633   2.618442  -0.531819
H  -3.147896   0.203814  -1.881100
H  -3.259868  -1.525349  -1.507071
S  -4.503208  -0.275137   0.079679
H  -4.276392   1.030815   0.329715
C   2.245778  -1.807086   0.195095
C   2.829298  -0.363476   0.345741
O   2.614331  -2.438231  -0.821936
O   1.435855  -2.132259   1.087330
H   3.251525  -0.225200   1.338756
C   1.701123   0.632150   0.097421
N   3.934988  -0.191660  -0.660917
H   4.836961  -0.480744  -0.281608
H   0.950404   0.458597   0.863210
H   1.248060   0.463006  -0.880964
S   2.177565   2.408501   0.095919
H   3.717656  -0.829790  -1.442605
H   4.019903   0.778762  -0.979801
H   2.566451   2.467673   1.386019
28
Dimer 133 of the n...z  type
C  -3.336738   0.348169   0.661519
C  -1.828078   0.534541   0.440753
N  -3.604006  -0.510855   1.803262
H  -3.272977  -1.452230   1.606952
H  -4.605038  -0.588406   1.953240
H  -3.743245   1.344708   0.871823
C  -4.022744  -0.114739  -0.629891
O  -4.761778  -1.066108  -0.715409
O  -3.706168   0.678056  -1.672206
H  -4.166549   0.359364  -2.465064
H  -1.645140   1.293129  -0.316406
H  -1.367616   0.853726   1.374674
S  -0.946119  -1.013858  -0.047089
H  -1.240976  -0.969018  -1.361235
C   4.066514  -0.874352  -0.455162
C   2.693552  -0.191946  -0.147193
O   4.631198  -1.412832   0.529659
O   4.452861  -0.765208  -1.633439
H   1.952946  -0.467974  -0.894506
C   2.877237   1.323386  -0.106767
N   2.211027  -0.699579   1.183670
H   1.652699  -1.546470   1.075025
H   3.254076   1.644790  -1.075534
H   3.607199   1.601983   0.656201
S   1.345620   2.258672   0.318746
H   3.058472  -0.944519   1.720358
H   1.634026  -0.007935   1.672148
H   0.613608   1.813261  -0.721111
28
Dimer 134 of the n...z  type
C  -2.142654   0.622548   0.080002
C  -3.417016   0.619132   0.944296
N  -2.360837   1.233563  -1.218384
H  -2.970867   0.644057  -1.777926
```

```
H  -1.460530   1.279732  -1.689773
H  -1.397824   1.213348   0.619897
C  -1.591991  -0.800658   0.002426
O  -1.696114  -1.542144  -0.946887
O  -1.001159  -1.164563   1.160747
H  -0.723278  -2.091933   1.093442
H  -3.202388   0.223441   1.936623
H  -3.767620   1.646603   1.044586
S  -4.852107  -0.297906   0.228664
H  -4.319728  -1.534327   0.313053
C   1.193399   0.953943  -0.120438
C   2.354913  -0.061023   0.136614
O   0.646739   0.863537  -1.251016
O   0.960559   1.745181   0.807833
H   2.193506  -0.576299   1.080931
C   3.686879   0.684799   0.146021
N   2.315775  -1.079846  -0.973888
H   1.827303  -1.931945  -0.699445
H   3.648352   1.431979   0.935809
H   3.842530   1.199333  -0.804795
S   5.166791  -0.392560   0.361819
H   1.761379  -0.622812  -1.723831
H   3.249215  -1.349155  -1.296159
H   4.819065  -0.889107   1.566819
28
Dimer 135 of the n...z  type
C   1.959249   0.605212  -0.058433
C   2.686015  -0.189499  -1.159045
N   2.731660   1.755998   0.370062
H   3.582921   1.447794   0.832417
H   2.208608   2.296726   1.051672
H   1.024878   0.966594  -0.501723
C   1.563049  -0.327626   1.088029
O   1.943560  -0.244772   2.226172
O   0.715866  -1.304373   0.663502
H   0.507122  -1.899558   1.402359
H   2.045590  -0.975502  -1.555411
H   2.937194   0.497537  -1.966620
S   4.291646  -0.934549  -0.636490
H   3.772207  -1.910066   0.136900
C  -3.579615  -1.123216   0.127315
C  -2.339704  -0.169435   0.019505
O  -3.665106  -1.994068  -0.777073
O  -4.337769  -0.896129   1.085696
H  -1.760702  -0.235734   0.939028
C  -2.785220   1.267386  -0.224417
N  -1.476757  -0.686844  -1.101307
H  -0.616133  -1.104147  -0.732008
H  -3.457815   1.555497   0.580369
H  -3.329648   1.346636  -1.168083
S  -1.400609   2.480733  -0.352658
H  -2.064334  -1.424822  -1.542647
H  -1.219350   0.028403  -1.783029
H  -0.946493   2.325419   0.907757
28
Dimer 136 of the n...z  type
C  -2.761527  -0.937487   0.081505
C  -1.340963  -0.396244   0.302920
N  -2.935183  -1.431281  -1.275055
H  -2.810665  -0.665711  -1.932963
H  -3.885313  -1.764773  -1.404974
H  -2.882517  -1.776089   0.777915
C  -3.813886   0.096832   0.496889
O  -4.729495   0.469461  -0.197788
O  -3.601414   0.547552   1.749129
H  -4.293351   1.189176   1.976141
H  -1.177310  -0.170789   1.355159
H  -0.617228  -1.143502  -0.024178
S  -0.941024   1.108373  -0.691476
```



```
H  -1.674677   1.983020   0.027078
C   2.293164  -1.796772  -0.079264
C   2.314780  -0.312282   0.408217
O   3.296969  -2.481348   0.234821
O   1.297984  -2.107478  -0.761648
H   1.359222  -0.036896   0.846089
C   2.641334   0.602312  -0.770147
N   3.364032  -0.183416   1.480316
H   2.997909  -0.447427   2.395283
H   1.873249   0.460297  -1.527241
H   3.608159   0.339072  -1.204002
S   2.759317   2.387654  -0.331379
H   4.108734  -0.857336   1.247946
H   3.728349   0.772938   1.544605
H   1.461047   2.520834   0.015166
28
Dimer 137 of the n...z  type
C  -3.168408   0.584256   0.029000
C  -2.680300   0.343600  -1.408854
N  -2.373925   1.591427   0.706059
H  -1.418764   1.267354   0.857369
H  -2.756835   1.768532   1.629393
H  -4.198806   0.951117  -0.060403
C  -3.277781  -0.739148   0.797225
O  -2.812132  -0.944839   1.892299
O  -3.973871  -1.662429   0.101900
H  -4.032352  -2.476464   0.627264
H  -3.412390  -0.228159  -1.975138
H  -2.532377   1.308822  -1.891938
S  -1.056270  -0.524532  -1.535938
H  -1.495585  -1.756581  -1.211740
C   1.186603   1.302534   0.421058
C   2.066782   0.093962  -0.025888
O   1.206047   2.297187  -0.341532
O   0.591133   1.143620   1.505537
H   1.506379  -0.830627   0.090020
C   3.352282   0.059167   0.796601
N   2.379474   0.264034  -1.487729
H   1.604728  -0.062858  -2.067720
H   3.079359  -0.025277   1.846583
H   3.919421   0.982609   0.662688
S   4.515463  -1.296866   0.337132
H   2.469207   1.276607  -1.656705
H   3.228795  -0.238673  -1.763344
H   3.688797  -2.316810   0.645764
28
Dimer 138 of the n...z  type
C   2.392833   1.033060  -0.806549
C   2.213908   1.706301   0.569463
N   1.136441   0.943029  -1.532328
H   0.576372   0.187087  -1.154359
H   1.308846   0.706462  -2.504334
H   3.080292   1.670297  -1.369781
C   3.085373  -0.315607  -0.606766
O   2.524925  -1.387770  -0.664605
O   4.383538  -0.171048  -0.303374
H   4.772644  -1.043838  -0.129867
H   3.179898   1.833649   1.056734
H   1.763072   2.686538   0.415997
S   1.066930   0.842515   1.727823
H   1.767075  -0.300906   1.867241
C  -3.290644   0.819054  -0.616743
C  -2.063294   0.102278   0.031951
O  -4.313187   0.898699   0.108582
O  -3.116967   1.187264  -1.793503
H  -1.160429   0.679814  -0.138950
C  -1.942122  -1.306438  -0.553998
N  -2.283470   0.040061   1.518033
H  -1.927090   0.875924   1.981842
```

```
H  -1.722630  -1.213718  -1.616059
H  -2.890452  -1.837761  -0.452023
S  -0.705240  -2.408220   0.259143
H  -3.305252   0.013702   1.660604
H  -1.819603  -0.774578   1.935108
H   0.416614  -1.762076  -0.124486
28
Dimer 139 of the n...z  type
C  -3.033125  -0.169469   0.566020
C  -1.848656  -1.060959   0.173592
N  -4.268366  -0.622317  -0.053256
H  -4.181813  -0.572658  -1.065425
H  -5.034625  -0.006897   0.201071
H  -3.138086  -0.256355   1.655287
C  -2.750578   1.317986   0.322902
O  -3.549746   2.110025  -0.104088
O  -1.485564   1.679420   0.673342
H  -1.390920   2.638982   0.549255
H  -0.991149  -0.888155   0.818084
H  -2.144152  -2.101697   0.304376
S  -1.344852  -0.934325  -1.597174
H  -0.960632   0.357813  -1.567481
C   1.616282  -1.674075   0.531054
C   1.935737  -0.228966   0.024969
O   1.096931  -1.741796   1.673155
O   1.948978  -2.585370  -0.247364
H   1.502026  -0.081326  -0.961699
C   3.449169  -0.026553  -0.018393
N   1.299842   0.759637   0.963544
H   0.313732   0.941333   0.742631
H   3.868904  -0.768819  -0.693516
H   3.885806  -0.178601   0.971188
S   3.982200   1.664661  -0.526446
H   1.314397   0.336525   1.901260
H   1.793383   1.658025   0.954066
H   3.447453   1.629198  -1.764422
28
Dimer 140 of the n...z  type
C   2.542424  -0.838973  -0.856031
C   1.298508  -1.103985   0.006434
N   3.632752  -1.730559  -0.500645
H   3.932442  -1.541248   0.452429
H   4.436858  -1.553401  -1.094273
H   2.246994  -1.041323  -1.892511
C   2.926945   0.644303  -0.821304
O   4.019657   1.070491  -0.539543
O   1.887081   1.439239  -1.160875
H   2.182375   2.364387  -1.153948
H   0.451413  -0.534835  -0.366146
H   1.048706  -2.162245  -0.054296
S   1.521097  -0.756377   1.804007
H   1.470380   0.589269   1.716080
C  -3.091952  -1.299695  -0.346521
C  -2.377410   0.084282  -0.491676
O  -4.346767  -1.258449  -0.365424
O  -2.323526  -2.265065  -0.186567
H  -1.560320   0.009476  -1.205804
C  -1.874415   0.533937   0.876793
N  -3.373602   1.074108  -1.034692
H  -3.416626   1.044010  -2.053573
H  -1.221697  -0.239562   1.277238
H  -2.711000   0.657657   1.568133
S  -0.997622   2.155183   0.871319
H  -4.296303   0.773980  -0.682974
H  -3.156983   2.035118  -0.752440
H  -0.032044   1.804034  -0.002680
28
Dimer 141 of the n...z  type
C   1.723837  -0.175632  -0.506670
```



```
C   1.833416   0.708365   0.745464
N   0.899420  -1.345105  -0.252165
H   1.326431  -1.915473   0.473062
H   0.847001  -1.926410  -1.082781
H   1.236182   0.435727  -1.276153
C   3.108119  -0.508299  -1.072315
O   3.489299  -1.618885  -1.354612
O   3.858081   0.599536  -1.229337
H   4.718833   0.350479  -1.602465
H   2.293978   1.663397   0.503332
H   0.832431   0.890916   1.133501
S   2.753092  -0.067017   2.145903
H   3.982164   0.033154   1.598867
C  -3.558847  -1.127918  -0.093066
C  -3.096264   0.362141   0.020564
O  -3.276580  -1.852278   0.892812
O  -4.110260  -1.424167  -1.169484
H  -3.881417   1.034436  -0.319204
C  -1.819464   0.544356  -0.795941
N  -2.831131   0.660103   1.472861
H  -3.694957   0.865346   1.975686
H  -2.042720   0.303685  -1.833460
H  -1.042409  -0.141039  -0.444509
S  -1.074876   2.229259  -0.695137
H  -2.440350  -0.198622   1.886417
H  -2.194021   1.454603   1.591081
H  -2.099726   2.897217  -1.263754
28
Dimer 142 of the n...z  type
C   2.202428  -0.833564   0.878269
C   1.526937   0.547199   0.817457
N   3.396922  -0.801776   1.701190
H   4.094629  -0.199903   1.271466
H   3.809194  -1.727333   1.761227
H   1.473710  -1.512172   1.336694
C   2.444576  -1.367411  -0.535696
O   3.517754  -1.643387  -1.005395
O   1.283982  -1.496507  -1.230754
H   1.478436  -1.830271  -2.122125
H   0.587130   0.492262   0.277946
H   1.323842   0.878288   1.835624
S   2.533943   1.885512   0.051778
H   2.381185   1.485970  -1.227583
C  -3.328824  -1.151479   0.523791
C  -2.154404  -0.183386   0.143931
O  -3.604482  -2.021533  -0.342202
O  -3.858981  -0.930536   1.625846
H  -1.408042  -0.212392   0.934844
C  -2.683090   1.236006  -0.044342
N  -1.535906  -0.714911  -1.122460
H  -0.615971  -1.146664  -0.978890
H  -3.170578   1.542844   0.878324
H  -3.425416   1.261166  -0.845244
S  -1.406409   2.478316  -0.527445
H  -2.212806  -1.445755  -1.429176
H  -1.419663  -0.000300  -1.843020
H  -0.715233   2.461325   0.629059
28
Dimer 143 of the n...z  type
C  -2.416588   0.790390   0.112898
C  -3.806726   0.401879   0.652584
N  -2.475973   1.236175  -1.267179
H  -2.739452   0.460210  -1.868272
H  -1.553174   1.541577  -1.563420
H  -2.069379   1.618997   0.736037
C  -1.451736  -0.373896   0.341500
O  -1.091634  -1.151179  -0.511354
O  -1.059564  -0.451849   1.628859
H  -0.488866  -1.229504   1.741524
```

```
H  -3.742068   0.140533   1.708406
H  -4.468605   1.261155   0.543918
S  -4.652114  -0.962363  -0.259188
H  -3.815282  -1.954619   0.107346
C   3.625943  -1.272653  -0.159743
C   2.904061   0.000535   0.395747
O   4.770042  -1.069630  -0.637371
O   2.959891  -2.321453  -0.099218
H   2.450730  -0.212026   1.361644
C   1.848104   0.453166  -0.607744
N   3.945068   1.071342   0.590588
H   4.381316   1.009521   1.510729
H   1.140800  -0.361161  -0.747789
H   2.305482   0.682235  -1.572650
S   0.947692   1.989045  -0.123036
H   4.680237   0.875749  -0.109311
H   3.559707   2.014585   0.482387
H   0.497421   1.529566   1.062411
28
Dimer 144 of the n...z  type
C  -3.018788  -0.890141   0.721862
C  -1.538803  -0.847324   0.308985
N  -3.690467  -2.057089   0.174765
H  -3.705365  -1.995784  -0.840271
H  -4.660794  -2.072257   0.472796
H  -3.032239  -0.957903   1.816009
C  -3.710282   0.434793   0.377238
O  -4.711064   0.537988  -0.291446
O  -3.062073   1.486143   0.914133
H  -3.527441   2.303227   0.673784
H  -1.022199  -0.046407   0.833479
H  -1.073874  -1.798679   0.565960
S  -1.263165  -0.635997  -1.505226
H  -1.545739   0.682143  -1.548615
C   3.838782  -0.990973   0.483975
C   3.270242   0.446961   0.240963
O   4.141423  -1.627287  -0.556375
O   3.878572  -1.345297   1.675958
H   3.732190   1.160045   0.920289
C   1.755549   0.419311   0.428564
N   3.618849   0.846048  -1.168766
H   4.533321   1.295414  -1.217415
H   1.545189   0.106703   1.449325
H   1.298954  -0.299910  -0.253704
S   0.906196   2.015597   0.068404
H   3.682510  -0.037744  -1.701133
H   2.927782   1.485766  -1.573378
H   1.505454   2.730934   1.042353
28
Dimer 145 of the n...z  type
C   2.739778  -0.858226  -0.451703
C   1.604733  -1.222356   0.519615
N   4.044869  -1.080738   0.149266
H   4.155743  -0.470453   0.955203
H   4.778338  -0.824810  -0.504304
H   2.632053  -1.524677  -1.315781
C   2.555872   0.561917  -0.999938
O   3.390958   1.432878  -0.959813
O   1.333356   0.732407  -1.544364
H   1.257051   1.640653  -1.878641
H   0.645069  -1.248297   0.006096
H   1.803128  -2.216088   0.921393
S   1.494574  -0.127563   2.003279
H   1.073190   0.984899   1.366564
C  -2.332211  -1.602455  -0.513168
C  -2.624534  -0.063993  -0.545858
O  -3.076992  -2.274601   0.241836
O  -1.358929  -1.962673  -1.201007
H  -2.693210   0.289452  -1.572529
```



```
C  -1.501288   0.647303   0.198382
N  -3.952773   0.170700   0.123172
H  -4.721430   0.144352  -0.546847
H  -0.578445   0.426472  -0.326313
H  -1.419312   0.271952   1.219706
S  -1.673627   2.471953   0.359581
H  -4.082002  -0.627092   0.768733
H  -3.991870   1.068022   0.615521
H  -1.599353   2.748409  -0.958836
28
Dimer 146 of the n...z  type
C  -1.562686   0.461647   0.091160
C  -1.300648  -0.930087  -0.508484
N  -1.126103   0.530238   1.474881
H  -1.656970  -0.130151   2.036375
H  -1.312161   1.451648   1.857845
H  -0.970746   1.174125  -0.496178
C  -3.021723   0.882037  -0.116035
O  -3.765385   1.250493   0.761357
O  -3.382015   0.795905  -1.411025
H  -4.306629   1.076468  -1.503085
H  -1.483359  -0.923720  -1.581292
H  -0.262657  -1.212289  -0.328284
S  -2.289975  -2.285764   0.259027
H  -3.478392  -1.942163  -0.279525
C   2.824730  -1.421863   0.139954
C   2.877503   0.067338  -0.335181
O   1.946338  -2.121468  -0.428858
O   3.607386  -1.719343   1.058467
H   3.902455   0.420736  -0.419105
C   2.088348   0.905031   0.676387
N   2.252110   0.127690  -1.702552
H   2.938966  -0.026873  -2.440449
H   2.664839   0.941313   1.598983
H   1.120070   0.454849   0.907169
S   1.663982   2.612734   0.135135
H   1.578741  -0.656102  -1.735009
H   1.790494   1.028823  -1.872421
H   2.925458   3.077005   0.015750
28
Dimer 147 of the n...z  type
C   1.760781  -0.229865   0.122490
C   2.663087  -1.302500   0.756300
N   1.329099  -0.611189  -1.210645
H   2.144029  -0.797639  -1.789650
H   0.847990   0.168210  -1.648942
H   0.886274  -0.153080   0.775598
C   2.440546   1.140036   0.178386
O   2.762898   1.795233  -0.783792
O   2.651312   1.530476   1.450408
H   3.091613   2.395307   1.447823
H   2.875220  -1.051075   1.793742
H   2.139702  -2.258256   0.725720
S   4.256225  -1.599983  -0.128605
H   4.840434  -0.426375   0.189153
C  -3.676309  -1.368854   0.061418
C  -3.191091   0.046434  -0.400403
O  -4.447718  -1.371076   1.052591
O  -3.201676  -2.320627  -0.584082
H  -3.263376   0.138631  -1.482054
C  -1.751377   0.252786   0.061063
N  -4.103467   1.072584   0.218021
H  -4.929406   1.234706  -0.358785
H  -1.139441  -0.520717  -0.400460
H  -1.677659   0.150623   1.146058
S  -1.050879   1.918564  -0.310803
H  -4.430018   0.661631   1.107688
H  -3.633724   1.971475   0.365755
H  -1.208957   1.859210  -1.649283
```

```
28
Dimer 148 of the n...z  type
C  -2.124499   0.128423   0.757489
C  -1.580485  -1.204413   0.220492
N  -3.013517  -0.075955   1.888242
H  -3.833825  -0.596448   1.587585
H  -3.352087   0.817564   2.231169
H  -1.254598   0.703411   1.096408
C  -2.746762   0.959157  -0.370522
O  -3.838214   1.471884  -0.333808
O  -1.913035   1.062643  -1.428834
H  -2.327803   1.612952  -2.112576
H  -0.802375  -1.031313  -0.519419
H  -1.158651  -1.767598   1.052439
S  -2.860533  -2.308807  -0.521004
H  -3.044349  -1.593449  -1.649803
C   3.802045  -0.900489  -0.321247
C   2.442228  -0.206468   0.021746
O   4.560731  -1.100439   0.659442
O   3.983448  -1.127629  -1.531245
H   1.624443  -0.693570  -0.504168
C   2.527039   1.272524  -0.351559
N   2.204436  -0.358002   1.500724
H   1.775791  -1.257004   1.722334
H   2.722549   1.342403  -1.419665
H   3.353852   1.749439   0.179026
S   1.033756   2.264859   0.078763
H   3.136380  -0.350756   1.943732
H   1.604321   0.384760   1.873905
H   0.187507   1.676392  -0.790699
28
Dimer 149 of the n...z  type
C  -3.275450   0.909784   0.623726
C  -4.030846  -0.428104   0.569879
N  -3.789224   1.846044  -0.362967
H  -3.627356   1.479162  -1.297549
H  -3.281008   2.723151  -0.309287
H  -3.458566   1.324072   1.622883
C  -1.760352   0.682860   0.550063
O  -1.047465   1.259840  -0.262712
O  -1.320044  -0.176016   1.444753
H  -0.319943  -0.323313   1.359162
H  -3.734969  -1.071426   1.395357
H  -5.099304  -0.227720   0.644616
S  -3.826392  -1.360249  -1.011365
H  -2.570136  -1.795923  -0.784617
C   1.902700  -1.081249   0.321388
C   2.242796  -0.116730  -0.851616
O   1.205507  -0.591000   1.261077
O   2.361743  -2.225327   0.225182
H   1.715664  -0.467770  -1.741695
C   3.739365  -0.069404  -1.154466
N   1.709226   1.254195  -0.551987
H   1.891078   1.904309  -1.318218
H   3.946971   0.590793  -1.998052
H   4.068658  -1.072690  -1.413896
S   4.769403   0.569938   0.235358
H   0.677024   1.225784  -0.381751
H   2.156987   1.622079   0.291696
H   4.599519  -0.490128   1.051896
28
Dimer 150 of the n...z  type
C  -2.957711   0.171413  -1.047620
C  -3.840760   0.618590   0.128076
N  -3.346417  -1.147424  -1.525777
H  -3.185135  -1.833507  -0.792433
H  -2.752054  -1.421810  -2.301956
H  -3.132464   0.895858  -1.852201
C  -1.467831   0.298439  -0.688408
```



```
O  -0.684060  -0.630363  -0.771428
O  -1.149884   1.519971  -0.289593
H  -0.165488   1.593636  -0.039381
H  -3.634764   1.653841   0.390205
H  -4.887335   0.529153  -0.162910
S  -3.679645  -0.419610   1.647845
H  -2.456044   0.014305   2.013973
C   2.168283   1.270557  -0.379591
C   3.403053   0.561358   0.248610
O   1.309358   1.691232   0.459609
O   2.144303   1.352239  -1.610275
H   4.272162   1.202810   0.095545
C   3.667023  -0.809471  -0.374131
N   3.176553   0.455477   1.733225
H   4.018492   0.603777   2.288811
H   4.612700  -1.223742  -0.023231
H   3.718223  -0.683382  -1.453054
S   2.394497  -2.073166   0.056602
H   2.450993   1.153117   1.964041
H   2.785435  -0.471013   1.953428
H   1.316603  -1.414939  -0.432226
28
Dimer 151 of the n...z  type
C   2.494109   0.979204  -0.584999
C   3.599493  -0.024966  -0.946320
N   2.975701   1.981906   0.351942
H   3.236227   1.533242   1.226665
H   2.229949   2.632749   0.577861
H   2.222456   1.483625  -1.520868
C   1.217311   0.258159  -0.134627
O   0.640692   0.531045   0.909064
O   0.811814  -0.656009  -0.993655
H  -0.042337  -1.106922  -0.692211
H   3.273347  -0.686642  -1.745462
H   4.476456   0.527556  -1.282729
S   4.193968  -1.052175   0.468754
H   3.121407  -1.866853   0.542697
C  -2.548554  -1.416880  -0.331873
C  -2.988714  -0.507617   0.850211
O  -1.360030  -1.858283  -0.279777
O  -3.406483  -1.621234  -1.198529
H  -3.784990  -1.023095   1.392133
C  -3.525634   0.844502   0.385606
N  -1.845536  -0.349482   1.815795
H  -2.103393   0.220578   2.623102
H  -3.803575   1.466927   1.237536
H  -4.420215   0.662405  -0.205893
S  -2.344966   1.859351  -0.599300
H  -1.554788  -1.267899   2.154680
H  -0.993950   0.062434   1.368869
H  -2.327779   1.061535  -1.686617
28
Dimer 152 of the n...z  type
C  -3.068110   0.761384   0.339938
C  -3.376148  -0.739667   0.186500
N  -3.489020   1.506883  -0.837549
H  -2.939659   1.205945  -1.638897
H  -3.285707   2.493891  -0.712745
H  -3.650223   1.109936   1.200649
C  -1.588181   0.938295   0.706569
O  -0.796621   1.541682   0.002579
O  -1.275793   0.332743   1.840679
H  -0.263998   0.222217   1.927630
H  -3.212853  -1.258058   1.128875
H  -4.419643  -0.856696  -0.105607
S  -2.401486  -1.594808  -1.131122
H  -1.210172  -1.601861  -0.485494
C   1.534074  -0.851609   0.931751
C   2.984296  -0.721740   0.386583
```

```
O   0.801727  -1.696571   0.377275
O   1.256113  -0.079581   1.885360
H   3.655065  -1.203342   1.100675
C   3.424112   0.724591   0.169856
N   3.045098  -1.513209  -0.890738
H   3.937709  -1.982210  -1.042476
H   4.465937   0.769609  -0.149352
H   3.323614   1.254803   1.113711
S   2.463119   1.593488  -1.141443
H   2.271121  -2.196131  -0.840003
H   2.849524  -0.893718  -1.687949
H   1.256964   1.547314  -0.524303
28
Dimer 153 of the n...z  type
C   3.266993  -0.834637   0.709391
C   4.164679   0.402978   0.563150
N   3.816057  -1.970911  -0.017970
H   3.838183  -1.754483  -1.011679
H   3.198879  -2.771299   0.079587
H   3.261702  -1.081011   1.778537
C   1.805404  -0.506990   0.355606
O   1.149168  -1.160585  -0.433829
O   1.353645   0.549034   1.015591
H   0.378827   0.753395   0.790364
H   3.826977   1.201978   1.218927
H   5.185917   0.132974   0.831951
S   4.284685   1.064599  -1.157998
H   3.045781   1.594989  -1.214113
C  -2.032021   0.547364   0.945407
C  -3.433525   0.956291   0.400998
O  -1.068919   1.218864   0.459023
O  -1.996242  -0.350238   1.792188
H  -3.868217   1.652537   1.121703
C  -4.387975  -0.214795   0.197811
N  -3.221062   1.726098  -0.875096
H  -3.944984   2.420944  -1.060355
H  -5.370182   0.133563  -0.123503
H  -4.496172  -0.735714   1.145927
S  -3.835434  -1.402347  -1.098227
H  -2.295022   2.172787  -0.794238
H  -3.178283   1.073961  -1.667919
H  -2.738953  -1.834836  -0.442079
28
Dimer 154 of the n...z  type
C   3.064785  -0.768919   0.340320
C   3.382722   0.730463   0.191615
N   3.483292  -1.513629  -0.838624
H   2.934607  -1.209771  -1.639337
H   3.276972  -2.500220  -0.715394
H   3.643437  -1.123534   1.200887
C   1.583439  -0.938337   0.704175
O   0.789598  -1.535340  -0.002748
O   1.273135  -0.335092   1.839948
H   0.261502  -0.220991   1.926871
H   3.219656   1.248063   1.134457
H   4.427974   0.841182  -0.096643
S   2.418538   1.595192  -1.127510
H   1.229089   1.627133  -0.479414
C  -1.532842   0.852112   0.928996
C  -2.984468   0.726494   0.386905
O  -0.796900   1.689973   0.368906
O  -1.257292   0.083280   1.886064
H  -3.652624   1.210441   1.101821
C  -3.428585  -0.718926   0.172208
N  -3.045497   1.516938  -0.891094
H  -3.936355   1.990007  -1.040467
H  -4.471134  -0.761398  -0.145020
H  -3.327848  -1.248482   1.116423
S  -2.472330  -1.591544  -1.140038
```



```
H  -2.268140   2.196231  -0.843515
H  -2.855225   0.895871  -1.688362
H  -1.264440  -1.545945  -0.526177
28
Dimer 155 of the n...z  type
C   2.932645  -0.492350   1.047695
C   3.889395   0.650152   0.671679
N   3.542512  -1.791415   0.805515
H   3.717467  -1.903206  -0.190090
H   2.895334  -2.530274   1.062587
H   2.749403  -0.392348   2.124405
C   1.563527  -0.303418   0.375391
O   1.039189  -1.146022  -0.328701
O   1.021164   0.872441   0.662557
H   0.125870   1.002460   0.208756
H   3.478054   1.610329   0.974815
H   4.839179   0.496001   1.183496
S   4.324572   0.731587  -1.121632
H   3.137181   1.198975  -1.559081
C  -2.418104   1.421468  -0.099234
C  -2.768954   0.245655   0.864673
O  -3.351857   2.193970  -0.387782
O  -1.220391   1.479782  -0.477734
H  -2.337833   0.481202   1.839951
C  -2.257001  -1.118778   0.411880
N  -4.264810   0.243928   1.027200
H  -4.576648  -0.013372   1.963467
H  -2.488349  -1.881279   1.156832
H  -1.178311  -1.075537   0.284334
S  -3.019318  -1.719155  -1.154787
H  -4.575262   1.198190   0.777320
H  -4.687695  -0.397192   0.345088
H  -2.480265  -0.778197  -1.957404
28
Dimer 156 of the n...z  type
C   3.094442   0.956039  -0.178858
C   3.498806  -0.395083  -0.788646
N   3.762921   1.180636   1.094446
H   3.459303   0.478376   1.764628
H   3.479708   2.076626   1.478850
H   3.435421   1.721639  -0.886566
C   1.565700   1.102772  -0.120918
O   0.957397   1.381288   0.896262
O   0.992467   0.898340  -1.299267
H  -0.015247   1.034726  -1.258543
H   3.093099  -0.501782  -1.792108
H   4.586271  -0.444722  -0.841485
S   3.004745  -1.870348   0.208231
H   1.695790  -1.858987  -0.112949
C  -2.431697   1.187829  -0.486866
C  -2.059991   0.331761   0.760808
O  -3.547929   1.739812  -0.465260
O  -1.554689   1.254846  -1.388221
H  -1.320736   0.898633   1.328787
C  -1.472866  -1.039687   0.438710
N  -3.302969   0.215618   1.598415
H  -3.124861   0.230620   2.602275
H  -1.169930  -1.553212   1.351955
H  -0.592461  -0.904178  -0.182314
S  -2.643252  -2.185495  -0.403644
H  -3.904855   1.007316   1.314209
H  -3.809399  -0.645957   1.361384
H  -2.759975  -1.475464  -1.545026
28
Dimer 157 of the n...z  type
C   3.016249   1.046497  -0.220286
C   3.197279  -0.250374  -1.029592
N   3.957013   1.109158   0.887582
H   3.745387   0.371553   1.554604
```

```
H   3.847667   1.985960   1.387530
H   3.239032   1.867266  -0.911340
C   1.547655   1.224756   0.188042
O   1.162782   1.242369   1.341424
O   0.748477   1.348884  -0.866857
H  -0.212536   1.475257  -0.601337
H   2.543537  -0.253904  -1.899721
H   4.231662  -0.305004  -1.368408
S   2.926220  -1.809138  -0.076396
H   1.574349  -1.784859  -0.079143
C  -2.859544   1.070742  -0.342577
C  -2.862355   0.029859   0.823131
O  -3.934801   1.184625  -0.962735
O  -1.796963   1.718965  -0.508497
H  -3.072436   0.586908   1.739398
C  -1.572134  -0.755543   1.011074
N  -4.038983  -0.877993   0.586428
H  -4.476571  -1.217319   1.443019
H  -1.656549  -1.437850   1.858121
H  -0.762042  -0.059797   1.218483
S  -1.132404  -1.826195  -0.423834
H  -4.706398  -0.322028   0.024655
H  -3.754049  -1.684128   0.018439
H  -0.907188  -0.829248  -1.304134
28
Dimer 158 of the n...z  type
C   3.299084  -0.819986   0.709918
C   4.180183   0.429967   0.568217
N   3.874173  -1.952117  -0.003468
H   3.905383  -1.741635  -0.998181
H   3.268050  -2.761181   0.091770
H   3.283481  -1.060265   1.780346
C   1.837373  -0.516132   0.335886
O   1.201532  -1.181925  -0.460009
O   1.360585   0.534197   0.987176
H   0.382976   0.717168   0.756370
H   3.821104   1.228126   1.213588
H   5.201317   0.177231   0.853631
S   4.315409   1.082038  -1.155510
H   3.070139   1.594675  -1.232849
C  -2.022134   0.470395   0.934481
C  -3.444300   0.919357   0.484265
O  -1.076525   1.160928   0.439964
O  -1.955086  -0.472465   1.728377
H  -3.842733   1.559741   1.274295
C  -4.410991  -0.233679   0.239532
N  -3.286926   1.782254  -0.738852
H  -3.992761   2.514982  -0.814770
H  -5.406522   0.137379  -0.006451
H  -4.474198  -0.825417   1.149390
S  -3.924682  -1.317751  -1.169255
H  -2.339827   2.188861  -0.683299
H  -3.322739   1.198875  -1.584039
H  -2.795364  -1.793498  -0.605180
28
Dimer 159 of the n...z  type
C  -3.580688  -0.354403  -0.578062
C  -3.232908   1.122189  -0.838796
N  -4.625884  -0.478207   0.428787
H  -4.281267  -0.114925   1.314238
H  -4.833240  -1.458972   0.591309
H  -3.961161  -0.754155  -1.525410
C  -2.296626  -1.133657  -0.256164
O  -2.121380  -1.733835   0.787301
O  -1.402884  -1.044863  -1.231973
H  -0.474061  -1.302244  -0.904271
H  -2.585095   1.209825  -1.708132
H  -4.153528   1.674747  -1.026006
S  -2.424998   1.983636   0.583392
```



```
H  -1.220665   1.365818   0.496205
C   1.409158  -0.471963   0.277669
C   2.842461  -0.634811   0.861772
O   1.013477  -1.451441  -0.426297
O   0.800316   0.563152   0.574583
H   2.730883  -0.995649   1.886729
C   3.661740   0.650903   0.870254
N   3.532361  -1.726438   0.088546
H   4.212465  -2.249746   0.640700
H   4.630147   0.492259   1.346102
H   3.114302   1.401080   1.435491
S   4.036322   1.298258  -0.813666
H   2.789217  -2.349692  -0.262206
H   4.008426  -1.324995  -0.728749
H   2.762005   1.595213  -1.141891
28
Dimer 160 of the n...z  type
C   3.584363  -0.354135   0.571331
C   3.240970   1.122398   0.837157
N   4.622531  -0.477718  -0.442951
H   4.273628  -0.108827  -1.324424
H   4.824066  -1.458665  -0.611621
H   3.970908  -0.756534   1.515170
C   2.297569  -1.132197   0.256739
O   2.119636  -1.739958  -0.781824
O   1.405303  -1.034581   1.233142
H   0.475760  -1.293753   0.908788
H   2.604074   1.210180   1.714479
H   4.164185   1.674305   1.013138
S   2.414947   1.984211  -0.574450
H   1.211974   1.365929  -0.471162
C  -1.408962  -0.475809  -0.279315
C  -2.844404  -0.643476  -0.856381
O  -1.012466  -1.446328   0.436511
O  -0.799352   0.553906  -0.592993
H  -2.736508  -1.013158  -1.878512
C  -3.663109   0.642582  -0.873540
N  -3.532224  -1.727991  -0.071268
H  -4.212052  -2.257962  -0.617458
H  -4.633550   0.479754  -1.343744
H  -3.117731   1.386958  -1.448347
S  -4.030571   1.306514   0.805373
H  -2.787725  -2.346456   0.285227
H  -4.007948  -1.319143   0.742485
H  -2.754765   1.605226   1.126117
28
Dimer 161 of the n...z  type
C  -3.298366   0.967426   0.591869
C  -4.182729  -0.278387   0.739208
N  -3.938263   1.970339  -0.249209
H  -4.067366   1.590434  -1.184062
H  -3.322286   2.770479  -0.356156
H  -3.191410   1.384764   1.601331
C  -1.869778   0.598253   0.153273
O  -1.285534   1.166340  -0.750711
O  -1.355469  -0.381269   0.881577
H  -0.396994  -0.602180   0.607440
H  -3.769900  -0.958195   1.480607
H  -5.178267   0.029711   1.058667
S  -4.454720  -1.215079  -0.830569
H  -3.217875  -1.746290  -0.916571
C   2.024341  -0.578561   0.840519
C   3.411302  -1.062985   0.320107
O   1.034101  -1.085947   0.226828
O   2.021746   0.209430   1.790889
H   3.720504  -1.893465   0.958886
C   4.493530   0.009836   0.335315
N   3.208749  -1.628552  -1.059844
H   3.863996  -2.373959  -1.296686
```

```
H   5.454582  -0.399981   0.022374
H   4.588992   0.384134   1.351584
S   4.156996   1.414908  -0.808661
H   2.236434  -1.971714  -1.091416
H   3.293685  -0.878519  -1.757004
H   3.067545   1.873099  -0.158355
28
Dimer 162 of the n...z  type
C   3.577707  -0.885135   0.307846
C   3.833106   0.393228   1.119345
N   4.663888  -1.138335  -0.629443
H   4.710516  -0.376874  -1.302446
H   4.462045  -1.974523  -1.168998
H   3.553250  -1.706759   1.034710
C   2.180133  -0.867126  -0.337566
O   1.989699  -1.104888  -1.516089
O   1.227618  -0.581281   0.536156
H   0.295529  -0.575052   0.113250
H   3.099793   0.496355   1.915625
H   4.828352   0.339799   1.560562
S   3.837625   1.947281   0.119435
H   2.507187   2.003835  -0.095342
C  -2.097503  -0.924291   0.226461
C  -3.500449  -0.786436  -0.436323
O  -2.060145  -1.502652   1.328862
O  -1.146626  -0.451464  -0.448713
H  -3.594944  -1.597198  -1.162154
C  -3.744099   0.544150  -1.139583
N  -4.517022  -1.043047   0.641672
H  -5.366199  -1.496946   0.304746
H  -4.725225   0.558864  -1.615630
H  -2.983747   0.670815  -1.906115
S  -3.723037   1.995586  -0.005121
H  -4.039024  -1.630049   1.345416
H  -4.770503  -0.160020   1.101098
H  -2.416231   1.907489   0.317847
28
Dimer 163 of the n...z  type
C   2.892366   1.064444  -0.430254
C   3.333144  -0.408539  -0.472981
N   3.199954   1.793126   0.803328
H   3.316989   1.154174   1.585957
H   4.036492   2.356782   0.715979
H   3.378863   1.569775  -1.271211
C   1.389057   1.205117  -0.711784
O   0.820359   0.629869  -1.623370
O   0.755082   2.021550   0.113385
H   1.472156   2.329830   0.738505
H   2.959402  -0.879996  -1.380511
H   4.422219  -0.450147  -0.484626
S   2.837353  -1.410511   0.992703
H   1.510536  -1.529031   0.731545
C  -1.153932  -1.711745  -0.170303
C  -1.798343  -0.294177  -0.054241
O  -1.375465  -2.315438  -1.245397
O  -0.510776  -2.090669   0.832626
H  -1.136499   0.373178   0.493979
C  -3.157215  -0.387884   0.635249
N  -1.934301   0.254681  -1.442594
H  -0.999802   0.584223  -1.749110
H  -3.016084  -0.802128   1.631839
H  -3.826565  -1.049200   0.081708
S  -4.057881   1.217363   0.755650
H  -2.210313  -0.523258  -2.054828
H  -2.612960   1.019605  -1.490326
H  -3.169623   1.836110   1.560248
28
Dimer 164 of the n...z  type
C   2.697203   0.710480  -0.416193
```



```
C   3.103932  -0.504421  -1.260170
N   3.746113   1.322587   0.401689
H   4.428096   0.628941   0.699131
H   4.233652   2.057683  -0.095999
H   2.313777   1.471003  -1.106498
C   1.503582   0.389121   0.494424
O   0.567598  -0.310012   0.145962
O   1.555873   0.960833   1.683647
H   2.435133   1.430297   1.689129
H   2.250215  -0.870090  -1.827979
H   3.885982  -0.208787  -1.959337
S   3.822328  -1.894282  -0.285326
H   2.673587  -2.285528   0.303206
C  -3.701166  -1.243643  -0.081992
C  -2.958412   0.084650   0.304248
O  -3.437109  -2.235526   0.645813
O  -4.484458  -1.161169  -1.045476
H  -3.698899   0.750824   0.748779
C  -2.300974   0.754646  -0.895357
N  -1.959529  -0.256388   1.377343
H  -1.968275   0.399698   2.155551
H  -3.055474   0.906585  -1.662718
H  -1.514551   0.120822  -1.307366
S  -1.469002   2.357343  -0.517200
H  -2.245142  -1.203443   1.690246
H  -0.988180  -0.309545   1.008018
H  -2.589449   3.061179  -0.256288
28
Dimer 165 of the n...z  type
C  -3.178105   0.130183   0.743128
C  -2.462834  -1.228680   0.846523
N  -4.382110   0.034867  -0.069531
H  -4.126502  -0.219206  -1.020645
H  -4.829608   0.944238  -0.131557
H  -3.469150   0.401056   1.765175
C  -2.184068   1.205911   0.278994
O  -2.343920   1.891999  -0.710237
O  -1.126161   1.274049   1.081050
H  -0.345791   1.733593   0.639741
H  -1.657063  -1.175487   1.575117
H  -3.181128  -1.982930   1.167585
S  -1.777349  -1.866189  -0.747159
H  -0.810969  -0.930179  -0.916923
C   1.518618   1.008742  -0.653145
C   3.030370   0.646679  -0.575050
O   1.162449   1.977944   0.082618
O   0.824004   0.324541  -1.416621
H   3.533309   1.137565  -1.411001
C   3.302657  -0.852775  -0.631110
N   3.580197   1.261013   0.684456
H   4.569764   1.502176   0.623497
H   4.373474  -1.056375  -0.594473
H   2.907555  -1.238237  -1.567637
S   2.566456  -1.790540   0.775383
H   3.010408   2.099384   0.873631
H   3.444134   0.617926   1.473930
H   1.278445  -1.605655   0.416995
28
Dimer 166 of the n...z  type
C   2.930773  -1.220587  -0.034033
C   3.453485  -0.082288   0.858719
N   3.219355  -1.098763  -1.465494
H   3.390419  -0.128691  -1.719804
H   4.019555  -1.653592  -1.743140
H   3.367675  -2.152353   0.340076
C   1.415373  -1.400370   0.138066
O   0.862142  -1.429133   1.224280
O   0.749911  -1.531724  -0.996048
H   1.459240  -1.459789  -1.697106
```

```
H   3.096763  -0.221272   1.878022
H   4.543149  -0.109054   0.866043
S   3.020379   1.612019   0.276559
H   1.676523   1.568948   0.495865
C  -0.940449   1.324525  -0.350873
C  -1.945939   0.155065  -0.113200
O  -0.319449   1.725672   0.668241
O  -0.877645   1.737594  -1.522936
H  -1.725181  -0.644043  -0.819617
C  -3.384184   0.640894  -0.278992
N  -1.728579  -0.405756   1.261112
H  -0.823952  -0.920768   1.300026
H  -3.497072   1.050773  -1.280026
H  -3.610660   1.430619   0.440706
S  -4.666725  -0.652720   0.013206
H  -1.649286   0.367735   1.926963
H  -2.495331  -1.028257   1.532135
H  -4.339779  -1.440119  -1.032122
28
Dimer 167 of the n...z  type
C   2.637144   1.092218  -0.683859
C   2.381448  -0.208574  -1.464368
N   3.863407   1.126723   0.118084
H   4.190555   0.185995   0.324455
H   4.607376   1.637597  -0.340810
H   2.662597   1.908459  -1.413084
C   1.450017   1.422596   0.233252
O   0.291092   1.404149  -0.146305
O   1.787760   1.747948   1.466761
H   2.785572   1.681943   1.462320
H   1.396500  -0.171921  -1.927688
H   3.130786  -0.304052  -2.250154
S   2.534449  -1.749119  -0.464042
H   1.413909  -1.602215   0.297536
C  -1.221531  -1.687954   0.650109
C  -2.304203  -0.562888   0.616597
O  -0.240964  -1.481081   1.411218
O  -1.454930  -2.670129  -0.078107
H  -3.207621  -0.945390   1.095203
C  -2.609337  -0.111707  -0.808683
N  -1.829375   0.599457   1.442654
H  -1.489413   0.260980   2.344079
H  -2.921748  -0.978590  -1.384676
H  -1.714846   0.306721  -1.272939
S  -3.886538   1.214737  -0.934642
H  -1.022174   1.056155   0.965993
H  -2.569758   1.289833   1.581573
H  -4.931413   0.472095  -0.514851
28
Dimer 168 of the n...z  type
C  -3.155888  -0.759552  -0.566649
C  -3.649028   0.563819  -1.166931
N  -4.068778  -1.441954   0.352621
H  -4.651199  -0.773919   0.851865
H  -4.673347  -2.095821  -0.129674
H  -2.942725  -1.435612  -1.402743
C  -1.804722  -0.586292   0.142332
O  -0.912894   0.129652  -0.280483
O  -1.674021  -1.299739   1.246319
H  -2.560758  -1.741881   1.356535
H  -2.898767   0.974781  -1.840173
H  -4.561944   0.381258  -1.733655
S  -4.100300   1.839081   0.085478
H  -2.843371   2.115855   0.488713
C   2.648070  -1.712488  -0.322984
C   2.544113  -0.151693  -0.259697
O   2.284305  -2.316870   0.716443
O   3.113773  -2.170575  -1.384142
H   2.101023   0.227178  -1.179504
```



```
C   3.935987   0.445155  -0.052153
N   1.628704   0.206430   0.871207
H   0.638700   0.208059   0.546955
H   4.565320   0.156789  -0.891266
H   4.388107   0.056954   0.863094
S   3.965780   2.278548   0.146653
H   1.727567  -0.543124   1.571190
H   1.850923   1.122745   1.269314
H   3.492808   2.579879  -1.080141
28
Dimer 169 of the n...z  type
C   2.718210  -0.975525   0.439854
C   3.687896   0.083113   0.989983
N   3.367146  -1.824007  -0.547782
H   3.641006  -1.262115  -1.349992
H   2.708820  -2.513388  -0.897255
H   2.431042  -1.597999   1.295912
C   1.417468  -0.320800  -0.048603
O   0.985732  -0.445546  -1.179745
O   0.816785   0.377994   0.906450
H  -0.023488   0.826660   0.567793
H   3.229624   0.633370   1.808809
H   4.582836  -0.417703   1.358914
S   4.303617   1.289668  -0.265547
H   3.158787   1.990361  -0.399360
C  -2.489054   1.438664  -0.164913
C  -3.222755   0.393432   0.734905
O  -3.197791   2.007244  -1.018226
O  -1.276602   1.644133   0.090329
H  -3.465606   0.903415   1.669958
C  -2.445368  -0.879665   1.053227
N  -4.527739   0.083447   0.053484
H  -5.297519  -0.097645   0.697584
H  -3.017016  -1.512464   1.733544
H  -1.514270  -0.614919   1.548270
S  -2.093437  -1.933763  -0.416179
H  -4.724981   0.905603  -0.543817
H  -4.413502  -0.726956  -0.567114
H  -1.157834  -1.132685  -0.972740
28
Dimer 170 of the n...z  type
C  -2.755807   0.211586   1.077952
C  -3.234301  -1.125098   0.494580
N  -3.879443   1.080365   1.399695
H  -4.393689   1.296004   0.548783
H  -3.537106   1.968248   1.753954
H  -2.233540  -0.033558   2.012027
C  -1.689728   0.872552   0.188389
O  -1.692689   2.051476  -0.101497
O  -0.754748   0.010040  -0.202817
H  -0.014525   0.461427  -0.716119
H  -2.406563  -1.824218   0.402664
H  -3.983090  -1.552006   1.161557
S  -4.074293  -0.986517  -1.144494
H  -2.963842  -0.744503  -1.870739
C   2.347924   1.105120  -0.614736
C   3.546558   0.107897  -0.625581
O   2.452956   2.088399   0.141830
O   1.403027   0.802723  -1.389491
H   4.135568   0.309143  -1.523020
C   3.132515  -1.361370  -0.616676
N   4.411148   0.446619   0.557788
H   5.406438   0.290328   0.399605
H   4.005762  -2.010910  -0.687611
H   2.493782  -1.541799  -1.477612
S   2.259521  -1.884756   0.921325
H   4.220109   1.439125   0.772258
H   4.112207  -0.101861   1.373672
H   1.121577  -1.189164   0.711136
```

```
28
Dimer 171 of the n...z  type
C   3.183279  -0.902455   0.664912
C   3.065155   0.535374   1.191012
N   4.232848  -1.146541  -0.327214
H   4.437054  -0.304990  -0.860413
H   5.091253  -1.472899   0.099439
H   3.362897  -1.552495   1.528367
C   1.847243  -1.382979   0.079382
O   0.769917  -1.150856   0.600367
O   1.958965  -2.078835  -1.037598
H   2.938547  -2.075018  -1.226906
H   2.262238   0.602445   1.923025
H   4.000727   0.818873   1.672846
S   2.789091   1.798024  -0.124430
H   1.504860   1.477018  -0.393233
C  -3.827846  -0.894279   0.353815
C  -2.494238  -0.082373   0.192654
O  -3.905930  -1.941113  -0.340000
O  -4.652833  -0.413248   1.151264
H  -1.982969  -0.076723   1.155367
C  -2.763921   1.346010  -0.265300
N  -1.622218  -0.833689  -0.774135
H  -0.728060  -1.125032  -0.333899
H  -3.435665   1.815964   0.448875
H  -3.250922   1.354131  -1.243043
S  -1.253306   2.385729  -0.471261
H  -2.197752  -1.658985  -1.035171
H  -1.394988  -0.292476  -1.608586
H  -0.873170   2.368382   0.822728
28
Dimer 172 of the n...z  type
C  -3.013766   0.760874   0.656506
C  -2.401902  -0.439733   1.392986
N  -4.293485   0.530479  -0.016592
H  -4.388625  -0.442535  -0.297234
H  -5.081303   0.773125   0.571582
H  -3.148058   1.558722   1.395161
C  -2.023536   1.339528  -0.367813
O  -0.833906   1.465457  -0.140629
O  -2.574513   1.704453  -1.511464
H  -3.534675   1.456659  -1.405923
H  -1.447306  -0.160026   1.835318
H  -3.075417  -0.756336   2.189202
S  -2.168383  -1.936273   0.341397
H  -1.198412  -1.434116  -0.449558
C   3.106579   1.305460   0.627983
C   2.131829   0.197259   0.104209
O   3.451561   2.163000  -0.224248
O   3.450056   1.191174   1.819373
H   1.291886   0.094934   0.788747
C   2.878732  -1.128282  -0.031524
N   1.585475   0.659987  -1.215711
H   0.672218   1.122925  -1.070240
H   3.271895  -1.402991   0.944738
H   3.719043  -1.029894  -0.722177
S   1.866817  -2.514032  -0.706807
H   2.264812   1.356697  -1.564927
H   1.454208  -0.108047  -1.878247
H   0.967667  -2.560801   0.297894
28
Dimer 173 of the n...z  type
C   2.847322  -1.329995  -0.078543
C   3.480349  -0.400633   0.966279
N   3.325019  -1.186146  -1.455347
H   3.642849  -0.236465  -1.633887
H   4.084013  -1.823651  -1.662824
H   3.033352  -2.359548   0.246891
C   1.316744  -1.194770  -0.085285
```



```
O   0.652512  -1.076817   0.928602
O   0.774383  -1.230577  -1.290328
H   1.557962  -1.291686  -1.905729
H   3.039949  -0.584269   1.944759
H   4.550369  -0.599978   1.022882
S   3.342577   1.393124   0.563495
H   2.004909   1.495495   0.715865
C  -3.883429  -0.542281   0.026861
C  -2.743630   0.485294   0.364427
O  -3.954475  -1.533391   0.799403
O  -4.599460  -0.251087  -0.947655
H  -3.225299   1.383392   0.752457
C  -1.898168   0.827199  -0.854513
N  -1.929167  -0.107040   1.482467
H  -1.772954   0.542423   2.250995
H  -2.559934   1.189340  -1.637229
H  -1.375976  -0.054185  -1.229116
S  -0.565500   2.066390  -0.550800
H  -2.507780  -0.911227   1.797653
H  -1.004924  -0.470339   1.176484
H  -1.369543   3.086870  -0.187714
28
Dimer 174 of the n...z  type
C  -2.971324  -0.930255   0.076873
C  -3.207977   0.145527  -0.999389
N  -3.638432  -0.585276   1.322454
H  -3.214707   0.251841   1.714424
H  -3.499225  -1.321889   2.007247
H  -3.414129  -1.853576  -0.312626
C  -1.466048  -1.193801   0.217244
O  -0.833418  -0.950639   1.228279
O  -0.939442  -1.685540  -0.896593
H   0.063333  -1.747023  -0.838028
H  -2.795335  -0.173801  -1.954254
H  -4.281286   0.297701  -1.112312
S  -2.536907   1.816818  -0.588630
H  -1.227701   1.525013  -0.739105
C   2.431387  -1.242280  -0.086591
C   3.143957   0.114526  -0.377641
O   1.661615  -1.649759  -1.012979
O   2.698398  -1.779861   0.990919
H   4.200463  -0.098319  -0.548576
C   3.012556   1.117796   0.767364
N   2.584077   0.668329  -1.660698
H   3.278482   1.130579  -2.247151
H   3.635161   1.995458   0.589731
H   3.344603   0.632580   1.682177
S   1.303086   1.773394   0.988242
H   2.155435  -0.134550  -2.150337
H   1.827621   1.331985  -1.447264
H   0.696372   0.596551   1.265821
28
Dimer 175 of the n...z  type
C  -2.957216   0.635609   0.711819
C  -3.497242  -0.737808   1.131030
N  -3.901713   1.538510   0.051515
H  -4.588990   1.017059  -0.487416
H  -4.393068   2.124186   0.715807
H  -2.590725   1.129583   1.619196
C  -1.713657   0.506904  -0.180535
O  -0.853782  -0.340545  -0.011693
O  -1.633886   1.414588  -1.136920
H  -2.481448   1.931913  -1.053602
H  -2.715052  -1.317178   1.618476
H  -4.317124  -0.598882   1.835626
S  -4.208105  -1.726188  -0.252989
H  -3.038278  -2.011893  -0.860481
C   2.486794   1.714785   0.242590
C   2.447122   0.150605   0.184832
```

```
O   2.251036   2.297777  -0.845124
O   2.781962   2.197593   1.352482
H   1.883039  -0.235137   1.032597
C   3.873068  -0.398556   0.197474
N   1.719060  -0.245518  -1.064018
H   0.700664  -0.310386  -0.862941
H   4.357269  -0.080072   1.118063
H   4.445680  -0.003110  -0.644042
S   3.993533  -2.231539   0.031921
H   1.866411   0.521639  -1.736923
H   2.044357  -1.141520  -1.436223
H   3.343130  -2.538052   1.173252
28
Dimer 176 of the n...z  type
C  -2.833138  -1.329974   0.098015
C  -3.431094  -0.430640  -0.992867
N  -3.338105  -1.131537   1.458222
H  -3.652078  -0.173728   1.595120
H  -4.106167  -1.755511   1.673299
H  -3.024337  -2.368252  -0.195078
C  -1.301844  -1.209902   0.132305
O  -0.616356  -1.136606  -0.871812
O  -0.784474  -1.204219   1.348424
H  -1.580725  -1.234829   1.949692
H  -2.974847  -0.657848  -1.954794
H  -4.502988  -0.614836  -1.063296
S  -3.270371   1.374294  -0.653423
H  -1.926717   1.446461  -0.767142
C   3.817133  -0.596231  -0.067419
C   2.725631   0.497395  -0.345754
O   3.891648  -1.506507  -0.932695
O   4.503550  -0.424938   0.956334
H   3.249495   1.385174  -0.703629
C   1.901335   0.835153   0.888357
N   1.874702   0.002106  -1.482552
H   1.629922   0.734029  -2.147159
H   2.577677   1.169149   1.670903
H   1.364056  -0.041089   1.253000
S   0.589755   2.104500   0.617110
H   2.455582  -0.728287  -1.932389
H   0.993640  -0.446751  -1.162624
H   1.408098   3.114978   0.257587
28
Dimer 177 of the n...z  type
C   2.728038  -1.269214   0.388187
C   3.457690   0.052938   0.665780
N   3.028159  -1.925352  -0.886111
H   3.286616  -1.244842  -1.596535
H   3.778688  -2.599637  -0.798976
H   2.986352  -1.957693   1.200441
C   1.205412  -1.102722   0.493572
O   0.660445  -0.429789   1.351461
O   0.519988  -1.765299  -0.422341
H   1.222547  -2.186613  -0.992476
H   3.144295   0.460851   1.625131
H   4.530877  -0.133612   0.701036
S   3.233947   1.334794  -0.640565
H   1.958112   1.660061  -0.345792
C  -3.446974  -0.780673  -0.448382
C  -2.716732   0.424654   0.243451
O  -3.527041  -1.827792   0.244807
O  -3.883513  -0.558668  -1.592383
H  -3.477090   1.165170   0.493700
C  -1.649634   1.045161  -0.649109
N  -2.140687  -0.085876   1.537650
H  -2.331758   0.524252   2.329811
H  -2.113391   1.353014  -1.582549
H  -0.876264   0.313964  -0.885166
S  -0.764181   2.478653   0.102413
```



```
H  -2.602856  -1.006912   1.664352
H  -1.113232  -0.240006   1.483959
H  -1.747277   3.386962  -0.060627
28
Dimer 178 of the n...z  type
C  -2.895971  -0.434453  -0.757111
C  -2.206176   0.931229  -0.648733
N  -4.310894  -0.469835  -0.375275
H  -4.516883   0.232364   0.331128
H  -4.921396  -0.314674  -1.168383
H  -2.813664  -0.758840  -1.800763
C  -2.160141  -1.522721   0.044924
O  -0.947694  -1.633213   0.098678
O  -2.963111  -2.365724   0.667193
H  -3.876974  -2.013620   0.472243
H  -1.183533   0.896923  -1.019805
H  -2.756771   1.649708  -1.256603
S  -2.203645   1.642685   1.051735
H  -1.292189   0.799177   1.577235
C   1.568913   1.677590  -0.242235
C   2.043979   0.220676   0.078421
O   1.043964   1.834025  -1.373775
O   1.794142   2.516681   0.649118
H   1.666532  -0.081967   1.054389
C   3.571099   0.158900   0.054245
N   1.451268  -0.699424  -0.946749
H   0.512684  -1.027948  -0.649140
H   3.955810   0.835209   0.814120
H   3.954489   0.482349  -0.915823
S   4.276106  -1.526668   0.302714
H   1.331381  -0.140045  -1.803351
H   2.044679  -1.517495  -1.111832
H   3.805221  -1.714079   1.552802
28
Dimer 179 of the n...z  type
C   2.824644  -1.168672   0.146192
C   2.539753  -0.382454   1.432336
N   4.085464  -0.865075  -0.534001
H   4.351141   0.106546  -0.391355
H   4.842298  -1.454728  -0.209314
H   2.828371  -2.231891   0.412886
C   1.682989  -1.041811  -0.877156
O   0.501056  -1.037831  -0.584532
O   2.091672  -0.977534  -2.131791
H   3.086221  -0.969458  -2.061635
H   1.588058  -0.691175   1.861420
H   3.322424  -0.598656   2.159406
S   2.563599   1.449433   1.231273
H   1.411659   1.565112   0.532838
C  -3.737024  -0.789534   0.309285
C  -2.618435   0.309253   0.405789
O  -3.559867  -1.794258   1.046242
O  -4.680504  -0.532835  -0.459309
H  -3.038366   1.146363   0.965297
C  -2.134524   0.776911  -0.959656
N  -1.503961  -0.267425   1.238447
H  -1.939438  -1.097307   1.686720
H  -2.993584   1.125337  -1.527040
H  -1.666704  -0.042972  -1.505754
S  -0.848487   2.097678  -0.909095
H  -0.708657  -0.589811   0.659377
H  -1.152874   0.381765   1.939825
H  -1.606137   3.042100  -0.314260
28
Dimer 180 of the n...z  type
C   2.068460  -0.595580  -0.314030
C   1.864602   0.929273  -0.387044
N   1.365390  -1.177030   0.829768
H   1.797921  -0.851538   1.692686
```

```
H   1.469329  -2.188319   0.825398
H   1.638196  -1.011113  -1.230858
C   3.556661  -0.948648  -0.328803
O   4.143085  -1.504108   0.568955
O   4.143024  -0.551593  -1.471124
H   5.085842  -0.781780  -1.440482
H   2.353736   1.329011  -1.273073
H   0.798416   1.148672  -0.454263
S   2.442422   1.859038   1.095349
H   3.766864   1.764540   0.856211
C  -1.999695   0.669594  -1.101576
C  -2.035153  -0.684137  -0.316985
O  -1.542637   1.654335  -0.464913
O  -2.439253   0.621137  -2.265128
H  -1.444169  -1.415715  -0.871002
C  -3.453594  -1.232603  -0.174961
N  -1.365128  -0.463935   1.007175
H  -0.346864  -0.772188   0.974169
H  -3.445249  -2.202729   0.325012
H  -3.867426  -1.365575  -1.172673
S  -4.594509  -0.188199   0.828335
H  -1.368691   0.560380   1.142898
H  -1.835744  -0.921195   1.787731
H  -4.612055   0.875315  -0.001234
28
Dimer 181 of the n...z  type
C   1.930639  -0.135284  -0.826795
C   1.499320   1.215597  -0.233017
N   0.937606  -1.172399  -0.559817
H   0.770016  -1.245276   0.442196
H   1.286343  -2.079031  -0.860183
H   1.997780   0.008458  -1.911981
C   3.336268  -0.535155  -0.372482
O   3.623993  -1.594830   0.131241
O   4.230758   0.438838  -0.617910
H   5.108550   0.141147  -0.329506
H   2.186647   1.999652  -0.542639
H   0.499589   1.463122  -0.592155
S   1.361304   1.234223   1.606813
H   2.683602   1.247444   1.874855
C  -2.512979   1.201737  -0.495161
C  -2.735417  -0.270514  -0.974452
O  -1.666405   1.855540  -1.154300
O  -3.221654   1.574081   0.460855
H  -3.587643  -0.242038  -1.660499
C  -3.069597  -1.270752   0.125325
N  -1.551243  -0.681873  -1.799441
H  -1.753259  -1.483520  -2.396462
H  -3.130345  -2.283562  -0.276959
H  -4.045460  -1.009988   0.528881
S  -1.869406  -1.365445   1.520144
H  -1.308208   0.126266  -2.381957
H  -0.667687  -0.895295  -1.235944
H  -2.037777  -0.090728   1.926246
28
Dimer 182 of the n...z  type
C   2.848688   0.745066  -0.595587
C   2.653407  -0.609291  -1.299046
N   3.733908   0.744080   0.572509
H   3.816474  -0.192393   0.961806
H   4.660761   1.084812   0.349012
H   3.245226   1.441044  -1.342221
C   1.497388   1.351684  -0.184464
O   0.530908   1.407918  -0.920074
O   1.475024   1.840370   1.049819
H   2.388969   1.643521   1.400673
H   1.866916  -0.518904  -2.046831
H   3.579479  -0.888129  -1.801938
S   2.283532  -2.022656  -0.174707
```



```
H   1.113159  -1.564705   0.357780
C  -1.587825  -1.079080   1.029282
C  -2.471683   0.210383   1.072530
O  -0.397882  -0.911668   1.405171
O  -2.154228  -2.122701   0.659559
H  -2.953798   0.234119   2.053870
C  -3.553935   0.284466   0.003676
N  -1.553983   1.398217   1.025169
H  -2.014856   2.270148   1.286518
H  -4.134164   1.204005   0.096896
H  -4.225533  -0.559164   0.144840
S  -2.925350   0.270422  -1.727577
H  -0.764342   1.217328   1.655983
H  -1.117423   1.506906   0.091689
H  -2.398064  -0.969656  -1.669425
28
Dimer 183 of the n...z  type
C   2.078351   0.314541  -0.980126
C   2.054646  -1.051996  -0.285421
N   3.386982   0.775057  -1.454045
H   4.134213   0.426628  -0.858330
H   3.570873   0.475307  -2.404007
H   1.406226   0.250048  -1.843852
C   1.472234   1.425907  -0.106075
O   0.490547   1.283135   0.601632
O   2.092388   2.587133  -0.208191
H   2.860956   2.396561  -0.815418
H   1.037466  -1.365885  -0.058434
H   2.484767  -1.792328  -0.960547
S   3.080249  -1.142035   1.243687
H   2.272457  -0.393359   2.022033
C  -1.822670  -1.946926  -0.555921
C  -2.402791  -0.729427   0.244104
O  -0.946260  -2.614004   0.051327
O  -2.312042  -2.133297  -1.684936
H  -3.374622  -1.035292   0.635904
C  -2.544015   0.530411  -0.599576
N  -1.509544  -0.494799   1.431512
H  -1.122647  -1.414908   1.682774
H  -3.177970   0.307962  -1.453590
H  -1.571005   0.853709  -0.972246
S  -3.211627   1.988072   0.313771
H  -0.711632   0.116300   1.174564
H  -1.999600  -0.072474   2.219318
H  -4.461083   1.510551   0.489667
28
Dimer 184 of the n...z  type
C  -2.374372  -0.652203   0.581931
C  -2.055479   0.835713   0.817883
N  -1.347809  -1.303064  -0.229241
H  -1.360220  -0.912275  -1.169818
H  -1.560573  -2.292098  -0.330821
H  -2.387106  -1.126796   1.568737
C  -3.780355  -0.825927   0.000789
O  -4.026985  -1.337130  -1.065209
O  -4.717185  -0.333994   0.829409
H  -5.592473  -0.454184   0.426921
H  -2.816542   1.285934   1.451664
H  -1.096900   0.920178   1.328859
S  -1.864298   1.844663  -0.712746
H  -3.164673   1.864989  -1.071018
C   3.662170  -1.102115  -0.223408
C   2.200387  -0.606928  -0.491973
O   3.761629  -2.035732   0.611490
O   4.556893  -0.541541  -0.886728
H   1.841779  -1.143443  -1.375095
C   2.075460   0.889202  -0.754860
N   1.337516  -1.040275   0.651489
H   0.320320  -1.164972   0.375099
```

```
H   1.052042   1.159937  -1.017209
H   2.727198   1.148703  -1.585222
S   2.501157   1.939625   0.699363
H   1.749839  -1.912918   1.007131
H   1.403753  -0.350734   1.407062
H   3.804197   1.593166   0.748238
28
Dimer 185 of the n...z  type
C   2.145927  -0.619826   0.139176
C   1.920293   0.262224  -1.099249
N   1.423258  -0.108606   1.302628
H   1.717845   0.849475   1.487347
H   1.681379  -0.643135   2.128742
H   1.747411  -1.611036  -0.106754
C   3.637494  -0.826396   0.420837
O   4.170441  -0.623175   1.485278
O   4.285861  -1.282374  -0.664303
H   5.221751  -1.414493  -0.442606
H   2.400063  -0.178810  -1.969705
H   0.852257   0.331271  -1.299678
S   2.482073   2.008581  -0.915708
H   3.806243   1.765507  -1.000004
C  -3.303548   1.167095   0.353754
C  -1.993492   0.385989  -0.007963
O  -3.441736   1.438687   1.572875
O  -4.043729   1.455300  -0.606447
H  -1.282072   1.131112  -0.374841
C  -2.176712  -0.683682  -1.078581
N  -1.417796  -0.176930   1.253356
H  -0.357382  -0.173092   1.267605
H  -1.230458  -1.171354  -1.318835
H  -2.561999  -0.210583  -1.978191
S  -3.306949  -2.051401  -0.577730
H  -1.805509   0.407297   2.010422
H  -1.761989  -1.129944   1.403096
H  -4.414224  -1.288270  -0.470561
28
Dimer 186 of the n...z  type
C   2.817600  -0.798306   0.715294
C   2.423947   0.661197   0.986717
N   4.166277  -1.002827   0.174955
H   4.482445  -0.190049  -0.347461
H   4.842630  -1.201339   0.901987
H   2.741502  -1.335032   1.667480
C   1.815562  -1.512908  -0.207753
O   0.607377  -1.451339  -0.077140
O   2.379961  -2.246018  -1.152645
H   3.358042  -2.113601  -1.004276
H   1.394720   0.734699   1.333680
H   3.080851   1.056502   1.761451
S   2.616674   1.783542  -0.469161
H   1.347425   1.691358  -0.931626
C  -1.129902   1.378816  -0.112443
C  -2.027676   0.102487  -0.119479
O  -0.829233   1.811045   1.026309
O  -0.823250   1.832601  -1.234779
H  -1.749790  -0.541372  -0.950919
C  -3.501484   0.493935  -0.197596
N  -1.735082  -0.652366   1.142637
H  -0.828158  -1.129580   1.023930
H  -3.668567   1.040978  -1.123719
H  -3.772285   1.144380   0.636351
S  -4.673929  -0.925858  -0.098625
H  -1.630448   0.038154   1.896680
H  -2.467486  -1.332266   1.365342
H  -4.300295  -1.521055  -1.249866
28
Dimer 187 of the n...z  type
C  -2.592926   1.328222   0.457122
```



```
C  -3.225619   0.042255   1.013970
N  -3.020674   1.732204  -0.886401
H  -3.370320   0.930403  -1.406899
H  -3.745818   2.438711  -0.858044
H  -2.830954   2.134615   1.158865
C  -1.056940   1.246309   0.479881
O  -0.404484   0.833584   1.408052
O  -0.487554   1.722356  -0.638302
H  -1.267088   1.965089  -1.217314
H  -2.729771  -0.231914   1.944027
H  -4.279836   0.223500   1.223879
S  -3.211812  -1.390770  -0.144947
H  -1.859765  -1.519351  -0.295632
C   0.963913  -1.736145  -0.150648
C   2.179544  -0.820742  -0.505054
O  -0.029512  -1.627918  -0.915492
O   1.124730  -2.484761   0.829138
H   2.956362  -1.451411  -0.941741
C   2.727241  -0.072022   0.704868
N   1.764198   0.145047  -1.582431
H   1.376485  -0.370996  -2.373610
H   2.998329  -0.802969   1.462330
H   1.961996   0.582873   1.123555
S   4.172828   1.014020   0.338259
H   1.011424   0.769509  -1.248770
H   2.554726   0.716190  -1.892451
H   5.046585   0.029637   0.042643
28
Dimer 188 of the n...z  type
C   2.430461   0.805823   0.620501
C   2.261402  -0.543829   1.340291
N   1.198401   1.228014  -0.034831
H   0.985349   0.632159  -0.833853
H   1.304587   2.168123  -0.406590
H   2.680515   1.539018   1.394606
C   3.624398   0.770543  -0.338068
O   3.561617   0.968564  -1.527860
O   4.767847   0.486702   0.310854
H   5.498096   0.467241  -0.328481
H   3.176135  -0.807528   1.867198
H   1.459489  -0.453334   2.072610
S   1.761002  -1.946974   0.254355
H   2.941192  -2.101787  -0.380366
C  -1.766767  -0.578262  -0.969463
C  -1.993520  -0.287411   0.548189
O  -1.210690   0.326858  -1.634461
O  -2.183814  -1.693181  -1.347635
H  -1.419304  -1.015323   1.125048
C  -3.462650  -0.409003   0.949232
N  -1.448122   1.066266   0.893614
H  -1.560122   1.279345   1.885751
H  -3.597168  -0.218825   2.015516
H  -3.793586  -1.422455   0.734984
S  -4.582242   0.786953   0.100266
H  -0.414197   1.140465   0.637647
H  -1.945115   1.778799   0.353552
H  -4.467627   0.255763  -1.134338
28
Dimer 189 of the n...z  type
C  -2.137982  -0.388667   0.190683
C  -1.617171   0.989273   0.613428
N  -1.715492  -0.874244  -1.124811
H  -1.610491  -0.098845  -1.775036
H  -0.821251  -1.362308  -1.053814
H  -1.758079  -1.095184   0.937556
C  -3.669979  -0.474967   0.284996
O  -4.331292   0.049123   1.155489
O  -4.214268  -1.220915  -0.677267
H  -3.434527  -1.468396  -1.247521
```

```
H  -2.001756   1.251038   1.597925
H  -0.530327   0.951257   0.667441
S  -2.006835   2.353219  -0.564837
H  -3.335632   2.391540  -0.335967
C   1.346919  -1.436400   0.472386
C   2.782836  -0.824547   0.412868
O   0.977664  -2.024349  -0.580842
O   0.711872  -1.223492   1.517200
H   3.334406  -1.048166   1.323690
C   2.670097   0.687410   0.203933
N   3.501687  -1.466826  -0.742670
H   3.999433  -2.311026  -0.458804
H   2.153334   1.111568   1.062140
H   2.084192   0.907814  -0.691022
S   4.274020   1.553781  -0.054667
H   2.759201  -1.751754  -1.404998
H   4.178429  -0.830561  -1.176985
H   4.804618   1.272974   1.153408
28
Dimer 190 of the n...z  type
C   1.766144  -0.414855  -0.096118
C   2.223963   0.673800  -1.080883
N   0.845742   0.120489   0.901254
H   1.339005   0.777832   1.502599
H   0.509599  -0.628966   1.499811
H   1.232586  -1.166904  -0.687008
C   2.960648  -1.140456   0.528378
O   3.176741  -1.221446   1.713828
O   3.748591  -1.689124  -0.413178
H   4.492026  -2.139746   0.018888
H   2.846460   0.242381  -1.861726
H   1.345858   1.117481  -1.548785
S   3.117825   2.088834  -0.306275
H   4.285660   1.450453  -0.086397
C  -3.750202   0.467542   0.471671
C  -2.660607   0.780353  -0.609489
O  -3.589392   1.043961   1.577469
O  -4.665018  -0.296568   0.108829
H  -3.106561   1.503212  -1.297431
C  -2.221534  -0.430156  -1.425044
N  -1.524874   1.481179   0.081012
H  -1.192276   2.290992  -0.439881
H  -1.395058  -0.174078  -2.089665
H  -3.061487  -0.748442  -2.038251
S  -1.641181  -1.875887  -0.438718
H  -1.935995   1.789875   0.980874
H  -0.682480   0.877224   0.311151
H  -2.827544  -2.122085   0.153170
28
Dimer 191 of the n...z  type
C   2.899246  -1.056630   0.644467
C   3.416219   0.390123   0.653217
N   3.287668  -1.868795  -0.512924
H   3.513920  -1.284064  -1.313651
H   4.081668  -2.464430  -0.312429
H   3.266454  -1.539457   1.555841
C   1.366437  -1.082866   0.767835
O   0.730567  -0.422496   1.555938
O   0.779913  -1.919513  -0.099254
H   1.547945  -2.306356  -0.612201
H   3.030631   0.918738   1.523342
H   4.504934   0.382623   0.703500
S   3.013157   1.348460  -0.870743
H   1.682452   1.456194  -0.662247
C  -3.615490  -0.793771   0.002742
C  -2.229144  -0.065216   0.042740
O  -3.883083  -1.377600  -1.080043
O  -4.298577  -0.698067   1.037963
H  -1.649824  -0.422687   0.892016
```



```
C  -2.420714   1.445241   0.139388
N  -1.487812  -0.455209  -1.208187
H  -0.738557  -1.126637  -0.998724
H  -2.986696   1.656612   1.043767
H  -2.987302   1.824178  -0.714011
S  -0.852797   2.419326   0.146923
H  -2.212528  -0.913658  -1.796182
H  -1.065497   0.341718  -1.688610
H  -0.282246   1.780793   1.189315
28
Dimer 192 of the n...z  type
C   1.965815  -0.687015   0.115283
C   3.465491  -0.844083  -0.190486
N   1.110951  -0.361191  -1.031811
H   1.531296   0.396848  -1.564647
H   1.055081  -1.161151  -1.655115
H   1.620166  -1.650601   0.504883
C   1.698653   0.339970   1.232053
O   2.520854   1.161891   1.592667
O   0.499388   0.290937   1.793284
H  -0.073548  -0.495350   1.487999
H   4.027298  -1.009804   0.729087
H   3.601050  -1.715635  -0.833071
S   4.245011   0.541334  -1.125429
H   4.112245   1.464864  -0.153692
C  -1.901030  -1.669359   0.324827
C  -2.079905  -0.296080  -0.379212
O  -2.656620  -2.589880  -0.034792
O  -1.010743  -1.698979   1.218758
H  -1.096085   0.079027  -0.663832
C  -2.807179   0.684539   0.540946
N  -2.867743  -0.529947  -1.638995
H  -2.256976  -0.785721  -2.416192
H  -2.211788   0.819222   1.441441
H  -3.786841   0.299827   0.832859
S  -3.142249   2.325172  -0.227615
H  -3.492461  -1.330824  -1.464647
H  -3.397243   0.304737  -1.912642
H  -1.852948   2.678835  -0.407881
28
Dimer 193 of the n...z  type
C   2.870393  -0.369761   0.611576
C   3.071848  -0.107593  -0.888802
N   2.569268  -1.754226   0.991329
H   2.084355  -2.238581   0.239278
H   3.407157  -2.275391   1.219721
H   3.791781  -0.066987   1.121296
C   1.784921   0.544972   1.202150
O   1.645285   1.712309   0.932859
O   0.988694  -0.082342   2.085773
H   1.318186  -1.025455   2.079903
H   3.221442   0.956227  -1.065521
H   3.960929  -0.642060  -1.222913
S   1.699923  -0.713538  -1.963457
H   0.831645   0.322751  -1.791468
C  -1.247260   1.693367  -0.585684
C  -1.540025   0.288836   0.035475
O  -1.692044   2.667909   0.062668
O  -0.641300   1.676486  -1.678411
H  -0.675526  -0.362920  -0.070838
C  -2.750652  -0.337452  -0.654512
N  -1.788093   0.483030   1.502634
H  -0.901788   0.401194   2.018217
H  -2.520090  -0.456671  -1.711285
H  -3.625847   0.309037  -0.566079
S  -3.273332  -1.955760   0.056038
H  -2.147246   1.445570   1.606249
H  -2.446588  -0.206620   1.877177
H  -2.132529  -2.622078  -0.217105
```

```
28
Dimer 194 of the n...z  type
C  -2.885787   0.873054  -0.308162
C  -3.204629   0.382765   1.108872
N  -3.083034  -0.091852  -1.393890
H  -2.880420  -1.039909  -1.086517
H  -4.031611  -0.070894  -1.748838
H  -3.531567   1.736623  -0.505660
C  -1.455826   1.426230  -0.408001
O  -0.879710   2.019433   0.472406
O  -0.885286   1.194745  -1.599562
H  -1.541197   0.620046  -2.082527
H  -3.092209   1.193410   1.826572
H  -4.236246   0.032858   1.140731
S  -2.178587  -1.045763   1.670223
H  -1.079530  -0.330528   1.982375
C   3.155905   0.658652   0.765620
C   1.960697   0.015108  -0.021474
O   3.759235   1.581315   0.157826
O   3.356866   0.201500   1.904580
H   1.043959   0.336573   0.471116
C   2.042674  -1.504960  -0.067300
N   1.949769   0.631026  -1.395919
H   1.018581   0.982870  -1.651209
H   2.059169  -1.877107   0.953991
H   2.966006  -1.826176  -0.555079
S   0.689680  -2.330127  -1.012817
H   2.646311   1.400606  -1.320371
H   2.252806  -0.018318  -2.122350
H  -0.325728  -1.981071  -0.191405
28
Dimer 195 of the n...z  type
C   1.704419   0.568771   0.062353
C   1.993179  -0.608822   1.010154
N   0.779562   0.187140  -1.000434
H   1.268600  -0.380713  -1.689596
H   0.453859   1.015493  -1.491303
H   1.223883   1.339302   0.671458
C   3.004114   1.171061  -0.471756
O   3.327186   1.199954  -1.635747
O   3.762045   1.663755   0.524151
H   4.583234   2.021498   0.149940
H   2.672439  -0.301060   1.802644
H   1.056786  -0.924641   1.468767
S   2.659029  -2.119981   0.191916
H   3.906085  -1.658016  -0.035211
C  -3.377008   0.192201  -0.937385
C  -2.784377  -0.904220   0.008736
O  -2.974708   0.145819  -2.125224
O  -4.206801   0.964909  -0.415927
H  -3.495842  -1.735748   0.002716
C  -2.585794  -0.476198   1.456561
N  -1.524355  -1.428288  -0.616209
H  -1.229381  -2.320266  -0.218164
H  -2.118091  -1.272716   2.038248
H  -3.564978  -0.274538   1.884797
S  -1.520417   1.005115   1.703879
H  -1.717668  -1.545121  -1.616206
H  -0.704902  -0.751377  -0.579047
H  -2.327244   1.861546   1.044729
28
Dimer 196 of the n...z  type
C  -1.807302  -0.250966  -0.100078
C  -3.190749  -0.830020  -0.410988
N  -1.415089  -0.229478   1.312194
H  -2.222279  -0.243172   1.928114
H  -0.786264  -0.998444   1.532057
H  -1.075279  -0.858317  -0.645105
C  -1.643048   1.165281  -0.681826
```



```
O  -2.034459   1.499069  -1.779697
O  -0.985602   1.991256   0.131277
H  -0.852834   1.437674   0.954374
H  -3.379797  -0.794272  -1.482567
H  -3.229950  -1.869733  -0.085439
S  -4.584742  -0.001026   0.470623
H  -4.513056   1.174310  -0.187333
C   1.440578  -1.714936   0.077582
C   2.767755  -0.891525   0.088004
O   0.943536  -1.913138  -1.058585
O   0.994903  -2.026421   1.199256
H   3.501908  -1.353463   0.744906
C   2.466746   0.541174   0.534933
N   3.314521  -0.904441  -1.314020
H   3.939374  -1.695729  -1.470773
H   2.087948   0.508831   1.554078
H   1.697817   0.989276  -0.097558
S   3.896569   1.696588   0.430225
H   2.491060  -1.033476  -1.927596
H   3.827502  -0.044483  -1.535440
H   4.677697   1.069259   1.333708
28
Dimer 197 of the n...z  type
C   2.659518   0.767445   0.227156
C   3.765362   0.370350  -0.758404
N   2.985932   0.666694   1.652439
H   3.655903  -0.079925   1.821287
H   3.371769   1.530442   2.014485
H   2.391202   1.808117   0.012307
C   1.364163  -0.019613  -0.031766
O   0.932832  -0.294545  -1.125794
O   0.717810  -0.349669   1.095755
H   1.317498  -0.019994   1.824104
H   3.416181   0.490703  -1.782566
H   4.629779   1.016959  -0.607617
S   4.397391  -1.346561  -0.532177
H   3.298403  -1.981104  -0.988778
C  -3.841296  -1.114904  -0.171042
C  -3.047273   0.153330   0.308348
O  -3.638069  -2.157577   0.505555
O  -4.586724  -0.944363  -1.150895
H  -3.770911   0.870734   0.695149
C  -2.237015   0.775199  -0.821014
N  -2.182927  -0.286322   1.462782
H  -2.337130   0.261065   2.307503
H  -2.914050   0.993720  -1.643217
H  -1.471428   0.084740  -1.179773
S  -1.307291   2.299012  -0.357743
H  -2.485697  -1.273949   1.611899
H  -1.173309  -0.274367   1.257655
H  -2.378406   3.049421  -0.026912
28
Dimer 198 of the n...z  type
C  -1.566437  -0.256689  -0.238466
C  -2.799428  -0.559900  -1.095156
N  -1.365484  -1.108895   0.934187
H  -2.215512  -1.591208   1.206995
H  -0.605031  -1.767512   0.773073
H  -0.690630  -0.362956  -0.888208
C  -1.537792   1.218023   0.210173
O  -1.807591   2.154286  -0.510522
O  -1.143172   1.365337   1.473520
H  -1.054167   0.415738   1.788345
H  -2.857786   0.139017  -1.927863
H  -2.723374  -1.571475  -1.494449
S  -4.397841  -0.530662  -0.173282
H  -4.419820   0.796103   0.069319
C   2.059709  -1.377706   0.574405
C   3.165534  -0.725589  -0.319631
```

```
O   1.216943  -2.077347  -0.053386
O   2.143434  -1.155854   1.791823
H   4.059531  -1.346333  -0.230485
C   3.515434   0.708448   0.059799
N   2.699111  -0.833960  -1.746865
H   3.447822  -1.016306  -2.414701
H   4.332155   1.087880  -0.555504
H   3.830091   0.718504   1.100408
S   2.129654   1.901205  -0.173615
H   2.006431  -1.605944  -1.734809
H   2.202789   0.020372  -2.024854
H   1.344077   1.424508   0.813588
28
Dimer 199 of the n...z  type
C   2.881409   1.010790  -0.258808
C   3.066335  -0.310317  -1.033105
N   3.692589   1.033999   0.945565
H   3.376820   0.310214   1.585916
H   3.583426   1.919691   1.429238
H   3.219336   1.810053  -0.927479
C   1.391166   1.252170  -0.017595
O   0.873839   1.270507   1.086438
O   0.709750   1.409842  -1.147837
H  -0.251418   1.519798  -0.945513
H   2.531060  -0.275500  -1.980114
H   4.129115  -0.439390  -1.236015
S   2.560799  -1.827922  -0.112228
H   1.218739  -1.705788  -0.252289
C  -2.005932  -1.529455  -0.238520
C  -2.486855  -0.637566   0.954497
O  -0.878451  -2.063973  -0.082776
O  -2.804560  -1.634185  -1.186863
H  -3.151196  -1.260945   1.558321
C  -3.255694   0.619630   0.566615
N  -1.295296  -0.343198   1.822343
H  -1.543512  -0.171764   2.796689
H  -3.394355   1.275326   1.427301
H  -4.239078   0.324749   0.208488
S  -2.495115   1.672750  -0.745571
H  -0.685215  -1.169965   1.748776
H  -0.719504   0.449546   1.483972
H  -2.653414   0.772093  -1.738060
28
Dimer 200 of the n...z  type
C   3.221901  -0.517867  -0.490810
C   1.793166   0.005887  -0.664576
N   3.418555  -1.551096   0.529659
H   2.795117  -1.409639   1.320611
H   3.252692  -2.479868   0.160603
H   3.525904  -0.938597  -1.456181
C   4.220474   0.628173  -0.239311
O   4.144367   1.714526  -0.767951
O   5.206801   0.305345   0.597404
H   4.986812  -0.614186   0.896832
H   1.753408   0.749027  -1.459039
H   1.117249  -0.811188  -0.917499
S   1.080525   0.748389   0.869840
H   1.727015   1.929762   0.804160
C  -1.827521  -1.529471  -0.175894
C  -2.552845  -0.193335   0.195016
O  -1.147338  -1.495523  -1.231504
O  -2.034165  -2.476821   0.605081
H  -2.406302   0.025580   1.251633
C  -4.042554  -0.297687  -0.128394
N  -1.916884   0.917909  -0.588544
H  -1.022289   1.190666  -0.149102
H  -4.464601  -1.114534   0.452925
H  -4.192333  -0.519071  -1.187121
S  -5.003822   1.246113   0.177410
```



```
H  -1.683985   0.543024  -1.517387
H  -2.528916   1.736571  -0.659591
H  -4.831743   1.278299   1.514925
28
Dimer 201 of the n...z  type
C  -2.026652  -0.397380  -0.001042
C  -2.100092   0.949170   0.728236
N  -1.984085  -0.345512  -1.463523
H  -2.517310   0.449174  -1.808212
H  -1.015393  -0.254372  -1.773380
H  -1.106312  -0.890101   0.335401
C  -3.155705  -1.349577   0.432232
O  -3.574549  -1.437634   1.566052
O  -3.615607  -2.104891  -0.564933
H  -3.111300  -1.773469  -1.359193
H  -2.097722   0.797402   1.806536
H  -1.228993   1.543640   0.451444
S  -3.562717   1.976063   0.272067
H  -4.494211   1.208707   0.874554
C   1.367401   0.529550  -0.688782
C   2.517760   0.007384   0.230166
O   0.947347  -0.305748  -1.535204
O   1.018036   1.706036  -0.503252
H   2.339046   0.299576   1.262992
C   3.856481   0.556956  -0.264704
N   2.505321  -1.495801   0.158350
H   1.884299  -1.903115   0.858086
H   3.828837   1.641958  -0.189776
H   4.016281   0.291478  -1.311797
S   5.332639  -0.097343   0.622558
H   2.115635  -1.726099  -0.771806
H   3.440924  -1.893884   0.292070
H   5.021839   0.400055   1.837344
28
Dimer 202 of the n...z  type
C   3.030114  -0.279249  -0.065024
C   2.928171   1.204514  -0.449163
N   2.658038  -1.240853  -1.106296
H   2.001490  -0.830490  -1.765058
H   3.464111  -1.570315  -1.623248
H   4.072048  -0.462993   0.218466
C   2.229853  -0.584353   1.216468
O   2.262653   0.109977   2.207094
O   1.524052  -1.713941   1.153835
H   1.692145  -2.057360   0.236402
H   3.153935   1.826730   0.415376
H   3.654859   1.421755  -1.232208
S   1.302426   1.716675  -1.153673
H   0.549523   1.621295  -0.025476
C  -2.117451   1.160786   0.622520
C  -2.753754  -0.263921   0.736293
O  -2.791007   2.001499  -0.021981
O  -1.023802   1.304077   1.204183
H  -3.132345  -0.368515   1.754760
C  -1.785203  -1.406651   0.446775
N  -3.943447  -0.286434  -0.185441
H  -4.753812  -0.775741   0.192373
H  -2.246195  -2.371864   0.660327
H  -0.909536  -1.288775   1.080272
S  -1.249189  -1.510206  -1.313741
H  -4.150306   0.715708  -0.361868
H  -3.682754  -0.707995  -1.085125
H  -0.532627  -0.363570  -1.336468
28
Dimer 203 of the n...z  type
C  -2.317742  -0.538974   0.206644
C  -1.717417   0.584026   1.062437
N  -1.557476  -0.944064  -0.976913
H  -1.151470  -0.124159  -1.423914
```

```
H  -0.785750  -1.560261  -0.718907
H  -2.405952  -1.416794   0.858695
C  -3.764591  -0.230249  -0.217301
O  -4.564640   0.364653   0.470427
O  -4.076766  -0.717118  -1.419806
H  -3.223435  -1.106916  -1.749454
H  -2.383428   0.824443   1.889736
H  -0.763507   0.246219   1.467630
S  -1.336829   2.129022   0.131494
H  -2.612272   2.512007  -0.084015
C   1.458781  -1.530739   0.795822
C   2.756200  -0.771538   0.369933
O   0.957742  -2.270189  -0.094088
O   1.038136  -1.275547   1.935720
H   3.518733  -0.857522   1.141680
C   2.416891   0.697235   0.107623
N   3.276464  -1.428173  -0.880276
H   3.926293  -2.185771  -0.668128
H   2.057275   1.134376   1.036412
H   1.623887   0.782892  -0.638137
S   3.806562   1.702906  -0.561348
H   2.449226  -1.850947  -1.335738
H   3.753881  -0.761344  -1.495749
H   4.619370   1.585638   0.508917
28
Dimer 204 of the n...z  type
C   2.321123   0.527656   0.410819
C   3.408612   0.731833  -0.648435
N   2.778629   0.243882   1.774128
H   3.661324  -0.261385   1.766866
H   2.900375   1.092302   2.314021
H   1.703780   1.432550   0.443574
C   1.316657  -0.555419  -0.003411
O   0.900344  -0.718721  -1.125288
O   0.876297  -1.292817   1.030598
H   1.403957  -0.952557   1.806742
H   2.956220   0.938497  -1.616943
H   4.029982   1.582359  -0.367657
S   4.577476  -0.685239  -0.818953
H   3.709954  -1.538307  -1.401046
C  -1.322878   1.430135  -0.054708
C  -2.190578   0.131599  -0.125539
O  -0.694676   1.609216   1.016725
O  -1.360122   2.138844  -1.079063
H  -1.839493  -0.476748  -0.958513
C  -3.671139   0.464866  -0.289841
N  -1.969343  -0.680498   1.122296
H  -1.022340  -1.085392   1.132503
H  -3.788994   1.059308  -1.192435
H  -4.033066   1.054888   0.555066
S  -4.783411  -1.005906  -0.359486
H  -2.025421  -0.067550   1.940083
H  -2.662680  -1.431548   1.198817
H  -4.309161  -1.507963  -1.518112
28
Dimer 205 of the n...z  type
C   3.072263   0.389528  -0.749473
C   2.646785   1.785331  -0.274183
N   2.220732  -0.319096  -1.695926
H   1.247170  -0.387518  -1.397262
H   2.262068   0.085087  -2.623426
H   4.046298   0.531778  -1.235891
C   3.382036  -0.551681   0.428621
O   3.777617  -0.178654   1.509853
O   3.209431  -1.842838   0.138951
H   2.836791  -1.850674  -0.777856
H   3.422159   2.206254   0.364424
H   2.520328   2.437211  -1.138827
S   1.046968   1.894686   0.631400
```



```
H   1.327481   1.003958   1.603887
C  -1.113569  -1.435284   0.097468
C  -2.090126  -0.220744   0.201140
O  -0.432279  -1.490282  -0.958213
O  -1.143675  -2.221059   1.060874
H  -1.952981   0.275403   1.161712
C  -3.538021  -0.679456   0.039915
N  -1.727252   0.770928  -0.865704
H  -0.849000   1.247613  -0.603600
H  -3.756216  -1.408241   0.817275
H  -3.684302  -1.160094  -0.929677
S  -4.781087   0.681923   0.092577
H  -1.537826   0.259672  -1.734132
H  -2.467407   1.465545  -1.006325
H  -4.567977   1.041931   1.374842
28
Dimer 206 of the n...z  type
C   2.544024   0.137036  -0.860092
C   2.781470   1.368776   0.021050
N   1.378294   0.172711  -1.748285
H   0.615467   0.769704  -1.413696
H   1.629404   0.489243  -2.676964
H   3.439971   0.017709  -1.480828
C   2.485287  -1.156702  -0.023658
O   3.190890  -1.380956   0.934311
O   1.587807  -2.030590  -0.482199
H   1.166546  -1.538037  -1.244580
H   3.749713   1.296905   0.513686
H   2.779292   2.261696  -0.604040
S   1.496324   1.664327   1.312369
H   1.898716   0.688004   2.150603
C  -2.012999   1.547689  -0.563407
C  -1.816425   0.229730   0.249310
O  -3.063819   2.185527  -0.318437
O  -1.108864   1.793946  -1.388369
H  -0.806399   0.202884   0.651392
C  -2.086423  -0.981835  -0.640778
N  -2.771035   0.259543   1.412276
H  -2.357320   0.721255   2.223054
H  -1.404847  -0.940518  -1.487964
H  -3.107182  -0.960097  -1.028361
S  -1.923900  -2.606427   0.215396
H  -3.573384   0.835410   1.109223
H  -3.061924  -0.680549   1.698153
H  -0.595351  -2.527363   0.441140
28
Dimer 207 of the n...z  type
C   2.690580  -0.902530   0.012860
C   3.343157   0.371639  -0.542191
N   1.890029  -1.682457  -0.930164
H   1.440115  -1.082176  -1.616637
H   2.452300  -2.363478  -1.425979
H   3.496912  -1.544148   0.385681
C   1.842143  -0.584517   1.255059
O   2.156967   0.210853   2.107712
O   0.702274  -1.286823   1.329031
H   0.641918  -1.831845   0.499975
H   3.958775   0.848115   0.218629
H   3.977116   0.109339  -1.389046
S   2.136631   1.606976  -1.193651
H   1.702994   2.044720   0.005789
C  -2.231990  -1.545333  -0.680019
C  -2.501659  -0.220368   0.106187
O  -1.551986  -2.412614  -0.082084
O  -2.719697  -1.570331  -1.827274
H  -3.576835  -0.044984   0.148212
C  -1.801826   0.959729  -0.564689
N  -2.032438  -0.368520   1.529239
H  -2.577131  -1.080819   2.015501
```

```
H  -2.174114   1.040527  -1.583111
H  -0.723436   0.801361  -0.614569
S  -2.003542   2.574560   0.303059
H  -1.046072  -0.676867   1.555109
H  -2.121162   0.519461   2.033605
H  -3.326820   2.712648   0.080477
28
Dimer 208 of the n...z  type
C  -2.986892   0.153228   0.604759
C  -2.852265  -1.328977   0.228658
N  -3.461860   1.046456  -0.457061
H  -3.159847   0.715969  -1.370591
H  -4.471833   1.123697  -0.463157
H  -3.693150   0.214114   1.440605
C  -1.672826   0.718841   1.168919
O  -0.943220   0.116947   1.924395
O  -1.414168   1.966722   0.769541
H  -2.161819   2.180628   0.147673
H  -2.461800  -1.896491   1.071741
H  -3.838527  -1.718491  -0.023322
S  -1.789130  -1.643718  -1.247213
H  -0.588650  -1.742293  -0.610571
C   2.245816  -1.598542   0.190131
C   1.989731  -0.065025   0.329125
O   3.439669  -1.961271   0.316282
O   1.236941  -2.288714  -0.066496
H   1.141766   0.117443   0.985818
C   1.744307   0.545793  -1.052460
N   3.213313   0.538698   0.964314
H   3.139202   0.559790   1.981559
H   0.833829   0.112799  -1.461931
H   2.568181   0.313632  -1.730321
S   1.613386   2.383849  -1.062579
H   3.994514  -0.094814   0.722469
H   3.378087   1.496947   0.637503
H   0.510782   2.457518  -0.286126
28
Dimer 209 of the n...z  type
C   3.305099   0.601799   0.303241
C   1.819454   0.417684   0.631423
N   3.617598   1.345033  -0.919455
H   2.925865   1.170366  -1.644132
H   3.654745   2.343610  -0.753223
H   3.752491   1.140678   1.146343
C   4.048505  -0.747796   0.272085
O   3.800689  -1.668450   1.017831
O   5.020773  -0.802539  -0.637721
H   4.959957   0.061759  -1.119780
H   1.699389  -0.115094   1.572923
H   1.337314   1.391028   0.716698
S   0.871734  -0.473177  -0.678681
H   1.365379  -1.703627  -0.430072
C  -4.026399  -1.066214  -0.302378
C  -3.137017  -0.017874   0.457352
O  -3.733815  -2.269804  -0.080433
O  -4.931116  -0.591473  -1.011043
H  -3.743405   0.391843   1.265997
C  -2.641690   1.102373  -0.446959
N  -2.005875  -0.779700   1.096580
H  -2.322328  -1.768908   1.052548
H  -3.505326   1.588142  -0.893465
H  -2.024116   0.705689  -1.254763
S  -1.583187   2.357467   0.393060
H  -1.124464  -0.702325   0.559933
H  -1.816865  -0.496532   2.056705
H  -2.517412   2.805694   1.256689
28
Dimer 210 of the n...z  type
C   3.035945   0.361236  -0.722133
```



```
C   1.541072   0.121649  -0.489059
N   3.688479  -0.481557  -1.727258
H   3.285343  -1.415096  -1.742149
H   3.607744  -0.088472  -2.657437
H   3.142655   1.404978  -1.039600
C   3.834304   0.276746   0.593494
O   3.413641   0.660378   1.661625
O   5.056190  -0.234472   0.444648
H   5.099816  -0.498178  -0.510460
H   1.146894   0.819687   0.247096
H   1.000736   0.263165  -1.425031
S   1.132223  -1.599300   0.045771
H   1.659727  -1.510646   1.284612
C  -1.590643   1.553880  -0.099835
C  -2.372872   0.199354  -0.158124
O  -1.067654   1.821736   1.010325
O  -1.600588   2.216929  -1.153410
H  -2.113452  -0.341048  -1.067366
C  -3.877156   0.463035  -0.105751
N  -1.938385  -0.636945   1.009435
H  -1.044471  -1.113378   0.797875
H  -4.150448   1.073100  -0.964017
H  -4.141232   1.011427   0.800880
S  -4.920508  -1.056922  -0.063558
H  -1.751708   0.011064   1.787588
H  -2.641091  -1.338427   1.262484
H  -4.575214  -1.521065  -1.282179
28
Dimer 211 of the n...z  type
C  -2.378823  -0.395081   0.861905
C  -3.854425   0.046465   0.844407
N  -1.511958   0.650979   1.359438
H  -1.555037   1.465150   0.753575
H  -0.543433   0.333418   1.404889
H  -2.324766  -1.249971   1.546982
C  -1.975957  -0.954150  -0.507108
O  -1.061289  -0.542912  -1.194326
O  -2.751946  -1.974521  -0.887016
H  -2.455505  -2.299179  -1.753299
H  -4.507254  -0.783270   0.581813
H  -4.115459   0.396753   1.842551
S  -4.218068   1.470732  -0.272012
H  -4.170139   0.790513  -1.435896
C   2.051537  -1.017358   0.635983
C   2.289159  -0.381565  -0.767938
O   1.286861  -0.387059   1.408462
O   2.675751  -2.075911   0.838496
H   2.011102  -1.107722  -1.533397
C   3.748172   0.019888  -0.978788
N   1.370240   0.795652  -0.939112
H   1.530613   1.291690  -1.817454
H   3.904922   0.418900  -1.982467
H   4.363170  -0.870225  -0.864487
S   4.354479   1.335721   0.162466
H   0.385622   0.468574  -0.919968
H   1.505885   1.447078  -0.161796
H   4.306736   0.602643   1.293714
28
Dimer 212 of the n...z  type
C  -2.891668   0.873172   0.338878
C  -2.992594  -0.450528   1.113655
N  -3.532725   0.898952  -0.979052
H  -3.606952  -0.040667  -1.362324
H  -4.458968   1.306868  -0.944143
H  -3.345326   1.648227   0.965821
C  -1.424485   1.314647   0.186835
O  -0.621527   1.295462   1.096272
O  -1.116228   1.751002  -1.030368
H  -1.963869   1.646710  -1.543347
```

```
H  -2.389074  -0.392835   2.018244
H  -4.030823  -0.619873   1.399937
S  -2.521317  -1.947140   0.147304
H  -1.202661  -1.678022  -0.047034
C   1.796232  -1.826393  -0.416269
C   2.027166  -0.460193   0.299018
O   2.804989  -2.566909  -0.507081
O   0.641101  -2.008906  -0.853560
H   1.224095  -0.252234   1.001534
C   2.116208   0.657951  -0.740580
N   3.316647  -0.562755   1.068978
H   3.168737  -0.933872   2.007685
H   1.169125   0.706087  -1.272669
H   2.912165   0.455599  -1.460124
S   2.494167   2.314233  -0.028753
H   3.898148  -1.245528   0.555893
H   3.780420   0.347872   1.155362
H   1.340084   2.407671   0.667557
28
Dimer 213 of the n...z  type
C   2.838900  -0.721407   0.196388
C   2.285595  -0.804289  -1.232810
N   1.834642  -0.993639   1.233146
H   1.003145  -1.450218   0.849620
H   2.214888  -1.597997   1.953451
H   3.671840  -1.431919   0.240396
C   3.459941   0.667814   0.437890
O   4.367753   1.116760  -0.226254
O   2.893990   1.343960   1.435638
H   2.186604   0.729920   1.769832
H   3.094975  -0.663712  -1.946557
H   1.843268  -1.786967  -1.394556
S   0.943003   0.390174  -1.656037
H   1.693111   1.511493  -1.637453
C  -1.879987  -1.814677   0.159711
C  -2.302395  -0.382123   0.617470
O  -0.646147  -2.055803   0.140720
O  -2.828343  -2.561311  -0.141453
H  -2.914049  -0.472687   1.515878
C  -3.082207   0.343358  -0.478044
N  -1.089301   0.419053   0.998966
H  -0.580140  -0.016687   1.768858
H  -3.944838  -0.262940  -0.742145
H  -2.468468   0.468599  -1.372430
S  -3.627483   2.046534  -0.025655
H  -0.422987   0.473895   0.203518
H  -1.354817   1.371167   1.269838
H  -4.505334   1.681929   0.931194
28
Dimer 214 of the n...z  type
C   3.006381   0.248329  -0.377674
C   1.926931  -0.396529  -1.252074
N   4.205047  -0.548239  -0.109225
H   3.986867  -1.540235  -0.060156
H   4.912226  -0.416213  -0.822440
H   3.318554   1.165612  -0.889909
C   2.426707   0.740800   0.964139
O   1.312998   1.205058   1.089426
O   3.273049   0.633127   1.982303
H   4.066701   0.176398   1.597909
H   1.113430   0.308024  -1.412808
H   2.350790  -0.661236  -2.220452
S   1.251247  -1.983058  -0.585739
H   0.930213  -1.529726   0.644478
C  -3.269209  -0.797005   0.871489
C  -2.107294  -0.076972   0.104991
O  -3.879889  -1.676962   0.210609
O  -3.465270  -0.401901   2.034082
H  -1.192672  -0.142344   0.692322
```



```
C  -2.455435   1.387906  -0.149591
N  -1.894102  -0.827674  -1.182052
H  -1.045415  -1.411881  -1.144094
H  -2.604000   1.872492   0.812758
H  -3.381839   1.474423  -0.721453
S  -1.190368   2.306811  -1.127575
H  -2.730826  -1.442009  -1.253726
H  -1.809571  -0.209865  -1.992566
H  -0.160716   2.094154  -0.276952
28
Dimer 215 of the n...z  type
C  -1.789440   0.387136   0.291721
C  -1.619434  -0.370380  -1.030443
N  -1.957046  -0.430093   1.495013
H  -2.463585  -1.284657   1.275968
H  -1.037419  -0.679130   1.864888
H  -0.883114   0.988373   0.431141
C  -2.930989   1.416620   0.223398
O  -3.186213   2.086887  -0.753613
O  -3.604973   1.528049   1.367899
H  -3.190231   0.832225   1.950082
H  -1.459029   0.327374  -1.850837
H  -0.755126  -1.029228  -0.946335
S  -3.032168  -1.478325  -1.453542
H  -3.926317  -0.504962  -1.722775
C   1.472231  -1.119545   1.006765
C   2.818691  -0.470335   0.552948
O   1.056599  -2.056769   0.284405
O   0.939513  -0.595795   2.006304
H   3.483350  -0.339037   1.404331
C   2.533832   0.871417  -0.123800
N   3.471829  -1.422536  -0.411952
H   4.079089  -2.089119   0.065712
H   2.060878   1.526630   0.604418
H   1.846648   0.741017  -0.962532
S   4.012447   1.697828  -0.847384
H   2.696263  -1.960386  -0.836256
H   4.030659  -0.933672  -1.118858
H   4.681238   1.872483   0.310950
28
Dimer 216 of the n...z  type
C  -1.624025  -0.869618  -0.271452
C  -1.973742   0.400379  -1.058590
N  -2.563675  -1.986435  -0.402784
H  -3.507733  -1.654075  -0.583740
H  -2.296989  -2.616157  -1.149881
H  -0.636220  -1.211561  -0.611033
C  -1.420581  -0.566675   1.220859
O  -0.824842   0.408019   1.634753
O  -1.915176  -1.494862   2.032628
H  -2.348308  -2.146009   1.417473
H  -1.232327   1.177722  -0.883828
H  -1.989819   0.171181  -2.124207
S  -3.642527   1.093715  -0.688283
H  -3.359228   1.530328   0.556050
C   2.464889  -1.415416  -0.582667
C   2.219017  -0.085701   0.201965
O   3.668134  -1.748810  -0.712772
O   1.434584  -1.968038  -1.014940
H   1.450178  -0.231072   0.956962
C   1.810703   1.021750  -0.765946
N   3.501862   0.272305   0.905053
H   3.534630  -0.108995   1.850902
H   0.959144   0.670675  -1.344061
H   2.617569   1.241355  -1.468127
S   1.403838   2.623484   0.047363
H   4.249082  -0.190610   0.356950
H   3.645554   1.283824   0.969666
H   0.407011   2.128337   0.813357
```

```
28
Dimer 217 of the n...z  type
C   2.670222  -0.910239  -0.125037
C   3.315801   0.402381  -0.592340
N   1.796346  -1.579111  -1.087225
H   1.300294  -0.902734  -1.662369
H   2.316132  -2.193995  -1.701688
H   3.483711  -1.599053   0.130307
C   1.907044  -0.703991   1.193589
O   2.286630   0.006345   2.093773
O   0.764465  -1.400996   1.277748
H   0.640990  -1.869953   0.410701
H   3.975266   0.799434   0.177330
H   3.903158   0.212207  -1.490527
S   2.100120   1.706638  -1.068785
H   1.721242   2.031529   0.184107
C  -2.263490  -1.533978  -0.655140
C  -2.487733  -0.214021   0.153359
O  -1.544846  -2.402569  -0.106209
O  -2.822630  -1.554119  -1.769276
H  -3.558717  -0.037984   0.254355
C  -1.826439   0.971635  -0.546869
N  -1.943195  -0.369953   1.549013
H  -2.487260  -1.053435   2.075792
H  -2.248397   1.053352  -1.545711
H  -0.750056   0.823257  -0.647772
S  -1.997458   2.581601   0.336450
H  -0.970534  -0.718749   1.525783
H  -1.968441   0.525525   2.047791
H  -3.333420   2.702233   0.193082
28
Dimer 218 of the n...z  type
C   1.438853  -0.250052  -0.233787
C   2.090539   1.046971  -0.739915
N   0.621920  -0.008060   0.943947
H   1.200606   0.268633   1.732202
H   0.111917  -0.846462   1.208993
H   0.772065  -0.604027  -1.026913
C   2.476667  -1.355613  -0.038431
O   2.678094  -1.949703   0.994584
O   3.152094  -1.606030  -1.177832
H   3.794247  -2.314870  -1.012610
H   2.614186   0.874126  -1.677798
H   1.306398   1.784844  -0.909475
S   3.248027   1.850291   0.453028
H   4.266193   0.976157   0.315367
C  -2.070375  -1.395533  -0.088752
C  -3.176022  -0.362738  -0.483677
O  -2.108537  -1.801344   1.103243
O  -1.280157  -1.708769  -0.996211
H  -3.855313  -0.861723  -1.176321
C  -2.625090   0.902062  -1.147897
N  -3.952410  -0.046373   0.765242
H  -4.963213  -0.037525   0.638521
H  -3.428570   1.475682  -1.611126
H  -1.927223   0.591122  -1.922515
S  -1.799951   2.073167   0.013813
H  -3.658156  -0.780731   1.439174
H  -3.643930   0.861666   1.139983
H  -0.784385   1.235007   0.433208
28
Dimer 219 of the n...z  type
C  -2.864078   0.726569   0.552249
C  -2.333378  -0.440356   1.398835
N  -4.042454   0.443889  -0.272013
H  -4.077028  -0.540569  -0.526646
H  -4.904020   0.681562   0.204385
H  -3.113826   1.538639   1.244014
C  -1.759289   1.317258  -0.343016
```



```
O  -0.625158   1.514689   0.036012
O  -2.168637   1.629480  -1.569335
H  -3.121839   1.346200  -1.585072
H  -1.418615  -0.141855   1.908967
H  -3.077139  -0.702584   2.151400
S  -2.035908  -1.997129   0.458366
H  -0.947031  -1.604834  -0.256014
C   1.796158  -1.640437  -0.729305
C   1.938647  -0.399029   0.205334
O   2.771322  -2.428822  -0.727861
O   0.744290  -1.683355  -1.401311
H   1.005776  -0.191354   0.722681
C   2.357498   0.816667  -0.622658
N   2.989628  -0.722487   1.232913
H   2.593574  -1.206184   2.039470
H   1.563823   1.025416  -1.335131
H   3.281236   0.614811  -1.168969
S   2.690346   2.330585   0.372192
H   3.646167  -1.373961   0.773299
H   3.464303   0.122511   1.568706
H   1.415515   2.507125   0.775008
28
Dimer 220 of the n...z  type
C  -2.825878  -0.227087  -0.904628
C  -3.407163  -0.863702   0.371667
N  -1.790162  -1.058438  -1.483586
H  -1.021631  -1.229938  -0.838328
H  -1.395502  -0.624372  -2.311224
H  -3.658494  -0.159002  -1.614252
C  -2.407657   1.218325  -0.615619
O  -1.267550   1.633023  -0.620361
O  -3.464720   1.994433  -0.338990
H  -3.157755   2.894242  -0.140398
H  -4.293031  -0.325663   0.702362
H  -3.686317  -1.891620   0.142427
S  -2.229681  -0.982787   1.791715
H  -2.368107   0.285272   2.230410
C   1.868961  -1.141745  -0.765770
C   1.743085   0.392099  -0.479069
O   1.013283  -1.855922  -0.178719
O   2.764410  -1.479487  -1.559320
H   1.135603   0.813383  -1.283112
C   3.066090   1.142102  -0.412502
N   0.936794   0.553167   0.777911
H   0.247591   1.303953   0.697253
H   2.903914   2.210857  -0.265193
H   3.590736   0.995746  -1.353397
S   4.152810   0.614275   0.980192
H   0.437608  -0.347374   0.903811
H   1.542200   0.699583   1.590859
H   4.338677  -0.648307   0.542629
28
Dimer 221 of the n...z  type
C   2.799161   0.507619   0.479263
C   1.301290   0.539548   0.160647
N   3.677068   1.248846  -0.429545
H   3.330444   1.215662  -1.385067
H   3.762015   2.221460  -0.159193
H   2.919305   0.933058   1.482273
C   3.319980  -0.937004   0.608323
O   2.677368  -1.842295   1.090848
O   4.566142  -1.098365   0.164000
H   4.813464  -0.212830  -0.207758
H   0.743961  -0.004458   0.921177
H   0.939396   1.566590   0.138439
S   0.878941  -0.147692  -1.500110
H   1.038270  -1.450653  -1.190104
C  -1.895749   1.759332   0.665426
C  -2.726874   0.589035   0.036458
```

```
O  -1.353464   2.536062  -0.156504
O  -1.887588   1.792609   1.911101
H  -3.783515   0.849212   0.123427
C  -2.461440  -0.757899   0.698057
N  -2.424226   0.524890  -1.435641
H  -2.489709   1.465752  -1.830873
H  -2.662257  -0.663076   1.761714
H  -1.417136  -1.051015   0.577918
S  -3.432871  -2.161305  -0.003563
H  -1.446347   0.215935  -1.583039
H  -3.050527  -0.117125  -1.926481
H  -4.642255  -1.748576   0.428029
28
Dimer 222 of the n...z  type
C   1.726713  -0.198494   0.425420
C   3.054865  -0.780101   0.923366
N   1.053028  -0.937089  -0.643707
H   1.710943  -1.486674  -1.188718
H   0.313594  -1.539364  -0.274361
H   1.055102  -0.161844   1.291138
C   1.883865   1.274560  -0.006323
O   2.525985   2.097412   0.607134
O   1.206503   1.565745  -1.117835
H   0.839965   0.676178  -1.394888
H   3.458407  -0.162960   1.724307
H   2.885762  -1.785526   1.309100
S   4.337050  -0.977023  -0.388075
H   4.580290   0.336953  -0.571617
C  -2.422759  -1.802114   0.208750
C  -2.393997  -0.341334  -0.346844
O  -3.557536  -2.323218   0.294384
O  -1.308813  -2.263623   0.538162
H  -1.582303  -0.229380  -1.061354
C  -2.242174   0.646554   0.809927
N  -3.688792  -0.100164  -1.073384
H  -3.639140  -0.404521  -2.046556
H  -1.335702   0.400314   1.358253
H  -3.082366   0.559124   1.502133
S  -2.207728   2.419580   0.309838
H  -4.394940  -0.689526  -0.603579
H  -3.955512   0.889330  -1.064771
H  -1.041080   2.372793  -0.368202
28
Dimer 223 of the n...z  type
C   2.006353  -1.034198   0.287636
C   3.272192  -1.346655  -0.532843
N   0.821655  -0.704464  -0.513407
H   1.058378   0.006237  -1.205457
H   0.549908  -1.525007  -1.050774
H   1.769374  -1.934292   0.865122
C   2.325016   0.029203   1.349609
O   3.295555  -0.076921   2.063583
O   1.507644   1.078565   1.480552
H   0.765080   1.086873   0.842488
H   4.085210  -1.646835   0.125197
H   3.058605  -2.167624  -1.217914
S   3.835372   0.044782  -1.602539
H   4.348755   0.808697  -0.616682
C  -3.936923  -0.868948  -0.150532
C  -2.819273   0.108601   0.369061
O  -3.689354  -2.092673   0.007645
O  -4.935007  -0.324141  -0.652835
H  -3.242860   0.701699   1.179234
C  -2.327781   1.018981  -0.749179
N  -1.716910  -0.741895   0.939322
H  -1.551172  -0.564123   1.928215
H  -3.186526   1.540431  -1.164472
H  -1.864318   0.439528  -1.550141
S  -1.049928   2.254107  -0.256061
```



```
H  -2.083631  -1.710091   0.818299
H  -0.806443  -0.649392   0.428887
H  -1.748896   2.835651   0.740129
28
Dimer 224 of the n...z  type
C  -2.652261  -0.485714  -0.997420
C  -3.160270  -1.128684   0.296331
N  -1.573844  -1.190029  -1.699260
H  -0.996786  -1.727472  -1.059016
H  -1.935838  -1.829758  -2.396481
H  -3.510083  -0.425263  -1.676962
C  -2.233381   0.980291  -0.780104
O  -2.793956   1.740545  -0.025612
O  -1.193291   1.352461  -1.532131
H  -0.924114   0.522971  -2.007118
H  -4.034616  -0.595216   0.664400
H  -3.443069  -2.162253   0.098654
S  -1.933488  -1.208645   1.675425
H  -1.972303   0.103021   1.987354
C   2.881621  -1.224443  -0.533327
C   1.858435  -0.164349   0.001383
O   3.253260  -2.080417   0.310822
O   3.217867  -1.086295  -1.722373
H   0.978514  -0.169439  -0.637644
C   2.479980   1.229921   0.020382
N   1.439627  -0.604067   1.377064
H   0.478762  -0.972488   1.388173
H   2.762229   1.489498  -0.997353
H   3.380619   1.243390   0.637656
S   1.388600   2.538192   0.726801
H   2.107320  -1.367903   1.609343
H   1.480667   0.152751   2.063649
H   0.370648   2.371583  -0.144016
28
Dimer 225 of the n...z  type
C  -2.083132  -0.762451   0.301763
C  -1.773697   0.333537   1.331853
N  -1.216363  -0.837534  -0.872310
H  -0.951688   0.095739  -1.177820
H  -0.368277  -1.381904  -0.692538
H  -2.011645  -1.720480   0.830653
C  -3.550291  -0.692552  -0.167265
O  -4.478412  -0.384842   0.547512
O  -3.710849  -1.039770  -1.443846
H  -2.781819  -1.191561  -1.767572
H  -2.517527   0.320057   2.126760
H  -0.796036   0.151964   1.778666
S  -1.670484   2.037448   0.635756
H  -2.969462   2.156058   0.292247
C   2.306905  -1.820335   0.147105
C   2.234543  -0.269391   0.332530
O   3.393559  -2.350639   0.470423
O   1.279872  -2.342394  -0.335923
H   1.302473   0.003336   0.819497
C   2.358685   0.427334  -1.022233
N   3.362583   0.136397   1.241265
H   3.087245   0.102412   2.223396
H   1.561353   0.070380  -1.669998
H   3.309750   0.176950  -1.496566
S   2.322371   2.267209  -0.943977
H   4.103597  -0.571399   1.104089
H   3.698307   1.083777   1.040954
H   1.048551   2.382431  -0.510748
28
Dimer 226 of the n...z  type
C  -2.772788  -0.959652   0.213897
C  -3.462286   0.412681   0.253500
N  -2.010998  -1.326813   1.404172
H  -1.598011  -0.509800   1.846043
```

```
H  -2.590299  -1.795165   2.090170
H  -3.558367  -1.709815   0.068152
C  -1.885460  -1.089604  -1.037794
O  -2.227113  -0.691953  -2.131226
O  -0.715362  -1.692169  -0.846816
H  -0.617498  -1.882755   0.118356
H  -4.033172   0.581799  -0.657690
H  -4.142854   0.447323   1.104364
S  -2.298739   1.823939   0.502237
H  -1.771165   1.816728  -0.738402
C   2.230248  -1.515121   0.431736
C   2.520087   0.022571   0.507573
O   2.889484  -2.138633  -0.435712
O   1.351387  -1.929349   1.210883
H   2.670517   0.330777   1.540229
C   1.358905   0.792927  -0.119856
N   3.797546   0.272640  -0.248617
H   4.614576   0.227136   0.360759
H   0.465700   0.610785   0.472085
H   1.165717   0.442568  -1.135149
S   1.623376   2.607708  -0.279237
H   3.868061  -0.512112  -0.921857
H   3.798721   1.185037  -0.715779
H   1.708645   2.874367   1.040469
28
Dimer 227 of the n...z  type
C   2.533773  -0.376474  -0.793419
C   3.029889   0.808358   0.044538
N   1.547432  -0.094808  -1.837905
H   0.871696   0.635503  -1.592887
H   1.990316   0.163746  -2.711123
H   3.417424  -0.806593  -1.280410
C   1.992947  -1.518751   0.087919
O   2.450637  -1.828634   1.163945
O   0.965417  -2.163941  -0.473148
H   0.821843  -1.666038  -1.328011
H   3.880173   0.508390   0.654955
H   3.354092   1.604474  -0.625604
S   1.769764   1.575997   1.154200
H   1.826166   0.635012   2.117999
C  -1.513360   1.772147  -0.592146
C  -1.570612   0.405518   0.162900
O  -2.251670   2.672778  -0.128622
O  -0.763936   1.795284  -1.589696
H  -0.563659   0.028169   0.317276
C  -2.411482  -0.600108  -0.620538
N  -2.169758   0.661204   1.518058
H  -1.454757   0.921801   2.198521
H  -1.960676  -0.731769  -1.602140
H  -3.428861  -0.230198  -0.762038
S  -2.596129  -2.235298   0.211026
H  -2.793977   1.476534   1.399920
H  -2.670241  -0.157071   1.879669
H  -1.281927  -2.545948   0.213831
28
Dimer 228 of the n...z  type
C   2.921888   0.627662   0.434379
C   1.870198  -0.290452   1.068219
N   2.366918   1.574534  -0.543203
H   1.347389   1.635272  -0.510311
H   2.731837   2.508063  -0.390504
H   3.417820   1.146085   1.262152
C   4.012189  -0.217529  -0.253311
O   4.710072  -1.015386   0.334515
O   4.106125   0.001346  -1.561776
H   3.404457   0.693461  -1.729703
H   2.336369  -0.926626   1.816999
H   1.102463   0.314334   1.550073
S   0.988855  -1.389054  -0.140212
```



```
H   1.695178  -2.511595   0.091978
C  -1.229022   1.131211   0.428345
C  -2.231252   0.046941  -0.078184
O  -1.114086   1.206173   1.674019
O  -0.658679   1.800664  -0.458931
H  -1.888746  -0.370068  -1.023069
C  -3.630039   0.643580  -0.219104
N  -2.231146  -1.065135   0.931973
H  -1.371024  -1.620135   0.831310
H  -3.593813   1.446724  -0.952937
H  -3.964815   1.063705   0.731286
S  -4.932506  -0.571650  -0.694759
H  -2.206620  -0.633134   1.865064
H  -3.049763  -1.673595   0.830071
H  -4.443932  -0.857878  -1.918942
28
Dimer 229 of the n...z  type
C  -1.607171  -0.131744   0.352101
C  -2.385283   1.194232   0.419389
N  -0.715390  -0.169207  -0.791748
H  -1.244041  -0.177369  -1.658856
H  -0.126274  -0.999648  -0.756605
H  -0.997112  -0.173820   1.262167
C  -2.557782  -1.330238   0.432210
O  -2.654392  -2.199450  -0.400963
O  -3.290477  -1.303497   1.562903
H  -3.879023  -2.075372   1.574887
H  -2.968837   1.253572   1.335728
H  -1.668103   2.014592   0.408887
S  -3.494890   1.511200  -1.020100
H  -4.458153   0.627171  -0.688017
C   2.531429  -1.273375  -0.426037
C   3.282857  -0.306637   0.548429
O   3.007144  -1.342362  -1.586589
O   1.561116  -1.884011   0.063719
H   3.860099  -0.927723   1.236263
C   2.370170   0.616117   1.355582
N   4.266335   0.471837  -0.282243
H   5.168784   0.620419   0.167353
H   2.934024   1.128951   2.135411
H   1.607473   0.001090   1.828309
S   1.571668   1.949300   0.362077
H   4.360040  -0.082249  -1.155223
H   3.857013   1.380824  -0.536092
H   0.676600   1.147583  -0.310511
28
Dimer 230 of the n...z  type
C   2.497439  -0.609668  -0.346599
C   2.090977   0.730665  -0.973638
N   1.499837  -1.327210   0.446771
H   1.002291  -0.688110   1.062234
H   0.806694  -1.778696  -0.146200
H   2.776270  -1.262405  -1.183219
C   3.791792  -0.490168   0.480554
O   4.686033   0.287628   0.236200
O   3.861083  -1.363920   1.487237
H   2.985780  -1.828354   1.475440
H   2.941516   1.161391  -1.499552
H   1.276348   0.570809  -1.680910
S   1.422870   1.987044   0.198332
H   2.430585   1.980738   1.094455
C  -1.318803  -0.644458  -1.294079
C  -2.106602  -0.169597  -0.030077
O  -1.111079   0.238105  -2.159484
O  -0.996433  -1.850237  -1.297044
H  -1.657900  -0.585393   0.870397
C  -3.572578  -0.588532  -0.134199
N  -1.988612   1.326762   0.049820
H  -1.085391   1.602679   0.453465
```

```
H  -3.617464  -1.674489  -0.184946
H  -4.024932  -0.185860  -1.042540
S  -4.638178   0.018189   1.241646
H  -2.000909   1.680017  -0.917377
H  -2.743922   1.739287   0.607573
H  -4.032796  -0.674260   2.228354
28
Dimer 231 of the n...z  type
C   2.921623  -0.633613  -0.427157
C   1.865680   0.277788  -1.063527
N   2.373215  -1.571801   0.562035
H   1.354468  -1.645323   0.528023
H   2.749818  -2.503131   0.424561
H   3.413829  -1.158251  -1.253288
C   4.014027   0.219619   0.247157
O   4.711804   1.008543  -0.352700
O   4.109473   0.019265   1.558515
H   3.407871  -0.670028   1.737277
H   2.328101   0.908050  -1.819608
H   1.096794  -0.331679  -1.537396
S   0.988589   1.386063   0.139048
H   1.691888   2.507469  -0.107580
C  -1.229627  -1.129410  -0.429466
C  -2.232642  -0.046276   0.077862
O  -1.121269  -1.208958  -1.675441
O  -0.651845  -1.793097   0.457257
H  -1.890283   0.370766   1.022782
C  -3.631099  -0.643567   0.218543
N  -2.233010   1.066147  -0.931936
H  -1.371569   1.619371  -0.832421
H  -3.594668  -1.446984   0.952100
H  -3.965597  -1.063483  -0.732027
S  -4.934059   0.570955   0.694681
H  -2.211335   0.634814  -1.865347
H  -3.050419   1.675882  -0.828137
H  -4.445900   0.856543   1.919174
28
Dimer 232 of the n...z  type
C   2.125599   0.785292   0.254334
C   1.746366  -0.191250   1.377489
N   1.246753   0.840141  -0.911713
H   0.938998  -0.095758  -1.164949
H   0.419603   1.419902  -0.745010
H   2.143271   1.786009   0.702885
C   3.572436   0.557746  -0.225898
O   4.481427   0.207873   0.493533
O   3.744193   0.820359  -1.521871
H   2.829973   1.034708  -1.847976
H   2.514069  -0.188527   2.149474
H   0.807198   0.122909   1.833096
S   1.454732  -1.926605   0.827442
H   2.714945  -2.187464   0.423535
C  -2.237230   1.827582   0.151535
C  -2.159064   0.272043   0.293717
O  -3.301441   2.349756   0.553761
O  -1.240951   2.361823  -0.379655
H  -1.196741  -0.017801   0.706808
C  -2.378223  -0.393132  -1.064762
N  -3.225529  -0.150856   1.266564
H  -2.883685  -0.150122   2.228312
H  -1.622235  -0.027161  -1.756099
H  -3.357616  -0.128367  -1.468162
S  -2.349894  -2.234809  -1.020012
H  -3.964444   0.569894   1.200324
H  -3.585596  -1.088043   1.060040
H  -1.068837  -2.360711  -0.609745
28
Dimer 233 of the n...z  type
C   3.258892   0.682883  -0.216794
```



```
C   1.760609   0.677283   0.106726
N   3.624307   0.617222  -1.633358
H   2.964133   0.048348  -2.157669
H   3.654594   1.536839  -2.057366
H   3.666952   1.614550   0.191823
C   4.005902  -0.429492   0.544727
O   3.725462  -0.779260   1.669050
O   5.019818  -0.958126  -0.139606
H   4.979073  -0.516678  -1.026470
H   1.601501   0.753793   1.180476
H   1.280410   1.526361  -0.378393
S   0.865625  -0.809649  -0.523314
H   1.404837  -1.699257   0.335849
C  -3.955981  -1.066014  -0.324255
C  -3.139049   0.022142   0.459692
O  -3.698767  -2.254873  -0.002548
O  -4.780700  -0.631797  -1.147550
H  -3.812440   0.459785   1.198129
C  -2.571772   1.108598  -0.443080
N  -2.060876  -0.699818   1.223056
H  -2.390144  -1.683880   1.249325
H  -3.394039   1.573261  -0.980744
H  -1.887954   0.682933  -1.179977
S  -1.591561   2.398532   0.438763
H  -1.155807  -0.688465   0.722574
H  -1.910306  -0.332354   2.161126
H  -2.607953   2.900863   1.169362
28
Dimer 234 of the n...z  type
C   2.019480  -1.096327   0.100278
C   3.305451  -1.210044  -0.740767
N   0.835065  -0.658167  -0.647958
H   1.064249   0.159602  -1.213013
H   0.581181  -1.385177  -1.313677
H   1.806164  -2.095220   0.494991
C   2.294382  -0.243585   1.348246
O   3.238018  -0.482771   2.066549
O   1.472862   0.770065   1.636216
H   0.756741   0.902438   0.980976
H   4.120339  -1.602155  -0.135261
H   3.128351  -1.894560  -1.570812
S   3.833008   0.374286  -1.521708
H   4.302273   0.959589  -0.400607
C  -3.967565  -0.787472  -0.239920
C  -2.797609   0.060708   0.380358
O  -3.749872  -2.025092  -0.308469
O  -4.970272  -0.141224  -0.589375
H  -3.170182   0.537184   1.286247
C  -2.311745   1.113892  -0.607484
N  -1.711064  -0.903608   0.770747
H  -1.535273  -0.911785   1.773883
H  -3.166265   1.712060  -0.914232
H  -1.888816   0.648332  -1.500010
S  -0.987848   2.235586   0.017239
H  -2.106975  -1.824984   0.485222
H  -0.800538  -0.745509   0.276268
H  -1.662079   2.723801   1.078368
28
Dimer 235 of the n...z  type
C   2.236892   0.048867  -0.789432
C   3.688041  -0.451454  -0.884343
N   2.048773   1.492278  -0.841617
H   2.707981   1.958911  -0.223877
H   2.207667   1.842234  -1.779629
H   1.701381  -0.381588  -1.646237
C   1.523963  -0.547344   0.435498
O   1.889765  -1.577365   0.965102
O   0.447154   0.092446   0.878590
H   0.188854   0.899246   0.354259
```

Printed by sara


```
H   3.723529  -1.539564  -0.894023
H   4.128366  -0.080368  -1.810372
S   4.792979   0.168316   0.455612
H   4.253655  -0.570738   1.445678
C  -2.355029   1.729870   0.034791
C  -2.633317   0.238390   0.411917
O  -3.368851   2.440314  -0.145512
O  -1.147358   2.038173  -0.079694
H  -2.010822  -0.058623   1.252428
C  -2.363075  -0.663842  -0.792108
N  -4.075654   0.143629   0.831833
H  -4.187528   0.277987   1.837097
H  -1.327792  -0.533355  -1.094802
H  -2.999578  -0.387631  -1.635365
S  -2.700657  -2.449669  -0.493348
H  -4.555342   0.928587   0.358026
H  -4.492481  -0.759908   0.587778
H  -1.777402  -2.620358   0.475494
28
Dimer 236 of the n...z  type
C   1.460260  -0.374127  -0.177774
C   1.935032   0.908709  -0.881790
N   0.618306  -0.068495   0.968154
H   1.176784   0.273572   1.744822
H   0.117127  -0.897117   1.277231
H   0.843427  -0.917859  -0.901288
C   2.638918  -1.285031   0.161071
O   2.933429  -1.662291   1.271204
O   3.331636  -1.629267  -0.942933
H   4.071731  -2.201152  -0.683450
H   2.491294   0.671086  -1.786418
H   1.058447   1.495284  -1.155998
S   2.950385   2.039415   0.166272
H   4.092165   1.321578   0.134448
C  -1.994218  -1.414283  -0.344318
C  -3.168729  -0.389462  -0.468260
O  -1.901848  -2.000964   0.766919
O  -1.291474  -1.546635  -1.361582
H  -3.897386  -0.815461  -1.159401
C  -2.734811   0.985774  -0.983950
N  -3.824178  -0.299553   0.882425
H  -4.842483  -0.316419   0.854473
H  -3.600604   1.573472  -1.290838
H  -2.096626   0.826637  -1.850634
S  -1.863552   2.027207   0.263699
H  -3.442434  -1.112395   1.405036
H  -3.507432   0.557269   1.358694
H  -0.804887   1.175656   0.520997
28
Dimer 237 of the n...z  type
C   3.213369   0.609593  -0.432549
C   1.700380   0.622350  -0.188408
N   3.660282   0.188974  -1.762327
H   3.047426  -0.524953  -2.148413
H   3.688920   0.965546  -2.412313
H   3.568661   1.633055  -0.267824
C   3.950491  -0.227444   0.630975
O   3.619117  -0.278627   1.794063
O   5.015619  -0.873875   0.158598
H   5.009405  -0.685528  -0.814976
H   1.477568   0.980171   0.814593
H   1.222863   1.285617  -0.909166
S   0.890250  -1.018666  -0.431313
H   1.426948  -1.623684   0.648704
C  -3.987529  -1.008328  -0.249598
C  -3.117201   0.083915   0.468749
O  -3.770525  -2.189166   0.126161
O  -4.808984  -0.582593  -1.080684
H  -3.759762   0.574665   1.201447
```



```
C  -2.533965   1.110698  -0.491075
N  -2.047991  -0.640076   1.242041
H  -1.855505  -0.226324   2.152846
H  -3.351824   1.580982  -1.030590
H  -1.883490   0.630577  -1.224648
S  -1.491670   2.402615   0.314552
H  -2.415173  -1.606108   1.333883
H  -1.155981  -0.697011   0.720152
H  -2.478453   2.976925   1.032808
28
Dimer 238 of the n...z  type
C   2.801166   0.453980   0.838878
C   2.058132  -0.765350   1.397924
N   1.988068   1.599043   0.432044
H   1.065630   1.350294   0.068680
H   1.854062   2.253584   1.192747
H   3.488166   0.790608   1.624641
C   3.724064   0.061949  -0.331808
O   4.361444  -0.967763  -0.375252
O   3.779629   0.984696  -1.290163
H   3.130785   1.678258  -0.985994
H   2.767740  -1.505271   1.764498
H   1.424934  -0.451184   2.227879
S   0.921713  -1.583162   0.199892
H   1.873274  -2.141108  -0.574680
C  -1.935344   1.846958  -0.199004
C  -2.277135   0.403004   0.295632
O  -2.889262   2.661874  -0.167626
O  -0.767965   2.008581  -0.603812
H  -1.507433   0.042442   0.974183
C  -2.416211  -0.530592  -0.906614
N  -3.573539   0.485500   1.056645
H  -3.420310   0.652805   2.051303
H  -1.460321  -0.560666  -1.423938
H  -3.171743  -0.155381  -1.599870
S  -2.952830  -2.243836  -0.491441
H  -4.060273   1.315350   0.673074
H  -4.133729  -0.367471   0.961601
H  -1.853409  -2.580906   0.214617
28
Dimer 239 of the n...z  type
C   2.801162  -0.581352  -0.378361
C   1.320945  -0.298513  -0.100376
N   3.506216  -1.390707   0.620324
H   3.131401  -1.223477   1.551165
H   3.429917  -2.381277   0.422461
H   2.851520  -1.109272  -1.337608
C   3.593417   0.719157  -0.613226
O   3.156089   1.675342  -1.213668
O   4.830742   0.688550  -0.117695
H   4.888375  -0.189024   0.342997
H   0.895245   0.296240  -0.906731
H   0.770386  -1.236432  -0.033468
S   0.999975   0.549944   1.504904
H   1.525875   1.745185   1.166630
C  -1.857047  -1.701600  -0.613541
C  -2.953499  -0.617319  -0.341974
O  -1.401917  -2.264976   0.414993
O  -1.530882  -1.844611  -1.804501
H  -3.796265  -0.752800  -1.016657
C  -2.345090   0.776328  -0.505282
N  -3.441311  -0.819917   1.067647
H  -4.243941  -1.448396   1.106614
H  -2.015237   0.886259  -1.535958
H  -1.478100   0.893646   0.148721
S  -3.464481   2.167076  -0.050516
H  -2.656327  -1.286544   1.557507
H  -3.706997   0.063055   1.515458
H  -4.409675   1.930232  -0.983612
```

```
28
Dimer 240 of the n...z  type
C  -2.292152  -0.333964  -0.635297
C  -2.011379   1.169253  -0.753300
N  -3.637271  -0.775766  -1.011169
H  -4.328553  -0.062058  -0.792708
H  -3.701159  -0.986940  -1.999835
H  -1.573324  -0.851546  -1.281059
C  -1.973316  -0.860305   0.776458
O  -1.034596  -0.477366   1.440365
O  -2.805828  -1.810697   1.195221
H  -3.480507  -1.882980   0.470405
H  -0.984557   1.387586  -0.466669
H  -2.155709   1.482096  -1.787448
S  -3.146758   2.230765   0.238425
H  -2.662601   1.887309   1.449469
C   3.340532   1.345035  -0.114029
C   3.080984  -0.192174  -0.271583
O   3.492461   1.737529   1.070898
O   3.327394   1.997628  -1.172504
H   3.707649  -0.604397  -1.059685
C   1.601429  -0.426982  -0.568496
N   3.468023  -0.843412   1.029897
H   4.438042  -1.158955   1.024247
H   1.358479   0.068657  -1.505994
H   0.976735  -0.004993   0.220541
S   1.095020  -2.196597  -0.647732
H   3.389629  -0.087257   1.735761
H   2.875042  -1.647708   1.256375
H   1.841343  -2.533189  -1.719936
28
Dimer 241 of the n...z  type
C   3.164620  -0.019483   0.601442
C   1.739103   0.223955   1.105803
N   4.090742   1.114188   0.671898
H   3.606709   1.994691   0.515013
H   4.553823   1.165581   1.571391
H   3.581372  -0.830357   1.209978
C   3.170212  -0.577451  -0.835436
O   2.338152  -1.345586  -1.262593
O   4.201971  -0.157136  -1.567601
H   4.676026   0.487133  -0.981108
H   1.142723  -0.685989   1.057217
H   1.773433   0.564759   2.140957
S   0.841990   1.558557   0.194441
H   0.726951   0.896102  -0.975514
C  -1.690169  -1.842598   0.402464
C  -2.636439  -0.593653   0.461951
O  -0.849450  -1.919444   1.331973
O  -1.903791  -2.629057  -0.539044
H  -3.588876  -0.928622   0.878018
C  -2.856573   0.064654  -0.892901
N  -2.065598   0.384076   1.452428
H  -1.715093  -0.152591   2.252573
H  -3.240260  -0.684726  -1.579931
H  -1.916364   0.441036  -1.299754
S  -3.986216   1.523880  -0.860676
H  -1.244898   0.877466   1.055204
H  -2.749867   1.081610   1.749786
H  -5.113644   0.844310  -0.566168
28
Dimer 242 of the n...z  type
C  -2.414700   0.175370   1.054844
C  -1.833617   1.535860   0.650162
N  -1.512993  -0.971486   1.103794
H  -0.846430  -1.007697   0.330861
H  -0.997617  -1.012199   1.974444
H  -2.828571   0.311131   2.061832
C  -3.639383  -0.199897   0.197613
```



```
O  -4.424845   0.604384  -0.252582
O  -3.778162  -1.513825   0.022585
H  -2.973702  -1.902523   0.458263
H  -2.602593   2.302940   0.728953
H  -1.015792   1.795372   1.322763
S  -1.102745   1.623471  -1.038071
H  -2.208929   1.279878  -1.727892
C   1.762086  -1.954488  -0.326821
C   2.609507  -0.653643  -0.492064
O   0.606542  -1.911581  -0.818692
O   2.320541  -2.868217   0.307137
H   3.616238  -0.904963  -0.823197
C   2.657609   0.108923   0.832624
N   1.991617   0.203667  -1.561898
H   2.139552  -0.196743  -2.488670
H   3.130555  -0.528711   1.576188
H   1.650594   0.345595   1.181359
S   3.529236   1.731776   0.757205
H   0.970341   0.260488  -1.417023
H   2.390851   1.148903  -1.546378
H   4.759652   1.240461   0.503342
28
Dimer 243 of the n...z  type
C  -2.525350  -0.811411   0.705502
C  -1.649704  -1.337643  -0.437875
N  -3.968002  -0.764928   0.447855
H  -4.154728  -0.605752  -0.539334
H  -4.428876  -1.623596   0.724207
H  -2.353370  -1.463963   1.568919
C  -2.067723   0.580986   1.177852
O  -0.903369   0.908317   1.280344
O  -3.067389   1.396911   1.492402
H  -3.885169   0.872020   1.278545
H  -0.601780  -1.386170  -0.142944
H  -1.972267  -2.347089  -0.695413
S  -1.807541  -0.383534  -2.009795
H  -1.242875   0.761705  -1.573557
C   2.560935  -1.544225   0.289016
C   2.152137  -0.043382   0.439238
O   3.782898  -1.787103   0.458351
O   1.631654  -2.315696  -0.013397
H   1.269413   0.048480   1.067408
C   1.880653   0.558958  -0.936679
N   3.295519   0.668984   1.113148
H   3.185078   0.689469   2.127056
H   1.099387  -0.026653  -1.416333
H   2.770746   0.511814  -1.567527
S   1.399611   2.338945  -0.900363
H   4.130473   0.092516   0.902079
H   3.400497   1.634325   0.787544
H   0.361690   2.188793  -0.049070
28
Dimer 244 of the n...z  type
C  -2.550737  -0.413277   0.677004
C  -1.319324   0.471678   0.893489
N  -2.342501  -1.457129  -0.332787
H  -1.354408  -1.692667  -0.460651
H  -2.822961  -2.311146  -0.069139
H  -2.804023  -0.825064   1.660429
C  -3.752323   0.458916   0.258468
O  -4.239837   1.308632   0.970287
O  -4.202740   0.197550  -0.967048
H  -3.602277  -0.526639  -1.293401
H  -1.560270   1.241370   1.623166
H  -0.497281  -0.134757   1.271040
S  -0.787314   1.337479  -0.667055
H  -0.372480   2.476506  -0.080485
C   1.347490  -1.749245   0.190302
C   2.489675  -0.783914  -0.267195
```

```
O   0.520246  -2.056106  -0.707274
O   1.382306  -2.108540   1.379835
H   3.379650  -1.383821  -0.467449
C   2.802805   0.298539   0.759765
N   2.083445  -0.166813  -1.576845
H   1.825842  -0.903493  -2.236445
H   3.064656  -0.183643   1.697568
H   1.928569   0.927047   0.937690
S   4.139361   1.465773   0.255426
H   1.233120   0.408600  -1.434814
H   2.825509   0.419454  -1.966998
H   5.151666   0.575344   0.295579
28
Dimer 245 of the n...z  type
C  -3.141368  -0.527163   0.766163
C  -3.942454   0.773437   0.527281
N  -1.938249  -0.404511   1.569875
H  -1.297395   0.306690   1.217668
H  -2.180057  -0.155030   2.522713
H  -3.825103  -1.202086   1.294244
C  -2.905221  -1.226143  -0.582062
O  -3.851279  -1.479834  -1.306673
O  -1.677469  -1.548489  -0.956769
H  -0.910277  -1.279819  -0.367215
H  -4.889675   0.554674   0.036113
H  -4.150252   1.239327   1.491371
S  -3.052657   2.063441  -0.444901
H  -3.147658   1.455956  -1.645411
C   1.231562  -0.055135   0.461190
C   2.780564  -0.109229   0.599955
O   0.709080  -1.098434  -0.026085
O   0.686743   1.004707   0.800805
H   3.085733   0.291065   1.564713
C   3.422286   0.683565  -0.541330
N   3.203049  -1.552789   0.542490
H   3.152263  -1.998061   1.459909
H   3.097612   1.718890  -0.461895
H   3.094813   0.297285  -1.508788
S   5.261239   0.603161  -0.590843
H   2.526973  -2.034924  -0.068869
H   4.162326  -1.652020   0.192159
H   5.480975   1.233919   0.581230
28
Dimer 246 of the n...z  type
C   3.282601   0.501967   0.473944
C   3.328614  -0.956256  -0.022627
N   3.033288   0.565139   1.902340
H   2.113787   0.185883   2.112199
H   3.032180   1.528613   2.221257
H   4.273109   0.925351   0.271684
C   2.299370   1.319695  -0.367021
O   1.318095   1.882392   0.074672
O   2.641489   1.342893  -1.660899
H   1.998742   1.871607  -2.161032
H   3.608595  -0.997870  -1.073191
H   4.079792  -1.488007   0.560491
S   1.766648  -1.908780   0.220568
H   1.084609  -1.433609  -0.843512
C  -1.677588  -1.217619  -0.485772
C  -1.544491   0.146906   0.270070
O  -1.239614  -1.206114  -1.668037
O  -2.154992  -2.161676   0.165706
H  -0.634498   0.078665   0.870808
C  -2.714327   0.488835   1.181870
N  -1.292684   1.202894  -0.768765
H  -0.512234   1.820928  -0.517660
H  -2.528501   1.415131   1.727569
H  -2.835746  -0.319601   1.898824
S  -4.305630   0.757627   0.290025
```



```
H  -1.044474   0.653120  -1.616039
H  -2.139354   1.741871  -0.968534
H  -4.421423  -0.500986  -0.181690
28
Dimer 247 of the n...z  type
C  -2.596564   0.243201   0.816377
C  -2.855895   1.175015  -0.372884
N  -1.440333   0.575252   1.655627
H  -0.725900   1.106520   1.155769
H  -1.713130   1.115634   2.467749
H  -3.495333   0.265599   1.443417
C  -2.481138  -1.225663   0.362129
O  -3.183098  -1.726297  -0.488527
O  -1.545836  -1.909939   1.019021
H  -1.132055  -1.226666   1.613210
H  -3.782097   0.899323  -0.874050
H  -2.949154   2.199619  -0.012750
S  -1.496675   1.211436  -1.619921
H  -1.767822   0.012710  -2.174775
C   2.007157   1.730680   0.202055
C   2.544325   0.261260   0.256247
O   1.133735   2.008677   1.065797
O   2.473416   2.436024  -0.707294
H   3.626703   0.247799   0.146750
C   1.879036  -0.563390  -0.847517
N   2.209728  -0.301752   1.612205
H   2.939018  -0.097943   2.296349
H   2.191802  -0.160373  -1.808398
H   0.792907  -0.480581  -0.789744
S   2.224207  -2.371290  -0.782540
H   1.351856   0.182515   1.922866
H   2.077404  -1.319085   1.576870
H   3.552249  -2.296238  -1.009137
28
Dimer 248 of the n...z  type
C  -2.099400  -0.047470   0.597019
C  -3.575825  -0.127253   1.004002
N  -1.478774   1.279136   0.647347
H  -2.152370   2.004463   0.411448
H  -1.108075   1.484316   1.567825
H  -1.539509  -0.706918   1.269978
C  -1.866805  -0.655266  -0.798051
O  -2.419975  -1.654173  -1.197274
O  -0.956544  -0.003827  -1.526655
H  -0.712843   0.783682  -0.976438
H  -3.928732  -1.154992   0.938215
H  -3.685183   0.212862   2.033871
S  -4.691456   0.958398   0.015630
H  -4.618204   0.256449  -1.133783
C   3.480268   1.404993  -0.159210
C   2.920352  -0.044018  -0.366975
O   4.637552   1.460483   0.329320
O   2.701438   2.323536  -0.467999
H   2.500915  -0.144624  -1.366111
C   1.864225  -0.341693   0.694406
N   4.083864  -0.992151  -0.242803
H   4.499332  -1.209672  -1.148819
H   1.064357   0.388120   0.591092
H   2.283674  -0.239188   1.697426
S   1.179136  -2.051845   0.638612
H   4.788795  -0.465544   0.308020
H   3.824637  -1.875969   0.205387
H   0.708023  -1.990641  -0.624083
28
Dimer 249 of the n...z  type
C  -1.699584   0.184812  -0.305784
C  -2.913578   1.073438  -0.592480
N  -1.448376  -0.902814  -1.257325
H  -2.320880  -1.271880  -1.628306
```

```
H  -0.880282  -0.595117  -2.037473
H  -0.814952   0.836435  -0.285938
C  -1.744236  -0.400936   1.113407
O  -2.161175   0.194285   2.081612
O  -1.222875  -1.628823   1.199734
H  -1.030911  -1.877025   0.257717
H  -2.997417   1.852539   0.163376
H  -2.792600   1.547351  -1.566851
S  -4.516675   0.164003  -0.695942
H  -4.610662  -0.137575   0.615225
C   2.118593   1.818277   0.168257
C   2.454910   0.435296  -0.484217
O   3.114105   2.514196   0.484708
O   0.904592   2.045954   0.328638
H   1.838792   0.275646  -1.366245
C   2.242476  -0.681603   0.535864
N   3.894112   0.484034  -0.923112
H   3.987055   0.822886  -1.881060
H   1.208528  -0.654678   0.871266
H   2.881799  -0.531658   1.408219
S   2.651976  -2.368071  -0.080722
H   4.341190   1.182232  -0.302219
H   4.351950  -0.430697  -0.865749
H   1.724777  -2.402513  -1.060091
28
Dimer 250 of the n...z  type
C  -2.864851   0.737489   0.127441
C  -3.280875  -0.730846  -0.016894
N  -2.496141   1.433526  -1.109013
H  -2.068943   0.795264  -1.775366
H  -3.300162   1.860157  -1.553799
H  -3.713238   1.270619   0.571977
C  -1.727020   0.907527   1.151340
O  -1.626253   0.256941   2.166339
O  -0.862287   1.872069   0.830736
H  -1.162521   2.186366  -0.061770
H  -3.568206  -1.139567   0.950226
H  -4.136315  -0.797094  -0.689080
S  -1.989451  -1.821820  -0.755274
H  -1.186074  -1.912378   0.324520
C   2.260426  -1.754685   0.341580
C   2.377219  -0.428907  -0.480598
O   1.157904  -2.348824   0.254981
O   3.267131  -2.041867   1.016048
H   3.326955  -0.400493  -1.011838
C   2.254094   0.768660   0.462396
N   1.282055  -0.399536  -1.513125
H   1.542908  -0.922553  -2.349636
H   3.077834   0.729262   1.171574
H   1.316181   0.731988   1.016917
S   2.225887   2.409685  -0.375607
H   0.447040  -0.862473  -1.120252
H   1.061159   0.563107  -1.793464
H   3.473081   2.352050  -0.887367
28
Dimer 251 of the n...z  type
C   1.956274  -0.737392   0.251716
C   3.437091  -0.626150   0.653788
N   1.791869  -1.323154  -1.064812
H   2.164175  -0.692667  -1.769610
H   0.796001  -1.441774  -1.259588
H   1.475553  -1.395544   0.981303
C   1.258482   0.614351   0.416168
O   0.784996   1.270862  -0.484929
O   1.235345   1.016547   1.701286
H   0.791657   1.878417   1.751157
H   3.536117  -0.276838   1.680135
H   3.889259  -1.614721   0.575508
S   4.467076   0.447232  -0.441335
```



```
H   3.975287   1.634076  -0.029922
C  -1.657661  -1.357065  -0.180488
C  -3.127626  -0.845454  -0.017807
O  -1.043212  -1.573774   0.894672
O  -1.267654  -1.515031  -1.352527
H  -3.787780  -1.678663  -0.264952
C  -3.476778   0.346056  -0.906250
N  -3.320814  -0.539533   1.442178
H  -4.218008  -0.843899   1.817440
H  -4.545800   0.559270  -0.870864
H  -3.203133   0.094219  -1.928283
S  -2.658951   1.920932  -0.408925
H  -2.527214  -1.025913   1.906249
H  -3.207588   0.468491   1.607720
H  -1.380404   1.528822  -0.604491
28
Dimer 252 of the n...z  type
C  -2.874304  -1.276319   0.281708
C  -3.684394  -0.445963  -0.723476
N  -3.065213  -0.950764   1.697407
H  -3.303394   0.031093   1.816212
H  -3.797140  -1.509885   2.118686
H  -3.151555  -2.325362   0.130965
C  -1.369337  -1.221656  -0.041342
O  -0.918669  -1.261389  -1.163580
O  -0.591615  -1.138673   1.040793
H  -1.234573  -1.102196   1.799058
H  -3.450056  -0.757196  -1.740005
H  -4.748487  -0.603638  -0.546977
S  -3.439109   1.375846  -0.574872
H  -2.157056   1.411186  -0.996495
C   3.527548  -0.923125  -0.353074
C   2.545870   0.053750   0.377525
O   4.719621  -0.524544  -0.417921
O   3.012412  -1.956337  -0.814981
H   1.911014  -0.488676   1.074503
C   1.686090   0.782516  -0.653901
N   3.392594   1.027051   1.155039
H   3.551910   0.713561   2.112578
H   1.066586   0.045390  -1.159717
H   2.309728   1.288330  -1.393726
S   0.610509   2.105512   0.049228
H   4.307374   1.026160   0.665864
H   2.973729   1.961776   1.191699
H  -0.074969   1.306047   0.891402
28
Dimer 253 of the n...z  type
C  -2.961979   0.449065  -0.493238
C  -3.172515  -1.021420  -0.877308
N  -2.026668   1.204783  -1.330816
H  -1.310086   0.610999  -1.738154
H  -2.500494   1.684179  -2.086722
H  -3.939866   0.939017  -0.548434
C  -2.532093   0.573563   0.981084
O  -3.032264  -0.057082   1.882741
O  -1.537841   1.446078   1.180374
H  -1.345259   1.822820   0.280127
H  -3.988927  -1.454450  -0.303097
H  -3.421755  -1.083089  -1.936097
S  -1.688486  -2.105827  -0.642637
H  -1.920439  -2.399176   0.652876
C   3.199561  -1.272692   0.054864
C   2.098729  -0.184448  -0.197953
O   3.086255  -1.902076   1.137764
O   4.070236  -1.364574  -0.828702
H   1.686814  -0.302869  -1.198938
C   2.698040   1.208983  -0.026701
N   0.997963  -0.432643   0.793951
H   0.247000  -1.001289   0.378438
```

```
H   3.531568   1.309432  -0.718407
H   3.079788   1.342286   0.987877
S   1.511732   2.597400  -0.280036
H   1.444302  -0.988719   1.547286
H   0.564239   0.422755   1.154421
H   1.232582   2.330893  -1.572239
28
Dimer 254 of the n...z  type
C   2.856779   0.020552  -0.949028
C   1.753920   1.068272  -0.736385
N   2.383011  -1.363250  -0.811874
H   1.359982  -1.420042  -0.812673
H   2.726040  -1.938797  -1.572916
H   3.281185   0.209544  -1.941128
C   4.000375   0.253561   0.057390
O   4.654257   1.273732   0.109103
O   4.187174  -0.770834   0.884157
H   3.494420  -1.430340   0.581750
H   2.146427   2.063576  -0.935835
H   0.934111   0.864937  -1.424264
S   0.980974   1.067105   0.940635
H   1.962416   1.744327   1.571143
C  -1.378871  -1.466165  -0.164835
C  -2.756810  -0.747939  -0.338398
O  -1.252217  -2.148943   0.877689
O  -0.551967  -1.241880  -1.072852
H  -3.158637  -0.944429  -1.330490
C  -2.591338   0.754920  -0.113242
N  -3.698427  -1.339085   0.676297
H  -4.229253  -2.121058   0.292477
H  -1.900888   1.137041  -0.861443
H  -2.163227   0.954115   0.870816
S  -4.162430   1.715950  -0.155008
H  -3.089081  -1.716011   1.424302
H  -4.366605  -0.649220   1.033790
H  -4.487983   1.437997  -1.434290
28
Dimer 255 of the n...z  type
C   2.785252  -0.194407   1.037099
C   2.104713  -1.454121   0.486701
N   1.973103   1.022474   1.090166
H   1.317604   1.075258   0.312704
H   1.442948   1.085505   1.951051
H   3.108965  -0.426819   2.058407
C   4.092656   0.116175   0.282611
O   4.856900  -0.732259  -0.119987
O   4.327507   1.420214   0.137446
H   3.524032   1.857783   0.523730
H   2.789935  -2.299184   0.525769
H   1.230895  -1.687287   1.095755
S   1.462237  -1.282607  -1.232932
H   2.660933  -1.214368  -1.846858
C  -3.695961  -1.168029   0.297159
C  -3.212260   0.145059  -0.408789
O  -4.487350  -0.990558   1.258042
O  -3.211097  -2.221732  -0.150424
H  -3.279199   0.041879  -1.489715
C  -1.777602   0.449796   0.017996
N  -4.141987   1.248756   0.020443
H  -4.922076   1.363153  -0.627167
H  -1.144152  -0.369527  -0.313208
H  -1.704226   0.519668   1.105202
S  -1.100569   2.044244  -0.608293
H  -4.531714   0.926916   0.926403
H  -3.665242   2.151796   0.103033
H  -1.101679   1.718268  -1.917048
28
Dimer 256 of the n...z  type
C  -2.966565   0.534590  -0.283596
```



```
C  -1.874929   1.403315   0.356816
N  -3.977457  -0.013894   0.624323
H  -3.596204  -0.134010   1.559827
H  -4.793205   0.583149   0.686814
H  -3.474216   1.157744  -1.028140
C  -2.352588  -0.614503  -1.107134
O  -1.390570  -0.478993  -1.830536
O  -3.001198  -1.770516  -0.982836
H  -3.721037  -1.575738  -0.325882
H  -1.148785   1.696025  -0.400211
H  -2.325771   2.308251   0.764591
S  -1.011196   0.625849   1.787672
H  -0.436899  -0.430389   1.146587
C   1.982197  -1.710106   0.316140
C   2.324670  -0.185985   0.366875
O   2.923368  -2.464248  -0.025828
O   0.797902  -1.991400   0.592441
H   1.952478   0.246026   1.293138
C   1.719394   0.524395  -0.839817
N   3.825204  -0.054596   0.361577
H   4.209784  -0.061043   1.306833
H   0.667182   0.261590  -0.917144
H   2.204149   0.198065  -1.762644
S   1.908320   2.356614  -0.802412
H   4.173310  -0.903272  -0.117147
H   4.145464   0.800169  -0.101407
H   1.226453   2.544484   0.347684
28
Dimer 257 of the n...z  type
C   2.101884  -0.870171   0.519284
C   1.873370   0.565566   1.007589
N   3.428489  -1.438896   0.777576
H   4.143382  -0.715450   0.754104
H   3.471977  -1.897181   1.679960
H   1.361156  -1.504980   1.019442
C   1.785223  -1.017994  -0.981193
O   0.857764  -0.464820  -1.530281
O   2.609445  -1.842788  -1.624656
H   3.272270  -2.112707  -0.936010
H   0.853949   0.892915   0.811839
H   2.046684   0.606938   2.083348
S   3.036783   1.799787   0.283151
H   2.501476   1.809189  -0.954630
C  -1.829058   1.809432   0.019026
C  -2.728222   0.556108   0.295225
O  -1.857069   2.235881  -1.162518
O  -1.163905   2.208328   0.992181
H  -3.349917   0.722603   1.172893
C  -1.840956  -0.674096   0.484791
N  -3.632887   0.386853  -0.896097
H  -4.531306   0.849943  -0.760051
H  -1.234292  -0.515838   1.373745
H  -1.169110  -0.796063  -0.366625
S  -2.743195  -2.272963   0.623653
H  -3.144973   0.875300  -1.670028
H  -3.813779  -0.599041  -1.109949
H  -3.417463  -1.986381   1.756602
28
Dimer 258 of the n...z  type
C  -1.982788  -0.559247   0.805100
C  -3.506033  -0.351381   0.696956
N  -1.324515   0.567453   1.438639
H  -1.391377   1.390736   0.847107
H  -0.338950   0.336571   1.546015
H  -1.837666  -1.441546   1.434699
C  -1.418747  -0.919030  -0.570535
O  -0.804126  -0.167814  -1.295296
O  -1.726754  -2.180625  -0.921021
H  -1.378356  -2.352921  -1.810710
```

```
H  -3.987395  -1.237201   0.284807
H  -3.897542  -0.176560   1.699162
S  -4.034817   1.126942  -0.272028
H  -3.700915   0.663978  -1.493996
C   2.045091  -1.180180   0.478827
C   3.140579  -0.066815   0.601970
O   2.256305  -2.037214  -0.414265
O   1.112057  -1.112705   1.301350
H   3.765784  -0.332292   1.456920
C   2.600720   1.345872   0.805072
N   4.002558  -0.169103  -0.626388
H   4.996493  -0.024578  -0.453147
H   3.409511   2.042807   1.028506
H   1.919318   1.329276   1.652552
S   1.753402   2.050363  -0.671541
H   3.814324  -1.123813  -0.989750
H   3.681774   0.504060  -1.333606
H   0.728554   1.167694  -0.727688
28
Dimer 259 of the n...z  type
C  -1.843643  -0.594305  -0.389188
C  -1.812693   0.839203  -0.931855
N  -3.003846  -1.407320  -0.764011
H  -3.835645  -0.831836  -0.872504
H  -2.847256  -1.906644  -1.631382
H  -0.940419  -1.100820  -0.751514
C  -1.689905  -0.619294   1.139352
O  -0.955300   0.124073   1.754032
O  -2.409725  -1.567755   1.737313
H  -2.925374  -1.983918   0.997790
H  -0.915645   1.353261  -0.591233
H  -1.806059   0.813932  -2.021742
S  -3.283719   1.857276  -0.480250
H  -2.993490   1.979182   0.831306
C   2.023000  -1.772156  -0.304872
C   2.696330  -0.364179  -0.435772
O   1.060602  -1.973137  -1.093192
O   2.487025  -2.509178   0.580314
H   3.779165  -0.463921  -0.475396
C   2.278090   0.512364   0.747055
N   2.232091   0.226860  -1.740695
H   2.892475   0.047938  -2.497166
H   2.685559   0.070439   1.653844
H   1.191732   0.543657   0.850541
S   2.794977   2.275544   0.622974
H   1.355897  -0.283564  -1.960692
H   2.086696   1.239710  -1.679870
H   4.126626   2.058867   0.621324
28
Dimer 260 of the n...z  type
C   3.178042   0.418412   0.665843
C   1.906177  -0.420712   0.837035
N   3.058712   1.616141  -0.170159
H   2.398805   1.462630  -0.929095
H   2.741825   2.416314   0.364184
H   3.490135   0.737651   1.667051
C   4.353406  -0.432968   0.148692
O   4.552433  -1.579618   0.481818
O   5.162456   0.222169  -0.684342
H   4.732424   1.108775  -0.799837
H   2.097606  -1.273754   1.485435
H   1.129318   0.196896   1.287847
S   1.181684  -1.021513  -0.748720
H   2.116455  -1.957529  -1.010074
C  -4.031369  -1.167540   0.043139
C  -3.210126   0.078311  -0.436275
O  -5.165773  -0.902680   0.516681
O  -3.450772  -2.261391  -0.066357
H  -2.784926  -0.103726  -1.421297
```



```
C  -2.107830   0.384111   0.576532
N  -4.172079   1.231578  -0.542068
H  -4.561455   1.318369  -1.480882
H  -1.436047  -0.470946   0.611214
H  -2.528345   0.529391   1.573614
S  -1.154374   1.924833   0.236754
H  -4.949637   0.985137   0.098754
H  -3.735710   2.125525  -0.295655
H  -0.670396   1.552600  -0.966406
28
Dimer 261 of the n...z  type
C  -3.101773   0.272863  -0.818881
C  -1.649494   0.114964  -0.354155
N  -3.787504   1.497725  -0.398003
H  -3.456950   1.804436   0.513966
H  -3.644778   2.253608  -1.057086
H  -3.093041   0.250798  -1.914655
C  -3.959663  -0.942231  -0.416064
O  -3.558073  -2.084204  -0.431825
O  -5.208732  -0.625592  -0.074720
H  -5.231635   0.365620  -0.118275
H  -1.229812  -0.814573  -0.735158
H  -1.056475   0.947935  -0.731324
S  -1.430485   0.167224   1.476060
H  -1.985196  -1.034603   1.734154
C   3.606405  -1.495437   0.013165
C   3.352246   0.020753   0.316769
O   4.406615  -1.718293  -0.931032
O   2.959804  -2.294122   0.713010
H   3.407620   0.209328   1.386934
C   1.985306   0.425451  -0.231768
N   4.450853   0.798256  -0.358345
H   5.243542   0.956627   0.263953
H   1.226152  -0.157193   0.285943
H   1.917303   0.208208  -1.299653
S   1.591364   2.218852  -0.084014
H   4.774193   0.181500  -1.127189
H   4.131250   1.709184  -0.701606
H   1.585238   2.269563   1.263811
28
Dimer 262 of the n...z  type
C   3.697139  -0.429164  -0.534492
C   2.471098  -0.718404  -1.412593
N   4.150335  -1.531630   0.319232
H   3.372842  -2.137804   0.570350
H   4.859927  -2.094214  -0.134428
H   4.518368  -0.155867  -1.206295
C   3.482440   0.824002   0.336987
O   2.974583   1.845055  -0.069003
O   3.944924   0.697217   1.580996
H   4.289115  -0.234147   1.617880
H   2.189335   0.175365  -1.966891
H   2.719028  -1.505884  -2.124442
S   1.004907  -1.343657  -0.486262
H   0.640706  -0.177112   0.101082
C  -1.507997   1.439225   0.091165
C  -2.897486   0.779286   0.375306
O  -1.406535   2.021364  -1.015029
O  -0.648219   1.263380   0.977290
H  -3.251554   1.053254   1.367016
C  -2.773156  -0.740235   0.249937
N  -3.867355   1.325213  -0.638110
H  -4.365925   2.142155  -0.284947
H  -2.081657  -1.087862   1.014086
H  -2.366191  -1.015499  -0.725175
S  -4.364391  -1.658571   0.379013
H  -3.279562   1.637527  -1.432611
H  -4.563720   0.629022  -0.922845
H  -4.660079  -1.292759   1.643451
```

```
28
Dimer 263 of the n...z  type
C   2.700788  -0.620794   0.403260
C   3.293981   0.505832  -0.456681
N   2.006771  -1.694400  -0.295605
H   1.397551  -1.357923  -1.040027
H   2.651285  -2.371265  -0.684900
H   3.538299  -1.069628   0.951536
C   1.784400  -0.059282   1.503921
O   2.002856   0.975770   2.094605
O   0.721692  -0.815919   1.781521
H   0.735490  -1.574525   1.145571
H   3.854383   1.203184   0.164073
H   3.973749   0.071478  -1.189966
S   2.055694   1.452475  -1.441437
H   1.535813   2.146058  -0.408740
C  -1.377081  -1.618246  -0.773384
C  -2.393895  -0.454335  -0.526042
O  -1.281166  -2.447849   0.166537
O  -0.744676  -1.555741  -1.843345
H  -3.019961  -0.301084  -1.402881
C  -1.625118   0.819132  -0.175203
N  -3.276974  -0.869986   0.619817
H  -4.114910  -1.355090   0.298547
H  -0.981128   1.071504  -1.014334
H  -0.991721   0.650654   0.697215
S  -2.679256   2.260228   0.274066
H  -2.710829  -1.549518   1.160244
H  -3.571103  -0.072122   1.191890
H  -3.316600   2.371812  -0.909534
28
Dimer 264 of the n...z  type
C   2.086738  -0.003759  -0.587709
C   3.543690   0.001730  -1.065898
N   1.428948   1.302095  -0.485930
H   2.089834   2.019048  -0.194476
H   1.030944   1.589848  -1.372435
H   1.513246  -0.613957  -1.295223
C   1.934501  -0.744548   0.752685
O   2.542580  -1.750088   1.038984
O   1.028256  -0.201023   1.570291
H   0.731557   0.621586   1.104603
H   3.925151  -1.016180  -1.125253
H   3.594604   0.450968  -2.057753
S   4.677335   1.010140  -0.017789
H   4.685291   0.181659   1.046516
C  -3.497678   1.409239   0.175296
C  -2.945769  -0.044339   0.371800
O  -4.635277   1.475863  -0.356130
O  -2.731369   2.320721   0.532871
H  -2.549694  -0.164933   1.378110
C  -1.864819  -0.323727  -0.670148
N  -4.106812  -0.987804   0.202767
H  -4.552686  -1.209193   1.093280
H  -1.062506   0.396366  -0.525581
H  -2.257207  -0.192022  -1.680730
S  -1.191571  -2.039238  -0.641397
H  -4.792396  -0.457929  -0.368182
H  -3.833315  -1.869461  -0.241478
H  -0.723497  -2.001625   0.623444
28
Dimer 265 of the n...z  type
C   2.012628  -0.703561   0.433185
C   3.530603  -0.702132   0.695167
N   1.655034  -1.537092  -0.699795
H   1.972335  -1.103967  -1.562375
H   0.639617  -1.592541  -0.734743
H   1.542247  -1.123194   1.326052
C   1.508006   0.735456   0.320332
```



```
O   1.192317   1.285165  -0.710131
O   1.485767   1.353790   1.517830
H   1.194240   2.271086   1.390441
H   3.765804  -0.138393   1.596962
H   3.853495  -1.733767   0.835168
S   4.573070  -0.073510  -0.692184
H   4.260396   1.232735  -0.568321
C  -2.236197  -1.330914   0.424022
C  -2.755496  -0.571819  -0.843653
O  -3.119407  -1.939232   1.077317
O  -1.008954  -1.264346   0.626828
H  -2.561472  -1.214179  -1.705632
C  -2.107695   0.785205  -1.078786
N  -4.250936  -0.467569  -0.703946
H  -4.757443  -0.547098  -1.585447
H  -2.493939   1.252369  -1.985741
H  -1.035243   0.647524  -1.186655
S  -2.421425   1.999186   0.272732
H  -4.502882  -1.233369  -0.050942
H  -4.503189   0.418237  -0.251616
H  -1.737841   1.329942   1.223912
28
Dimer 266 of the n...z  type
C   2.989102   0.500240   0.289860
C   3.036356  -1.001285   0.602816
N   2.408836   1.361394   1.324429
H   1.692550   0.863751   1.848037
H   3.109593   1.688482   1.978721
H   4.021353   0.826325   0.118139
C   2.275779   0.777644  -1.047147
O   2.396376   0.086544  -2.032901
O   1.530013   1.884389  -1.034599
H   1.601175   2.216850  -0.101147
H   3.455447  -1.545112  -0.242178
H   3.673895  -1.165579   1.471703
S   1.407708  -1.740276   1.051495
H   0.842012  -1.703447  -0.176503
C  -1.781884  -1.604634  -0.631516
C  -2.500517  -0.222957  -0.479739
O  -2.084599  -2.461541   0.235024
O  -0.952351  -1.675323  -1.558392
H  -2.914654   0.098656  -1.433161
C  -1.501413   0.805502   0.049511
N  -3.637766  -0.417259   0.487494
H  -4.514129  -0.624556   0.008301
H  -0.720323   0.938995  -0.693824
H  -1.031846   0.447725   0.967942
S  -2.222472   2.441647   0.487736
H  -3.375436  -1.254541   1.040633
H  -3.787159   0.406261   1.079276
H  -2.633802   2.775708  -0.752867
28
Dimer 267 of the n...z  type
C  -2.374196  -0.508027  -0.781072
C  -1.467473  -1.219793   0.230821
N  -1.869157   0.735836  -1.367114
H  -1.304904   1.262099  -0.705079
H  -1.325954   0.573464  -2.205969
H  -2.550882  -1.214986  -1.599755
C  -3.776106  -0.244940  -0.194117
O  -4.349579  -1.009776   0.548428
O  -4.323482   0.898947  -0.605649
H  -3.619604   1.324404  -1.159993
H  -1.960783  -2.116578   0.602084
H  -0.525483  -1.509196  -0.238724
S  -0.968294  -0.180190   1.669312
H  -2.192896  -0.024164   2.212118
C   2.754013  -1.492879  -0.420563
C   2.497103  -0.117904   0.282892
```

```
O   3.956924  -1.743845  -0.679169
O   1.728307  -2.152370  -0.671220
H   1.853896  -0.256695   1.148830
C   1.864568   0.850482  -0.713166
N   3.824583   0.399999   0.768517
H   4.012448   0.116255   1.730724
H   0.957533   0.389391  -1.096795
H   2.530894   1.024240  -1.560828
S   1.487606   2.522314  -0.038423
H   4.529236  -0.063320   0.166056
H   3.891133   1.420629   0.723114
H   0.623564   2.104353   0.912270
28
Dimer 268 of the n...z  type
C  -2.913739   0.623747   0.190477
C  -2.960786  -0.047570  -1.189186
N  -2.339408   1.972075   0.234120
H  -1.644514   2.094973  -0.498568
H  -3.047055   2.688162   0.123353
H  -3.945668   0.682044   0.554318
C  -2.194685  -0.261768   1.227790
O  -2.338854  -1.460926   1.302279
O  -1.419243   0.423342   2.069340
H  -1.491660   1.363288   1.756432
H  -3.353353  -1.058960  -1.099322
H  -3.620586   0.523721  -1.842380
S  -1.343133  -0.101823  -2.072587
H  -0.740953  -1.063996  -1.333394
C   1.524262  -1.782662   0.384579
C   2.472028  -0.555045   0.176224
O   0.976248  -2.206792  -0.668480
O   1.388227  -2.163503   1.557788
H   3.348047  -0.634094   0.816009
C   1.698017   0.732101   0.457814
N   2.931508  -0.576509  -1.257581
H   3.770903  -1.143755  -1.380039
H   1.330255   0.702261   1.481124
H   0.837828   0.807821  -0.209795
S   2.650320   2.281736   0.167556
H   2.167474  -1.029898  -1.786491
H   3.128990   0.365066  -1.612085
H   3.598984   2.066005   1.101965
28
Dimer 269 of the n...z  type
C   1.550614  -0.272718  -0.230986
C   2.748669  -1.109626  -0.693247
N   1.171103   0.851440  -1.094836
H   2.001002   1.322215  -1.451631
H   0.646087   0.530164  -1.900616
H   0.687488  -0.949545  -0.155967
C   1.739086   0.235824   1.204429
O   2.275518  -0.402227   2.080680
O   1.211209   1.444932   1.432718
H   0.889928   1.759738   0.554929
H   2.922815  -1.932279  -0.001813
H   2.538990  -1.524103  -1.679617
S   4.312620  -0.150906  -0.893491
H   4.536609   0.056680   0.420147
C  -2.248132  -1.773078   0.180358
C  -2.341723  -0.353939  -0.473338
O  -3.345299  -2.359132   0.343552
O  -1.096044  -2.132039   0.489700
H  -1.598644  -0.259230  -1.261346
C  -2.145938   0.722958   0.590733
N  -3.702998  -0.232386  -1.103843
H  -3.701245  -0.549039  -2.074028
H  -1.205932   0.536948   1.104366
H  -2.943616   0.677621   1.335222
S  -2.173431   2.446360  -0.058565
```



```
H  -4.308351  -0.880036  -0.568578
H  -4.059507   0.727204  -1.087144
H  -1.087902   2.314211  -0.851163
28
Dimer 270 of the n...z  type
C  -1.629311  -0.224903  -0.533348
C  -3.044477  -0.227283  -1.125057
N  -1.206143  -1.566592  -0.166040
H  -1.795838  -1.921535   0.582520
H  -0.256796  -1.550553   0.194500
H  -0.954435   0.148900  -1.313156
C  -1.471301   0.798382   0.595083
O  -0.843744   0.608084   1.614999
O  -2.083013   1.962773   0.324910
H  -1.905978   2.586170   1.047791
H  -3.301743   0.749287  -1.528646
H  -3.080698  -0.959643  -1.930930
S  -4.367329  -0.745873   0.056014
H  -4.389893   0.393237   0.777707
C   2.107734   0.949040  -1.094187
C   2.928050   0.788743   0.231541
O   1.035435   1.593994  -0.976884
O   2.619306   0.443181  -2.109637
H   3.682593   1.579124   0.240615
C   3.622630  -0.555624   0.395155
N   1.992747   1.079729   1.370901
H   2.456923   1.449604   2.200121
H   4.157321  -0.610287   1.344634
H   4.341451  -0.671521  -0.412142
S   2.476199  -1.999604   0.381805
H   1.301016   1.750645   1.002220
H   1.454269   0.246814   1.633421
H   2.139829  -1.888941  -0.920008
28
Dimer 271 of the n...z  type
C   1.925580   0.695036  -0.447320
C   3.245643   0.124902  -1.000610
N   2.149287   1.740640   0.534755
H   2.605302   1.350341   1.354948
H   1.240734   2.094355   0.827458
H   1.401591   1.132673  -1.300557
C   1.048525  -0.445022   0.069593
O   0.951845  -0.784198   1.223999
O   0.392190  -1.080175  -0.934200
H  -0.178017  -1.769922  -0.540471
H   3.047933  -0.608199  -1.782018
H   3.816425   0.946716  -1.433561
S   4.380094  -0.619186   0.249020
H   3.641073  -1.712855   0.525372
C  -1.905203   1.350062   0.556123
C  -2.819169   0.762636  -0.572551
O  -0.857407   1.909661   0.144109
O  -2.329497   1.221505   1.717655
H  -3.558680   1.530949  -0.812940
C  -3.568120  -0.513786  -0.213815
N  -1.970835   0.612420  -1.808677
H  -2.513254   0.515600  -2.668124
H  -4.133927  -0.892349  -1.066328
H  -4.272794  -0.273301   0.578734
S  -2.528203  -1.925111   0.361307
H  -1.374063   1.448001  -1.853622
H  -1.324695  -0.181533  -1.725640
H  -2.097632  -1.320459   1.487944
28
Dimer 272 of the n...z  type
C   2.900128  -0.918592   0.662478
C   2.639120  -1.707029  -0.626649
N   1.817147  -0.923586   1.650339
H   0.911192  -1.025447   1.202169
```

```
H   1.921936  -1.675009   2.321418
H   3.790464  -1.352194   1.131247
C   3.291952   0.540325   0.356278
O   4.019664   0.862107  -0.556394
O   2.772722   1.424084   1.206989
H   2.201063   0.879062   1.809041
H   3.521326  -1.696562  -1.264026
H   2.403792  -2.741118  -0.375138
S   1.197279  -1.100379  -1.606914
H   1.798363  -0.000798  -2.105139
C  -3.370347  -0.973572   0.551237
C  -2.375136  -0.045217  -0.220926
O  -4.579272  -0.840486   0.231150
O  -2.852685  -1.706170   1.413208
H  -1.524251  -0.618121  -0.582349
C  -1.906593   1.086777   0.693309
N  -3.110173   0.506014  -1.413945
H  -2.965340  -0.065081  -2.246511
H  -1.387452   0.647608   1.542451
H  -2.757784   1.654858   1.074219
S  -0.824727   2.332882  -0.127436
H  -4.113498   0.443998  -1.159585
H  -2.825165   1.465139  -1.635780
H   0.157532   1.474214  -0.471169
28
Dimer 273 of the n...z  type
C   2.742014   0.994994  -0.015409
C   3.171959  -0.044508   1.034503
N   1.815693   1.969002   0.529642
H   0.893099   1.564278   0.687323
H   1.678624   2.724336  -0.134155
H   3.662596   1.516346  -0.307071
C   2.246977   0.316660  -1.298957
O   1.194655   0.552902  -1.847990
O   3.132225  -0.581361  -1.770031
H   2.778642  -0.973308  -2.584897
H   3.959411  -0.685150   0.642727
H   3.555282   0.487999   1.904444
S   1.815874  -1.106225   1.693615
H   1.627522  -1.857637   0.589015
C  -2.237403   1.304260   0.328928
C  -2.909613  -0.064505   0.686748
O  -2.843571   1.986783  -0.535582
O  -1.201991   1.574402   0.963932
H  -3.382888   0.058017   1.662814
C  -1.942702  -1.243014   0.750373
N  -4.010195  -0.286433  -0.315351
H  -4.865945  -0.669834   0.083901
H  -2.434440  -2.131373   1.148357
H  -1.119641  -0.970792   1.407710
S  -1.270367  -1.755983  -0.885773
H  -4.167880   0.654919  -0.727243
H  -3.682312  -0.902899  -1.068730
H  -0.546597  -0.642682  -1.129439
28
Dimer 274 of the n...z  type
C   1.676738   0.121581   0.375659
C   2.881643   1.078942   0.444625
N   1.650978  -0.805902   1.490638
H   2.431451  -1.452727   1.420154
H   0.791507  -1.347518   1.430505
H   0.786356   0.752317   0.432221
C   1.635236  -0.555451  -0.995350
O   1.980598  -1.689375  -1.229623
O   1.195637   0.292219  -1.951026
H   1.222533  -0.161171  -2.808940
H   2.838147   1.809571  -0.362095
H   2.841371   1.609007   1.396309
S   4.539958   0.270309   0.435497
```



```
H   4.524685  -0.145593  -0.847472
C  -2.297759  -1.242896   0.410515
C  -2.903150  -0.298374  -0.682981
O  -3.139063  -1.789924   1.164964
O  -1.058131  -1.359910   0.388998
H  -2.946205  -0.869242  -1.612822
C  -2.110031   0.979082  -0.922505
N  -4.323935  -0.014898  -0.275735
H  -4.976132   0.049337  -1.057129
H  -2.560775   1.578903  -1.714469
H  -1.102972   0.707371  -1.227377
S  -2.035396   2.092988   0.545148
H  -4.570128  -0.796942   0.360353
H  -4.369713   0.853143   0.269685
H  -1.400803   1.225013   1.360146
28
Dimer 275 of the n...z  type
C   2.599835   0.621669   0.923218
C   3.190158  -0.734850   0.501769
N   1.555344   0.464895   1.917487
H   0.763199  -0.074513   1.566258
H   1.189205   1.371962   2.187807
H   3.427203   1.184672   1.373538
C   2.182202   1.434236  -0.308667
O   1.078536   1.886629  -0.504530
O   3.208180   1.599471  -1.167145
H   2.902402   2.123984  -1.924611
H   4.107014  -0.597482  -0.067147
H   3.418895  -1.303413   1.402590
S   2.048741  -1.806029  -0.479454
H   2.245050  -1.201285  -1.668805
C  -1.536730  -1.322849   0.411830
C  -1.457321  -0.144404  -0.610405
O  -1.983929  -2.403121  -0.049018
O  -1.123560  -1.065919   1.559093
H  -0.413202  -0.056985  -0.913272
C  -1.919798   1.198753  -0.063966
N  -2.242296  -0.564759  -1.823565
H  -1.824005  -0.276280  -2.707140
H  -1.777640   1.989789  -0.800465
H  -1.316624   1.435466   0.807283
S  -3.708663   1.250396   0.379359
H  -2.293567  -1.598993  -1.745553
H  -3.201427  -0.203896  -1.772964
H  -3.642988   0.344325   1.376670
28
Dimer 276 of the n...z  type
C   3.057573  -0.269556  -0.327549
C   3.102219   0.108750   1.158116
N   2.752820   0.811144  -1.268208
H   2.102635   1.482893  -0.867546
H   3.587621   1.309074  -1.553095
H   4.044506  -0.669979  -0.586579
C   2.087509  -1.438866  -0.582508
O   1.947682  -2.374571   0.172485
O   1.422755  -1.341360  -1.734336
H   1.712876  -0.471447  -2.113974
H   3.398503  -0.749043   1.759168
H   3.833539   0.903854   1.304026
S   1.514776   0.776318   1.821145
H   0.868027  -0.404423   1.902162
C  -3.021556  -1.191308   0.095647
C  -2.738134   0.337404  -0.096489
O  -2.761453  -1.635202   1.242970
O  -3.427684  -1.789951  -0.915839
H  -3.558738   0.818561  -0.624470
C  -1.423025   0.509593  -0.857230
N  -2.637360   0.947240   1.275443
H  -3.543215   1.272546   1.613608
```

```
H  -1.540422   0.086649  -1.852256
H  -0.621206  -0.030909  -0.354609
S  -0.815881   2.244013  -0.979962
H  -2.334076   0.172355   1.892544
H  -1.985298   1.738197   1.299837
H  -1.793199   2.700266  -1.790548
28
Dimer 277 of the n...z  type
C   2.811258  -0.237707   0.859191
C   3.019263  -1.293151  -0.240476
N   1.816592  -0.657254   1.827339
H   0.877370  -0.676709   1.429247
H   1.780351   0.002825   2.597391
H   3.777722  -0.147188   1.371091
C   2.550126   1.146215   0.250446
O   1.610587   1.858617   0.516446
O   3.511708   1.496089  -0.627396
H   3.311041   2.379142  -0.977140
H   3.878527  -1.043734  -0.859361
H   3.199533  -2.255082   0.238397
S   1.564948  -1.571027  -1.341566
H   1.695778  -0.448137  -2.077211
C  -2.183035  -1.015623   0.882699
C  -1.995982  -0.558038  -0.601951
O  -3.262145  -1.614719   1.121323
O  -1.236501  -0.761428   1.650849
H  -1.352005  -1.296552  -1.082782
C  -1.370219   0.819210  -0.769325
N  -3.347497  -0.649361  -1.257532
H  -3.319572  -0.953938  -2.230157
H  -1.214662   1.053048  -1.823741
H  -0.407260   0.827704  -0.267600
S  -2.406180   2.192801  -0.108414
H  -3.875179  -1.317761  -0.664451
H  -3.827503   0.256180  -1.202792
H  -2.325762   1.824218   1.186867
28
Dimer 278 of the n...z  type
C   2.920323   0.489381   0.603545
C   3.256768  -1.014022   0.535335
N   1.987855   0.805270   1.672839
H   1.021933   0.711009   1.372789
H   2.091849   1.777079   1.945398
H   3.872311   0.984367   0.821389
C   2.478428   0.989239  -0.774326
O   1.375842   1.409168  -1.038075
O   3.463919   0.875362  -1.683075
H   3.134035   1.173464  -2.546338
H   4.097524  -1.187095  -0.134315
H   3.530311  -1.347245   1.535551
S   1.892877  -2.145568   0.017367
H   1.883566  -1.826751  -1.293280
C  -1.723683   1.287656   0.496166
C  -1.525681  -0.070882  -0.255233
O  -1.298844   1.290509   1.684232
O  -2.237283   2.210184  -0.157230
H  -0.611129   0.040670  -0.841339
C  -2.665079  -0.464343  -1.184028
N  -1.249818  -1.111727   0.795552
H  -0.404084  -1.661890   0.596271
H  -2.428633  -1.380718  -1.727048
H  -2.810943   0.338276  -1.903052
S  -4.257211  -0.806274  -0.319661
H  -1.098854  -0.547688   1.657637
H  -2.054391  -1.726455   0.943354
H  -4.435941   0.443680   0.155386
28
Dimer 279 of the n...z  type
C   1.991936  -0.667384   0.550191
```



```
C   3.423271  -0.218119   0.899284
N   1.974496  -1.705485  -0.463750
H   2.284140  -1.322912  -1.352820
H   1.007613  -2.006636  -0.576256
H   1.565376  -1.078552   1.469265
C   1.139306   0.551513   0.194925
O   0.814263   0.873663  -0.925474
O   0.807017   1.264692   1.289760
H   0.282808   2.035329   1.016847
H   3.411232   0.508001   1.711200
H   3.986136  -1.093404   1.223886
S   4.402874   0.468958  -0.505468
H   3.732272   1.632169  -0.633171
C  -1.810599  -1.417639  -0.403107
C  -3.048864  -0.756859   0.292621
O  -0.898199  -1.782678   0.386335
O  -1.877898  -1.538103  -1.636350
H  -3.823010  -1.524543   0.353890
C  -3.615935   0.456355  -0.434706
N  -2.637510  -0.435506   1.703803
H  -3.363212  -0.610853   2.398001
H  -4.543078   0.794422   0.029985
H  -3.825170   0.165048  -1.461364
S  -2.508544   1.929838  -0.426458
H  -1.803863  -1.034826   1.865441
H  -2.338208   0.543961   1.772126
H  -1.477367   1.371202  -1.095997
28
Dimer 280 of the n...z  type
C  -2.827620  -1.235122   0.207667
C  -3.385059  -0.258585  -0.841349
N  -3.056113  -0.873970   1.610238
H  -3.168095   0.133018   1.706586
H  -3.880095  -1.325330   1.988414
H  -3.296256  -2.207136   0.019479
C  -1.323495  -1.498140  -0.005559
O  -0.826968  -1.718643  -1.087290
O  -0.617969  -1.512042   1.126147
H  -1.289375  -1.305281   1.830270
H  -3.034809  -0.546622  -1.831478
H  -4.474129  -0.308092  -0.832303
S  -2.990636   1.515817  -0.536880
H  -1.641446   1.475217  -0.750905
C   1.007565   1.627632  -0.036213
C   2.399721   0.946658  -0.237847
O   0.773923   2.040234   1.124231
O   0.276385   1.645798  -1.047848
H   2.859229   1.288896  -1.162856
C   2.230087  -0.571438  -0.237249
N   3.277290   1.373528   0.908595
H   3.775045   2.240985   0.706691
H   1.561708  -0.851849  -1.047606
H   1.763149  -0.904497   0.690549
S   3.806398  -1.517837  -0.352665
H   2.631489   1.561685   1.694378
H   3.970841   0.657159   1.146815
H   4.172145  -1.081538  -1.575782
28
Dimer 281 of the n...z  type
C   3.045016   0.443654   1.059060
C   3.628038  -0.953949   0.811411
N   1.622962   0.526737   1.386796
H   1.040440  -0.159695   0.900016
H   1.462259   0.427888   2.381661
H   3.607097   0.872768   1.897600
C   3.337144   1.395435  -0.116922
O   4.372969   1.395661  -0.746276
O   2.347025   2.251559  -0.361267
H   1.635377   1.978625   0.281301
```

Printed by sara


```
H   4.709667  -0.894826   0.699389
H   3.404470  -1.589948   1.668146
S   2.931812  -1.836291  -0.649779
H   3.544883  -1.084910  -1.586481
C  -1.307009  -0.384345  -0.440967
C  -2.866724  -0.480185  -0.513926
O  -0.781260  -1.043741   0.495728
O  -0.774221   0.359601  -1.279660
H  -3.190665  -0.634415  -1.540919
C  -3.479669   0.796629   0.062267
N  -3.279996  -1.678403   0.297931
H  -3.344054  -2.522441  -0.271869
H  -3.155071   1.636764  -0.547743
H  -3.130535   0.961621   1.083713
S  -5.317902   0.782412   0.187422
H  -2.510998  -1.815502   0.979479
H  -4.184225  -1.540342   0.760651
H  -5.568848   0.680084  -1.134051
28
Dimer 282 of the n...z  type
C   2.747317  -0.995565  -0.035031
C   3.167583   0.081802  -1.050857
N   1.803811  -1.939698  -0.601877
H   0.883034  -1.522143  -0.731840
H   1.669127  -2.718588   0.034382
H   3.668698  -1.534154   0.219705
C   2.280488  -0.359597   1.280311
O   1.215518  -0.568017   1.816118
O   3.209374   0.467216   1.796066
H   2.873492   0.836956   2.628659
H   3.961086   0.706050  -0.644298
H   3.540167  -0.418989  -1.944249
S   1.806478   1.168637  -1.657378
H   1.627160   1.878800  -0.524280
C  -2.252941  -1.296361  -0.350494
C  -2.925445   0.080827  -0.673935
O  -2.870944  -2.007980   0.481983
O  -1.208019  -1.544331  -0.979396
H  -3.403403  -0.020004  -1.650241
C  -1.961238   1.262039  -0.713932
N  -4.022383   0.278914   0.336896
H  -4.874629   0.684655  -0.047281
H  -2.458214   2.160224  -1.082631
H  -1.142970   1.010597  -1.385605
S  -1.275341   1.727724   0.930657
H  -4.188997  -0.673934   0.717074
H  -3.686108   0.866414   1.109150
H  -0.538657   0.613864   1.129321
28
Dimer 283 of the n...z  type
C  -1.758738  -0.762498  -0.245626
C  -1.971602   0.426335  -1.190107
N  -2.691466  -1.843256  -0.533801
H  -3.641945  -1.544190  -0.330034
H  -2.502873  -2.634770   0.073084
H  -0.743847  -1.130502  -0.439419
C  -1.745320  -0.346253   1.226626
O  -2.249304  -0.973445   2.128170
O  -1.076201   0.810320   1.429993
H  -1.056912   0.995816   2.382676
H  -1.179844   1.163538  -1.076694
H  -1.957984   0.058230  -2.215634
S  -3.601793   1.276590  -1.007200
H  -3.320418   1.912352   0.148454
C   1.819955  -1.345571  -0.083993
C   3.165058  -0.547704  -0.162944
O   1.352031  -1.703378  -1.197798
O   1.374130  -1.554228   1.056659
H   3.968957  -1.247754   0.074013
```



```
C   3.250119   0.645743   0.781719
N   3.344212  -0.141341  -1.600039
H   4.311361  -0.158470  -1.921811
H   4.245147   1.091840   0.760810
H   3.046047   0.295356   1.790661
S   2.091991   2.016376   0.359071
H   2.750744  -0.807362  -2.130546
H   2.957612   0.799177  -1.745896
H   0.949470   1.357001   0.647035
28
Dimer 284 of the n...z  type
C   2.054038  -0.462665   0.755568
C   1.633563   1.011436   0.733172
N   3.325505  -0.768009   1.419297
H   3.982829   0.001017   1.312577
H   3.200495  -0.942248   2.409337
H   1.264069  -1.015127   1.276923
C   2.078163  -1.068921  -0.660872
O   1.266324  -0.806521  -1.520737
O   3.064918  -1.943038  -0.852668
H   3.577897  -1.923526  -0.002539
H   0.666052   1.142373   0.248950
H   1.559016   1.376230   1.758283
S   2.853223   2.126655  -0.086634
H   2.610212   1.720929  -1.349662
C  -2.375732   1.658961  -0.246380
C  -2.319195   0.249251   0.434053
O  -3.533485   2.105859  -0.445337
O  -1.272031   2.149987  -0.544051
H  -1.590362   0.245955   1.242322
C  -1.971558  -0.807405  -0.613543
N  -3.677067  -0.013569   1.028596
H  -3.737671   0.309925   1.993998
H  -0.978634  -0.595843  -1.004866
H  -2.683765  -0.775595  -1.440807
S  -2.035513  -2.546413  -0.005078
H  -4.332121   0.559340   0.466226
H  -3.920267  -1.009216   1.011644
H  -1.041666  -2.441287   0.900119
28
Dimer 285 of the n...z  type
C   2.833667   0.340869   0.939067
C   3.574601  -1.002270   0.902350
N   1.416338   0.285573   1.297367
H   0.995415  -0.573471   0.951864
H   1.282704   0.318761   2.301117
H   3.344572   0.970590   1.676936
C   2.995345   1.104303  -0.388419
O   4.012268   1.092331  -1.046250
O   1.929147   1.818861  -0.746799
H   1.229220   1.632404  -0.073466
H   4.621831  -0.851727   0.646325
H   3.518704  -1.470139   1.885331
S   2.842079  -2.232373  -0.260962
H   3.214856  -1.611868  -1.398960
C  -3.343795  -1.478475  -0.316163
C  -3.188618   0.078100  -0.422966
O  -3.984840  -1.877580   0.689609
O  -2.785972  -2.131729  -1.214637
H  -3.432480   0.414326  -1.428605
C  -1.761488   0.476793  -0.051152
N  -4.179656   0.686743   0.533700
H  -5.059221   0.921372   0.073364
H  -1.081261   0.012144  -0.761632
H  -1.508390   0.118404   0.948158
S  -1.459572   2.292916   0.011818
H  -4.373239  -0.068220   1.219986
H  -3.822645   1.535643   0.982191
H  -1.730046   2.545906  -1.285467
```

```
28
Dimer 286 of the n...z  type
C  -1.466196   0.092636   0.472481
C  -2.536424  -0.945315   0.859427
N  -1.618730   1.336345   1.202334
H  -2.450656   1.825137   0.884405
H  -0.812623   1.923414   0.997220
H  -0.504320  -0.349279   0.748478
C  -1.439167   0.266410  -1.048468
O  -1.832043   1.236383  -1.650785
O  -0.940603  -0.830180  -1.660784
H  -0.944476  -0.684136  -2.620412
H  -2.352989  -1.893748   0.356521
H  -2.482763  -1.105599   1.936152
S  -4.280583  -0.429045   0.550819
H  -4.259539  -0.536491  -0.793598
C   1.940335   1.274346  -0.360258
C   3.126050   0.491602   0.301282
O   1.227743   1.927071   0.450092
O   1.852631   1.188571  -1.594592
H   4.022960   1.100147   0.167291
C   3.376304  -0.894795  -0.278588
N   2.845206   0.447790   1.778910
H   3.673445   0.549426   2.364269
H   4.262059  -1.350877   0.165778
H   3.539190  -0.790976  -1.348593
S   2.006577  -2.093183   0.017044
H   2.176604   1.228610   1.930227
H   2.364607  -0.424762   2.025266
H   1.100034  -1.508222  -0.794227
28
Dimer 287 of the n...z  type
C   3.270936   0.475301  -0.214679
C   2.845686  -0.636825  -1.194450
N   4.162008  -0.035272   0.812857
H   3.670538  -0.715960   1.386027
H   4.452266   0.714689   1.432335
H   3.814099   1.214911  -0.813190
C   2.031107   1.193741   0.326656
O   1.652678   1.159502   1.475443
O   1.392183   1.866916  -0.646314
H   0.556559   2.238924  -0.293249
H   2.217024  -0.226246  -1.982754
H   3.744064  -1.054470  -1.648794
S   1.994460  -2.082403  -0.426855
H   0.801236  -1.507705  -0.137947
C  -2.317528  -1.255434   0.579171
C  -2.711405  -0.487837  -0.729348
O  -3.288129  -1.646471   1.271699
O  -1.096196  -1.406522   0.773203
H  -2.692465  -1.211975  -1.546637
C  -1.797009   0.674873  -1.082686
N  -4.143929  -0.057411  -0.563106
H  -4.684805  -0.074773  -1.427395
H  -2.110931   1.162054  -2.006536
H  -0.790677   0.287827  -1.221998
S  -1.763868   2.010327   0.192060
H  -4.530184  -0.717576   0.138196
H  -4.192796   0.884143  -0.160495
H  -1.284974   1.237843   1.190652
28
Dimer 288 of the n...z  type
C  -2.506059  -0.375114  -1.055008
C  -3.576319  -0.715529  -0.003163
N  -1.439276  -1.353678  -1.060803
H  -0.932559  -1.360348  -0.176569
H  -0.733312  -1.131108  -1.756015
H  -3.018715  -0.403904  -2.025233
C  -2.034656   1.075968  -0.898888
```



```
O  -0.884831   1.430154  -0.775849
O  -3.065774   1.943308  -0.933289
H  -2.719773   2.846008  -0.844859
H  -4.441294  -0.063301  -0.102484
H  -3.894163  -1.746200  -0.158498
S  -2.987026  -0.656622   1.745548
H  -2.936425   0.686657   1.858204
C   1.830447  -1.352389  -0.145317
C   3.189109  -0.709554   0.294688
O   1.708140  -1.546656  -1.382384
O   1.037719  -1.615974   0.776841
H   3.795506  -1.503833   0.734186
C   3.042529   0.423165   1.305875
N   3.884158  -0.260601  -0.961553
H   4.887897  -0.437235  -0.969997
H   4.016542   0.751029   1.671696
H   2.460225   0.050425   2.145237
S   2.249438   1.938092   0.619669
H   3.396725  -0.783460  -1.716677
H   3.715059   0.740491  -1.118190
H   1.059173   1.385261   0.300553
28
Dimer 289 of the n...z  type
C  -2.060747   0.062802  -0.814827
C  -3.499184   0.609861  -0.879393
N  -2.014904  -1.371368  -1.028689
H  -2.432694  -1.853069  -0.237568
H  -1.037483  -1.650266  -1.080435
H  -1.508938   0.548326  -1.624775
C  -1.388823   0.518175   0.482012
O  -1.121333  -0.192087   1.423694
O  -1.144078   1.845478   0.473367
H  -0.777580   2.103729   1.334290
H  -3.503514   1.695941  -0.801346
H  -3.928023   0.327302  -1.840654
S  -4.663369  -0.084511   0.373334
H  -4.180197   0.576257   1.445226
C   1.703622  -1.238998  -0.331931
C   2.132065  -0.251389   0.802829
O   2.174282  -2.398899  -0.232415
O   0.919103  -0.775071  -1.182023
H   1.322724  -0.249553   1.535966
C   2.387671   1.176060   0.337673
N   3.337380  -0.851492   1.472023
H   3.386071  -0.674190   2.474923
H   2.626414   1.827250   1.179777
H   1.484547   1.544879  -0.142087
S   3.807327   1.335751  -0.826957
H   3.268992  -1.866050   1.270429
H   4.195544  -0.504458   1.028129
H   3.252072   0.619658  -1.826408
28
Dimer 290 of the n...z  type
C  -2.781903   0.399351  -0.787485
C  -1.546211   1.286511  -0.547852
N  -3.962013   0.968107  -0.158111
H  -3.843335   0.973682   0.851539
H  -4.777447   0.395749  -0.352575
H  -2.938901   0.379720  -1.871437
C  -2.495964  -1.047191  -0.370033
O  -3.014824  -1.616124   0.560852
O  -1.588760  -1.635587  -1.177064
H  -1.433171  -2.534991  -0.845748
H  -0.688827   0.910922  -1.103198
H  -1.774148   2.290358  -0.905500
S  -1.066883   1.494750   1.220552
H  -0.536338   0.256903   1.381473
C   1.780914  -0.962666   1.134569
C   2.681712  -0.994948  -0.145618
```

```
O   0.620329  -1.434741   0.979338
O   2.303706  -0.488623   2.154326
H   3.406580  -1.799630  -0.011701
C   3.429830   0.309751  -0.404673
N   1.794480  -1.374207  -1.300673
H   2.192809  -2.079316  -1.919392
H   4.146935   0.195585  -1.218439
H   3.967422   0.576016   0.502242
S   2.342990   1.707014  -0.921197
H   0.923429  -1.717482  -0.847244
H   1.550946  -0.541253  -1.849336
H   1.585204   1.763962   0.198053
28
Dimer 291 of the n...z  type
C  -2.568153   0.272629   1.094718
C  -2.539525   1.584710   0.293975
N  -1.318945   0.060706   1.805270
H  -0.555904  -0.040915   1.141826
H  -1.356220  -0.807110   2.330665
H  -3.372559   0.383179   1.832038
C  -2.985048  -0.904095   0.203407
O  -2.351686  -1.921779   0.054498
O  -4.167781  -0.673644  -0.400443
H  -4.397667  -1.443123  -0.945735
H  -3.526033   1.819241  -0.099756
H  -2.226877   2.388555   0.959650
S  -1.329122   1.615999  -1.101259
H  -2.051199   0.869670  -1.962189
C   2.667948   1.165611   0.082517
C   3.266777  -0.282170   0.149747
O   2.233949   1.609869   1.177648
O   2.718539   1.716313  -1.030226
H   4.352901  -0.181999   0.106721
C   2.807338  -1.202276  -0.977646
N   2.922449  -0.831508   1.507687
H   3.683487  -1.334701   1.961359
H   3.360166  -2.142591  -0.965621
H   2.996440  -0.697921  -1.922426
S   1.032949  -1.689600  -0.870500
H   2.644949   0.014215   2.048411
H   2.101438  -1.444946   1.443576
H   0.501122  -0.460853  -1.063210
28
Dimer 292 of the n...z  type
C   1.737743   0.033683  -0.172009
C   2.755998   0.949690  -0.873973
N   1.029130   0.729828   0.886156
H   1.676723   0.984001   1.626695
H   0.333216   0.105116   1.286372
H   1.016610  -0.260646  -0.942017
C   2.408193  -1.265950   0.284395
O   2.524514  -1.625986   1.431950
O   2.870790  -1.977641  -0.763227
H   3.294812  -2.785288  -0.431410
H   3.208762   0.443004  -1.724197
H   2.230387   1.834255  -1.233186
S   4.095019   1.606201   0.213446
H   4.805430   0.464440   0.318851
C  -1.619838  -0.822858  -0.523062
C  -2.067052   0.672521  -0.401626
O  -1.685275  -1.305055  -1.681060
O  -1.210668  -1.349459   0.529584
H  -1.154833   1.273119  -0.390081
C  -2.878352   0.988392   0.848060
N  -2.813176   1.010833  -1.662857
H  -2.658959   1.963180  -1.992496
H  -3.111353   2.052364   0.908224
H  -2.286538   0.713146   1.717510
S  -4.505835   0.125780   0.916222
```



```
H  -2.482480   0.312761  -2.354800
H  -3.820045   0.861076  -1.531419
H  -4.005402  -1.124608   0.995479
28
Dimer 293 of the n...z  type
C  -2.147059  -0.155768   0.767518
C  -2.262168  -1.061537  -0.471736
N  -3.091573  -0.548505   1.799007
H  -4.043488  -0.370465   1.490190
H  -2.947628   0.003494   2.638355
H  -1.126920  -0.286716   1.147246
C  -2.248744   1.315683   0.366202
O  -3.090699   2.094879   0.737275
O  -1.253390   1.674084  -0.488071
H  -1.367843   2.608075  -0.729201
H  -1.492374  -0.822322  -1.202365
H  -2.129844  -2.094820  -0.152335
S  -3.906179  -1.030189  -1.310948
H  -3.776738   0.180238  -1.892032
C   1.781091  -0.862541   0.749431
C   2.115010  -0.412735  -0.710039
O   1.028181  -0.096310   1.396786
O   2.295943  -1.944231   1.093147
H   1.550601  -1.048864  -1.395458
C   3.599249  -0.509595  -1.048853
N   1.613074   0.991188  -0.916316
H   1.582921   1.253017  -1.903137
H   3.792818  -0.213321  -2.080866
H   3.910320  -1.543010  -0.919058
S   4.667021   0.583146  -0.015157
H   0.671933   1.097554  -0.511229
H   2.231888   1.647787  -0.428295
H   4.455819  -0.065149   1.148789
28
Dimer 294 of the n...z  type
C   2.718083  -0.411230  -0.871687
C   1.348415   0.026743  -0.329088
N   2.882755  -1.852986  -0.793150
H   2.861063  -2.148805   0.179376
H   3.790841  -2.124224  -1.156813
H   2.733514  -0.116169  -1.927790
C   3.851598   0.378924  -0.206559
O   4.784651  -0.108267   0.387208
O   3.692962   1.707566  -0.368914
H   4.432839   2.166855   0.059862
H   1.190504   1.090513  -0.496180
H   0.564191  -0.536176  -0.833344
S   1.070012  -0.338772   1.457955
H   1.874214   0.622899   1.958242
C  -2.513960  -1.337170  -0.090426
C  -3.279376  -0.169524  -0.802367
O  -3.108151  -1.835524   0.900279
O  -1.427397  -1.656597  -0.603390
H  -3.690745  -0.575682  -1.728468
C  -2.413162   1.041767  -1.137123
N  -4.441809   0.192535   0.081227
H  -5.309593   0.373472  -0.421600
H  -2.955834   1.747906  -1.766805
H  -1.538764   0.691834  -1.681031
S  -1.883626   2.021931   0.331501
H  -4.532337  -0.624602   0.718679
H  -4.205179   1.010578   0.655836
H  -1.089611   1.079738   0.894233
28
Dimer 295 of the n...z  type
C  -1.872489  -0.073452  -0.768724
C  -1.969228   1.259504  -0.009943
N  -2.746894  -0.084293  -1.930083
H  -3.719392  -0.063521  -1.634342
```

```
H  -2.622014  -0.944196  -2.454445
H  -0.834159  -0.165274  -1.113071
C  -2.079112  -1.263232   0.172277
O  -2.863002  -2.165890  -0.002107
O  -1.252043  -1.205991   1.239438
H  -1.400317  -1.989029   1.793569
H  -1.232988   1.310009   0.789523
H  -1.779117   2.070934  -0.712024
S  -3.632974   1.626164   0.703065
H  -3.564133   0.761673   1.736324
C   1.946620  -1.211523  -0.452431
C   1.937071  -0.185283   0.730872
O   2.637821  -2.241512  -0.251592
O   1.249338  -0.907140  -1.438256
H   1.033008  -0.386371   1.309406
C   1.946687   1.276790   0.304751
N   3.110736  -0.523319   1.607836
H   2.947793  -0.364672   2.601624
H   1.882610   1.939831   1.168670
H   1.085203   1.454542  -0.333784
S   3.477059   1.780070  -0.590303
H   3.301175  -1.523256   1.409451
H   3.935339   0.013057   1.313973
H   3.280521   0.987247  -1.664011
28
Dimer 296 of the n...z  type
C  -2.485662   0.197308   1.013977
C  -2.428993   1.467162   0.147177
N  -1.167874  -0.221874   1.451673
H  -0.609773  -0.583052   0.677826
H  -1.251016  -0.985448   2.115259
H  -3.075337   0.470095   1.898202
C  -3.291272  -0.908024   0.318059
O  -2.903575  -2.035738   0.124765
O  -4.514495  -0.476273  -0.052875
H  -4.990052  -1.208540  -0.476983
H  -3.429615   1.855516  -0.030971
H  -1.849423   2.220948   0.679541
S  -1.573752   1.279412  -1.475726
H  -2.500027   0.501061  -2.072335
C   2.175946  -1.379554  -0.492393
C   3.008586  -0.658767   0.622956
O   2.828289  -1.691881  -1.520474
O   0.979038  -1.592117  -0.225621
H   3.247934  -1.410191   1.378326
C   2.307076   0.514673   1.296750
N   4.307783  -0.244624  -0.013587
H   5.119301  -0.329862   0.597299
H   2.917354   0.918658   2.105721
H   1.360743   0.172284   1.709352
S   1.983587   1.935841   0.169716
H   4.388709  -0.861066  -0.845815
H   4.239796   0.721894  -0.352463
H   1.066536   1.310413  -0.598936
28
Dimer 297 of the n...z  type
C   3.466803  -0.816737   0.023770
C   3.864332   0.583759   0.525912
N   3.942251  -1.045771  -1.330283
H   3.471438  -0.412226  -1.971057
H   3.707360  -1.986585  -1.630686
H   3.958373  -1.531740   0.692828
C   1.958630  -1.027683   0.202487
O   1.176710  -1.204142  -0.705532
O   1.600061  -0.972699   1.493133
H   0.622153  -1.056619   1.571418
H   3.603544   0.703314   1.575473
H   4.942522   0.698011   0.416012
S   3.140464   1.985499  -0.433236
```



```
H   1.875600   1.868136   0.020276
C  -3.462489  -0.233503  -1.122215
C  -2.799733   0.940217  -0.318384
O  -4.718353  -0.263718  -1.060755
O  -2.680578  -0.976172  -1.740158
H  -2.612267   1.748917  -1.028044
C  -1.493301   0.584289   0.372555
N  -3.842243   1.443240   0.644936
H  -3.816290   2.450872   0.799365
H  -1.077296   1.441401   0.904070
H  -0.779243   0.254068  -0.378269
S  -1.661736  -0.747697   1.639198
H  -4.741071   1.147575   0.215782
H  -3.763375   0.967514   1.549086
H  -1.979321  -1.727968   0.767531
28
Dimer 298 of the n...z  type
C  -2.231694   0.774568   0.483583
C  -1.637056  -0.569223   0.937501
N  -3.472805   1.068795   1.179884
H  -4.177380   0.378753   0.932216
H  -3.836358   1.970343   0.887603
H  -1.492308   1.538673   0.751581
C  -2.352158   0.830151  -1.043446
O  -3.374697   1.022579  -1.656607
O  -1.157752   0.642843  -1.643787
H  -1.273644   0.692081  -2.606324
H  -0.656424  -0.744522   0.499174
H  -1.540377  -0.549655   2.023092
S  -2.695021  -2.039612   0.579230
H  -2.401012  -2.140165  -0.733408
C   2.384241  -1.241434  -0.489471
C   3.088583  -0.407556   0.635534
O   3.028642  -1.329200  -1.566073
O   1.288155  -1.741013  -0.180441
H   3.509454  -1.119755   1.347821
C   2.164441   0.549324   1.380507
N   4.242726   0.307254  -0.013149
H   5.091289   0.332737   0.551172
H   2.681595   1.025734   2.214448
H   1.328735  -0.024668   1.773718
S   1.534308   1.938791   0.346915
H   4.389842  -0.214904  -0.900605
H   3.972681   1.266539  -0.259566
H   0.812933   1.192966  -0.516365
28
Dimer 299 of the n...z  type
C  -3.580555   0.221897   1.004070
C  -2.488349  -0.832083   0.783143
N  -4.964031  -0.261277   0.980320
H  -5.064617  -1.046383   0.341496
H  -5.275231  -0.557279   1.897681
H  -3.397797   0.673523   1.985830
C  -3.444569   1.392567   0.011010
O  -2.383214   1.857026  -0.339728
O  -4.613407   1.877125  -0.408661
H  -5.290069   1.285944   0.012927
H  -1.502944  -0.372823   0.836878
H  -2.561154  -1.593590   1.559785
S  -2.630150  -1.748480  -0.809703
H  -2.229634  -0.737980  -1.608163
C   4.998500   0.508201   0.163817
C   3.761405  -0.405234  -0.135522
O   5.222382   0.711445   1.384255
O   5.585999   0.953987  -0.837529
H   4.010022  -1.147899  -0.890835
C   2.586955   0.459900  -0.594140
N   3.420782  -1.127424   1.140047
H   3.913804  -2.017544   1.213587
```

```
H   2.866060   0.950338  -1.524110
H   2.368079   1.232154   0.146198
S   0.997446  -0.446534  -0.810338
H   3.771559  -0.513926   1.897562
H   2.415367  -1.310487   1.223794
H   1.408168  -1.271065  -1.795510
28
Dimer 300 of the n...z  type
C  -2.732823  -0.880138  -0.123699
C  -1.448490  -0.051452   0.049657
N  -3.001130  -1.152154  -1.525402
H  -3.176225  -0.279798  -2.017380
H  -3.841894  -1.712810  -1.621487
H  -2.549792  -1.832539   0.387551
C  -3.904851  -0.227086   0.618211
O  -4.938725   0.135222   0.107953
O  -3.648399  -0.101268   1.935625
H  -4.411724   0.316094   2.366060
H  -1.208286   0.064462   1.104883
H  -0.623335  -0.561396  -0.448529
S  -1.494462   1.619398  -0.733553
H  -2.271344   2.226322   0.187339
C   2.325986  -1.347352  -0.519044
C   2.351510  -0.533602   0.821689
O   3.160375  -2.285827  -0.581228
O   1.471757  -0.992073  -1.350753
H   1.547708  -0.923470   1.449975
C   2.172000   0.969756   0.657827
N   3.647492  -0.874676   1.506859
H   3.582519  -0.927826   2.522920
H   2.153963   1.471200   1.626598
H   1.225104   1.155993   0.157529
S   3.528532   1.779337  -0.290756
H   3.920781  -1.787131   1.093296
H   4.370220  -0.191918   1.251908
H   3.272599   1.161688  -1.462528
28
Dimer 301 of the n...z  type
C  -1.420202  -0.100336   0.571674
C  -2.264656  -1.170815  -0.143388
N  -1.820510   0.056706   1.958127
H  -2.754586   0.454277   2.008815
H  -1.199111   0.704178   2.432509
H  -0.392550  -0.476471   0.547931
C  -1.431666   1.204556  -0.225157
O  -1.894057   2.253690   0.145101
O  -0.862746   1.047993  -1.450159
H  -0.909214   1.892486  -1.927723
H  -1.893176  -1.343775  -1.151629
H  -2.183106  -2.099009   0.421205
S  -4.074000  -0.817946  -0.222064
H  -4.023497   0.130508  -1.179928
C   2.296609  -1.515275   0.362892
C   2.767340  -0.577645  -0.799841
O   1.272141  -2.199766   0.111055
O   2.994787  -1.487000   1.392107
H   3.603136  -1.075132  -1.297787
C   3.219518   0.805854  -0.352572
N   1.649252  -0.518275  -1.806883
H   1.971605  -0.377022  -2.764204
H   3.460786   1.435620  -1.210115
H   4.116565   0.692717   0.251372
S   1.962709   1.741736   0.618851
H   1.154816  -1.420150  -1.713681
H   0.955717   0.209392  -1.582811
H   1.989409   0.925358   1.692258
28
Dimer 302 of the n...z  type
C  -2.220102  -0.641312   0.535377
```



```
C  -3.639657  -1.162932   0.279172
N  -2.072267   0.337274   1.616899
H  -2.914164   0.899093   1.719281
H  -1.876587  -0.108521   2.505079
H  -1.595923  -1.509462   0.775079
C  -1.594979  -0.059113  -0.747221
O  -1.725137  -0.548205  -1.845603
O  -0.854521   1.030339  -0.527686
H  -0.966467   1.205016   0.445940
H  -3.633482  -1.883644  -0.536725
H  -4.009602  -1.657192   1.177503
S  -4.873550   0.156610  -0.089187
H  -4.400676   0.479901  -1.310229
C   3.195503  -1.586344  -0.196797
C   2.369658  -0.280208   0.053210
O   3.752418  -2.064522   0.824507
O   3.233437  -1.974717  -1.377664
H   1.398738  -0.340979  -0.432443
C   3.145905   0.929746  -0.466805
N   2.147410  -0.170614   1.538224
H   1.274056  -0.612932   1.825000
H   3.276869   0.812174  -1.540302
H   4.134488   0.983465  -0.006041
S   2.357975   2.557235  -0.105545
H   2.924801  -0.708502   1.963786
H   2.129127   0.804063   1.855146
H   1.194476   2.307203  -0.741266
28
Dimer 303 of the n...z  type
C   2.398778  -0.431592  -0.735466
C   1.661906   0.896627  -0.483814
N   1.633545  -1.563405  -0.239425
H   1.611901  -1.545975   0.776804
H   2.080517  -2.435409  -0.504123
H   2.490623  -0.524455  -1.823410
C   3.830283  -0.367361  -0.191949
O   4.279376  -1.080979   0.674071
O   4.546908   0.600926  -0.796027
H   5.440228   0.614518  -0.416493
H   2.206057   1.728464  -0.926977
H   0.671341   0.838324  -0.937129
S   1.340401   1.276193   1.293449
H   2.594291   1.636518   1.636202
C  -2.442815   1.076007  -0.878399
C  -2.284473   0.509614   0.574562
O  -3.491711   1.741541  -1.073881
O  -1.506957   0.828250  -1.660196
H  -1.614832   1.185763   1.110627
C  -1.727422  -0.905418   0.649909
N  -3.634650   0.622407   1.230713
H  -3.599313   0.860249   2.221558
H  -1.596110  -1.219771   1.686501
H  -0.758421  -0.935752   0.156701
S  -2.825397  -2.174946  -0.111945
H  -4.124505   1.353326   0.679666
H  -4.157028  -0.253245   1.113701
H  -2.715560  -1.720672  -1.377512
28
Dimer 304 of the n...z  type
C  -1.564014  -0.030286  -0.165270
C  -2.419190   1.164223  -0.615378
N  -1.425787  -1.012536  -1.228357
H  -2.333228  -1.414183  -1.449211
H  -0.834712  -1.779409  -0.921106
H  -0.575328   0.388480   0.061685
C  -2.078368  -0.613953   1.152798
O  -2.362751  -1.774227   1.339541
O  -2.162345   0.327145   2.115430
H  -2.475093  -0.089642   2.934302
```

```
H  -2.417736   1.946418   0.140617
H  -1.993541   1.565738  -1.534713
S  -4.168518   0.750833  -1.042003
H  -4.608951   0.569059   0.220017
C   2.464089   1.369498   0.389971
C   2.989305   0.514173  -0.815225
O   3.352389   1.744436   1.195329
O   1.240529   1.598848   0.398832
H   3.105899   1.193146  -1.662668
C   2.081262  -0.636570  -1.224619
N   4.368285   0.042520  -0.438686
H   5.024655   0.006304  -1.218299
H   2.486682  -1.170983  -2.084944
H   1.108965  -0.233980  -1.498676
S   1.869073  -1.919389   0.083238
H   4.677830   0.718007   0.286565
H   4.319821  -0.883390   0.000595
H   1.327302  -1.100690   1.008669
28
Dimer 305 of the n...z  type
C   2.740201   0.401273   0.946245
C   1.382315   0.996242   0.531572
N   3.832180   1.314224   0.659191
H   3.933235   1.433516  -0.345028
H   4.709971   0.934018   0.998589
H   2.684491   0.252674   2.029931
C   2.909339  -0.993367   0.331837
O   3.729640  -1.290439  -0.504341
O   2.012098  -1.863733   0.832887
H   2.130867  -2.724662   0.400449
H   0.570528   0.323891   0.803835
H   1.244242   1.946498   1.046543
S   1.230019   1.409002  -1.261013
H   1.189985   0.142933  -1.725180
C  -2.978198   0.311119   1.124841
C  -3.077968   0.361377  -0.439166
O  -2.496574   1.344264   1.655450
O  -3.408422  -0.725925   1.661593
H  -4.101991   0.657977  -0.680266
C  -2.778152  -0.954947  -1.143382
N  -2.192170   1.487163  -0.898360
H  -2.546271   1.977902  -1.718731
H  -2.819844  -0.837253  -2.227395
H  -3.536378  -1.676461  -0.848083
S  -1.112691  -1.654392  -0.782351
H  -2.140638   2.120539  -0.079825
H  -1.222337   1.188691  -1.094266
H  -1.332343  -1.867235   0.531055
28
Dimer 306 of the n...z  type
C   3.355222  -0.116539  -0.705667
C   2.146453  -1.044045  -0.913850
N   4.509659  -0.848747  -0.216277
H   4.309716  -1.238979   0.700924
H   5.300556  -0.223272  -0.100182
H   3.596454   0.292711  -1.694198
C   2.976384   1.096152   0.155377
O   3.551016   1.442600   1.159083
O   1.912778   1.754034  -0.351663
H   1.717419   2.519547   0.212812
H   1.334019  -0.507546  -1.398978
H   2.448021  -1.869269  -1.558264
S   1.518039  -1.857015   0.618884
H   1.083232  -0.742884   1.242927
C  -3.417167  -0.860540   0.514997
C  -2.065765  -0.572719  -0.223723
O  -4.329611  -1.320960  -0.218993
O  -3.425835  -0.629436   1.736800
H  -1.405410  -1.422816  -0.036218
```



```
C  -1.359081   0.702044   0.216816
N  -2.369068  -0.572006  -1.697618
H  -1.632587  -0.977308  -2.274491
H  -0.403490   0.826352  -0.292972
H  -1.175857   0.637229   1.286800
S  -2.298687   2.244828  -0.146376
H  -3.252512  -1.113308  -1.771763
H  -2.562436   0.381741  -2.022587
H  -3.333963   1.982046   0.677810
28
Dimer 307 of the n...z  type
C   2.308735  -1.019654   0.108599
C   3.130382  -0.192563   1.110907
N   3.162681  -1.868860  -0.703847
H   3.777449  -1.292675  -1.273201
H   2.601952  -2.417231  -1.348317
H   1.650930  -1.661900   0.707840
C   1.367432  -0.126890  -0.708623
O   1.273398  -0.132340  -1.911371
O   0.623994   0.678728   0.083102
H  -0.037103   1.173956  -0.456083
H   2.479111   0.334176   1.804879
H   3.769364  -0.870657   1.675711
S   4.284196   1.023219   0.337768
H   3.351060   1.928468  -0.021423
C  -2.929555  -1.256117  -0.085210
C  -2.886435  -0.186692   1.060369
O  -2.114884  -2.202082   0.049111
O  -3.781571  -1.062771  -0.972311
H  -3.656125  -0.476021   1.780242
C  -3.170353   1.249823   0.637962
N  -1.573746  -0.358520   1.779444
H  -1.608370  -0.079777   2.759884
H  -2.996301   1.943375   1.462020
H  -4.219983   1.313001   0.360520
S  -2.184756   1.906995  -0.778590
H  -1.356679  -1.364728   1.688874
H  -0.797381   0.136195   1.320239
H  -2.600230   0.982348  -1.669476
28
Dimer 308 of the n...z  type
C  -2.743561  -0.286308   0.875460
C  -1.893876   0.953415   0.569525
N  -2.077861  -1.584031   0.741185
H  -1.376926  -1.565301   0.004607
H  -1.631703  -1.873803   1.602950
H  -3.090096  -0.190641   1.910698
C  -4.036539  -0.300119   0.035537
O  -4.677794   0.692447  -0.227195
O  -4.408145  -1.518840  -0.355214
H  -3.681763  -2.113351  -0.033806
H  -2.481646   1.858744   0.709370
H  -1.041238   0.986615   1.247626
S  -1.168131   0.968763  -1.125329
H  -2.309541   1.231964  -1.793014
C   3.162024  -1.559841  -0.481104
C   2.314794  -0.340956   0.017044
O   3.777548  -2.181745   0.421392
O   3.154377  -1.744084  -1.711309
H   1.328325  -0.356305  -0.441258
C   3.042905   0.963141  -0.311903
N   2.145027  -0.488805   1.504751
H   1.311478  -1.028311   1.738218
H   3.128800   1.043069  -1.393293
H   4.049779   0.957484   0.110657
S   2.251713   2.482893   0.369102
H   2.965577  -1.035957   1.819703
H   2.079147   0.418894   1.977565
H   1.099949   2.392041  -0.330677
```

```
28
Dimer 309 of the n...z  type
C  -3.324704  -0.218370  -0.830817
C  -2.376389   0.963134  -1.106351
N  -4.619942   0.245157  -0.364886
H  -4.518412   0.701941   0.537641
H  -5.246970  -0.540759  -0.225157
H  -3.461670  -0.728000  -1.791430
C  -2.665486  -1.247311   0.094991
O  -3.069133  -1.560363   1.189153
O  -1.562382  -1.787308  -0.466099
H  -1.193768  -2.446600   0.143862
H  -1.440428   0.613343  -1.537341
H  -2.859111   1.626404  -1.823559
S  -2.009171   2.029318   0.353888
H  -1.148535   1.189381   0.972512
C   3.868822   0.641832  -0.190767
C   2.782067  -0.263909  -0.867939
O   4.937562   0.047347   0.103176
O   3.562200   1.838039  -0.046866
H   2.852821  -0.101743  -1.945657
C   1.354253   0.011952  -0.419412
N   3.188156  -1.690913  -0.613535
H   2.984457  -2.325667  -1.384882
H   0.641934  -0.617896  -0.952628
H   1.125393   1.055264  -0.625130
S   1.053193  -0.334890   1.365612
H   4.207704  -1.633764  -0.425562
H   2.737368  -2.043447   0.238026
H   1.859991   0.635308   1.843109
28
Dimer 310 of the n...z  type
C  -1.980938   0.129761  -0.088953
C  -2.246785  -1.377242   0.003662
N  -2.028036   0.875932   1.169877
H  -2.698522   0.463896   1.814013
H  -1.119510   0.903278   1.619289
H  -0.979757   0.256532  -0.516917
C  -2.915484   0.809609  -1.107517
O  -3.256065   0.302604  -2.152985
O  -3.292261   2.036238  -0.747563
H  -2.899358   2.155887   0.156886
H  -2.159411  -1.838608  -0.978482
H  -1.513421  -1.832365   0.669233
S  -3.885067  -1.812232   0.730412
H  -4.634643  -1.393174  -0.309596
C   4.205240  -0.795374  -0.608826
C   2.800308  -0.172093  -0.294832
O   4.492588  -1.816363   0.067294
O   4.878488  -0.189392  -1.459586
H   2.261573   0.027837  -1.218550
C   2.976974   1.108797   0.517159
N   2.032437  -1.203218   0.489708
H   1.446172  -1.780498  -0.112980
H   3.587692   1.798377  -0.061289
H   3.495161   0.901943   1.455931
S   1.408009   1.943464   1.008165
H   2.769382  -1.815579   0.892546
H   1.434883  -0.793488   1.212064
H   0.994001   2.251822  -0.237918
28
Dimer 311 of the n...z  type
C  -2.972770  -0.910317   0.507280
C  -3.668262   0.447852   0.326575
N  -2.286112  -0.985783   1.785896
H  -1.575660  -0.260826   1.838945
H  -1.801556  -1.873291   1.875060
H  -3.769621  -1.664908   0.497276
C  -2.083722  -1.254952  -0.694423
```



```
O  -0.939960  -1.638805  -0.620792
O  -2.736182  -1.104864  -1.862012
H  -2.143544  -1.357896  -2.588174
H  -4.298123   0.445566  -0.559914
H  -4.293079   0.635269   1.199347
S  -2.522738   1.893536   0.234509
H  -2.101188   1.687032  -1.029883
C   2.996828  -1.136942  -0.070990
C   1.771102  -0.335982   0.483326
O   3.868713  -1.427896   0.789874
O   2.961178  -1.418308  -1.282024
H   0.962753  -1.050096   0.648019
C   1.257199   0.762936  -0.436046
N   2.180309   0.199053   1.829435
H   1.431665   0.208131   2.521174
H   0.370515   1.239443  -0.016577
H   0.987544   0.310986  -1.386597
S   2.462223   2.129158  -0.715667
H   2.959900  -0.422840   2.119579
H   2.560125   1.147437   1.733531
H   3.386896   1.383428  -1.355263
28
Dimer 312 of the n...z  type
C   2.135313  -0.312086   0.691929
C   1.551923   0.847143  -0.132154
N   2.647477   0.153989   1.969816
H   3.451648   0.757750   1.819897
H   2.974662  -0.630594   2.524309
H   1.294441  -0.992566   0.871191
C   3.153123  -1.109671  -0.127861
O   4.289715  -1.345931   0.209806
O   2.628260  -1.540732  -1.293298
H   3.304110  -2.046003  -1.772922
H   1.069899   0.470295  -1.031506
H   0.808795   1.356632   0.479842
S   2.764314   2.153294  -0.611404
H   3.412973   1.441785  -1.556544
C  -1.454355  -0.410436   0.539794
C  -2.181583   0.333769  -0.632435
O  -0.975923  -1.533544   0.234118
O  -1.396365   0.204890   1.617744
H  -1.473418   1.058568  -1.040353
C  -3.455460   1.063070  -0.226975
N  -2.427892  -0.689034  -1.707940
H  -2.334150  -0.328197  -2.656830
H  -3.878635   1.613704  -1.067945
H  -3.205398   1.768548   0.561635
S  -4.799958  -0.055646   0.353348
H  -1.731679  -1.434264  -1.512438
H  -3.360957  -1.101995  -1.597465
H  -4.158369  -0.498924   1.454147
28
Dimer 313 of the n...z  type
C  -3.337181   0.633889  -0.347095
C  -3.513099  -0.515773   0.663503
N  -3.551238   0.182571  -1.710755
H  -2.855824  -0.517896  -1.954158
H  -3.431604   0.953032  -2.360636
H  -4.108985   1.372774  -0.100791
C  -2.003689   1.358243  -0.131281
O  -1.128600   1.474587  -0.957655
O  -1.926675   1.887080   1.105084
H  -1.083300   2.357088   1.195501
H  -3.386996  -0.148103   1.680122
H  -4.524326  -0.908820   0.559287
S  -2.408043  -1.967498   0.399120
H  -1.235693  -1.407013   0.802482
C   1.218182  -0.652746   0.103584
C   1.993756   0.684412  -0.127265
```

```
O   0.635675  -0.730694   1.221421
O   1.223787  -1.464543  -0.834744
H   1.287349   1.359591  -0.615217
C   3.254398   0.560853  -0.969433
N   2.277974   1.262914   1.232889
H   2.266050   2.282211   1.259865
H   3.712856   1.535601  -1.140853
H   2.981916   0.130668  -1.930001
S   4.569167  -0.472264  -0.190887
H   1.542285   0.863455   1.843668
H   3.186452   0.930219   1.575467
H   3.876968  -1.629717  -0.226335
28
Dimer 314 of the n...z  type
C  -2.146430   0.647754  -1.004405
C  -3.327450  -0.334752  -0.939424
N  -0.944041   0.010366  -1.506741
H  -0.572242  -0.663528  -0.838392
H  -0.214475   0.700385  -1.651607
H  -2.446155   1.429312  -1.714499
C  -1.954983   1.382003   0.329864
O  -0.898881   1.526365   0.901995
O  -3.112955   1.883724   0.799438
H  -2.936910   2.357429   1.628418
H  -4.255084   0.188059  -0.716660
H  -3.422760  -0.818701  -1.910949
S  -3.122925  -1.716324   0.266455
H  -3.340214  -0.992525   1.383786
C   1.760733  -1.291521   0.808909
C   3.091009  -0.828039   0.123556
O   0.967103  -1.918298   0.056328
O   1.650917  -1.013373   2.013601
H   3.888125  -1.474414   0.494892
C   3.461093   0.625668   0.402552
N   2.945294  -1.101106  -1.349352
H   3.775568  -1.501788  -1.783539
H   4.453462   0.857374   0.013729
H   3.465052   0.771527   1.480199
S   2.336276   1.854498  -0.386938
H   2.134764  -1.751145  -1.402801
H   2.685757  -0.240990  -1.846274
H   1.209419   1.529403   0.286867
28
Dimer 315 of the n...z  type
C   3.564264   0.518399  -0.018757
C   3.347937  -0.654318  -0.994553
N   4.210760   0.074253   1.204733
H   3.608569  -0.586716   1.688634
H   4.349336   0.857595   1.835277
H   4.231852   1.221735  -0.529851
C   2.253561   1.279173   0.209311
O   1.678605   1.379308   1.267699
O   1.812355   1.849796  -0.928140
H   0.965436   2.286535  -0.747125
H   2.924930  -0.293986  -1.930628
H   4.316867  -1.108010  -1.201858
S   2.290974  -2.020760  -0.346404
H   1.086334  -1.396039  -0.494734
C  -1.294603  -0.306996   0.220036
C  -2.319711   0.875515   0.176631
O  -0.590140  -0.423149  -0.820791
O  -1.267414  -0.973525   1.265399
H  -1.851443   1.720872   0.685776
C  -3.660658   0.574496   0.832910
N  -2.473848   1.265630  -1.268858
H  -2.601718   2.266043  -1.418322
H  -4.309756   1.451089   0.823355
H  -3.478072   0.286448   1.865218
S  -4.615996  -0.754124  -0.015300
```



```
H  -1.606917   0.923870  -1.724243
H  -3.263582   0.760489  -1.687483
H  -3.741855  -1.748002   0.245312
28
Dimer 316 of the n...z  type
C   2.159412  -0.770087  -0.068587
C   2.114083   0.511726  -0.909513
N   1.658849  -0.662779   1.304800
H   1.826694   0.267739   1.680063
H   0.666052  -0.853828   1.361505
H   1.557116  -1.521368  -0.591759
C   3.578368  -1.371413  -0.033516
O   4.327305  -1.384279  -0.984832
O   3.901345  -1.910525   1.141629
H   3.119960  -1.716231   1.722852
H   2.494767   0.320102  -1.911139
H   1.083615   0.858028  -0.983389
S   3.031460   1.931047  -0.173880
H   4.271886   1.437788  -0.367932
C  -4.273792  -0.938745   0.021713
C  -2.845434  -0.312299   0.177689
O  -4.529139  -1.391286  -1.123598
O  -4.993478  -0.882974   1.033776
H  -2.370655  -0.681299   1.084375
C  -2.950370   1.212240   0.203612
N  -2.033219  -0.769557  -1.004600
H  -1.508577  -1.620612  -0.801461
H  -3.547599   1.499323   1.066206
H  -3.451853   1.579623  -0.694381
S  -1.333383   2.097001   0.240411
H  -2.739316  -1.001668  -1.729884
H  -1.369809  -0.057120  -1.322651
H  -0.922797   1.631093   1.437517
28
Dimer 1 of the z...z  type
C   0.530028   1.668862   0.134520
C   1.957368   1.067280   0.296421
O  -0.200790   1.632448   1.169871
O   0.251790   2.131120  -0.980426
H   2.658713   1.899597   0.382312
C   2.338715   0.178390  -0.885099
N   2.023015   0.293664   1.579754
H   2.969956  -0.047006   1.757614
H   2.250889   0.765865  -1.795382
H   1.662984  -0.674686  -0.962503
S   4.029303  -0.548593  -0.765922
H   1.711331   0.877062   2.356586
H   1.361384  -0.525531   1.512435
H   4.709835   0.611811  -0.866752
C  -0.530268  -1.668942   0.136022
C  -1.957364  -1.066883   0.297460
O   0.201191  -1.630510   1.170895
O  -0.252723  -2.133844  -0.977999
H  -2.658927  -1.898919   0.384507
C  -2.338773  -0.179170  -0.884970
N  -2.022545  -0.291825   1.579901
H  -1.711974  -0.874687   2.357525
H  -2.251108  -0.767683  -1.794605
H  -1.662791   0.673674  -0.963223
S  -4.029183   0.548130  -0.766278
H  -1.360364   0.526665   1.511921
H  -2.969295   0.049850   1.757080
H  -4.709809  -0.612420  -0.864774
28
Dimer 2 of the z...z  type
C   1.784535  -0.844415   0.700633
C   2.206667  -0.447138  -0.740482
O   1.057240  -0.014331   1.323216
O   2.246209  -1.916590   1.112500
```

```
H   1.905085  -1.244798  -1.421229
C   3.720390  -0.257443  -0.840848
N   1.468585   0.785158  -1.162852
H   1.763784   1.111020  -2.083820
H   4.015637  -0.019287  -1.864142
H   4.199443  -1.190503  -0.550930
S   4.381080   1.121781   0.192261
H   0.434215   0.569675  -1.192972
H   1.630048   1.532975  -0.484395
H   4.097516   0.567210   1.388810
C  -1.784601  -0.844317  -0.700762
C  -2.206723  -0.447211   0.740402
O  -1.057305  -0.014161  -1.323249
O  -2.246281  -1.916441  -1.112757
H  -1.905134  -1.244952   1.421052
C  -3.720446  -0.257533   0.840801
N  -1.468640   0.785037   1.162910
H  -1.763844   1.110803   2.083912
H  -4.015689  -0.019513   1.864128
H  -4.199500  -1.190555   0.550763
S  -4.381143   1.121827  -0.192121
H  -0.434271   0.569545   1.193014
H  -1.630094   1.532928   0.484534
H  -4.097572   0.567422  -1.388745
28
Dimer 3 of the z...z  type
C  -0.677447  -0.835422  -0.555541
C  -1.943423  -0.177344   0.059582
O  -0.424157  -0.547156  -1.738667
O  -0.039067  -1.608985   0.212143
H  -1.815985  -0.081385   1.135794
C  -3.175770  -1.011557  -0.277233
N  -2.051082   1.210402  -0.491459
H  -1.248060   1.755987  -0.079113
H  -3.035571  -2.020447   0.108709
H  -3.306541  -1.073787  -1.359328
S  -4.755691  -0.316461   0.373373
H  -1.933349   1.179004  -1.507937
H  -2.947574   1.643775  -0.259017
H  -4.451671  -0.448819   1.680623
C   1.267567   1.780473   0.109856
C   2.318938   0.736933   0.587166
O   0.190582   1.814154   0.778631
O   1.586559   2.470698  -0.866996
H   3.147865   1.280036   1.044365
C   2.820801  -0.113069  -0.578703
N   1.717457  -0.135656   1.652907
H   2.430477  -0.706877   2.109149
H   3.246598   0.551149  -1.326488
H   1.990473  -0.657037  -1.030776
S   4.056031  -1.394802  -0.099266
H   1.238110   0.441973   2.344289
H   0.998855  -0.773197   1.218604
H   5.040978  -0.543162   0.253634
28
Dimer 4 of the z...z  type
C   1.169207  -0.763937  -0.286567
C   2.025578   0.512208  -0.503020
O   0.864636  -1.027956   0.915231
O   0.871655  -1.392712  -1.311826
H   1.538285   1.113745  -1.271390
C   3.444673   0.168124  -0.950122
N   2.021520   1.336153   0.751504
H   2.614907   2.162042   0.667306
H   4.018145   1.074221  -1.152673
H   3.378358  -0.410004  -1.870358
S   4.434321  -0.762097   0.298833
H   1.029935   1.635269   0.953132
H   2.349332   0.767967   1.536144
```



```
H   3.665637  -1.870456   0.314318
C  -1.130562   1.368793  -0.135271
C  -2.013882   0.094595  -0.034592
O  -0.635687   1.785522   0.955331
O  -0.987389   1.838023  -1.272969
H  -1.697231  -0.595897  -0.816088
C  -3.487827   0.455787  -0.195742
N  -1.753980  -0.588014   1.273785
H  -0.756885  -0.932589   1.269283
H  -3.612919   0.976399  -1.143322
H  -3.809589   1.121931   0.607603
S  -4.629842  -0.990059  -0.121080
H  -1.839428   0.085639   2.037850
H  -2.402988  -1.364147   1.423340
H  -4.168851  -1.603047  -1.230467
28
Dimer 5 of the z...z  type
C   1.278344  -0.310986   0.933931
C   2.088404  -0.682882  -0.335573
O   0.325575   0.507293   0.762703
O   1.656726  -0.840966   1.986978
H   2.050281  -1.767122  -0.449344
C   3.546612  -0.243350  -0.218264
N   1.433652  -0.097337  -1.551422
H   0.493204  -0.555654  -1.706211
H   4.116816  -0.547428  -1.097680
H   3.977379  -0.725384   0.657196
S   3.775683   1.583466  -0.091495
H   1.278175   0.902835  -1.407794
H   2.003799  -0.224335  -2.388464
H   3.184001   1.727929   1.112172
C  -1.324762  -1.609157  -0.556383
C  -2.407639  -0.648386   0.028167
O  -0.945054  -2.517730   0.201898
O  -0.935512  -1.365232  -1.733686
H  -3.368341  -1.166519   0.000219
C  -2.494970   0.682784  -0.705909
N  -2.059682  -0.432273   1.473308
H  -1.955756  -1.336436   1.937150
H  -2.722315   0.490687  -1.751004
H  -1.536130   1.197741  -0.653511
S  -3.735272   1.854502  -0.000522
H  -1.120917   0.050684   1.492273
H  -2.763240   0.131127   1.953604
H  -4.831135   1.126607  -0.298498
28
Dimer 6 of the z...z  type
C   1.062653   1.632276   0.421462
C   2.430553   0.902083   0.548807
O   0.318522   1.543840   1.443925
O   0.833920   2.228814  -0.639978
H   3.131044   1.611889   0.998410
C   3.019298   0.426237  -0.773198
N   2.272946  -0.224933   1.530937
H   3.146062  -0.732091   1.675699
H   3.982582  -0.061273  -0.612270
H   3.179192   1.300117  -1.401101
S   1.986422  -0.795628  -1.682852
H   1.960192   0.156492   2.425387
H   1.515068  -0.902500   1.244471
H   0.975471   0.054607  -1.945973
C  -0.751345  -1.932098   0.322019
C  -1.911615  -0.898203   0.419214
O   0.145749  -1.842055   1.210484
O  -0.841826  -2.738569  -0.612927
H  -2.849108  -1.452430   0.477211
C  -1.921520   0.026452  -0.796201
N  -1.785434  -0.096910   1.681286
H  -1.631837  -0.714301   2.478324
```

```
H  -1.954928  -0.587610  -1.693461
H  -1.021391   0.641168  -0.826480
S  -3.310200   1.239660  -0.810584
H  -0.955974   0.555366   1.620736
H  -2.622927   0.465175   1.843049
H  -4.304643   0.337072  -0.939231
28
Dimer 7 of the z...z  type
C   0.949153  -0.956933   0.512871
C   2.127717  -0.269580  -0.225469
O   0.542804  -0.358739   1.554609
O   0.522115  -2.012979   0.024722
H   2.011615  -0.423326  -1.296879
C   3.454064  -0.849748   0.262058
N   2.055148   1.206680   0.016427
H   1.183834   1.576992  -0.449491
H   3.447979  -1.925180   0.091149
H   3.577154  -0.672927   1.332599
S   4.941573  -0.103957  -0.532999
H   1.956382   1.379596   1.020222
H   2.887937   1.685396  -0.334906
H   4.678271  -0.536267  -1.783135
C  -1.213385   1.499290  -0.108747
C  -2.043828   0.184516  -0.069095
O  -1.454613   2.323919   0.790315
O  -0.376257   1.587373  -1.051168
H  -1.517966  -0.604147  -0.603336
C  -3.431782   0.416226  -0.655194
N  -2.120405  -0.244640   1.365551
H  -1.133006  -0.462338   1.674604
H  -3.329799   0.762828  -1.683000
H  -3.958985   1.184035  -0.085726
S  -4.521598  -1.072164  -0.613970
H  -2.468096   0.533542   1.931945
H  -2.730608  -1.055951   1.485491
H  -3.791554  -1.817803  -1.468251
28
Dimer 8 of the z...z  type
C   1.550327   0.568701   1.115026
C   2.346021  -0.664181   0.606709
O   0.437668   0.782842   0.547322
O   2.099738   1.225797   2.009272
H   2.510894  -1.334664   1.451922
C   3.701239  -0.257349   0.028835
N   1.528950  -1.419278  -0.395977
H   2.026445  -2.236156  -0.752154
H   4.266772  -1.131727  -0.297644
H   4.265141   0.248672   0.809807
S   3.585694   0.837199  -1.453225
H   0.607781  -1.736224   0.018187
H   1.307780  -0.807427  -1.185638
H   3.061774   1.902985  -0.813629
C  -1.846721  -1.843355  -0.135081
C  -2.575954  -0.530064   0.266326
O  -0.858822  -2.174764   0.588780
O  -2.298969  -2.423265  -1.131526
H  -3.613341  -0.769176   0.503222
C  -2.505571   0.484043  -0.875618
N  -1.944486   0.045632   1.498187
H  -1.851451  -0.668933   2.219703
H  -2.930113   0.029073  -1.767254
H  -1.464732   0.745373  -1.074765
S  -3.352132   2.083879  -0.522366
H  -0.973254   0.383677   1.252913
H  -2.488145   0.830935   1.860073
H  -4.606747   1.590202  -0.478734
28
Dimer 9 of the z...z  type
C  -1.127110  -0.853063   0.102293
```



```
C  -2.105324  -0.065985   1.009242
O  -0.901335  -0.336515  -1.033405
O  -0.668456  -1.909223   0.562257
H  -1.765947  -0.154921   2.040902
C  -3.529399  -0.616459   0.908912
N  -2.042393   1.387572   0.650845
H  -2.709616   1.946191   1.183271
H  -4.184017  -0.114628   1.623843
H  -3.500707  -1.676400   1.159912
S  -4.342613  -0.379234  -0.730184
H  -1.058513   1.734043   0.826705
H  -2.238204   1.499596  -0.347322
H  -3.398528  -1.000142  -1.467179
C   1.218406   1.550377  -0.001075
C   1.954415   0.180462  -0.020500
O   1.343568   2.251175  -1.019937
O   0.572979   1.805888   1.055334
H   1.492530  -0.500293   0.691804
C   3.435459   0.375103   0.282983
N   1.745739  -0.413490  -1.380888
H   0.708603  -0.592929  -1.477195
H   3.537761   0.843459   1.261306
H   3.889384   1.033541  -0.460232
S   4.425236  -1.181495   0.240943
H   2.019950   0.268838  -2.092614
H   2.283885  -1.273770  -1.504207
H   3.825314  -1.772699   1.294032
28
Dimer 10 of the z...z  type
C   0.930377  -1.270077  -0.045592
C   2.032710  -0.174851  -0.066355
O   0.467694  -1.564910   1.097746
O   0.610981  -1.734481  -1.148415
H   1.769003   0.552147  -0.834056
C   3.395267  -0.798957  -0.356648
N   2.031105   0.559417   1.240877
H   1.133041   1.105127   1.313163
H   3.329331  -1.343308  -1.296780
H   3.671733  -1.504266   0.429934
S   4.780734   0.415924  -0.433449
H   2.040322  -0.112195   2.011766
H   2.837277   1.184721   1.315906
H   4.336174   1.084254  -1.517274
C  -0.930999   1.271400  -0.042595
C  -2.032685   0.175563  -0.065817
O  -0.464719   1.559951   1.100864
O  -0.615608   1.742253  -1.143822
H  -1.768953  -0.548891  -0.835875
C  -3.395966   0.799444  -0.353167
N  -2.029436  -0.562926   1.239101
H  -1.130416  -1.107182   1.309752
H  -3.330964   1.347220  -1.291362
H  -3.672799   1.501741   0.435989
S  -4.780355  -0.416443  -0.433720
H  -2.040108   0.105961   2.012314
H  -2.834312  -1.190145   1.312049
H  -4.336092  -1.079965  -1.520609
28
Dimer 11 of the z...z  type
C   0.997443  -1.242935  -0.402620
C   2.222384  -0.290620  -0.374129
O   0.096580  -0.992188   0.453641
O   1.009501  -2.150639  -1.247106
H   2.663765  -0.244860  -1.368432
C   3.243574  -0.786300   0.646927
N   1.746545   1.089164  -0.040104
H   1.070759   1.409275  -0.780428
H   3.540057  -1.800995   0.384404
H   2.800632  -0.806263   1.644630
```

```
S   4.743956   0.276324   0.791574
H   1.201700   1.073940   0.826990
H   2.525535   1.743227   0.061028
H   5.212145   0.047257  -0.452336
C  -1.191791   1.498634  -0.639913
C  -2.432622   0.562097  -0.547200
O  -0.424925   1.283362  -1.626181
O  -1.040723   2.346011   0.254142
H  -3.225361   0.981792  -1.170124
C  -2.912930   0.376750   0.887133
N  -2.050050  -0.763970  -1.137744
H  -1.631988  -0.622879  -2.059223
H  -3.141617   1.351702   1.308614
H  -2.120346  -0.075840   1.484759
S  -4.372081  -0.739769   1.056928
H  -1.300587  -1.170449  -0.519683
H  -2.846846  -1.398987  -1.202686
H  -5.253132   0.053721   0.414229
28
Dimer 12 of the z...z  type
C   1.557567   1.792990   0.559417
C   2.573927   0.617165   0.518294
O   0.506386   1.596814   1.242289
O   1.869817   2.794680  -0.098698
H   3.509793   0.953460   0.966492
C   2.802865   0.165085  -0.924039
N   2.060238  -0.531373   1.333950
H   1.763176  -0.209885   2.255100
H   3.123557   1.023880  -1.508397
H   1.871078  -0.217972  -1.345001
S   4.022326  -1.207361  -1.102813
H   1.211643  -0.939913   0.850413
H   2.769321  -1.259451   1.435857
H   5.104445  -0.506395  -0.706213
C  -1.182686  -1.378463   0.553596
C  -2.216206  -0.239723   0.330346
O  -0.090365  -1.264288  -0.082684
O  -1.536174  -2.282131   1.321907
H  -2.555787   0.110176   1.305130
C  -3.400916  -0.750411  -0.487884
N  -1.548016   0.918417  -0.340390
H  -0.795449   1.310962   0.291191
H  -3.823790  -1.614045   0.021603
H  -3.074282  -1.064770  -1.481721
S  -4.716630   0.508399  -0.780691
H  -1.073585   0.598985  -1.187638
H  -2.222975   1.646612  -0.585484
H  -5.088446   0.641964   0.508913
28
Dimer 13 of the z...z  type
C   2.293215   0.611617   1.324520
C   2.957951  -0.456789   0.413265
O   1.177551   0.290665   1.833594
O   2.919077   1.673514   1.441239
H   3.892784  -0.767921   0.885004
C   3.278743   0.087628  -0.976045
N   2.086767  -1.676980   0.336465
H   2.560536  -2.451131  -0.130579
H   3.600643  -0.711188  -1.645808
H   4.094572   0.800452  -0.881392
S   1.875718   0.936184  -1.827677
H   1.838316  -1.976528   1.280818
H   1.162843  -1.524426  -0.149731
H   1.913383   2.047834  -1.063804
C  -1.473083  -1.763793  -0.083843
C  -2.017068  -0.380702   0.376436
O  -0.378205  -1.745479  -0.720464
O  -2.192141  -2.736021   0.185058
H  -2.268691  -0.434321   1.435402
```



```
C  -3.258198  -0.020857  -0.441823
N  -0.956015   0.659296   0.221267
H  -0.183223   0.536598   0.925703
H  -3.990787  -0.816223  -0.322660
H  -3.007056   0.059368  -1.501869
S  -4.014634   1.601444   0.002302
H  -0.499951   0.565333  -0.694157
H  -1.346249   1.601167   0.311411
H  -4.359347   1.268664   1.263066
28
Dimer 14 of the z...z  type
C   1.482743  -1.559222   0.193762
C   2.625679  -0.926219  -0.654555
O   0.435086  -1.833325  -0.469796
O   1.704372  -1.772515   1.390798
H   3.183717  -1.765416  -1.079335
C   3.608855  -0.062754   0.126845
N   2.012550  -0.196702  -1.820549
H   2.718917   0.226199  -2.423402
H   4.509623   0.116201  -0.462470
H   3.885007  -0.625016   1.016867
S   3.010168   1.613819   0.603236
H   1.458302  -0.857885  -2.367268
H   1.332672   0.543931  -1.511476
H   1.756736   1.272501   1.004564
C  -0.643528   0.911483  -0.061999
C  -1.932158   0.062385  -0.234744
O  -0.246469   1.046146   1.115459
O  -0.128807   1.367462  -1.114862
H  -1.971339  -0.339677  -1.245250
C  -3.166886   0.902960   0.073665
N  -1.813888  -1.104792   0.696363
H  -0.967917  -1.659877   0.372659
H  -3.195588   1.755366  -0.604219
H  -3.117639   1.281314   1.096560
S  -4.757726  -0.024319  -0.037029
H  -1.620325  -0.764219   1.641675
H  -2.661874  -1.674929   0.703424
H  -4.701689  -0.263987  -1.362994
28
Dimer 15 of the z...z  type
C  -1.254991   0.873123  -0.334329
C  -1.865395  -0.496214  -0.752325
O  -0.746430   0.924984   0.816413
O  -1.366485   1.779477  -1.179776
H  -1.516205  -0.744142  -1.755426
C  -3.392629  -0.426457  -0.769504
N  -1.364833  -1.563602   0.168266
H  -0.353129  -1.795975  -0.074571
H  -3.820757  -1.364804  -1.127382
H  -3.686610   0.368474  -1.453137
S  -4.165922  -0.146286   0.882404
H  -1.391117  -1.205639   1.126845
H  -1.919334  -2.418031   0.114428
H  -3.569436   1.036472   1.137179
C   2.211296  -1.737066  -0.213836
C   2.151127  -0.320171   0.414569
O   3.336365  -2.256263  -0.317165
O   1.101896  -2.194972  -0.603582
H   1.282167  -0.210983   1.059435
C   2.105337   0.733599  -0.694198
N   3.382351  -0.119452   1.255823
H   3.300854  -0.586089   2.159828
H   1.221673   0.556292  -1.304064
H   2.985717   0.655095  -1.335117
S   2.068573   2.461255  -0.059633
H   4.180540  -0.543175   0.766334
H   3.548276   0.880430   1.420820
H   0.841075   2.327260   0.502487
```

```
28
Dimer 16 of the z...z  type
C  -1.260857   0.898511  -0.252013
C  -1.840057  -0.461292  -0.740612
O  -0.732423   0.900921   0.890416
O  -1.409301   1.846678  -1.044560
H  -1.455323  -0.665585  -1.740735
C  -3.366320  -0.411260  -0.806379
N  -1.355486  -1.559304   0.152877
H  -0.341399  -1.790743  -0.081047
H  -3.769072  -1.341550  -1.211450
H  -3.649396   0.404447  -1.469490
S  -4.194796  -0.200157   0.828478
H  -1.394616  -1.232820   1.122081
H  -1.912239  -2.409306   0.062985
H  -3.667082   1.006938   1.118507
C   2.224295  -1.741044  -0.208582
C   2.161644  -0.319232   0.408370
O   3.350007  -2.259060  -0.310362
O   1.114941  -2.203906  -0.592481
H   1.301813  -0.212166   1.065880
C   2.086333   0.722559  -0.710291
N   3.403872  -0.099945   1.228056
H   3.338118  -0.555138   2.139247
H   1.190216   0.532906  -1.298035
H   2.951630   0.640499  -1.371099
S   2.059729   2.457736  -0.097351
H   4.197283  -0.526088   0.732905
H   3.566621   0.903008   1.377170
H   0.849799   2.332551   0.501457
28
Dimer 17 of the z...z  type
C   1.876896   1.935511   0.158696
C   2.146970   0.459515  -0.236867
O   2.871621   2.657830   0.345639
O   0.659577   2.235051   0.301954
H   1.475368   0.136244  -1.029930
C   1.985715  -0.433506   0.997579
N   3.552983   0.356428  -0.762842
H   3.620379   0.717292  -1.715179
H   0.958450  -0.352041   1.349148
H   2.644596  -0.091994   1.798364
S   2.390021  -2.208168   0.714524
H   4.167620   0.935190  -0.178418
H   3.870749  -0.619518  -0.763510
H   1.210392  -2.515565   0.113225
C  -0.900505  -1.205487  -0.709089
C  -1.822186  -0.144037  -0.037925
O  -0.368518  -0.876063  -1.794290
O  -0.798596  -2.282610  -0.081087
H  -1.553793  -0.052692   1.014377
C  -3.282360  -0.574047  -0.170769
N  -1.581892   1.185955  -0.673895
H  -0.660290   1.592332  -0.328967
H  -3.393242  -1.560293   0.275478
H  -3.566805  -0.638535  -1.223133
S  -4.490170   0.595839   0.588250
H  -1.504926   1.059013  -1.686555
H  -2.337512   1.841600  -0.459733
H  -4.066075   0.448185   1.860141
28
Dimer 18 of the z...z  type
C   0.508626   1.877653  -0.285686
C   1.834015   1.181039   0.168927
O  -0.237381   2.257124   0.653270
O   0.327887   1.985478  -1.513037
H   2.587184   1.964929   0.274803
C   2.320893   0.113527  -0.802254
N   1.605086   0.595715   1.530971
```



```
H   1.083550   1.276332   2.089067
H   2.433960   0.562599  -1.785216
H   1.596557  -0.697683  -0.875696
S   3.896235  -0.701988  -0.291893
H   1.014892  -0.279104   1.432993
H   2.480625   0.350697   1.993805
H   4.682700   0.386518  -0.419225
C  -0.689894  -2.026183   0.340592
C  -1.472623  -0.844593  -0.292795
O  -1.103271  -3.171939   0.094025
O   0.274499  -1.685728   1.078405
H  -0.793245  -0.135086  -0.759526
C  -2.318257  -0.145188   0.773240
N  -2.354755  -1.377171  -1.389755
H  -1.814149  -1.594437  -2.228011
H  -1.647311   0.221211   1.546674
H  -3.025857  -0.845688   1.220832
S  -3.300373   1.271627   0.125242
H  -2.785235  -2.254795  -1.077214
H  -3.076111  -0.690772  -1.639133
H  -2.214118   2.074245  -0.009711
28
Dimer 19 of the z...z  type
C   1.163191   0.684012   0.549316
C   1.855600  -0.696515   0.385935
O   0.670086   1.184458  -0.496059
O   1.194668   1.158459   1.701148
H   1.373163  -1.389205   1.074595
C   3.348464  -0.622843   0.694056
N   1.600562  -1.219542  -0.993670
H   0.551807  -1.364780  -1.139601
H   3.817462  -1.604299   0.602363
H   3.467723  -0.277998   1.719409
S   4.309179   0.479763  -0.434905
H   1.912199  -0.530322  -1.680820
H   2.089098  -2.098593  -1.164404
H   3.711200   1.632061  -0.068855
C  -1.568068  -1.334845  -0.139614
C  -3.034825  -0.825775  -0.139851
O  -1.057553  -1.595246   0.967924
O  -1.049781  -1.446884  -1.280087
H  -3.660366  -1.635134  -0.519951
C  -3.249491   0.424437  -0.993511
N  -3.431600  -0.582494   1.290293
H  -4.415005  -0.774449   1.480349
H  -4.313158   0.646688  -1.086148
H  -2.844794   0.225641  -1.983310
S  -2.482789   1.946148  -0.288723
H  -2.817787  -1.169587   1.874442
H  -3.227984   0.399083   1.527798
H  -1.169578   1.571211  -0.395787
28
Dimer 20 of the z...z  type
C  -1.781512   1.964145  -0.286069
C  -2.149542   0.578089   0.308049
O  -2.728855   2.721287  -0.560893
O  -0.549110   2.155090  -0.477226
H  -1.441757   0.270648   1.074633
C  -2.184651  -0.466439  -0.810025
N  -3.507326   0.684229   0.947891
H  -3.456233   1.128285   1.865448
H  -1.201367  -0.511570  -1.274793
H  -2.913212  -0.185466  -1.573210
S  -2.655582  -2.147548  -0.227054
H  -4.096344   1.280670   0.353148
H  -3.930374  -0.244602   1.060056
H  -1.534408  -2.308105   0.518557
C   0.935279  -1.366576   0.090777
C   1.907720  -0.172698  -0.149858
```

```
O   0.276449  -1.331425   1.163570
O   0.932683  -2.236290  -0.798829
H   1.817105   0.160686  -1.183170
C   3.338606  -0.625189   0.138649
N   1.509753   0.972099   0.723851
H   0.657639   1.455766   0.306049
H   3.569941  -1.472151  -0.504411
H   3.434817  -0.945867   1.178312
S   4.611455   0.692257  -0.081843
H   1.249008   0.604751   1.643510
H   2.269802   1.650415   0.819047
H   4.419690   0.869600  -1.405142
28
Dimer 21 of the z...z  type
C  -0.937372   1.368891   0.103222
C  -1.906610   0.174049  -0.145865
O  -0.273930   1.325528   1.172801
O  -0.941591   2.247535  -0.777603
H  -1.812779  -0.153537  -1.180787
C  -3.339355   0.621764   0.141101
N  -1.508637  -0.974752   0.722576
H  -0.655319  -1.456290   0.304600
H  -3.570287   1.472243  -0.497395
H  -3.439543   0.935799   1.182428
S  -4.608657  -0.696952  -0.091517
H  -1.249692  -0.612073   1.644565
H  -2.268178  -1.654314   0.812947
H  -4.411659  -0.866387  -1.415073
C   1.784906  -1.965084  -0.282405
C   2.151210  -0.576427   0.306586
O   2.732868  -2.722699  -0.553667
O   0.552739  -2.157568  -0.473646
H   1.444855  -0.268771   1.074420
C   2.179632   0.464975  -0.814628
N   3.511190  -0.676648   0.942639
H   3.464093  -1.117570   1.861923
H   1.194327   0.506287  -1.275436
H   2.905466   0.183352  -1.580192
S   2.649991   2.148758  -0.239084
H   4.100240  -1.273484   0.348408
H   3.931803   0.253849   1.050261
H   1.532313   2.310411   0.511292
28
Dimer 22 of the z...z  type
C   1.653368  -1.333730  -0.384011
C   1.652223   0.200955  -0.662148
O   0.533481  -1.791191  -0.000911
O   2.702581  -1.956685  -0.591226
H   1.128795   0.333996  -1.611855
C   3.028487   0.845132  -0.760327
N   0.796190   0.892320   0.362500
H   0.200986   1.598410  -0.093047
H   2.943290   1.895768  -1.042317
H   3.596455   0.321760  -1.526156
S   3.979757   0.826948   0.820000
H   0.142535   0.216724   0.792333
H   1.368694   1.297578   1.108181
H   4.035682  -0.517610   0.912814
C  -2.335905   1.150001  -0.820960
C  -2.818496  -0.328622  -0.748421
O  -1.218498   1.341443  -1.375762
O  -3.101239   1.978570  -0.301332
H  -3.694832  -0.424957  -1.393296
C  -3.211510  -0.746797   0.665943
N  -1.766367  -1.240260  -1.313808
H  -2.142550  -2.165646  -1.523087
H  -3.367466  -1.824847   0.726957
H  -4.144427  -0.251617   0.924101
S  -1.969898  -0.334846   1.972211
```



```
H  -1.405933  -0.831633  -2.178745
H  -0.921014  -1.372012  -0.688566
H  -2.228661   0.989217   1.978898
28
Dimer 23 of the z...z  type
C  -1.186871   0.840402   0.217784
C  -1.775277  -0.228929  -0.746000
O  -0.797177   0.432427   1.336008
O  -1.180290   2.006524  -0.235699
H  -1.285222  -0.136572  -1.716680
C  -3.278512  -0.030332  -0.943153
N  -1.457693  -1.591717  -0.217472
H  -0.415491  -1.791981  -0.312881
H  -3.674615  -0.750396  -1.661575
H  -3.438738   0.972067  -1.336490
S  -4.288337  -0.266599   0.582410
H  -1.692710  -1.624716   0.777451
H  -1.973410  -2.324267  -0.705029
H  -3.764001   0.762679   1.278943
C   2.194200  -1.764796  -0.147853
C   2.138626  -0.306110   0.374136
O   3.299584  -2.333320  -0.119902
O   1.101770  -2.209217  -0.597746
H   1.272488  -0.157746   1.015867
C   2.089481   0.659905  -0.813199
N   3.367381  -0.043155   1.201845
H   3.304596  -0.501116   2.111989
H   1.189519   0.448785  -1.388233
H   2.949869   0.501261  -1.466511
S   2.113057   2.442846  -0.349921
H   4.185398  -0.439606   0.725796
H   3.495468   0.963774   1.353459
H   0.802526   2.509814   0.001893
28
Dimer 24 of the z...z  type
C   1.515773   1.472560  -0.211910
C   2.989456   1.115537   0.122560
O   0.750309   1.621081   0.791585
O   1.230739   1.598475  -1.408907
H   3.585826   2.011630  -0.057262
C   3.540793  -0.036808  -0.716709
N   3.080352   0.808109   1.592763
H   3.980581   1.054597   2.003269
H   4.617802  -0.132996  -0.575109
H   3.342154   0.189118  -1.762187
S   2.834361  -1.682281  -0.276079
H   2.312635   1.310336   2.059182
H   2.915432  -0.199795   1.729374
H   1.533221  -1.441641  -0.626590
C  -0.978200  -1.329254   0.147878
C  -2.030627  -0.196627   0.348589
O  -0.333900  -1.272670  -0.933386
O  -0.916151  -2.181050   1.052905
H  -2.093619   0.059833   1.405218
C  -3.385720  -0.676669  -0.167500
N  -1.570443   1.019726  -0.384588
H  -0.750407   1.447911   0.129262
H  -3.661713  -1.586566   0.363052
H  -3.324849  -0.903927  -1.233853
S  -4.742815   0.564420  -0.010345
H  -1.186519   0.738565  -1.293810
H  -2.321417   1.701903  -0.507148
H  -4.740059   0.622831   1.337144
28
Dimer 25 of the z...z  type
C   1.578701  -1.405293   0.285170
C   3.011601  -0.814758   0.197897
O   1.038814  -1.698522  -0.827263
O   1.109980  -1.557401   1.419701
```

```
H   3.697830  -1.589426   0.544017
C   3.201928   0.444482   1.044824
N   3.333362  -0.548472  -1.248309
H   4.323269  -0.657734  -1.469409
H   4.259034   0.702698   1.115653
H   2.824799   0.231790   2.042926
S   2.371443   1.939289   0.354400
H   2.757157  -1.184067  -1.816055
H   3.046110   0.415325  -1.475834
H   1.073520   1.503349   0.444845
C  -1.213952   0.865409  -0.714735
C  -2.054089  -0.422437  -0.953207
O  -0.705500   0.968508   0.434659
O  -1.176325   1.672722  -1.660652
H  -1.933321  -0.738489  -1.989506
C  -3.539960  -0.161391  -0.695152
N  -1.518163  -1.515196  -0.086789
H  -2.130830  -2.328632  -0.042142
H  -4.135455  -1.037235  -0.960494
H  -3.852852   0.667602  -1.329466
S  -3.978438   0.192591   1.062897
H  -0.554635  -1.787357  -0.435007
H  -1.357781  -1.151934   0.858502
H  -3.069742   1.171028   1.257731
28
Dimer 26 of the z...z  type
C   1.222057  -1.059108   0.607197
C   2.766454  -1.079891   0.743071
O   0.750000  -1.663867  -0.398112
O   0.615681  -0.432982   1.489926
H   3.024280  -1.620389   1.654559
C   3.346364   0.333858   0.805855
N   3.352962  -1.846642  -0.413799
H   4.271680  -2.239743  -0.205067
H   4.421807   0.309500   0.985653
H   2.863236   0.857260   1.628074
S   3.131584   1.287927  -0.759552
H   2.701999  -2.590245  -0.685961
H   3.439882  -1.219969  -1.224646
H   1.780782   1.313603  -0.754001
C  -3.697045  -0.696593   0.092457
C  -2.271928  -0.057202   0.027059
O  -3.994987  -1.469319  -0.855228
O  -4.378922  -0.375098   1.085674
H  -1.692026  -0.414386   0.877744
C  -2.346532   1.464305   0.065473
N  -1.579463  -0.561428  -1.204603
H  -0.691421  -1.069255  -0.945203
H  -2.866718   1.753517   0.975561
H  -2.909631   1.849743  -0.787743
S  -0.700405   2.305842  -0.006039
H  -2.261232  -1.200915  -1.647135
H  -1.338025   0.185294  -1.855723
H  -0.108378   1.540087   0.937418
28
Dimer 27 of the z...z  type
C  -1.338553   0.895831   1.080615
C  -2.798154   0.725330   0.588688
O  -0.710581   1.897028   0.674532
O  -0.927168  -0.006410   1.853484
H  -3.456818   0.942559   1.431609
C  -3.107876  -0.673141   0.053159
N  -3.051126   1.776998  -0.457376
H  -4.023983   2.080171  -0.501933
H  -4.174741  -0.773875  -0.148420
H  -2.826336  -1.395753   0.815785
S  -2.255457  -1.076442  -1.532947
H  -2.427304   2.569709  -0.252636
H  -2.771808   1.410822  -1.377184
```



```
H  -1.032281  -1.421719  -1.036692
C   1.344373  -1.830308   0.239120
C   1.608432  -0.304642   0.087452
O   1.868719  -2.377756   1.233955
O   0.661048  -2.347358  -0.676630
H   0.764404   0.175441  -0.402532
C   2.884010  -0.077415  -0.717545
N   1.706080   0.290259   1.454699
H   0.737033   0.238562   1.872253
H   2.777382  -0.561890  -1.687171
H   3.744606  -0.514883  -0.207471
S   3.308745   1.702553  -0.962552
H   2.351044  -0.265780   2.021055
H   2.005598   1.266432   1.418739
H   2.206386   2.023663  -1.670321
28
Dimer 28 of the z...z  type
C  -0.551869   1.862251  -0.076682
C  -1.900989   1.081988  -0.223398
O  -0.063344   2.275789  -1.152162
O  -0.106033   1.996783   1.085129
H  -2.697712   1.828686  -0.250770
C  -2.166151   0.071083   0.883288
N  -1.880482   0.401978  -1.559938
H  -1.563488   1.074217  -2.262358
H  -2.084004   0.572218   1.843881
H  -1.433101  -0.734537   0.849340
S  -3.800985  -0.776075   0.743857
H  -1.180283  -0.387171  -1.518561
H  -2.796203   0.028139  -1.812127
H  -4.560871   0.319463   0.947999
C   0.834098  -1.992368  -0.554205
C   1.494114  -0.866271   0.287389
O   1.376927  -3.111030  -0.511722
O  -0.170749  -1.640440  -1.227288
H   0.748761  -0.305245   0.846458
C   2.280404   0.080230  -0.620583
N   2.416258  -1.499076   1.294802
H   1.898988  -1.880247   2.087849
H   1.590243   0.512769  -1.341555
H   3.061541  -0.463088  -1.156048
S   3.118537   1.439775   0.298025
H   2.899016  -2.286263   0.845189
H   3.093381  -0.811801   1.646572
H   1.960406   2.003057   0.733461
28
Dimer 29 of the z...z  type
C  -3.427950  -0.779506   0.006225
C  -1.865928  -0.728237  -0.068480
O  -4.018761  -0.863478  -1.098368
O  -3.910849  -0.758243   1.157157
H  -1.508838  -1.748813   0.093836
C  -1.224122   0.182743   0.970897
N  -1.453188  -0.348127  -1.456436
H  -0.485360  -0.685655  -1.684456
H  -0.141330   0.200643   0.867345
H  -1.479947  -0.189338   1.959765
S  -1.755074   1.943264   0.834475
H  -2.169313  -0.705553  -2.097512
H  -1.423399   0.672030  -1.544901
H  -3.029290   1.718438   1.217318
C   1.596724   0.260172  -1.214819
C   3.058289   0.338310  -0.688605
O   0.936672   1.321022  -1.168785
O   1.219636  -0.865598  -1.625830
H   3.706198   0.525912  -1.547886
C   3.533547  -0.912861   0.038763
N   3.139054   1.557237   0.191045
H   4.070006   1.972797   0.225026
```

```
H   4.553486  -0.786813   0.404214
H   3.515118  -1.743495  -0.662638
S   2.507520  -1.342020   1.511570
H   2.451505   2.231605  -0.178444
H   2.847920   1.322821   1.146702
H   1.419905  -1.705388   0.803279
28
Dimer 30 of the z...z  type
C   1.501587   1.673546  -0.055359
C   2.391662   0.652197   0.720067
O   0.477390   2.067182   0.562584
O   1.909268   2.000948  -1.184754
H   3.200206   1.233119   1.173197
C   3.025447  -0.430253  -0.145669
N   1.596806   0.077386   1.855493
H   2.194525  -0.342731   2.566692
H   3.518823  -1.180447   0.474526
H   3.779766   0.037133  -0.774455
S   1.868110  -1.361096  -1.242438
H   1.061954   0.843526   2.274619
H   0.869421  -0.649527   1.571243
H   1.636937  -0.326434  -2.076166
C  -1.160534  -1.843610   0.583062
C  -1.434627  -0.518060  -0.173327
O  -1.826596  -2.835427   0.235046
O  -0.311985  -1.757756   1.510497
H  -0.502597  -0.008784  -0.391341
C  -2.337968   0.395609   0.652037
N  -2.079628  -0.845261  -1.491076
H  -1.387744  -1.168361  -2.168754
H  -1.846011   0.581186   1.604662
H  -3.303196  -0.077004   0.843171
S  -2.684287   2.004542  -0.179168
H  -2.742967  -1.616212  -1.345982
H  -2.554692  -0.022797  -1.879948
H  -1.383845   2.401171  -0.148543
28
Dimer 31 of the z...z  type
C   0.935559   1.188082  -0.398375
C   2.476551   1.324026  -0.551128
O   0.455783   1.636973   0.688226
O   0.334304   0.682530  -1.351214
H   2.659754   2.137509  -1.254948
C   3.142052   0.048380  -1.065170
N   3.051462   1.744136   0.774983
H   3.870640   2.345658   0.689822
H   4.188463   0.231028  -1.312528
H   2.615303  -0.259073  -1.965872
S   3.156164  -1.327143   0.163152
H   2.297455   2.217516   1.293064
H   3.309116   0.900490   1.306771
H   1.802352  -1.467110   0.324411
C  -0.946984  -1.740906   0.164921
C  -2.123879  -0.778671   0.520734
O   0.037395  -1.701242   0.950045
O  -1.127064  -2.474707  -0.823954
H  -2.900893  -1.384628   0.993215
C  -2.690967  -0.062644  -0.698909
N  -1.656861   0.211574   1.545553
H  -1.249262  -0.286501   2.336920
H  -2.980114  -0.812094  -1.431441
H  -1.923761   0.573682  -1.139887
S  -4.125763   1.039436  -0.331507
H  -0.883531   0.829475   1.156628
H  -2.427515   0.799193   1.868777
H  -4.983982   0.070147   0.047723
28
Dimer 32 of the z...z  type
C  -0.890969  -0.466303  -1.536894
```



```
C  -1.613316   0.070743  -0.265072
O  -0.584830   0.383601  -2.400640
O  -0.701891  -1.704973  -1.546104
H  -1.177320  -0.390824   0.619732
C  -3.100778  -0.270830  -0.337113
N  -1.395237   1.545818  -0.161534
H  -0.387679   1.771901   0.098517
H  -3.201141  -1.348746  -0.448641
H  -3.561720   0.209608  -1.202745
S  -4.078963   0.300275   1.118602
H  -1.564452   1.968011  -1.078369
H  -2.022169   1.963808   0.530557
H  -3.463388  -0.481140   2.029528
C   2.216018   1.644521   0.303579
C   2.091641   0.137406  -0.049536
O   3.363264   2.106159   0.419579
O   1.112290   2.229526   0.477711
H   1.386949   0.012011  -0.868102
C   1.653324  -0.647388   1.188031
N   3.422484  -0.367428  -0.535189
H   3.594068  -0.094300  -1.503957
H   0.740274  -0.197823   1.572490
H   2.409234  -0.567290   1.972081
S   1.411309  -2.449078   0.896694
H   4.163816   0.061786   0.031155
H   3.471169  -1.390711  -0.475239
H   0.457906  -2.307573  -0.067021
28
Dimer 33 of the z...z  type
C  -1.229055   1.047289   0.619280
C  -2.768478   1.044872   0.778295
O  -0.788403   1.583512  -0.436753
O  -0.597630   0.484375   1.527718
H  -3.031393   1.530758   1.718237
C  -3.320206  -0.382313   0.768474
N  -3.385766   1.855768  -0.331923
H  -4.374795   2.052097  -0.167160
H  -4.395768  -0.387729   0.949480
H  -2.828929  -0.934937   1.566943
S  -3.087827  -1.260375  -0.838579
H  -2.881218   2.736591  -0.452896
H  -3.297875   1.337501  -1.214613
H  -1.737173  -1.294609  -0.816112
C   3.700146   0.700962   0.064677
C   2.272394   0.065445   0.021524
O   3.981324   1.480425  -0.882615
O   4.399046   0.374601   1.044444
H   1.699858   0.448599   0.866364
C   2.335391  -1.454671   0.099453
N   1.573045   0.543798  -1.216393
H   2.235052   1.207421  -1.652840
H   2.857339  -1.724078   1.014542
H   2.892287  -1.866683  -0.745319
S   0.682879  -2.285206   0.056760
H   1.370245  -0.210448  -1.871758
H   0.662400   1.017749  -0.965404
H   0.102319  -1.492699   0.985281
28
Dimer 34 of the z...z  type
C   2.000785   1.459883  -0.321542
C   2.079794  -0.057126  -0.651224
O   2.969754   2.162363  -0.661765
O   0.939936   1.819979   0.256848
H   1.373269  -0.261030  -1.458162
C   1.732224  -0.951537   0.535420
N   3.462677  -0.349312  -1.167058
H   3.486001  -1.109000  -1.847459
H   1.724996  -2.002049   0.245087
H   0.738099  -0.691213   0.883797
```

```
S   2.915799  -0.814244   1.942593
H   3.821879   0.522449  -1.580656
H   4.085188  -0.584033  -0.384354
H   2.644232   0.475356   2.232342
C  -1.208735  -1.292791  -0.892911
C  -1.947238  -0.227183  -0.021172
O  -0.721167  -0.871220  -1.971302
O  -1.206713  -2.445744  -0.421788
H  -1.635365  -0.325696   1.018160
C  -3.456196  -0.437856  -0.134684
N  -1.555365   1.141273  -0.480540
H  -0.576828   1.386211  -0.160059
H  -3.685897  -1.454016   0.179604
H  -3.781752  -0.315968  -1.170023
S  -4.468162   0.761009   0.836557
H  -1.538260   1.143596  -1.504842
H  -2.208687   1.850761  -0.138578
H  -4.018535   0.389297   2.052654
28
Dimer 35 of the z...z  type
C   1.622609   0.330185  -1.205770
C   3.071486   0.343135  -0.640991
O   0.978400   1.396711  -1.106860
O   1.236870  -0.761251  -1.694930
H   3.747157   0.550761  -1.473579
C   3.487661  -0.956830   0.036204
N   3.163230   1.516656   0.297994
H   4.101131   1.912224   0.364430
H   4.498813  -0.881143   0.438194
H   3.466133  -1.750629  -0.706537
S   2.403891  -1.425231   1.454817
H   2.493706   2.220969  -0.048607
H   2.852987   1.244543   1.237448
H   1.323404  -1.706239   0.698848
C  -3.374359  -0.818867  -0.023711
C  -1.815521  -0.697473  -0.099010
O  -3.963088  -0.894202  -1.130190
O  -3.855415  -0.852888   1.127405
H  -1.413719  -1.706196   0.030922
C  -1.213026   0.207259   0.969014
N  -1.427014  -0.256660  -1.475996
H  -0.457204  -0.570479  -1.730560
H  -0.131471   0.266279   0.873487
H  -1.461870  -0.200549   1.945523
S  -1.802521   1.951173   0.874030
H  -2.147332  -0.604438  -2.118717
H  -1.418337   0.766331  -1.526190
H  -3.076069   1.672100   1.222001
28
Dimer 36 of the z...z  type
C  -1.215820  -0.922499  -0.296173
C  -2.716130  -1.289911  -0.472933
O  -0.674251  -1.383619   0.749671
O  -0.704348  -0.236617  -1.194633
H  -2.758045  -2.162210  -1.128274
C  -3.558289  -0.169274  -1.071408
N  -3.248251  -1.725875   0.866309
H  -4.028633  -2.379613   0.797754
H  -4.594447  -0.485283  -1.199701
H  -3.144794   0.082444  -2.045033
S  -3.616817   1.345414  -0.020575
H  -2.464077  -2.152298   1.379484
H  -3.551590  -0.904939   1.403536
H  -2.314808   1.661750  -0.166444
C   3.789496  -0.612834  -0.462934
C   2.397710  -0.028027  -0.057807
O   4.163521  -1.625432   0.183906
O   4.364166  -0.028100  -1.402839
H   1.686905  -0.275567  -0.847896
```



```
C   2.434155   1.482511   0.132534
N   1.913463  -0.738050   1.171450
H   0.898763  -1.015015   1.067884
H   2.765759   1.933366  -0.799496
H   3.143720   1.758312   0.916107
S   0.811091   2.206460   0.646972
H   2.527647  -1.562665   1.262214
H   2.014790  -0.168974   2.011319
H   0.074498   1.518535  -0.254634
28
Dimer 37 of the z...z  type
C   3.750963  -0.087621   0.142392
C   2.389227   0.377707   0.747724
O   4.124377   0.509288  -0.897968
O   4.296633  -1.032478   0.747949
H   2.536757   0.628999   1.798336
C   1.336627  -0.726279   0.663407
N   1.934303   1.617096   0.030652
H   2.273385   2.454289   0.501279
H   0.407939  -0.393443   1.124977
H   1.709242  -1.591072   1.209046
S   0.897413  -1.238441  -1.053437
H   2.391769   1.588542  -0.890930
H   0.882455   1.686777  -0.076709
H   2.072335  -1.838131  -1.336720
C  -1.554421   1.265858   0.475614
C  -2.974218   0.971690  -0.092677
O  -0.729784   1.706493  -0.379342
O  -1.371525   1.048453   1.680002
H  -3.578457   1.867629   0.064588
C  -3.663024  -0.223762   0.554974
N  -2.838508   0.792613  -1.580969
H  -3.688697   1.019293  -2.097571
H  -4.669366  -0.358275   0.156851
H  -3.730167  -0.036944   1.624100
S  -2.786999  -1.819485   0.261675
H  -2.056063   1.390256  -1.883909
H  -2.577315  -0.179559  -1.786286
H  -1.679542  -1.499117   0.959617
28
Dimer 38 of the z...z  type
C  -1.492786  -1.123750   0.980632
C  -2.387896   0.150905   1.101188
O  -0.337161  -0.996045   1.463716
O  -2.011245  -2.126671   0.455028
H  -2.837252   0.123573   2.098472
C  -3.515203   0.243322   0.081417
N  -1.497114   1.353694   1.061200
H  -2.004185   2.223491   1.220606
H  -4.118884   1.137245   0.250031
H  -4.151095  -0.630397   0.207213
S  -2.969931   0.337882  -1.676173
H  -0.753748   1.263242   1.760730
H  -0.950491   1.417077   0.155874
H  -2.340232  -0.854632  -1.677251
C   1.389921   1.365911  -0.069080
C   2.710714   0.807163  -0.666862
O   1.442479   1.817144   1.096656
O   0.401352   1.323324  -0.840933
H   3.067129   1.536686  -1.396049
C   2.547525  -0.551586  -1.348814
N   3.722969   0.747990   0.443049
H   4.675748   0.949179   0.141150
H   3.446573  -0.810026  -1.909406
H   1.710786  -0.475966  -2.039825
S   2.281457  -1.949465  -0.176381
H   3.409521   1.419523   1.160938
H   3.695261  -0.191609   0.865872
H   1.120063  -1.512254   0.406494
```

```
28
Dimer 39 of the z...z  type
C  -3.338612  -1.222257   0.595688
C  -2.023056  -0.429760   0.326263
O  -3.452598  -2.310360  -0.021436
O  -4.122970  -0.702590   1.418890
H  -1.303561  -0.693269   1.103990
C  -2.176540   1.084287   0.341403
N  -1.416687  -0.882933  -0.965021
H  -0.393300  -0.601777  -1.030869
H  -1.213048   1.562375   0.163551
H  -2.543192   1.385083   1.319503
S  -3.326754   1.725484  -0.953034
H  -1.540739  -1.896062  -1.035954
H  -1.925935  -0.465407  -1.748543
H  -4.424751   1.116249  -0.459475
C   1.499302   0.423352   0.057303
C   2.908879   1.081509   0.080363
O   0.868841   0.420905   1.132259
O   1.134739  -0.020243  -1.062198
H   2.790422   2.112513  -0.260411
C   3.945172   0.379122  -0.788850
N   3.342413   1.144699   1.519672
H   3.990886   1.907302   1.716475
H   4.908601   0.887552  -0.734541
H   3.594625   0.400356  -1.817882
S   4.263616  -1.365815  -0.285041
H   2.485220   1.240694   2.083857
H   3.784097   0.257866   1.789509
H   3.027810  -1.816072  -0.585513
28
Dimer 40 of the z...z  type
C  -2.042228   0.262512  -1.376646
C  -2.451669  -0.882491  -0.398290
O  -0.974249   0.081234  -2.017019
O  -2.822210   1.232798  -1.415011
H  -3.295642  -1.402752  -0.857558
C  -2.890977  -0.372101   0.970375
N  -1.327604  -1.870069  -0.303341
H  -1.656633  -2.828130  -0.197185
H  -3.049364  -1.201846   1.661323
H  -3.832083   0.159424   0.849088
S  -1.679292   0.750999   1.794451
H  -0.767796  -1.788017  -1.163981
H  -0.631179  -1.680903   0.465935
H  -1.770701   1.737408   0.879756
C   1.670651  -1.334124   0.304999
C   2.742634  -0.249616   0.594288
O   1.694499  -1.877874  -0.820809
O   0.862437  -1.529685   1.244849
H   3.362472  -0.599486   1.420021
C   2.107633   1.093109   0.972315
N   3.618576  -0.132480  -0.624558
H   4.614085  -0.081515  -0.413060
H   2.857515   1.775966   1.372641
H   1.354022   0.899284   1.733298
S   1.346388   1.992307  -0.443751
H   3.405152  -0.960584  -1.205422
H   3.343308   0.695744  -1.170537
H   0.456398   1.044844  -0.828556
28
Dimer 41 of the z...z  type
C   1.261246   1.403371  -0.515665
C   2.706544   1.047404  -0.065089
O   0.585821   2.084665   0.285705
O   0.922947   0.952438  -1.636875
H   3.382438   1.784474  -0.503829
C   3.156056  -0.353844  -0.464678
N   2.763220   1.238203   1.427373
```



```
H   3.699601   1.446065   1.775415
H   4.168134  -0.550640  -0.108224
H   3.151534  -0.417015  -1.549921
S   2.099836  -1.701389   0.225027
H   2.113229   2.003254   1.657940
H   2.413244   0.397638   1.901533
H   1.060115  -1.536164  -0.615279
C  -1.509875  -1.994590   0.272041
C  -1.574372  -0.459723  -0.015384
O  -1.195388  -2.708245  -0.717805
O  -1.724664  -2.335477   1.449012
H  -0.674847  -0.029606   0.422466
C  -2.791108   0.225223   0.587096
N  -1.477968  -0.250477  -1.501043
H  -1.336734  -1.199192  -1.889102
H  -2.771137   0.060718   1.661717
H  -3.715557  -0.200750   0.191711
S  -2.861843   2.038146   0.213941
H  -2.318705   0.169975  -1.895589
H  -0.645922   0.348321  -1.726585
H  -1.530734   2.266629   0.339704
28
Dimer 42 of the z...z  type
C   1.165643  -0.871418   0.979944
C   2.587943  -1.168352   0.429751
O   0.266765  -1.685163   0.607325
O   1.037118   0.110501   1.723883
H   3.111964  -1.752377   1.189635
C   3.401783   0.076997   0.099619
N   2.434439  -2.060807  -0.773385
H   3.253476  -2.642591  -0.952274
H   4.397902  -0.195420  -0.251140
H   3.500039   0.669335   1.006181
S   2.658200   1.116618  -1.230059
H   1.601897  -2.644889  -0.613153
H   2.247380  -1.491101  -1.606923
H   1.641507   1.605735  -0.489498
C  -3.118843  -0.782659  -0.713369
C  -2.469266   0.301343   0.209414
O  -3.276909  -1.915503  -0.200956
O  -3.436321  -0.380146  -1.853119
H  -3.261770   0.986519   0.516411
C  -1.354473   1.071516  -0.488383
N  -1.939808  -0.359229   1.446467
H  -2.676688  -0.895908   1.902112
H  -1.728084   1.430637  -1.443299
H  -0.505803   0.416496  -0.682580
S  -0.703609   2.505466   0.483573
H  -1.161035  -1.020821   1.178613
H  -1.537550   0.319527   2.096715
H  -1.621056   3.399696   0.062482
28
Dimer 43 of the z...z  type
C   1.549606   0.106802   1.357526
C   2.803336  -0.419687   0.605922
O   0.851437  -0.784549   1.934589
O   1.348498   1.325150   1.329374
H   3.662225  -0.273670   1.264444
C   3.063333   0.275413  -0.727647
N   2.644098  -1.906228   0.422791
H   3.531583  -2.401766   0.332607
H   4.008658  -0.061736  -1.153976
H   3.124530   1.345281  -0.542657
S   1.776782  -0.055139  -2.009081
H   2.108147  -2.258351   1.227345
H   2.079606  -2.084168  -0.417650
H   0.826503   0.825539  -1.573521
C  -1.252641   1.780710  -0.194314
C  -1.501505   0.268057   0.086639
```

```
O  -1.726304   2.576460   0.646350
O  -0.658412   2.027989  -1.269496
H  -0.639224  -0.319053  -0.221206
C  -2.739606  -0.179325  -0.685763
N  -1.664728   0.077429   1.561244
H  -0.724563  -0.182427   1.962198
H  -2.576448   0.016735  -1.744119
H  -3.618929   0.381049  -0.361748
S  -3.174420  -1.956349  -0.443834
H  -1.988563   0.974582   1.945156
H  -2.326098  -0.669784   1.782838
H  -2.017701  -2.459061  -0.922025
28
Dimer 44 of the z...z  type
C   2.978560  -0.477051  -0.979131
C   1.581523  -0.410139  -0.287938
O   3.609634  -1.549375  -0.818033
O   3.299295   0.534172  -1.639807
H   0.837632  -0.725087  -1.023563
C   1.196376   0.966567   0.237553
N   1.509374  -1.425599   0.812314
H   0.511643  -1.533159   1.136474
H   0.218203   0.934721   0.717328
H   1.159027   1.660984  -0.597923
S   2.358042   1.623849   1.512277
H   1.900486  -2.306766   0.473809
H   2.087400  -1.121667   1.600012
H   3.420839   1.700829   0.684491
C  -1.706962  -1.271155   0.304917
C  -3.113117  -0.610506   0.256272
O  -1.119428  -1.214873   1.425862
O  -1.292332  -1.775808  -0.747758
H  -3.856116  -1.404062   0.360313
C  -3.370664   0.161820  -1.032674
N  -3.255909   0.279438   1.463578
H  -4.226056   0.413156   1.751372
H  -4.356430   0.627644  -1.018745
H  -3.324749  -0.536622  -1.864575
S  -2.162251   1.527906  -1.316913
H  -2.708640  -0.143090   2.225346
H  -2.841423   1.198878   1.272692
H  -1.105956   0.720731  -1.536322
28
Dimer 45 of the z...z  type
C  -1.422196  -0.307533  -0.239643
C  -2.746110  -0.128866  -1.034772
O  -0.960698  -1.487696  -0.222882
O  -0.929094   0.711144   0.266951
H  -2.471458   0.188913  -2.043069
C  -3.701155   0.890235  -0.426043
N  -3.390332  -1.483264  -1.165744
H  -3.981000  -1.572766  -1.993077
H  -4.602247   0.991863  -1.032083
H  -3.192415   1.850587  -0.388960
S  -4.273668   0.434149   1.266402
H  -2.629826  -2.177590  -1.187539
H  -3.958369  -1.679029  -0.332919
H  -3.058152   0.527322   1.844198
C   3.873630  -0.710023   0.243827
C   2.401578  -0.715197  -0.282264
O   4.043732  -1.206308   1.384497
O   4.721023  -0.236838  -0.542063
H   2.298685  -1.593039  -0.925960
C   2.020768   0.520775  -1.088023
N   1.463130  -0.917130   0.865765
H   0.549321  -1.324152   0.544910
H   0.977501   0.475827  -1.396484
H   2.657893   0.570843  -1.967313
S   2.184645   2.094959  -0.139566
```



```
H   1.944608  -1.491362   1.564888
H   1.234036  -0.014179   1.291855
H   3.530559   2.047929  -0.055615
28
Dimer 46 of the z...z  type
C   1.174906  -1.175549  -0.408195
C   2.719096  -1.293504  -0.234960
O   0.759048  -1.016395  -1.571522
O   0.519588  -1.273631   0.659592
H   2.960238  -2.358055  -0.201406
C   3.258102  -0.614478   1.018287
N   3.352968  -0.739161  -1.481101
H   4.257232  -1.158137  -1.698438
H   4.334350  -0.764388   1.111798
H   2.763787  -1.056975   1.879652
S   3.001257   1.211942   1.027202
H   2.683963  -0.895437  -2.249598
H   3.473877   0.276548  -1.382690
H   1.651955   1.174695   0.982725
C  -3.706678  -0.593234   0.130992
C  -2.606139   0.496663   0.365061
O  -3.762692  -1.503181   0.996203
O  -4.425891  -0.417686  -0.872526
H  -3.116314   1.388276   0.734151
C  -1.831564   0.819032  -0.904838
N  -1.692558   0.020689   1.455494
H  -2.246568  -0.623365   2.034598
H  -2.533687   1.162344  -1.660290
H  -1.310678  -0.062807  -1.278533
S  -0.490869   2.066461  -0.677319
H  -0.869572  -0.522345   1.070515
H  -1.323597   0.785647   2.017122
H  -1.280815   3.082411  -0.270520
28
Dimer 47 of the z...z  type
C  -3.574482  -0.255966   0.793611
C  -2.076130  -0.341827   0.373777
O  -4.305003  -1.193632   0.390261
O  -3.877624   0.723729   1.508831
H  -1.524807  -0.830378   1.179962
C  -1.412214   1.001662   0.103609
N  -1.932652  -1.241465  -0.816441
H  -0.912764  -1.474087  -0.975555
H  -0.375738   0.860379  -0.203532
H  -1.435678   1.591415   1.016235
S  -2.207184   1.963100  -1.257277
H  -2.503213  -2.076125  -0.668807
H  -2.297982  -0.771978  -1.648951
H  -3.384470   2.129699  -0.619216
C   1.262463  -1.361168   0.075959
C   2.728410  -0.858122   0.119711
O   0.745224  -1.414115  -1.080181
O   0.728836  -1.588659   1.169112
H   3.312003  -1.456600   0.816317
C   2.732820   0.613413   0.538709
N   3.344235  -1.013181  -1.244692
H   3.606044  -1.982727  -1.428983
H   2.252475   0.688949   1.512280
H   2.163645   1.216144  -0.172352
S   4.407255   1.378565   0.594614
H   2.641896  -0.753103  -1.947716
H   4.181278  -0.427335  -1.342361
H   4.898436   0.595099   1.576785
28
Dimer 48 of the z...z  type
C   1.374079   0.715665  -0.978441
C   1.330288  -0.152758   0.311401
O   1.982489   0.218368  -1.953623
O   0.853109   1.849672  -0.870005
```

```
H   0.366342  -0.032343   0.802121
C   2.421330   0.282263   1.290627
N   1.452356  -1.593972  -0.080392
H   0.487644  -2.015049  -0.076649
H   2.320134  -0.250546   2.237868
H   2.293799   1.347338   1.481467
S   4.148802  -0.060149   0.736036
H   1.844358  -1.595356  -1.032677
H   2.066960  -2.128355   0.533063
H   4.028690   0.480909  -0.494437
C  -1.660962  -1.210881  -0.577973
C  -3.037504  -0.610059  -0.186497
O  -1.193684  -2.079275   0.221958
O  -1.148491  -0.794826  -1.623216
H  -3.795282  -1.111972  -0.791803
C  -3.134033   0.898251  -0.403639
N  -3.317811  -0.972180   1.248613
H  -4.313229  -0.994474   1.472682
H  -4.151329   1.244941  -0.219312
H  -2.876235   1.104559  -1.439762
S  -2.042293   1.887756   0.708628
H  -2.883307  -1.886236   1.430420
H  -2.855538  -0.292847   1.865742
H  -0.876609   1.761649   0.007625
28
Dimer 49 of the z...z  type
C   1.798859  -1.315579   0.375055
C   2.683684  -0.089257   0.750022
O   2.211020  -2.043709  -0.549322
O   0.772494  -1.438005   1.087508
H   3.160613  -0.317233   1.704456
C   1.889769   1.208883   0.881490
N   3.773786   0.025160  -0.280796
H   4.669410   0.327144   0.101366
H   2.506249   2.002121   1.306023
H   1.051017   1.012324   1.545961
S   1.273087   1.873250  -0.722756
H   3.853047  -0.904873  -0.722164
H   3.481082   0.687809  -1.011649
H   0.528789   0.799389  -1.101279
C  -1.858263   0.010465  -1.442285
C  -2.582339  -0.840125  -0.355996
O  -0.790744  -0.476192  -1.895967
O  -2.414382   1.085119  -1.737761
H  -3.444096  -1.314569  -0.833754
C  -3.096385  -0.010213   0.814623
N  -1.673894  -1.949953   0.086110
H  -2.138115  -2.590815   0.730284
H  -3.566786  -0.642820   1.569506
H  -3.846576   0.674657   0.425263
S  -1.811880   0.959847   1.711144
H  -1.373209  -2.474956  -0.736581
H  -0.769880  -1.621037   0.526722
H  -1.421008   1.697918   0.650627
28
Dimer 50 of the z...z  type
C  -1.216786  -1.343003   0.101630
C  -2.580240  -1.043400   0.790522
O  -0.222677  -1.304242   0.889036
O  -1.216056  -1.596136  -1.110742
H  -2.884814  -1.963714   1.294371
C  -3.695317  -0.593529  -0.142627
N  -2.316646  -0.034358   1.877657
H  -3.036374  -0.029768   2.601835
H  -4.635702  -0.490100   0.400089
H  -3.816373  -1.356624  -0.908439
S  -3.396309   1.038238  -0.943658
H  -1.401604  -0.257951   2.290206
H  -2.259091   0.908190   1.471662
```



```
H  -2.265860   0.666551  -1.578758
C   3.930030  -0.204860  -0.025388
C   2.534981   0.326320  -0.502826
O   4.172843  -1.409312  -0.288363
O   4.655998   0.639267   0.536762
H   2.714199   0.957464  -1.374766
C   1.815264   1.117956   0.581870
N   1.708058  -0.843023  -0.948055
H   2.367008  -1.589167  -1.203551
H   2.466009   1.917442   0.925494
H   1.578227   0.473189   1.429007
S   0.193205   1.816419   0.047838
H   1.084138  -1.184293  -0.170455
H   1.075384  -0.631687  -1.719134
H   0.676179   2.854113  -0.664289
28
Dimer 51 of the z...z  type
C   1.089383  -0.523344   0.046518
C   2.117191   0.521957  -0.476405
O   0.541671  -0.222711   1.149541
O   0.871995  -1.494219  -0.686318
H   1.603561   1.107813  -1.241488
C   3.375752  -0.094724  -1.072910
N   2.436028   1.468399   0.649518
H   2.695339   2.402449   0.331010
H   4.048545   0.675473  -1.451930
H   3.076609  -0.736851  -1.898175
S   4.358957  -1.072095   0.143006
H   1.600705   1.508707   1.251761
H   3.200751   1.088342   1.219100
H   3.426698  -2.027945   0.336556
C  -3.583083  -1.039413  -0.258820
C  -2.845715   0.231223   0.290893
O  -3.577619  -2.040403   0.501987
O  -4.112170  -0.904426  -1.378695
H  -3.612161   0.960906   0.553595
C  -1.891831   0.820211  -0.739494
N  -2.134555  -0.156898   1.557828
H  -2.252775   0.529719   2.299632
H  -2.457665   1.059217  -1.635994
H  -1.130697   0.089192  -1.012733
S  -0.986775   2.328447  -0.171155
H  -2.557007  -1.060383   1.828537
H  -1.100747  -0.298204   1.411246
H  -1.967569   3.218042  -0.422977
28
Dimer 52 of the z...z  type
C  -3.825513  -0.715135   0.074962
C  -2.454783  -0.419215   0.765971
O  -3.866091  -1.746673  -0.641435
O  -4.731837   0.105080   0.324223
H  -2.571904  -0.666396   1.823318
C  -2.023789   1.039021   0.658054
N  -1.422585  -1.352316   0.201369
H  -0.853160  -1.771454   0.936281
H  -1.018952   1.168933   1.058474
H  -2.714966   1.646324   1.237802
S  -1.984230   1.685820  -1.070231
H  -1.946660  -2.063137  -0.330170
H  -0.723196  -0.862230  -0.404721
H  -3.324476   1.697108  -1.226740
C   1.364284   0.042895   0.422985
C   2.677678   0.874322   0.464014
O   0.967416  -0.419714   1.517752
O   0.815992  -0.048029  -0.700285
H   2.388130   1.922786   0.562532
C   3.563605   0.708143  -0.763954
N   3.403972   0.502672   1.728019
H   3.995663   1.250772   2.090205
```

```
H   4.464027   1.317979  -0.681832
H   2.999152   1.031152  -1.635419
S   4.143479  -1.023931  -1.016585
H   2.683753   0.241524   2.417851
H   3.984726  -0.328196   1.564412
H   2.918333  -1.536800  -1.254202
28
Dimer 53 of the z...z  type
C   1.630112  -1.221826   0.751690
C   2.051274  -0.876967  -0.705912
O   0.644864  -0.601825   1.225695
O   2.356144  -2.068335   1.307175
H   2.084454  -1.802851  -1.281818
C   3.431993  -0.221451  -0.741145
N   1.025545   0.000805  -1.354536
H   1.255607   0.190351  -2.330676
H   3.746903  -0.024471  -1.767543
H   4.145087  -0.903524  -0.282799
S   3.503520   1.415419   0.111241
H   0.072387  -0.458903  -1.312182
H   0.978979   0.897322  -0.862144
H   3.226477   0.957068   1.349282
C  -2.239302  -1.169004  -0.432380
C  -3.059412   0.154897  -0.542904
O  -2.636581  -1.988906   0.414902
O  -1.301590  -1.288627  -1.262008
H  -3.741286   0.018493  -1.384706
C  -2.231372   1.414220  -0.776636
N  -3.905212   0.267652   0.694550
H  -4.810473   0.708563   0.532792
H  -2.879261   2.262911  -0.999699
H  -1.595658   1.241583  -1.642858
S  -1.227188   1.940732   0.678130
H  -4.022081  -0.695233   1.049012
H  -3.398177   0.806774   1.408664
H  -0.492931   0.796056   0.838281
28
Dimer 54 of the z...z  type
C   3.579267   1.195762   0.569272
C   2.233245   0.465229   0.274541
O   3.787219   2.239547  -0.098285
O   4.291261   0.679698   1.457750
H   1.491254   0.822442   0.991792
C   2.289672  -1.050655   0.396205
N   1.727710   0.868131  -1.075088
H   0.682770   0.687187  -1.166937
H   1.310213  -1.480271   0.186143
H   2.574841  -1.305565   1.413783
S   3.473906  -1.844997  -0.776469
H   1.956089   1.856817  -1.211666
H   2.224827   0.343777  -1.799885
H   4.575526  -1.265972  -0.255075
C  -1.341953  -0.117419  -0.121550
C  -2.845957  -0.501417  -0.140029
O  -0.745993  -0.224058   0.966919
O  -0.900510   0.306092  -1.221480
H  -3.068726  -1.117567  -1.008764
C  -3.694675   0.770695  -0.149654
N  -3.135911  -1.316615   1.090791
H  -2.869687  -2.294097   0.965389
H  -3.443412   1.337935  -1.043582
H  -3.467938   1.386505   0.722935
S  -5.510671   0.470117  -0.077133
H  -2.558864  -0.943814   1.856710
H  -4.131946  -1.283915   1.335077
H  -5.602731  -0.189383  -1.250143
28
Dimer 55 of the z...z  type
C  -1.583561   1.335964   0.284714
```



```
C  -1.388380  -0.131211  -0.214867
O  -1.045963   1.592065   1.392577
O  -2.200398   2.098489  -0.483764
H  -0.456908  -0.135961  -0.787373
C  -2.505943  -0.661874  -1.100304
N  -1.145451  -1.022599   0.963375
H  -0.469175  -1.773168   0.716043
H  -2.291541  -1.679228  -1.431413
H  -2.589308  -0.017443  -1.972023
S  -4.142704  -0.743798  -0.251365
H  -0.701655  -0.446175   1.691849
H  -2.023495  -1.398361   1.328434
H  -4.241677   0.586310  -0.047620
C   1.869966  -1.239084   0.672782
C   3.118974  -0.702418  -0.081787
O   1.180863  -2.095684   0.045002
O   1.659880  -0.767853   1.801204
H   3.988432  -1.255266   0.279478
C   3.347268   0.792357   0.113289
N   2.957238  -1.048205  -1.539132
H   3.844487  -1.159778  -2.030452
H   4.223870   1.127338  -0.442713
H   3.514426   0.974384   1.171997
S   1.937477   1.832985  -0.465315
H   2.404541  -1.916725  -1.581382
H   2.409146  -0.321019  -2.012392
H   1.084978   1.505186   0.531645
28
Dimer 56 of the z...z  type
C  -2.486332  -1.418852   0.099031
C  -2.797870  -0.181698  -0.802872
O  -3.474174  -1.999570   0.585737
O  -1.270900  -1.709207   0.232009
H  -2.775946  -0.525232  -1.839205
C  -1.840332   0.991696  -0.644979
N  -4.215765   0.230605  -0.508256
H  -4.693644   0.647056  -1.307510
H  -2.160155   1.832790  -1.262174
H  -0.850015   0.707544  -0.985279
S  -1.749703   1.653884   1.072010
H  -4.703812  -0.622104  -0.191074
H  -4.233351   0.898181   0.270894
H  -1.361599   0.499431   1.651341
C   1.820900   1.235113  -0.342042
C   1.615198  -0.173232   0.306729
O   1.428733   1.341394  -1.533380
O   2.341942   2.091506   0.394937
H   0.801970  -0.081674   1.028086
C   2.850832  -0.681429   1.037989
N   1.138783  -1.117771  -0.758507
H   0.171237  -1.449843  -0.522011
H   2.660641  -1.651720   1.499986
H   3.100381   0.034753   1.817558
S   4.317782  -0.929497  -0.053358
H   1.109096  -0.547881  -1.621421
H   1.768066  -1.906536  -0.905435
H   4.437562   0.362636  -0.422397
28
Dimer 57 of the z...z  type
C  -1.457499   1.476152   0.184829
C  -1.989064   0.241678   0.977436
O  -2.051142   2.550780   0.390860
O  -0.467113   1.269811  -0.560750
H  -1.388037   0.164461   1.886411
C  -1.942370  -1.086267   0.236364
N  -3.392060   0.573998   1.413007
H  -3.675119   0.095523   2.268491
H  -2.409247  -1.871253   0.833964
H  -0.909082  -1.381075   0.073256
```

```
S  -2.853178  -1.076490  -1.366185
H  -3.427056   1.597057   1.537501
H  -4.055949   0.335770   0.667962
H  -2.065983  -0.159610  -1.965678
C   1.847361  -1.931439   0.562869
C   2.323837  -0.688786  -0.260875
O   0.956260  -2.632839   0.019623
O   2.425422  -2.102338   1.653270
H   3.296211  -0.942589  -0.686920
C   2.432650   0.570390   0.589981
N   1.375340  -0.476273  -1.406076
H   1.021153  -1.396410  -1.687516
H   3.106643   0.373165   1.419318
H   1.456205   0.843036   0.990679
S   2.998169   2.059097  -0.342649
H   0.559952   0.113532  -1.093868
H   1.818988  -0.004401  -2.192866
H   4.271602   1.653308  -0.525561
28
Dimer 58 of the z...z  type
C   1.248306  -0.025310   0.624098
C   2.395968   0.725102  -0.104463
O   1.103896   0.221600   1.839561
O   0.540938  -0.767488  -0.103958
H   1.953275   1.633062  -0.520608
C   3.066204  -0.067435  -1.217905
N   3.382554   1.163836   0.943133
H   3.911147   1.996146   0.680017
H   3.845587   0.521909  -1.702346
H   2.307829  -0.319371  -1.955424
S   3.885446  -1.610436  -0.628415
H   2.844168   1.335167   1.804901
H   4.043618   0.403703   1.141858
H   2.746817  -2.218732  -0.236396
C  -3.762803  -0.991539  -0.115529
C  -2.790604   0.168144   0.292471
O  -3.677937  -2.039731   0.570878
O  -4.537852  -0.728407  -1.057080
H  -3.387878   0.905366   0.832383
C  -2.117223   0.815010  -0.911480
N  -1.772195  -0.373407   1.249357
H  -2.214329  -1.135264   1.773831
H  -2.886760   1.127033  -1.612532
H  -1.458617   0.101597  -1.408298
S  -1.029879   2.249005  -0.498976
H  -0.938564  -0.756623   0.732238
H  -1.388955   0.332736   1.878187
H  -2.010346   3.091542  -0.115326
28
Dimer 59 of the z...z  type
C  -3.894020  -0.507369  -0.082529
C  -2.642486  -0.317177   0.831756
O  -3.968634  -1.605991  -0.681476
O  -4.685893   0.459083  -0.101362
H  -2.956249  -0.485440   1.865333
C  -2.020666   1.072465   0.741887
N  -1.627985  -1.375809   0.518431
H  -2.042747  -2.301906   0.615652
H  -1.145526   1.149248   1.386798
H  -2.767252   1.798489   1.053908
S  -1.422045   1.515573  -0.946052
H  -1.310086  -1.293712  -0.452107
H  -0.763689  -1.264745   1.092542
H  -2.641580   1.547432  -1.521724
C   1.282808  -0.402912   0.199494
C   2.233775   0.788868  -0.102265
O   0.779056  -0.409067   1.361131
O   1.065780  -1.193422  -0.731257
H   1.628037   1.554469  -0.592443
```



```
C   3.415014   0.431905  -0.994240
N   2.682945   1.367174   1.212850
H   2.925993   2.356553   1.157472
H   4.034556   1.307561  -1.192380
H   3.024832   0.057115  -1.937553
S   4.536582  -0.828072  -0.248858
H   1.914362   1.220139   1.882462
H   3.496842   0.847752   1.562337
H   3.627890  -1.825057  -0.244710
28
Dimer 60 of the z...z  type
C  -1.102841  -0.841680   0.220059
C  -1.861361   0.509241   0.377216
O  -0.996209  -1.288950  -0.945641
O  -0.709492  -1.344492   1.295278
H  -1.288806   1.153774   1.043264
C  -3.256071   0.291851   0.960311
N  -1.913097   1.201601  -0.951161
H  -2.529730   2.013841  -0.946122
H  -3.760791   1.244278   1.133807
H  -3.145030  -0.216956   1.916759
S  -4.407581  -0.664212  -0.121800
H  -0.927947   1.499859  -1.209646
H  -2.234498   0.536337  -1.658747
H  -3.645793  -1.773008  -0.222722
C   1.284711   1.608509  -0.261805
C   2.584483   0.764154  -0.253298
O   0.920714   2.095342   0.825861
O   0.695738   1.641338  -1.373799
H   3.195721   0.991189  -1.124366
C   2.202352  -0.716342  -0.220134
N   3.378742   1.122953   0.974501
H   3.908136   1.986053   0.843263
H   1.575800  -0.930657  -1.083091
H   1.606060  -0.940776   0.666723
S   3.638066  -1.865663  -0.157737
H   2.714653   1.289185   1.741948
H   4.037151   0.376129   1.222189
H   4.154147  -1.543426  -1.361724
28
Dimer 61 of the z...z  type
C  -3.660277  -1.119843   0.505331
C  -2.258022  -0.483470   0.264676
O  -3.925213  -2.131398  -0.190157
O  -4.357790  -0.570233   1.385711
H  -1.564037  -0.900440   0.997375
C  -2.213042   1.031482   0.406635
N  -1.734881  -0.896464  -1.075706
H  -0.692716  -0.694891  -1.153485
H  -1.200366   1.396177   0.233571
H  -2.515090   1.293456   1.417476
S  -3.298156   1.920871  -0.793983
H  -1.945896  -1.886929  -1.215271
H  -2.226892  -0.380296  -1.809583
H  -4.454530   1.414306  -0.317476
C   1.315271   0.014513  -0.017170
C   2.796694   0.474831   0.011290
O   0.743155  -0.125920   1.079860
O   0.863475  -0.204499  -1.171745
H   2.950260   1.291308  -0.691998
C   3.706427  -0.706952  -0.329707
N   3.103744   0.995655   1.389203
H   2.770148   1.952725   1.509390
H   3.445694  -1.055056  -1.327004
H   3.546047  -1.527880   0.372255
S   5.507973  -0.332310  -0.237564
H   2.598198   0.416885   2.071877
H   4.112591   0.980170   1.576213
H   5.526155   0.582181  -1.228750
```

```
28
Dimer 62 of the z...z  type
C  -3.409144   1.554955  -0.473299
C  -2.501983   0.348762  -0.069069
O  -3.440835   2.511898   0.343439
O  -3.983143   1.447824  -1.575647
H  -1.623498   0.358369  -0.715646
C  -3.217495  -0.987886  -0.213792
N  -1.993637   0.568095   1.324918
H  -0.950907   0.385420   1.376148
H  -3.519458  -1.097871  -1.252494
H  -4.115928  -1.016917   0.406996
S  -2.198024  -2.440002   0.312299
H  -2.217980   1.552290   1.540116
H  -2.464937  -0.025895   2.006203
H  -1.055672  -2.030019  -0.286869
C   1.130712  -0.353869   0.224406
C   2.677978  -0.461437   0.196990
O   0.524564  -0.702403  -0.810556
O   0.664027   0.119187   1.289798
H   3.038274  -0.931990   1.109765
C   3.282659   0.934550   0.036321
N   3.073475  -1.340615  -0.957991
H   2.975448  -2.330040  -0.727462
H   2.955938   1.542149   0.877707
H   2.925711   1.402659  -0.883505
S   5.120669   0.954321  -0.085916
H   2.432206  -1.151389  -1.738759
H   4.045712  -1.169752  -1.237976
H   5.360652   0.469024   1.149550
28
Dimer 63 of the z...z  type
C  -3.618281   0.174954   0.163839
C  -2.283048  -0.360039   0.771521
O  -3.897134  -0.258968  -0.984750
O  -4.222273   1.011988   0.859906
H  -2.429483  -0.588024   1.826578
C  -1.181801   0.695332   0.670160
N  -1.899483  -1.630277   0.071463
H  -0.855958  -1.819156   0.069473
H  -0.265605   0.336489   1.137202
H  -1.527620   1.576640   1.207846
S  -0.723860   1.202680  -1.056461
H  -2.375892  -2.427605   0.490828
H  -2.250553  -1.552366  -0.891306
H  -1.875898   0.836325  -1.660227
C   1.625817  -1.394456   0.508105
C   2.786518  -0.874651  -0.391158
O   0.728479  -2.016426  -0.134881
O   1.683512  -1.161451   1.721755
H   3.468914  -1.712076  -0.557737
C   3.579039   0.293513   0.177549
N   2.191640  -0.545161  -1.736665
H   2.895756  -0.290441  -2.430763
H   4.377356   0.586787  -0.505970
H   4.031548  -0.033247   1.111437
S   2.593594   1.818332   0.491373
H   1.658474  -1.356035  -2.064170
H   1.509133   0.222591  -1.649440
H   1.888853   1.302909   1.517742
28
Dimer 64 of the z...z  type
C   3.594856  -1.124374  -0.063674
C   2.202504  -0.423999  -0.053317
O   3.909121  -1.726765   0.991349
O   4.233688  -1.030761  -1.134806
H   1.478305  -1.113585  -0.493721
C   2.144868   0.887239  -0.827248
N   1.743642  -0.211680   1.356264
```



```
H   0.721713   0.070335   1.349847
H   1.145834   1.322144  -0.763945
H   2.384281   0.687589  -1.868602
S   3.299316   2.176809  -0.182791
H   1.901289  -1.067332   1.890689
H   2.299586   0.526349   1.794734
H   4.425638   1.493011  -0.473477
C  -1.298721  -0.337305   0.203972
C  -2.683163   0.016234  -0.398996
O  -0.851617  -1.478805  -0.017690
O  -0.780863   0.583814   0.888188
H  -2.615530   0.952250  -0.951403
C  -3.726623   0.124255   0.713185
N  -3.068628  -1.058170  -1.379705
H  -2.563212  -0.957291  -2.260732
H  -3.399769   0.895783   1.406945
H  -3.802183  -0.818038   1.259889
S  -5.433324   0.483672   0.118907
H  -2.802854  -1.970956  -0.992971
H  -4.075345  -1.033085  -1.577224
H  -5.163664   1.701928  -0.393645
28
Dimer 65 of the z...z  type
C  -2.144390  -1.569320  -0.011624
C  -2.791625  -0.345058  -0.730892
O  -2.934225  -2.377990   0.508444
O  -0.888353  -1.624435  -0.043203
H  -2.860817  -0.592795  -1.792462
C  -2.043801   0.971825  -0.568991
N  -4.203498  -0.230037  -0.219066
H  -4.841121   0.209251  -0.883565
H  -2.597481   1.786971  -1.037831
H  -1.076710   0.915739  -1.060215
S  -1.809074   1.477570   1.186709
H  -4.515000  -1.184994   0.011340
H  -4.215996   0.307841   0.654865
H  -1.007678   0.445351   1.519789
C   1.536807   1.732036  -0.266633
C   1.880257   0.226026  -0.012546
O   1.018747   1.990533  -1.382669
O   1.846046   2.503753   0.659857
H   1.402280  -0.096667   0.912182
C   3.394550   0.057012   0.088306
N   1.306256  -0.594509  -1.126236
H   0.364036  -0.970837  -0.839516
H   3.757465   0.693150   0.892166
H   3.875501   0.368884  -0.841502
S   3.951360  -1.681421   0.358873
H   1.188207   0.039601  -1.927717
H   1.903267  -1.387794  -1.369429
H   3.299657  -1.870275   1.524548
28
Dimer 66 of the z...z  type
C   3.720036  -0.076522   0.226714
C   2.418656  -0.876357  -0.087182
O   3.828883   0.362812   1.395517
O   4.497593   0.045754  -0.743233
H   2.692133  -1.896507  -0.362718
C   1.625538  -0.265993  -1.239953
N   1.563969  -0.970301   1.143428
H   0.619500  -1.371635   0.907549
H   0.742591  -0.867771  -1.458502
H   2.267802  -0.236300  -2.117035
S   0.986378   1.430627  -0.894701
H   2.024200  -1.537215   1.854153
H   1.461184  -0.036341   1.546418
H   2.183331   2.034389  -0.748448
C  -1.932714  -1.801801   0.006064
C  -2.236520  -0.314653  -0.346248
```

```
O  -2.902799  -2.578412   0.020436
O  -0.726336  -2.048729   0.263874
H  -1.560123   0.034010  -1.123875
C  -2.110328   0.559797   0.901958
N  -3.630385  -0.234919  -0.906142
H  -3.653818  -0.521447  -1.885580
H  -1.087296   0.495043   1.263996
H  -2.775947   0.201483   1.689507
S  -2.558467   2.326254   0.641815
H  -4.223700  -0.896657  -0.390729
H  -4.004033   0.718771  -0.837835
H  -1.554217   2.608394  -0.212599
28
Dimer 67 of the z...z  type
C   3.811210  -0.244423   0.162187
C   2.435067   0.084565   0.823801
O   4.422857   0.732438  -0.331943
O   4.157217  -1.443672   0.222555
H   2.610038   0.195972   1.897452
C   1.370238  -0.986273   0.619906
N   1.943468   1.412793   0.336832
H   1.098384   1.724733   0.870443
H   0.431325  -0.681287   1.080726
H   1.710637  -1.906577   1.087318
S   0.990279  -1.328718  -1.153506
H   2.707363   2.089906   0.363995
H   1.625326   1.344240  -0.635668
H   2.171433  -1.924573  -1.417702
C  -1.154552   1.509951   0.148017
C  -2.573641   0.881681   0.162976
O  -0.666680   1.767829  -0.972868
O  -0.612894   1.622089   1.276196
H  -3.197033   1.369249   0.909808
C  -2.453123  -0.615798   0.455356
N  -3.202543   1.105345  -1.185057
H  -3.617450   2.035492  -1.255498
H  -1.990497  -0.731708   1.433213
H  -1.818182  -1.102318  -0.287878
S  -4.047137  -1.535256   0.400100
H  -2.455615   1.054284  -1.891345
H  -3.933972   0.411625  -1.376990
H  -4.633675  -0.890121   1.429215
28
Dimer 68 of the z...z  type
C   2.687178  -1.356502  -0.087673
C   1.999929   0.007510  -0.391586
O   2.593621  -1.778178   1.088862
O   3.278891  -1.865553  -1.065017
H   1.235741  -0.141171  -1.155993
C   2.994211   1.051099  -0.891049
N   1.271229   0.482227   0.825619
H   0.844734   1.396158   0.668035
H   2.492726   1.993365  -1.119819
H   3.456044   0.672925  -1.800654
S   4.313305   1.484316   0.326679
H   0.475402  -0.179748   1.008971
H   1.901481   0.510889   1.627939
H   4.901171   0.270458   0.348296
C  -1.556941  -0.288090  -0.184881
C  -2.951275  -0.796851  -0.649618
O  -0.999726  -0.987632   0.711220
O  -1.118328   0.730768  -0.742166
H  -2.793228  -1.411813  -1.538480
C  -3.936094   0.319178  -0.973893
N  -3.489233  -1.710071   0.419792
H  -4.169278  -2.386234   0.070497
H  -4.890393  -0.088374  -1.309435
H  -3.512013   0.924130  -1.771646
S  -4.322448   1.395858   0.473158
```



```
H  -2.686042  -2.192446   0.844720
H  -3.933203  -1.151029   1.158030
H  -3.075647   1.895713   0.598581
28
Dimer 69 of the z...z  type
C   4.037869   0.451727  -0.498397
C   2.854915  -0.230296   0.276548
O   4.243970   1.657337  -0.214538
O   4.662611  -0.291958  -1.280250
H   3.306261  -0.843954   1.058493
C   1.985300  -1.088789  -0.631680
N   2.054148   0.839421   0.954573
H   1.560348   0.516881   1.787288
H   2.631749  -1.768896  -1.179612
H   1.443624  -0.466389  -1.345104
S   0.674312  -2.062009   0.231785
H   2.700054   1.604072   1.180070
H   1.304656   1.213821   0.320823
H   1.489001  -2.798544   1.013244
C  -0.957746   0.857230   0.451716
C  -2.185789   0.026310   0.001573
O  -0.818838   1.032448   1.680135
O  -0.220518   1.268833  -0.479868
H  -1.904779  -0.638789  -0.812305
C  -3.320102   0.956223  -0.423619
N  -2.608314  -0.831252   1.161128
H  -1.958909  -1.611153   1.285629
H  -2.962457   1.577145  -1.243126
H  -3.606706   1.610190   0.402295
S  -4.867071   0.086193  -0.920938
H  -2.557669  -0.265002   2.017037
H  -3.554825  -1.203593   1.030984
H  -4.351516  -0.537633  -1.999866
28
Dimer 70 of the z...z  type
C   0.690865  -1.200923   0.706090
C   2.213090  -1.017186   0.472956
O   0.065499  -1.877761  -0.131290
O   0.239915  -0.555846   1.687629
H   2.766284  -1.187703   1.394454
C   2.456493   0.400360  -0.050523
N   2.668232  -2.036899  -0.534332
H   2.823621  -2.947904  -0.100789
H   2.104807   1.101763   0.702507
H   1.887698   0.574215  -0.966516
S   4.206384   0.764013  -0.489923
H   1.916630  -2.158976  -1.225415
H   3.538219  -1.744465  -0.993575
H   4.707439   0.631362   0.755325
C  -3.321340  -0.640606  -0.622945
C  -2.646686   0.606024   0.044975
O  -3.744967  -1.515302   0.173841
O  -3.399843  -0.607888  -1.867178
H  -3.402993   1.393016   0.076439
C  -1.425300   1.093847  -0.722117
N  -2.316144   0.254657   1.465422
H  -2.987920  -0.475859   1.736215
H  -1.737291   1.376843  -1.723935
H  -0.696163   0.291788  -0.816632
S  -0.536557   2.503000   0.071569
H  -1.335463  -0.145025   1.559339
H  -2.384918   1.056571   2.088993
H  -1.469373   3.443126  -0.185009
28
Dimer 71 of the z...z  type
C   1.525651   0.352699   1.133997
C   2.436294   0.637853  -0.098199
O   1.923886   0.802872   2.223270
O   0.466381  -0.275610   0.876591
```

```
H   2.129528   1.610098  -0.489767
C   2.359426  -0.395293  -1.212591
N   3.841975   0.804211   0.410982
H   4.428243   1.373854  -0.200129
H   3.017091  -0.124834  -2.039478
H   1.336735  -0.423472  -1.580246
S   2.869790  -2.086261  -0.686120
H   3.775147   1.234521   1.344096
H   4.284439  -0.115259   0.523350
H   1.848808  -2.258049   0.178713
C  -3.586985  -0.890729   0.162607
C  -2.740120   0.422277   0.045532
O  -3.683223  -1.372674   1.319587
O  -4.095461  -1.293484  -0.902005
H  -3.444577   1.246679  -0.075858
C  -1.771609   0.383916  -1.129481
N  -2.021124   0.635344   1.348259
H  -2.556501   0.106290   2.051209
H  -2.338617   0.227120  -2.043232
H  -1.063958  -0.435523  -1.010345
S  -0.726148   1.895800  -1.293156
H  -1.041654   0.246503   1.310272
H  -1.958344   1.618690   1.603939
H  -1.689182   2.710702  -1.769541
28
Dimer 72 of the z...z  type
C  -1.309959   1.466355   0.178024
C  -1.826626   0.211348   0.947979
O  -1.796660   2.558099   0.525305
O  -0.436959   1.251789  -0.700883
H  -1.111363   0.006688   1.748323
C  -2.002622  -1.042734   0.102260
N  -3.119537   0.606442   1.611570
H  -3.309921   0.087702   2.469109
H  -2.443937  -1.844475   0.696958
H  -1.030739  -1.393531  -0.232654
S  -3.126667  -0.809637  -1.339540
H  -3.055840   1.618163   1.801293
H  -3.902954   0.469331   0.963548
H  -2.337789   0.078382  -1.979127
C   1.688361  -1.974459   0.548439
C   2.285896  -0.773157  -0.258619
O   0.833957  -2.663714  -0.064465
O   2.147841  -2.131641   1.695805
H   3.263571  -1.091642  -0.625635
C   2.425445   0.490534   0.580646
N   1.420133  -0.525697  -1.460404
H   1.058526  -1.433447  -1.770436
H   3.047296   0.267796   1.443488
H   1.449810   0.823507   0.933949
S   3.115429   1.933976  -0.339499
H   0.603257   0.086309  -1.196207
H   1.928864  -0.064471  -2.213460
H   4.368454   1.451479  -0.471151
28
Dimer 73 of the z...z  type
C  -1.914779  -1.355391   0.492624
C  -2.915697  -0.878034  -0.600310
O  -2.364069  -1.508182   1.643496
O  -0.746131  -1.539671   0.066973
H  -3.133163  -1.730936  -1.245381
C  -2.372282   0.269850  -1.447871
N  -4.200150  -0.497705   0.085258
H  -5.028862  -0.655294  -0.488240
H  -3.066422   0.529876  -2.247903
H  -1.430366  -0.052813  -1.885336
S  -2.115942   1.829615  -0.496750
H  -4.249229  -1.042433   0.958163
H  -4.167792   0.493810   0.353690
```



```
H  -1.211672   1.337666   0.373910
C   3.114318  -1.206739  -0.638717
C   2.084197  -0.170953  -0.079941
O   3.467210  -2.111555   0.158597
O   3.489480  -0.993497  -1.808596
H   1.262599  -0.068211  -0.787267
C   2.774671   1.172707   0.135502
N   1.508313  -0.713946   1.192035
H   0.598993  -1.194989   0.959007
H   3.224830   1.483318  -0.805010
H   3.566481   1.084408   0.882600
S   1.661343   2.506061   0.760738
H   2.187581  -1.399796   1.550317
H   1.317399   0.014221   1.882337
H   0.851582   2.546489  -0.317235
28
Dimer 74 of the z...z  type
C   1.209018   1.621828   0.015778
C   2.362037   0.898029   0.782738
O   0.134029   1.709244   0.667682
O   1.486854   2.088055  -1.102201
H   2.874590   1.680699   1.349445
C   3.405868   0.204454  -0.085698
N   1.750124  -0.029318   1.795544
H   2.454897  -0.563788   2.303191
H   4.304514  -0.009366   0.496003
H   3.666039   0.899108  -0.882216
S   2.901698  -1.415746  -0.807681
H   1.202998   0.521466   2.457767
H   1.053576  -0.691613   1.350651
H   1.620697  -1.080223  -1.136518
C  -0.853242  -1.179557  -0.230849
C  -2.404321  -1.265605  -0.290394
O  -0.334974  -1.477549   0.884224
O  -0.287611  -0.863569  -1.287540
H  -2.650531  -2.201719  -0.795093
C  -3.051850  -0.100637  -1.036210
N  -2.921251  -1.370747   1.118353
H  -3.770982  -1.928628   1.200566
H  -4.113472  -0.290345  -1.198790
H  -2.557723  -0.011520  -2.001203
S  -2.965065   1.501079  -0.126723
H  -2.159915  -1.771581   1.685725
H  -3.110710  -0.422359   1.473486
H  -1.600222   1.577237   0.009576
28
Dimer 75 of the z...z  type
C  -1.464775  -0.732398   0.233866
C  -3.012247  -0.899531   0.212713
O  -0.955152  -0.684283   1.395267
O  -0.890359  -0.680421  -0.859244
H  -3.220910  -1.967622   0.120956
C  -3.697018  -0.151079  -0.923902
N  -3.541753  -0.468305   1.554473
H  -4.430008  -0.907274   1.798580
H  -4.775707  -0.311296  -0.903726
H  -3.302907  -0.528573  -1.864420
S  -3.453421   1.676192  -0.848498
H  -2.820283  -0.698582   2.251756
H  -3.663816   0.551174   1.566739
H  -2.121100   1.637690  -1.057498
C   2.427708  -1.592311  -0.646262
C   2.146440  -0.159277  -0.096401
O   2.504199  -2.507995   0.210755
O   2.581565  -1.657900  -1.883483
H   1.324499   0.271115  -0.664359
C   3.408413   0.690376  -0.218434
N   1.697619  -0.265736   1.325750
H   0.652532  -0.459914   1.371716
```

```
H   3.717276   0.701081  -1.261620
H   4.220284   0.266557   0.377117
S   3.218233   2.423149   0.392392
H   2.183797  -1.065741   1.744673
H   1.891624   0.590544   1.849400
H   2.243528   2.779774  -0.469025
28
Dimer 76 of the z...z  type
C  -0.815054   1.417838  -0.067363
C  -1.874087   0.273821  -0.008303
O  -0.502473   1.940125   1.028478
O  -0.413703   1.697487  -1.217395
H  -1.549619  -0.542155  -0.651228
C  -3.230593   0.814041  -0.450555
N  -1.930253  -0.268411   1.386199
H  -1.052433  -0.834134   1.560619
H  -3.125035   1.235679  -1.448377
H  -3.569243   1.604439   0.222763
S  -4.588541  -0.437772  -0.449448
H  -1.923512   0.516485   2.043659
H  -2.760129  -0.846963   1.533176
H  -4.057319  -1.238983  -1.395417
C   0.963781  -1.549335   0.395152
C   2.416880  -1.041015   0.209880
O   0.386855  -1.989767  -0.617215
O   0.502873  -1.384185   1.554848
H   3.047200  -1.369604   1.033825
C   2.389767   0.484201   0.109042
N   2.971620  -1.630462  -1.059460
H   3.281018  -2.593967  -0.925832
H   1.914033   0.882160   1.002707
H   1.783735   0.799969  -0.742538
S   4.042678   1.253261  -0.144385
H   2.214091  -1.650421  -1.754362
H   3.765051  -1.081140  -1.407359
H   4.583835   0.856328   1.025664
28
Dimer 77 of the z...z  type
C   1.634621   1.358613  -0.111092
C   2.923489   0.867576  -0.829795
O   1.772204   2.228976   0.767599
O   0.575748   0.811953  -0.515354
H   2.970183   1.363754  -1.801095
C   2.970339  -0.643642  -1.031767
N   4.101438   1.352083  -0.028969
H   4.940496   1.498039  -0.590916
H   3.883160  -0.940387  -1.549851
H   2.114617  -0.930921  -1.638482
S   2.957542  -1.594373   0.548505
H   3.815220   2.226632   0.433524
H   4.314875   0.676921   0.714985
H   1.720856  -1.212751   0.932646
C  -3.940501   0.714915  -0.221021
C  -2.509498   0.080726  -0.172448
O  -4.088149   1.766460   0.451634
O  -4.763732   0.122089  -0.945767
H  -2.025874   0.301766  -1.126248
C  -2.547294  -1.425371   0.041924
N  -1.694736   0.785818   0.869259
H  -2.190313   1.669161   1.058738
H  -3.097048  -1.868620  -0.784559
H  -3.069825  -1.671017   0.969209
S  -0.880267  -2.216145   0.194601
H  -1.636776   0.258648   1.740048
H  -0.721445   0.942159   0.485911
H  -0.256943  -1.463127  -0.735355
28
Dimer 78 of the z...z  type
C  -1.171545  -0.269341  -0.298230
```



```
C  -2.477377  -0.096220  -1.125253
O  -0.765687  -1.433961  -0.125971
O  -0.646566   0.805861   0.091361
H  -2.182013   0.136429  -2.150491
C  -3.404393   0.996457  -0.606123
N  -3.163204  -1.435051  -1.160923
H  -3.739840  -1.572457  -1.991309
H  -4.301055   1.073938  -1.222176
H  -2.868862   1.941865  -0.645366
S  -3.994345   0.697004   1.115352
H  -2.423721  -2.151009  -1.108748
H  -3.751626  -1.548129  -0.327035
H  -2.778509   0.806559   1.689868
C   2.936998   1.632591  -0.420728
C   2.746606   0.318517   0.408818
O   2.465589   2.673083   0.098082
O   3.574893   1.505786  -1.487279
H   3.677180   0.153646   0.957363
C   2.429567  -0.896886  -0.452999
N   1.664891   0.534230   1.422651
H   1.833733   1.415636   1.909267
H   3.194558  -0.987632  -1.219232
H   1.459806  -0.784903  -0.935379
S   2.307729  -2.477469   0.496094
H   0.720903   0.619415   0.931852
H   1.622558  -0.235779   2.091478
H   3.625970  -2.591247   0.762215
28
Dimer 79 of the z...z  type
C   4.056946  -0.706436  -0.005055
C   2.532982  -0.654430  -0.351070
O   4.350650  -1.312102   1.054830
O   4.814572  -0.163529  -0.836120
H   2.339340  -1.473148  -1.049461
C   2.080349   0.648911  -0.997783
N   1.733794  -0.948326   0.879409
H   0.780485  -1.321312   0.637187
H   1.007216   0.638076  -1.182270
H   2.609334   0.771360  -1.939588
S   2.380364   2.133848   0.055630
H   2.285679  -1.584343   1.463748
H   1.573067  -0.084285   1.405533
H   3.726023   2.067068  -0.019029
C  -1.249618  -0.255861   0.161536
C  -2.672623  -0.016545  -0.405681
O  -0.802290  -1.434726   0.043556
O  -0.702205   0.721967   0.693505
H  -2.671847   0.878509  -1.025369
C  -3.670060   0.127560   0.744047
N  -3.051072  -1.174523  -1.290292
H  -2.571206  -1.125386  -2.190160
H  -3.346430   0.958437   1.367755
H  -3.681979  -0.776115   1.357049
S  -5.414677   0.380102   0.207930
H  -2.748746  -2.046867  -0.842163
H  -4.062624  -1.193974  -1.461845
H  -5.222032   1.576655  -0.384294
28
Dimer 80 of the z...z  type
C  -2.476739   1.763535  -0.321611
C  -2.339628   0.227943  -0.085277
O  -2.581972   2.471969   0.709110
O  -2.502395   2.107722  -1.522356
H  -1.558626  -0.175673  -0.726833
C  -3.673835  -0.453306  -0.378894
N  -1.902332  -0.013401   1.323210
H  -0.871160   0.221618   1.390095
H  -3.963736  -0.219670  -1.401554
H  -4.451720  -0.083765   0.292804
```

```
S   -3.659090  -2.284713  -0.140501
H   -2.421098   0.614763   1.942639
H   -2.043778  -0.988202   1.596739
H   -2.725004  -2.552070  -1.075915
C    1.122311  -0.210268   0.213864
C    2.530866   0.093589  -0.359194
O    0.695307   0.620908   1.070404
O    0.572127  -1.243001  -0.191491
H    2.529952  -0.048963  -1.437948
C    3.558880  -0.825573   0.302701
N    2.865563   1.537096  -0.090109
H    2.415723   2.156632  -0.765337
H    3.277021  -1.855130   0.091051
H    3.557384  -0.684528   1.385382
S    5.297638  -0.522485  -0.225865
H    2.496931   1.785040   0.836755
H    3.878751   1.696502  -0.130717
H    5.124302  -0.853545  -1.521893
28
Dimer 81 of the z...z  type
C    2.166052   0.653868   1.077943
C    2.778707   0.440410  -0.341266
O    2.879134   0.333425   2.048207
O    1.014704   1.150759   1.066774
H    3.075973   1.420660  -0.718370
C    1.815639  -0.204333  -1.331924
N    4.035594  -0.372258  -0.175871
H    4.762481  -0.142175  -0.853027
H    2.274293  -0.294673  -2.317634
H    0.930797   0.424515  -1.419421
S    1.330012  -1.926485  -0.872326
H    4.364063  -0.207241   0.788202
H    3.817070  -1.372900  -0.251179
H    0.827877  -1.631701   0.344469
C   -1.578917   1.654225  -0.872555
C   -1.713719   0.362322  -0.010912
O   -1.912039   2.722136  -0.305649
O   -1.186730   1.464595  -2.043120
H   -0.894910  -0.309435  -0.244615
C   -3.047420  -0.319253  -0.292226
N   -1.577964   0.733889   1.432261
H   -0.563186   0.919203   1.606991
H   -3.116337  -0.518480  -1.360334
H   -3.880830   0.325652  -0.006570
S   -3.293669  -1.890974   0.646553
H   -2.097707   1.603481   1.589481
H   -1.914635  -0.005582   2.053197
H   -2.252438  -2.554130   0.102391
28
Dimer 82 of the z...z  type
C   -2.369028  -1.926355   0.118193
C   -2.016891  -0.405918   0.071153
O   -2.877079  -2.405352  -0.926203
O   -2.128952  -2.486447   1.207419
H   -1.003368  -0.259581   0.436883
C   -2.993055   0.378433   0.944231
N   -2.045065   0.057048  -1.350569
H   -1.071365  -0.032266  -1.750584
H   -2.902453   0.009787   1.963930
H   -4.021807   0.227939   0.609914
S   -2.726121   2.209018   0.906184
H   -2.691196  -0.559078  -1.858337
H   -2.327122   1.037126  -1.422577
H   -1.377430   2.164559   0.949351
C    1.053456   0.684973  -1.063256
C    2.538734   0.466113  -0.679198
O    0.449187   1.608143  -0.478526
O    0.590609  -0.152295  -1.878598
H    3.142510   0.281298  -1.565345
```



```
C    2.623064  -0.714044   0.292239
N    3.046986   1.720862  -0.024764
H    3.318503   2.421297  -0.715930
H    2.238548  -1.596519  -0.215064
H    2.002947  -0.527596   1.171630
S    4.312270  -1.041210   0.947972
H    2.276153   2.122499   0.526041
H    3.858242   1.526048   0.572891
H    4.878216  -1.357411  -0.234880
28
Dimer 83 of the z...z  type
C   -1.211093   0.111643   0.356147
C   -2.276249  -0.806647  -0.303067
O   -0.821233  -0.227778   1.498072
O   -0.807153   1.066231  -0.346874
H   -1.724825  -1.524484  -0.914696
C   -3.290956  -0.074719  -1.170024
N   -2.941493  -1.589334   0.796255
H   -3.302454  -2.492933   0.489491
H   -4.001467  -0.772050  -1.615502
H   -2.751229   0.433473  -1.965497
S   -4.301605   1.152222  -0.234468
H   -2.238165  -1.711803   1.539688
H   -3.714905  -1.043657   1.193877
H   -3.268613   1.966134   0.066701
C    3.690139   0.690824  -0.269058
C    2.719802   0.258825   0.875912
O    3.587666   1.881474  -0.649482
O    4.483140  -0.194604  -0.654423
H    3.287330   0.334842   1.808019
C    2.194705  -1.168622   0.766441
N    1.607080   1.257929   0.984191
H    1.077101   1.147526   1.850903
H    1.429669  -1.354279   1.520390
H    3.029118  -1.847124   0.927550
S    1.401355  -1.590726  -0.843226
H    2.006378   2.196612   0.914382
H    0.882250   1.175451   0.235537
H    2.524860  -1.463064  -1.578059
28
Dimer 84 of the z...z  type
C    1.716185  -0.828620  -0.772107
C    1.797324  -0.227052   0.664219
O    2.374965  -1.865553  -0.971829
O    0.977251  -0.201840  -1.575701
H    0.931695  -0.603353   1.215621
C    1.816223   1.295561   0.704216
N    3.014239  -0.811759   1.328669
H    2.927806  -0.879198   2.342903
H    1.859698   1.659767   1.731285
H    0.904071   1.665943   0.242850
S    3.266969   2.037425  -0.160511
H    3.156430  -1.743726   0.911627
H    3.842817  -0.248458   1.105145
H    2.923005   1.596289  -1.388369
C   -1.433346  -1.654633   0.392386
C   -1.808301  -0.182239   0.032085
O   -1.681228  -2.504332  -0.493212
O   -0.957372  -1.813079   1.537228
H   -1.099030   0.504795   0.488596
C   -3.221140   0.119476   0.522810
N   -1.694759  -0.013329  -1.449432
H   -0.673135  -0.022718  -1.686422
H   -3.260682  -0.051384   1.597392
H   -3.941691  -0.545303   0.042404
S   -3.802007   1.830216   0.146194
H   -2.133419  -0.820779  -1.902833
H   -2.131055   0.854767  -1.769197
H   -2.892863   2.470208   0.909875
```

```
28
Dimer 85 of the z...z  type
C   3.930235  -0.118931   0.357340
C   2.865759  -0.270703  -0.772671
O   3.880194  -0.960915   1.283836
O   4.705927   0.849350   0.206769
H   3.381476  -0.477903  -1.712220
C   2.031520   0.994181  -0.955643
N   1.978310  -1.447776  -0.488386
H   1.094760  -1.420500  -1.047683
H   1.289284   0.862991  -1.742218
H   2.699497   1.810011  -1.220515
S   1.066377   1.473435   0.542983
H   2.475674  -2.324859  -0.631608
H   1.688790  -1.434915   0.494025
H   2.080584   2.027158   1.235242
C  -1.058636  -0.979695  -0.184515
C  -2.216218  -0.013834   0.178864
O  -0.539090  -0.808310  -1.326583
O  -0.759192  -1.823566   0.673275
H  -2.085956   0.338351   1.200321
C  -3.557183  -0.730290   0.021619
N  -2.154958   1.190112  -0.722371
H  -1.423330   1.839482  -0.420601
H  -3.555152  -1.600930   0.674558
H  -3.691244  -1.072567  -1.006536
S  -5.022636   0.327142   0.382202
H  -1.906227   0.878228  -1.668662
H  -3.051319   1.689626  -0.732484
H  -4.735738   0.543399   1.682386
28
Dimer 86 of the z...z  type
C  -4.040284   0.045178  -0.193865
C  -2.665168   0.252080   0.539166
O  -4.458508  -1.138721  -0.226202
O  -4.577053   1.083355  -0.625829
H  -2.890973   0.663983   1.523974
C  -1.744823   1.189104  -0.228218
N  -2.042663  -1.098270   0.742926
H  -1.490600  -1.178222   1.597081
H  -2.291769   2.105623  -0.429441
H  -1.442278   0.738949  -1.173251
S  -0.201578   1.625878   0.713964
H  -2.820045  -1.772960   0.742872
H  -1.380266  -1.338630  -0.042353
H  -0.308169   2.965046   0.627565
C   0.928229  -1.389755  -0.121841
C   2.273516  -0.741694  -0.558755
O   0.007889  -1.325006  -0.991850
O   0.863491  -1.887972   1.011458
H   2.835701  -1.486426  -1.125329
C   3.102917  -0.227720   0.610519
N   1.954540   0.372761  -1.520103
H   1.387459   0.010397  -2.289829
H   3.289916  -1.057083   1.287608
H   2.555414   0.536906   1.162527
S   4.695031   0.557677   0.112336
H   1.383846   1.085337  -1.033826
H   2.804918   0.812997  -1.881983
H   5.275588  -0.560648  -0.368838
28
Dimer 87 of the z...z  type
C   2.036952   1.068770   0.614673
C   1.971671   0.384631  -0.788573
O   3.067844   1.732238   0.836325
O   1.045426   0.908616   1.370575
H   1.500041   1.103659  -1.460384
C   1.210606  -0.929402  -0.868336
N   3.394433   0.223241  -1.255513
```



```
H   3.489504   0.223385  -2.271303
H   1.303310  -1.362801  -1.865730
H   0.153025  -0.745138  -0.706807
S   1.824619  -2.230700   0.282296
H   3.924859   1.004955  -0.840698
H   3.792805  -0.648068  -0.889981
H   1.654167  -1.517991   1.414339
C  -2.738252  -0.942146  -0.322943
C  -2.450892  -0.036616   0.919498
O  -1.994553  -1.948024  -0.443969
O  -3.676625  -0.562715  -1.049742
H  -3.270962  -0.190956   1.623577
C  -2.377874   1.449228   0.581710
N  -1.196070  -0.531702   1.580674
H  -1.283147  -0.579707   2.594096
H  -2.000184   2.021789   1.429948
H  -3.381092   1.799750   0.350780
S  -1.278880   1.866384  -0.842378
H  -1.045834  -1.479870   1.198077
H  -0.341062   0.053051   1.366482
H  -2.065801   1.298511  -1.778894
28
Dimer 88 of the z...z  type
C  -0.952288  -1.099757  -0.517825
C  -2.492617  -0.912663  -0.509088
O  -0.473748  -1.632597   0.525303
O  -0.353620  -0.643774  -1.502306
H  -2.916421  -1.180519  -1.474769
C  -2.803031   0.547249  -0.167986
N  -3.089234  -1.829107   0.524537
H  -3.164780  -2.786267   0.177496
H  -2.362211   1.174848  -0.939690
H  -2.357022   0.817325   0.791425
S  -4.597060   0.927814  -0.004714
H  -2.454754  -1.851992   1.333105
H  -4.023644  -1.512030   0.807347
H  -4.926590   0.662781  -1.285843
C   4.003809  -0.302001  -0.325390
C   2.508675   0.052820  -0.034473
O   4.460609  -1.290729   0.305016
O   4.570484   0.416657  -1.172694
H   1.919400  -0.245792  -0.902997
C   2.307607   1.537576   0.234376
N   2.025847  -0.796010   1.104788
H   1.068180  -1.195859   0.906430
H   2.694845   2.092275  -0.616704
H   2.860898   1.851061   1.123006
S   0.551082   2.018911   0.551596
H   2.737924  -1.536711   1.205194
H   1.984698  -0.277200   1.981508
H   0.056102   1.480511  -0.581120
28
Dimer 89 of the z...z  type
C   4.000602  -0.218283   0.411748
C   2.791912   0.398686  -0.371105
O   4.265232  -1.417189   0.148617
O   4.586947   0.564887   1.186742
H   3.210354   1.038540  -1.150460
C   1.864761   1.204667   0.530220
N   2.039718  -0.704469  -1.049127
H   1.504351  -0.394222  -1.861094
H   2.451537   1.955278   1.052717
H   1.391728   0.554474   1.267091
S   0.462711   2.013740  -0.357808
H   2.710647  -1.434947  -1.304459
H   1.317651  -1.116030  -0.401273
H   1.198022   2.939858  -1.006035
C  -1.002965  -1.233937  -0.503153
C  -2.466304  -1.023889  -0.035186
```

```
O  -0.142745  -1.333040   0.421838
O  -0.815094  -1.233099  -1.728527
H  -3.128371  -1.688110  -0.587956
C  -2.869504   0.435425  -0.245497
N  -2.572573  -1.388328   1.422006
H  -2.592297  -2.400612   1.553056
H  -2.728874   0.677605  -1.296822
H  -2.232828   1.098141   0.342121
S  -4.592326   0.824171   0.276289
H  -1.736187  -1.042661   1.907086
H  -3.420336  -0.992032   1.842797
H  -5.207318   0.019224  -0.614138
28
Dimer 90 of the z...z  type
C   4.021599  -0.186744   0.342523
C   2.784457   0.394805  -0.423429
O   4.296657  -1.387780   0.100951
O   4.615780   0.622510   1.083999
H   3.172978   1.020635  -1.229203
C   1.872206   1.210898   0.484524
N   2.030288  -0.735097  -1.053671
H   1.468960  -0.450498  -1.857145
H   2.461466   1.987844   0.963899
H   1.436272   0.575001   1.255733
S   0.424004   1.965956  -0.376378
H   2.704672  -1.461010  -1.312443
H   1.334182  -1.142256  -0.372801
H   1.115048   2.880111  -1.087324
C  -0.977114  -1.268206  -0.358356
C  -2.407303  -0.985241   0.170422
O  -0.071842  -1.330668   0.524981
O  -0.858166  -1.385398  -1.586902
H  -3.041815  -1.841716  -0.058806
C  -2.986923   0.286635  -0.444322
N  -2.362954  -0.870365   1.672268
H  -2.074692  -1.753721   2.095917
H  -2.957594   0.179353  -1.526108
H  -2.383425   1.154514  -0.174673
S  -4.697221   0.683538   0.114151
H  -1.663804  -0.177416   1.953045
H  -3.277901  -0.597832   2.047057
H  -5.295185  -0.388873  -0.444101
28
Dimer 91 of the z...z  type
C   2.459066  -0.595852  -0.866579
C   2.555789   0.886146  -0.376673
O   1.745412  -0.782097  -1.882947
O   3.121414  -1.420019  -0.205836
H   3.510446   1.270200  -0.745731
C   2.529704   1.053531   1.136237
N   1.483574   1.685034  -1.055592
H   1.747103   2.661479  -1.181844
H   2.444251   2.105904   1.411494
H   3.463321   0.669833   1.540888
S   1.139604   0.188457   1.986515
H   1.334444   1.245989  -1.972984
H   0.540456   1.677755  -0.581498
H   1.610115  -1.052003   1.743732
C  -2.166148   1.504072  -0.253522
C  -2.060615   0.054530   0.293067
O  -3.309092   1.929095  -0.503180
O  -1.064113   2.095194  -0.392395
H  -1.494566   0.067266   1.221435
C  -1.390492  -0.873546  -0.715069
N  -3.444094  -0.442014   0.622639
H  -3.828183   0.052118   1.429219
H  -0.408507  -0.486521  -0.971727
H  -1.979034  -0.938800  -1.632750
S  -1.197349  -2.607223  -0.121833
```



```
H  -4.075148  -0.251302  -0.161514
H  -3.432841  -1.447218   0.826347
H  -0.297099  -2.326258   0.840789
28
Dimer 92 of the z...z  type
C   1.325156  -0.351695   0.031191
C   2.424060   0.742008   0.082539
O   0.733126  -0.577803   1.127797
O   1.098440  -0.856848  -1.078727
H   1.954849   1.658389  -0.280779
C   3.652393   0.430113  -0.761235
N   2.781914   0.977194   1.525122
H   3.123986   1.921119   1.707157
H   4.373144   1.247801  -0.722674
H   3.328028   0.296603  -1.790547
S   4.562060  -1.074645  -0.204792
H   1.935356   0.783728   2.078750
H   3.498392   0.303860   1.820652
H   3.571181  -1.944260  -0.491349
C  -4.128645  -0.781728   0.165787
C  -2.727900  -0.082571   0.279761
O  -4.099512  -2.025976  -0.009729
O  -5.112929  -0.027514   0.292188
H  -2.596297   0.190248   1.328437
C  -2.634390   1.161348  -0.593584
N  -1.663917  -1.085781  -0.043772
H  -2.123295  -2.006332   0.024825
H  -3.471564   1.810513  -0.349695
H  -2.711280   0.895799  -1.650739
S  -1.052987   2.101793  -0.443816
H  -1.295563  -0.988480  -0.992345
H  -0.818520  -1.014243   0.574304
H  -1.156613   2.371274   0.873287
28
Dimer 93 of the z...z  type
C   1.169202  -0.244595  -0.769128
C   2.251393   0.764785  -0.303130
O   0.660052  -0.948849   0.152170
O   0.935875  -0.269958  -1.983542
H   2.094837   1.714523  -0.814141
C   3.661241   0.257341  -0.606789
N   2.066786   1.022271   1.167947
H   2.760248   1.671717   1.543511
H   4.408911   1.005164  -0.336712
H   3.726318   0.081454  -1.679710
S   4.146907  -1.267012   0.311307
H   1.125037   1.410591   1.321159
H   2.123832   0.134886   1.677564
H   3.171053  -2.062248  -0.173133
C  -3.690962  -0.947514  -0.124804
C  -2.709605   0.097610   0.518166
O  -3.600746  -2.115982   0.329005
O  -4.466188  -0.493055  -0.986706
H  -3.306720   0.720078   1.186229
C  -2.031677   0.961195  -0.534943
N  -1.724350  -0.655395   1.362772
H  -2.170974  -1.570688   1.535889
H  -2.806426   1.433602  -1.133116
H  -1.404397   0.359875  -1.195374
S  -0.906688   2.269081   0.130294
H  -0.818154  -0.824051   0.855627
H  -1.511869  -0.187266   2.241017
H  -1.761725   2.800951   1.026881
28
Dimer 94 of the z...z  type
C  -2.576560  -0.670209   0.990276
C  -2.782722  -0.577526  -0.555313
O  -3.592256  -0.477629   1.683673
O  -1.414544  -0.961711   1.367915
```

```
H  -2.969194  -1.593161  -0.910832
C  -1.605339   0.005147  -1.321733
N  -4.045562   0.205123  -0.793895
H  -4.524980  -0.051907  -1.657128
H  -1.817978   0.053775  -2.390261
H  -0.755410  -0.657854  -1.178004
S  -1.171448   1.728849  -0.827510
H  -4.651234   0.035931   0.023744
H  -3.839652   1.209896  -0.819273
H  -0.967691   1.459426   0.478172
C   2.294603  -1.754833  -0.599375
C   1.873005  -0.349851  -0.061669
O   2.370005  -2.665459   0.261635
O   2.538868  -1.794113  -1.821358
H   1.133424   0.088168  -0.725417
C   3.090041   0.569663   0.003777
N   1.249443  -0.518767   1.288830
H   1.696737  -1.341464   1.715702
H   3.525499   0.631607  -0.991372
H   3.842134   0.170709   0.687601
S   2.706370   2.264855   0.628788
H   1.381634   0.310127   1.874382
H   0.220777  -0.713948   1.232173
H   1.765265   2.551893  -0.295186
28
Dimer 95 of the z...z  type
C   3.961758   0.498854  -0.556831
C   2.845650  -0.264734   0.239878
O   4.169789   1.682636  -0.195215
O   4.540802  -0.170848  -1.435381
H   3.357412  -0.932379   0.935597
C   1.924964  -1.061850  -0.674805
N   2.078148   0.728232   1.059030
H   2.732084   1.456501   1.361083
H   2.539117  -1.697928  -1.306252
H   1.340363  -0.393268  -1.308065
S   0.666249  -2.096571   0.194681
H   1.321053   1.180391   0.486703
H   1.599830   0.311460   1.858906
H   1.521326  -2.865004   0.898326
C  -0.939133   0.832806   0.583044
C  -2.152072   0.037416   0.039032
O  -0.811539   0.894667   1.822514
O  -0.194834   1.330555  -0.300767
H  -1.834628  -0.586928  -0.794075
C  -3.262035   0.991849  -0.394515
N  -2.633700  -0.879244   1.127899
H  -1.977658  -1.652425   1.260336
H  -2.858854   1.657971  -1.155109
H  -3.596997   1.597316   0.449996
S  -4.775416   0.152943  -1.029180
H  -2.656667  -0.357612   2.011790
H  -3.560932  -1.261552   0.914182
H  -4.198447  -0.403534  -2.113947
28
Dimer 96 of the z...z  type
C  -1.160961   0.021164   0.065067
C  -2.624205  -0.243035  -0.375223
O  -0.711299  -0.764394   0.952528
O  -0.594843   0.987239  -0.463428
H  -2.686755  -0.241968  -1.462177
C  -3.540437   0.828497   0.217950
N  -3.043679  -1.609164   0.098982
H  -2.623292  -2.345755  -0.469155
H  -3.199313   1.798802  -0.137290
H  -3.479212   0.821849   1.308305
S  -5.326805   0.601410  -0.171587
H  -2.703121  -1.745364   1.057379
H  -4.064099  -1.713858   0.066813
```



```
H  -5.220265   0.783763  -1.503792
C   2.736992  -1.705304  -0.360283
C   2.417413  -0.202073  -0.091912
O   2.805787  -2.445469   0.651509
O   2.923108  -1.992692  -1.561790
H   1.646114   0.138481  -0.780404
C   3.687475   0.627511  -0.262117
N   1.854499  -0.057133   1.284875
H   0.831186  -0.343312   1.267370
H   4.080978   0.457365  -1.262246
H   4.446497   0.325905   0.463159
S   3.450275   2.439080   0.011043
H   2.358422  -0.690859   1.911443
H   1.920856   0.907542   1.616624
H   2.561063   2.625605  -0.985765
28
Dimer 97 of the z...z  type
C  -3.960701  -0.500594  -0.557090
C  -2.845758   0.265126   0.239170
O  -4.168364  -1.683840  -0.193553
O  -4.539369   0.167210  -1.437348
H  -3.358491   0.933840   0.933145
C  -1.924647   1.060980  -0.676246
N  -2.078378  -0.725778   1.060950
H  -2.732158  -1.453434   1.364738
H  -2.538571   1.695807  -1.309174
H  -1.339424   0.391517  -1.307999
S  -0.666843   2.097433   0.192446
H  -1.321153  -1.179258   0.489847
H  -1.600306  -0.307002   1.859948
H  -1.522609   2.866651   0.894410
C   0.939031  -0.831284   0.585317
C   2.151886  -0.037143   0.039236
O   0.811605  -0.890311   1.824937
O   0.194635  -1.331092  -0.297255
H   1.834285   0.585446  -0.795128
C   3.261531  -0.992750  -0.392636
N   2.634198   0.881581   1.126031
H   1.978626   1.655402   1.257033
H   2.857863  -1.660723  -1.151348
H   3.596909  -1.596116   0.453225
S   4.774514  -0.155403  -1.030233
H   2.657012   0.361651   2.010934
H   3.561600   1.262918   0.911329
H   4.196843   0.398441  -2.115984
28
Dimer 98 of the z...z  type
C  -0.917107  -0.854015   0.635660
C  -2.163218  -0.033650   0.197020
O  -0.264463  -1.387111  -0.311057
O  -0.702473  -0.910457   1.851938
H  -2.225068   0.875921   0.791519
C  -3.428261  -0.872929   0.361376
N  -1.982056   0.384614  -1.235509
H  -1.262081   1.115416  -1.290414
H  -3.504893  -1.172179   1.405187
H  -3.373697  -1.775874  -0.250292
S  -4.974725  -0.015566  -0.160340
H  -1.634298  -0.418012  -1.771782
H  -2.856435   0.732969  -1.641436
H  -4.936184   0.952177   0.778251
C   3.952817  -0.547568   0.058252
C   2.807012   0.395856  -0.456111
O   4.095600  -1.615536  -0.588466
O   4.608932  -0.115115   1.024185
H   3.290343   1.206020  -1.005202
C   1.972753   0.960892   0.683271
N   1.979122  -0.378904  -1.438866
H   2.595210  -1.138025  -1.769541
```

```
H   2.640430   1.480596   1.365342
H   1.464982   0.167524   1.234304
S   0.625477   2.115260   0.161900
H   1.141401  -0.818743  -0.981696
H   1.653354   0.188069  -2.219123
H   1.370695   2.903308  -0.639073
28
Dimer 99 of the z...z  type
C  -0.917256   0.850804  -0.639009
C  -2.163721   0.032030  -0.198470
O  -0.265598   1.387241   0.306540
O  -0.701500   0.903274  -1.855247
H  -2.227198  -0.877963  -0.792132
C  -3.427906   0.872743  -0.362259
N  -1.981397  -0.385047   1.234262
H  -1.263666  -1.118158   1.288787
H  -3.505601   1.170628  -1.406387
H  -3.371328   1.776454   0.248088
S  -4.974768   0.018098   0.162747
H  -1.630077   0.417378   1.768624
H  -2.856146  -0.730005   1.642282
H  -4.938325  -0.951412  -0.774100
C   3.952134   0.549269  -0.057214
C   2.806476  -0.394016   0.457794
O   4.093174   1.618806   0.587333
O   4.609847   0.115318  -1.021379
H   3.289791  -1.202611   1.009181
C   1.974184  -0.961963  -0.681560
N   1.976803   0.382142   1.437996
H   1.650566  -0.183468   2.219037
H   2.643249  -1.482307  -1.361782
H   1.466378  -0.170139  -1.234766
S   0.627296  -2.116802  -0.160085
H   2.592009   1.142343   1.767867
H   1.139279   0.820546   0.979001
H   1.371924  -2.901988   0.644239
28
Dimer 100 of the z...z  type
C   1.664923  -0.454802  -0.221998
C   3.109413  -0.910451   0.127429
O   1.289963  -0.628766  -1.399358
O   1.010274   0.030213   0.737320
H   3.035246  -1.900437   0.581955
C   3.845249   0.027244   1.077059
N   3.850435  -1.078956  -1.170912
H   4.587583  -1.782957  -1.127906
H   4.849919  -0.341453   1.287847
H   3.286089   0.074219   2.008358
S   4.060123   1.730286   0.403607
H   3.148133  -1.330491  -1.882631
H   4.263679  -0.183813  -1.457512
H   2.744541   2.029070   0.380470
C  -2.580291  -1.705614   0.431346
C  -2.555870  -0.143686   0.378282
O  -1.940039  -2.294557  -0.472221
O  -3.277103  -2.186040   1.349746
H  -2.427355   0.250515   1.385569
C  -3.874005   0.351082  -0.216738
N  -1.391920   0.305949  -0.438901
H  -0.482182   0.267130   0.107156
H  -4.693817  -0.028679   0.389848
H  -3.995592  -0.023931  -1.235421
S  -4.006439   2.187139  -0.359493
H  -1.253547  -0.349967  -1.215753
H  -1.522526   1.254821  -0.796820
H  -3.930002   2.448785   0.961535
28
Dimer 101 of the z...z  type
C  -1.644954   1.257107  -0.992055
```



```
C  -2.718737   0.134170  -1.006052
O  -0.464037   0.881929  -1.262487
O  -2.049470   2.382583  -0.677918
H  -3.428340   0.326446  -1.812610
C  -3.473614   0.051560   0.317160
N  -2.047458  -1.176863  -1.319323
H  -2.714345  -1.943514  -1.426228
H  -4.140313  -0.811507   0.336971
H  -4.074241   0.951870   0.422089
S  -2.384006  -0.101692   1.803023
H  -1.502399  -1.089471  -2.179525
H  -1.366686  -1.430142  -0.583820
H  -2.007928   1.193592   1.818554
C   3.446958   0.849363   0.097357
C   2.327653  -0.157205   0.543706
O   3.231768   2.052032   0.383543
O   4.437102   0.333397  -0.456881
H   2.687798  -0.643837   1.452513
C   2.020019  -1.196951  -0.522862
N   1.104540   0.625167   0.912055
H   1.417546   1.509632   1.326024
H   2.947851  -1.696964  -0.786721
H   1.606161  -0.722192  -1.414327
S   0.757366  -2.455343  -0.031010
H   0.540283   0.851646   0.052621
H   0.487990   0.121483   1.550504
H   1.186432  -2.670393   1.229044
28
Dimer 102 of the z...z  type
C   3.585586  -0.407529   0.724469
C   2.691737   0.410244  -0.269492
O   3.520358  -1.657521   0.623934
O   4.244016   0.286561   1.525541
H   3.303934   1.174461  -0.744572
C   1.539150   1.054981   0.497301
N   2.183146  -0.521946  -1.331311
H   2.795052  -0.531865  -2.144948
H   1.944918   1.533600   1.385835
H   0.829169   0.290975   0.814696
S   0.576045   2.310203  -0.457225
H   2.210708  -1.459835  -0.902669
H   1.189609  -0.356222  -1.639498
H   1.461227   3.317039  -0.309461
C  -0.866806  -1.109789  -0.786114
C  -2.358100  -0.924450  -0.405383
O  -0.163619  -1.788664  -0.007888
O  -0.503784  -0.477183  -1.808619
H  -2.998004  -1.084550  -1.271080
C  -2.546901   0.488315   0.151938
N  -2.713951  -1.955101   0.630852
H  -2.917509  -2.859737   0.203982
H  -2.272517   1.196250  -0.626553
H  -1.887393   0.655196   1.006152
S  -4.244268   0.847531   0.769183
H  -1.894249  -2.088134   1.237691
H  -3.530583  -1.665854   1.180639
H  -4.870930   0.718101  -0.418374
28
Dimer 103 of the z...z  type
C   3.587470   0.547315   0.402175
C   2.412040  -0.441094   0.679065
O   3.418383   1.725413   0.795363
O   4.562821   0.042532  -0.193143
H   2.746249  -1.180804   1.409684
C   1.954478  -1.181038  -0.575139
N   1.263044   0.294796   1.302840
H   1.011856   1.084677   0.704474
H   1.152512  -1.881599  -0.339744
H   2.805089  -1.728175  -0.974408
```

```
S   1.270872  -0.078748  -1.890103
H   0.426175  -0.345758   1.420692
H   1.544144   0.696456   2.196318
H   2.389397   0.651631  -2.078632
C  -1.709509  -1.608867   0.646051
C  -2.035339  -0.305550  -0.136914
O  -2.480295  -2.568507   0.481693
O  -0.702598  -1.526930   1.400128
H  -1.129626   0.107932  -0.576774
C  -2.685112   0.714783   0.797450
N  -2.962720  -0.644141  -1.270613
H  -2.456382  -1.055857  -2.056134
H  -2.010839   0.897318   1.632040
H  -3.622779   0.325563   1.198868
S  -3.117442   2.306578  -0.023504
H  -3.631981  -1.350989  -0.940595
H  -3.456809   0.189357  -1.608260
H  -1.854831   2.657013  -0.343861
28
Dimer 104 of the z...z  type
C  -1.161800   1.358526   0.133741
C  -2.648776   0.938704   0.266229
O  -0.769712   1.698338  -1.001579
O  -0.493515   1.255135   1.193727
H  -3.092397   1.393529   1.149936
C  -2.740130  -0.585737   0.339177
N  -3.396124   1.447574  -0.936539
H  -3.625127   2.438312  -0.845812
H  -2.160186  -0.919589   1.197011
H  -2.310638  -1.035309  -0.557382
S  -4.460189  -1.236037   0.437588
H  -2.780762   1.356097  -1.755153
H  -4.268416   0.926192  -1.078758
H  -4.774588  -0.662698   1.617476
C   4.230789   0.114679   0.288224
C   2.695447  -0.205915   0.310781
O   4.529459   1.332162   0.194417
O   4.984132  -0.873336   0.393156
H   2.448236  -0.504964   1.331220
C   2.330650  -1.324995  -0.655253
N   1.935109   1.052254   0.028619
H   1.080096   1.179529   0.634221
H   2.938513  -2.194621  -0.418309
H   2.549064  -1.030324  -1.684799
S   0.543183  -1.783693  -0.652951
H   2.614557   1.817952   0.150374
H   1.573919   1.096452  -0.926233
H   0.466925  -2.113637   0.652286
28
Dimer 105 of the z...z  type
C   2.321528  -1.333753  -0.765052
C   2.061994  -0.280268   0.354447
O   3.509576  -1.610964  -1.000561
O   1.284346  -1.761806  -1.333821
H   1.293459  -0.647470   1.032080
C   1.650083   1.049456  -0.275240
N   3.324660  -0.119709   1.156003
H   3.408425  -0.848142   1.866759
H   0.790591   0.881748  -0.922224
H   2.454149   1.443041  -0.900498
S   1.279344   2.387563   0.936636
H   4.118778  -0.239350   0.511693
H   3.369263   0.789853   1.626602
H   0.362967   1.709006   1.657662
C  -2.294343  -1.439133   1.075055
C  -1.672278  -0.398461   0.084522
O  -2.638322  -2.533354   0.564363
O  -2.399271  -1.041165   2.250841
H  -0.760375  -0.009447   0.527655
```



```
C  -2.656233   0.744013  -0.153864
N  -1.316438  -1.104283  -1.185680
H  -0.296496  -1.388144  -1.201185
H  -2.896356   1.197320   0.805565
H  -3.579565   0.364508  -0.596248
S  -2.053301   2.038173  -1.324827
H  -1.897662  -1.956745  -1.199313
H  -1.492467  -0.532903  -2.014261
H  -1.064100   2.520080  -0.541274
28
Dimer 106 of the z...z  type
C  -1.027894   1.185569   1.115653
C  -2.373978   0.650102   0.568222
O  -0.525453   2.152656   0.508343
O  -0.531826   0.517341   2.057983
H  -2.993110   0.253086   1.369385
C  -2.068246  -0.421661  -0.479982
N  -3.114150   1.794648  -0.070275
H  -3.562034   2.390758   0.627613
H  -1.458243  -1.204806  -0.032118
H  -1.493361   0.012801  -1.301307
S  -3.564456  -1.151834  -1.267379
H  -2.427668   2.371886  -0.573793
H  -3.835434   1.456855  -0.717049
H  -4.062161  -1.721440  -0.149954
C   1.328456  -1.804468   0.512873
C   1.712124  -0.356654   0.074906
O   1.817844  -2.189442   1.601628
O   0.604629  -2.424349  -0.294834
H   0.860801   0.124171  -0.401529
C   2.891632  -0.405394  -0.890878
N   2.035520   0.443408   1.296893
H   1.126126   0.616120   1.804878
H   2.622132  -1.035061  -1.737254
H   3.769364  -0.837576  -0.405846
S   3.433499   1.251234  -1.500970
H   2.643115  -0.114131   1.903334
H   2.479437   1.333759   1.062620
H   2.282634   1.562178  -2.131460
28
Dimer 107 of the z...z  type
C   2.215608  -1.357918  -0.355337
C   2.713096  -0.526580   0.863489
O   3.094768  -1.824207  -1.105218
O   0.970298  -1.491953  -0.443009
H   2.616121  -1.156906   1.750081
C   1.965253   0.782913   1.092194
N   4.185350  -0.276117   0.664676
H   4.697183  -0.147652   1.537835
H   2.362468   1.298406   1.967907
H   0.910499   0.580598   1.259905
S   2.108037   1.971489  -0.310391
H   4.559660  -1.072953   0.131039
H   4.316419   0.555943   0.077704
H   1.165849   1.401726  -1.090355
C  -1.410979   0.856805  -0.489754
C  -1.914703  -0.612291  -0.590935
O  -1.238431   1.288763   0.677375
O  -1.271724   1.451870  -1.577728
H  -1.448768  -1.088400  -1.452059
C  -3.434086  -0.672993  -0.741604
N  -1.438583  -1.350002   0.623109
H  -0.407302  -1.493718   0.486979
H  -3.763468  -1.701126  -0.904585
H  -3.709841  -0.084565  -1.616303
S  -4.389510  -0.097448   0.729651
H  -1.588579  -0.752317   1.441680
H  -1.906353  -2.245695   0.759526
H  -3.773570   1.096122   0.858553
```

```
28
Dimer 108 of the z...z  type
C  -0.864260  -0.523016  -0.853705
C  -2.032071  -0.114763   0.080512
O  -0.687306   0.173188  -1.875067
O  -0.189730  -1.510606  -0.464869
H  -1.707134  -0.182234   1.117852
C  -3.244850  -1.010202  -0.162714
N  -2.365946   1.326024  -0.184808
H  -1.586279   1.927120   0.119362
H  -2.948481  -2.042191   0.016760
H  -3.580901  -0.925367  -1.198202
S  -4.713404  -0.589455   0.868011
H  -2.467053   1.470187  -1.194697
H  -3.226516   1.605337   0.296528
H  -4.156828  -0.895991   2.057563
C   3.650552  -0.857005   0.609258
C   2.794062   0.229206  -0.131552
O   3.846135  -1.919514  -0.026736
O   4.068747  -0.521365   1.736157
H   3.481840   0.996982  -0.490209
C   1.741458   0.848595   0.778100
N   2.162945  -0.399239  -1.340668
H   2.865004  -0.957661  -1.829271
H   2.237129   1.178353   1.686420
H   0.991282   0.106683   1.049274
S   0.865975   2.309612   0.029560
H   1.380469  -1.039994  -1.057891
H   1.745959   0.285865  -1.974296
H   1.379260   3.252389   0.842722
28
Dimer 109 of the z...z  type
C   1.006495  -0.282250  -1.237942
C   2.001051  -0.020499  -0.071354
O   1.216006  -1.290450  -1.934411
O   0.094709   0.580814  -1.344412
H   1.466360   0.340230   0.804926
C   3.056270   0.993551  -0.509335
N   2.637576  -1.329890   0.301945
H   1.995240  -1.884818   0.874844
H   2.547748   1.916986  -0.780607
H   3.596662   0.628773  -1.384846
S   4.348720   1.333587   0.759607
H   2.812952  -1.859381  -0.561509
H   3.509358  -1.189772   0.823625
H   3.521563   1.870388   1.679845
C  -3.598168   1.122904   0.253171
C  -2.844402  -0.248033   0.149818
O  -3.922757   1.644253  -0.842513
O  -3.819358   1.522854   1.413082
H  -3.569911  -1.030595   0.377824
C  -1.660176  -0.332088   1.102936
N  -2.414536  -0.441933  -1.277814
H  -2.387797  -1.423779  -1.547905
H  -2.012804  -0.170124   2.117370
H  -0.931917   0.440314   0.860753
S  -0.750064  -1.942845   1.018458
H  -3.089443   0.077941  -1.853636
H  -1.458746  -0.028042  -1.446813
H  -1.454255  -2.598512   1.960459
28
Dimer 110 of the z...z  type
C  -3.454662  -0.407566  -0.853472
C  -2.638404   0.441037   0.176589
O  -3.651881  -1.606368  -0.545585
O  -3.834585   0.222595  -1.863281
H  -3.319016   1.168215   0.622878
C  -1.465105   1.157895  -0.484770
N  -2.147300  -0.452584   1.274394
```



```
H  -2.919237  -0.994253   1.661181
H  -1.840833   1.713661  -1.339779
H  -0.735247   0.429918  -0.841579
S  -0.537845   2.300865   0.632993
H  -1.432054  -1.121962   0.889105
H  -1.671064   0.068596   2.014672
H  -1.485154   3.259100   0.683714
C   0.902426  -1.128085   0.990595
C   2.316702  -0.974702   0.373650
O   0.041334  -1.678633   0.240205
O   0.733448  -0.631005   2.112466
H   3.079948  -1.221825   1.108235
C   2.481979   0.466752  -0.112853
N   2.452714  -1.936962  -0.775057
H   2.670955  -2.880069  -0.450208
H   2.359528   1.127845   0.742332
H   1.711927   0.715730  -0.845353
S   4.079685   0.807210  -0.961290
H   1.545511  -1.986642  -1.256741
H   3.190226  -1.641092  -1.424681
H   4.873030   0.575695   0.104740
28
Dimer 111 of the z...z  type
C  -3.498927  -0.640056  -0.067935
C  -2.088878   0.023448   0.107808
O  -3.761258  -1.054187  -1.225391
O  -4.202847  -0.659561   0.958975
H  -1.570586  -0.474844   0.926519
C  -2.252131   1.510416   0.403494
N  -1.305091  -0.218334  -1.146162
H  -0.587670  -0.982211  -0.980232
H  -2.899922   1.622443   1.269964
H  -2.719850   2.019479  -0.442065
S  -0.670967   2.416395   0.700948
H  -2.005702  -0.547538  -1.833332
H  -0.837155   0.618258  -1.493760
H  -0.355898   1.808791   1.863042
C   1.446140  -2.046499   0.186183
C   1.907346  -0.571971   0.362345
O   2.176911  -2.926487   0.668893
O   0.380839  -2.180281  -0.474857
H   1.077980   0.042929   0.705816
C   2.451817  -0.034610  -0.961541
N   2.971011  -0.515700   1.423014
H   2.567492  -0.561179   2.359748
H   1.686364  -0.153552  -1.725434
H   3.326354  -0.607465  -1.275898
S   2.998168   1.723122  -0.888242
H   3.577854  -1.338315   1.320112
H   3.519989   0.348283   1.351358
H   1.782732   2.230652  -0.590199
28
Dimer 112 of the z...z  type
C  -3.817958   0.746251  -0.364781
C  -2.459251   0.005847  -0.113999
O  -3.718718   1.928514  -0.780455
O  -4.842534   0.069925  -0.150859
H  -2.327694  -0.713254  -0.924654
C  -2.430527  -0.717094   1.224220
N  -1.338804   0.993367  -0.247941
H  -0.542969   0.565176  -0.798507
H  -3.264727  -1.413327   1.254366
H  -2.548979  -0.009990   2.048846
S  -0.851972  -1.610465   1.574490
H  -1.751414   1.805801  -0.732626
H  -0.997122   1.312264   0.658134
H  -0.826626  -2.334671   0.436780
C   1.470426  -0.940126  -1.385778
C   2.136793  -0.302757  -0.134220
```

```
O   2.081289  -1.870605  -1.938295
O   0.381539  -0.404497  -1.724018
H   1.407928  -0.238056   0.671877
C   2.688539   1.079825  -0.479001
N   3.238194  -1.210401   0.339309
H   2.864000  -2.004702   0.860347
H   1.870183   1.690915  -0.854750
H   3.446282   1.009808  -1.262029
S   3.514196   1.930454   0.931445
H   3.721041  -1.595646  -0.481261
H   3.898347  -0.709843   0.944372
H   2.419692   2.016562   1.714934
28
Dimer 113 of the z...z  type
C   1.058858  -0.936735  -0.097882
C   2.306231  -0.020261  -0.057100
O   0.597802  -1.274429   1.029734
O   0.636488  -1.243817  -1.226092
H   2.215410   0.748382  -0.822308
C   3.577813  -0.840044  -0.258935
N   2.326582   0.678905   1.273670
H   1.548725   1.342196   1.327911
H   3.505974  -1.347493  -1.219454
H   3.675710  -1.597471   0.521408
S   5.132305   0.148208  -0.185767
H   2.173641  -0.010300   2.017542
H   3.209936   1.175914   1.425701
H   4.892525   0.889765  -1.286459
C  -4.272903  -0.300133   0.072717
C  -2.750290   0.043789   0.276802
O  -4.542996  -1.526250   0.024646
O  -5.042593   0.678268   0.024718
H  -2.640554   0.338453   1.321775
C  -2.277696   1.164631  -0.638106
N  -1.960017  -1.214866   0.090576
H  -1.113187  -1.299092   0.697541
H  -3.039700   1.939774  -0.645785
H  -2.156304   0.805344  -1.662088
S  -0.666454   1.957149  -0.195298
H  -2.627621  -1.985361   0.242025
H  -1.585882  -1.305841  -0.858378
H  -1.119925   2.634073   0.878867
28
Dimer 114 of the z...z  type
C   1.452346  -0.286140  -0.297351
C   2.757038  -0.067176  -1.118059
O   0.627899   0.671423  -0.336010
O   1.342039  -1.375720   0.289481
H   2.606491  -0.539003  -2.091351
C   4.003758  -0.647805  -0.462497
N   2.895905   1.410971  -1.367532
H   3.409205   1.632802  -2.221110
H   4.885582  -0.484734  -1.082966
H   3.853952  -1.717773  -0.339769
S   4.388312   0.108159   1.175376
H   1.940119   1.794825  -1.405351
H   3.369517   1.855346  -0.572387
H   3.280839  -0.351020   1.794084
C  -3.048264  -1.248412  -0.500083
C  -2.775934  -0.638366   0.914167
O  -2.106400  -1.907187  -0.998612
O  -4.190579  -1.032818  -0.959848
H  -3.184215  -1.343927   1.643555
C  -3.432502   0.716512   1.148594
N  -1.300719  -0.581245   1.150950
H  -1.067742  -0.393301   2.125982
H  -3.186413   1.105661   2.138309
H  -4.510284   0.585860   1.086820
S  -2.924690   2.026169  -0.046505
```



```
H  -0.851607  -1.460197   0.870702
H  -0.799196   0.124048   0.557439
H  -3.457868   1.419753  -1.127160
28
Dimer 115 of the z...z  type
C   1.147810  -1.889687  -0.218692
C   2.008204  -0.701464   0.299817
O   1.764609  -2.930304  -0.504932
O  -0.079316  -1.638580  -0.346955
H   1.487608  -0.153882   1.082545
C   2.340580   0.219825  -0.876532
N   3.275064  -1.257196   0.889900
H   3.126375  -1.609522   1.836457
H   1.411622   0.601468  -1.295705
H   2.856000  -0.340154  -1.659420
S   3.462376   1.618163  -0.457611
H   3.568519  -2.054243   0.309070
H   4.012580  -0.543734   0.921912
H   2.604437   2.259622   0.361597
C  -3.705676  -0.268760  -0.336853
C  -2.599135   0.481039   0.481457
O  -3.959176  -1.438142   0.042866
O  -4.233408   0.396948  -1.249907
H  -3.113839   1.164503   1.159488
C  -1.631996   1.250002  -0.408170
N  -1.866091  -0.520083   1.324750
H  -1.445549  -0.100355   2.152560
H  -2.196760   1.950077  -1.017524
H  -1.099358   0.562364  -1.066832
S  -0.312752   2.148662   0.520890
H  -2.540433  -1.247728   1.585720
H  -1.107791  -0.978759   0.752394
H  -1.120604   2.996993   1.187968
28
Dimer 116 of the z...z  type
C  -1.073810  -1.103815  -0.163387
C  -2.626917  -1.219899  -0.203607
O  -0.520138  -1.427717   0.905578
O  -0.556235  -0.695739  -1.231926
H  -2.872725  -2.157527  -0.706191
C  -3.310427  -0.063826  -0.925130
N  -3.103058  -1.345917   1.218363
H  -3.970304  -1.874776   1.312372
H  -4.392426  -0.199561  -0.947211
H  -2.938209  -0.038280  -1.946374
S  -3.021170   1.571819  -0.122771
H  -2.339395  -1.794586   1.746062
H  -3.239288  -0.411678   1.622598
H  -1.680285   1.583555  -0.296819
C   3.032403  -0.874897   0.204970
C   2.874380   0.447443  -0.619650
O   2.919698  -1.925275  -0.472761
O   3.300692  -0.742838   1.416083
H   3.851236   0.632515  -1.076681
C   2.507368   1.687711   0.181631
N   1.922501   0.180635  -1.748056
H   1.832906   0.977997  -2.377458
H   2.567435   2.582836  -0.440602
H   3.236032   1.773507   0.985210
S   0.810436   1.708813   0.899225
H   2.273850  -0.623723  -2.271604
H   0.954646  -0.110117  -1.413125
H   0.782058   0.422958   1.309650
28
Dimer 117 of the z...z  type
C  -2.691190   1.367294  -0.174132
C  -2.161575  -0.083924  -0.372664
O  -2.612073   1.835675   0.985540
O  -3.159310   1.892216  -1.208410
```

```
H  -1.341500  -0.068536  -1.092217
C  -3.246726  -1.023872  -0.890006
N  -1.571341  -0.578810   0.909706
H  -1.237970  -1.539434   0.818060
H  -2.855098  -2.031760  -1.039898
H  -3.600624  -0.642603  -1.845568
S  -4.683101  -1.223746   0.253809
H  -0.719601  -0.000713   1.122821
H  -2.251613  -0.501785   1.666788
H  -5.120462   0.049330   0.166160
C   1.368978  -0.169854   0.031161
C   2.819839   0.164189  -0.403212
O   0.855423   0.623072   0.873391
O   0.874401  -1.198301  -0.457912
H   2.912983   0.075730  -1.483629
C   3.791260  -0.785826   0.299093
N   3.122576   1.592272  -0.035149
H   2.722919   2.244962  -0.710739
H   3.527822  -1.804069   0.020009
H   3.700071  -0.692881   1.383163
S   5.566778  -0.464948  -0.072634
H   2.680443   1.791375   0.871036
H   4.134555   1.756621   0.012405
H   5.499566  -0.740712  -1.391404
28
Dimer 118 of the z...z  type
C  -1.040408   2.089556  -0.023217
C  -1.571373   0.624733   0.047316
O  -1.231601   2.792339   1.000047
O  -0.481529   2.386671  -1.097707
H  -0.766028  -0.039258  -0.243750
C  -2.741317   0.398816  -0.900443
N  -1.922141   0.304092   1.470347
H  -1.145718  -0.266189   1.883934
H  -2.417158   0.667037  -1.903930
H  -3.597505   1.020257  -0.630769
S  -3.352233  -1.349021  -0.875224
H  -1.989516   1.212629   1.954300
H  -2.793325  -0.222765   1.553038
H  -2.130494  -1.928728  -0.864233
C   0.727532  -1.555109   0.863173
C   2.132805  -1.086912   0.396027
O   0.079993  -2.277358   0.077961
O   0.365904  -1.065534   1.963217
H   2.866525  -1.259820   1.181102
C   2.073041   0.401545   0.038774
N   2.539928  -1.902835  -0.800777
H   2.910664  -2.812956  -0.523909
H   1.741353   0.943888   0.921834
H   1.353458   0.596566  -0.759062
S   3.663246   1.097287  -0.572773
H   1.700613  -2.082615  -1.365924
H   3.254066  -1.420374  -1.357090
H   4.368528   0.886560   0.557498
28
Dimer 119 of the z...z  type
C  -0.992744   0.076242  -0.153854
C  -2.410300   0.000469   0.470586
O  -0.585890   1.200172  -0.506594
O  -0.409978  -1.032552  -0.272753
H  -2.398230  -0.632278   1.355394
C  -3.392540  -0.544705  -0.567290
N  -2.806701   1.387122   0.898502
H  -2.424821   1.619661   1.816454
H  -3.054506  -1.535899  -0.863036
H  -3.405389   0.092436  -1.454211
S  -5.139862  -0.615260   0.011125
H  -2.398518   2.049454   0.224552
H  -3.826624   1.489692   0.939894
```



```
H  -4.931931  -1.495433   1.011943
C   3.201404  -1.621480   0.391692
C   2.941478  -0.252844  -0.321169
O   2.820103  -2.640399  -0.231708
O   3.800497  -1.546590   1.485943
H   3.886369   0.050907  -0.777329
C   2.450180   0.836798   0.623943
N   1.954235  -0.455357  -1.429556
H   2.269489  -1.207289  -2.041713
H   3.113090   0.873473   1.484145
H   1.440600   0.618509   0.969152
S   2.352445   2.511491  -0.152078
H   1.012948  -0.739861  -1.021004
H   1.818285   0.403134  -1.966251
H   3.682432   2.739517  -0.167931
28
Dimer 120 of the z...z  type
C  -3.197976   0.698192   0.532835
C  -3.062368  -0.368619  -0.606528
O  -3.165175   1.887469   0.140031
O  -3.358537   0.248985   1.686677
H  -4.068233  -0.506803  -1.014068
C  -2.566016  -1.739846  -0.174756
N  -2.242771   0.229860  -1.712307
H  -2.206059  -0.366217  -2.539270
H  -2.501146  -2.420226  -1.025777
H  -3.291307  -2.138982   0.530881
S  -0.908288  -1.800282   0.626178
H  -2.665469   1.127254  -1.962106
H  -1.259389   0.471421  -1.413704
H  -1.140684  -0.826349   1.529825
C   0.829905   1.133022  -0.034603
C   1.992810   0.109927   0.106774
O   0.234040   1.118195  -1.153132
O   0.632224   1.859808   0.945247
H   1.874987  -0.432992   1.043760
C   3.343848   0.818332   0.070080
N   1.885002  -0.900318  -0.999331
H   1.034444  -1.459141  -0.850256
H   3.358286   1.560965   0.865351
H   3.484677   1.333686  -0.882253
S   4.790577  -0.313085   0.237661
H   1.768580  -0.417803  -1.894855
H   2.704471  -1.514234  -1.032489
H   4.512977  -0.725209   1.491593
28
Dimer 121 of the z...z  type
C   1.062276  -0.183198  -0.115768
C   2.496007   0.066602   0.419780
O   0.530475  -1.273900   0.169889
O   0.597276   0.746787  -0.824633
H   2.572707   1.067670   0.839195
C   3.500837  -0.116913  -0.719345
N   2.761594  -0.918788   1.525304
H   2.377872  -0.597290   2.415058
H   3.268837   0.612416  -1.492891
H   3.411923  -1.115464  -1.152369
S   5.263414   0.038631  -0.207011
H   2.276839  -1.794812   1.287617
H   3.767555  -1.080123   1.648532
H   5.193235   1.325588   0.190612
C  -3.435862  -1.416451   0.061783
C  -2.494607  -0.169287  -0.001177
O  -3.367684  -2.195771  -0.922263
O  -4.122753  -1.519648   1.098146
H  -1.643694  -0.389534   0.644944
C  -3.179558   1.111398   0.450897
N  -1.925831  -0.052275  -1.382917
H  -0.924124   0.282071  -1.331444
```

```
H  -3.481016   0.982220   1.487190
H  -4.076274   1.303114  -0.142416
S  -2.137217   2.633227   0.274890
H  -1.989316  -0.994861  -1.790777
H  -2.470762   0.580031  -1.968531
H  -0.946349   2.054036   0.536377
28
Dimer 122 of the z...z  type
C  -0.993872   0.310360  -1.318638
C  -2.457646   0.315700  -0.809753
O  -0.494937  -0.833812  -1.536988
O  -0.433578   1.413003  -1.390986
H  -3.038862   1.061299  -1.349565
C  -2.464584   0.613386   0.691535
N  -3.081347  -1.028043  -1.079448
H  -3.345840  -1.126264  -2.060592
H  -1.992235   1.581419   0.845314
H  -1.889033  -0.138729   1.235229
S  -4.139298   0.593910   1.457990
H  -2.387346  -1.758937  -0.883586
H  -3.918548  -1.168019  -0.502135
H  -4.636012   1.639127   0.765089
C   3.019632   1.398747   0.412809
C   2.114560   0.147970   0.159051
O   3.390074   2.016034  -0.616733
O   3.241023   1.664050   1.611607
H   1.092859   0.460010   0.372343
C   2.491693  -1.039903   1.031188
N   2.136221  -0.190192  -1.300768
H   1.190694  -0.539653  -1.597105
H   2.367203  -0.744685   2.069861
H   3.537903  -1.314336   0.879400
S   1.525740  -2.581075   0.683391
H   2.371273   0.684046  -1.788435
H   2.852261  -0.881715  -1.521565
H   0.349146  -1.983034   0.402799
28
Dimer 123 of the z...z  type
C  -0.978533   0.736557  -1.184279
C  -2.445883   0.632968  -0.694256
O  -0.510693  -0.318350  -1.710562
O  -0.386291   1.796321  -0.943337
H  -2.995728   1.537922  -0.943915
C  -2.438586   0.407613   0.820080
N  -3.114266  -0.519308  -1.395226
H  -3.433224  -0.254952  -2.328292
H  -1.938243   1.255245   1.284173
H  -1.880885  -0.496579   1.072994
S  -4.107407   0.169682   1.560037
H  -2.418325  -1.267192  -1.513888
H  -3.923350  -0.859306  -0.862692
H  -4.581479   1.397639   1.265724
C   3.174479   1.134206   0.651489
C   2.142099   0.060397   0.172575
O   3.642817   1.873516  -0.250563
O   3.390104   1.159000   1.879642
H   1.156011   0.438970   0.441963
C   2.372305  -1.301968   0.809452
N   2.158450  -0.000080  -1.325572
H   1.180921  -0.139169  -1.686992
H   2.258287  -1.191833   1.884847
H   3.386002  -1.656246   0.609840
S   1.255332  -2.639054   0.181701
H   2.551941   0.899863  -1.634297
H   2.761763  -0.745893  -1.671229
H   0.157430  -1.875348   0.007717
28
Dimer 124 of the z...z  type
C  -1.802326  -1.533412  -0.267946
```



```
C  -2.126218  -0.162707  -0.944075
O  -2.525348  -2.488228  -0.607069
O  -0.835458  -1.539862   0.534716
H  -1.465668  -0.067494  -1.808921
C  -1.958461   1.059065  -0.050697
N  -3.530457  -0.259538  -1.477217
H  -3.695708   0.325287  -2.296702
H  -2.258666   1.966037  -0.578010
H  -0.909945   1.171150   0.205661
S  -2.973366   1.006279   1.484962
H  -3.688458  -1.254994  -1.698447
H  -4.203648  -0.001988  -0.747098
H  -2.355301  -0.070768   2.012548
C   3.267079  -1.188430   0.039843
C   2.128436  -0.119860  -0.086887
O   3.421587  -1.683586   1.184899
O   3.901683  -1.407246  -1.009466
H   1.461495  -0.417591  -0.895125
C   2.731481   1.251261  -0.377013
N   1.335893  -0.130695   1.185334
H   0.422258  -0.628087   1.025173
H   3.323574   1.180828  -1.286638
H   3.389622   1.560615   0.438003
S   1.491940   2.609273  -0.536710
H   1.911936  -0.671919   1.852635
H   1.148370   0.803281   1.551179
H   0.845250   2.104308  -1.607107
28
Dimer 125 of the z...z  type
C   2.596916  -1.727874  -0.374186
C   2.771213  -0.176025  -0.434442
O   1.877261  -2.163139   0.556003
O   3.236181  -2.358714  -1.242522
H   2.708631   0.155944  -1.470179
C   4.131453   0.193780   0.155206
N   1.661616   0.477507   0.316897
H   0.744799   0.445856  -0.218017
H   4.905713  -0.332316  -0.399792
H   4.188699  -0.113303   1.201881
S   4.495940   2.004093   0.161758
H   1.467865  -0.049537   1.174655
H   1.880588   1.448944   0.548648
H   4.486154   2.165887  -1.177311
C  -1.441379  -0.012326   0.271705
C  -2.932505  -0.365232   0.029016
O  -1.053559   0.044015   1.455601
O  -0.783040   0.218546  -0.776555
H  -3.012679  -1.149453  -0.722162
C  -3.692880   0.885168  -0.415018
N  -3.509865  -0.903566   1.310183
H  -3.234492  -1.874497   1.464236
H  -3.240211   1.245970  -1.336226
H  -3.608998   1.670883   0.338624
S  -5.501920   0.628248  -0.652383
H  -3.129553  -0.359693   2.094079
H  -4.534890  -0.854549   1.302537
H  -5.399840  -0.245784  -1.674450
28
Dimer 126 of the z...z  type
C  -1.636072  -2.033373   0.111249
C  -2.062133  -0.645178  -0.457610
O  -2.503528  -2.925618   0.092403
O  -0.466422  -2.094395   0.567139
H  -1.316988  -0.276060  -1.159838
C  -2.246424   0.347347   0.695212
N  -3.349140  -0.837152  -1.211394
H  -3.180055  -1.106904  -2.180722
H  -1.272199   0.542701   1.137998
H  -2.899124  -0.072344   1.462917
```

```
S  -3.034073   1.942811   0.222428
H  -3.848001  -1.619066  -0.761185
H  -3.916189   0.019134  -1.207615
H  -2.071179   2.356237  -0.624562
C   3.537442  -0.750009  -0.339455
C   2.178770   0.003430  -0.129951
O   3.917810  -1.461418   0.624910
O   4.090625  -0.543252  -1.436045
H   1.504120  -0.272262  -0.940053
C   2.409728   1.510247  -0.125064
N   1.573104  -0.475449   1.154627
H   0.727738  -1.078942   0.966788
H   2.917573   1.786570  -1.046150
H   3.045603   1.798630   0.715016
S   0.870640   2.513415   0.057746
H   2.317174  -1.045738   1.594430
H   1.302941   0.284108   1.778802
H   0.340003   2.175206  -1.134769
28
Dimer 127 of the z...z  type
C  -2.510542  -1.710946   0.406458
C  -2.731486  -0.163478   0.416889
O  -1.841191  -2.162215  -0.553480
O  -3.068945  -2.324684   1.339940
H  -2.612466   0.212403   1.432432
C  -4.139658   0.138260  -0.094050
N  -1.694316   0.489601  -0.433249
H  -0.760328   0.551238   0.071012
H  -4.857220  -0.386492   0.533661
H  -4.255554  -0.215661  -1.120874
S  -4.563309   1.934388  -0.151757
H  -1.509025  -0.101451  -1.250847
H  -1.979125   1.425144  -0.731189
H  -4.460366   2.157371   1.174527
C   1.430583   0.040867  -0.312177
C   2.898482  -0.329651   0.024979
O   1.085144  -0.026404  -1.508112
O   0.746117   0.411488   0.677867
H   2.927219  -0.992997   0.887081
C   3.697813   0.944596   0.301178
N   3.479466  -1.076589  -1.145013
H   3.193295  -2.056495  -1.141092
H   3.232332   1.457715   1.140249
H   3.670843   1.609818  -0.564445
S   5.485241   0.659552   0.644308
H   3.107574  -0.660715  -2.008459
H   4.504772  -1.040588  -1.138143
H   5.310930  -0.056791   1.773733
28
Dimer 128 of the z...z  type
C   3.714335  -1.031530  -0.424610
C   2.462504  -0.131547  -0.158361
O   4.184831  -1.624082   0.578491
O   4.116535  -1.033390  -1.604869
H   1.679484  -0.386082  -0.871613
C   2.842450   1.338956  -0.300129
N   1.930401  -0.445668   1.205793
H   1.121171  -1.095545   1.102034
H   3.274603   1.485008  -1.287876
H   3.587411   1.620026   0.447530
S   1.436467   2.508285  -0.049444
H   2.698623  -0.885295   1.730994
H   1.550897   0.372319   1.689458
H   0.637555   1.988421  -1.003646
C  -1.110678  -0.381104   0.776443
C  -2.322849   0.044103  -0.094426
O  -0.455775  -1.376334   0.347952
O  -0.916690   0.279532   1.807214
H  -2.337014   1.126928  -0.201921
```



```
C  -3.615647  -0.453112   0.552665
N  -2.158169  -0.551964  -1.467155
H  -1.538047   0.011941  -2.049625
H  -3.686035  -0.015885   1.546749
H  -3.597818  -1.540129   0.656043
S  -5.139219  -0.079057  -0.413909
H  -1.711220  -1.471974  -1.355590
H  -3.059779  -0.638443  -1.949211
H  -5.033303   1.264081  -0.349929
28
Dimer 129 of the z...z  type
C   2.177515  -1.235026   0.466668
C   2.587608   0.231791   0.827048
O   3.054249  -1.908847  -0.128331
O   1.037245  -1.571901   0.838044
H   2.767207   0.258435   1.902942
C   1.532946   1.273294   0.478069
N   3.904844   0.507145   0.148856
H   4.552750   1.058427   0.710268
H   1.831071   2.264502   0.823229
H   0.598331   1.001504   0.964138
S   1.250936   1.462331  -1.337152
H   4.291186  -0.430228  -0.067234
H   3.750622   0.983906  -0.746465
H   0.837022   0.197213  -1.560672
C  -2.199753   0.306890   1.406199
C  -2.930139   0.454173   0.028025
O  -1.471946   1.283448   1.724210
O  -2.451554  -0.731006   2.040815
H  -3.940659   0.805226   0.248514
C  -3.022384  -0.833884  -0.778737
N  -2.244086   1.559040  -0.727269
H  -2.866621   2.097969  -1.328199
H  -3.643389  -0.696718  -1.665295
H  -3.481291  -1.586121  -0.140910
S  -1.407709  -1.461235  -1.408415
H  -1.813264   2.154407   0.001025
H  -1.473598   1.188154  -1.296510
H  -0.745023  -1.534481  -0.228418
28
Dimer 130 of the z...z  type
C  -1.862647   1.140563   0.848075
C  -2.883526  -0.032052   0.665706
O  -2.202097   2.231027   0.330285
O  -0.850915   0.868841   1.525218
H  -3.441596  -0.111970   1.600705
C  -2.248629  -1.379117   0.342217
N  -3.859038   0.391264  -0.398219
H  -4.813097   0.069291  -0.240689
H  -2.997946  -2.171976   0.344752
H  -1.508580  -1.599198   1.107723
S  -1.468094  -1.448169  -1.325363
H  -3.805590   1.425251  -0.413871
H  -3.534973   0.049009  -1.311960
H  -0.554566  -0.453264  -1.157958
C   1.861902   1.141876  -0.846832
C   2.883402  -0.030286  -0.665018
O   2.200692   2.232232  -0.328386
O   0.850386   0.869974  -1.524228
H   3.441566  -0.109431  -1.600027
C   2.249207  -1.377858  -0.342247
N   3.858626   0.393008   0.399180
H   4.812880   0.071682   0.241511
H   2.998946  -2.170316  -0.345161
H   1.509293  -1.597929  -1.107888
S   1.468633  -1.448152   1.325265
H   3.804575   1.426955   0.415394
H   3.534724   0.050067   1.312721
H   0.554584  -0.453654   1.158271
```

```
28
Dimer 131 of the z...z  type
C   1.861950   1.481505  -0.876693
C   1.850937  -0.025025  -0.482284
O   2.957096   1.940830  -1.269883
O   0.766037   2.066651  -0.719945
H   0.996541  -0.533889  -0.924427
C   1.811317  -0.163566   1.036897
N   3.096996  -0.672262  -1.028859
H   2.974089  -0.957954  -2.000785
H   0.988958   0.440507   1.412044
H   2.730634   0.217786   1.486219
S   1.638031  -1.908732   1.609988
H   3.839706   0.041309  -1.017438
H   3.367529  -1.498653  -0.486646
H   0.564968  -2.184061   0.824554
C  -1.861924  -1.481539  -0.876678
C  -1.850925   0.024980  -0.482256
O  -2.956971  -1.940818  -1.270103
O  -0.766065  -2.066722  -0.719741
H  -0.996514   0.533839  -0.924382
C  -1.811347   0.163572   1.036918
N  -3.096928   0.672280  -1.028872
H  -2.974165   0.957416  -2.000974
H  -0.988982  -0.440465   1.412106
H  -2.730662  -0.217786   1.486238
S  -1.638119   1.908767   1.609948
H  -3.839902  -0.040966  -1.016942
H  -3.367038   1.499026  -0.487011
H  -0.565050   2.184108   0.824542
28
Dimer 132 of the z...z  type
C   1.643706   1.462882   0.048602
C   2.955146   0.615527   0.106324
O   1.150886   1.765301   1.166165
O   1.242752   1.762057  -1.090474
H   3.785138   1.305214  -0.059134
C   3.027191  -0.510241  -0.919518
N   3.085384   0.099946   1.514369
H   4.046190   0.049340   1.851184
H   4.013094  -0.976833  -0.911582
H   2.850998  -0.079535  -1.902567
S   1.825743  -1.877307  -0.619228
H   2.515001   0.748886   2.086440
H   2.653508  -0.829655   1.583650
H   0.684598  -1.240978  -1.011013
C  -1.943276  -0.012641  -1.349688
C  -1.677949   0.765857  -0.026894
O  -2.903256   0.406873  -2.039758
O  -1.158015  -0.954197  -1.595065
H  -0.891692   1.491039  -0.239272
C  -1.246743  -0.093826   1.150220
N  -2.927792   1.544368   0.289270
H  -2.748908   2.418427   0.783253
H  -1.084306   0.519229   2.036176
H  -0.304572  -0.571290   0.904919
S  -2.480399  -1.383628   1.617912
H  -3.384657   1.716927  -0.621173
H  -3.570931   0.973642   0.848331
H  -2.404420  -2.061961   0.453963
28
Dimer 133 of the z...z  type
C   1.424365  -1.426893  -0.089091
C   2.685685  -0.808852   0.594896
O   1.604919  -1.950188  -1.203570
O   0.364574  -1.356965   0.582495
H   3.050982  -1.553734   1.304828
C   2.420292   0.494147   1.338329
N   3.743686  -0.652643  -0.462558
```



```
H   4.693094  -0.783304  -0.113401
H   3.303628   0.821343   1.887897
H   1.624371   0.307894   2.056000
S   1.981122   1.912156   0.239182
H   3.522063  -1.348358  -1.192898
H   3.677267   0.278340  -0.891240
H   1.028142   1.278686  -0.497197
C  -3.120283  -0.586553   0.538566
C  -2.416354   0.813199   0.478550
O  -2.962832  -1.195847   1.623559
O  -3.785020  -0.914263  -0.464116
H  -3.091877   1.515990   0.974487
C  -2.143144   1.356581  -0.915827
N  -1.181464   0.740538   1.334852
H  -0.678210   1.628344   1.378545
H  -1.802355   2.392790  -0.868538
H  -3.086910   1.322287  -1.455723
S  -0.854555   0.471996  -1.897839
H  -1.464503   0.461755   2.276640
H  -0.531060  -0.018504   1.003542
H  -1.075774  -0.767903  -1.418038
28
Dimer 134 of the z...z  type
C   1.935206  -0.136302  -1.334927
C   1.691059  -0.771991   0.066493
O   2.878527  -0.635254  -1.994132
O   1.154200   0.783154  -1.664104
H   0.920846  -1.533212  -0.063381
C   1.244477   0.195436   1.149938
N   2.959293  -1.485083   0.456720
H   2.801272  -2.294882   1.056219
H   1.111636  -0.324043   2.098527
H   0.284504   0.613715   0.869406
S   2.440702   1.565089   1.459658
H   3.407360  -1.761942  -0.431733
H   3.598375  -0.839340   0.932480
H   2.323316   2.112751   0.231954
C  -1.637211  -1.456249   0.161313
C  -2.949607  -0.607937   0.139793
O  -1.153481  -1.666027   1.304208
O  -1.227091  -1.847093  -0.945854
H  -3.777601  -1.308492   0.015897
C  -3.008129   0.438032  -0.968179
N  -3.095784   0.010426   1.504364
H  -4.058331   0.068212   1.834397
H  -3.993080   0.905116  -1.004720
H  -2.823093  -0.065768  -1.914155
S  -1.806171   1.821310  -0.758752
H  -2.515581  -0.585874   2.123450
H  -2.680938   0.950049   1.506125
H  -0.664407   1.152890  -1.092720
28
Dimer 135 of the z...z  type
C  -3.230873   0.677137   0.333952
C  -3.041214  -0.595240  -0.558764
O  -3.269963   1.756709  -0.304324
O  -3.370497   0.478837   1.558068
H  -4.035440  -0.841381  -0.943946
C  -2.512377  -1.832242   0.152424
N  -2.211498  -0.209105  -1.748799
H  -2.103474  -0.976936  -2.411435
H  -2.534565  -2.699367  -0.510638
H  -3.173834  -2.024369   0.994743
S  -0.775318  -1.726625   0.754143
H  -2.674560   0.573761  -2.214865
H  -1.250066   0.157951  -1.481986
H  -0.882758  -0.510615   1.328885
C   0.792459   1.149616  -0.289974
C   2.331106   0.931082  -0.294363
```

```
O   0.287562   1.524456   0.784969
O   0.232095   0.852621  -1.374606
H   2.773519   1.337644  -1.201254
C   2.613983  -0.566831  -0.173762
N   2.917393   1.668042   0.879176
H   3.055938   2.657609   0.671097
H   2.132864  -1.071418  -1.008545
H   2.187111  -0.962386   0.749527
S   4.402905  -1.002479  -0.109237
H   2.234432   1.614026   1.647740
H   3.819446   1.269906   1.161751
H   4.716658  -0.553447  -1.342155
28
Dimer 136 of the z...z  type
C   2.070672  -1.205190   0.535712
C   2.620188   0.227284   0.838088
O   2.841669  -1.959197  -0.105658
O   0.932595  -1.437173   0.989382
H   2.865852   0.260515   1.900854
C   1.631441   1.342345   0.522332
N   3.911890   0.382825   0.077422
H   4.629970   0.901120   0.581885
H   2.021436   2.312765   0.833096
H   0.700144   1.146743   1.049981
S   1.273003   1.522856  -1.279736
H   4.217793  -0.584079  -0.131927
H   3.736739   0.844014  -0.822730
H   0.728793   0.298423  -1.451235
C  -2.182766   0.397856   1.343269
C  -2.926253   0.406221  -0.035401
O  -1.429153   1.386983   1.542972
O  -2.457214  -0.552437   2.094169
H  -3.948820   0.730763   0.166623
C  -2.968756  -0.946425  -0.736521
N  -2.282369   1.471011  -0.880561
H  -2.941861   2.033202  -1.416349
H  -3.630516  -0.915653  -1.603157
H  -3.354570  -1.672999  -0.024683
S  -1.343940  -1.522563  -1.390730
H  -1.754050   2.051548  -0.199378
H  -1.599071   1.055196  -1.522506
H  -0.646471  -1.539553  -0.223096
28
Dimer 137 of the z...z  type
C  -2.053698   0.670475   1.178990
C  -2.395492  -0.800278   0.777038
O  -3.033779   1.422535   1.378280
O  -0.832758   0.921276   1.276068
H  -2.274803  -1.416095   1.670448
C  -1.528168  -1.364085  -0.340385
N  -3.856958  -0.835816   0.412990
H  -4.313012  -1.722413   0.627357
H  -1.811553  -2.392973  -0.568367
H  -0.486248  -1.367934  -0.026168
S  -1.703119  -0.452551  -1.935633
H  -4.295746  -0.055069   0.927218
H  -3.970408  -0.637550  -0.587761
H  -1.226855   0.729932  -1.492712
C   2.578249  -1.486054   0.290646
C   2.100404  -0.008243   0.423960
O   3.808727  -1.679650   0.454862
O   1.677645  -2.297345  -0.001318
H   1.212365   0.050983   1.048803
C   1.799679   0.559405  -0.959644
N   3.199780   0.782280   1.086089
H   3.126298   0.748251   2.102842
H   1.074094  -0.093626  -1.439169
H   2.699524   0.576094  -1.578401
S   1.167635   2.293770  -0.931531
```



```
H   4.084175   0.317037   0.828450
H   3.193380   1.765724   0.799117
H   0.192252   2.051024  -0.017357
28
Dimer 138 of the z...z  type
C   2.018568  -1.755235   0.403981
C   1.976145  -0.198783   0.267374
O   3.159412  -2.251977   0.575077
O   0.913830  -2.318559   0.287389
H   1.226918   0.224673   0.933467
C   1.660989   0.170976  -1.178499
N   3.325798   0.332442   0.675313
H   3.344391   0.599671   1.659424
H   0.719834  -0.306742  -1.443633
H   2.427176  -0.212653  -1.855146
S   1.574438   1.991301  -1.490254
H   3.974956  -0.466546   0.557010
H   3.615644   1.143932   0.122512
H   0.805499   2.266482  -0.406830
C  -1.485182   1.227097   1.491926
C  -2.357558   0.592035   0.366455
O  -0.473329   1.871097   1.108200
O  -1.864183   0.975718   2.647994
H  -3.413731   0.745729   0.580241
C  -2.042448  -0.901833   0.278334
N  -2.076339   1.274509  -0.947841
H  -2.538275   2.183276  -0.999155
H  -2.339652  -1.358712   1.220323
H  -0.974430  -1.088673   0.146459
S  -2.855923  -1.775297  -1.124882
H  -1.069883   1.449649  -1.045356
H  -2.401510   0.697517  -1.733333
H  -4.128319  -1.598096  -0.712825
28
Dimer 139 of the z...z  type
C  -1.714411   1.120772  -0.948627
C  -1.574666   0.444891   0.450808
O  -2.111939   2.312593  -0.933868
O  -1.382670   0.410340  -1.918291
H  -0.519183   0.533357   0.716164
C  -2.013806  -1.009880   0.492341
N  -2.338298   1.289449   1.434738
H  -1.938938   1.286764   2.372642
H  -1.872420  -1.437747   1.485667
H  -1.405124  -1.570261  -0.211267
S  -3.797948  -1.254031   0.084375
H  -2.340627   2.240835   1.030190
H  -3.314915   0.979811   1.486223
H  -3.702826  -0.802881  -1.183750
C   2.534509   1.091682   0.466784
C   2.696197   0.141844  -0.769127
O   3.607839   1.414099   1.031059
O   1.366950   1.436091   0.734140
H   2.713102   0.775180  -1.658568
C   1.602902  -0.905263  -0.922257
N   4.060355  -0.487980  -0.656594
H   4.502487  -0.680577  -1.554998
H   1.803861  -1.554892  -1.776203
H   0.654767  -0.403200  -1.102934
S   1.459956  -2.034297   0.529646
H   4.619887   0.181016  -0.099101
H   4.002941  -1.360863  -0.121318
H   1.138567  -1.074201   1.420650
28
Dimer 140 of the z...z  type
C   1.349234   1.408715   0.132048
C   2.845159   0.987516  -0.025309
O   0.965058   1.615174   1.307909
O   0.717391   1.536036  -0.936765
```

```
H   3.390420   1.880008  -0.338178
C   3.090260  -0.131825  -1.034321
N   3.345794   0.619403   1.343562
H   4.319410   0.869444   1.511241
H   4.158348  -0.253445  -1.219685
H   2.601684   0.146938  -1.965429
S   2.487386  -1.783069  -0.480339
H   2.709969   1.101844   2.002661
H   3.226835  -0.393168   1.483711
H   1.139310  -1.558289  -0.605051
C  -1.349095  -1.408050   0.133637
C  -2.845137  -0.987136  -0.023375
O  -0.964287  -1.613258   1.309508
O  -0.717790  -1.536423  -0.935370
H  -3.390477  -1.879969  -0.335117
C  -3.090798   0.131205  -1.033358
N  -3.345109  -0.617758   1.345406
H  -4.318526  -0.867993   1.513947
H  -4.158981   0.252602  -1.218321
H  -2.602669  -0.148462  -1.964429
S  -2.487713   1.782998  -0.481280
H  -2.708698  -1.099310   2.004607
H  -3.226429   0.394994   1.484465
H  -1.139686   1.558030  -0.606143
28
Dimer 141 of the z...z  type
C  -1.820197   1.193195  -0.689414
C  -2.687810   0.726271   0.526179
O  -2.400118   1.170174  -1.803201
O  -0.664785   1.560003  -0.407318
H  -2.979775   1.624021   1.073477
C  -1.964359  -0.223523   1.474338
N  -3.943515   0.113645  -0.031381
H  -4.784156   0.329658   0.502653
H  -2.564124  -0.418401   2.364547
H  -1.037238   0.256551   1.781396
S  -1.619979  -1.885202   0.751942
H  -4.003330   0.477886  -1.000573
H  -3.835295  -0.906314  -0.091939
H  -0.832768  -1.494269  -0.284937
C   1.773907  -1.560406  -0.838611
C   1.794321  -0.347982   0.138993
O   2.774357  -2.313666  -0.785514
O   0.777704  -1.617944  -1.591227
H   0.804120  -0.142256   0.531055
C   2.314144   0.889181  -0.587302
N   2.694174  -0.692595   1.296078
H   2.216660  -1.286304   1.975060
H   1.631022   1.097744  -1.406598
H   3.318173   0.718654  -0.981095
S   2.423676   2.381765   0.489953
H   3.484787  -1.233410   0.919901
H   3.021391   0.151929   1.778022
H   1.089457   2.483202   0.668813
28
Dimer 142 of the z...z  type
C   1.230973   1.496156   0.079550
C   2.595125   0.983587  -0.473483
O   1.208126   1.833421   1.287276
O   0.323019   1.552299  -0.775560
H   2.919031   1.703822  -1.224846
C   2.519761  -0.410874  -1.115367
N   3.586415   1.029744   0.656946
H   4.494651   1.413041   0.400446
H   3.358291  -0.562345  -1.795158
H   1.581621  -0.478126  -1.665583
S   2.584911  -1.805012   0.087358
H   3.129011   1.596137   1.393336
H   3.705164   0.075760   1.032554
```



```
H   1.290733  -1.667869   0.534915
C  -1.112588  -1.331653  -0.154608
C  -2.650569  -1.126224   0.020736
O  -0.460735  -1.402878   0.928143
O  -0.708931  -1.449780  -1.318370
H  -3.126391  -2.068105  -0.256757
C  -3.226981   0.002565  -0.833831
N  -2.919679  -0.900585   1.484035
H  -3.765615  -1.354129   1.826249
H  -4.316533  -0.043972  -0.849624
H  -2.854776  -0.127651  -1.848078
S  -2.810720   1.689529  -0.215155
H  -2.077055  -1.249242   1.972944
H  -2.980360   0.113157   1.658836
H  -1.445855   1.617516  -0.388640
28
Dimer 143 of the z...z  type
C  -1.927777  -0.707858  -1.193308
C  -2.763098  -0.459158   0.108261
O  -1.242013  -1.764204  -1.186430
O  -2.065553   0.139099  -2.090170
H  -3.763854  -0.854358  -0.079270
C  -2.874972   1.000437   0.523026
N  -2.166863  -1.303107   1.201795
H  -2.828853  -1.550712   1.937676
H  -3.528817   1.114415   1.388656
H  -3.297982   1.553050  -0.312546
S  -1.271005   1.769386   1.004308
H  -1.798160  -2.147863   0.740971
H  -1.368286  -0.812643   1.624786
H  -0.647765   1.653015  -0.192319
C   1.927417   0.707500  -1.193636
C   2.763069   0.459320   0.107824
O   1.241443   1.763715  -1.186974
O   2.065161  -0.139712  -2.090245
H   3.763721   0.854593  -0.080106
C   2.875222  -1.000157   0.522950
N   2.167070   1.303452   1.201332
H   2.829113   1.550752   1.937279
H   3.529206  -1.113867   1.388512
H   3.298140  -1.552958  -0.312542
S   1.271363  -1.769152   1.004672
H   1.798596   2.148387   0.740743
H   1.368333   0.813187   1.624287
H   0.647969  -1.652856  -0.191835
28
Dimer 144 of the z...z  type
C   1.241245   1.973967   0.658558
C   1.681383   0.761139  -0.217292
O   2.003879   2.970635   0.622489
O   0.203954   1.794201   1.328177
H   0.838756   0.331758  -0.752241
C   2.332824  -0.301473   0.667326
N   2.666588   1.256583  -1.243767
H   2.194972   1.657127  -2.054766
H   1.582751  -0.648448   1.374207
H   3.180203   0.119123   1.212293
S   2.975903  -1.751274  -0.273410
H   3.204097   2.014856  -0.799901
H   3.279334   0.498519  -1.565442
H   1.743385  -2.125061  -0.721137
C  -0.801096  -1.744851  -0.013871
C  -2.201559  -1.071156  -0.151027
O  -0.410173  -1.967136   1.153932
O  -0.204452  -1.932555  -1.097006
H  -2.814457  -1.616953  -0.865668
C  -2.020701   0.383736  -0.583235
N  -2.877962  -1.140796   1.192379
H  -3.395639  -2.011794   1.310311
```

```
H  -1.526715   0.385894  -1.552957
H  -1.387996   0.917408   0.131389
S  -3.583603   1.353056  -0.675442
H  -2.124133  -1.138554   1.897001
H  -3.528189  -0.360818   1.333398
H  -4.181804   0.608582  -1.628626
28
Dimer 145 of the z...z  type
C   0.687963   1.833525  -0.088389
C   1.623990   0.611754  -0.306266
O  -0.009653   2.133746  -1.098213
O   0.643922   2.311209   1.057094
H   1.005197  -0.270520  -0.117909
C   2.847825   0.533454   0.583912
N   1.987177   0.548085  -1.767820
H   1.154871   0.850144  -2.291903
H   2.523909   0.678192   1.611769
H   3.580060   1.307924   0.346631
S   3.729148  -1.085260   0.421130
H   2.744719   1.193676  -2.000307
H   2.275783  -0.389874  -2.052198
H   2.648762  -1.849085   0.694967
C  -1.036346  -2.118057   0.104577
C  -1.721866  -0.779284  -0.323478
O  -1.781608  -3.123821   0.125113
O   0.168743  -2.017271   0.421548
H  -1.110031  -0.225029  -1.032546
C  -1.990074   0.079662   0.914062
N  -3.016706  -1.115433  -1.012063
H  -2.872535  -1.334645  -1.997992
H  -1.036281   0.317621   1.381407
H  -2.600181  -0.470087   1.633593
S  -2.901981   1.640488   0.556439
H  -3.385426  -1.966260  -0.563623
H  -3.683823  -0.337631  -0.948012
H  -1.913171   2.186142  -0.196637
28
Dimer 146 of the z...z  type
C   2.259487  -0.774060   1.090506
C   2.875317   0.477481   0.377923
O   2.835435  -1.864381   0.845837
O   1.295095  -0.535945   1.839090
H   3.446356   1.022859   1.131206
C   1.839398   1.412159  -0.234977
N   3.856029  -0.030544  -0.644128
H   4.710884   0.520195  -0.711682
H   2.305177   2.328661  -0.599670
H   1.128172   1.674771   0.545125
S   0.962685   0.706139  -1.695096
H   4.052996  -1.005467  -0.346401
H   3.409128  -0.073194  -1.567879
H   0.428866  -0.403656  -1.107187
C  -1.939962  -1.694645  -0.345130
C  -2.280787  -0.173167  -0.372792
O  -2.851950  -2.453035   0.055584
O  -0.772189  -1.968671  -0.696315
H  -1.934503   0.265221  -1.305445
C  -1.640115   0.519515   0.824581
N  -3.778539  -0.014230  -0.325046
H  -4.198902  -0.136400  -1.247282
H  -0.601078   0.208601   0.911462
H  -2.141645   0.224088   1.749076
S  -1.751013   2.358516   0.759548
H  -4.143704  -0.768200   0.273348
H  -4.057293   0.904823   0.030496
H  -1.064791   2.489197  -0.396407
28
Dimer 147 of the z...z  type
C  -1.632765  -1.357942  -0.597534
```



```
C  -3.045317  -0.977172  -0.037351
O  -0.893854  -1.977415   0.209830
O  -1.405663  -1.014148  -1.770730
H  -3.738609  -1.757230  -0.359300
C  -3.566944   0.376764  -0.498434
N  -2.960092  -1.065941   1.463396
H  -3.823829  -1.372310   1.910526
H  -4.551369   0.582165  -0.075785
H  -3.648528   0.357979  -1.582473
S  -2.495043   1.794565  -0.000126
H  -2.185325  -1.729744   1.644492
H  -2.686503  -0.160617   1.859109
H  -1.474030   1.481503  -0.820108
C   3.194735  -0.612346  -0.588992
C   1.983121  -0.603308   0.397248
O   4.304498  -0.891951  -0.065420
O   2.924716  -0.364046  -1.778967
H   1.480106  -1.570207   0.331065
C   0.947165   0.470422   0.120215
N   2.546613  -0.486860   1.789410
H   1.988782  -0.960265   2.499396
H   0.138439   0.416813   0.846653
H   0.533148   0.283046  -0.866549
S   1.592346   2.193400   0.209409
H   3.498466  -0.887450   1.728571
H   2.645440   0.500239   2.049001
H   2.439180   2.069937  -0.833666
28
Dimer 148 of the z...z  type
C  -2.178932   1.367349   0.383334
C  -3.067688   0.180804   0.888907
O  -2.730775   2.131212  -0.446353
O  -1.040936   1.428280   0.884215
H  -3.451383   0.463568   1.871004
C  -2.321792  -1.141943   1.004852
N  -4.251055   0.081425  -0.035162
H  -5.115820  -0.195470   0.428313
H  -2.955678  -1.916896   1.437849
H  -1.467485  -0.984594   1.659411
S  -1.766078  -1.820309  -0.618005
H  -4.334127   1.020955  -0.463209
H  -4.050579  -0.582787  -0.792062
H  -0.985906  -0.781437  -0.999668
C   1.494646   0.533544  -1.071805
C   1.606063  -0.167898   0.321939
O   0.907345  -0.154887  -1.948978
O   1.956234   1.684490  -1.156063
H   0.653939   0.027632   0.820481
C   2.748999   0.311213   1.201618
N   1.689478  -1.652938   0.075832
H   1.326687  -2.215410   0.845716
H   2.739453  -0.191496   2.169932
H   2.627778   1.379994   1.360513
S   4.419252  -0.008912   0.482440
H   1.149388  -1.839827  -0.780634
H   2.664598  -1.921994  -0.099440
H   4.241026   0.786264  -0.592739
28
Dimer 149 of the z...z  type
C   1.896816   0.826594   1.133758
C   2.207978   0.790221  -0.396406
O   2.875457   1.048185   1.884540
O   0.696576   0.661412   1.437018
H   1.826317   1.718327  -0.827290
C   1.599152  -0.390253  -1.144834
N   3.707065   0.817073  -0.543578
H   4.030694   1.289244  -1.387586
H   1.824017  -0.327513  -2.210413
H   0.522979  -0.385351  -1.006150
```

```
S   2.204330  -2.030624  -0.561547
H   4.065186   1.276890   0.309056
H   4.076490  -0.139570  -0.532389
H   1.327119  -2.115142   0.459649
C  -2.044385  -1.243539   0.495735
C  -2.973268  -0.406046  -0.449761
O  -2.390905  -1.256634   1.704828
O  -1.105107  -1.832449  -0.065970
H  -3.589112  -1.122190  -0.996452
C  -2.230030   0.476180  -1.448065
N  -3.893459   0.394671   0.430519
H  -4.852227   0.452495   0.090532
H  -2.918112   0.898298  -2.181926
H  -1.509317  -0.149989  -1.970353
S  -1.386916   1.926281  -0.681545
H  -3.842539  -0.083065   1.351182
H  -3.516682   1.344307   0.547709
H  -0.579895   1.245165   0.191152
28
Dimer 150 of the z...z  type
C   2.289261   1.566833  -0.450178
C   1.978796   0.059452  -0.198093
O   3.497050   1.853031  -0.635717
O   1.291004   2.313615  -0.401841
H   1.148495  -0.251784  -0.826872
C   1.672396  -0.163935   1.279517
N   3.178972  -0.753281  -0.607772
H   3.162920  -0.962532  -1.606286
H   0.875601   0.516583   1.568381
H   2.545716   0.071863   1.891942
S   1.228035  -1.902047   1.710141
H   4.009932  -0.169478  -0.433172
H   3.229992  -1.640569  -0.097177
H   0.218397  -2.051873   0.815261
C  -1.257219  -1.222319  -1.277855
C  -2.287504  -0.154085  -0.804359
O  -1.273930  -2.297905  -0.623564
O  -0.481033  -0.860988  -2.179672
H  -2.652201   0.440452  -1.638473
C  -1.607810   0.722372   0.245918
N  -3.460496  -0.873233  -0.191733
H  -4.110353  -1.210001  -0.903142
H  -0.731557   1.202480  -0.191069
H  -1.282254   0.103202   1.084943
S  -2.696081   2.006525   0.994066
H  -3.083516  -1.697384   0.298532
H  -3.977318  -0.269979   0.457073
H  -2.886702   2.721024  -0.134047
28
Dimer 151 of the z...z  type
C  -1.276740   1.408517  -0.442374
C  -1.662812   0.396471   0.684123
O  -1.511166   2.614002  -0.176876
O  -0.749059   0.913479  -1.457546
H  -0.772606   0.220638   1.290319
C  -2.172739  -0.948518   0.193202
N  -2.668573   1.084189   1.569405
H  -2.646730   0.766527   2.538026
H  -2.427899  -1.595570   1.033337
H  -1.381144  -1.428209  -0.375177
S  -3.700276  -0.823069  -0.836198
H  -2.453455   2.092149   1.502222
H  -3.614562   0.947554   1.196620
H  -3.122670  -0.121799  -1.833771
C   1.183188  -1.742844   0.590485
C   2.464631  -0.881483   0.342702
O   0.802093  -2.437006  -0.383553
O   0.661557  -1.595877   1.712083
H   3.181825  -1.030292   1.147806
```



```
C   2.069417   0.589143   0.230358
N   3.095555  -1.355372  -0.940321
H   3.746270  -2.124519  -0.780376
H   1.598773   0.883831   1.166021
H   1.346188   0.727427  -0.575271
S   3.461995   1.731535  -0.155700
H   2.322188  -1.729064  -1.513892
H   3.599433  -0.604794  -1.423020
H   4.190932   1.474452   0.950085
28
Dimer 152 of the z...z  type
C   1.462349  -1.330986   1.412270
C   2.356512  -0.658734   0.326060
O   0.452158  -1.950581   0.984964
O   1.823841  -1.131510   2.583566
H   3.407746  -0.837674   0.544180
C   2.062694   0.841626   0.296331
N   2.073120  -1.282498  -1.016769
H   2.560657  -2.172544  -1.123940
H   2.331330   1.251116   1.268256
H   1.003450   1.052248   0.128757
S   2.937730   1.763861  -1.036685
H   1.069098  -1.483903  -1.089422
H   2.363288  -0.658716  -1.779445
H   4.193486   1.526056  -0.603938
C  -1.913907   1.829232   0.377680
C  -1.927689   0.270337   0.276821
O  -3.020664   2.365400   0.631590
O  -0.807186   2.355433   0.152937
H  -1.131028  -0.163478   0.877031
C  -1.770336  -0.145293  -1.183300
N  -3.242756  -0.215396   0.827669
H  -3.179589  -0.425589   1.823743
H  -0.837789   0.277682  -1.551689
H  -2.582072   0.259017  -1.791869
S  -1.801756  -1.973107  -1.452964
H  -3.896971   0.578754   0.720094
H  -3.579481  -1.055107   0.347173
H  -0.913195  -2.261699  -0.470480
28
Dimer 153 of the z...z  type
C  -1.217929  -1.201124   0.126869
C  -2.761980  -1.255039   0.381665
O  -0.517297  -1.000394   1.155022
O  -0.859669  -1.368309  -1.049975
H  -3.043027  -2.308264   0.442502
C  -3.591918  -0.573624  -0.698767
N  -3.005444  -0.656279   1.740736
H  -3.786963  -1.074574   2.244322
H  -4.659279  -0.678404  -0.500228
H  -3.360437  -1.048379  -1.649339
S  -3.281912   1.240308  -0.825079
H  -2.114481  -0.783225   2.252169
H  -3.165596   0.354087   1.655157
H  -1.975157   1.144798  -1.149162
C   3.456803  -0.899310  -0.379081
C   2.454348   0.042938   0.359987
O   4.639730  -0.474237  -0.448172
O   2.963911  -1.940209  -0.851758
H   1.799810  -0.517073   1.023990
C   1.606732   0.786571  -0.669552
N   3.266357   1.012333   1.180309
H   3.483159   0.635399   2.102777
H   0.996830   0.049064  -1.187054
H   2.237109   1.313556  -1.388745
S   0.501562   2.074047   0.057103
H   4.160280   1.127260   0.675593
H   2.779349   1.906565   1.302753
H  -0.182128   1.219861   0.847897
```

```
28
Dimer 154 of the z...z  type
C  -1.204440  -0.065443   0.281099
C  -2.400847  -0.980825   0.694972
O  -0.769078   0.685363   1.197451
O  -0.795145  -0.190630  -0.882916
H  -1.980561  -1.943158   0.994917
C  -3.426640  -1.200206  -0.408540
N  -3.026173  -0.377432   1.924334
H  -3.469203  -1.062128   2.537102
H  -4.233537  -1.852359  -0.071908
H  -2.922983  -1.669229  -1.250355
S  -4.234923   0.358819  -0.973455
H  -2.266482   0.121416   2.413653
H  -3.724536   0.323701   1.651379
H  -3.109147   0.913540  -1.468986
C   3.126856  -1.455508  -0.327847
C   2.397694  -0.187236   0.216751
O   4.353806  -1.525991  -0.054707
O   2.424798  -2.228966  -1.005568
H   1.434217  -0.437192   0.651913
C   2.201383   0.819870  -0.917401
N   3.261920   0.404445   1.299916
H   3.048383   0.007910   2.214639
H   1.492516   0.382652  -1.615342
H   3.146102   1.023268  -1.425885
S   1.560541   2.454262  -0.349055
H   4.231374   0.138605   1.062146
H   3.144441   1.423553   1.350161
H   0.470208   1.928914   0.278691
28
Dimer 155 of the z...z  type
C   3.655915  -0.954043  -0.178932
C   2.631634   0.093930   0.379159
O   4.849680  -0.555571  -0.222802
O   3.169700  -2.036382  -0.546961
H   1.974701  -0.359072   1.120228
C   1.799295   0.647169  -0.772994
N   3.441876   1.181727   1.038083
H   3.534050   1.030630   2.042217
H   1.219087  -0.177193  -1.181064
H   2.435647   1.063848  -1.556083
S   0.666824   2.028852  -0.281501
H   4.385755   1.087468   0.613119
H   3.048880   2.115571   0.892175
H   0.199518   1.432284   0.836800
C  -0.945349  -0.899115   0.407926
C  -2.200880  -0.008294   0.133292
O  -0.618243  -1.640936  -0.547361
O  -0.425245  -0.776610   1.535259
H  -2.160320   0.913236   0.709731
C  -3.465927  -0.797320   0.457443
N  -2.175806   0.366395  -1.322462
H  -1.465965   1.091207  -1.484090
H  -3.441852  -1.073964   1.510407
H  -3.501542  -1.711906  -0.137399
S  -5.036029   0.096287   0.084411
H  -1.892423  -0.469604  -1.850440
H  -3.087350   0.705947  -1.645865
H  -4.858654   1.094508   0.973922
28
Dimer 156 of the z...z  type
C  -3.594891  -0.337852  -0.847114
C  -2.188812  -0.079392  -0.202496
O  -4.360117   0.661240  -0.872002
O  -3.794031  -1.502325  -1.230892
H  -1.415657  -0.407275  -0.893367
C  -2.079580  -0.820282   1.127272
N  -2.067725   1.410387  -0.006232
```



```
H  -1.452713   1.858377  -0.687309
H  -2.237563  -1.879558   0.935880
H  -2.858434  -0.479513   1.813114
S  -0.481955  -0.580953   2.019681
H  -3.043771   1.748344  -0.155999
H  -1.722585   1.667341   0.920422
H   0.298623  -1.304530   1.165315
C   2.593824  -1.497069  -0.432562
C   2.578849  -0.016956   0.065928
O   3.569675  -1.828716  -1.138483
O   1.591553  -2.162110  -0.087980
H   2.374654   0.008108   1.134303
C   1.521554   0.765443  -0.704384
N   3.942060   0.584117  -0.148765
H   4.578144   0.345292   0.613188
H   0.602211   0.190490  -0.669870
H   1.804119   0.880724  -1.752226
S   1.219548   2.465268  -0.063037
H   4.332767   0.161502  -1.002696
H   3.911803   1.604833  -0.225501
H   0.864269   2.067227   1.178400
28
Dimer 157 of the z...z  type
C   1.652040  -1.236903   0.215878
C   3.150833  -0.900694   0.508138
O   1.425749  -1.793877  -0.883736
O   0.855139  -0.921053   1.123432
H   3.508210  -1.617269   1.249512
C   3.366484   0.514761   1.034259
N   3.919474  -1.150520  -0.761431
H   4.845782  -1.549452  -0.614500
H   4.408187   0.674189   1.315776
H   2.738892   0.643775   1.913114
S   2.985001   1.832372  -0.199745
H   3.317772  -1.784063  -1.317744
H   4.010493  -0.277237  -1.294081
H   1.661004   1.580906  -0.303168
C  -3.088479  -1.151034   0.026657
C  -3.426139   0.238999  -0.610058
O  -3.598906  -1.358750   1.158450
O  -2.384303  -1.897688  -0.673896
H  -4.157867   0.060009  -1.399597
C  -2.218226   0.961825  -1.199115
N  -4.108304   1.059641   0.451287
H  -4.875190   1.634695   0.105532
H  -2.525821   1.855496  -1.743586
H  -1.723744   0.280627  -1.888085
S  -1.014803   1.542718   0.071116
H  -4.433544   0.361272   1.146348
H  -3.421344   1.659702   0.923543
H  -0.543231   0.326837   0.460075
28
Dimer 158 of the z...z  type
C  -3.619665   0.392675   0.771487
C  -2.177343   0.118125   0.226891
O  -4.343543  -0.627468   0.892858
O  -3.886725   1.590374   0.973017
H  -1.444598   0.689788   0.792865
C  -2.125319   0.484459  -1.254636
N  -1.895710  -1.349650   0.418702
H  -1.513750  -1.549383   1.343696
H  -2.393760   1.535018  -1.348160
H  -2.852932  -0.105188  -1.815839
S  -0.497123   0.188608  -2.069246
H  -2.820833  -1.811333   0.368771
H  -1.246794  -1.730827  -0.276673
H   0.208737   1.089726  -1.324406
C   2.382376   1.634347   0.331050
C   2.556353   0.135084  -0.072253
```

```
O   3.293392   2.124209   1.030804
O   1.315867   2.150739  -0.071849
H   2.376571   0.017770  -1.138512
C   1.588853  -0.721609   0.736265
N   3.977896  -0.281153   0.191872
H   4.594641  -0.003655  -0.573035
H   0.611276  -0.255532   0.666136
H   1.872340  -0.744317   1.790269
S   1.488639  -2.477167   0.190731
H   4.297796   0.225572   1.028783
H   4.069969  -1.293034   0.319685
H   1.138320  -2.193352  -1.082849
28
Dimer 159 of the z...z  type
C   4.094242   0.219888   0.072639
C   2.759785  -0.543143   0.404953
O   4.250800   1.304594   0.690354
O   4.842016  -0.344782  -0.742844
H   3.023971  -1.428286   0.982986
C   2.011069  -0.942039  -0.857126
N   1.950178   0.357094   1.299860
H   1.597648  -0.109023   2.134195
H   2.679446  -1.544504  -1.467176
H   1.722856  -0.064436  -1.438098
S   0.443472  -1.876258  -0.575783
H   2.626302   1.108769   1.553498
H   1.154202   0.792712   0.807302
H   0.952175  -2.823156   0.238318
C  -4.094344  -0.219598   0.072595
C  -2.759709   0.543067   0.404998
O  -4.251103  -1.304424   0.690013
O  -4.842032   0.345474  -0.742700
H  -3.023664   1.428191   0.983154
C  -2.010935   0.941926  -0.857059
N  -1.950323  -0.357522   1.299773
H  -1.597964   0.108380   2.134306
H  -2.679279   1.544439  -1.467100
H  -1.722770   0.064307  -1.438040
S  -0.443303   1.876096  -0.575738
H  -2.626567  -1.109184   1.553158
H  -1.154232  -0.793070   0.807355
H  -0.951908   2.822959   0.238464
28
Dimer 160 of the z...z  type
C  -3.195143  -0.919512   0.478269
C  -2.722234  -0.131514  -0.792712
O  -4.406533  -0.758477   0.774307
O  -2.331327  -1.623439   1.030944
H  -2.805373  -0.812662  -1.642168
C  -1.300586   0.401608  -0.721327
N  -3.726929   0.968441  -1.021936
H  -3.858649   1.210006  -2.003762
H  -1.052020   0.975070  -1.616357
H  -0.617402  -0.443700  -0.649412
S  -1.026487   1.549461   0.697881
H  -4.604365   0.618932  -0.597769
H  -3.453634   1.811212  -0.506162
H  -1.222015   0.618275   1.654692
C   2.017757  -1.845112  -0.423611
C   2.185834  -0.461592   0.286733
O   3.023617  -2.595331  -0.380998
O   0.920905  -2.016072  -0.987670
H   1.321561  -0.236268   0.907562
C   2.377795   0.624160  -0.772715
N   3.393244  -0.548682   1.180547
H   3.152354  -0.909822   2.103681
H   1.480620   0.660458  -1.386324
H   3.222161   0.380207  -1.420477
S   2.745078   2.298279  -0.095679
```



```
H   4.024076  -1.237319   0.738719
H   3.848676   0.362264   1.300638
H   1.568514   2.439508   0.551159
28
Dimer 161 of the z...z  type
C   3.403334   1.408311   0.166684
C   2.998792   0.047134  -0.494408
O   3.730357   1.337436   1.376936
O   3.307886   2.399470  -0.579340
H   3.467858  -0.054781  -1.470616
C   1.478942  -0.014074  -0.601785
N   3.505071  -1.062177   0.390301
H   4.472995  -1.302291   0.174378
H   1.144793   0.838230  -1.189988
H   1.020964   0.037408   0.388756
S   0.828593  -1.575477  -1.344834
H   3.494911  -0.683359   1.351236
H   2.943739  -1.915411   0.316041
H   1.311304  -1.378516  -2.588484
C  -1.425317  -1.089990   1.188389
C  -2.012852  -0.108256   0.123037
O  -1.892098  -2.252680   1.155221
O  -0.587157  -0.586432   1.962417
H  -1.240221   0.562487  -0.245532
C  -3.158469   0.688752   0.739868
N  -2.496798  -0.920402  -1.043224
H  -1.689348  -1.230403  -1.597214
H  -2.770831   1.236013   1.597346
H  -3.947611   0.019280   1.087694
S  -3.981754   1.856382  -0.427154
H  -2.951004  -1.763269  -0.668693
H  -3.139864  -0.392334  -1.641010
H  -2.903501   2.636901  -0.644229
28
Dimer 162 of the z...z  type
C  -1.165369  -1.515033   0.072451
C  -2.709241  -1.274368   0.186691
O  -0.542717  -1.479666   1.167389
O  -0.744513  -1.764837  -1.069345
H  -3.181456  -2.252589   0.079931
C  -3.288359  -0.324886  -0.861332
N  -2.975872  -0.800487   1.588083
H  -3.810063  -1.199526   2.014975
H  -4.376397  -0.397287  -0.885955
H  -2.894614  -0.624048  -1.830681
S  -2.928490   1.456530  -0.553480
H  -2.113751  -1.062967   2.106752
H  -3.047196   0.226345   1.592645
H  -1.574281   1.440001  -0.766730
C   0.943059   1.647984  -0.073922
C   2.338250   0.966888  -0.217215
O   0.651417   2.066400   1.069445
O   0.259240   1.658764  -1.120505
H   2.840254   1.319737  -1.115949
C   2.155677  -0.546862  -0.245641
N   3.173427   1.369999   0.970558
H   3.640415   2.264829   0.821518
H   1.496900  -0.807595  -1.070170
H   1.654939  -0.893453   0.659351
S   3.729662  -1.500161  -0.349672
H   2.518174   1.495139   1.756936
H   3.888452   0.668060   1.186985
H   4.125466  -1.032754  -1.551732
28
Dimer 163 of the z...z  type
C   4.130553  -0.215325  -0.137490
C   2.758551   0.497367  -0.428764
O   4.264866  -1.345318  -0.674491
O   4.921713   0.424158   0.575111
```

```
H   2.964164   1.371861  -1.044684
C   2.067183   0.911163   0.861685
N   1.934977  -0.460149  -1.248572
H   1.600815  -0.062358  -2.124550
H   2.762969   1.521433   1.432311
H   1.806356   0.040241   1.465591
S   0.489139   1.844475   0.648181
H   2.603782  -1.239744  -1.435304
H   1.123924  -0.838122  -0.732812
H   0.966304   2.802113  -0.172688
C  -4.130780   0.214793  -0.137627
C  -2.758399  -0.497427  -0.428677
O  -4.264932   1.345137  -0.673953
O  -4.922048  -0.425255   0.574242
H  -2.963475  -1.371953  -1.044707
C  -2.067265  -0.911029   0.861904
N  -1.934918   0.460642  -1.247981
H  -1.601030   0.063550  -2.124363
H  -2.762867  -1.521478   1.432577
H  -1.806565  -0.040042   1.465774
S  -0.488924  -1.844013   0.648286
H  -2.604153   1.240276  -1.433934
H  -1.123769   0.838533  -0.732340
H  -0.966147  -2.802177  -0.171942
28
Dimer 164 of the z...z  type
C  -3.589972  -0.335213  -0.869154
C  -2.221298  -0.064802  -0.147521
O  -4.245208   0.696235  -1.170013
O  -3.853458  -1.531233  -1.080581
H  -1.436533  -0.284731  -0.870009
C  -2.048980  -0.905375   1.108801
N  -2.160049   1.412677   0.144888
H  -1.262473   1.851684  -0.098651
H  -2.163599  -1.951186   0.834371
H  -2.829607  -0.663541   1.833527
S  -0.445653  -0.669837   1.994942
H  -2.941605   1.803529  -0.421054
H  -2.344046   1.618823   1.127714
H   0.338442  -1.365654   1.124760
C   2.676754  -1.441139  -0.486633
C   2.595347   0.024727   0.050200
O   3.660961  -1.704428  -1.210732
O   1.714123  -2.163139  -0.147940
H   2.401894   0.009112   1.120527
C   1.491972   0.771626  -0.688509
N   3.927525   0.692995  -0.163548
H   4.571620   0.492893   0.602683
H   0.609197   0.141565  -0.678365
H   1.764296   0.948931  -1.730404
S   1.087397   2.422115   0.027054
H   4.337549   0.276623  -1.012910
H   3.852270   1.709905  -0.252267
H   0.828077   1.957488   1.269786
28
Dimer 165 of the z...z  type
C   2.182088  -1.376693  -0.301908
C   1.912100  -0.306268   0.811170
O   3.124544  -2.169239  -0.055674
O   1.418382  -1.330653  -1.283808
H   1.103113  -0.691216   1.436199
C   1.534251   1.069761   0.282209
N   3.140019  -0.256047   1.679127
H   2.949097  -0.041326   2.657253
H   1.342560   1.765433   1.101073
H   0.636022   0.984342  -0.325041
S   2.859013   1.847460  -0.738700
H   3.581070  -1.186734   1.575163
H   3.800546   0.436477   1.308556
```



```
H   2.808063   0.934057  -1.730606
C  -2.181966   1.376588  -0.301826
C  -1.911779   0.306392   0.811424
O  -3.124379   2.169186  -0.055588
O  -1.418435   1.330345  -1.283852
H  -1.102676   0.691467   1.436226
C  -1.534034  -1.069745   0.282671
N  -3.139539   0.256355   1.679618
H  -2.948440   0.041827   2.657751
H  -1.342195  -1.765255   1.101637
H  -0.635916  -0.984453  -0.324762
S  -2.858991  -1.847646  -0.737832
H  -3.580604   1.187024   1.575550
H  -3.800139  -0.436238   1.309309
H  -2.808225  -0.934438  -1.729928
28
Dimer 166 of the z...z  type
C   2.140337  -0.708250  -1.101971
C   1.910038  -0.613099   0.444388
O   3.158647  -1.356206  -1.448934
O   1.274431  -0.154787  -1.804581
H   1.191015  -1.392626   0.701387
C   1.398260   0.736320   0.929677
N   3.207886  -0.989978   1.106740
H   3.094449  -1.460767   2.003994
H   1.254593   0.732282   2.011569
H   0.448382   0.963884   0.450006
S   2.562181   2.123283   0.577532
H   3.693952  -1.594047   0.421280
H   3.788022  -0.154118   1.238960
H   2.453059   2.041237  -0.764820
C  -2.439899   1.529028  -0.316613
C  -2.364202   0.154274   0.427082
O  -3.559541   1.806009  -0.812730
O  -1.368551   2.160974  -0.357389
H  -1.816642   0.263578   1.361572
C  -1.698431  -0.876317  -0.477418
N  -3.772060  -0.269004   0.755707
H  -4.091197   0.129000   1.639254
H  -0.721512  -0.506978  -0.786062
H  -2.292523  -1.030770  -1.380949
S  -1.536781  -2.551460   0.283823
H  -4.364769   0.129451   0.009004
H  -3.868420  -1.287614   0.808753
H  -0.942109  -2.148457   1.425460
28
Dimer 167 of the z...z  type
C   1.397182  -0.625424   0.629007
C   2.053760   0.256421  -0.486451
O   0.993325   0.010334   1.637189
O   1.320940  -1.840001   0.377931
H   1.300009   0.410817  -1.259875
C   3.295241  -0.360996  -1.114879
N   2.330290   1.601291   0.126639
H   2.132932   2.383186  -0.496160
H   3.698499   0.278272  -1.901367
H   3.011882  -1.317210  -1.548456
S   4.676140  -0.616763   0.081184
H   1.708744   1.629837   0.957906
H   3.298681   1.657528   0.457158
H   4.016480  -1.497818   0.861501
C  -3.507468  -1.275614  -0.299247
C  -3.123636   0.239517  -0.395357
O  -3.992602  -1.634064   0.802642
O  -3.244361  -1.947487  -1.312681
H  -3.470764   0.660081  -1.336749
C  -1.611211   0.378072  -0.256082
N  -3.820035   0.961133   0.728402
H  -4.758779   1.257874   0.461331
```

```
H  -1.142991  -0.200379  -1.050044
H  -1.268354  -0.018824   0.700320
S  -0.994388   2.114896  -0.289201
H  -3.923341   0.264685   1.485099
H  -3.302103   1.787431   1.042359
H  -1.422334   2.415311  -1.532638
28
Dimer 168 of the z...z  type
C  -1.618238  -1.218034  -0.179970
C  -3.133266  -0.951703  -0.458094
O  -1.342420  -1.655208   0.961656
O  -0.857538  -0.966024  -1.137348
H  -3.481723  -1.719483  -1.150418
C  -3.400007   0.425576  -1.058738
N  -3.869458  -1.149402   0.839761
H  -4.773927  -1.607852   0.738026
H  -4.450056   0.539134  -1.331069
H  -2.789594   0.522838  -1.953633
S  -3.040804   1.820838   0.094708
H  -3.225724  -1.707036   1.429382
H  -4.000515  -0.246859   1.311795
H  -1.714958   1.591083   0.223182
C   3.138542  -1.119000  -0.102910
C   3.408143   0.238741   0.629450
O   3.678176  -1.232104  -1.234409
O   2.452376  -1.937363   0.532648
H   4.133058   0.038345   1.420280
C   2.161557   0.874952   1.239409
N   4.071923   1.154805  -0.362893
H   4.798926   1.747133   0.035449
H   2.425426   1.737461   1.853077
H   1.674932   0.129693   1.864818
S   0.969266   1.503769  -0.018724
H   4.449279   0.520113  -1.091094
H   3.362181   1.745069  -0.814377
H   0.534915   0.301220  -0.484506
28
Dimer 169 of the z...z  type
C  -3.369971  -0.586170   0.994684
C  -2.003201  -0.347691   0.263256
O  -4.042673  -1.550645   0.551974
O  -3.617306   0.188732   1.936359
H  -1.235922  -0.850062   0.857536
C  -1.628827   1.119054   0.099957
N  -2.063952  -1.065781  -1.053174
H  -1.151542  -1.460447  -1.319576
H  -0.665203   1.232193  -0.396127
H  -1.566669   1.564910   1.089672
S  -2.826798   2.072973  -0.926940
H  -2.772400  -1.808249  -0.922736
H  -2.401811  -0.439400  -1.790208
H  -3.870330   1.914596  -0.086439
C   3.644976   1.216452   0.210821
C   3.274604  -0.246548  -0.215200
O   3.971418   1.353235   1.415220
O   3.525987   2.066279  -0.689104
H   3.758022  -0.500621  -1.155922
C   1.757673  -0.352014  -0.339087
N   3.792002  -1.178977   0.847807
H   4.761501  -1.443591   0.670644
H   1.436293   0.372003  -1.084691
H   1.280890  -0.110250   0.613086
S   1.133427  -2.029265  -0.789470
H   3.783351  -0.635297   1.727044
H   3.241604  -2.038288   0.930853
H   1.648874  -2.062817  -2.035189
28
Dimer 170 of the z...z  type
C  -3.133648  -0.474732  -1.054847
```



```
C  -2.775052   0.395311   0.196603
O  -3.342986  -1.690199  -0.818199
O  -3.122438   0.137952  -2.137979
H  -3.336050   1.327445   0.187849
C  -1.272006   0.653993   0.196956
N  -3.171201  -0.381349   1.424626
H  -4.150255  -0.227262   1.666727
H  -1.022028   1.175191  -0.725532
H  -0.724686  -0.292472   0.231149
S  -0.653236   1.620897   1.638837
H  -3.075007  -1.377897   1.169891
H  -2.599652  -0.143458   2.240691
H  -1.298197   2.770668   1.354599
C   1.845709  -1.889474   0.234110
C   2.231401  -0.375736   0.266377
O   2.806966  -2.691195   0.161392
O   0.618438  -2.108529   0.247567
H   1.671826   0.131534   1.047449
C   1.956566   0.253789  -1.096165
N   3.693789  -0.265510   0.608161
H   3.845135  -0.277079   1.617676
H   0.937681   0.014079  -1.387778
H   2.624409  -0.164995  -1.851835
S   2.219637   2.077850  -1.148750
H   4.146025  -1.110129   0.225415
H   4.118823   0.588027   0.234491
H   1.343788   2.371241  -0.162122
28
Dimer 171 of the z...z  type
C   3.009093  -0.861231   1.208613
C   1.893268  -0.022438   0.496774
O   3.606444  -0.264659   2.137114
O   3.153424  -2.025012   0.788708
H   0.953921  -0.255306   1.002728
C   1.746087  -0.295557  -0.993415
N   2.162341   1.431466   0.760139
H   1.304007   1.990593   0.685892
H   0.960068   0.321167  -1.431127
H   1.489297  -1.343752  -1.125453
S   3.268621   0.088522  -1.959593
H   2.581143   1.492918   1.697929
H   2.853214   1.787628   0.091144
H   4.033444  -0.849150  -1.362629
C  -3.536905  -1.363219  -0.184225
C  -3.121689   0.133249  -0.403535
O  -4.372222  -1.554199   0.733126
O  -2.952166  -2.178484  -0.918903
H  -3.137174   0.378110  -1.463276
C  -1.728413   0.344085   0.181544
N  -4.137910   0.997876   0.294552
H  -4.935293   1.196838  -0.310529
H  -1.054732  -0.339071  -0.331380
H  -1.716292   0.105388   1.246808
S  -1.097332   2.074547   0.068488
H  -4.491278   0.432607   1.084632
H  -3.751512   1.891181   0.612996
H  -1.052925   2.145843  -1.277776
28
Dimer 172 of the z...z  type
C  -3.568928  -0.933322   0.117241
C  -2.843155   0.355250  -0.396366
O  -4.402291  -0.739630   1.038546
O  -3.202709  -1.996246  -0.414435
H  -2.770796   0.349900  -1.481843
C  -1.455721   0.428880   0.236399
N  -3.677908   1.540729   0.012040
H  -4.402447   1.748135  -0.675671
H  -0.873381  -0.426287  -0.106877
H  -1.528710   0.399706   1.325935
```

```
S  -0.531397   1.981294  -0.141304
H  -4.153680   1.259813   0.885220
H  -3.109682   2.383960   0.143294
H  -0.411400   1.771686  -1.467998
C   1.663352  -1.908270  -0.355426
C   2.145091  -0.600144   0.352313
O   2.427760  -2.896225  -0.233481
O   0.600162  -1.797043  -0.994996
H   1.328390  -0.126243   0.891041
C   2.713147   0.354310  -0.695886
N   3.203579  -0.978446   1.353149
H   2.792492  -1.258169   2.244104
H   1.920495   0.580424  -1.405583
H   3.536849  -0.115915  -1.236800
S   3.401078   1.918286  -0.006618
H   3.682800  -1.806747   0.966924
H   3.859018  -0.210159   1.529593
H   2.229365   2.373576   0.484358
28
Dimer 173 of the z...z  type
C   1.786943   0.648263   1.156060
C   2.184423   0.788341  -0.353650
O   2.371224   1.444413   1.930446
O   0.925568  -0.215319   1.404734
H   1.461774   1.464065  -0.814641
C   2.208263  -0.526648  -1.120379
N   3.514716   1.488950  -0.395944
H   3.653743   2.063736  -1.226802
H   2.463360  -0.370304  -2.169300
H   1.218869  -0.972232  -1.062408
S   3.441219  -1.732310  -0.468284
H   3.547753   2.060620   0.465600
H   4.274305   0.801327  -0.336814
H   2.844089  -1.882562   0.732492
C  -2.887348  -0.509234   1.098349
C  -1.806332  -0.673015  -0.021291
O  -3.717376  -1.452543   1.168367
O  -2.793808   0.513850   1.800869
H  -0.927139  -1.126196   0.438960
C  -1.376367   0.625830  -0.682606
N  -2.358685  -1.656778  -1.018690
H  -1.648548  -2.230410  -1.472520
H  -0.644068   0.440211  -1.469036
H  -0.924190   1.251124   0.082066
S  -2.752731   1.537183  -1.505167
H  -3.019455  -2.239205  -0.474110
H  -2.901737  -1.168981  -1.738692
H  -3.449137   1.761187  -0.371366
28
Dimer 174 of the z...z  type
C   3.417557   0.810934   0.366146
C   3.564341  -0.430082  -0.578208
O   3.873921   0.652695   1.527890
O   2.895962   1.814091  -0.150608
H   4.407182  -0.229836  -1.242399
C   2.325889  -0.726988  -1.415455
N   3.950376  -1.602729   0.283568
H   4.596683  -2.252034  -0.163446
H   2.509127  -1.551077  -2.106307
H   2.085123   0.166119  -1.987632
S   0.861881  -1.229102  -0.414933
H   4.362686  -1.178768   1.134449
H   3.108837  -2.113028   0.576073
H   0.678958  -0.047858   0.219570
C  -1.440059   1.501691   0.179337
C  -2.822073   0.792121   0.351753
O  -1.242956   2.034470  -0.937684
O  -0.674488   1.397386   1.158623
H  -3.272721   1.062055   1.304647
```



```
C  -2.620839  -0.719334   0.256554
N  -3.729135   1.272913  -0.748993
H  -4.213646   2.132572  -0.489982
H  -1.941817  -1.020122   1.051772
H  -2.162873  -0.984224  -0.698114
S  -4.173826  -1.707683   0.344728
H  -3.114069   1.498617  -1.547064
H  -4.433968   0.572449  -1.000838
H  -4.535481  -1.316799   1.583999
28
Dimer 175 of the z...z  type
C   3.271864   0.908336  -0.519620
C   3.169477  -0.604949  -0.916903
O   4.359358   1.239795   0.016399
O   2.279750   1.602082  -0.801807
H   3.422951  -0.677082  -1.976457
C   1.796674  -1.222498  -0.692803
N   4.251164  -1.333440  -0.163359
H   4.640378  -2.130647  -0.666347
H   1.782148  -2.274342  -0.980564
H   1.078286  -0.678867  -1.301390
S   1.255614  -1.168928   1.071134
H   4.972613  -0.613437   0.021775
H   3.906726  -1.653522   0.747081
H   1.082205   0.169745   1.132322
C  -1.462053   1.454684   0.461842
C  -2.227565   0.181054  -0.024114
O  -0.977477   1.396773   1.617402
O  -1.443024   2.386744  -0.364853
H  -1.791221  -0.159980  -0.962837
C  -3.710919   0.486756  -0.211489
N  -2.035652  -0.925981   0.972966
H  -1.050828  -1.244546   0.965012
H  -3.798679   1.310119  -0.916791
H  -4.163814   0.796374   0.733144
S  -4.717396  -0.948540  -0.789262
H  -2.208664  -0.568238   1.916455
H  -2.655227  -1.718356   0.778895
H  -4.110658  -1.085251  -1.986096
28
Dimer 176 of the z...z  type
C  -1.105759   1.346810  -0.046979
C  -2.026904   0.116603   0.223655
O  -1.125035   2.248542   0.819235
O  -0.468658   1.289673  -1.121352
H  -1.477050  -0.809273   0.063973
C  -3.243513   0.180972  -0.698072
N  -2.450332   0.161162   1.667636
H  -1.765487  -0.290625   2.274076
H  -2.886999   0.173601  -1.726012
H  -3.799112   1.106710  -0.534180
S  -4.467117  -1.173169  -0.440479
H  -2.480446   1.156926   1.935011
H  -3.356493  -0.295187   1.816106
H  -3.636923  -2.188684  -0.754718
C   2.432153  -2.036607  -0.125520
C   2.030494  -0.545474   0.093788
O   3.102562  -2.556955   0.801679
O   2.073454  -2.521737  -1.215457
H   0.991453  -0.382877  -0.178013
C   2.929499   0.362519  -0.745719
N   2.190783  -0.225356   1.556624
H   1.357992  -0.472060   2.090763
H   2.766224   0.114643  -1.792714
H   3.981442   0.188246  -0.510145
S   2.636476   2.163682  -0.468807
H   2.962135  -0.816638   1.901961
H   2.383194   0.772899   1.700538
H   1.312766   2.118347  -0.787665
```

```
28
Dimer 177 of the z...z  type
C  -1.539509   1.392492   0.160640
C  -3.007704   0.905162  -0.087487
O  -0.894113   1.670633  -0.885618
O  -1.183731   1.459508   1.347835
H  -3.658074   1.776593   0.012550
C  -3.473574  -0.183175   0.869895
N  -3.087325   0.461946  -1.523410
H  -3.998837   0.612483  -1.954749
H  -4.511654  -0.459497   0.679670
H  -3.390518   0.201569   1.883493
S  -2.505621  -1.747232   0.724942
H  -2.346942   0.996075  -2.010891
H  -2.846161  -0.533336  -1.595238
H  -1.315826  -1.208827   1.064498
C   2.882113   1.018823   0.302424
C   3.242342  -0.484369   0.044718
O   2.632326   1.304423   1.501564
O   2.921533   1.757785  -0.696898
H   4.330825  -0.542397  -0.016293
C   2.636802  -1.068446  -1.225466
N   2.828089  -1.254605   1.269022
H   3.445666  -2.031431   1.501583
H   3.014846  -2.074501  -1.412259
H   2.918427  -0.425021  -2.055804
S   0.801840  -1.234599  -1.159033
H   2.802526  -0.546744   2.025750
H   1.871389  -1.608971   1.145390
H   0.501638   0.083484  -1.067876
28
Dimer 178 of the z...z  type
C   3.382664   0.851496   0.438266
C   2.756595  -0.317091  -0.396050
O   4.061717   0.489589   1.431588
O   3.095196   1.994067   0.040448
H   2.888380  -0.137573  -1.460792
C   1.276504  -0.436665  -0.048526
N   3.494555  -1.580260  -0.037579
H   4.327620  -1.704137  -0.613701
H   0.786277   0.500677  -0.308022
H   1.150483  -0.615486   1.021813
S   0.405170  -1.839844  -0.876435
H   3.813263  -1.449953   0.937056
H   2.910420  -2.416185  -0.136880
H   0.644221  -1.443307  -2.143631
C  -1.727045   2.019185  -0.237106
C  -2.668214   0.767879  -0.208243
O  -0.808047   1.969528  -1.096826
O  -1.967413   2.885545   0.619507
H  -3.710751   1.075863  -0.175228
C  -2.323154  -0.078708   1.017638
N  -2.436579   0.007981  -1.486570
H  -3.139836   0.224139  -2.192641
H  -2.556894   0.506184   1.904729
H  -1.259674  -0.310665   1.037142
S  -3.163491  -1.714938   1.092441
H  -1.518066   0.352098  -1.826207
H  -2.419851  -1.006495  -1.344656
H  -4.423998  -1.239099   1.160358
28
Dimer 179 of the z...z  type
C  -3.639934  -0.141305  -1.155340
C  -3.125558   0.242903   0.272525
O  -3.338905  -1.299954  -1.535711
O  -4.243806   0.765078  -1.757197
H  -3.904296   0.752156   0.835910
C  -1.893515   1.134093   0.127756
N  -2.780236  -1.030512   0.994595
```



```
H  -3.593418  -1.425840   1.466702
H  -2.186626   2.032138  -0.411881
H  -1.125657   0.622221  -0.452097
S  -1.077824   1.592039   1.715772
H  -2.488445  -1.699214   0.264125
H  -2.034388  -0.898510   1.684793
H  -2.120965   2.269527   2.237268
C   3.714423   0.704557  -0.944982
C   3.010999  -0.590302  -0.411248
O   4.491471   1.262161  -0.130597
O   3.383286   1.043631  -2.094648
H   3.062134  -1.383381  -1.154260
C   1.560334  -0.261744  -0.069003
N   3.758040  -1.042052   0.815604
H   4.526045  -1.670259   0.578149
H   1.081282   0.104745  -0.974807
H   1.514762   0.524330   0.687532
S   0.600267  -1.671180   0.631635
H   4.177880  -0.183776   1.212573
H   3.156968  -1.519337   1.493602
H   0.634130  -2.447128  -0.470562
28
Dimer 180 of the z...z  type
C  -3.916124  -0.726405   0.519594
C  -2.459798  -0.269782   0.148103
O  -4.393050  -1.628137  -0.218018
O  -4.427939  -0.131398   1.481435
H  -1.819663  -0.376286   1.021218
C  -2.475181   1.173299  -0.339519
N  -1.973811  -1.216921  -0.921500
H  -1.274752  -1.874514  -0.577973
H  -2.950652   1.781250   0.427046
H  -3.063052   1.263916  -1.255492
S  -0.823987   1.895570  -0.737447
H  -2.842101  -1.745584  -1.169578
H  -1.587011  -0.748948  -1.742099
H  -0.322811   1.866338   0.516576
C   2.295163   1.518774   0.570680
C   2.094390   0.036870   0.119883
O   2.828410   2.268064  -0.280970
O   1.907532   1.772851   1.728162
H   1.160449  -0.346841   0.521670
C   3.266906  -0.820956   0.582265
N   1.984293   0.017270  -1.378216
H   1.076689   0.406729  -1.658823
H   3.343241  -0.744615   1.665501
H   4.200700  -0.461642   0.145326
S   3.138549  -2.594759   0.088920
H   2.707925   0.640243  -1.756926
H   2.083107  -0.928589  -1.759473
H   2.029816  -2.868736   0.806567
28
Dimer 181 of the z...z  type
C  -3.214008   0.328946  -1.150505
C  -1.919114   0.437628  -0.274818
O  -3.681734   1.429464  -1.537169
O  -3.611477  -0.827534  -1.379726
H  -1.070867   0.301592  -0.947592
C  -1.832511  -0.584521   0.848864
N  -1.851274   1.849592   0.241382
H  -0.902899   2.200403   0.372825
H  -0.909459  -0.472827   1.419069
H  -1.849550  -1.575866   0.404603
S  -3.197699  -0.436923   2.080153
H  -2.368469   2.414060  -0.454737
H  -2.358921   1.923108   1.130040
H  -4.184315  -0.744855   1.212790
C   3.801701   1.114857   0.151691
C   3.282180  -0.256885  -0.397253
```

```
O   4.205813   1.092748   1.340958
O   3.705884   2.067124  -0.642794
H   3.675368  -0.444186  -1.394070
C   1.755404  -0.235350  -0.413330
N   3.784213  -1.338247   0.521562
H   4.704250  -1.676658   0.239012
H   1.438512   0.571526  -1.071807
H   1.372089  -0.028844   0.588132
S   0.962432  -1.819514  -0.924323
H   3.893753  -0.887033   1.445605
H   3.148589  -2.141213   0.563372
H   1.455820  -1.838080  -2.179168
28
Dimer 182 of the z...z  type
C  -4.147917  -0.491119  -0.202813
C  -3.164799   0.507028   0.502780
O  -4.571886  -1.418002   0.531633
O  -4.408243  -0.237390  -1.392655
H  -3.764343   1.346537   0.861323
C  -2.052941   1.039978  -0.387931
N  -2.629878  -0.192168   1.723981
H  -2.395553   0.435817   2.492770
H  -1.398493   1.717215   0.161201
H  -2.511084   1.580643  -1.212534
S  -0.948004  -0.266293  -1.074040
H  -3.371371  -0.860335   1.996284
H  -1.801411  -0.745403   1.485243
H  -1.910766  -0.927664  -1.748518
C   3.947675  -1.086312  -0.226179
C   2.623299  -0.251598  -0.157522
O   4.135909  -1.867349   0.741367
O   4.668185  -0.849525  -1.211044
H   2.088903  -0.322367  -1.101936
C   2.954808   1.202210   0.166958
N   1.767256  -0.863790   0.919560
H   1.075642  -1.508600   0.534982
H   3.621492   1.576939  -0.606792
H   3.470621   1.272670   1.127106
S   1.487949   2.310481   0.320957
H   2.435569  -1.407948   1.499167
H   1.263090  -0.166583   1.472847
H   1.044468   2.150772  -0.942767
28
Dimer 183 of the z...z  type
C  -2.711161   1.654096  -0.377218
C  -1.956700   0.294161  -0.188249
O  -2.535548   2.487119   0.546181
O  -3.371670   1.760559  -1.427262
H  -1.006094   0.384758  -0.718946
C  -2.697604  -0.926739  -0.713515
N  -1.625224   0.153263   1.269710
H  -0.790066  -0.423533   1.420832
H  -2.108332  -1.833749  -0.571387
H  -2.874159  -0.785151  -1.776903
S  -4.307117  -1.228590   0.135445
H  -1.498024   1.110462   1.630182
H  -2.417908  -0.261929   1.770624
H  -4.887687  -0.085121  -0.284138
C   4.473189   0.417580  -0.322554
C   3.215441  -0.211214   0.382842
O   4.694636   1.619258  -0.024182
O   5.112509  -0.349763  -1.060490
H   3.585180  -0.848022   1.187112
C   2.346412  -1.010580  -0.571851
N   2.469312   0.924399   1.035760
H   2.106253   0.699089   1.960989
H   2.975239  -1.755470  -1.052946
H   1.928265  -0.369274  -1.350446
S   0.895402  -1.848766   0.203705
```



```
H   3.178956   1.685504   1.077049
H   1.694644   1.261144   0.462173
H   1.575681  -2.525845   1.150404
28
Dimer 184 of the z...z  type
C  -1.680719   0.460984   1.046062
C  -3.200336   0.368008   0.724996
O  -1.109243   1.527289   0.735942
O  -1.181722  -0.552939   1.596035
H  -3.743561   0.578196   1.648793
C  -3.641033  -0.986075   0.178554
N  -3.512160   1.484670  -0.233042
H  -4.450567   1.869671  -0.123816
H  -4.718567  -1.008256   0.012270
H  -3.386121  -1.749232   0.909618
S  -2.881588  -1.410352  -1.446386
H  -2.793498   2.208796  -0.068771
H  -3.397826   1.155813  -1.199551
H  -1.616056  -1.523696  -0.994541
C   3.387291  -1.080180   0.054652
C   1.887860  -0.635111   0.032075
O   3.860175  -1.314255   1.195095
O   3.938511  -1.183974  -1.059943
H   1.297316  -1.530308  -0.182607
C   1.552765   0.421311  -1.014515
N   1.496139  -0.187481   1.405329
H   0.467388  -0.346494   1.594989
H   0.493527   0.676011  -0.981733
H   1.801547   0.030473  -1.997823
S   2.430926   2.022683  -0.758573
H   2.104990  -0.695862   2.058984
H   1.695484   0.811185   1.512793
H   3.671956   1.543500  -0.983241
28
Dimer 185 of the z...z  type
C  -1.616033  -1.360679  -0.120821
C  -3.074608  -0.824198  -0.150539
O  -1.139754  -1.632405   1.000255
O  -1.070414  -1.478294  -1.247575
H  -3.708666  -1.623831  -0.537880
C  -3.259304   0.425613  -1.013227
N  -3.482620  -0.574561   1.274843
H  -4.453713  -0.812455   1.475113
H  -4.319079   0.652030  -1.135509
H  -2.831200   0.224853  -1.992455
S  -2.514804   1.952072  -0.298249
H  -2.832769  -1.131681   1.855096
H  -3.322302   0.417484   1.500917
H  -1.197556   1.595974  -0.384949
C   1.216444   0.728784   0.570527
C   1.866341  -0.669618   0.364543
O   0.709347   1.262352  -0.451397
O   1.282281   1.176274   1.731873
H   1.365025  -1.367142   1.036566
C   3.362964  -0.664973   0.666291
N   1.583676  -1.145850  -1.027833
H   0.539997  -1.333263  -1.142997
H   3.787665  -1.664218   0.553591
H   3.504772  -0.349284   1.697800
S   4.372397   0.411632  -0.442578
H   1.838979  -0.412843  -1.692834
H   2.100154  -1.995784  -1.255791
H   3.873310   1.590790  -0.018400
28
Dimer 186 of the z...z  type
C  -3.086130  -1.169035  -0.083952
C  -3.399415   0.262415  -0.638541
O  -3.667055  -1.457999   0.995698
O  -2.333495  -1.864015  -0.786446
```

H	-4.088688	0.136212	-1.475676
C	-2.175167	1.038333	-1.117505
N	-4.145986	0.993139	0.444725
H	-4.924135	1.559418	0.109490
H	-2.472048	1.950829	-1.635762
H	-1.625397	0.406209	-1.810994
S	-1.055888	1.589701	0.238118
H	-4.466794	0.231458	1.076930
H	-3.500000	1.586424	0.979270
H	-0.534753	0.377408	0.570913
C	1.696026	-1.286490	0.297515
C	3.174552	-0.838487	0.550012
O	1.518280	-1.991617	-0.724669
O	0.869245	-0.914710	1.154705
H	3.574327	-1.479382	1.338457
C	3.331310	0.620106	0.970654
N	3.941646	-1.150807	-0.705291
H	4.886569	-1.496876	-0.543363
H	4.360333	0.833113	1.263161
H	2.682147	0.797338	1.824950
S	2.946127	1.836081	-0.360574
H	3.356014	-1.855477	-1.193650
H	3.985735	-0.318189	-1.305445
H	1.610389	1.628352	-0.398419
